# Cationic Selenuranes – Bench‐Stable Sources of Se(III) Radicals

**DOI:** 10.1002/anie.202513534

**Published:** 2025-09-16

**Authors:** Kirill Zhiliaev, Boris Maryasin, Hanspeter Kählig, Marcos Gil‐Sepulcre, Javier Mateos

**Affiliations:** ^1^ Institute of Organic Chemistry University of Vienna Währinger Straße 38 Vienna 1090 Austria; ^2^ Institute of Theoretical Chemistry University of Vienna Währinger Straße 17 Vienna 1090 Austria; ^3^ Departament de Química Universitat Autònoma de Barcelona Cerdanyola del Vallès Barcelona 08193 Spain

**Keywords:** Chalcogen, Main‐group, Radical, Selenium

## Abstract

Radicals are often considered unstable and synthetically unpredictable. Stable radicals, defined as species that can be stored and handled under ambient conditions, serve as valuable reagents for redox and radical‐mediated transformations. We report the multigram‐scale synthesis, isolation, and characterization of cationic selenuranes–formally λ^4^‐selane species–as bench‐stable reservoirs of one‐center/one‐electron selenium radical cations. Solid‐state stability arises from a selective, reversible head‐to‐head Se–Se oligomerization, with the salts remaining stable under air and moisture for over a month. In solution, electrostatic repulsion weakens the Se─Se σ‐bond (Δ*H*_298_ = 29.9 kcal·mol^−1^), enabling dissociation into radical cations without external activation. The resulting Se(III) radicals promote oxidation and substitution reactions with hydrazines, alcohols, sulfinates, borates, silanes, and stannanes, including functionalizations of complex molecules.

## Graphical abstract

We present the synthesis of cationic selenurane salts via oligomerization of dibenzoselenophene‐based radical cations, allowing the formation of radicals without external activation. The salts can be prepared on a multigram scale and stored as solids for more than a month at ambient conditions. The salt structure has been analyzed in the solid‐state and the radical cation properties in solution. Direct access to Se(III) radicals allows their use as reagents.

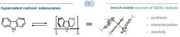

Organic radicals, particularly stable radicals, play an important role as reagents in organic synthesis, promoting redox reactions and serving as polymer initiators.^[^ ^1^, ^2^ ^]^ In 1900, Gomberg prepared the triphenylmethyl radical,^[^ ^3^ ^]^ which persists in solution for several days due to dimerization (*K*_ass_ ≈ 3.0 × 10^3^ M^−1^ at 293 K, Figure 1a).^[^ ^4^, ^5^ ^]^ Schlenk,^[^ ^6^ ^]^ Ziegler,^[^ ^7^ ^]^ Marvel,^[^ ^8^ ^]^ and others, studied the reversible dimerization, though the structure of the dimer remained debated. Lankamp et al. later used NMR spectroscopy to confirm a quinoid‐type dimer structure via head‐to‐tail dimerization (σ dimer, Figure 1a right).^[^ ^9^, ^10^, ^11^ ^]^ Dimerization is not restricted to C‐centered radicals. Conjugated aromatic systems containing heteroatoms (such as O, N, or S) can dimerize, oligomerize, or polymerize–often irreversibly.^[^ ^12^, ^13^ ^]^ Bontempelli and co‐workers demonstrated that anodic oxidation of dibenzothiophene leads to an irreversible dimerization–deprotonation sequence, yielding a sulfonium salt (Figure 1b).^[^ ^14^ ^]^ In this case, due to the C─S bond formation, radical cation regeneration is not possible. Consequently, synthetic applications of chalcogen‐centered (Ch) radicals have focused on systems where the spin density is delocalized across multiple heteroatoms, such as tetrathiafulvalene, phenothiazine, thianthrene, or 1,2,4‐trithia‐3,5‐diazolyl radical cations.^[^ ^15^, ^16^, ^17^, ^18^ ^]^ In contrast, one‐center/one‐electron (1c1e) Ch(III) radicals have hitherto been challenging to access, and no bench‐stable alternatives have been reported to date.^[^ ^19^ ^]^ A reversible oligomerization mechanism would allow the use of heterocyclic radicals with a single heteroatom, unlocking chalcogen radicals for new applications.

**Figure 1:**
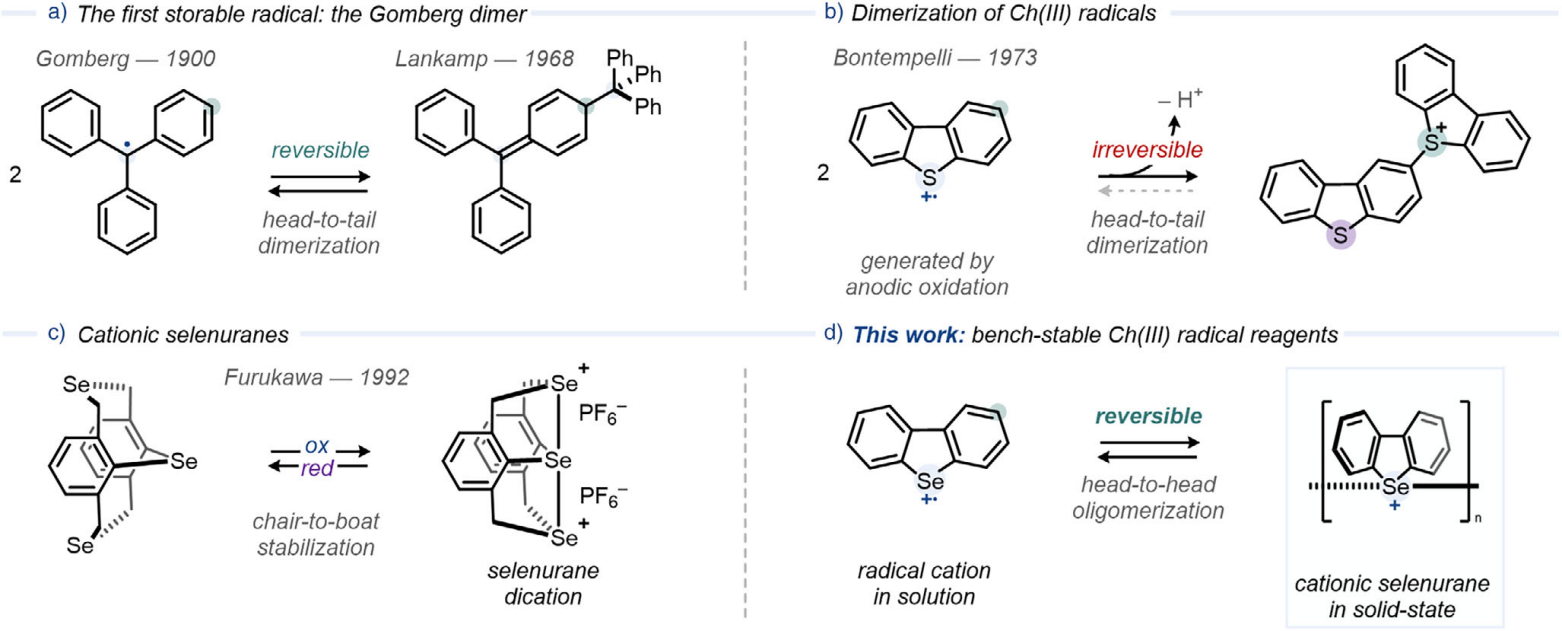
a) Reversible dimerization of the Gomberg radical forms a quinoid‐type σ‐dimer. b). Irreversible dimerization–deprotonation of dibenzothiophene radical cations forms sulfonium salts. c). Selenurane dication stabilization by transannular chair–boat stabilization equilibria. d). **This work**: reversible oligomerization of dibenzoselenophene‐based radical cations enables the multi‐gram preparation of bench‐stable Se(III) radical precursors.

Head‐to‐head dimerization of aryl selenide radicals via Se─Se bond formation between two independent radical subunits enables the generation of chalcogen‐centered radicals. Diselenides serve as 1c1e radical precursors, yet Ch─Ch bond homolysis requires external activation due to high bond dissociation energies (BDE PhSe–SePh = 66.9 ± 4.5 kcal·mol^−1^).^[^ ^20^ ^]^ Electrostatic repulsion in polycationic structures weakens Ch─Ch bond strength.^[^ ^21^, ^22^ ^]^ Di‐ and tri‐selenide cation salts were first reported by Furukawa via the two‐electron oxidation of cyclic alkyl selenides.^[^ ^23^ ^]^ These dications feature an intramolecular Se─Se σ‐bond, yet radical cation generation in solution is precluded by transannular chair–boat stabilization equilibria (Figure 1c).^[^ ^24^ ^]^ A rigid polycationic Se─Se bond connecting two discrete subunits, would instead enable radical generation without external activation.

Herein, we report the synthesis of bench‐stable cationic selenurane salts via oligomerization of dibenzoselenophene‐based radical cations, allowing the formation of chalcogen radicals without external activation (Figure 1d). The salts can be prepared on a multigram scale (>17 g) in two steps from commercial materials. Due to their kinetic stability, the salts can be stored as solids for over six months at –35 °C under inert conditions or for more than a month at ambient conditions without excluding moisture or air. In solution, the electrostatic repulsion of the Se─Se σ‐bond (dimerization enthalpy, Δ*H*_298_ = 29.9 kcal·mol^−1^)^[^ ^25^ ^]^ promotes dissociation to generate diarylorganoselenium radical cations. The salt structure has been analyzed in the solid‐state and the radical cation properties in solution. Direct access to Se(III) radicals without external activation allows their use as oxidants and group transfer reagents.

We identified in the literature that 1c1e selenium radical cations are persistent in solution, yet storable analogues remain rare.^[^ ^19^ ^]^ Efforts to stabilize Ch(III) radicals have typically involved introducing steric hindrance around the chalcogen center. Beckmann and co‐workers, reported the isolation of a stable Te(III) radical from the oxidation of diphenylchalcogenides, whereas analogous S‐ and Se‐centered radical cations remained inaccessible.^[^ ^26^ ^]^ The difficulty in isolating stable selenium radicals prompted us to consider whether the solid‐state structure contributes significantly to their stability. We hypothesized that stability arises not only from the solution‐phase structure but also from the solid‐state arrangement of the reagent. Selenium supports high‐order assemblies in the solid state through chalcogen bonding and accommodates λ^4^‐selane structures.^[^ ^27^, ^28^ ^]^ We propose the following design elements considering both solution‐phase and solid‐state features: i) planarity restricts aryl group rotation and promotes π–π interactions between Ch(III) subunits (purple highlight); ii) the choice of chalcogen dictates the extent of oligomerization, as spin density increases down the group (O < S < Se, gray highlight) and minimizes radical delocalization (gray highlight);^[^ ^29^ ^]^ and iii) substitution at C4 suppress head‐to‐tail dimer formation (green highlight).^[^ ^4^, ^5^ ^]^

Cationic selenurane **2a** is prepared through the oxidation of dibenzoselenophene (**1a**) with nitrosonium salts (Figure 2b). Upon addition of NOSbF_6_ to a solution of **1a**, an immediate color change to dark blue occurs (Figure S2). The blue color is attributed to chalcogen radical cations in solution.^[^ ^26^, ^30^ ^]^ Se(III) radical cations exhibit high reactivity toward oxygen‐containing solvents. In the presence of water (5.0 equiv.) or ethereal solvents (such as tetrahydrofuran, diethyl ether, or 1,4‐dioxane), the solution fades, yielding undesired sideproducts (Figures S8–S11). Addition of pentane to the Se(III) radical solution induces the formation of a brown crystalline precipitate. Filtration under atmospheric conditions provides **[2a]SbF_6_** in 93% yield. The reaction is scalable to a multigram scale (up to 40 mmol) without yield erosion (1.0 mmol scale 93% yield, versus 40 mmol scale 93% yield). Different nitrosonium salts facilitate the oxidation of **1a** (Figure 2c), affording cationic selenuranes with various counterions (i.e., **[2a]PF_6_** and **[2a]BF_4_**). The synthesis of C3‐ (**2b**), C4‐ (**2c**), and C3‐ and C4‐substituted (**2d**) salts is also achieved with the same procedure in multigram scale. Attempts to oxidize C2‐substituted or electron‐deficient selenides with nitrosonium salts resulted in 74% recovery of the starting material, likely due to steric hindrance near the chalcogen center and instability of the corresponding radical cations. Attempts to oxidize diphenyl‐ and dibenzylselenide using this method resulted in complex reaction mixtures or starting material recovery, and oxidation of sulfur analogues yielded the head‐to‐tail dimer (Figure S5).^[^ ^14^ ^]^

**Figure 2:**
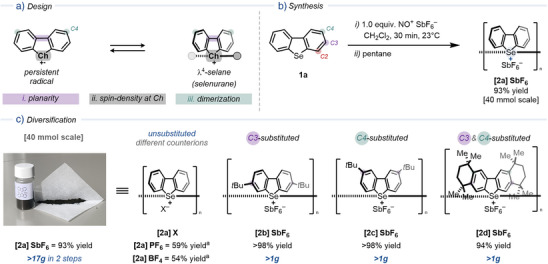
a). Initial design based on previous works. b) Synthesis of **[2a]SbF_6_**. c) Diversification of the salt structure to avoid undesired “head‐to‐tail” dimerization. ^a^1.5 mmol scale.

To understand the initiation of the oligomerization over head‐to‐tail dimers–and to clarify the role of selenium versus sulfur–we employed density functional theory (DFT) calculations in the presence and absence of counterions (Figure 3a).^[^ ^25^ ^]^ The irreversible head‐to‐tail dimerization is observed only for the sulfur analogue (**S–5a**, gray dashed lines), consistent with an exergonic process (Δ*G* = –13.1 kcal·mol^−1^) involving spontaneous counterion‐mediated deprotonation of the Wheland intermediate. In contrast, for selenium (black doted lines), the formation of the Wheland intermediate **I** is endergonic both with counterion (Δ*G* = +1.8 kcal·mol^−1^) and without counterion (Δ*G* = +9.7 kcal·mol^−1^). Moreover, the initial step for the cationic selenurane formation (**Se–3a**, head‐to‐head dimerization) is more exergonic (Δ*G* = –11.5 kcal·mol^−1^ with BF_4_^–^ as counterion; –1.4 kcal·mol^−1^ without) than for the sulfur analogue (**S–3a**, Δ*G* = –0.8 kcal·mol^−1^ and +10.4 kcal·mol^−1^, respectively). These data highlight the decisive role of the chalcogen atom in controlling dimerization selectivity, while the counterion modulates the dynamic equilibrium.

**Figure 3:**
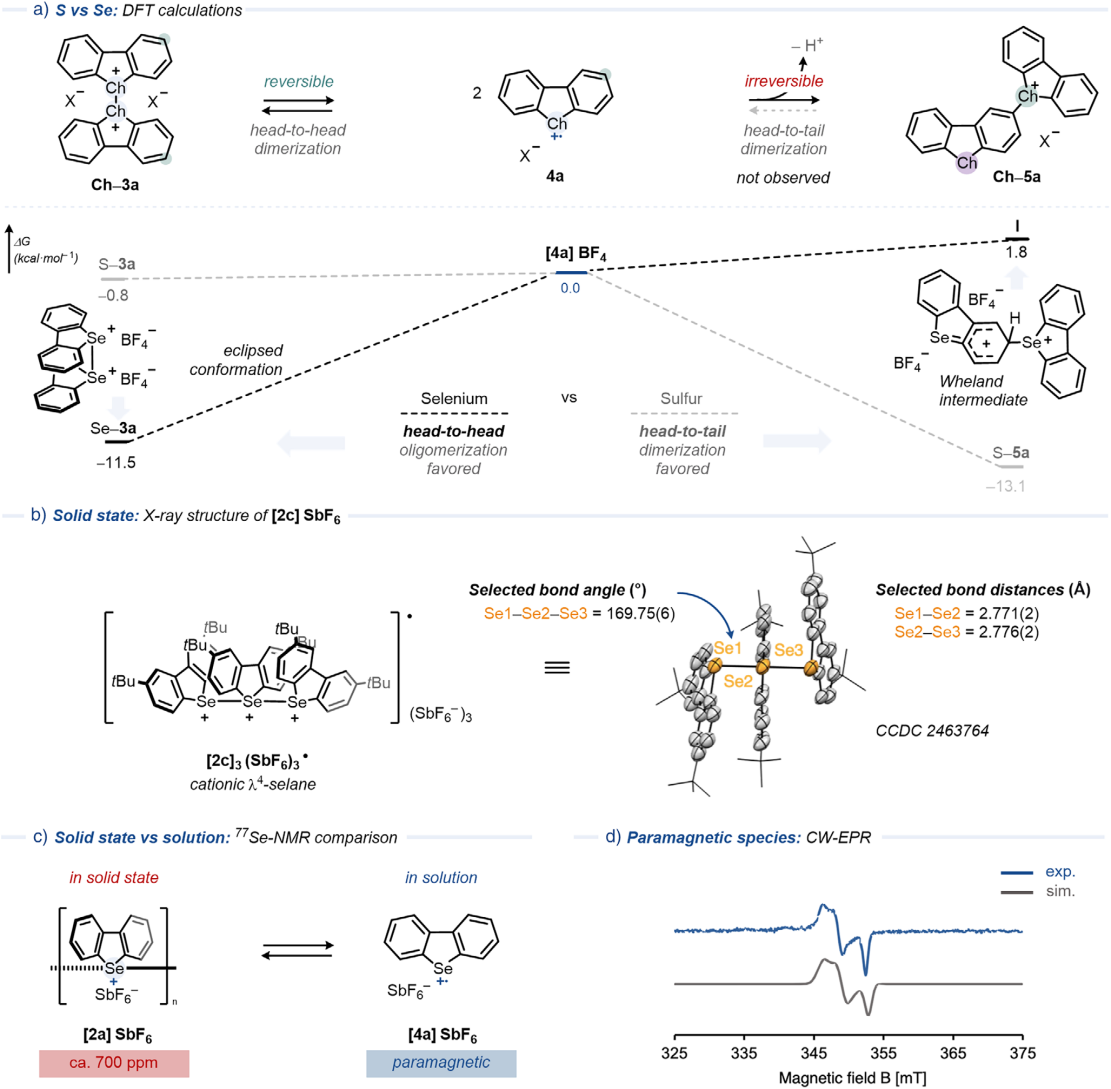
a) Characterization of the dimerization process. The use of selenium in place of sulfur favors head‐to‐head dimerization to form dication **Se–3a** over the head‐to‐tail dimer **Se–5a**. b) Solid‐state structure of **[2c]SbF_6_** determined by X‐ray Diffraction at 50% probability ellipsoids with H atoms and counterions omitted for clarity. c) Comparison of ^77^Se‐NMR spectra of **[2a]SbF_6_** in the solid state and in a CD_2_Cl_2_ solution. d) X‐Band CW‐EPR spectrum of **[2b]SbF_6_** at 298 K (blue: experimental, gray: simulated).

The Se─Se bond formation and the λ^4^‐selane structure of reagents **2** was confirmed by single‐crystal X‐ray diffraction (SC‐XRD, Figure 3b).^[^ ^31^ ^]^ The minimal cationic selenurane unit corresponds to a trimeric structure, formulated as the tricationic radical **[2c]_3_^·^(SbF_6_)_3_**. The solid‐state structure features a linear triatomic Se array flanked by eclipsed aromatic rings and three SbF_6_^–^ counterions. The Se1─Se2 and Se2─Se3 bond lengths are nearly identical (2.771(2) and 2.776(2) Å, respectively). Both distances exceed typical values for Se(0)─Se(0) or PhSe─SePh bonds (2.33 Å),^[^ ^32^ ^]^ but remain shorter than 1,2,4‐triseleno‐3,5‐diazolium dicationic dimers (3.12 Å)^[^ ^33^ ^]^ and π−π stacked Se(II)⋯Se(II) distances (4.55 Å) via chalcogen–chalcogen interactions,^[^ ^34^ ^]^ consistent with the formation of weak σ‐bonds between Se atoms in selenurane cations due to electrostatic repulsion. The distorted Se1─Se2─Se3 bond angle of 169.75(6)° matches previously reported values for cationic selenuranes,^[^ ^23^, ^35^ ^]^ and contrasts with the nearly linear C─Se─C bond angle of 179.2° reported for tetraphenylselenurane.^[^ ^36^ ^]^

The equilibrium between cationic selenuranes and radical cations was studied experimentally. Selenurane cations **2** were unambiguously identified over head‐to‐tail dimers **5** using nuclear magnetic resonance (NMR) spectroscopy. ^77^Se NMR spectroscopy is a valuable tool for the characterization of organoselenium compounds both in solution and in solid state due to its sensitivity toward electronic changes at Se (Figure 3c and Supporting Information S75–S100).^[^ ^37^ ^]^ In solution, the signal for dibenzoselenophene (**1a**, Se(II)) appears at 450 ppm in CD_2_Cl_2_ while its selenonium salt derivatives (Se(IV)) resonate at 520 ppm. The head‐to‐tail dimer **Se–5a** displays two distinct signals: at 470 ppm (Se(II)) and at 520 ppm (Se(IV)), both of which remain stable over time (>12 h) indicating the persistence of **Se–5a** in solution.^[^ ^38^ ^]^ In the solid‐state, the ^77^Se NMR spectrum of compound **Se–5a** shows a broad average signal at 500 ppm. In contrast, the solid‐state ^77^Se NMR spectrum of the brown crystalline solid (**[2a]SbF_6_**) exhibits multiple signals centered at 700 ppm, consistent with previously reported dicationic Se(III) salts and highlighting the possibility of different oligomeric chain sizes.^[^ ^23^ ^]^ Dissolving **[2a]SbF_6_** in CD_2_Cl_2_ leads to blue solutions as well as signal broadening in ^1^H NMR spectrum. A dynamic equilibrium between **[2c]SbF_6_** and **[4c]SbF_6_** was detected by variable‐temperature ^1^H NMR (298–238 K; Figures S50 and S51) consistent with the calculated dimerization barrier without counterions (Δ*G* = –1.4 kcal·mol^−1^).^[^ ^25^ ^]^

The paramagnetic properties of cationic selenuranes were examined by X‐band continuous‐wave electron paramagnetic resonance (CW‐EPR) spectroscopy at 298 K. In the solid‐state, we anticipate that only uneven oligomeric chains exhibit EPR activity, whereas in solution, Se─Se bond dissociation consistently produces EPR‐active samples. The paramagnetic species, assigned to cationic selenurane **[2b]SbF_6_**, showed a defined g‐tensor of 2.0281, 1.9924, and 2.0134–consistent with the enhanced g‐anisotropy observed for heavy chalcogen radicals due to increased spin–orbit coupling–with no observable hyperfine splitting (Figure 3d).^[^ ^39^ ^]^ The absence of resolved ^77^Se hyperfine splitting is likely due to the low natural abundance of ^77^Se (*I* = ½, 7.6%), with signal intensity falling below the signal‐to‐noise ratio of the recorded spectra, and/or to other line broadening effects. Solution‐phase measurements at 298 K give a single isotropic signal (Figures S52–S63). The rhombic g‐tensor in solid‐state and the *S* = ½ isotropic signal in solution–common for organic radicals–support the assignment of selenium‐centered radicals.^[^ ^26^, ^39^ ^]^ Mulliken spin‐density calculations corroborate the experimental data, indicating localization of the unpaired electron at the selenium atom (0.679).^[^ ^24^ ^]^

Building on the association equilibria in solid‐state and the oxidative properties of Se(III) radicals in solution, we evaluated the long‐term stability of cationic selenuranes **2** as solids over periods ranging from 1 to 180 days (Figure 4a). Solutions containing 1.0 equiv. of radical cation **4a** (i.e., one monomeric subunit) were chemically reduced using (2,2,6,6‐tetramethylpiperidin‐1‐yl)oxyl (TEMPO). Se(III) radicals were quantitatively converted to selenides **1** within minutes. No variation in the yield of **1a** was observed after 180 days of storage at –35 °C under inert atmosphere. Under ambient conditions (23–25 °C, 43% relative humidity, 21% O_2_), **[2a]SbF_6_** exhibited moderate stability, with less than 10% yield loss over one week. After one month, 58% of the selenide was recovered. The main decomposition product was the head‐to‐tail dimer **Se–5a**, formed in 14% yield. Incorporation of *tert*‐butyl groups at the C4 position suppressed this pathway. Accordingly, **[2c]SbF_6_**–bearing *^t^* Bu substituents at C4–remained stable on the bench for over a month.

**Figure 4:**
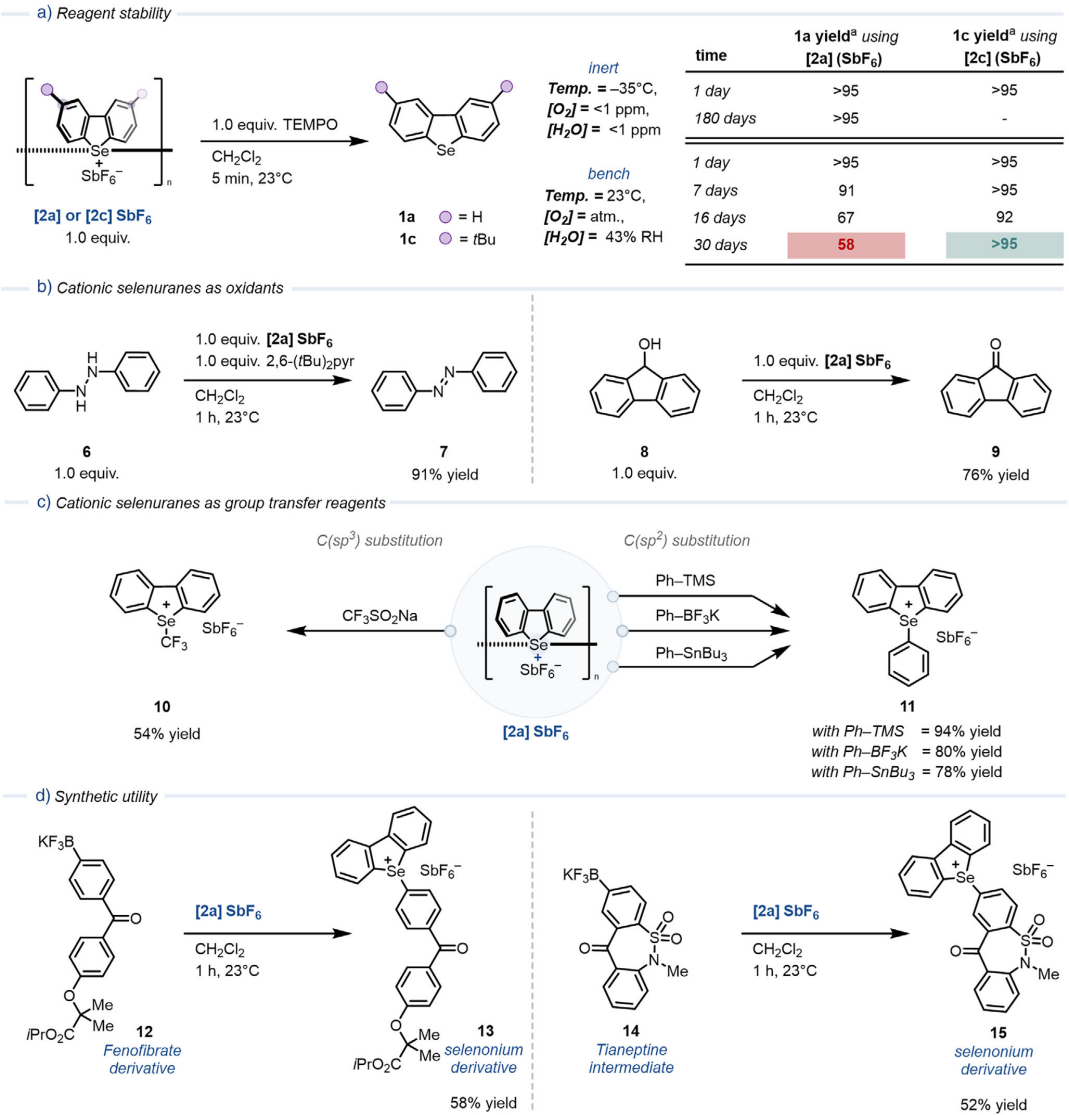
a) Stability assessment of **[2a]SbF_6_** and **[2c]SbF_6_** by chemical reduction to **1**. b) Reactivity of **[2a]SbF_6_** as oxidant for the oxidation of 1,2‐diphenylhydrazine (left) and fluorenol (right). c) Reactivity of **[2a]SbF_6_** with C(*sp* ^3^)‐ (left) and C(*sp* ^2^)‐hybridized (right) nucleophiles. d) Reactivity of **[2a]SbF_6_** with drug derivatives. The Supporting Information provides detailed experimental procedures. ^a^Yield determined by ^1^H NMR spectroscopy using CH_2_Br_2_ as internal standard.

The versatility of cationic selenuranes as oxidants (*E_ox_* (**1a**/**4a**) = +1.53 V versus Ag/AgCl) was further demonstrated beyond the oxidation of TEMPO (Figure 4b). **[2a]SbF_6_** oxidized diphenylhydrazine (**6**) to the corresponding diazo compound **7** in 91% yield, and the alcohol group of fluorenol (**8**) to the ketone derivative **9** in 76% yield. Cationic selenuranes also enabled the transformation of primary alcohols to aldehydes (Figures S15 and S16) giving as byproduct dibenzoselenophenes **1**. All reactions proceeded under ambient conditions, without the need for an inert atmosphere, and tolerated the presence of air.

To further highlight the operational simplicity of Se(III) radicals in synthesis, the use of cationic selenuranes as group transfer reagents was evaluated with C(*sp* ^3^)‐ and C(*sp* ^2^)‐hybridized nucleophiles (Figure 4c). The reactions proceeded on 0.5 mmol scale without exclusion of air or moisture. The selenium analogue of the Umemoto reagent **10** was obtained in a single step from trifluoromethylsulfinate in 54% yield and enabled direct access to electrophilic trifluoromethylating reagents, circumventing the common three‐step sequence involving selenide oxidation, selenoxide activation, and cyclization.^[^ ^40^, ^41^ ^]^ *Ipso*‐substitution of aryl silanes, trifluoroborate salts, and stannanes with **[2a]SbF_6_** furnished arylselenonium salts **11** in 78%–94% yield. Electron‐donating, electron‐withdrawing, and sterically hindered aryl trifluoroborate salts were also compatible, delivering the corresponding products in up to 85% yield (Supporting Information S66–S69). C3‐, C4, and C3,C4‐substituted cationic selenuranes **2b–d** also demonstrated to be efficient group transfer reagents in these transformations (in >98% yield in all cases, Supporting Information S57–S59). Complex molecules were also tolerated (Figure 4d). Fenofibrate (**12**) and tianeptine (**14**) derivatives gave selenonium salts **13** and **15** in 58% and 52% yield, respectively. Although arylselenonium salts have demonstrated catalytic activity as Lewis acids,^[^ ^42^, ^43^ ^]^ their structural diversification has remained limited.^[^ ^44^, ^45^ ^]^ Strategies developed for the derivatization of sulfonium salts proved equally effective for selenonium salts (i.e., C(*sp* ^2^) bromination, thiocyanation, and photochemical activation; see Figure S20).^[^ ^46^, ^47^ ^]^ The sequence of C–Se bond formation, cleavage, and functionalization in addition to the oxidation properties of Se(III) radicals demonstrates the synthetic utility of this class of salts.

In conclusion, we have developed a scalable synthesis of bench‐stable cationic selenuranes from readily available dibenzoselenophene heterocycles, achieving up to 40 mmol scale and 93% yield (>17 grams). Characterization in the solid state and in solution confirms a selective head‐to‐head oligomerization to form λ^4^‐selane structures via a linear array of Se atoms, enabled by increased spin density at selenium relative to lighter chalcogens. The solid‐state stability of these salts allows storage under ambient conditions for over a month without the need to exclude air or moisture. In solution, the salts dissociate to give access to persistent 1c1e Se(III) radicals to introduce a bench‐stable platform for exploring an underutilized oxidation state in organoselenium chemistry. Following initial studies as oxidants and group transfer reagents–and overcoming acidic Se(IV) activation strategies–we anticipate that these systems will broaden the scope, synthetic utility, and catalytic potential of selenium‐based transformations. Ongoing efforts in our laboratories aim to expand the reactivity and application of these compounds.

## Supporting Information

The authors have cited additional references within the Supporting Information.^[48–69]^

## Conflict of Interests

The authors declare no conflict of interest.

## Supporting information



Supporting Information

## Data Availability

The data that support the findings of this study are available from the corresponding author upon reasonable request.
