# Supplementary material for: Cationic Selenuranes – Bench‐Stable Sources of Se(III) Radicals
Source: Angew Chem Int Ed Engl. 2025 Sep 16;64(44):e202513534. doi: 10.1002/anie.202513534 (PMC12559465; doi:10.1002/anie.202513534)
Supplement: Supplementary file 1 — Supporting Information [file ANIE-64-e202513534-s001.pdf]

SUPPORTING INFORMATION

Cationic Selenuranes – Bench-Stable Sources of Se(III) Radicals

Kirill Zhiliaev,[1] Boris Maryasin,[1,2] Hanspeter Kählig,[1] Marcos Gil-Sepulcre,[3] Javier Mateos[1]*

[1.] Institute of Organic Chemistry, University of Vienna, Währinger Straße 38, 1090 Vienna (Austria)

[2.] Institute of Theoretical Chemistry, Faculty of Chemistry, University of Vienna, Währinger Straße 17, 1090 Vienna (Austria)

[3.] Departament de Química, Universitat Autònoma de Barcelona, Cerdanyola del Vallès, 08193 Barcelona (Spain)

*E-mail: javier.mateos@univie.ac.at

Table of Contents

Table of Contents **1**

Materials and Methods **9**

Solvents 9

Chromatography 9

Spectroscopy and instruments 9

Computational details 11

Starting materials 11

Safety statement 12

Author contributions 12

Experimental data – Synthetic procedures **13**

General procedure for the synthesis of cationic selenuranes 13

1.00 mmol scale: 13

40.0 mmol scale: 14

General procedure for the utilization of cationic selenuranes as oxidants 15

0.50 mmol scale: 15

General procedure for the utilization of cationic selenuranes as group-transfer reagents 16

0.50 mmol scale: 16

Preparation of selenides **1a-e**, and **S1–S8** 17

Dibenzo[*b*,*d*]selenophene (**1a**) 17

3,7-Di-*tert*-butyldibenzo[*b*,*d*]selenophene (**1b**) 18

3,3'-Di-*tert*-butyl-1,1'-biphenyl (**S1**) 19

2,8-Di-*tert*-butyldibenzo[*b*,*d*]selenophene (**1c**) 20

Octamethyl-octahydro binaphthalene **S2** 21

Octamethyl-octahydro dinaphthoselenophene **1d** 22

4-(2,6-Diisopropylphenyl)dibenzo[*b*,*d*]selenophene **1e** 23

4,4'-Bis(trifluoromethyl)-[1,1'-biphenyl]-2-amine (**S3**) 24

2-Iodo-4,4'-bis(trifluoromethyl)-1,1'-biphenyl (**S4**) 25

3,7-Bis(trifluoromethyl)dibenzo[*b*,*d*]iodol-5-ium trifluoromethanesulfonate (**S5**) 26

3,7-bis(trifluoromethyl)dibenzo[*b*,*d*]selenophene (**1f**) 27

Diphenylselenide (**S6**) 28

Dibenzylselenide (**S7**) 28

3,7-Di-*tert*-butyldibenzo[*b*,*d*]thiophene (**S8**) 29

PREPARATION OF CATIONIC SELENURANES 2 30
Cationic selenurane **[2a]SbF$_6$** 30
Cationic selenurane **[2a]PF$_6$** 30
Cationic selenurane **[2a]BF$_4$** 31
Cationic selenurane **[2b]SbF$_6$** 32
Cationic selenurane **[2c]SbF$_6$** 32
Cationic selenurane **[2d] SbF$_6$** 33
Failed substrates 35
PREPARATION OF SELENONIUM SALT SE–[5A]OTF 39
Dibenzo[*b*,*d*]selenophene 5-oxide **S6** 39
[2,5'-Bidibenzoselenophen]-5'-ium trifluoromethanesulfonate **Se–[5a]OTf** 39
Stability of cationic selenurane **[2a]SbF$_6$** in different solvents 41
OXIDATIONS WITH CATIONIC SELENURANES – STABILITY TESTS WITH TEMPO 46
Stability test of **[2a]SbF$_6$** – oxidation of TEMPO to the corresponding oxoammonium salt 46
NMR experiment of benzylic alcohol oxidation with formed *in situ* oxoammonium salt 47
Stability of **[2c]SbF$_6$** – oxidation of TEMPO to the corresponding oxoammonium salt 48
OXIDATIONS WITH CATIONIC SELENURANES – FURTHER EXAMPLES 49
(*E*)-1,2-diphenyldiazene (**7**) 49
Fluorenone (**9**) 49
Additional substrates 50
PREPARATION OF SELENONIUM SALTS FROM CATIONIC SELENURANES 52
5-(trifluoromethyl) -dibenzoselenophenium hexafluoroantimonate (**10**) 53
5-(phenyl) -dibenzoselenophenium hexafluoroantimonate (**11**) 54
3,7-Di-*tert*-butyl-5-phenyl-5*H*-dibenzo[*b*,*d*]selenophen-5-ium hexafluoroantimonate (**11b**) 57
2,8-Di-*tert*-butyl-5-phenyl-5*H*-dibenzo[*b*,*d*]selenophen-5-ium hexafluoroantimonate (**11c**) 58
Octamethyl-octahydro dinaphthoselenophen-5-ium hexafluoroantimonate **11d** 59
PREPARATION OF SELENONIUM SALTS FROM CATIONIC SELENURANES – STABILITY TESTS 60
i) O_2 sensitivity experiments 60
ii) Moisture sensitivity experiments 60
PREPARATION OF SELENONIUM SALTS FROM CATIONIC SELENURANES – COMPLEX MOLECULES 61
Bpin fenofibrate derivative **S11** 61
BF_3K fenofibrate derivative **12** 62
Selenonium fenofibrate derivative **13** 62
Bpin tianeptine intermediate **S12** 63
BF_3K tianeptine intermediate **14** 64
Selenonium tianeptine derivative **15** 65

PREPARATION OF SELENONIUM SALTS FROM CATIONIC SELENURANES – ADDITIONAL EXAMPLES 66
5-(4-(*Tert*-butyl)phenyl) -dibenzoselenophenium hexafluoroantimonate (S13) 66
5-(4-Methoxyphenyl)-5*H*-dibenzo[*b*,*d*]selenophen-5-ium hexafluoroantimonate (S14) 66
5-(4-(Trifluoromethyl)phenyl)-5*H*-dibenzo[*b*,*d*]selenophen-5-ium hexafluoroantimonate (S15) 67
5-(*O*-tolyl)-5*H*-dibenzo[*b*,*d*]selenophen-5-ium hexafluoroantimonate (S16) 68
Group transfer reactivity summary 69
PROPOSED RADICAL REACTIVITY OF SE(III) RADICALS 70
Radical trap experiment 70
MODIFICATION OF SELENONIUM SALTS WITH PREVIOUSLY REPORTED METHODS FOR SULFONIUM SALTS 71

EXPERIMENTAL DATA – ADDITIONAL CHARACTERIZATION 75

^{77}SE MAS-NMR SPECTROSCOPY OF CATIONIC SELENURANES 75
^{77}Se MAS-NMR of dibenzo[*b*,*d*]selenophene (1a) 75
^{77}Se MAS-NMR of cationic selenurane [2a]SbF_6 77
^{77}Se MAS-NMR of cationic selenurane [2b]SbF_6 78
^{77}Se MAS-NMR of cationic selenurane [2c]SbF_6 79
^{77}Se MAS-NMR of cationic selenurane [2d]SbF_6 80
^{77}Se MAS-NMR of [2,5'-bidibenzoselenophen]-5'-ium trifluoromethanesulfonate (Se–[5a]OTf) 81
^{77}Se MAS-NMR of 5-(phenyl) -dibenzoselenophenium hexafluoroantimonate (11) 82
NMR SPECTROSCOPY OF CATIONIC SELENURANES IN SOLUTION (^{1}H, ^{13}C, ^{19}F, AND ^{77}SE) 83
^{1}H NMR of radical cation [4a]SbF_6 – *i.e.* [2a]SbF_6 sample dissolved in CD_2Cl_2 83
^{19}F NMR of radical cation [4a]SbF_6 – *i.e.* [2a]SbF_6 sample dissolved in CD_2Cl_2 84
^{1}H NMR of radical cation [4a]PF_6 – *i.e.* [2a]PF_6 sample dissolved in CD_2Cl_2 85
^{19}F NMR of radical cation [4a]PF_6 – *i.e.* [2a]PF_6 sample dissolved in CD_2Cl_2 86
^{1}H NMR of radical cation [4a]BF_4 – *i.e.* [2a]BF_4 sample dissolved in CD_2Cl_2 87
^{19}F NMR of radical cation [4a]BF_4 – *i.e.* [2a]BF_4 sample dissolved in CD_2Cl_2 88
^{1}H NMR of radical cation [4b]SbF_6 – *i.e.* [2b]SbF_6 sample dissolved in CD_2Cl_2 89
^{13}C NMR of radical cation [4b]SbF_6 – *i.e.* [2b]SbF_6 sample dissolved in CD_2Cl_2 90
^{19}F NMR of radical cation [4b]SbF_6 – *i.e.* [2b]SbF_6 sample dissolved in CD_2Cl_2 91
^{77}Se NMR of radical cation [4b]SbF_6 – *i.e.* [2b]SbF_6 sample dissolved in CD_2Cl_2 92
^{1}H NMR of radical cation [4c]SbF_6 – *i.e.* [2c]SbF_6 sample dissolved in CD_2Cl_2 93
^{13}C NMR of radical cation [4c]SbF_6 – *i.e.* [2c]SbF_6 sample dissolved in CD_2Cl_2 94
^{19}F NMR of radical cation [4c]SbF_6 – *i.e.* [2c]SbF_6 sample dissolved in CD_2Cl_2 95
^{77}Se NMR of radical cation [4c]SbF_6 – *i.e.* [2c]SbF_6 sample dissolved in CD_2Cl_2 96
^{1}H NMR of radical cation [4d]SbF_6 – *i.e.* [2d]SbF_6 sample dissolved in CD_2Cl_2 97
^{13}C NMR of radical cation [4d]SbF_6 – *i.e.* [2d]SbF_6 sample dissolved in CD_2Cl_2 98
^{19}F NMR of radical cation [4d]SbF_6 – *i.e.* [2d]SbF_6 sample dissolved in CD_2Cl_2 99

^{77}Se NMR of radical cation **[4d]SbF$_6$** – *i.e.* **[2d]SbF$_6$** sample dissolved in CD_2Cl_2 100

VT-NMR EXPERIMENTS – OLIGOMERIZATION EQUILIBRIA 101

EPR SPECTROSCOPY – CHARACTERIZATION OF PARAMAGNETIC SPECIES 103

SAMPLE PREPARATION 103

Solid-state EPR of **[2a]SbF$_6$** at 298K 103

Solution-phase EPR of **[4a]SbF$_6$** in CH_2Cl_2 at 298K....................104

Solid-state vs solution-phase EPR at 298K – **[2a]SbF$_6$** vs **[4a]SbF$_6$**....................104

Solid-state EPR of **[2b]SbF$_6$** at 298K....................105

Solution-phase EPR of **[4b]SbF$_6$** in CH_2Cl_2 at 298K 105

Solid-state vs solution-phase EPR at 298K – **[2b]SbF$_6$** vs **[4b]SbF$_6$** 106

Solid-state EPR of **[2c]SbF$_6$** at 298K 107

Solution-phase EPR of **[4c]SbF$_6$** in CH_2Cl_2 at 298K....................107

Solid-state vs solution-phase EPR at 298K – **[2c]SbF$_6$** vs **[4c]SbF$_6$** 108

Solid-state EPR of **[2d]SbF$_6$** at 298K....................109

Solution-phase EPR of **[4d]SbF$_6$** in CH_2Cl_2 at 298K 109

Solid-state vs solution-phase EPR at 298K – **[2d]SbF$_6$** vs **[4d]SbF$_6$** 110

CYCLIC VOLTAMMETRY 111

SAMPLE PREPARATION 111

Oxidation of dibenzo[*b*,*d*]selenophene (**1a**) 111

Oxidation of 3,7-di-*tert*-butyldibenzo[*b*,*d*]selenophene (**1b**) 112

Oxidation of 2,8-di-*tert*-butyldibenzo[*b*,*d*]selenophene (**1c**)....................112

Oxidation of octamethyl-octahydro dinaphthoselenophene **1d**....................113

X-RAY CRYSTALLOGRAPHIC ANALYSIS 114

TRIMERIC CATIONIC SELENURANE $[2C]_3^{\cdot}(SBF_6)_3$ (CCDC 2463764) 114

COMPUTATIONAL DATA 117

Calculation of the dimerization enthalpy 117

Spin-density comparison....................117

Cartesian coordinates of the most stable ($\Delta G_{298,DCM}$) conformations as computed at the ωB97X-D/def2-TZVP,SMD(DCM)//ωB97X-D/def2-SVP,SMD(DCM) level of theory. 118

Closed-shell vs diradical nature of the Se dicationic dimer dimer Se-3a....................129

STRUCTURAL COMPARISONS AND BONDING.................... 130

Organoselenium oxidation states 130

Chalcogen radical cations containing multiple heteroatoms 130

Stable Ch(III) radical cations 131
Se – Se bond length comparison (graphical representation) 131
Bonding model 132

SPECTROSCOPIC DATA 134

^{1}H NMR OF DIBENZO[*B*,*D*]SELENOPHENE (1A) 134
^{13}C NMR OF DIBENZO[*B*,*D*]SELENOPHENE (1A) 135
^{77}SE NMR OF DIBENZO[*B*,*D*]SELENOPHENE (1A) 136
^{1}H NMR OF 3,7-DI-*TERT*-BUTYLDIBENZO[*B*,*D*]SELENOPHENE (1B) 137
^{13}C NMR OF 3,7-DI-*TERT*-BUTYLDIBENZO[*B*,*D*]SELENOPHENE (1B) 138
^{77}SE NMR OF 3,7-DI-*TERT*-BUTYLDIBENZO[*B*,*D*]SELENOPHENE (1B) 139
^{1}H NMR OF 2,8-DI-*TERT*-BUTYLDIBENZO[*B*,*D*]SELENOPHENE (1C) 140
^{13}C NMR OF 2,8-DI-*TERT*-BUTYLDIBENZO[*B*,*D*]SELENOPHENE (1C) 141
^{77}SE NMR OF 2,8-DI-*TERT*-BUTYLDIBENZO[*B*,*D*]SELENOPHENE (1C) 142
^{1}H NMR OF OCTAMETHYL-OCTAHYDRO DINAPHTHOSELENOPHENE (1D) 143
^{13}C NMR OF OCTAMETHYL-OCTAHYDRO DINAPHTHOSELENOPHENE (1D) 144
^{77}SE NMR OF OCTAMETHYL-OCTAHYDRO DINAPHTHOSELENOPHENE (1D) 145
^{1}H NMR OF 4-(2,6-DIISOPROPYLPHENYL)DIBENZO[*B*,*D*]SELENOPHENE (1E) 146
^{13}C NMR OF 4-(2,6-DIISOPROPYLPHENYL)DIBENZO[*B*,*D*]SELENOPHENE (1E) 147
^{77}SE NMR OF 4-(2,6-DIISOPROPYLPHENYL)DIBENZO[*B*,*D*]SELENOPHENE (1E) 148
^{13}C OF 3,7-BIS(TRIFLUOROMETHYL)DIBENZO[*B*,*D*]SELENOPHENE (1F) 150
^{77}SE OF 3,7-BIS(TRIFLUOROMETHYL)DIBENZO[*B*,*D*]SELENOPHENE (1F) 152
^{1}H NMR OF [2,5'-BIDIBENZOSELENOPHEN]-5'-IUM TRIFLUOROMETHANESULFONATE (SE–[5A]OTF) 153
^{1}H NMR OF [2,5'-BIDIBENZOSELENOPHEN]-5'-IUM TRIFLUOROMETHANESULFONATE (SE–[5A]OTF) 154
^{13}C NMR OF [2,5'-BIDIBENZOSELENOPHEN]-5'-IUM TRIFLUOROMETHANESULFONATE (SE–[5A]OTF) 155
^{19}F NMR OF [2,5'-BIDIBENZOSELENOPHEN]-5'-IUM TRIFLUOROMETHANESULFONATE (SE–[5A]OTF) 156
^{77}SE NMR OF [2,5'-BIDIBENZOSELENOPHEN]-5'-IUM TRIFLUOROMETHANESULFONATE SE–[5A]OTF 157
^{1}H NMR OF (*E*)-1,2-DIPHENYLDIAZENE (7) 158
^{13}C NMR OF (*E*)-1,2-DIPHENYLDIAZENE (7) 159
^{1}H NMR OF FLUORENONE (9) 160
^{13}C NMR OF FLUORENONE (9) 161
^{1}H NMR OF 5-(TRIFLUOROMETHYL) -DIBENZOSELENOPHENIUM HEXAFLUOROANTIMONATE (10) 162
^{13}C NMR OF 5-(TRIFLUOROMETHYL) -DIBENZOSELENOPHENIUM HEXAFLUOROANTIMONATE (10) 163
^{19}F NMR OF 5-(TRIFLUOROMETHYL) -DIBENZOSELENOPHENIUM HEXAFLUOROANTIMONATE (10) 164

^{77}Se NMR of 5-(trifluoromethyl) -dibenzoselenophenium hexafluoroantimonate (10) 165

^{1}H NMR of 5-(phenyl) -dibenzoselenophenium hexafluoroantimonate (11) 166

^{13}C NMR of 5-(phenyl) -dibenzoselenophenium hexafluoroantimonate (11) 167

^{19}F NMR of 5-(phenyl) -dibenzoselenophenium hexafluoroantimonate (11) 168

^{77}Se NMR of 5-(phenyl) -dibenzoselenophenium hexafluoroantimonate (11) 169

^{1}H NMR of 5-(phenyl) -dibenzoselenophenium hexafluoroantimonate (11b) 170

^{13}C NMR of 5-(phenyl) -dibenzoselenophenium hexafluoroantimonate (11b) 171

^{19}F NMR of 5-(phenyl) -dibenzoselenophenium hexafluoroantimonate (11b) 172

^{77}Se NMR of 5-(phenyl) -dibenzoselenophenium hexafluoroantimonate (11b) 173

^{1}H NMR of 5-(phenyl) -dibenzoselenophenium hexafluoroantimonate (11c) 174

^{13}C NMR of 5-(phenyl) -dibenzoselenophenium hexafluoroantimonate (11c) 175

^{19}F NMR of 5-(phenyl) -dibenzoselenophenium hexafluoroantimonate (11c) 176

^{77}Se NMR of 5-(phenyl) -dibenzoselenophenium hexafluoroantimonate (11c) 177

^{1}H NMR of 5-(phenyl) -dibenzoselenophenium hexafluoroantimonate (11d) 178

^{13}C NMR of 5-(phenyl) -dibenzoselenophenium hexafluoroantimonate (11d) 179

^{19}F NMR of 5-(phenyl) -dibenzoselenophenium hexafluoroantimonate (11d) 180

^{77}Se NMR of 5-(phenyl) -dibenzoselenophenium hexafluoroantimonate (11d) 181

^{1}H NMR of BF_3K fenofibrate derivative (12) 182

^{13}C NMR of BF_3K fenofibrate derivative (12) 183

^{19}F NMR of BF_3K fenofibrate derivative (12) 184

^{11}B NMR of BF_3K fenofibrate derivative (12) 185

^{1}H NMR of selenonium fenofibrate derivative (13) 186

^{13}C NMR of selenonium fenofibrate derivative (13) 187

^{19}F NMR of selenonium fenofibrate derivative (13) 188

^{77}Se NMR of selenonium fenofibrate derivative (13) 189

^{1}H NMR of BF_3K tianeptine intermediate (14) 190

^{13}C NMR of 5-(phenyl) -dibenzoselenophenium hexafluoroantimonate (14) 191

^{19}F NMR of BF_3K tianeptine intermediate (14) 192

^{11}B NMR of BF_3K tianeptine intermediate (14) 193

^{1}H NMR of selenonium tianeptine derivative (15) 194

^{13}C NMR of selenonium tianeptine derivative (15) 195

^{19}F NMR of selenonium tianeptine derivative (15) 196

^{77}Se NMR of selenonium tianeptine derivative (15) 197

^{1}H NMR OF 3,3'-DI-*TERT*-BUTYL-1,1'-BIPHENYL (S1) 198
^{13}C NMR OF 3,3'-DI-*TERT*-BUTYL-1,1'-BIPHENYL (S1) 199
^{1}H NMR OF OCTAMETHYL-OCTAHYDRO BINAPHTHALENE (S2) 200
^{13}C NMR OF OCTAMETHYL-OCTAHYDRO BINAPHTHALENE (S2) 201
^{13}C NMR OF 4,4'-BIS(TRIFLUOROMETHYL)-[1,1'-BIPHENYL]-2-AMINE (S3) 203
^{19}F NMR OF 4,4'-BIS(TRIFLUOROMETHYL)-[1,1'-BIPHENYL]-2-AMINE (S3) 204
^{1}H NMR OF 2-IODO-4,4'-BIS(TRIFLUOROMETHYL)-1,1'-BIPHENYL (S4) 205
^{13}C NMR OF 2-IODO-4,4'-BIS(TRIFLUOROMETHYL)-1,1'-BIPHENYL (S4) 206
^{19}F NMR OF 2-IODO-4,4'-BIS(TRIFLUOROMETHYL)-1,1'-BIPHENYL (S4) 207
^{1}H NMR OF 3,7-BIS(TRIFLUOROMETHYL)DIBENZO[*B*,*D*]IODOL-5-IUM TRIFLUOROMETHANESULFONATE (S5) ... 208
^{13}C NMR OF 3,7-BIS(TRIFLUOROMETHYL)DIBENZO[*B*,*D*]IODOL-5-IUM TRIFLUOROMETHANESULFONATE (S5) .. 209
^{19}F NMR OF 3,7-BIS(TRIFLUOROMETHYL)DIBENZO[*B*,*D*]IODOL-5-IUM TRIFLUOROMETHANESULFONATE (S5) ... 210
^{1}H NMR OF DIPHENYLSELENIDE (S6) 211
^{13}C NMR OF DIPHENYLSELENIDE (S6) 212
^{77}SE NMR OF DIPHENYLSELENIDE (S6) 213
^{1}H NMR OF DIBENZYLSELENIDE (S7) 214
^{13}C NMR OF DIBENZYLSELENIDE (S7) 215
^{77}SE NMR OF DIBENZYLSELENIDE (S7) 216
^{13}C NMR OF 3,7-DI-*TERT*-BUTYLDIBENZO[*B*,*D*]THIOPHENE (S8) 218
^{1}H NMR OF DIBENZO[*B*,*D*]SELENOPHENE 5-OXIDE (S9) 219
^{13}C NMR OF DIBENZO[*B*,*D*]SELENOPHENE 5-OXIDE (S9) 220
^{77}SE NMR OF DIBENZO[*B*,*D*]SELENOPHENE 5-OXIDE (S9) 221
^{1}H NMR OF BPIN FENOFIBRATE DERIVATIVE (S11) 222
^{13}C NMR OF BPIN FENOFIBRATE DERIVATIVE (S11) 223
^{11}B NMR OF BPIN FENOFIBRATE DERIVATIVE (S11) 224
^{1}H NMR OF BPIN TIANEPTINE INTERMEDIATE (S12) 225
^{13}C NMR OF BPIN TIANEPTINE INTERMEDIATE (S12) 226
^{11}B NMR OF BPIN TIANEPTINE INTERMEDIATE (S12) 227
^{1}H NMR OF 5-(4-(*TERT*-BUTYL)PHENYL) -DIBENZOSELENOPHENIUM HEXAFLUOROANTIMONATE (S13) 228
^{13}C NMR OF 5-(4-(*TERT*-BUTYL)PHENYL) -DIBENZOSELENOPHENIUM HEXAFLUOROANTIMONATE (S13) 229
^{19}F NMR OF 5-(4-(*TERT*-BUTYL)PHENYL) -DIBENZOSELENOPHENIUM HEXAFLUOROANTIMONATE (S13)........ 230
^{77}SE NMR OF 5-(4-(*TERT*-BUTYL)PHENYL) -DIBENZOSELENOPHENIUM HEXAFLUOROANTIMONATE (S13)........ 231
^{1}H NMR OF 5-(4-METHOXYPHENYL)-5*H*-DIBENZO[*B*,*D*]SELENOPHEN-5-IUM HEXAFLUOROANTIMONATE (S14) 232

^{13}C NMR OF 5-(4-METHOXYPHENYL)-5*H*-DIBENZO[*B*,*D*]SELENOPHEN-5-IUM HEXAFLUOROANTIMONATE (S14) 233

^{19}F NMR OF 5-(4-METHOXYPHENYL)-5*H*-DIBENZO[*B*,*D*]SELENOPHEN-5-IUM HEXAFLUOROANTIMONATE (S14) 234

^{77}SE NMR OF 5-(4-METHOXYPHENYL)-5*H*-DIBENZO[*B*,*D*]SELENOPHEN-5-IUM HEXAFLUOROANTIMONATE (S14) 235

^{1}H NMR OF 5-(4-(TRIFLUOROMETHYL)PHENYL)-5*H*-DIBENZO[*B*,*D*]SELENOPHEN-5-IUM HEXAFLUOROANTIMONATE (S15) 236

^{13}C NMR OF 5-(4-(TRIFLUOROMETHYL)PHENYL)-5*H*-DIBENZO[*B*,*D*]SELENOPHEN-5-IUM HEXAFLUOROANTIMONATE (S15) 237

^{19}F NMR OF 5-(4-(TRIFLUOROMETHYL)PHENYL)-5*H*-DIBENZO[*B*,*D*]SELENOPHEN-5-IUM HEXAFLUOROANTIMONATE (S15) 238

^{77}SE NMR OF 5-(4-(TRIFLUOROMETHYL)PHENYL)-5*H*-DIBENZO[*B*,*D*]SELENOPHEN-5-IUM HEXAFLUOROANTIMONATE (S15) 239

^{1}H NMR OF 5-(*O*-TOLYL)-5*H*-DIBENZO[*B*,*D*]SELENOPHEN-5-IUM HEXAFLUOROANTIMONATE (S16) 240

^{13}C NMR OF 5-(*O*-TOLYL)-5*H*-DIBENZO[*B*,*D*]SELENOPHEN-5-IUM HEXAFLUOROANTIMONATE (S16) 241

^{19}F NMR OF 5-(*O*-TOLYL)-5*H*-DIBENZO[*B*,*D*]SELENOPHEN-5-IUM HEXAFLUOROANTIMONATE (S16)........ 242

^{77}SE NMR OF 5-(*O*-TOLYL)-5*H*-DIBENZO[*B*,*D*]SELENOPHEN-5-IUM HEXAFLUOROANTIMONATE (S16)........ 243

REFERENCES 244

Materials and Methods

All air- and moisture-insensitive reactions were carried out under ambient atmosphere in round-bottom flasks, vials fitted with rubber septa, or Schlenck flasks. The reaction progress was monitored by thin-layer chromatography (TLC). Concentration under reduced pressure was performed by rotary evaporation at 40 °C at an appropriate pressure. Purified compounds were further dried under high vacuum (0.010–0.005 mbar). Yields refer to purified and spectroscopically pure compounds, unless otherwise stated.

Solvents

Technical heptane and ethyl acetate were distilled using a 20L Rotary evaporator. Dry acetonitrile, dry DMSO, dry CH_2Cl_2 were purchased from Acros Organics. Reactions were performed using technical solvents obtained from Acros Organics, TCI, and Sigma-Aldrich, unless otherwise stated.

Chromatography

Thin layer chromatography (TLC) was performed on aluminum plates coated with silica gel 60 F_{254} with 0.20 mm thickness purchased from Macherey-Nagel®. Chromatograms were visualized by fluorescence quenching with UV light at 254 nm or by staining using potassium permanganate or phosphomolybdic acid stains followed by heating. Flash column chromatography was performed using silica gel 60 (230-400 mesh, Macherey-Nagel®).

Spectroscopy and instruments

NMR spectroscopy

^{1}H NMR, ^{11}B NMR, ^{13}C NMR, ^{19}F NMR, and ^{77}Se spectra were recorded using a Bruker AV-400, AV-500, AV-600 or AV-700 spectrometer at 300 K. Chemical shifts (δ) are given in parts per million (ppm), referenced to the solvent peak: for $CDCl_3$, defined at δ = 7.26 ppm (^{1}H NMR) and δ = 77.16 ppm (^{13}C NMR); for DMSO-d_6, defined at δ = 2.50 ppm (^{1}H NMR) and δ = 39.52 ppm (^{13}C NMR). Coupling constants (J) are reported in Hertz (Hz). ^{1}H NMR splitting patterns are designated as singlet (s), doublet (d), triplet (t), quartet (q), quintet (quint.) or a combination thereof, as they appeared in the spectrum. Splitting patterns that could not be interpreted or easily visualized are designated as multiplet (m) or broad (br). NMR shifts of heteronuclei were referenced using their respective Ξ value using the *xiref* au program in Bruker Topspin following IUPAC recommendations.[48]

Solid state NMR spectra were obtained on a Bruker Avance NEO 500 MHz wide bore system (Bruker BioSpin GmbH & Co. KG, Ettlingen, Rheinstetten, Germany) using a 4 mm BB/^{19}F-^{1}H dual resonance magic angle spinning (MAS) probe with automatic tuning and matching. The resonance frequency for ^{77}Se was set to 95.4 MHz, the MAS rotor spinning to 15 kHz. Except for **[2c]SbF$_6$**, which was recorded by direct excitation, cross polarization (CP) was used applying a ramped contact pulse with 8 ms contact time. The relaxation delay was set individually for every sample in a range between 10 s up to 300 s. During acquisition ^{1}H was high power decoupled using a swept-frequency two-pulse phase modulation

scheme. To identify the isotropic chemical shift, some of the experiments were repeated using a rotor spinning speed of 8 or 11 kHz.

Mass spectrometry

Mass spectra were obtained using a Bruker maXis UHR-TOF spectrometer (70 eV), using electrospray ionization (ESI) or atmospheric-pressure chemical ionization (APCI) or an Agilent 7200B GC/Q-TOF spectrometer (70 eV), using electron ionization (EI). MALDI-TOF Analysis: high-resolution MALDI-TOF MS analyses were performed using a Bruker timsTOF fleX ESI/MALDI dual source – trapped ion mobility separation – Qq-TOF massspectrometer in positive ion mode. The sum formulas of the detected ions were determined using Bruker Compass DataAnalysis 5.3 based on the mass accuracy ($\Delta m/z \leq 5$ ppm) and isotopic pattern matching (SmartFormula algorithm). One microliter of a solution of the matrix (trans-2-[3-(4-t-butylphenyl)-2-methyl-2-propenylidene]malonitrile, DCTB) in CH_2Cl_2 was spotted onto a well of the MALDI plate, and the solvent was allowed to evaporate. The solvent was evaporated prior to analysis. Data were collected in positive polarity mode.

EPR spectroscopy

Electron Paramagnetic Resonance was performed in an EMX Micro X-band EPR spectrometer (Bruker Biospin GmbH, Rheinstetten, Germany) at room temperature (*ca.* 298 K). Data acquisition: perpendicular mode, microwave frequency of 9.84 GHz, the modulation frequency to 100 kHz, the center field to 3484 G, the sweep width to 500 G, the sweep time to 30 s, the modulation amplitude of 1 G, a 1.28 ms time constant and 6.00 ms conversion time with a microwave power of 20.46 mW. The spectra were collected using the Bruker Xepr software.

Simulations were carried out using Easyspin[49] and MATLAB was used for further analysis.

SXRD

Single crystal X-ray diffraction data were collected with a Stadivari Diffractometer (STOE & Cie GmbH, Germany) equipped with an EIGER2 R500 detector (Dectris Ltd, Switzerland). Data were processed and scaled with the STOE software suite X-Area (STOE & Cie GmbH). Structures were solved with SHELXT[50] and refined with SHELXL[51] or Olex2.[52] Model building was done with Olex2 or ShelXle.[53] Structures were validated with CHECKCIF (https://checkcif.iucr.org/).

See the respective CIF files for exact versions and more details.

Cyclic Voltammetry

Cyclic voltammetry was measured using the Ossila potentiostat, an Ag/AgCl (3M NaCl) reference electrode, a glassy carbon disc working electrode, and a platinum disk counter electrode (purchased from "ALS Electrochemistry & Spectroelectrochemistry"). All measurements were carried out in CH_2Cl_2 with 0.10 M NBu_4PF_6 as electrolyte if not stated otherwise. The electrolyte was purchased from BLDPharm®, recrystallized twice with ethanol, dried in vaccuo for 24h ($5 \cdot 10^{-3}$ bar), and stored in a desiccator.

Computational details

The conformational space of all molecules has been initially searched using meta-dynamics simulations based on generic force field GFN-FF[54] calculations as implemented in Conformer-Rotamer Ensemble Sampling Tool CREST.[55,56]

The structures located with the CREST have then been subjected to ωB97X-D/def2-SVP[57] geometry optimization.

The nature of all stationary points (minima and transition states) was verified through the computation of the vibrational frequencies. The thermal corrections to the Gibbs free energy were combined with the single point energies calculated at the ωB97X-D/def2-TZVP[58] to yield ωB97X-D/def2-TZVP//ωB97X-D/def2-SVP Gibbs free energies ("G_{298}") and enthalpies ("H_{298}") at 298.15 K. The enthalpies ("H_{298}") were utilized to obtain the dissociation enthalpies. The complexes of the cationic species with the BF_4^- anion were computed to consider the counterion effect. All energies are reported in kcal·mol^{-1}. The polarizable continuum model (PCM) with SMD parameters[59,60] was applied to consider solvent (DCM) effects for single point energy calculations. Free energies in solution have been corrected to a reference state of 1 mol l^{-1} at 298.15 K through the addition of RTln(24.46) = +7.925 kJ mol^{-1} to the gas phase (1 atm) free energies.

Multiconfigurational calculations using the complete active space self-consistent field (CASSCF) method with ANO-R type basis sets (R2 for Se, R1 for C and R0 for H)[61] were employed for the head-to-head **Se-3a** dimer in both the singlet and triplet states. Two active spaces were explored: (2,2), which includes the σ and σ* orbitals of the Se-Se bond, and the extended (6,6), which incorporates the σ and σ* orbitals of the Se-Se bond along with two occupied π and two unoccupied π* orbitals.

The DFT geometry optimization and single point energy calculations have been performed with the Gaussian 16 program package.[62] The ORCA 5.0.1 software was applied for the spin density distribution calculations (ωB97X-D3/def2-TZVP,CPCM(DCM)//ωB97X-D/def2-SVP,SMD(DCM).[63] The CASSCF calculations have been performed with the OpenMOLCAS software.[64]

Starting materials

All substrates were used as received from commercial suppliers (Sigma-Aldrich, TCI, Fluorochem, or BLDPharm), unless otherwise stated.

SAFETY STATEMENT

The procedures reported in this work are intended for use only by individuals with proper training in experimental chemistry. All hazardous materials (solid, liquid, or gaseous) should be handled using the standard work procedures described in references such as Chapter 4 of "Prudent Practices in the Laboratory".[65] All chemical waste should be disposed of in accordance with local regulations. For general guidelines for the management of chemical waste, see Chapter 8 of "Prudent Practices in the Laboratory".[65] Reaction set-up, and chemical-specific hazards are highlighted in bold with "Caution:" notes in the procedures reported in these supplementary materials. It is important to note that the absence of a caution note does not imply that no significant hazards are associated with the chemicals involved in that procedure.

During the course of this study no explosions or violent decompositions occurred. A summary of the possible risks and hazards is described below:

Caution: When performing reactions in pressurized systems (such as closed vials, pressure tubes, and autoclaves), a blast shield must be used to minimize personal damage in case of an accident.

Caution: Reactions that generate corrosive gases (such as NO and NO_2) must be performed in well-ventilated fume hoods to minimize personal damage in case of inhalation.

AUTHOR CONTRIBUTIONS

Conceptualization: Kirill Zhiliaev and Javier Mateos.

Investigation: Kirill Zhiliaev.

Methodology: Kirill Zhiliaev developed the synthesis as well as the applications of cationic selenuranes.

DFT calculations: Boris Maryasin performed all the calculations reported in this work.

Solid-state NMR studies: Hanspeter Kählig performed the ^{77}Se MAS-NMR experiments.

EPR studies: Marcos Gil-Sepulcre measured all the EPR spectra reported in this work.

Writing: Kirill Zhiliaev and Javier Mateos wrote the main text and the supporting information considering the inputs from all the co-authors.

Direction: Javier Mateos directed the project and secured the funding.

All authors have given approval to the final version of the manuscript.

EXPERIMENTAL DATA – SYNTHETIC PROCEDURES

GENERAL PROCEDURE FOR THE SYNTHESIS OF CATIONIC SELENURANES

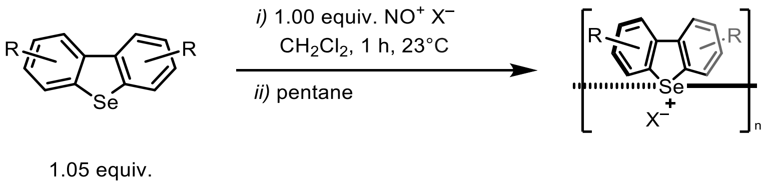

Caution: When performing reactions in pressurized systems (such as closed vials, pressure tubes, and autoclaves), a blast shield must be used to minimize personal damage in case of an accident. See safety statement on page S10. Reactions that generate corrosive gases (such as NO and NO_2) must be performed in well-ventilated fume hoods to minimize personal damage in case of inhalation.

1.00 mmol scale: To an oven-dried 50-mL round-bottomed Schlenk tube equipped with a magnetic stirring bar, selenide (1.05 mmol, 1.05 equiv.) was added. The tube was sealed with a septum, connected via a Tygon® tubing to a Schlenk line, and evacuated and purged with argon three times. CH_2Cl_2 (10 mL, 0.10 M) was added using a 10.0-mL syringe. Then, with a flow of argon the septum was removed and the nitrosonium salt (1.00 mmol, 1.00 equiv.) was added portionwise to the stirring (600 rpm) mixture, and the tube was sealed with a septum. The reaction mixture was stirred at room temperature for 1 h. Simultaneously, a receiving 100-mL Schlenk flask was sealed with a septum, connected via a Tygon® tubing to a Schlenk line, and evacuated and purged with argon three times. After 1 h of stirring, the flasks were connected via canula, the receiving flask was cooled down using a low form dewar flask cooled with a liquid nitrogen, and the solvent was evaporated to the receiving flask. The canula was removed. Then, pentane (10 mL) was added to the reaction tube, and the mixture was stirred for additional 5 min. With a flow of argon the septum of the reaction tube was removed, equipped with a canula filter (Figure S1), and connected to a 100-mL round bottom flask (second receiving flask). With a positive argon pressure, the reaction mixture was filtered, and washed with pentane (3×10 mL). The residue was dried under high vacuo for 2 h to afford the cationic selenurane product.

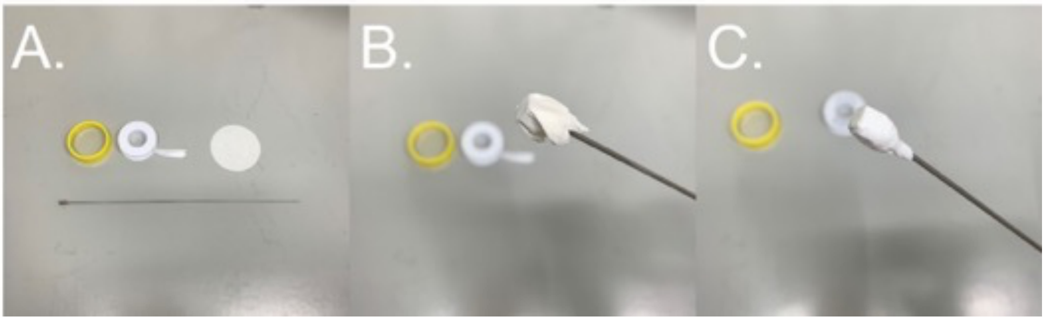

Figure S1. Canula filter. **A.** A long metal needle, paper filter, and Teflon tape. **B.** The paper filter is added on the hub of the needle **C.** The paper filter is fixed on the hub of the needle with a wrapped Teflon tape.

40.0 mmol scale: To an oven-dried 2-L round-bottomed two-necked flask equipped with a vacuum adaptor and a magnetic stirring bar dibenzo[*b*,*d*]selenophene (9.43 g, 40.8 mmol, 1.02 equiv.) was added (Figure S2). The flask was sealed with a septum, connected via a Tygon® tubing to a Schlenk line, and evacuated and purged with argon three times. CH_2Cl_2 (400 mL, 0.10 M) was added, and the reaction mixture was cooled to 0 °C with an ice bath. Then, with a flow of argon the septum was replaced for a funnel, and nitrosonium hexafluoroantimonate (10.6 g, 40.0 mmol, 1.00 equiv.) was added portionwise to the stirring (600 rpm) mixture, and the flask was sealed with a septum equipped with a deflated balloon. The ice bath was removed and the reaction mixture was stirred at room temperature for 2 h. **Caution:** NO gas is formed during the course of the reaction. After 2 h, pentane (400 mL) was added and the resulting mixture was stirred for additional 10 min. After 10 min, the stirring was turned off for to promote solid sedimentation. The septum equipped with the balloon was removed and the mixture was filtered on a fritted funnel under a flow of argon. The solid was washed with pentane (2×200 mL). Then, the residue was dried under high vacuo for 2 h to afford cationic selenurane **[2a]SbF$_6$** in 93% yield (17.3 g, 37.0 mmol).

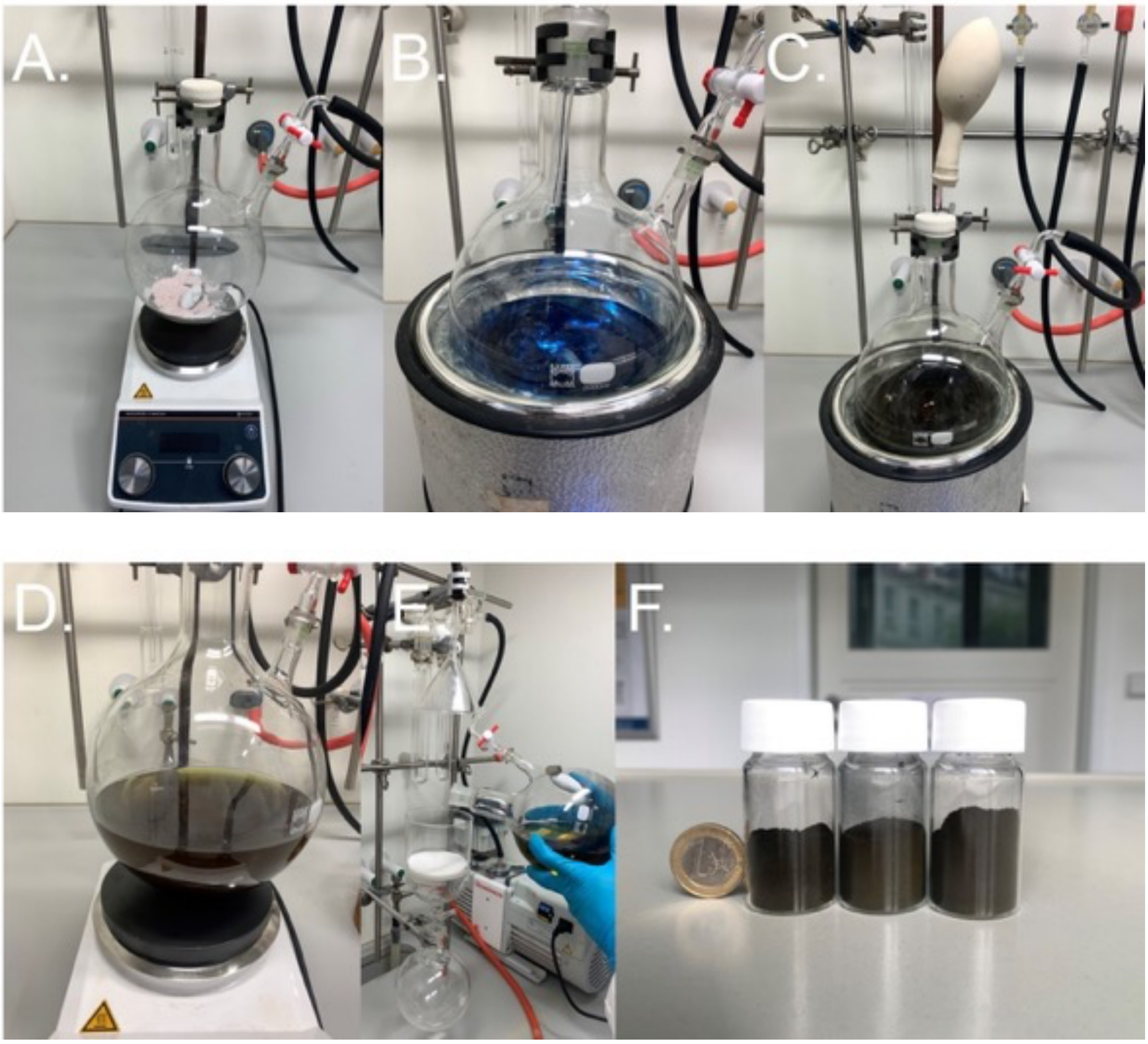

Figure S2. 40.0 mmol scale synthesis of cationic selenurane **[2a]SbF$_6$**. **A.** Reaction setup with dibenzo[*b*,*d*]selenophene and the stirring bar. **B.** Addition of nitrosonium hexafluoroantimonate.

C. The reaction flask is equipped with a deflated balloon. **D.** Addition of pentane after 2 h of reaction. **E.** Filtration of the mixture under a flow of argon. **F.** The obtained product in three different 20-mL vials (The 1 € coin was used for size comparison).

Storage recommendation note

We recommend storing the reported cationic selenurane salts under inert atmosphere (e.g., in a glovebox) for long-term stability. However, the salts remain stable under ambient conditions for several weeks. As they are hygroscopic, storage in a desiccator is advised to minimize moisture uptake.

General procedure for the utilization of cationic selenuranes as oxidants

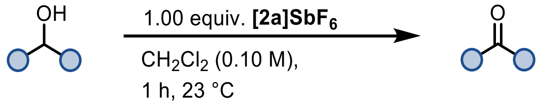

0.50 mmol scale: Under an ambient atmosphere, to a 20-mL borosilicate vial equipped with a magnetic stir bar selenurane (0.50 mmol, 1.00 equiv.) and CH_2Cl_2 (5 mL, 0.10 M) were added. Then, the reductant (0.50 mmol, 1.00 equiv.) was added portionwise to the stirring (200 rpm) mixture. The reaction mixture was stirred at 23 °C for 1 h. After 1 h, the mixture was concentrated by rotary evaporation under reduced pressure. The residue was purified by flash column chromatography on silica gel to afford a pure product.

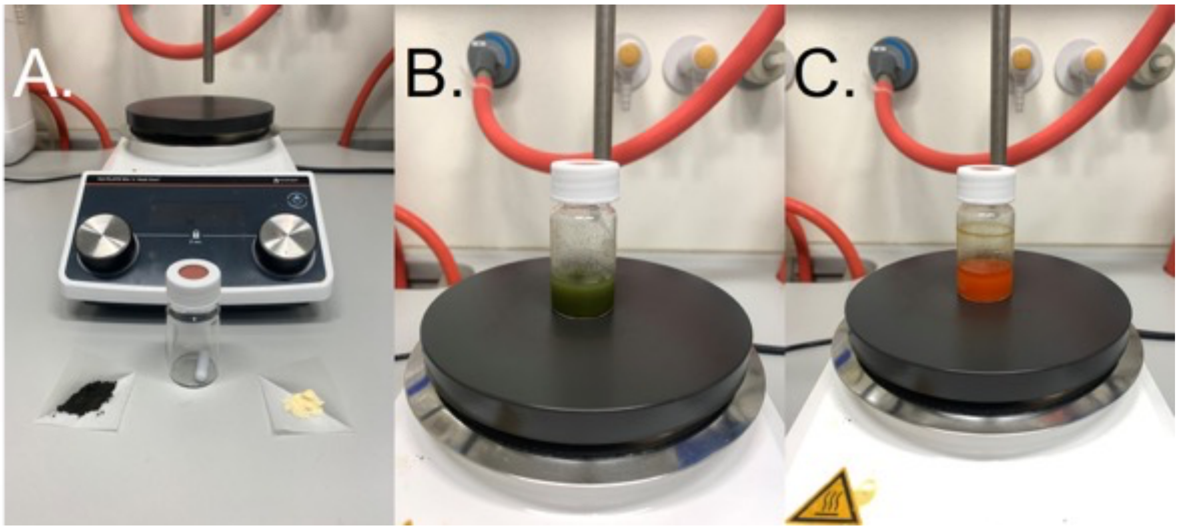

Figure S3. Oxidation of phenylhydrazine with selenurane **[2a]SbF₆**. **A.** The solids are weighted in the bench and added to a 20-mL borosilicate vial equipped with a magnetic stir bar. **B.** Initial reaction mixture. **C.** Reaction mixture after 1 h of stirring.

GENERAL PROCEDURE FOR THE UTILIZATION OF CATIONIC SELENURANES AS GROUP-TRANSFER REAGENTS

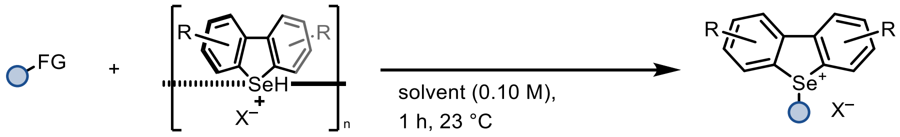

0.50 mmol scale: Under an ambient atmosphere, to a 20-mL borosilicate vial equipped with a magnetic stir bar, selenurane (1.00 mmol, 2.00 equiv.) and CH_2Cl_2 (5 mL, 0.10 M) were added. Then, the substrate was added portionwise to the stirring (200 rpm) mixture. The reaction mixture was stirred at 23 °C for 1 h. After 1 h, the mixture was concentrated by rotary evaporation under reduced pressure. The residue was purified by washing with Et_2O (3×10 mL). After flash column chromatography on silica gel, pure selenonium salts were obtained.

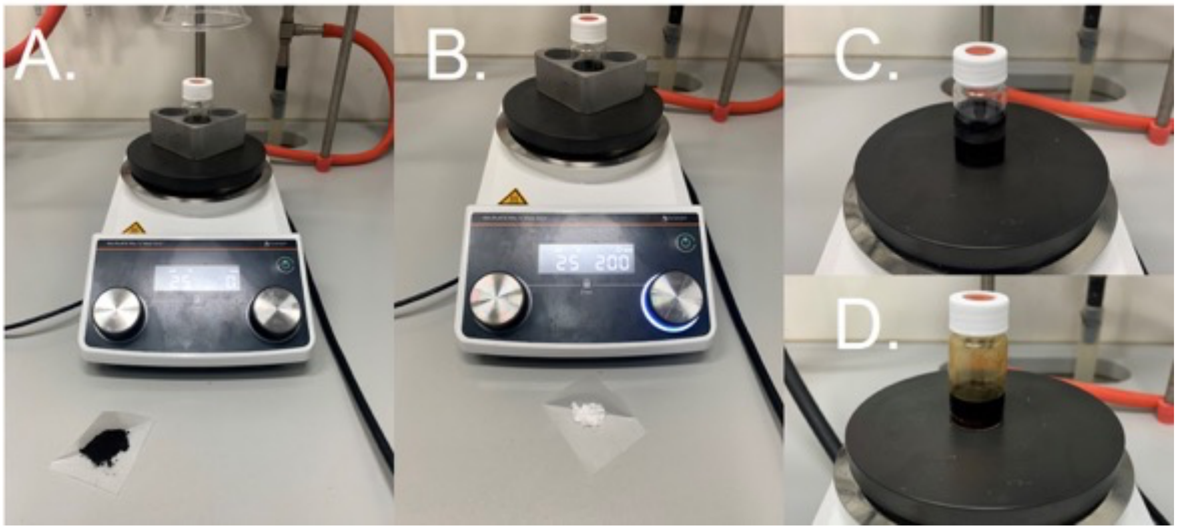

Figure S4. Reaction of potassium trifluoro(phenyl)borate with selenurane **[2a]SbF$_6$**. **A.** Selenurane **[2a]SbF$_6$** was weighted in the bench and added to a 20-mL borosilicate vial equipped with a magnetic stir bar. **B.** Potassium trifluoro(phenyl)borate was added to the reaction mixture. **C.** initial reaction mixture. **D.** Reaction mixture after 1 h of stirring.

Preparation of selenides 1a-e, and S1–S8

Dibenzo[*b*,*d*]selenophene (1a)

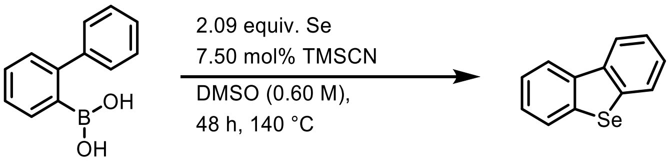

67% yield **1a**

Caution: When performing reactions in pressurized systems (such as closed vials, pressure tubes, and autoclaves), a blast shield must be used to minimize personal damage in case of an accident.

A 350-mL Ace pressure tube (150 psi maximum pressure) equipped with a magnetic stir bar was flushed with argon for 5 min and charged with 2-biphenylboronic acid (11.9 g, 60.0 mmol, 1.00 equiv.), selenium powder (10.0 g, 125 mmol, 2.09 equiv.) and dry DMSO (100 mL, 0.60 M). Then, trimethylsilyl cyanide (560 µL, 4.50 mmol, 7.50 mol%) was added using a 1.00-mL syringe, and the pressure tube was sealed with a front seal plug equipped with an O-ring, ensuring a tight and secure seal. The reaction mixture was stirred (800 rpm) for 48 h, and the pressure tube was heated to 140 °C in an oil bath on a heating plate. Then, the pressure tube was removed from the oil bath and allowed to cool to 23 °C (ca. 40 minutes). The reaction mixture was stirred (800 rpm) at 23 °C, and the seal was removed with caution while stirring to allow the gasses to escape. The reaction mixture was diluted with Et_2O (200 mL) and filtered through a pad of celite using a fritted funnel. Additional 600 mL of Et_2O were used to elute the apolar products. The combined organic layers were washed three times with water (3×200 mL). The combined organic phase was dried over Na_2SO_4, and filtered by gravity using an 8-cm diameter funnel containing a fluted filter paper. Then, the resulting mixture was concentrated by rotary evaporation under reduced pressure. The residue was purified by flash column chromatography on silica gel using pure heptane as eluent to afford the desired product as a pink crystalline solid in 67% yield (9.29 g, 40.2 mmol).

R_f = 0.25 (heptane).

NMR Spectroscopy:

^{1}H NMR (500 MHz, $CDCl_3$, 23 °C, δ): 8.14 (d, *J* = 7.8 Hz, 2H), 7.90 (d, *J* = 7.8 Hz, 2H), 7.50 – 7.43 (m, 2H), 7.43 – 7.38 (m, 2H).

^{13}C NMR (101 MHz, $CDCl_3$, 23 °C, δ): 139.4, 138.4, 127.0, 126.2, 125.0, 123.0.

^{77}Se NMR (115 MHz, $CDCl_3$, 23 °C, δ): 450.6.

HRMS-EI(m/z) calc'd for $C_{12}H_8Se$ $[M]^+$, 231.9791; found, 231.9782; deviation: –3.9 ppm.

3,7-Di-*tert*-butyldibenzo[*b*,*d*]selenophene (1b)

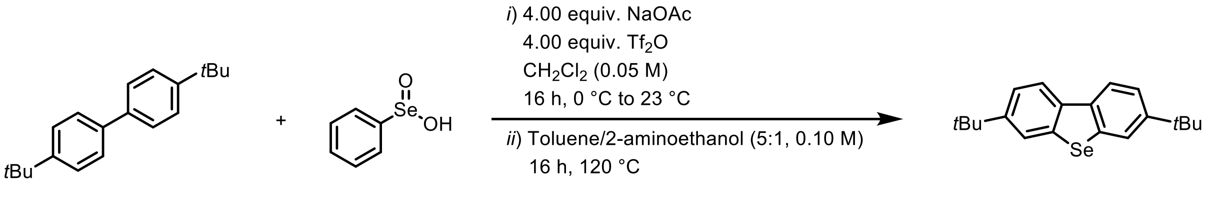

2.00 equiv. **57% yield** **1b**

i) To an oven-dried 250-mL round-bottomed Schlenk flask equipped with a magnetic stirring bar, 4,4'-di-*tert*-butyl-1,1'-biphenyl (1.33 g, 5.00 mmol, 1.00 equiv.), benzeneseleninic acid (1.91 g, 10.0 mmol, 2.00 equiv.) and sodium acetate (1.64 g, 20.0 mmol, 4.00 equiv.) were added. The flask was sealed with a septum, connected via a Tygon® tubing to a Schlenk line, and evacuated and purged with argon three times. CH_2Cl_2 (100 mL, 0.05 M) was added and the mixture was cooled to 0 °C using an ice bath. Trifluoromethanesulfonic anhydride (3.38 mL, 20.0 mmol, 4.00 equiv.) was added to the stirring (600 rpm) mixture dropwise using a 5.00-mL syringe. After the addition of trifluoromethansulfonic anhydride was completed, the ice bath was removed, and the reaction mixture was stirred for additional 16 h at 23 °C. The resulting mixture was quenched with a saturated aq. $NaHCO_3$ solution (50 mL), extracted with EtOAc (3×50 mL), and washed with brine. The combined organic phase was dried over Na_2SO_4, and filtered by gravity using an 8-cm diameter funnel containing a fluted filter paper to a 500-mL round-bottomed flask. The solvent mixture was evaporated under reduced pressure and the residue was dried under high vacuo.

ii) Next, the 500-mL round-bottomed flask was equipped with a stirring bar, toluene (50 mL, 0.10 M), and 2-aminoethanol (10.3 mL, 170 mmol, 34.0 equiv.) were added. The flask was equipped with a reflux condenser and the reaction mixture was stirred at 120 °C for 16 h using an oil bath. After 16h, the flask was removed from the oil bath and allowed to cool to 23 °C (ca. 30 minutes). Then, the mixture was quenched with water (50 mL) and brine (50 mL). The resulting mixture was transferred to a 250-mL separation funnel and organic phase was separated. The aqueous phase was extracted with EtOAc (3×50 mL). The combined organic phase was dried over Na_2SO_4, and filtered by gravity using an 8-cm diameter funnel containing a fluted filter paper. Then, the resulting mixture was concentrated by rotary evaporation under reduced pressure. The residue was purified by flash column chromatography on silica gel using pure heptane as eluent to afford the desired product as colorless crystalline solid in 57% yield (984 mg, 2.87 mmol).

$\mathbf{R}_f$ = 0.49 (heptane).

NMR Spectroscopy:

^{1}H NMR (600 MHz, $CDCl_3$, 23 ºC, δ): 8.00 (d, J = 8.3 Hz, 2H), 7.88 (d, J = 1.8 Hz, 2H), 7.49 (dd, J = 8.3, 1.8 Hz, 2H), 1.42 (s, 18H).

^{13}C NMR (151 MHz, $CDCl_3$, 23 ºC, δ): 150.0, 139.4, 136.0, 122.8, 122.6, 122.2, 35.2, 31.6.

^{77}**Se NMR** (115 MHz, $CDCl_3$, 23 °C, δ): 446.6.

HRMS-ESI(m/z) calc'd for $C_{20}H_{25}Se$ $[M+H]^+$, 345.1116; found, 345.1106; deviation: –2.9 ppm.

3,3'-Di-*tert*-butyl-1,1'-biphenyl (S1)

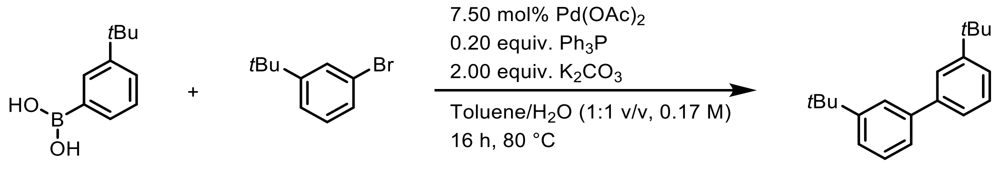

83% yield **S1**

Caution: When performing reactions in pressurized systems (such as closed vials, pressure tubes, and autoclaves), a blast shield must be used to minimize personal damage in case of an accident.

A 100-mL Ace pressure tube (150 psi maximum pressure) equipped with a magnetic stir bar was flushed with argon for 2 min, and was charged with 3-*tert*-butylphenylboronic acid (3.86 g, 21.6 mmol, 1.08 equiv.), palladium(II) acetate (337 mg, 1.50 mmol, 7.50 mol%), triphenylphosphine (1.05 g, 4.00 mmol, 0.200 equiv.), and potassium carbonate (5.53 g, 40.0 mmol, 2.00 equiv.). Then, toluene (30 mL) and water (30 mL) were added to the tube using a 60.0-mL syringes. 1-Bromo-3-(*tert*-butyl)benzene (3.42 mL, 20.0 mmol, 1.00 equiv.) was added to the resulting mixture using a 5.00-mL syringe. The pressure tube was sealed with a front seal plug equipped with an O-ring, ensuring a tight and secure seal and the reaction mixture was stirred at 80 °C for 16 h using an oil bath. After 16 h, the flask was removed from the oil bath and allowed to cool to 23 °C (ca. 30 minutes). After cooling down, the reaction was transferred to a 250-mL separation funnel and organic phase was separated. The aqueous phase was extracted with EtOAc (3×40 mL). The combined organic phase was dried over Na_2SO_4, and filtered by gravity using an 8-cm diameter funnel containing a fluted filter paper. Then, the resulting mixture was concentrated by rotary evaporation under reduced pressure. The residue was purified by flash column chromatography on silica gel using pure heptane as eluent to afford the desired product as colorless oil in 83% yield (9.29 g, 40.2 mmol).

$\mathbf{R}_f$ = 0.37 (heptane).

NMR Spectroscopy:

^{1}**H NMR** (400 MHz, $CDCl_3$, 23 °C, δ): 7.69 – 7.62 (m, 2H), 7.50 – 7.38 (m, 6H), 1.53 – 1.34 (m, 18H).

^{13}**C NMR** (125 MHz, $CDCl_3$, 23 °C, δ): 151.6, 141.9, 128.5, 124.7, 124.7, 124.3, 35.0, 31.6.

HRMS-EI(m/z) calc'd for $C_{20}H_{26}$ $[M]^+$, 266.2035; found, 266.2031; deviation: –1.6 ppm.

2,8-Di-*tert*-butyldibenzo[*b*,*d*]selenophene (1c)

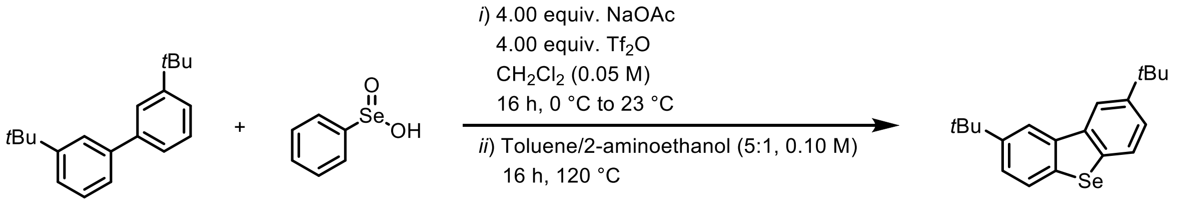

S1 2.00 equiv. **61% yield** **1c**

i) To an oven-dried 250-mL round-bottomed Schlenk flask equipped with a magnetic stirring bar, 3,3'-di-*tert*-butyl-1,1'-biphenyl **S1** (1.33 g, 5.00 mmol, 1.00 equiv.), benzeneseleninic acid (1.91 g, 10.0 mmol, 2.00 equiv.) and sodium acetate (1.64 g, 20.0 mmol, 4.00 equiv.) were added. The flask was sealed with a septum, connected via a Tygon® tubing to a Schlenk line, and evacuated and purged with argon three times. CH_2Cl_2 (100 mL, 0.05 M) was added and the mixture was cooled to 0 °C using an ice bath. Trifluoromethansulfonic anhydride (3.38 mL, 20.0 mmol, 4.00 equiv.) was added to the stirring (600 rpm) mixture dropwise using a 5.00-mL syringe. After the addition of trifluoromethanesulfonic anhydride was completed, the ice bath was removed, and the reaction mixture was stirred for additional 16 h at 23 °C. The resulting mixture was quenched with a saturated aq. $NaHCO_3$ solution (50 mL), extracted with EtOAc (3×50 mL), and washed with brine. The combined organic phase was dried over Na_2SO_4, and filtered by gravity using an 8-cm diameter funnel containing a fluted filter paper to a 500-mL round-bottomed flask. The solvent mixture was evaporated under reduced pressure and the residue was dried under high vacuo.

ii) Next, the 500-mL round-bottomed flask was equipped with a stirring bar, toluene (50 mL, 0.10 M), and 2-aminoethanol (10.3 mL, 170 mmol, 34.0 equiv) were added. The flask was equipped with a reflux condenser and the reaction mixture was stirred at 120 °C for 16 h using an oil bath. After 16 h, the flask was removed from the oil bath and allowed to cool to 23 °C (ca. 30 minutes). Then, the mixture was quenched with water (50 mL) and brine (50 mL). The resulting mixture was transferred to a 250-mL separation funnel and organic phase was separated. The aqueous phase was extracted with EtOAc (3×50 mL). The combined organic phase was dried over Na_2SO_4, and filtered by gravity using an 8-cm diameter funnel containing a fluted filter paper. Then, the resulting mixture was concentrated by rotary evaporation under reduced pressure. The residue was purified by flash column chromatography on silica gel using pure heptane as eluent to afford the desired product as colorless crystalline solid in 61% yield (1.05 g, 3.05 mmol).

R_f = 0.38 (heptane).

NMR Spectroscopy:

^{1}H NMR (400 MHz, $CDCl_3$, 23 ºC, δ): 8.03 (d, *J* = 2.1 Hz, 2H), 7.67 (d, *J* = 8.3 Hz, 2H), 7.33 (dd, *J* = 8.3, 2.1 Hz, 2H), 1.35 (s, 18H).

^{13}C NMR (101 MHz, $CDCl_3$, 23 ºC, δ): δ 148.1, 138.4, 136.6, 125.8, 124.9, 119.2, 35.0, 31.8.

^{77}**Se NMR** (101 MHz, $CDCl_3$, 23 °C, δ): 434.1.

HRMS-EI(m/z) calc'd for $C_{20}H_{24}Se$ $[M]^+$, 344.1043; found, 344.1042; deviation: –0.3 ppm.

Octamethyl-octahydro binaphthalene S2

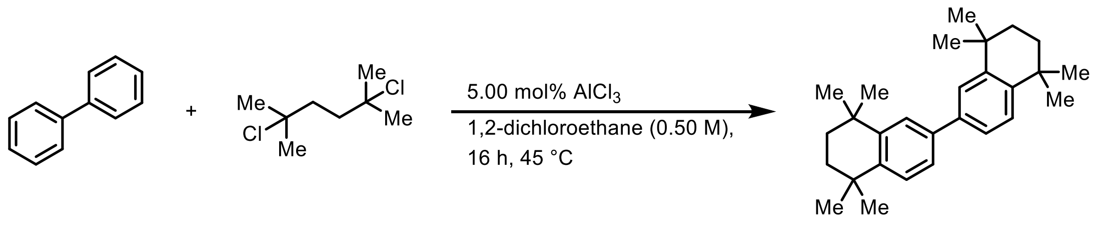

2.00 equiv. **79% yield** **S2**

Caution: When performing reactions in pressurized systems (such as closed vials, pressure tubes, and autoclaves), a blast shield must be used to minimize personal damage in case of an accident.

To an oven-dried 100-mL round-bottomed Schlenk flask equipped with a magnetic stirring bar, biphenyl (3.10 g, 20.0 mmol, 1.00 equiv.) was added. The flask was sealed with a septum, connected via a Tygon® tubing to a Schlenk line, and evacuated and purged with argon three times. 1,2-Dichloroethane (20 mL, 1.00 M) was added and after complete dissolution of the biphenyl, the septum was removed under the flow of argon, aluminum chloride (136 mg, 1.00 mmol, 5.00 mol%) was added to the stirring (500 rpm) mixture, and the flask was sealed with the septum again. Then, a 20-mL vial equipped with a stirring bar was charged with a 2,5-dichloro-2,5-dimethylhexane (7.41 g, 40.0 mmol, 2.00 equiv.) and 1,2-dichloroethane (20 mL, 2.00 M) was added, and the resulting mixture was stirred until the complete dissolution of the solids. This solution of 2,5-dichloro-2,5-dimethylhexane in 1,2-dichloroethane was added dropwise to a stirring (500 rpm) mixture in a Schlenk flask (0.50 M) over 40 min at 23 °C. Then, the reaction mixture was stirred for additional 16 h heating at 45 °C using an oil bath. After 16 h, the flask was removed from the oil bath and allowed to cool to 23 °C (ca. 10 minutes). In a 100-mL beaker equipped with a stirring bar a mixture of 22.0 g of ice and 2.20 mL of concentrated hydrochloric acid was prepared. The reaction mixture was poured to a beaker with the prepared ice mixture and stirred (400 rpm) for 15 min. The resulting mixture was transferred to a 250-mL separation funnel and organic phase was separated. The aqueous phase was extracted with CH_2Cl_2 (3×30 mL). The combined organic phase was dried over Na_2SO_4, and filtered by gravity using an 8-cm diameter funnel containing a fluted filter paper. Then, the resulting mixture was concentrated by rotary evaporation under reduced pressure. The residue was purified by recrystallization with CH_2Cl_2/methanol (1:1) mixture to afford pure product as a colorless solid in 79% yield (5.95 g, 15.9 mmol).

NMR Spectroscopy:

^{1}**H NMR** (400 MHz, $CDCl_3$, 23 °C, δ): 7.53 – 7.47 (m, 2H), 7.42 – 7.32 (m, 4H), 1.78 – 1.69 (m, 8H), 1.40 – 1.31 (m, 24H).

^{13}**C NMR** (101 MHz, $CDCl_3$, 23 °C, δ): 145.1, 143.7, 139.1, 126.9, 125.5, 124.7, 35.4, 35.3, 34.5, 34.3, 32.1.

HRMS-EI(m/z) calc'd for $C_{28}H_{38}$ $[M]^+$, 374.2974; found, 374.2967; deviation: –1.9 ppm.

Octamethyl-octahydro dinaphthoselenophene 1d

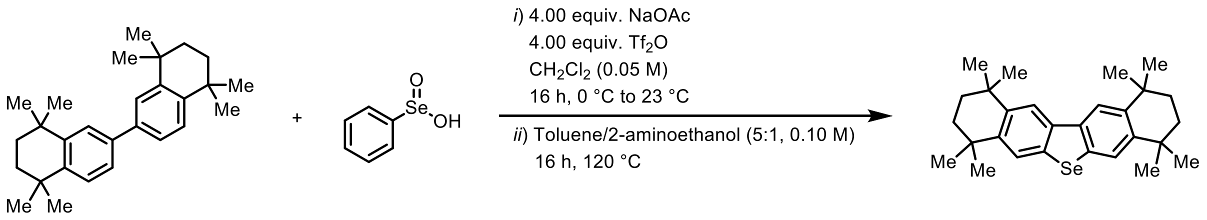

S2 | 2.00 equiv. | **78% yield** | **1d**

i) To an oven-dried 250-mL round-bottomed Schlenk flask equipped with a magnetic stirring bar, octamethyl-octahydro binaphthalene **S2** (1.87 g, 5.00 mmol, 1.00 equiv.), benzeneseleninic acid (1.91 g, 10.0 mmol, 2.00 equiv.) and sodium acetate (1.64 g, 20.0 mmol, 4.00 equiv.) were added. The flask was sealed with a septum, connected via a Tygon® tubing to a Schlenk line, and evacuated and purged with argon three times. CH_2Cl_2 (100 mL, 0.05 M) was added and the mixture was cooled to 0 °C using an ice bath. Trifluoromethanesulfonic anhydride (3.38 mL, 20.0 mmol, 4.00 equiv.) was added to the stirring (600 rpm) mixture dropwise using a 5.00-mL syringe. After the addition of trifluoromethanesulfonic anhydride was completed, the ice bath was removed, and the reaction mixture was stirred for additional 16 h at 23 °C. The resulting mixture was quenched with a saturated aq. $NaHCO_3$ solution (50 mL), extracted with EtOAc (3×50 mL), and washed with brine. The combined organic phase was dried over Na_2SO_4, and filtered by gravity using an 8-cm diameter funnel containing a fluted filter paper to a 500-mL round-bottomed flask. The solvent mixture was evaporated under reduced pressure and the residue was dried under high vacuo.

ii) Next, the 500-mL round-bottomed flask was equipped with a stirring bar, toluene (50 mL, 0.10 M), and 2-aminoethanol (10.3 mL, 170 mmol, 34.0 equiv) were added. The flask was equipped with a reflux condenser and the reaction mixture was stirred at 120 °C for 16 h using an oil bath. After 16 h, the flask was removed from the oil bath and allowed to cool to 23 °C (ca. 30 minutes). Then, the mixture was quenched with water (50 mL) and brine (50 mL). The resulting mixture was transferred to a 250-mL separation funnel and organic phase was separated. The aqueous phase was extracted with EtOAc (3×50 mL). The combined organic phase was dried over Na_2SO_4, and filtered by gravity using an 8-cm diameter funnel containing a fluted filter paper. Then, the resulting mixture was concentrated by rotary evaporation under reduced pressure. The residue was purified by flash column chromatography on silica gel using pure heptane as eluent to afford the desired product as colorless crystalline solid in 78% yield (1.75 g, 3.88 mmol).

R_f = 0.21 (heptane).

NMR Spectroscopy:

^{1}H NMR (400 MHz, $CDCl_3$, 23 ºC, δ): 7.96 (s, 2H), 7.74 (s, 2H), 1.76 (s, 8H), 1.42 (s, 12H), 1.35 (s, 12H).

^{13}C NMR (151 MHz, $CDCl_3$, 23 ºC, δ): 144.5, 142.2, 136.5, 136.3, 123.9, 120.2, 35.4, 35.28, 34.9,

34.6, 32.6, 32.3.

^{77}Se NMR (115 MHz, $CDCl_3$, 23 °C, δ): 426.8.

HRMS-EI(m/z) calc'd for $C_{28}H_{36}Se$ $[M]^+$, 452.1982; found, 452.1972; deviation: –2.3 ppm.

4-(2,6-Diisopropylphenyl)dibenzo[*b*,*d*]selenophene 1e

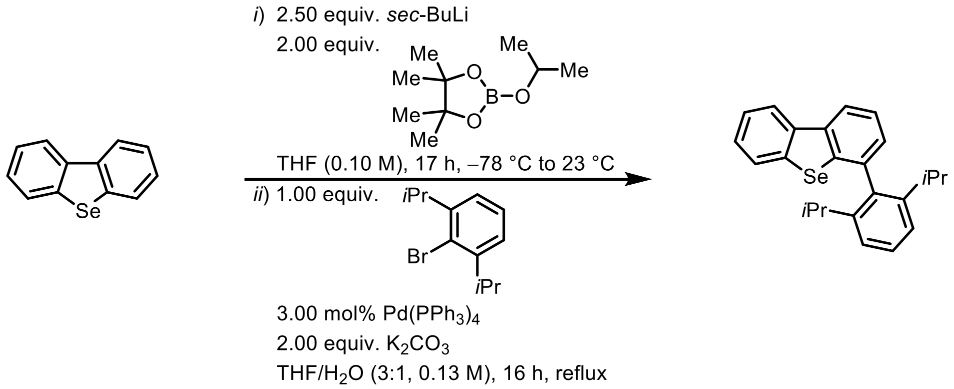

1a **42% yield** **1e**

i) To an oven-dried 100-mL round-bottomed Schlenk flask equipped with a magnetic stirring bar, dibenzo[*b*,*d*]selenophene (693 mg, 3.00 mmol, 1.00 equiv.) was added. The flask was sealed with a septum, connected via a Tygon® tubing to a Schlenk line, and evacuated and purged with argon three times. THF (30 mL, 0.10 M) was added and the mixture was cooled to –78 °C using liquid nitrogen and acetone bath. *Sec*-butyllithium solution in cyclohexane (5.36 mL, 1.40 M, 7.50 mmol, 2.50 equiv.) was added dropwise to a stirring (600 rpm) mixture using a 10.0-mL syringe at –78 °C. After the addition of *sec*-butyllithium solution, the acetone bath was removed and the reaction mixture was allowed to warm to room temperature during 1 h. After 1 h, dark red reaction mixture was cooled to –78 °C and 2-Isopropoxy-4,4,5,5-tetramethyl-1,3,2-dioxaborolane (1.84 mL, 9.00 mmol, 3.00 equiv.) was added dropwise using a 2.00-mL syringe, and resulting mixture was left stirring for 16 h slowly warming to room temperature. After 16 h, 5 mL of methanol was added, and the mixture was stirred for additional 10 min. Then, the resulting mixture was concentrated by rotary evaporation under reduced pressure and the crude yellow oil was used in the next step without further purification.

ii) To an oven-dried 50-mL Schlenk tube equipped with a magnetic stirring bar, tetrakis(triphenylphosphine)palladium(0) (106 mg, 0.09 mmol, 3.00 mol%) and potassium carbonate (1.24 g, 9.00 mmol, 3.00 equiv.) were added. The tube was sealed with a septum, connected via a Tygon® tubing to a Schlenk line, and evacuated and purged with argon three times. Crude oil from a previous step was dissolved in a dry THF (18 mL, 0.17 M) and the resulting solution was transferred to a Schlenk tube using a 20.0-mL syringe. 2-Bromo-1,3-diisopropylbenzene (637 µL, 3.00 mmol, 1.00 equiv.) and water (6 mL, 0.13 M) were added to a Schlenk tube, the septum was equipped with an Ar-filled balloon, and the reaction mixture was stirred with reflux for 16 h using an oil bath. After 16 h, the flask was removed from the oil bath and allowed to cool to 23 °C (ca. 20 minutes). Then, water (20 mL) was added, the resulting mixture was transferred to a 250-mL separation funnel. Organic phase was separated and aqueous phase was extracted with EtOAc (3×20 mL). The combined organic phase was

dried over Na_2SO_4, and filtered by gravity using an 8-cm diameter funnel containing a fluted filter paper. Then, the resulting mixture was concentrated by rotary evaporation under reduced pressure. The residue was purified by flash column chromatography on silica gel using pure heptane as eluent to afford the desired product as colorless crystalline solid in 42% yield (491 mg, 1.25 mmol) over two steps.

R*f* = 0.48 (heptane).

NMR Spectroscopy:

^{1}H NMR (400 MHz, $CDCl_3$, 23 °C, δ): 8.19 (d, *J* = 7.9 Hz, 1H), 8.14 (d, *J* = 7.9 Hz, 1H), 7.83 (d, *J* = 8.0 Hz, 1H), 7.57 (t, *J* = 7.5 Hz, 1H), 7.51 – 7.44 (m, 2H), 7.38 (t, *J* = 7.5 Hz, 1H), 7.30 (d, *J* = 7.9 Hz, 2H), 7.24 – 7.20 (m, 1H), 2.55 (hept, *J* = 6.9 Hz, 2H), 1.14 (d, *J* = 6.9 Hz, 6H), 1.07 (d, *J* = 6.9 Hz, 6H).

^{13}C NMR (151 MHz, $CDCl_3$, 23 °C, δ): 147.2, 142.4, 140.0, 139.0, 138.5, 138.4, 138.2, 128.9, 127.8, 126.9, 126.2, 125.3, 124.9, 123.3, 123.3, 121.3, 30.7, 25.3, 24.2.

^{77}Se NMR (101 MHz, $CDCl_3$, 23 °C, δ): 450.1.

HRMS-ESI-DI(m/z) calc'd for $C_{24}H_{24}Se$ $[M]^+$, 392.1039; found, 392.1035; deviation: –1.1 ppm.

4,4'-Bis(trifluoromethyl)-[1,1'-biphenyl]-2-amine (S3)

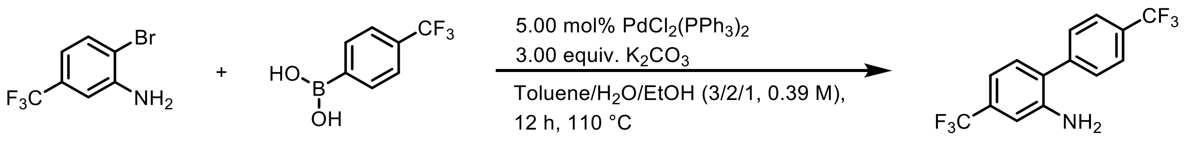

1.00 equiv. 1.00 equiv. **74% yield** **S3**

Caution: When performing reactions in pressurized systems (such as closed vials, pressure tubes, and autoclaves), a blast shield must be used to minimize personal damage in case of an accident.

To a 100-mL Schlenck tube equipped with a screw cap and a magnetic stir bar, 4-(trifluoromethyl)phenylboronic acid (1.34 g, 7.00 mmol, 1.00 equiv.), bis(triphenylphosphine)palladium(II) dichloride (246 mg, 0.35 mmol, 5.00 mol%), and potassium carbonate (2.90 g, 21.0 mmol, 3.00 equiv.) were added. The flask was sealed with a screw cap, connected via a Tygon® tubing to a Schlenk line, and evacuated and purged with argon three times. Then, toluene (9.0 mL), water (6.0 mL) and ethanol (3.0 mL) were added under flow of argon to the tube using a 10-mL syringes. 2-Bromo-5-(trifluoromethyl)aniline (1.00 mL, 7.00 mmol, 1.00 equiv.) was added flow of argon to the resulting mixture under using a 1.00-mL syringe. The pressure tube was sealed with a screw cap, ensuring a tight and secure seal and the reaction mixture was stirred at 110 °C for 12 h using an oil bath. After 12 h, the flask was removed from the oil bath and allowed to cool to 23 °C (ca. 30 minutes). After cooling down, the reaction was transferred to a 50-mL round bottom flask and ethanol was evaporated by rotary evaporation under reduced pressure. The residue was transferred to a 100-mL separation funnel, 20 mL of water was added, and organic phase was

separated. The aqueous phase was extracted with EtOAc (3×40 mL). The combined organic phase was washed with water (20 mL), washed with brine (20 mL), dried over Na_2SO_4, and filtered by gravity using an 8-cm diameter funnel containing a fluted filter paper. Then, the resulting mixture was concentrated by rotary evaporation under reduced pressure. The residue was purified by flash column chromatography on silica gel using EtOAc/heptane (1:20 (v/v)) as eluent to afford the desired product as a white solid in 74% yield (1.59 g, 5.20 mmol).

R_f = 0.25 (EtOAc/heptane (1:20 (v/v))).

NMR Spectroscopy:

^{1}H NMR (400 MHz, $CDCl_3$, 23 ºC, δ): 7.75 (d, J = 8.0 Hz, 2H), 7.58 (d, J = 8.0 Hz, 2H), 7.20 (d, J = 7.9 Hz, 1H), 7.08 (d, J = 7.9 Hz, 1H), 7.01 (s, 1H), 3.91 (s, 2H).

^{13}C NMR (101 MHz, $CDCl_3$, 23 ºC, δ): 144.0, 142.1, 131.5 (q, J = 32.7 Hz), 130.9, 130.2 (q, J = 32.7 Hz), 129.5, 129.0, 126.2 (q, J = 3.8 Hz), 124.2 (q, J = 272 Hz), 115.3 (q, J = 3.8 Hz), 112.3 (q, J = 3.8 Hz).

^{19}F NMR (377 MHz, $CDCl_3$, 23 ºC, δ): –62.64 (s,3F), –62.94 (s, 3H).

HRMS-ESI(m/z) calc'd for $C_{14}H_9F_6N$ $[M+H]^+$, 306.0712; found, 306.0709; deviation: –1.0 ppm.

2-Iodo-4,4'-bis(trifluoromethyl)-1,1'-biphenyl (S4)

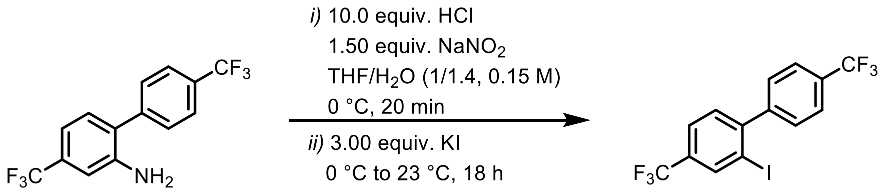

S3 **84% yield** **S4**

Caution: Reactions that generate gases (such as N_2) must be performed in well-ventilated fume hoods to minimize personal damage.

To a 100-mL round-bottom flask equipped with a magnetic stir bar, 4,4'-bis(trifluoromethyl)-[1,1'-biphenyl]-2-amine **S3** (1.37 g, 4.50 mmol, 1.00 equiv.), and THF (12 mL) were added. The mixture was stirred until the amine was completely dissolved, HCl (11.3 mL of 4 M aqueous solution, 45.0 mmol, 10.0 equiv.) was added, and the stirring (500 rpm) mixture was cooled to 0 °C using an ice bath. Then, solution of $NaNO_2$ (0.48 g, 6.75 mmol, 1.50 equiv.) in water (6 mL) was added dropwise to the reaction solution and the resulting mixture was stirred for 20 min at 0 °C. After 20 min, the solution of KI (2.26 g, 13.5 mmol, 3 .00 equiv.) in water (6 mL) was added dropwise to the reaction mixture. The reaction was allowed to warm up and was additionally stirred for 18 h at 23 °C. After 18 h, the reaction mixture was transferred to a 250-mL separation funnel, 40 mL of EtOAc was added, and the mixture was washed with 1 M aqueous $Na_2S_2O_3$ to remove the iodine. The organic phase was separated, washed with water (20 mL), washed with brine (20 mL), dried over Na_2SO_4, and filtered by gravity using an 8-cm diameter funnel containing a fluted filter paper. Then, the resulting mixture was concentrated by rotary

evaporation under reduced pressure. The residue was purified by flash column chromatography on silica gel using pure heptane as eluent to afford the desired product as a colorless oil in 84% yield (1.58 g, 3.79 mmol).

R*f* = 0.54 (heptane).

NMR Spectroscopy:

[1]**H NMR** (400 MHz, $CDCl_3$, 23 ºC, δ): 8.22 (s, 1H), 7.73 – 7.67 (m, 3H), 7.46 (d, *J* = 8.0 Hz, 2H), 7.39 (d, *J* = 8.0 Hz, 1H).

[13]**C NMR** (151 MHz, $CDCl_3$, 23 ºC, δ): 149.0, 146.5, 136.6 (q, *J* = 3.8 Hz), 131.6 (q, *J* = 33.3 Hz), 130.6 (q, *J* = 32.7 Hz), 130.1, 129.6, 127.8, 126.1 (q, *J* = 3.8 Hz), 125.4 (q, *J* = 3.8 Hz), 125.3 (q, *J* = 3.8 Hz), 124.2 (q, *J* = 272 Hz), 122.9 (q, *J* = 272 Hz), 97.8.

[19]**F NMR** (376 MHz, $CDCl_3$, 23 ºC, δ): –62.61 (s, 3F), –62.70 (s, 3F).

3,7-Bis(trifluoromethyl)dibenzo[*b*,*d*]iodol-5-ium trifluoromethanesulfonate (S5)

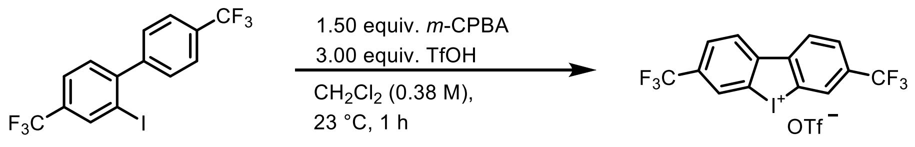

S4 **70% yield** **S5**

To an oven-dried 50-mL Schlenck flask equipped with a stirring bar, 2-iodo-4,4'-bis(trifluoromethyl)-1,1'-biphenyl **S4** (1.58 g, 3.79 mmol, 1.00 equiv.) was added. The tube was sealed with a septum, connected via a Tygon® tubing to a Schlenk line, and evacuated and purged with argon three times. Then, the septum was removed and 3-chloro-benzenecarboperoxoic acid (77% purity) (1.27 g, 5.68 mmol, 1.50 equiv.) was added portionwise under flow of argon to the stirring (400 rpm) mixture. The flask was sealed with a septum again and trifluoromethanesulfonic acid (1.00 mL, 11.4 mmol, 3.00 equiv.) was added dropwise using a 1.00-mL syringe. The resulting mixture was stirred at 23 °C for 1 h. After 1 h, the mixture was transferred to a 50-mL round bottom flask and concentrated by rotary evaporation under reduced pressure. Et_2O (10 mL) was added to the remained solid material. The resulting mixture was stirred for 20 min and then filtered. The obtained solid was washed with Et_2O (3×30 mL), and dried in high vacuo to afford the desired product as a white solid in 70% yield (1.50 g, 2.66 mmol).

NMR Spectroscopy:

[1]**H NMR** (400 MHz, DMSO-d_6, 23 ºC, δ): 8.85 (d, *J* = 8.3 Hz, 2H), 8.57 (d, *J* = 0.8 Hz, 2H), 8.31 (dd, *J* = 8.3, 0.8 Hz, 2H).

[13]**C NMR** (151 MHz, DMSO-d_6, 23 ºC, δ): 144.2, 131.0 (q, *J* = 33.0 Hz), 128.7, 127.9 (q, *J* = 3.7 Hz), 127.5 (q, *J* = 4.2 Hz), 123.5, 123.2 (q, *J* = 273 Hz), 120.6 (q, *J* = 321 Hz).

[19]**F NMR** (377 MHz, DMSO-d_6, 23 ºC, δ): –61.13, –77.79.

3,7-bis(trifluoromethyl)dibenzo[*b*,*d*]selenophene (1f)

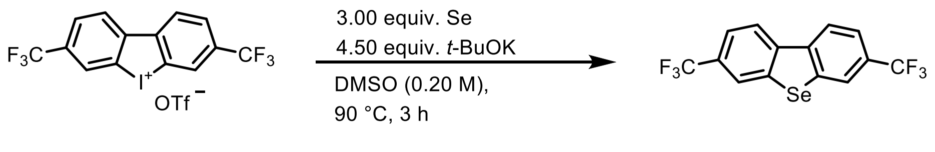

Caution: When performing reactions in pressurized systems (such as closed vials, pressure tubes, and autoclaves), a blast shield must be used to minimize personal damage in case of an accident.

To an oven-dried 50-mL Schlenck tube equipped with a stirring bar, 3,7-bis(trifluoromethyl)dibenzo[*b*,*d*]iodol-5-ium trifluoromethanesulfonate **S5** (564 mg, 1.00 mmol, 1.00 equiv.), selenium (239 mg, 3.00 mmol, 3.00 equiv.), and potassium *tert*-butoxide (505 mg, 4.50 mmol, 4.5 equiv.) were added. The tube was sealed with a septum, connected via a Tygon® tubing to a Schlenk line, and evacuated and purged with argon three times. Then, DMSO (5 mL) was added to the tube using a 5.0-mL syringe, the reaction mixture was heated at 90 °C using an oil bath and stirred (500 rpm) for 3 h. After 3 h, the tube was removed from the oil bath and allowed to cool to 23 °C (ca. 20 minutes), and the mixture was transferred to a 100-mL separation funnel. The aqueous phase was extracted with EtOAc (3×30 mL). The combined organic phase was dried over Na_2SO_4, and filtered by gravity using an 8-cm diameter funnel containing a fluted filter paper. Then, the resulting mixture was concentrated by rotary evaporation under reduced pressure. The residue was purified by flash column chromatography on silica gel using pure heptane as eluent to afford the desired product as a pale pink solid in 46% yield (168 mg, 0.46 mmol).

$\mathbf{R}_f$ = 0.43 (heptane).

NMR Spectroscopy:

^{1}H NMR (400 MHz, $CDCl_3$, 23 ºC, δ): 8.28 (d, *J* = 8.3 Hz, 2H), 8.21 (s, 2H), 7.76 (dd, *J* = 8.3, 1.0 Hz, 2H).

^{13}C NMR (101 MHz, $CDCl_3$, 23 ºC, δ): 140.61, 139.95, 130.0 (q, *J* = 32.7 Hz), 124.1 (q, *J* = 273 Hz), 123.9, 123.5 (q, *J* = 4.2 Hz), 122.3 (q, *J* = 3.6 Hz).

^{19}F NMR (376 MHz, $CDCl_3$, 23 ºC, δ): –61.93.

^{77}Se NMR (115 MHz, $CDCl_3$, 23 ºC, δ): 482.2.

HRMS-ESI(m/z) calc'd for $C_{14}H_5F_6Se$ $[M–H]^-$, 366.94661; found, 366.9464; deviation: –0.6 ppm.

Diphenylselenide (S6)

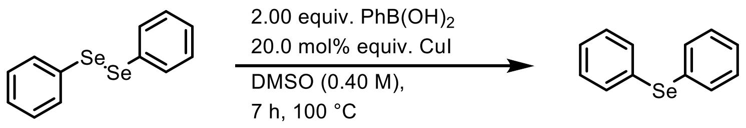

68% yield **S6**

To an oven-dried 50-mL round-bottomed flask equipped with a stirring bar, diphenyldiselenide (644 mg, 2.00 mmol, 1.00 equiv.), phenylboronic acid (498 mg, 4.00 mmol, 2.00 equiv.), and copper(I) iodide (76.2 mg, 0.40 mmol, 20.0 mol%) were added. DMSO (10 mL, 0.40 M) was added at 23 °C. The resulting mixture was heated to 100 °C using an oil bath and stirred at for 7 h. After 7 h, the flask was removed from the oil bath and allowed to cool to 23 °C (ca. 40 minutes). After cooling down, 50 mL of water were added, the reaction mixture was transferred to a 250-mL separatory funnel, and the mixture was extracted with EtOAc (3×20 mL). The combined organic phase was dried over Na_2SO_4, and filtered by gravity using an 8-cm diameter funnel containing a fluted filter paper. Then, the resulting mixture was concentrated by rotary evaporation under reduced pressure. The residue was purified by flash column chromatography on silica gel using gradient of eluent systems from pure heptane to EtOAc/heptane (1:20 (v/v)) to afford the desired product as a yellow oil in 68% yield (631 mg, 2.71 mmol).

R*f* = 0.39 (heptane).

NMR Spectroscopy:

^{1}**H NMR** (600 MHz, $CDCl_3$, 23 ºC, δ): 7.52 – 7.45 (m, 4H), 7.31 – 7.27 (m, 6H).

^{13}**C NMR** (125 MHz, $CDCl_3$, 23 ºC, δ): 133.1, 131.3, 129.5, 127.5.

^{77}**Se NMR** (101 MHz, $CDCl_3$, 23 ºC, δ): 416.4.

HRMS-EI(m/z) calc'd for $C_{12}H_{10}Se$ $[M]^+$, 233.9948; found, 233.9937; deviation: –4.7 ppm.

Dibenzylselenide (S7)

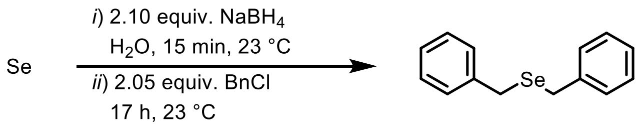

63% yield **S7**

i) To an oven-dried 25-mL Schlenk tube equipped with a magnetic stirring bar, Se powder (319 mg, 4.00 mmol, 1.00 equiv.) was added. The tube was sealed with a septum, connected via a Tygon® tubing to a Schlenk line, and evacuated and purged with argon three times. The septum was equipped with an Ar-filled balloon to compensate hydrogen gas overpressure. Then, water (4 mL) was added, and a solution of sodium borohydride (324 mg, 8.40 mmol, 2.10 equiv.) in 4 mL of water was added dropwise

using a 5.00-mL syringe to the stirring dispersion at 23 °C. After completion of gas evolution, complete dissolution of Se powder is observed.

ii) The resulting solution was stirred for additional 15 min and benzylchloride (940 µL, 8.20 mmol, 2.05 equiv.) was added using a 1.00-mL syringe. The reaction mixture was stirred for additional 17 h at 23 °C. Then, the reaction mixture was transferred to a 100-mL separatory funnel, and extracted with EtOAc (3×10 mL). The combined organic phase was dried over Na_2SO_4, and filtered by gravity using an 8-cm diameter funnel containing a fluted filter paper. Then, the resulting mixture was concentrated by rotary evaporation under reduced pressure. The residue was purified by recrystallization from pentane to afford the desired product as yellow crystals in 63% yield (661 mg, 2.53 mmol).

NMR Spectroscopy:

^{1}H NMR (400 MHz, $CDCl_3$, 23 °C, δ): 7.31 – 7.18 (m, 10H), 3.71 (s, 4H).

^{13}C NMR (125 MHz, $CDCl_3$, 23 °C, δ): 139.2, 129.0, 128.5, 126.7, 27.6.

^{77}Se NMR (101 MHz, $CDCl_3$, 23 °C, δ): 403.6.

HRMS-EI(m/z) calc'd for $C_{14}H_{14}Se$ $[M]^+$, 262.0261; found, 262.0256; deviation: –1.9 ppm.

3,7-Di-*tert*-butyldibenzo[*b*,*d*]thiophene (S8)

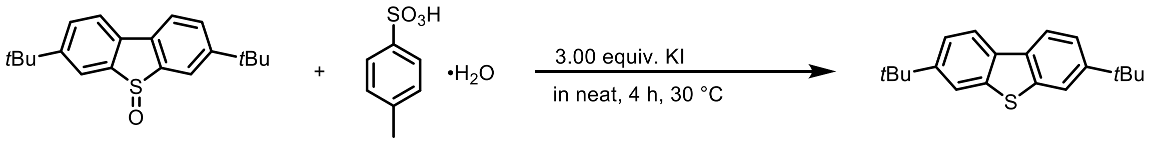

3.00 equiv. **13% yield** **S8**

Caution: When performing reactions in pressurized systems (such as closed vials, pressure tubes, and autoclaves), a blast shield must be used to minimize personal damage in case of an accident.

To a 20-mL borosilicate vial equipped with a magnetic stir bar, 3,7-di-*tert*-butyldibenzo[*b*,*d*]thiophene 5-oxide (1.00 g, 3.20 mmol, 1.00 equiv.), potassium iodide (1.510 g, 9.00 mmol, 3.00 equiv.), and p-toluolsulfonic acid monohydrate (1.71 g, 9.00 mmol, 3.00 equiv.) were added. The vial was capped and the reaction mixture was stirred at 30 °C for 4 h. After 4 h, 20 mL of sodium thiosulfate (0.10 M) aqueous solution was added. Then, the reaction mixture was transferred to a 100-mL separatory funnel, and extracted with heptane (3×20 mL). The combined organic phase was dried over Na_2SO_4, and filtered by gravity using an 8-cm diameter funnel containing a fluted filter paper. Then, the resulting mixture was concentrated by rotary evaporation under reduced pressure. The residue was purified by recrystallization from MeCN to afford the desired product as yellow crystals in 13% yield (120 mg, 0.41 mmol).

NMR Spectroscopy:

^{1}H NMR (400 MHz, $CDCl_3$, 23 °C, δ): 8.03 (d, J = 8.4 Hz, 2H), 7.83 (d, J = 1.6 Hz, 2H), 7.48 (dd, J = 8.4, 1.7 Hz, 2H), 1.41 (s, 18H).

^{13}C NMR (101 MHz, $CDCl_3$, 23 ºC, δ): 149.8, 139.6, 133.2, 122.4, 121.0, 119.2, 35.2, 31.7.

HRMS-EI(m/z) calc'd for $C_{20}H_{24}S$ $[M]^+$, 296.1599; found, 296.1589; deviation: –3.4 ppm.

Preparation of Cationic Selenuranes 2

Cationic selenurane [2a]SbF$_6$

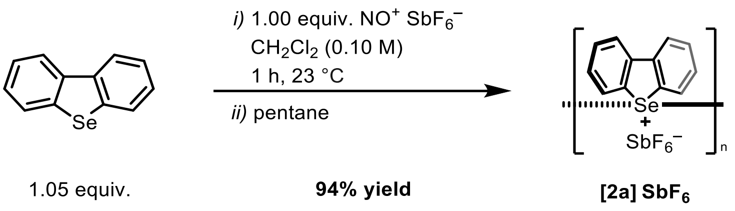

To an oven-dried 50-mL round-bottomed Schlenk tube equipped with a magnetic stirring bar, dibenzo[*b*,*d*]selenophene (485 mg, 2.10 mmol, 1.05 equiv.) was added. The tube was sealed with a septum, connected via a Tygon® tubing to a Schlenk line, and evacuated and purged with argon three times. CH_2Cl_2 (20 mL, 0.10 M) was added using a 20.0-mL syringe. Then, with a flow of argon the septum was removed and the nitrosonium hexafluoroantimonate (532 mg, 2.00 mmol, 1.00 equiv.) was added portionwise to the stirring (600 rpm) mixture, and the tube was sealed with a septum. The reaction mixture was stirred at room temperature for 1 h. After 1 h, pentane (20 mL) was added and the resulting mixture was stirred for additional 10 min. After 10 min, the stirring was turned off for to promote solid sedimentation. With a flow of argon the septum of the reaction tube was removed, equipped with a canula filter (Figure S1), and connected to a 250-mL round bottom flask (receiving flask). With a positive argon pressure, the reaction mixture was filtered, and washed with pentane (3×20 mL). The residue was dried under high vacuo for 2 h to afford the cationic selenurane **[2a]SbF$_6$** as a dark brown crystalline solid in 94% yield (874 mg, 1.87 mmol).

NMR Spectroscopy: See pages S75 and S100.

MALDI-HRMS-ESI-DI(m/z) calc'd for $C_{12}H_8Se$ $[M]^+$, 231.9788; found, 231.9784; deviation: –1.7 ppm.

HRMS-ESI-DI(m/z) calc'd for SbF_6 $[M]^-$, 234.8948; found, 234.8945; deviation: –1.3 ppm.

Elemental analysis calculated for $C_{12}H_8F_6SbSe$: C, 30.87; H, 1.73; found: C, 30.37; H, 1.88.

The content of the other elements was determined: N, 0.585; S, <0.02.

Cationic selenurane [2a]PF$_6$

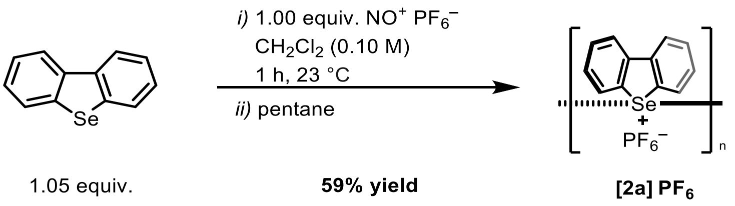

To an oven-dried 50-mL round-bottomed Schlenk tube equipped with a magnetic stirring bar, dibenzo[*b*,*d*]selenophene (243 mg, 1.05 mmol, 1.05 equiv.) was added. The tube was sealed with a septum, connected via a Tygon® tubing to a Schlenk line, and evacuated and purged with argon three times. CH_2Cl_2 (10 mL, 0.10 M) was added using a 10.0-mL syringe. Then, with a flow of argon the septum was removed and the nitrosonium hexafluorophosphate (184 mg, 1.00 mmol, 1.00 equiv.) was added portionwise to the stirring (600 rpm) mixture, and the tube was sealed with a septum. The reaction mixture was stirred at room temperature for 1 h. After 1 h, pentane (10 mL) was added and the resulting mixture was stirred for additional 10 min. After 10 min, the stirring was turned off for to promote solid sedimentation. With a flow of argon the septum of the reaction tube was removed, equipped with a canula filter (Figure S1), and connected to a 100-mL round bottom flask (receiving flask). With a positive argon pressure, the reaction mixture was filtered, and washed with pentane (3×10 mL). The residue was dried under high vacuo for 2 h to afford the cationic selenurane **[2a]PF$_6$** as a bright brown crystalline solid in 59% yield (220 mg, 0.585 mmol).

NMR Spectroscopy: See pages S62 and S87.

Cationic selenurane [2a]BF$_4$

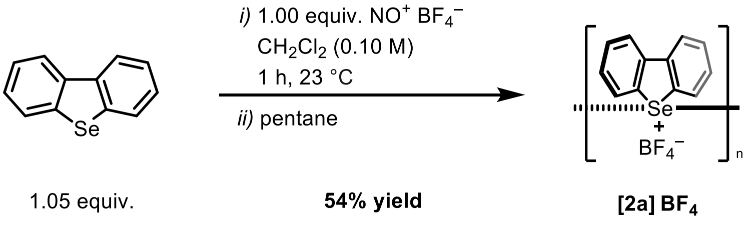

To an oven-dried 250-mL round-bottomed Schlenk flask equipped with a magnetic stirring bar, dibenzo[*b*,*d*]selenophene (1.58 g, 6.83 mmol, 1.05 equiv.) was added. The flask was sealed with a septum, connected via a Tygon® tubing to a Schlenk line, and evacuated and purged with argon three times. CH_2Cl_2 (65 mL, 0.10 M) was added. Then, with a flow of argon the septum was removed and the nitrosonium tetrafluoroborate (759 mg, 6.50 mmol, 1.00 equiv.) was added portionwise to the stirring (600 rpm) mixture, and the flask was sealed with a septum. The reaction mixture was stirred at room temperature for 1 h. After 1 h, pentane (65 mL) was added and the resulting mixture was stirred for additional 10 min. After 10 min, the stirring was turned off for to promote solid sedimentation. With a flow of argon the septum of the reaction flask was removed, equipped with a canula filter (Figure S1), and connected to a 250-mL round bottom flask (receiving flask). With a positive argon pressure, the reaction mixture was filtered, and washed with pentane (3×60 mL). The residue was dried under high vacuo for 2 h to afford the cationic selenurane **[2a]BF$_4$** as brown crystalline solid in 54% yield (1.11 g, 3.50 mmol).

NMR Spectroscopy: See pages S75 and S100.

Cationic selenurane [2b]SbF_6

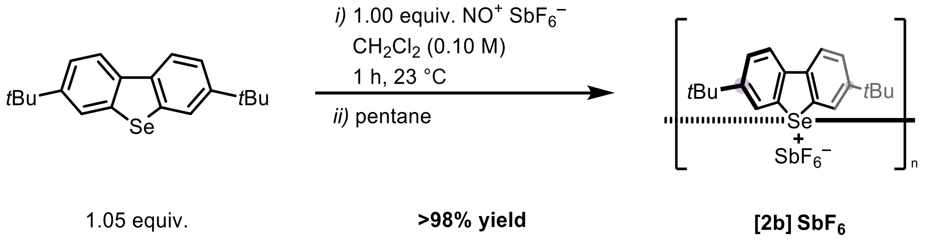

To an oven-dried 50-mL round-bottomed Schlenk tube equipped with a magnetic stirring bar, 3,7-di-*tert*-butyldibenzo[*b*,*d*]selenophene (793 mg, 2.31 mmol, 1.05 equiv.) was added. The tube was sealed with a septum, connected via a Tygon® tubing to a Schlenk line, and evacuated and purged with argon three times. CH_2Cl_2 (22 mL, 0.10 M) was added. Then, with a flow of argon the septum was removed and the nitrosonium hexafluoroantimonate (585 mg, 2.20 mmol, 1.00 equiv.) was added portionwise to the stirring (600 rpm) mixture, and the tube was sealed with a septum. The reaction mixture was stirred at room temperature for 1 h. Simultaneously, a receiving 100-mL Schlenk flask was sealed with a septum, connected via a Tygon® tubing to a Schlenk line, and evacuated and purged with argon three times. After 1 h of stirring, the flasks were connected via canula, the receiving flask was cooled down using a low form dewar flask cooled with a liquid nitrogen, and the solvent was evaporated to the receiving flask. The canula was removed. Then, pentane (10 mL) was added to the reaction tube, and the mixture was stirred for additional 5 min. With a flow of argon the septum of the reaction tube was removed and equipped with a canula filter (Figure S1), and connected to a 100-mL round bottom flask (second receiving flask). With a positive argon pressure, the reaction mixture was filtered, and washed with pentane (3×20 mL). The residue was dried under high vacuo for 2 h to afford the cationic selenurane **[2b]**$\mathbf{SbF_6}$ as a dark purple solid in 99% yield (1.27 g, 2.18 mmol).

NMR Spectroscopy: See pages S75 and S100.

LDI-HRMS-ESI-DI(m/z) calc'd for $C_{20}H_{24}Se$ $[M]^+$, 344.1042; found, 344.1034; deviation: –2.3 ppm.

HRMS-ESI(m/z) calc'd for SbF_6 $[M]^-$, 234.8948; found, 234.8948; deviation: 0.0 ppm.

Elemental analysis calculated for $C_{20}H_{24}F_6SbSe$: C, 41.48; H, 4.18; found: C, 42.68; H, 4.51.

The content of the other elements was determined: N, 0.378; O, 3.82; S, <0.02.

Cationic selenurane [2c]SbF_6

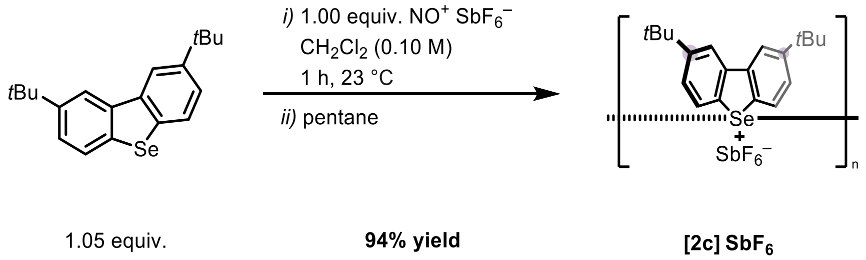

To an oven-dried 100-mL round-bottomed Schlenk flask equipped with a magnetic stirring bar, 2,8-di-*tert*-butyldibenzo[*b*,*d*]selenophene (793 mg, 2.31 mmol, 1.05 equiv.) was added. The flask was sealed with a septum, connected via a Tygon® tubing to a Schlenk line, and evacuated and purged with argon three times. CH_2Cl_2 (50 mL, 0.10 M) was added. Then, with a flow of argon the septum was removed and the nitrosonium hexafluoroantimonate (1.33 g, 5.00 mmol, 1.00 equiv.) was added portionwise to the stirring (600 rpm) mixture, and the flask was sealed with a septum. The reaction mixture was stirred at room temperature for 1 h. Simultaneously, a receiving 100-mL Schlenk flask was sealed with a septum, connected via a Tygon® tubing to a Schlenk line, and evacuated and purged with argon three times. After 1 h of stirring, the flasks were connected via canula, the receiving flask was cooled down using a low form dewar flask cooled with a liquid nitrogen, and the solvent was evaporated to the receiving flask. The canula was removed. Then, pentane (50 mL) was added to the reaction flask, and the mixture was stirred for additional 5 min. With a flow of argon the septum of the reaction flask was removed and equipped with a canula filter (Figure S1), and connected to a 250-mL round bottom flask (second receiving flask). With a positive argon pressure, the reaction mixture was filtered, and washed with pentane (3×50 mL). The residue was dried under high vacuo for 2 h to afford the cationic selenurane **[2c]SbF₆** as a dark purple solid in 99% yield (2.88 g, 4.97 mmol).

NMR Spectroscopy: See pages S75 and S100.

LDI-HRMS-ESI-DI(m/z) calc'd for $C_{20}H_{24}Se$ $[M]^+$, 344.1042; found, 344.1033; deviation: –2.6 ppm.

HRMS-ESI(m/z) calc'd for SbF_6 $[M]^-$, 234.8948; found, 234.8946; deviation: –0.9 ppm.

Elemental analysis calculated for $C_{20}H_{24}F_6SbSe$: C, 41.48; H, 4.18; found: C, 40.84; H, 4.14.

The content of the other elements was determined: N, 0.638; S, <0.02.

Cationic selenurane [2d] SbF_6

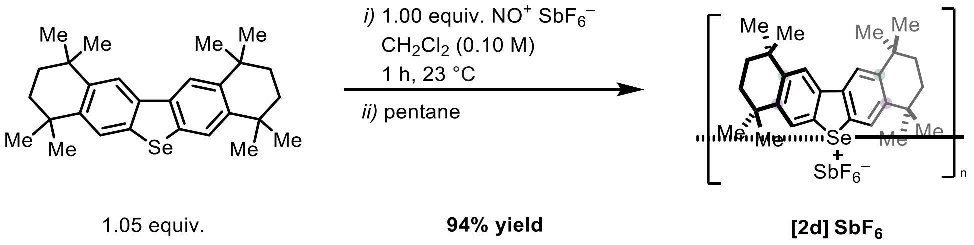

To an oven-dried 50-mL round-bottomed Schlenk tube equipped with a magnetic stirring bar, octamethyl-octahydro dinaphthoselenophene (1.19 g, 2.63 mmol, 1.05 equiv.) was added. The tube was sealed with a septum, connected via a Tygon® tubing to a Schlenk line, and evacuated and purged with argon three times. CH_2Cl_2 (25 mL, 0.10 M) was added. Then, with a flow of argon the septum was removed and the nitrosonium hexafluoroantimonate (664 mg, 2.50 mmol, 1.00 equiv.) was added portionwise to the stirring (600 rpm) mixture, and the tube was sealed with a septum. The reaction mixture was stirred at room temperature for 1 h. Simultaneously, a receiving 100-mL Schlenk flask was

sealed with a septum, connected via a Tygon® tubing to a Schlenk line, and evacuated and purged with argon three times. After 1 h of stirring, the flasks were connected via canula, the receiving flask was cooled down using a low form dewar flask cooled with a liquid nitrogen, and the solvent was evaporated to the receiving flask. The canula was removed. Then, pentane (25 mL) was added to the reaction tube, and the mixture was stirred for additional 5 min. With a flow of argon the septum of the reaction tube was removed and equipped with a canula filter (Figure S1), and connected to a 100-mL round bottom flask (second receiving flask). With a positive argon pressure, the reaction mixture was filtered, and washed with pentane (3×20 mL). The residue was dried under high vacuo for 2 h to afford the cationic selenurane **[2c]SbF$_6$** as a dark blue crystalline solid in 94% yield (1.61 g, 2.34 mmol).

NMR Spectroscopy: See pages S75 and S100.

LDI-HRMS-ESI-DI(m/z) calc'd for $C_{28}H_{36}Se$ $[M]^+$, 452.1984; found, 452.1970; deviation: –3.1 ppm.

HRMS-ESI(m/z) calc'd for SbF_6 $[M]^-$, 234.8948; found, 234.8948; deviation: 0.0 ppm.

Elemental analysis calculated for $C_{28}H_{36}F_6SbSe$: C, 48.93; H, 5.28; found: C, 47.78; H, 5.01.

The content of the other elements was determined: N, <0.05; S, <0.02.

Failed substrates

According to the general procedure for the synthesis of cationic selenuranes (pages S13-S15) substrates **1e**, **1f**, **S6**, **S7**, or **S8** were introduced in the reaction mixture and oxidized with nitrosonium hexafluoroantimonate (Figure S5). In these reactions, dark solutions formed when mixed. However, no defined products (complex mixtures of products) or starting materials were obtained.

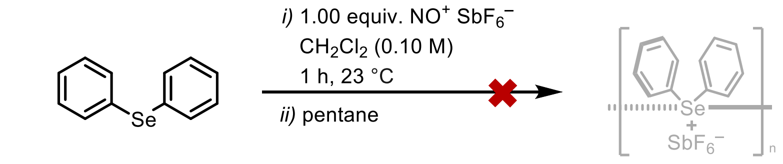

S6, 1.05 equiv. **complex mixture**

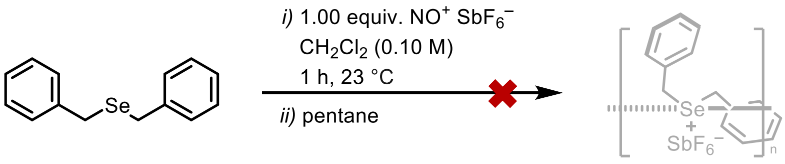

S7, 1.05 equiv. **complex mixture**

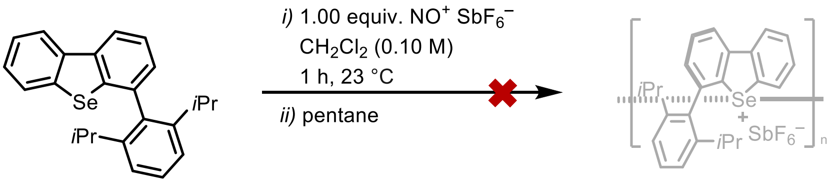

1e, 1.05 equiv. **32% of starting material recovered**

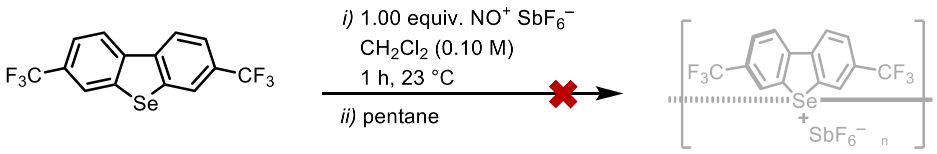

1f, 1.05 equiv. **74% of starting material recovered**

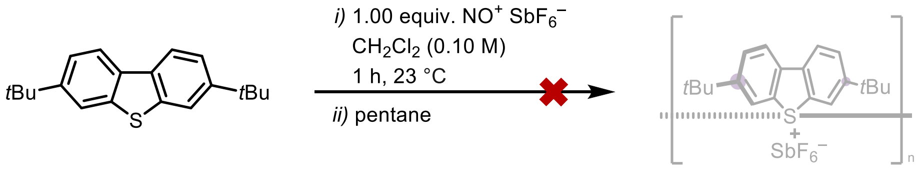

S8, 1.05 equiv. **complex mixture**

Figure S5. Failed substrates for the oxidation with nitrosonium hexafluoroantimonate.

The reaction with commercial dibenzo[*b*,*d*]thiophene was performed according to the following procedure.

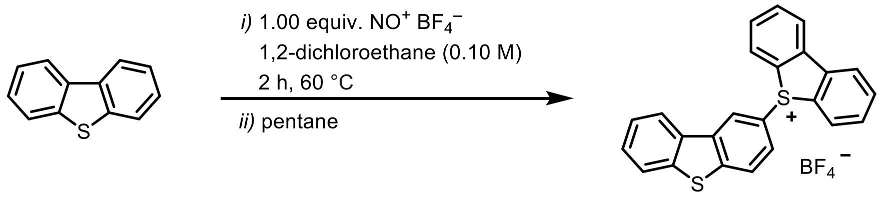

1.05 equiv. **3% yield** **S–[5a]BF$_4$**

To an oven-dried 50-mL round-bottomed Schlenk tube equipped with a magnetic stirring bar, dibenzo[*b*,*d*]thiophene (195 mg, 1.05 mmol, 1.05 equiv.) was added. The tube was sealed with a septum, connected via a Tygon® tubing to a Schlenk line, and evacuated and purged with argon three times. 1,2-Dichloroethane (25 mL, 0.10 M) was added. Then, with a flow of argon the septum was removed and the nitrosonium tetrafluoroborate (117 mg, 1.00 mmol, 1.00 equiv.) was added portionwise to the stirring (600 rpm) mixture, and the tube was sealed with a septum. The reaction mixture was stirred at 60 °C using oil bath for 2 h. After 2 h, pentane (10 mL) was added and the resulting mixture was stirred for additional 10 min. After 10 min, the stirring was turned off for to promote solid sedimentation. With a flow of argon the septum of the reaction tube was removed, equipped with a canula filter (Figure S1), and connected to a 100-mL round bottom flask (receiving flask). With a positive argon pressure, the reaction mixture was filtered. The solid residue was dried under high vacuo for 2 h to afford the mixture containing [2,5'-bidibenzo[*b*,*d*]thiophen]-5'-ium salt **S–[5a]BF$_4$**. The filtrate mixture was concentrated under reduced pressure affording a mixture containing dibenzo[*b*,*d*]thiophene. Both mixtures were characterized using ^{1}H and NMR spectroscopy (Figure S6). Weighted samples of mixtures were solubilized in CD_3CN and transferred to the NMR tubes, and weighted mass of CH_2Br_2 was added to the NMR tubes as an internal standard.

The yields were determined by comparing the integration of the CH_2Br_2 signal at 5.08 ppm (s, 2H) to the signal of **S–[5a]BF$_4$** at 8.42 ppm (d, 1H) for the spectrum of [2,5'-bidibenzo[*b*,*d*]thiophen]-5'-ium salt and 8.25 ppm (m, 2H) for the spectrum of dibenzo[*b*,*d*]thiophene.

89% of dibenzo[b,d]thiophene was recovered.

The yield of **S–[5a]BF$_4$** *was 3%.*

The ^{1}H NMR spectrum of the product **S–[5a]BF$_4$** is comparable to the previously reported one.[66] The formation of **S–[5a]BF$_4$** was further confirmed via Mass-spectrometry analysis MS-DI (Figure S7).

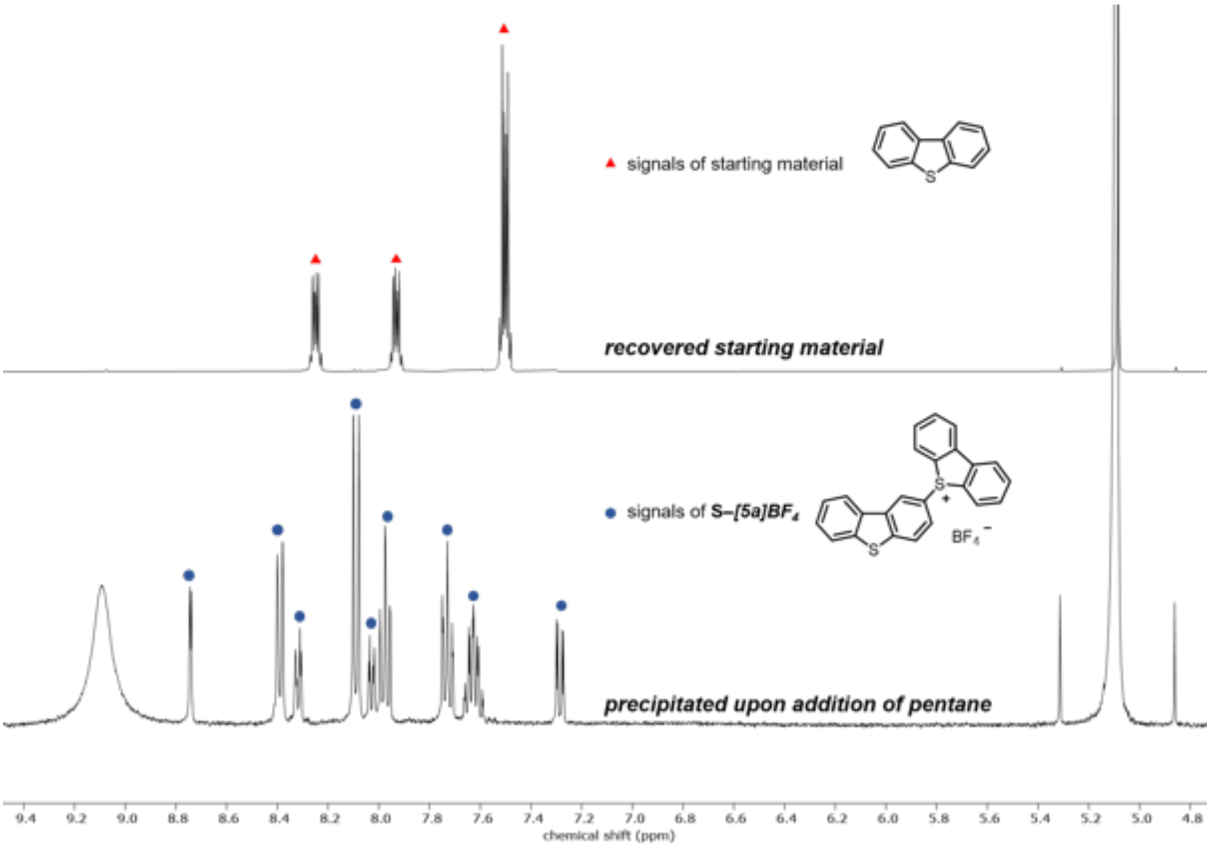

Figure S6. The two isolated products of the reaction of dibenzo[*b*,*d*]thiophene with nitrosonium tetrafluoroborate. **Top:** recovered starting material (89%). **Bottom:** irreversible formation of the head-to-tail dimer (3%).

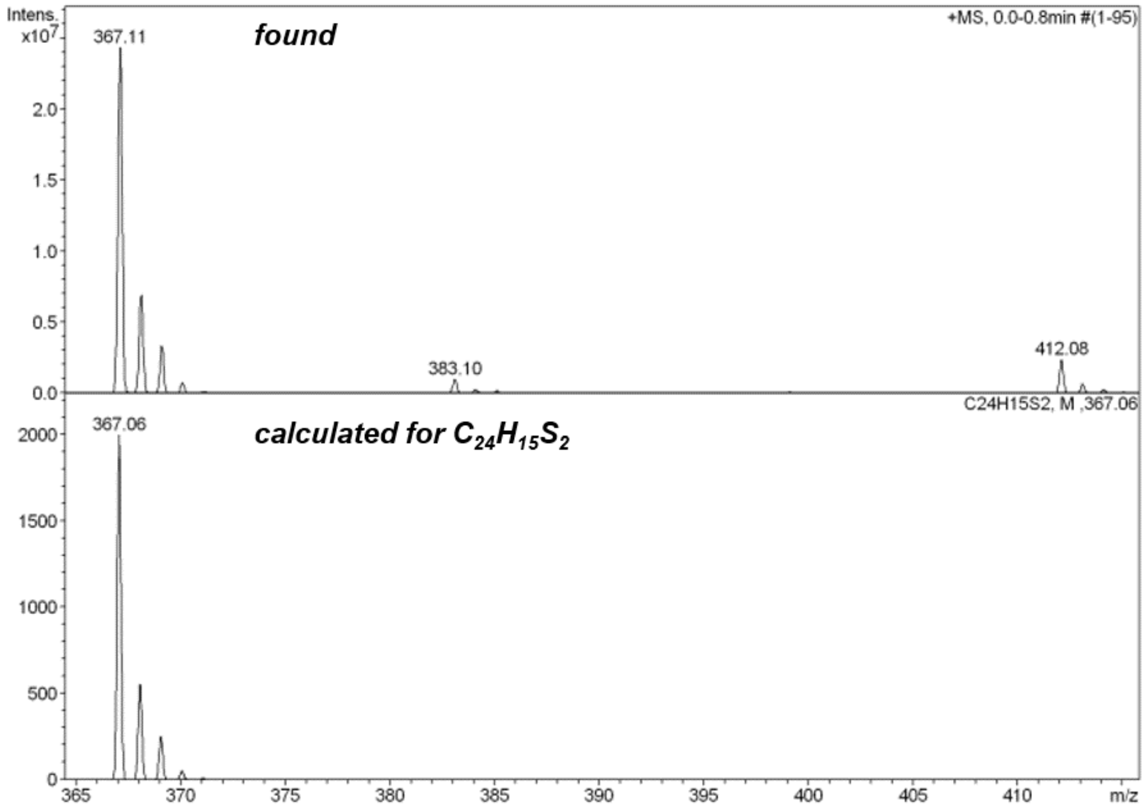

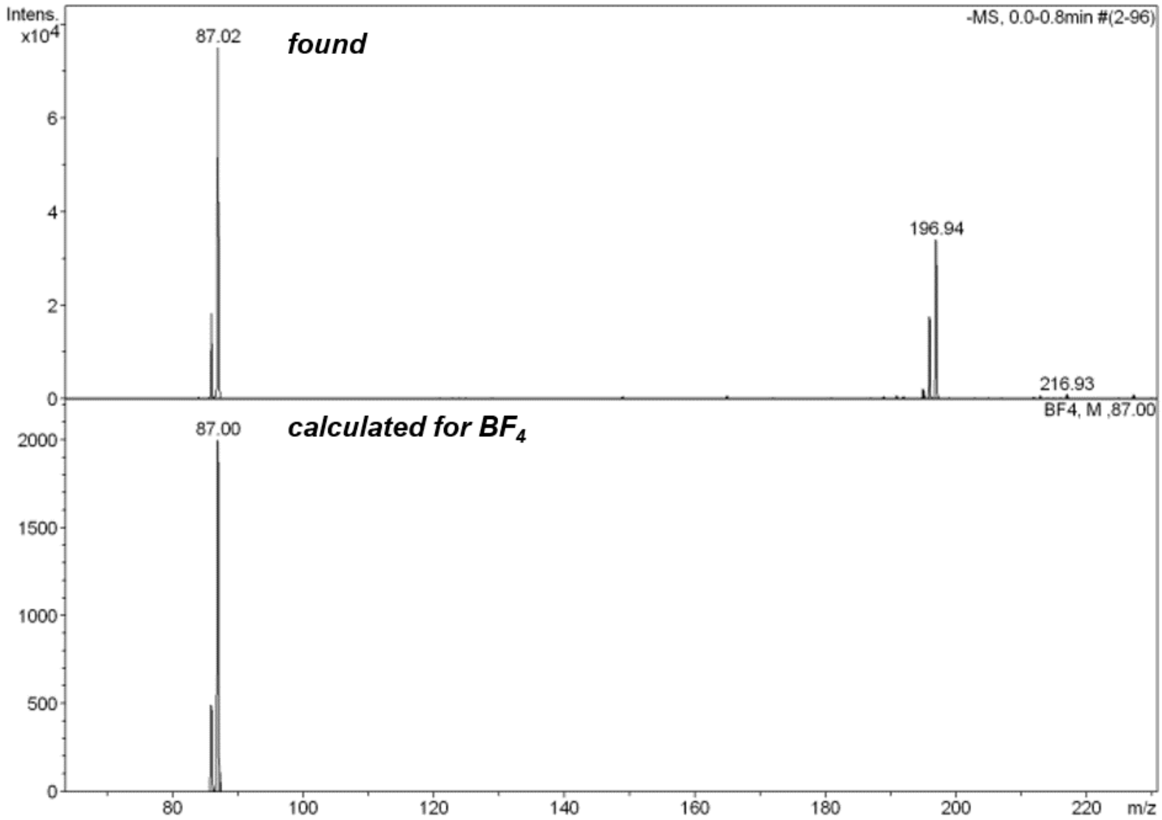

Figure S7. Mass spectra of the product **S–[5a]BF₄** in positive ion mode (top) and negative ion modes (bottom).

PREPARATION OF SELENONIUM SALT SE–[5A]OTF

Dibenzo[*b*,*d*]selenophene 5-oxide S6

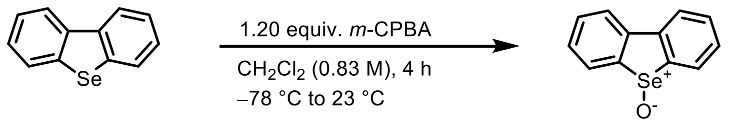

71% yield **S9**

To an 250-mL round bottom flask equipped with a magnetic stirring bar, dibenzo[*b*,*d*]selenophene (2.31 g, 10.0 mmol, 1.00 equiv.) was added. CH_2Cl_2 (120 mL, 0.83 M) was added and the mixture was cooled to –78 °C using liquid nitrogen and acetone bath. 3-Chloroperoxybenzoic acid (77% purity) (2.69 g, 12.0 mmol, 1.20 equiv.) was added portionwise to a stirring (400 rpm) mixture at –78 °C. Then, the cooling bath was removed and the reaction mixture was stirring for 4 h. After 4 h, a saturated aqueous solution of $NaHCO_3$ (100 mL) was added, and the mixture was stirred for additional 5 min. The resulting mixture was transferred to a 500-mL separation funnel and organic phase was separated. The organic phase was washed with brine (100 mL), dried over Na_2SO_4, and filtered by gravity using an 8-cm diameter funnel containing a fluted filter paper. Then, the resulting mixture was concentrated by rotary evaporation under reduced pressure. The residue was purified by recrystallization from CH_3CN to afford the desired product as colorless crystals in 71% yield (1.76 g, 7.13 mmol).

NMR Spectroscopy:

^{1}H NMR (400 MHz, $CDCl_3$, 23 °C, δ): 7.93 (dd, *J* = 7.6, 1.2 Hz, 2H), 7.84 (dd, *J* = 7.6, 1.2 Hz, 2H), 7.61 (td, *J* = 7.6, 1.2 Hz, 2H), 7.48 (td, *J* = 7.6, 1.2 Hz, 2H).

^{13}C NMR (151 MHz, $CDCl_3$, 23 °C, δ): 145.8, 140.9, 132.6, 130.1, 129.1, 123.2.

^{77}Se NMR (115 MHz, $CDCl_3$, 23 °C, δ): 924.4.

HRMS-ESI(m/z) calc'd for $C_{12}H_8SeNa$ $[M+Na]^+$, 270.9633; found, 270.9636; deviation: +1.2 ppm

[2,5'-Bidibenzoselenophen]-5'-ium trifluoromethanesulfonate Se–[5a]OTf

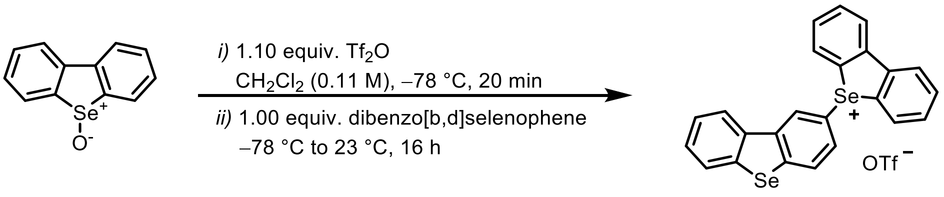

S9 **92% yield** **Se–[5a]OTf**

To an oven-dried 50-mL Schlenk tube equipped with a magnetic stirring bar, dibenzo[*b*,*d*]selenophene 5-oxide **S9** (420 mg, 1.70 mmol, 1.00 equiv.) was added. The tube was sealed with a septum, connected via a Tygon® tubing to a Schlenk line, and evacuated and purged with argon three times. CH_2Cl_2 (15.3 mL, 0.11 M) was added and the mixture was cooled to –78 °C using dry ice and acetone bath. Trifluoromethanesulfonic anhydride (316 µL, 1.87 mmol, 1.10 equiv.) was added dropwise to a stirring

(400 rpm) mixture using a 1.00-mL syringe. After the addition of trifluoromethanesulfonic anhydride, the reaction mixture was stirred for 20 min at –78 °C, the septum was exchanged for the funnel under argon flow, dibenzo[*b*,*d*]selenophene (393 mg, 1.70 mmol, 1.00 equiv.) was added portionwise to a stirring mixture, and the tube was sealed with septum again. The acetone bath was removed and the reaction mixture was stirred for 16 h. After 16 h, the resulting mixture was concentrated by rotary evaporation under reduced pressure, and residual solid was washed with Et_2O (2×20 mL). The residue was purified by flash column chromatography on silica gel using MeOH/CH_2Cl_2 (1:20 (v/v)) as eluent to afford the desired product as colorless crystalline solid in 92% yield (950 mg, 1.56 mmol).

$\mathbf{R}_f$ = 0.31 (MeOH/CH_2Cl_2 (1:20 (v/v))).

NMR Spectroscopy:

^{1}H NMR (700 MHz, DMSO-d_6, 23 ºC, δ): 8.79 (d, *J* = 1.9 Hz, 1H), 8.44 (dd, *J* = 7.8, 1.3 Hz, 2H), 8.40 (dd, *J* = 8.0, 1.1 Hz, 2H), 8.18 – 8.13 (m, 3H), 7.88 (dd, *J* = 7.7, 1.1 Hz, 2H), 7.71 (dd, *J* = 7.9, 1.3 Hz, 2H), 7.59 (dd, *J* = 7.6, 1.0 Hz, 1H), 7.52 (dd, *J* = 7.6, 1.1 Hz, 1H), 7.04 (dd, *J* = 8.6, 1.9 Hz, 1H).

^{1}H NMR (600 MHz, CD_3CN, 23 ºC, δ): 8.56 (d, J = 1.9 Hz, 1H), 8.30 (dd, J = 7.9, 0.9 Hz, 2H), 8.21 – 8.16 (m, 1H), 8.16 – 8.13 (m, 2H), 8.02 – 7.98 (m, 2H), 7.89 (dt, J = 7.9, 1.0 Hz, 2H), 7.67 (dt, J = 7.9, 1.3 Hz, 2H), 7.56 – 7.52 (m, 1H), 7.50 (dt, J = 7.8, 1.3 Hz, 1H), 7.09 (dd, J = 8.6, 1.9 Hz, 1H).

^{13}C NMR (151 MHz, DMSO-d_6, 23 ºC, δ): 143.6, 141.2, 140.1, 130.0, 137.0, 136.2, 132.9, 131.0, 129.7, 129.7, 128.8, 128.3, 126.9, 125.8, 125.0, 124.9, 124.9, 123.0, 121.2 (q, *J* = 322 Hz).

^{19}F NMR (659 MHz, DMSO-d_6, 23 ºC, δ): –77.7.

^{77}Se NMR (115 MHz, CD_3CN, 23 ºC, δ): 505.1, 477.7.

HRMS-ESI(m/z) calc'd for $C_{24}H_{15}Se_2$ $[M]^+$, 462.9501; found, 462.9506; deviation: +1.5 ppm.

HRMS-ESI(m/z) calc'd for SO_3CF_3 $[M]^-$, 148.9526; found, 148.9528; deviation: +1.3 ppm.

Stability of cationic selenurane [2a]SbF$_6$ in different solvents

To examine suitable solvents for the study of selenurane reactivity solutions of cationic selenurane **[2a]SbF$_6$** were examined:

Experimental procedure: In ten different 4-mL borosilicate vials flushed with argon for 30 s cationic selenurane **[2a]SbF$_6$** (46.7 mg, 0.100 mmol, 1.00 equiv.) was added with a flow of argon, and the vials were capped with a polypropylene caps equipped with silicon/PTFE septa. Then, CH_2Cl_2 (2.00 mL, 0.05 M) was added to each vial using a 2.00-mL syringe, formation of a blue solutions was observed (Figure S8 A.). One vial was used as control and no additional solvent was added. To the other nine vials a co-solvent (0.500 mmol, 5.00 equiv.) was added using a syringe.

The structures of the used co-solvents are shown in Figure S8. Namely, water, propan-2-ol, 1,1,1,3,3,3-hexafluoropropan-2-ol, diethyl ether, 1,4-dioxane, tetrahydrofuran, dimethyl sulfoxide, methanol, and acetonitrile.

Vials were shaken, and left for 1 h. After 1h, color change was observed for water, propan-2-ol, diethyl ether, tetrahydrofuran, dimethyl sulfoxide, and methanol (Figure S8 B.).

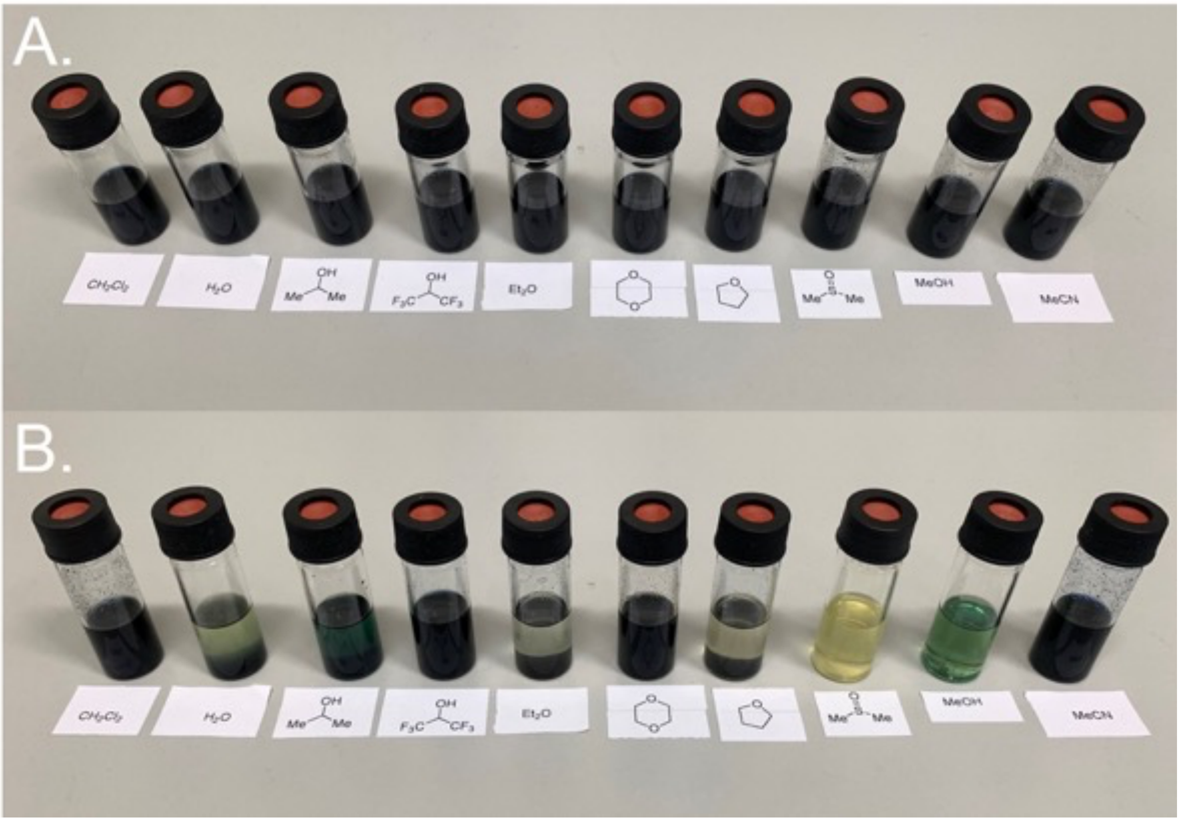

Figure S8. Stability study of cationic selenurane **[2a]SbF$_6$** in the presence of different co-solvents **A.** Vials with cationic selenurane **[2a]SbF$_6$** dissolved in CH_2Cl_2. **B.** Solutions of cationic selenurane **[2a]SbF$_6$** in CH_2Cl_2 after addition of 5.00 equiv. of different co-solvents.

Reactivity with propan-2-ol, 1,4-dioxane, and tetrahydrofuran

The reaction side-products of cationic selenurane **[2a]SbF$_6$** with propan-2-ol, 1,4-dioxane, and tetrahydrofuran were analyzed using ^{1}H NMR spectroscopy. The mixtures were concentrated under reduced pressure, dissolved in CD_3CN, transferred to the NMR tube, and CH_2Br_2 (7.1 μL, 0.10 mmol, 1.0 equiv.) was added to the NMR tube as an internal standard.

In case of reactions with propan-2-ol and tetrahydrofuran, dibenzo[*b*,*d*]selenophene **1a** was identified as the main product, and the yields were determined by comparing the integration of the CH_2Br_2 signal at 5.08 ppm (s, 2H) to the signal of **1a** at 7.42 ppm (dt, 2H) (Figure S9).

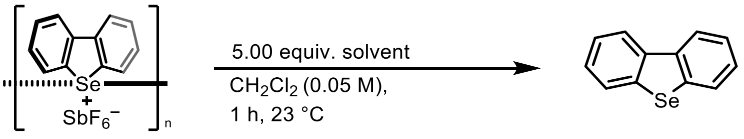

1a

**solvent**	yield of **1a, %**
propan-2ol	77
THF	70

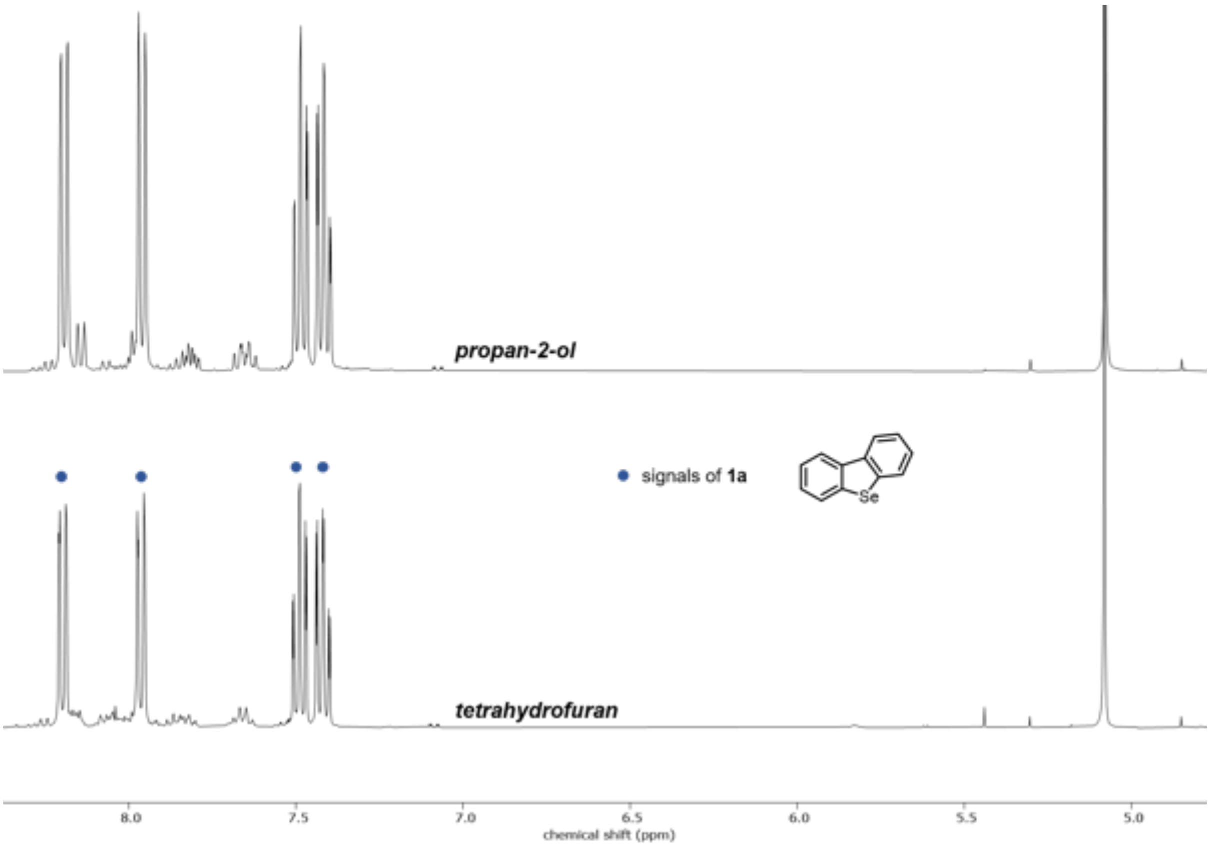

Figure S9. ^{1}H NMR spectra of the reactions of **[2a]SbF$_6$** with propan-2-ol and tetrahydrofuran.

For the reactions with propan-2-ol and tetrahydrofuran the yields were 77 and 70%, respectively.

A different product was obtained in the reaction with 1,4-dioxane. Despite the persistent blue color of the solution after addition of 1,4-dioxane, **Se–[5a]SbF₆** was observed (Figure S10). We confirmed the formation of **Se–[5a]SbF₆** by ^{1}H and ^{77}Se NMR spectra comparison between the reaction crude and a pure sample of **Se–[5a]OTf** (Figures S10 and S11).

The yield of the reaction was determined by comparing the integration of the CH_2Br_2 signal at 5.08 ppm (s, 2H) to the signal of **Se–[5a]OTf** at 8.46 ppm (d, 1H). The yield of the irreversible dimerization reaction was 64%.

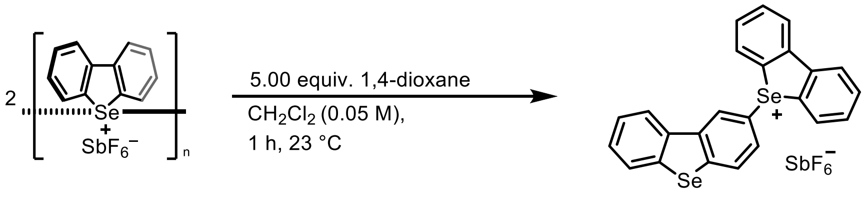

64% yield **Se–[5a]SbF₆**

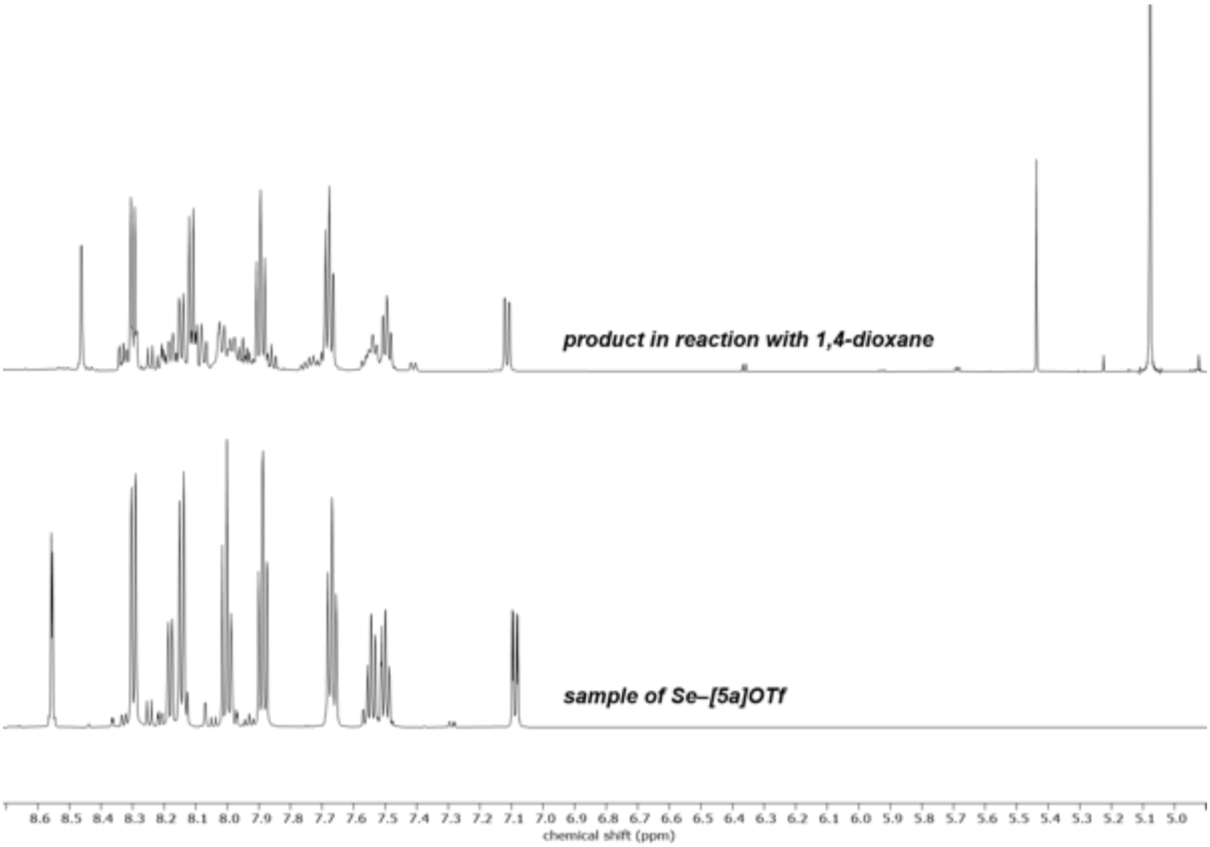

Figure S10. ^{1}H NMR spectra of the crude reaction of dimerization of **[2a]SbF₆** after addition of 1,4-dioxane and compound **Se–[5a]OTf** in CD_3CN. **Top:** crude reaction spectrum after addition of 1,4-dioxane in CD_3CN. **Bottom:** original sample of **Se–[5a]OTf** in CD_3CN.

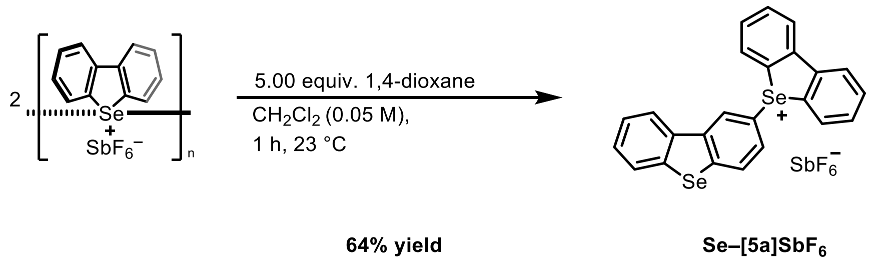

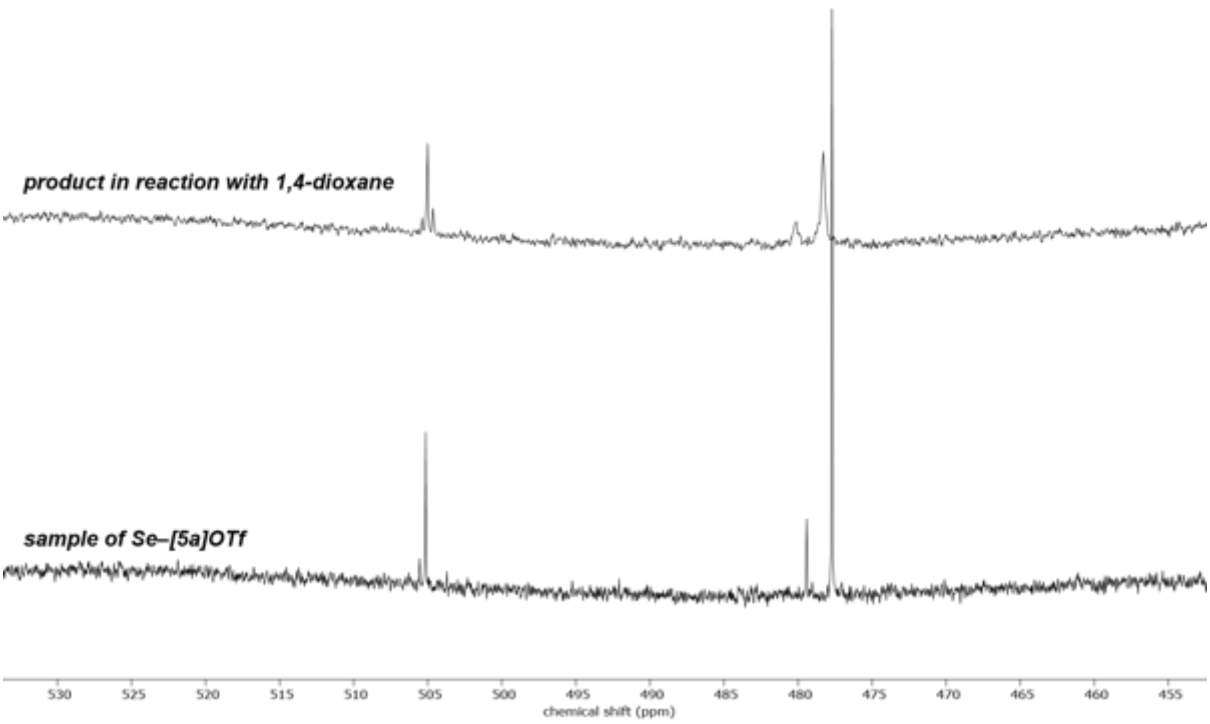

Figure S11. ^{77}Se NMR spectra of the crude reaction of dimerization of **[2a]SbF$_6$** after addition of 1,4-dioxane and compound **Se–[5a]OTf** in CD_3CN. **Top:** crude reaction spectrum after addition of 1,4-dioxane in CD_3CN. **Bottom:** original sample of **Se–[5a]OTf** in CD_3CN.

Reactivity with water

The reaction side-products of cationic selenurane **[2a]SbF6** with water were analyzed using ^{1}H NMR spectroscopy. The mixtures were concentrated under reduced pressure, dissolved in DMSO-d_6, transferred to the NMR tube, and CH_2Br_2 (7.1 µL, 0.10 mmol, 1.0 equiv.) was added to the NMR tube as an internal standard.

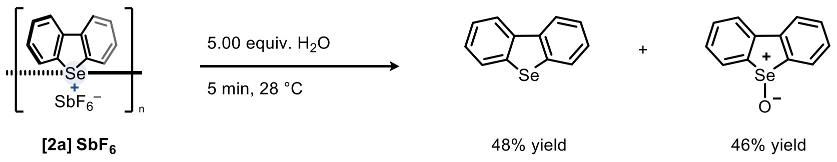

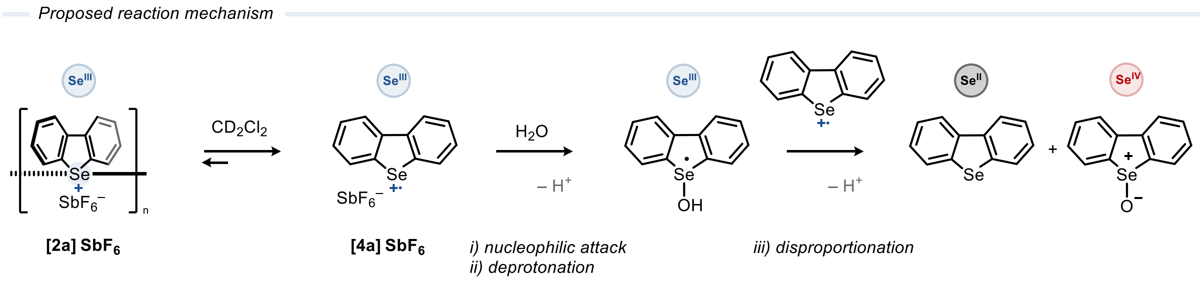

Dibenzo[*b*,*d*]selenophene (**1a** – 48% yield) and dibenzo[*b*,*d*]selenophene 5-oxide (46% yield) were identified as main products.

We propose the following mechanism for this transformation: *i)* nucleophilic attack of water at the Se(III) center; *ii)* deprotonation leads to the formation of a neutral Se(III) intermediate; *iii)* In the absence of external oxidants, a second equivalent of the Se(III) radical cation acts as the oxidant, effecting disproportionation. This process results in the formation of one equivalent of Se(IV) selenoxide and one equivalent of Se(II) selenide (**1a**).

OXIDATIONS WITH CATIONIC SELENURANES – STABILITY TESTS WITH TEMPO

Stability test of [2a]SbF₆ – oxidation of TEMPO to the corresponding oxoammonium salt

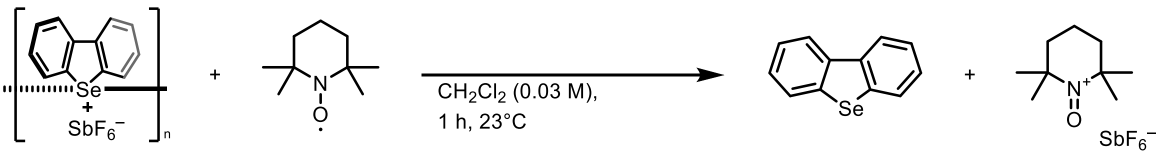

1a

Under an ambient atmosphere, to a 4-mL borosilicate vial equipped with a magnetic stir bar, selenurane **[2a]SbF₆** (14.0 mg, 0.030 mmol, 1.00 equiv.) and CH_2Cl_2 (0.90 mL, 0.03 M) were added. Then, 2,2,6,6-tetramethylpiperidine-1-oxyl (free radical) (4.70 mg, 0.03 mmol, 1.00 equiv.) was added portionwise to the stirring (200 rpm) mixture. The reaction mixture was stirred for 1 h. After 1 h, the mixture was concentrated by rotary evaporation under reduced pressure. $CDCl_3$ (0.50 mL) was added, resulting solution was transferred to the NMR tube, CH_2Br_2 (7.1 µL, 0.10 mmol, 3.3 equiv.) was added as internal standard, and the yield was determined by ^{1}H NMR spectroscopy by comparing the integration of the CH_2Br_2 signal at 4.93 ppm (s, 2H) to the signal of **1a** at 7.68 ppm (dt, 2H).

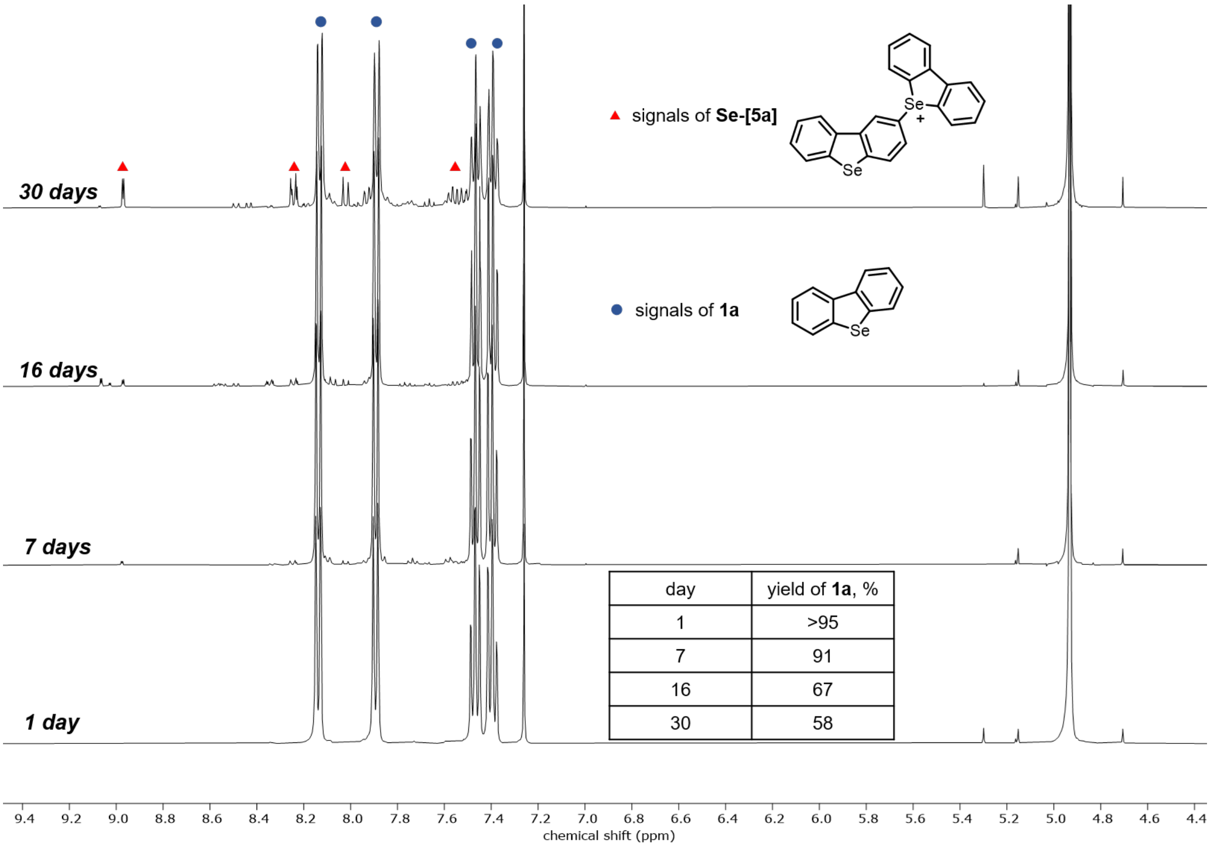

day	yield of **1a**, %
1	>95
7	91
16	67
30	58

Figure S12. ^{1}H NMR spectra (400 MHz, $CDCl_3$, 23 °C, δ) of the TEMPO oxidation reaction with **[2a]SbF₆**, performed after different days storing one batch of a cationic selenurane on the bench.

NMR experiment of benzylic alcohol oxidation with formed *in situ* oxoammonium salt

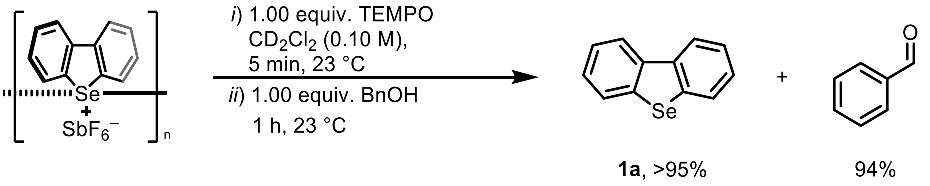

This experiment was conducted to demonstrate the generation of the oxoammonium salt.

Under an ambient atmosphere, to a NMR tube selenurane **[2a]SbF₆** (23.3 mg, 0.050 mmol, 1.00 equiv.) and CD_2Cl_2 (0.50 mL, 0.10 M) were added. The resulting solution was analyzed by ^{1}H NMR spectroscopy (Figure S13). Then, 2,2,6,6-tetramethylpiperidine-1-oxyl (free radical) (7.8 mg, 0.050 mmol, 1.0 equiv.) was added, and the NMR tube was carefully shaken until the blue color disappeared. In the ^{1}H NMR spectrum of the resulting mixture signals of the dibenzo[*b*,*d*]selenophene **1a** were identified. Next, benzylic alcohol (5.15 µL, 0.05 mmol, 1.00 equiv.) was added to the NMR tube, it was carefully shaken and left for 1 h. After 1 h, in the ^{1}H NMR spectrum of the resulting mixture signals of the dibenzo[*b*,*d*]selenophene **1a** and benzaldehyde were identified. Then, CH_2Br_2 (7.1 µL, 0.10 mmol, 2.0 equiv.) was added to the NMR tube and the yield of benzaldehyde was determined by comparison of integration of the CH_2Br_2 signal at 4.97 ppm (s, 2H) and the signal of benzaldehyde at 10.0 ppm (s, 1H). The oxidation procedure afforded benzaldehyde in 94% yield.

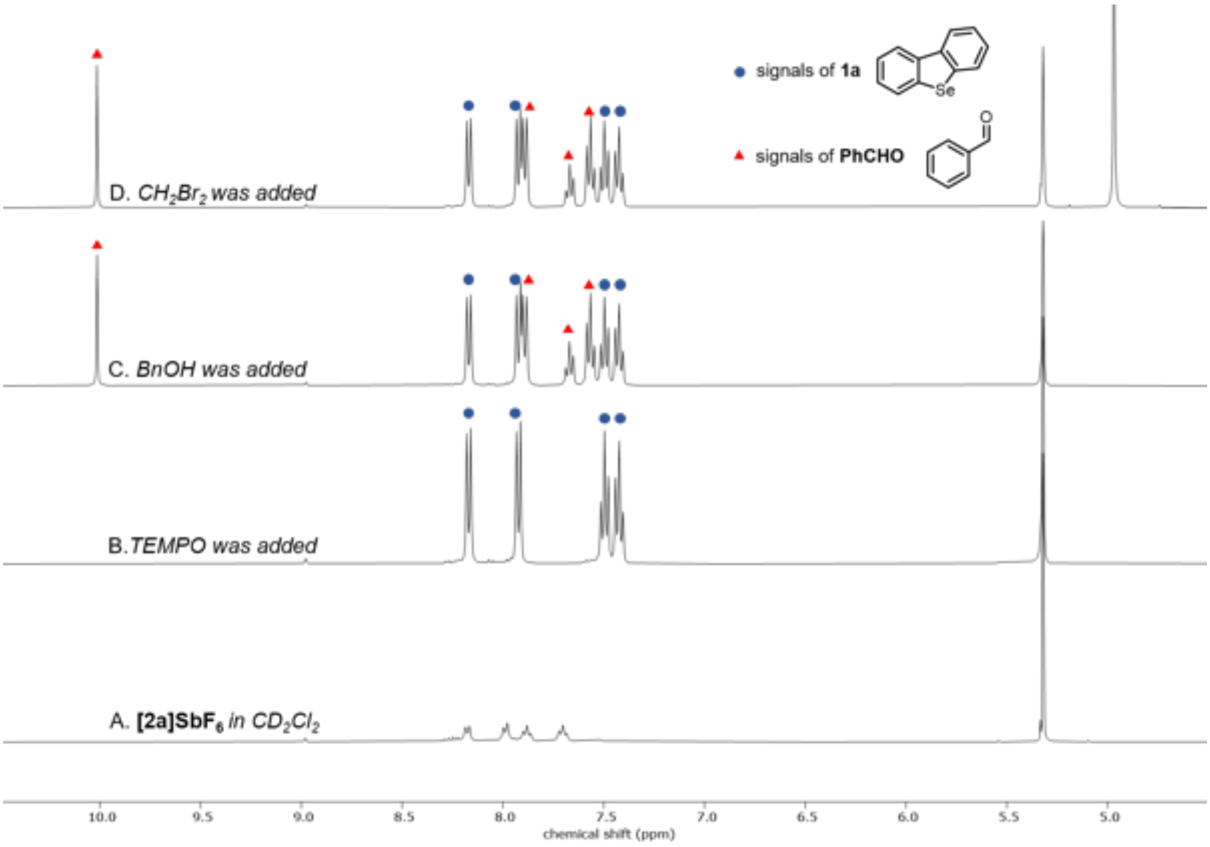

Figure S13.NMR experiment of benzylic alcohol oxidation with formed *in situ* oxoammonium salt.

Stability of [2c]SbF₆ – oxidation of TEMPO to the corresponding oxoammonium salt

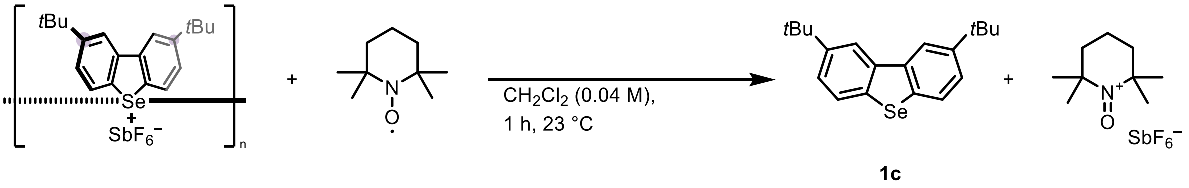

Under an ambient atmosphere, to a 4-mL borosilicate vial equipped with a magnetic stir bar, selenurane **[2c]SbF₆** (6.3 mg, 11 µmol, 1.0 equiv.) and CH_2Cl_2 (0.30 mL, 0.04 M) were added. Then, 2,2,6,6-tetramethylpiperidine-1-oxyl (free radical) (1.70 mg, 10.9 µmol, 1.00 equiv.) was added portionwise to the stirring (200 rpm) mixture. The reaction mixture was stirred for 1 h. After 1 h, the mixture was concentrated by rotary evaporation under reduced pressure. $CDCl_3$ (0.50 mL) was added, resulting solution was transferred to the NMR tube, CH_2Br_2 (1.5 µL, 21 µmol, 2.0 equiv.) was added as internal standard, and the yield was determined by ^{1}H NMR spectroscopy by comparing the integration of the CH_2Br_2 signal at 4.93 ppm (s, 2H) to the signal of **1a** at 8.13 ppm (d, 2H).

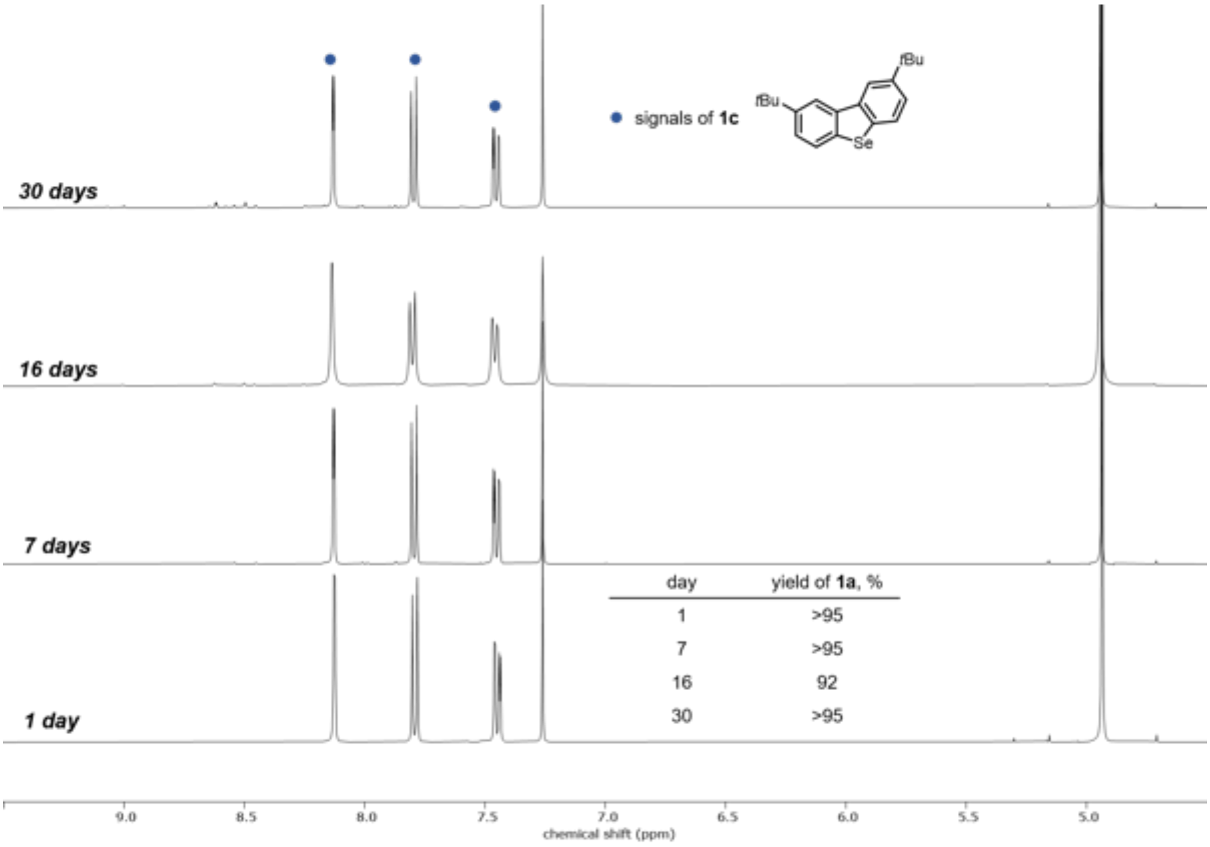

Figure S14. ^{1}H NMR spectra (400 MHz, $CDCl_3$, 23 ºC, δ) of the TEMPO oxidation reactions with **[2c]SbF₆**, performed after different numbers of days storing one batch of a cationic selenurane on the bench.

Oxidations with cationic selenuranes – Further examples

(*E*)-1,2-diphenyldiazene (7)

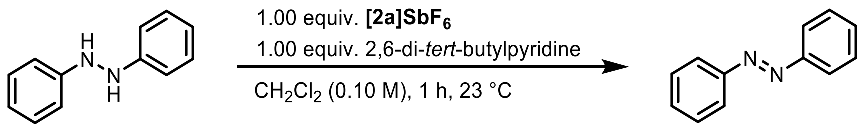

91% yield **7**

Under an ambient atmosphere, to a 20-mL borosilicate vial equipped with a magnetic stir bar selenurane **[2a]SbF$_6$** (467 mg, 0.500 mmol, 1.00 equiv.) and CH_2Cl_2 (5.00 mL, 0.10 M) were added. Then, 2,6-di-*tert*-butylpyridine (113 µL, 0.50 mmol, 1.00 equiv.) and 1,2-diphenylhydrazine (92.1 mg, 0.50 mmol, 1.00 equiv.) were added subsequently to a stirring (200 rpm) mixture. The reaction mixture was stirred at 23 °C for 1 h. After 1 h, the mixture was concentrated by rotary evaporation under reduced pressure. The residue was purified by flash column chromatography on silica gel using a gradient of eluent systems from pure heptane to EtOAc/heptane (1:20 (v/v)) to afford the desired product as red crystalline solid in 91% yield (83.0 mg, 0.455 mmol).

R*f* = 0.14 (heptane).

R*f* = 0.60 (EtOAc/heptane (1:20 (v/v))).

NMR Spectroscopy:

^{1}H NMR (400 MHz, $CDCl_3$, 23 ºC, δ): 7.98 – 7.91 (m, 2H), 7.58 – 7.47 (m, 3H).

^{13}C NMR (101 MHz, $CDCl_3$, 23 ºC, δ): 152.8, 131.1, 129.2, 123.00.

HRMS-EI(m/z) calc'd for $C_{12}H_{10}N$ $[M]^+$, 182.0844; found, 182.0842; deviation: – 1.1 ppm.

Fluorenone (9)

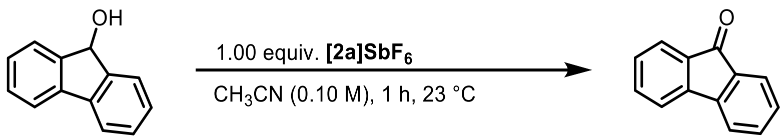

76% yield **9**

Under an ambient atmosphere, to a 20-mL borosilicate vial equipped with a magnetic stir bar selenurane **[2a]SbF$_6$** (233 mg, 0.50 mmol, 1.00 equiv.) and CH_3CN (5.00 mL, 0.10 M) were added. Then, 9-fluorenol (92.1 mg, 0.50 mmol, 1.00 equiv.) was added portionwise to a stirring (200 rpm) mixture. The reaction mixture was stirred at 23 °C for 1 h. After 1 h, the mixture was concentrated by rotary evaporation under reduced pressure. The residue was purified by flash column chromatography on silica gel using EtOAc/heptane (1:20 (v/v)) as eluent to afford the desired product as yellow crystalline solid in 76% yield (68.0 mg, 0.377 mmol).

R_f = 0.35 (EtOAc/heptane (1:20 (v/v))).

NMR Spectroscopy:

^{1}H NMR (500 MHz, $CDCl_3$, 23 °C, δ): 7.66 (d, J = 7.3 Hz, 2H), 7.55 – 7.44 (m, 4H), 7.33 – 7.25 (m, 2H).

^{13}C NMR (101 MHz, $CDCl_3$, 23 °C, δ): 194.1, 144.6, 134.8, 134.3, 129.2, 124.5, 120.4.

HRMS-EI(m/z) calc'd for $C_{13}H_8O$ $[M]^+$, 180.0575; found, 180.0578; deviation: +1.7 ppm.

Additional substrates

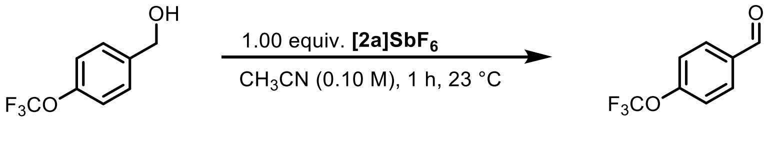

>98% yield **S10**

Under an ambient atmosphere, to a 4-mL borosilicate vial equipped with a magnetic stir bar, selenurane **[2a]SbF₆** (23.3 mg, 0.05 mmol, 1.00 equiv.) and CD_3CN (0.50 mL, 0.10 M) were added. Then, 4-(trifluoromethoxy)benzyl alcohol (7.2 µL, 0.05 mmol, 1.00 equiv.) was added using the 10.0-µL syringe to the stirring (200 rpm) mixture. The reaction mixture was stirred for 1 h. After 1 h, the mixture was transferred to the NMR tube, CH_2Br_2 (3.6 µL, 0.05 mmol, 1.0 equiv.) was added as internal standard, and the yield was determined by ^{1}H NMR spectroscopy by comparing the integration of the CH_2Br_2 signal at 5.08 ppm (s, 2H) to the signal of **S10** at 9.99 ppm (s, 1H) (Yield = 1.00/1.00 = 100%).

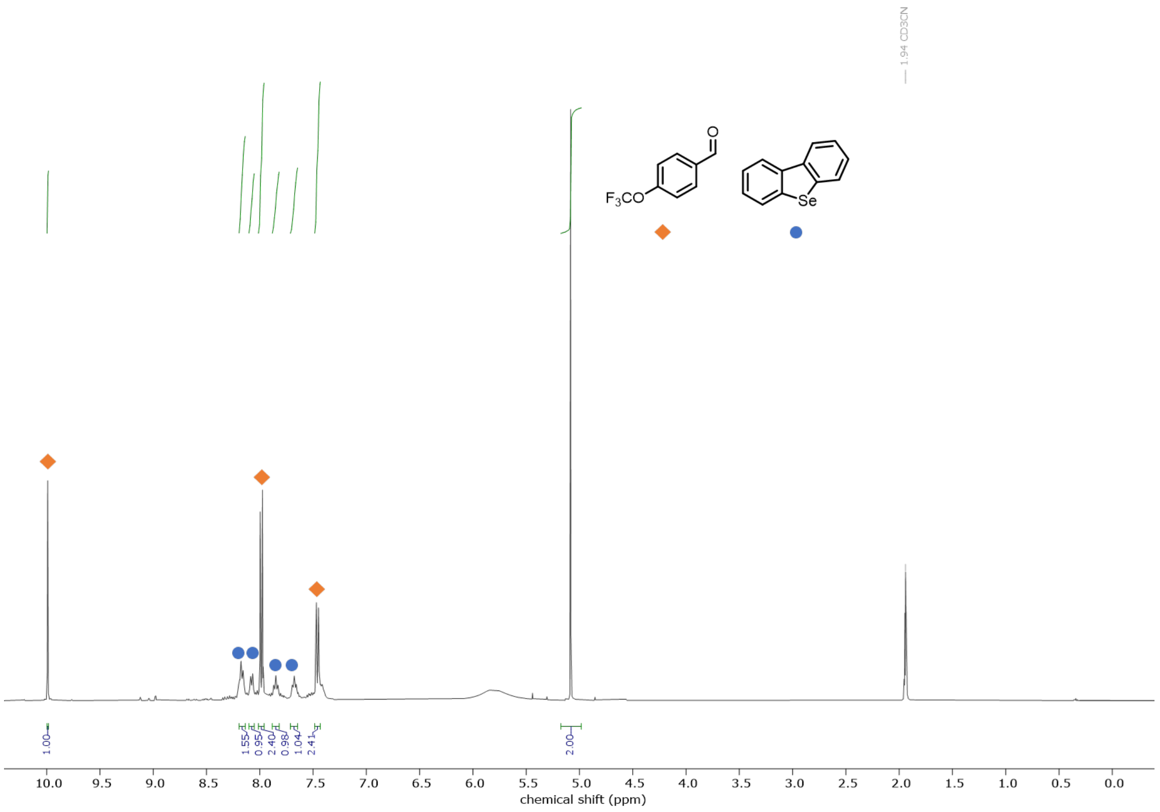

Figure S15.^{1}H NMR spectrum at 400 MHz recorded in CD_3CN solution of the reaction mixture for the formation of the aldehyde **S10**. The yield was determined by ^{1}H NMR spectroscopy at 400 MHz and 298 K of the crude reaction mixture in 0.5 mL of CD_3CN, and using CH_2Br_2 as internal standard (3.6 µL, 9.0 mg, 0.05 mmol, 1.0 equiv.). The integration of the CH_2Br_2 signal at 5.08 ppm (s, 2H, 1.0 equiv.) was compared to the signal of the product at 9.99 ppm (s, 1H, 1.00 equiv.). Orange diamonds = aldehyde **S10**, blue balls = dibenzoselenophene **1a**.

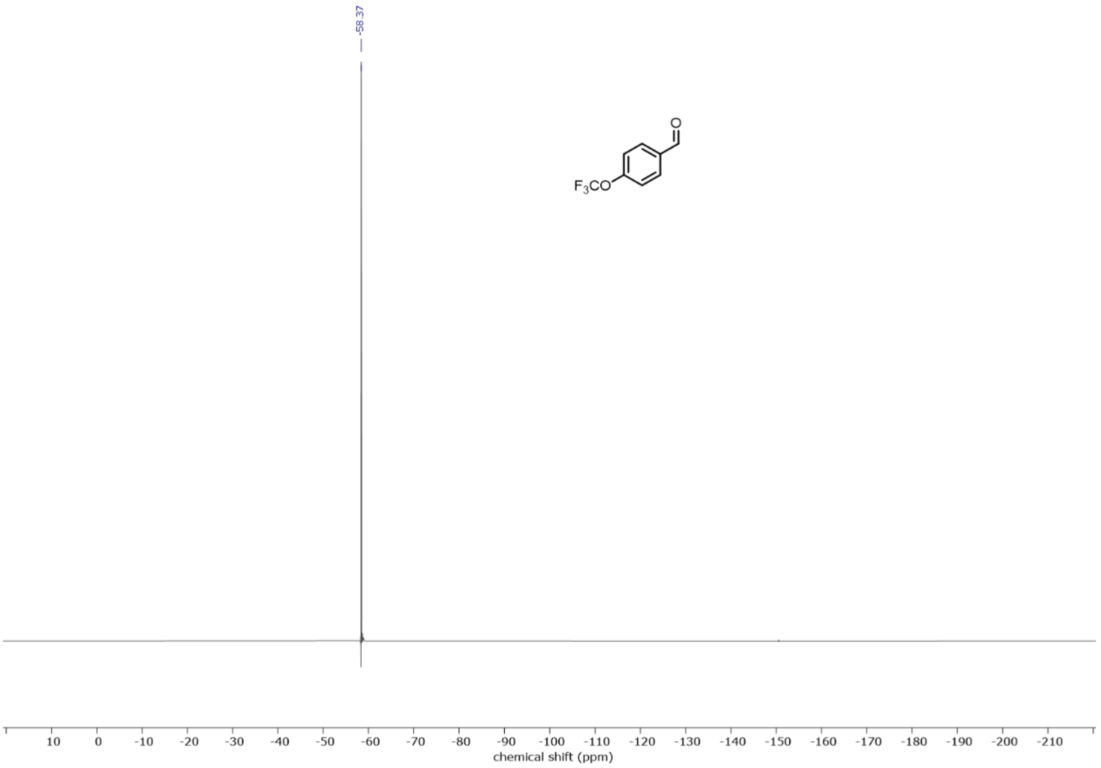

Figure S16.^{19}F NMR spectrum at 377 MHz recorded in $CDCl_3$ solution of the reaction mixture for the formation of the aldehyde **S10**.

PREPARATION OF SELENONIUM SALTS FROM CATIONIC SELENURANES

Reaction stoichiometry

Example of yield calculation for group transfer reactions:

1.0 mmol *of cationic selenurane will yield maximum* ***0.5 mmol*** *of dibenzoselenophene* ***1a*** *(Se(II)) and* ***0.5 mmol*** *of selenonium salt (Se(IV)).*

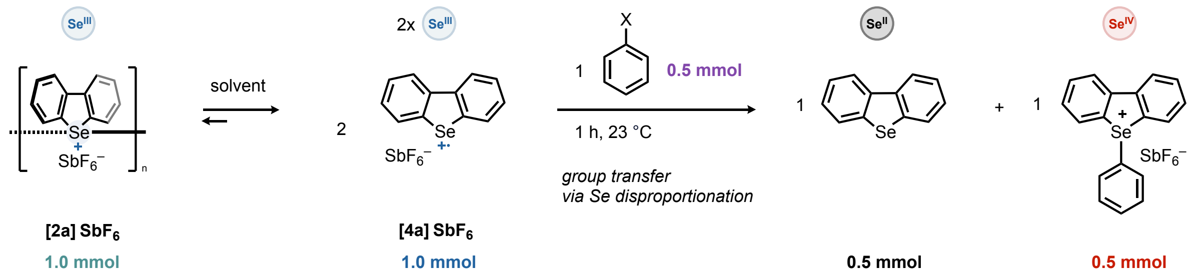

Example of a reaction crude

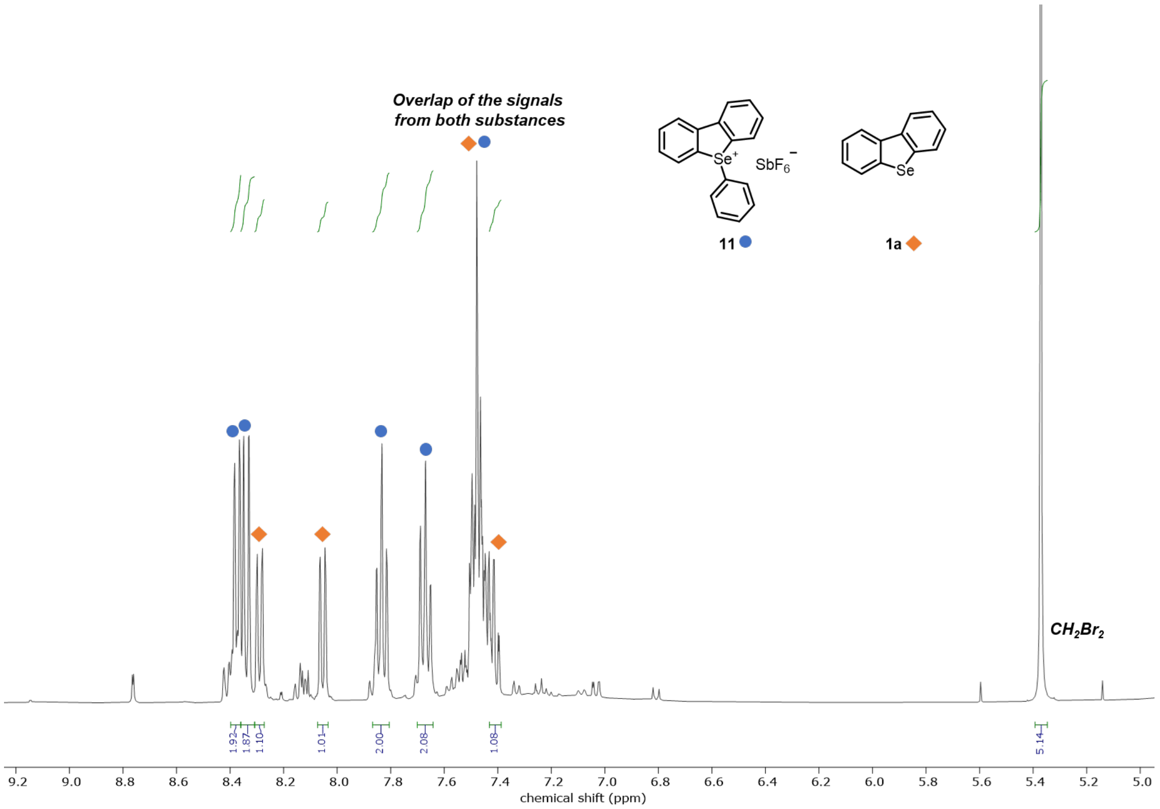

Figure S17. ^{1}H NMR spectrum at 400 MHz recorded in DMSO-d_6 solution of the reaction mixture for the formation of the selenonium salt **11** and selenide **1a** in group transfer reaction of cationic selenurane **[2a]SbF₆** and trimethyl(phenylsilane). Orange diamonds = dibenzoselenophene **1a**, blue balls = selenonium salt **11**.

5-(trifluoromethyl) -dibenzoselenophenium hexafluoroantimonate (10)

*General optimization procedure for the preparation of **10***

Under an ambient atmosphere, to a 4-mL borosilicate vial equipped with a magnetic stir bar, selenurane **[2a]SbF₆** (46.7 mg, 1.00 mmol) and solvent (0.50 mL, 0.10 M) were added. Then, sodium trifluoromethanesulfinate was added portionwise to the stirring (200 rpm) mixture. The reaction mixture was stirred at 23 °C. After the stirring for the reaction time, the mixture was concentrated by rotary evaporation under reduced pressure. The residue was fully solubilized in DMSO-d_6 (0.50 mL), transferred to the NMR tube, trifluorotoluene (6.0 µL, 0.50 mmol = theoretical maximum yield) was added as internal standard, and the yield was determined by ^{19}F NMR spectroscopy using trifluorotoluene as internal standard by comparing the integration of the trifluorotoluene signal at –61.3 ppm (s, 3F) to the signal of **10** at –47.3 ppm (s, 3F).

Optimization table

Table S1. Optimization of the reaction of **[2a]SbF$_6$** with sodium trifluoromethanesulfinate.

Entry	equiv. F_3CSO_2Na	solvent	reaction time, h	Yield **10*** (%)
1	1.00	CH_2Cl_2	1	22
2	1.00	CH_3CN	1	n.r.
3	1.00	HFIP	1	37
**4**	**1.50**	**HFIP**	**16**	**53**
5	2.00	HFIP	16	56
6	5.00	HFIP	16	64

** The yield was determined by ^{19}F NMR spectroscopy using α,α,α-trifluorotoluene as internal standard.*

Procedure and characterization

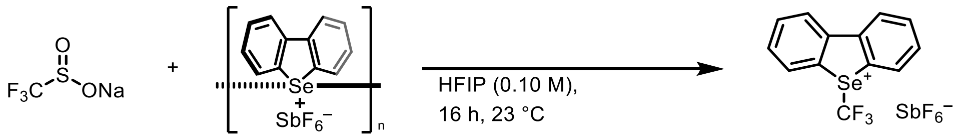

1.50 equiv. **54% yield** **10**

Under an ambient atmosphere, to a 20-mL borosilicate vial equipped with a magnetic stir bar, selenurane **[2a]SbF$_6$** (467 mg, 1.00 mmol) and HFIP (5.00 mL, 0.10 M) were added. Then, sodium trifluoromethanesulfinate (123 mg, 0.75 mmol, 1.50 equiv.) was added portionwise to the stirring (200 rpm) mixture. The reaction mixture was stirred at 23 °C for 16 h. After 16 h, the mixture was concentrated by rotary evaporation under reduced pressure. The residue was purified by washing with Et_2O (3×10 mL) and water (3×10 mL). The resulting solid was dried under high vacuo to afford the desired product as pale green solid in 54% yield (145 mg, 0.271 mmol).

NMR Spectroscopy:

^{1}H NMR (400 MHz, CD_3CN, 23 ºC, δ): δ 8.28 – 8.26 (m, 4H), 7.98 (td, *J* = 7.6, 1.1 Hz, 2H), 7.81 –

7.78 (m, 2H).

^{13}C NMR (151 MHz, CD_3CN, 23 ºC, δ): 143.9, 136.3, 133.0, 131.6, 129.8, 126.6, 121.9 (q, *J* = 352.6 Hz).

^{19}F NMR (659 MHz, CD_3CN, 23 ºC, δ): –46.71, –116.69 to –131.41 (m, 6H).

^{77}Se NMR (151 MHz, CD_3CN, 23 ºC, δ): 595.0 (q, *J* = 15.0).

HRMS-ESI(m/z) calc'd for $C_{13}H_8F_3Se$ $[M]^+$, 300.9738; found, 300.9740; deviation: +0.7 ppm.

HRMS-ESI(m/z) calc'd for SbF_6 $[M]^-$, 234.8948; found, 234.8954; deviation: +2.6 ppm.

5-(phenyl) -dibenzoselenophenium hexafluoroantimonate (11)

*General optimization procedure for the preparation of **11** from aryl stannanes*

Under an ambient atmosphere, to a 4-mL borosilicate vial equipped with a magnetic stir bar, selenurane **[2a]SbF₆** (46.7 mg, 0.10 mmol) and solvent (0.50 mL, 0.10 M) were added. Then, tributylphenyltin was added using the 100-µL syringe to the stirring (200 rpm) mixture. The reaction mixture was stirred at corresponding temperature for 1 h. After 1 h, the mixture was concentrated by rotary evaporation under reduced pressure. The residue was fully solubilized in DMSO-d_6 (0.50 mL), transferred to the NMR tube, CH_2Br_2 (7.1 µL, 0.10 mmol = 200% theoretical yield) was added as internal standard, and the yield was determined by ^{1}H NMR spectroscopy by comparing the integration of the CH_2Br_2 signal at 5.39 ppm (s, 2H) to the signal of **11** at 7.68 ppm (dt, 2H).

Optimization table

Table S2. Optimization of the reaction of **[2a]SbF₆** with tributylphenyltin.

Entry	equiv. $PhSnBu_3$	solvent	temperature, °C	Yield **11*** (%)
1	1.00	CH_2Cl_2	23	52
2	1.00	CH_2Cl_2	0	34
**3**	**1.50**	**CH_2Cl_2**	**23**	**94**
4	1.50	CH_3CN	23	75

* *The yield was determined by ^{1}H NMR spectroscopy using CH_2Br_2 as internal standard.*

Procedure

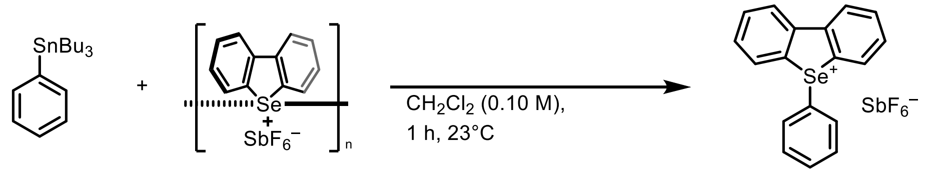

1.50 equiv. **78% yield** **11**

Under an ambient atmosphere, to a 20-mL borosilicate vial equipped with a magnetic stir bar, selenurane **[2a]SbF₆** (467 mg, 1.00 mmol) and CH_2Cl_2 (5.00 mL, 0.10 M) were added. Then, tributylphenyltin (245 µL, 0.75 mmol, 1.50 equiv.) was added using the 250-µL syringe to the stirring

(200 rpm) mixture. The reaction mixture was stirred at 23 °C for 1 h. After 1 h, the mixture was concentrated by rotary evaporation under reduced pressure. The residue was washed with Et_2O (3×10 mL) and purified for further characterization by flash column chromatography on silica gel using a gradient of eluent systems from MeOH/$CHCl_3$ (1:20 (v/v)) to MeOH/$CHCl_3$ (1:10 (v/v)) to afford the desired product as pale yellow solid in 78% yield (213 mg, 0.392 mmol).

*General optimization procedure for the preparation of **11** from aryl trifluoroborate salts*

Under an ambient atmosphere, to a 4-mL borosilicate vial equipped with a magnetic stir bar, selenurane **[2a]SbF₆** (46.7 mg, 0.10 mmol) and solvent (0.50 mL, 0.10 M) were added. Then, potassium trifluoro(phenyl)borate was added portionwise to the stirring (200 rpm) mixture. The reaction mixture was stirred at corresponding temperature for 1 h. After 1 h, the mixture was concentrated by rotary evaporation under reduced pressure. The residue was fully solubilized in DMSO-d_6 (0.50 mL), transferred to the NMR tube, CH_2Br_2 (7.1 µL, 0.10 mmol = 200% theoretical yield) was added as internal standard, and the yield was determined by ^{1}H NMR spectroscopy by comparing the integration of the CH_2Br_2 signal at 5.39 ppm (s, 2H) to the signal of **11** at 7.68 ppm (dt, 2H).

Optimization table

Table S3. Optimization of the reaction of **[2a]SbF_6** with potassium trifluoro(phenyl)borate

Entry	equiv. $PhBF_3K$	solvent	temperature, °C	Yield **11*** (%)
1	1.00	CH_2Cl_2	23	50
2	1.00	CH_2Cl_2	0	52
3	1.00	CH_3CN	23	46
4	1.00	HFIP	23	80**
5	1.50	CH_2Cl_2	23	70
**6**	**2.00**	**CH_2Cl_2**	**23**	**87**

** The yield was determined by ^{1}H NMR spectroscopy using CH_2Br_2 as internal standard. ** 5.0 µL of TMSCl were added*

Procedure

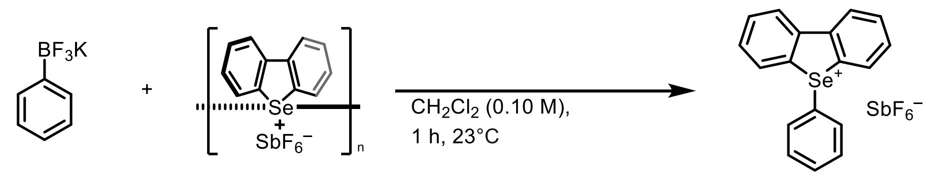

2.00 equiv. **80% yield** **11**

Under an ambient atmosphere, to a 20-mL borosilicate vial equipped with a magnetic stir bar, selenurane **[2a]SbF_6** (467 mg, 1.00 mmol) and CH_2Cl_2 (5.00 mL, 0.10 M) were added. Then, potassium trifluoro(phenyl)borate (190 mg, 1.00 mmol, 2.00 equiv.) was added portionwise to the stirring (200 rpm) mixture. The reaction mixture was stirred at 23 °C for 1 h. After 1 h, the mixture was concentrated by rotary evaporation under reduced pressure. The residue was washed with Et_2O (3×10 mL) and purified for further characterization by flash column chromatography on silica gel using a gradient of eluent

systems from MeOH/$CHCl_3$ (1:20 (v/v)) to MeOH/$CHCl_3$ (1:10 (v/v)) to afford the desired product as pale yellow solid in 80% yield (217 mg, 0.399 mmol).

*General optimization procedure for the preparation of **11** from aryl silanes*

Under an ambient atmosphere, to a 4-mL borosilicate vial equipped with a magnetic stir bar, selenurane **[2a]SbF₆** (46.7 mg, 0.10 mmol) and solvent (0.50 mL, 0.10 M) were added. Then, trimethyl(phenyl)silane was added using the 100-μL syringe to the stirring (200 rpm) mixture. The reaction mixture was stirred for 1 h. After 1 h, the mixture was concentrated by rotary evaporation under reduced pressure. The residue was fully solubilized in DMSO-d_6 (0.50 mL), transferred to the NMR tube, CH_2Br_2 (7.1 μL, 0.10 mmol = 200% theoretical yield) was added as internal standard, and the yield was determined by ^{1}H NMR spectroscopy by comparing the integration of the CH_2Br_2 signal at 5.39 ppm (s, 2H) to the signal of **11** at 7.68 ppm (dt, 2H).

Optimization table

Table S4. Optimization of the reaction of **[2a]SbF$_6$** with trimethyl(phenyl)silane

Entry	equiv. PhTMS	solvent	additive	Yield **11*** (%)
1	1.00	CH_2Cl_2	-	40
2	1.00	CH_3CN	-	52
3	1.00	HFIP	-	50
4	2.00	CH_3CN	-	97
5	2.00	HFIP	-	80
6	2.00	CH_3CN	TMSCl (5.0 μL)	85
7	2.00	HFIP	TMSCl (5.0 μL)	58
**8**	**1.50**	**CH_3CN**	**-**	**81**

** The yield was determined by ^{1}H NMR spectroscopy using CH_2Br_2 as internal standard.*

Procedure

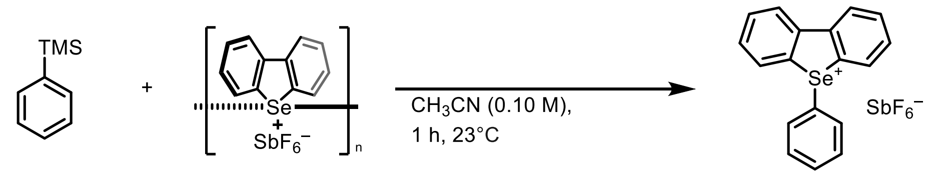

1.50 equiv. **80% yield** **11**

Under an ambient atmosphere, to a 20-mL borosilicate vial equipped with a magnetic stir bar, selenurane **[2a]SbF₆** (467 mg, 1.00 mmol) and CH_3CN (5.00 mL, 0.10 M) were added. Then, trimethyl(phenyl)silane (130 μL, 0.75 mmol, 1.50 equiv.) was added using the 250-μL syringe to the stirring (200 rpm) mixture. The reaction mixture was stirred at 23 °C for 1 h. After 1 h, the mixture was concentrated by rotary evaporation under reduced pressure. The residue was washed with Et_2O (3×10 mL) and purified for further characterization by flash column chromatography on silica gel using a

gradient of eluent systems from MeOH/$CHCl_3$ (1:20 (v/v)) to MeOH/$CHCl_3$ (1:10 (v/v)) to afford the desired product as gray solid in 94% yield (256 mg, 0.471 mmol).

R*f* = 0.27 (MeOH/$CHCl_3$ (1:10 (v/v))).

NMR Spectroscopy:

^{1}**H NMR** (600 MHz, DMSO-d_6, 23 °C, δ): 8.41 (dd, *J* = 7.9, 0.9 Hz, 2H), 8.36 (dd, *J* = 8.0, 0.9 Hz, 2H), 7.86 (dt, *J* = 8.0, 1.0 Hz, 2H), 7.69 (dt, *J* = 8.0, 1.0 Hz, 2H), 7.56 – 7.45 (m, 5H).

^{13}**C NMR** (151 MHz, DMSO-d_6, 23 °C, δ): 141.1, 136.8, 133.2, 132.7, 131.9, 130.8, 130.6, 129.7, 129.0, 124.8.

^{19}**F NMR** (376 MHz, DMSO-d_6, 23 °C, δ): –106.53 to –132.52 (m).

^{77}**Se NMR** (115 MHz, DMSO-d_6, 23 °C, δ): 518.7.

HRMS-ESI(m/z) calc'd for $C_{18}H_{13}Se$ $[M]^+$, 309.0177; found, 309.0182; deviation: +2.7 ppm.

HRMS-ESI(m/z) calc'd for SbF_6 $[M]^-$, 234.8948; found, 234.8956; deviation: +3.4 ppm.

To demonstrate the synthetic utility of different cationic selenuranes, compounds ***[2b]SbF₆, [2c]SbF₆,*** *and* ***[2d]SbF₆*** *were also tested in the group transfer reaction with potassium trifluoro(phenyl)borate in the optimized conditions.*

3,7-Di-*tert*-butyl-5-phenyl-5*H*-dibenzo[*b*,*d*]selenophen-5-ium hexafluoroantimonate (11b)

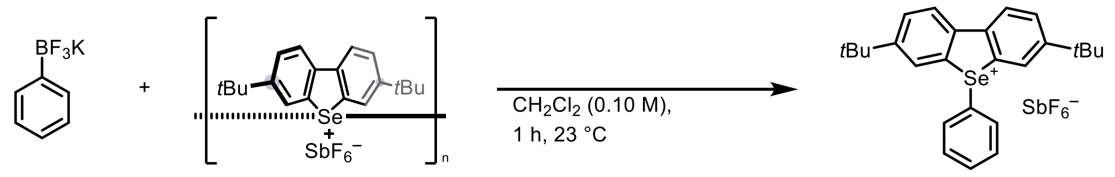

2.00 equiv. **98% yield** **11b**

Under an ambient atmosphere, to a 4-mL borosilicate vial equipped with a magnetic stir bar, selenurane **[2b]SbF₆** (232 mg, 0.40 mmol) and CH_2Cl_2 (2.00 mL, 0.10 M) were added. Then, potassium trifluoro(phenyl)borate (76.0 mg, 0.40 mmol, 2.00 equiv.) was added portionwise to the stirring (200 rpm) mixture. The reaction mixture was stirred at 23 °C for 1 h. After 1 h, the mixture was concentrated by rotary evaporation under reduced pressure. The residue was purified by flash column chromatography on silica gel using a gradient of eluent systems from $CHCl_3$ to MeOH/$CHCl_3$ (1:20 (v/v)) to afford the desired product as brown crystalline solid in 98% yield (128 mg, 0.195 mmol).

R*f* = 0.25 (MeOH/$CHCl_3$ (1:20 (v/v))).

NMR Spectroscopy:

^{1}**H NMR** (500 MHz, $CDCl_3$, 23 °C, δ): 8.43 (d, *J* = 1.8 Hz, 2H), 8.27 (d, *J* = 7.5 Hz, 2H), 7.87 (dd, *J* = 7.4, 1.8 Hz, 2H), 7.52–7.59 (m, 5H), 1.33 (s, 18H).

^{13}**C NMR** (101 MHz, DMSO-d_6, 23 °C, δ): 153.7, 138.5, 136.8, 133.3, 131.9, 130.7, 129.9, 128.9, 126.4, 124.2, 35.3, 30.9.

^{19}**F NMR** (376 MHz, DMSO-d_6, 23 °C, δ): –106.61 to –132.49 (m).

^{77}**Se NMR** (115 MHz, DMSO-d_6, 23 °C, δ): 517.3.

HRMS-ESI(m/z) calc'd for $C_{26}H_{29}Se$ $[M]^+$, 421.1429; found, 421.1426; deviation: –0.7 ppm.

HRMS-ESI(m/z) calc'd for SbF_6 $[M]^-$, 234.8948; found, 234.8949; deviation: +0.4 ppm

2,8-Di-*tert*-butyl-5-phenyl-5*H*-dibenzo[*b*,*d*]selenophen-5-ium hexafluoroantimonate (11c)

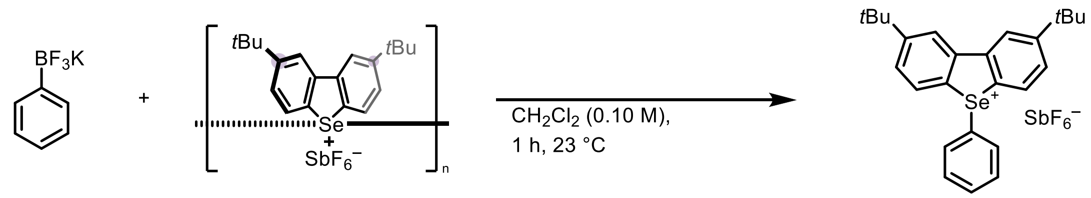

2.00 equiv. **98% yield** **11c**

Under an ambient atmosphere, to a 4-mL borosilicate vial equipped with a magnetic stir bar, selenurane **[2c]SbF$_6$** (232 mg, 0.40 mmol) and CH_2Cl_2 (2.00 mL, 0.10 M) were added. Then, potassium trifluoro(phenyl)borate (76.0 mg, 0.40 mmol, 2.00 equiv.) was added portionwise to the stirring (200 rpm) mixture. The reaction mixture was stirred at 23 °C for 1 h. After 1 h, the mixture was concentrated by rotary evaporation under reduced pressure. The residue was purified by flash column chromatography on silica gel using a gradient of eluent systems from $CHCl_3$ to MeOH/$CHCl_3$ (1:20 (v/v)) to afford the desired product as green crystalline solid in 98% yield (129 mg, 0.197 mmol).

R*f* = 0.32 (MeOH/$CHCl_3$ (1:20 (v/v))).

NMR Spectroscopy:

^{1}**H NMR** (600 MHz, $CDCl_3$, 23 °C, δ): 8.06 (d, *J* = 1.9 Hz, 2H), 7.98 (d, *J* = 8.5 Hz, 2H), 7.66 (dd, *J* = 8.5, 2.0 Hz, 2H), 7.58 – 7.54 (m, 1H), 7.52 – 7.48 (m, 2H), 7.48 – 7.44 (m, 2H), 1.45 (s, 18H).

^{13}**C NMR** (151 MHz, $CDCl_3$, 23 °C, δ): 158.3, 141.3, 133.7, 131.7, 131.3, 129.8, 129.8, 129.8, 129.6, 121.5, 35.8, 31.3.

^{19}**F NMR** (376 MHz, DMSO-d_6, 23 °C, δ): –112.13 to –126.96 (m).

^{77}**Se NMR** (115 MHz, $CDCl_3$, 23 °C, δ): –490.1.

HRMS-ESI(m/z) calc'd for $C_{26}H_{29}Se$ $[M]^+$, 421.1429; found, 421.1426; deviation: –0.2 ppm.

HRMS-ESI(m/z) calc'd for SbF_6 $[M]^-$, 234.8948; found, 234.8949; deviation: +0.4 ppm

Octamethyl-octahydro dinaphthoselenophen-5-ium hexafluoroantimonate 11d

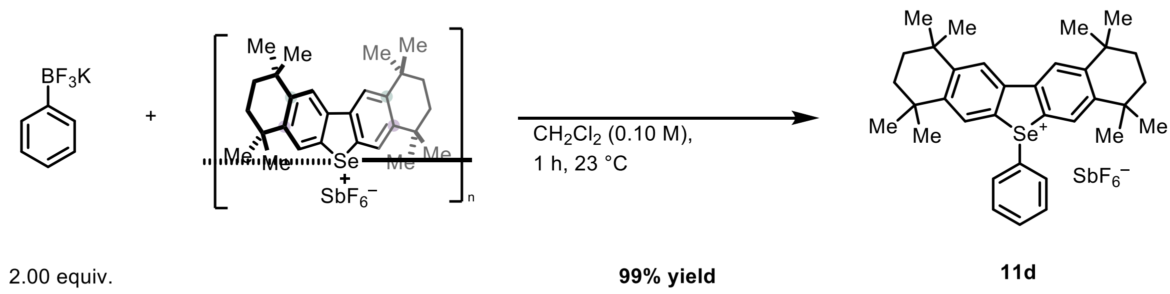

Under an ambient atmosphere, to a 4-mL borosilicate vial equipped with a magnetic stir bar, selenurane **[2d]SbF6** (275 mg, 0.40 mmol) and CH_2Cl_2 (2.00 mL, 0.10 M) were added. Then, potassium trifluoro(phenyl)borate (76.0 mg, 0.40 mmol, 2.00 equiv.) was added portionwise to the stirring (200 rpm) mixture. The reaction mixture was stirred at 23 °C for 1 h. After 1 h, the mixture was concentrated by rotary evaporation under reduced pressure. The residue was purified by flash column chromatography on silica gel using a gradient of eluent systems from $CHCl_3$ to MeOH/$CHCl_3$ (1:20 (v/v)) to afford the desired product as white solid in 99% yield (152 mg, 0.199 mmol).

$\mathbf{R}_f$ = 0.38 (MeOH/$CHCl_3$ (1:20 (v/v))).

NMR Spectroscopy:

^{1}**H NMR** (600 MHz, $CDCl_3$, 23 ºC, δ): 7.95 (s, 2H), 7.89 (s, 2H), 7.59 – 7.55 (m, 1H), 7.55 – 7.50 (m, 2H), 7.50 – 7.46 (m, 2H), 1.78 – 1.71 (m, 8H), 1.43 (d, J = 8.1 Hz, 12H), 1.35 (s, 6H), 1.24 (s, 6H).

^{13}**C NMR** (151 MHz, $CDCl_3$, 23 ºC, δ): 151.9, 150.4, 138.4, 133.5, 131.6, 131.4, 130.53 129.8, 128.4, 122.4, 35.6, 35.4, 34.7, 34.5, 32.0, 32.0, 31.8, 31.6.

^{19}**F NMR** (377 MHz, CD_3CN, 23 ºC, δ): –110.94 to –136.61 (m).

^{77}**Se NMR** (115 MHz, $CDCl_3$, 23 ºC, δ): –490.9.

HRMS-ESI(m/z) calc'd for $C_{34}H_{41}Se$ $[M]^+$, 529.2369; found, 529.2363; deviation: –1.1 ppm.

HRMS-ESI(m/z) calc'd for SbF_6 $[M]^-$, 234.8948; found, 234.8949; deviation: +0.4 ppm

Preparation of selenonium salts from cationic selenuranes – Stability tests

i) O_2 sensitivity experiments

Solutions of **[2a–d]SbF₆** in CH_2Cl_2 were exposed to an excess of O_2 by bubbling for 5 minutes with O_2. Potassium phenyltrifluoroborate was then added, and the yields of the resulting selenonium salts (**11a–d**) were compared with those from parallel reactions performed under standard ambient conditions (i.e., without O_2 bubbling but also without exclusion of air or moisture).

In all cases, comparable yields were obtained, indicating that O_2 does not significantly impact the performance of cationic selenuranes under synthetic conditions.

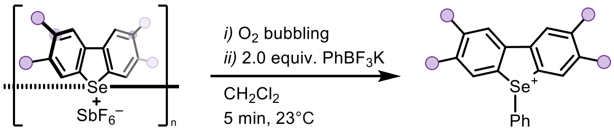

[2a-d] SbF$_6$ → **11a-d**

	**yield** without O_2 bubbling	**yield** with O_2 bubbling
[2a] SbF$_6$	80	90
[2b] SbF$_6$	98	95
[2c] SbF$_6$	98	83
[2d] SbF$_6$	99	92

ii) Moisture sensitivity experiments

We performed elemental analysis studies comparing **[2a]SbF₆** and **[2c]SbF₆** after 2 weeks of storage under ambient conditions with no special precautions (i.e., open Eppendorf tubes).

Sample measurement: After 17 days, two sample weights were placed in the autosampler at the same time and the first measurement was taken immediately. The following measurement started 7 minutes later, and the sample was stored under a dry helium stream during this time. A decrease in the oxygen value indicates that the water is being released during the helium purge, so that a reduced O-content (from absorbed moisture) is obtained in the second measurement.

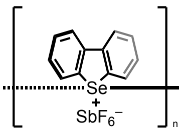

[2a] SbF$_6$

	**w% O**
0 days	<0.05
17 days	5.75

1.74 equiv. of water

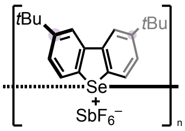

[2c] SbF$_6$

	**w% O**
0 days	<0.05
17 days	3.77

1.41 equiv. of water

The oxygen mass content indicated that **[2c]SbF₆**, bearing two hydrophobic *tert*-butyl groups, accumulated less water than **[2a]SbF₆**. These results confirm that the salts are hygroscopic and that exposure to atmospheric moisture affects their composition over time.

Preparation of selenonium salts from cationic selenuranes – Complex molecules

Bpin fenofibrate derivative S11

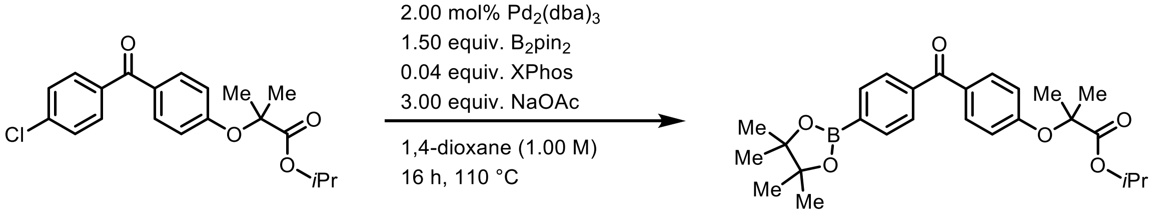

95% yield **S11**

To an oven-dried 25-mL Schlenk tube equipped with a magnetic stirring bar, fenofibrate (1.45 g, 4.00 mmol, 1.00 equiv.), tris(dibenzylideneacetone)dipalladium(0) (73.6 mg, 0.08 mmol, 2.00 mol%), bis(pinacolato)diboron (1.52 g, 6 mmol, 1.50 equiv.), XPhos (76.3 mg, 0.16 mmol, 0.04 equiv.) and sodium acetate (984 mg, 12.0 mmol, 3.00 equiv.) were added. The tube was sealed with a septum, connected via a Tygon® tubing to a Schlenk line, and evacuated and purged with argon three times. 1,4-Dioxane (4.00 mL, 1.00 M) was added, the septum was equipped with an Ar-filled balloon, and the reaction mixture was stirred at 110 °C for 16 h using an oil bath. After 16 h, the flask was removed from the oil bath and allowed to cool to 23 °C (ca. 30 minutes). Then, water (10 mL) was added, the resulting mixture was transferred to a 250-mL separation funnel. Organic phase was separated and aqueous phase was extracted with EtOAc (3×20 mL). The combined organic phase was dried over Na_2SO_4, and filtered by gravity using an 8-cm diameter funnel containing a fluted filter paper. Then, the resulting mixture was concentrated by rotary evaporation under reduced pressure. The residue was purified by flash column chromatography on silica gel using a gradient of eluent systems from EtOAc/heptane (1:5 (v/v)) to EtOAc/heptane (1:2 (v/v)) to afford the desired product as a dark oil in 95% yield (1.71 g, 3.25 mmol).

R_f = 0.53 (EtOAc/heptane (1:2 (v/v))).

NMR Spectroscopy:

^{1}H NMR (600 MHz, $CDCl_3$, 23 °C, δ): 7.90 (d, *J* = 8.2 Hz, 2H), 7.77 – 7.73 (m, 2H), 7.71 (d, *J* = 8.2 Hz, 2H), 6.88 – 6.81 (m, 2H), 5.08 (hept, *J* = 6.3 Hz, 1H), 1.65 (s, 6H), 1.36 (s, 12H), 1.20 (d, *J* = 6.3 Hz, 6H).

^{13}C NMR (151 MHz, $CDCl_3$, 23 °C, δ): 195.8, 173.3, 159.8, 140.5, 134.6, 132.2, 130.7, 128.8, 117.3, 84.3, 79.5, 69.4, 25.5, 25.0, 21.6.

^{11}B NMR (193 MHz, DMSO-d_6, 23 °C, δ): 30.59.

HRMS-ESI(m/z) calc'd for $C_{26}H_{33}BO_6Na$ $[M+Na]^+$, 475.2264; found, 475.2264; deviation: 0.0 ppm.

BF_3K fenofibrate derivative 12

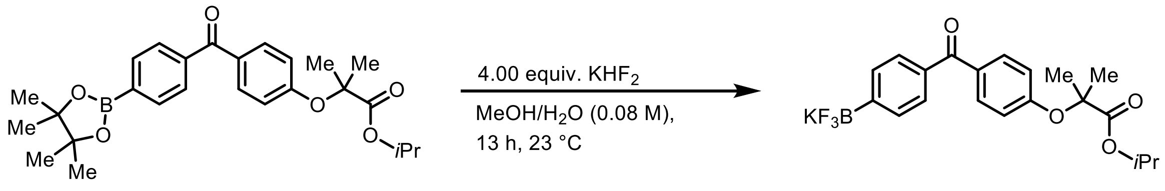

S11 **67% yield** **12**

To a 100-mL round bottom flask equipped with a magnetic stirring bar, isopropyl 2-methyl-2-(4-(4-(4,4,5,5-tetramethyl-1,3,2-dioxaborolan-2-yl)benzoyl)phenoxy)propanoate **S11** (905 mg, 2.00 mmol, 1.00 equiv.) and methanol (25.0 mL, 0.08 M) were added. Solution of potassium hydrogen fluoride (631 mg, 8.00 mmol, 4.00 equiv.) in water (5.00 mL, 1.60 M) was added to the stirring at 400 rpm mixture. The resulting mixture was stirred for 16 h at 23 °C. After 16 h, methanol was removed by rotary evaporation under reduced pressure, the water was removed by addition of CH_3CN (3×20 mL) and rotary evaporation of azeotropic mixture of solvents under reduced pressure. The resulting solid was dissolved in acetone, dried over $MgSO_4$, and filtered by gravity using an 8-cm diameter funnel containing a fluted filter paper. Then, the mixture was concentrated until saturation by rotary evaporation under reduced pressure, and Et_2O (90 mL) was added. The resulting precipitate was filtered, washed with Et_2O (2×10 mL), and dried under high vacuo to afford the desired product as a pale grey solid in 67% yield (594 mg, 1.37 mmol).

NMR Spectroscopy:

^{1}H NMR (700 MHz, DMSO-d$_6$, 23 °C, δ) 7.74 – 7.64 (m, 2H), 7.48 (q, *J* = 8.0 Hz, 4H), 6.95 – 6.83 (m, 2H), 4.98 (hept, *J* = 6.3 Hz, 1H), 1.60 (s, 6H), 1.15 (d, *J* = 6.3 Hz, 6H).

^{13}C NMR (176 MHz, DMSO-d$_6$, 23 °C, δ): 195.0, 172.2, 158.5, 134.5, 131.6, 131.2, 131.2, 130.9, 127.8, 117.0, 79.0, 68.9, 25.1, 21.2.

^{19}F NMR (659 MHz, DMSO-d$_6$, 23 °C, δ): –139.78 (d, *J* = 39.4 Hz).

^{11}B NMR (193 MHz, DMSO-d$_6$, 23 °C, δ): 3.01.

HRMS-ESI(m/z) calc'd for $C_{20}H_{21}BF_3O_4$ $[M]^-$, 393.1480; found, 393.1491; deviation: +2.8 ppm.

Selenonium fenofibrate derivative 13

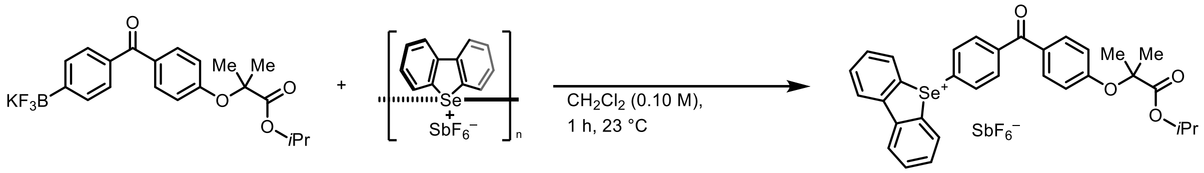

12, 1.00 equiv. **52%** **13**

Under an ambient atmosphere, to a 20-mL borosilicate vial equipped with a magnetic stir bar, selenurane **[2a]SbF$_6$** (467 mg, 1.00 mmol) and CH_2Cl_2 (5.00 mL, 0.10 M) were added. Then, **12** (223 mg, 0.5 mmol, 1.00 equiv.) was added portionwise to the stirring (200 rpm) mixture. The reaction mixture

was stirred at 23 °C for 1 h. After 1 h, the mixture was concentrated by rotary evaporation under reduced pressure. The residue was washed with Et_2O (3×10 mL) and purified for further characterization by flash column chromatography on silica gel using a gradient of eluent systems from pure $CHCl_3$ to MeOH/$CHCl_3$ (1:10 (v/v)) to afford the desired product as pale yellow solid in 52% yield (206 mg, 0.26 mmol).

R*f* = 0.19 (MeOH/$CHCl_3$ (1:10 (v/v))).

NMR Spectroscopy:

^{1}H NMR (700 MHz, DMSO-d_6, 23 °C, δ): 8.44 – 8.41 (m, 4H), 7.89 – 7.87 (m, 2H), 7.75 – 7.71 (m, 4H), 7.70 – 7.67 (m, 2H), 7.66 – 7.62 (m, 2H), 6.85 (d, *J* = 8.9 Hz, 2H), 4.95 (h, *J* = 6.3 Hz, 1H), 1.57 (s, 6H), 1.12 (d, *J* = 6.3 Hz, 6H).

^{13}C NMR (176 MHz, DMSO-d_6, 23 °C, δ): 193.0, 172.0, 159.6, 141.2, 140.5, 136.7, 132.9, 132.0, 130.9, 130.8, 129.9, 129.3, 128.8, 124.9, 117.1, 79.1, 68.9, 25.0, 21.2.

^{19}F NMR (659 MHz, DMSO-d_6, 23 °C, δ): –112.13 to –126.94 (m).

^{77}Se NMR (115 MHz, DMSO-d_6, 23 °C, δ): 519.1.

HRMS-ESI(m/z) calc'd for $C_{32}H_{29}O_4Se$ $[M]^+$, 557.1227; found, 557.1220; deviation: –1.3 ppm.

HRMS-ESI(m/z) calc'd for SbF_6 $[M]^-$, 234.8948; found, 234.8952; deviation: +1.7 ppm.

Bpin tianeptine intermediate S12

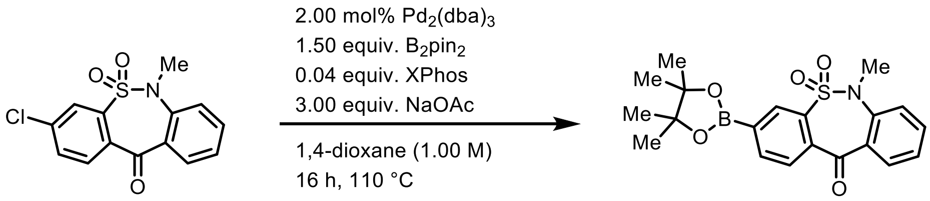

81% yield

S12

To an oven-dried 25-mL Schlenk tube equipped with a magnetic stirring bar, 3-chloro-6-methyldibenzo[*c*,*f*][1,2]thiazepin-11(6*H*)-one 5,5-dioxide (1.30 g, 4.00 mmol, 1.00 equiv.), tris(dibenzylideneacetone)dipalladium(0) (73.6 mg, 0.08 mmol, 2.00 mol%), bis(pinacolato)diboron (1.52 g, 6 mmol, 1.50 equiv.), XPhos (76.3 mg, 0.16 mmol, 0.04 equiv.) and sodium acetate (984 mg, 12.0 mmol, 3.00 equiv.) were added. The tube was sealed with a septum, connected via a Tygon® tubing to a Schlenk line, and evacuated and purged with argon three times. 1,4-Dioxane (4.00 mL, 1.00 M) was added, the septum was equipped with an Ar-filled balloon, and the reaction mixture was stirred at 110 °C for 16 h using an oil bath. After 16 h, the flask was removed from the oil bath and allowed to cool to 23 °C (ca. 30 minutes). Then, water (10 mL) was added, the resulting mixture was transferred to a 250-mL separation funnel. Organic phase was separated and aqueous phase was extracted with EtOAc (3×20 mL). The combined organic phase was dried over Na_2SO_4, and filtered by gravity using an 8-cm diameter funnel containing a fluted filter paper. Then, the resulting mixture was concentrated

by rotary evaporation under reduced pressure. The residue was purified by flash column chromatography on silica gel using a gradient of eluent systems from EtOAc/heptane (1:1 (v/v)) to EtOAc/heptane (3:1 (v/v)) to afford the desired product as a pale yellow solid in 81% yield (1.30 g, 3.25 mmol).

R*f* = 0.53 (EtOAc/heptane (1:2 (v/v))).

NMR Spectroscopy:

^{1}H NMR (600 MHz, $CDCl_3$, 23 °C, δ): 8.35 (s, 1H), 8.28 (d, *J* = 8.1 Hz, 1H), 8.11 (dd, *J* = 7.6, 0.9 Hz, 1H), 7.90 (d, *J* = 7.6 Hz, 1H), 7.65 – 7.60 (m, 1H), 7.35 (t, *J* = 7.6 Hz, 1H), 7.32 (d, *J* = 8.1 Hz, 1H), 3.34 (s, 3H), 1.38 (s, 12H).

^{13}C NMR (151 MHz, $CDCl_3$, 23 °C, δ): 191.4, 142.0, 139.5, 138.6, 136.1, 134.8, 132.0, 131.3, 130.9, 130.5, 125.9, 124.4, 84.9, 39.1, 25.0.

^{11}B NMR (193 MHz, DMSO-d_6, 23 °C, δ): 30.42.

HRMS-ESI(m/z) calc'd for $C_{20}H_{22}BNO_5SNa$ $[M+Na]^+$, 422.1205; found, 422.1203; deviation: –0.5 ppm.

BF₃K tianeptine intermediate 14

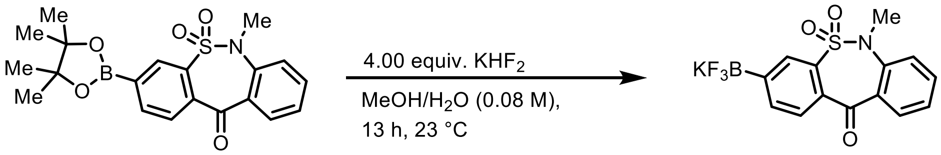

S12 **47% yield** **14**

To a 100-mL round bottom flask equipped with a magnetic stirring bar, isopropyl 6-methyl-3-(4,4,5,5-tetramethyl-1,3,2-dioxaborolan-2-yl)dibenzo[*c*,*f*][1,2]thiazepin-11(6*H*)-one 5,5-dioxide **S12** (799 mg, 2.00 mmol, 1.00 equiv.) and methanol (25.0 mL, 0.08 M) were added. Solution of potassium hydrogen fluoride (631 mg, 8.00 mmol, 4.00 equiv.) in water (5.00 mL, 1.60 M) was added to the stirring (400 rpm) mixture. The resulting mixture was stirred for 16 h at 23 °C. After 16 h, methanol was removed by rotary evaporation under reduced pressure, the water was removed by addition of CH_3CN (3×20 mL) and rotary evaporation of azeotropic mixture of solvents under reduced pressure. The resulting solid was dissolved in acetone, dried over $MgSO_4$, and filtered by gravity using an 8-cm diameter funnel containing a fluted filter paper. Then, the mixture was concentrated until saturation by rotary evaporation under reduced pressure, and Et_2O (90 mL) was added. The resulting precipitate was filtered, washed with Et_2O (2×10 mL), and dried under high vacuo to afford the desired product as a white solid in 47% yield (361 mg, 0.95 mmol).

NMR Spectroscopy:

^{1}H NMR (700 MHz, DMSO-d_6, 23 °C, δ): 8.14 (dd, *J* = 8.1, 1.6 Hz, 1H), 7.85 (s, 1H), 7.77 (d, *J* = 7.4 Hz, 1H), 7.73 (ddd, *J* = 8.2, 7.2, 1.7 Hz, 1H), 7.67 (d, *J* = 7.4 Hz, 1H), 7.56 (dd, *J* = 8.1, 0.9 Hz, 1H), 7.42 (ddd, *J* = 8.2, 7.2, 1.1 Hz, 1H), 3.25 (s, 3H).

^{13}**C NMR** (176 MHz, DMSO-d$_6$, 23 °C, δ): 191.8, 141.7, 136.5, 134.8, 133.7, 133.3, 130.8, 130.2, 129.0, 127.0, 125.5, 124.9, 38.7.

^{19}**F NMR** (659 MHz, DMSO-d$_6$, 23 °C, δ): –140.43.

^{11}**B NMR** (193 MHz, DMSO-d$_6$, 23 °C, δ): 2.54.

HRMS-ESI(m/z) calc'd for $C_{14}H_{10}BF_3NO_3S$ [M]$^-$, 340.0421; found, 340.0435; deviation: +4.1 ppm.

Selenonium tianeptine derivative 15

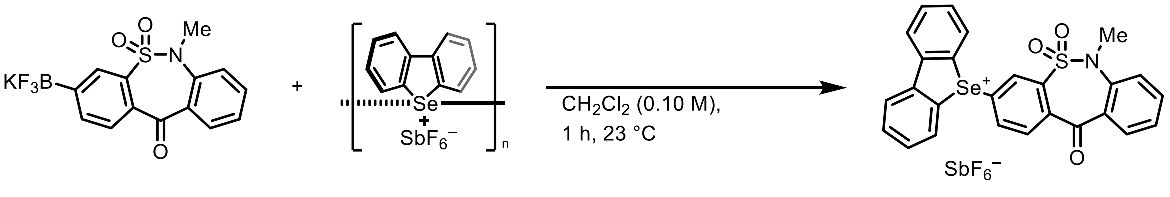

14, 1.00 equiv. **58%** **15**

Under an ambient atmosphere, to a 20-mL borosilicate vial equipped with a magnetic stir bar, selenurane **[2a]SbF$_6$** (467 mg, 1.00 mmol) and CH_2Cl_2 (5.00 mL, 0.10 M) were added. Then, **14** (195 mg, 0.5 mmol, 1.00 equiv.) was added portionwise to the stirring (200 rpm) mixture. The reaction mixture was stirred at 23 °C for 1 h. After 1 h, the mixture was concentrated by rotary evaporation under reduced pressure. The residue was washed with Et_2O (3×10 mL) and purified for further characterization by flash column chromatography on silica gel using a gradient of eluent systems from pure $CHCl_3$ to MeOH/$CHCl_3$ (1:10 (v/v)) to afford the desired product as pale yellow solid in 58% yield (216 mg, 0.292 mmol).

R$_f$ = 0.18 (MeOH/$CHCl_3$ (1:10 (v/v))).

NMR Spectroscopy:

^{1}**H NMR** (700 MHz, DMSO-d$_6$, 23 °C, δ): 8.55 (d, *J* = 1.8 Hz, 1H), 8.47 – 8.43 (m, 2H), 8.41 – 8.37 (m, 2H), 8.06 (dd, *J* = 8.1, 1.6 Hz, 1H), 7.89 (td, *J* = 7.7, 0.9 Hz, 2H), 7.85 (d, *J* = 8.3 Hz, 1H), 7.78 (ddd, *J* = 8.6, 7.3, 1.6 Hz, 1H), 7.75 – 7.71 (m, 2H), 7.62 – 7.58 (m, 1H), 7.48 (dd, *J* = 8.4, 1.8 Hz, 1H), 7.46 – 7.42 (m, 1H), 3.27 (s, 3H).

^{13}**C NMR** (176 MHz, DMSO-d$_6$, 23 °C, δ): 190.2, 141.3, 141.1, 138.1, 137.8, 136.7, 136.5, 135.6, 133.3, 133.3, 133.1, 131.1, 130.9, 129.8, 129.3, 127.0, 126.1, 125.1, 124.9, 38.7.

^{19}**F NMR** (659 MHz, DMSO-d$_6$, 23 °C, δ): –112.12 to –126.96 (m).

^{77}**Se NMR** (115 MHz, DMSO-d$_6$, 23 °C, δ): 521.2.

HRMS-ESI(m/z) calc'd for $C_{26}H_{18}NO_3SSe$ [M]$^+$, 604.0167;

HRMS-ESI(m/z) calc'd for SbF_6 [M]$^-$, 234.8948; found, 234.8950; deviation: +0.8 ppm.

Preparation of Selenonium Salts from Cationic Selenuranes – Additional Examples

5-(4-(*Tert*-butyl)phenyl) -dibenzoselenophenium hexafluoroantimonate (S13)

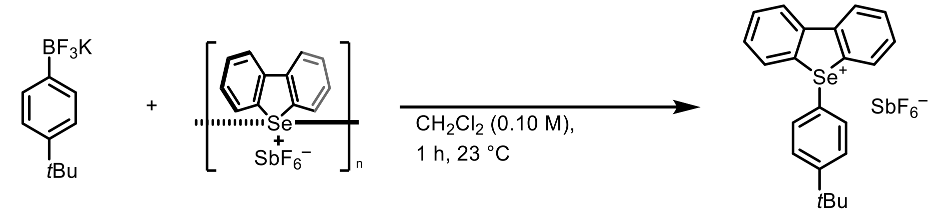

2.00 equiv. **97% yield** **S13**

Under an ambient atmosphere, to a 20-mL borosilicate vial equipped with a magnetic stir bar, selenurane **[2a]SbF6** (1.00 g, 2.14 mmol) and CH_2Cl_2 (10.0 mL, 0.11 M) were added. Then, potassium (4-(*tert*-butyl)phenyl)trifluoroborate (525 mg, 2.14 mmol, 2.00 equiv.) was added portionwise to the stirring (200 rpm) mixture. The reaction mixture was stirred at 23 °C for 1 h. After 1 h, the mixture was concentrated by rotary evaporation under reduced pressure. The residue was washed with Et_2O (3×20 mL) and purified for further characterization by flash column chromatography on silica gel using a gradient of eluent systems from MeOH/$CHCl_3$ (1:20 (v/v)) to MeOH/$CHCl_3$ (1:10 (v/v)) to afford the desired product as beige crystalline solid in 97% yield (622 mg, 1.04 mmol).

R_f = 0.12 (MeOH/$CHCl_3$ (1:10 (v/v))).

NMR Spectroscopy:

^{1}H NMR (400 MHz, DMSO-d_6, 23 ºC, δ): 8.41 (d, *J* = 7.6 Hz, 2H), 8.33 (d, *J* = 7.9 Hz, 2H), 7.86 (t, *J* = 7.6 Hz, 2H), 7.68 (t, *J* = 7.7 Hz, 2H), 7.50 (d, *J* = 8.6 Hz, 2H), 7.38 (d, *J* = 8.6 Hz, 2H), 1.19 (s, 9H).

^{13}C NMR (151 MHz, DMSO-d_6, 23 ºC, δ): 155.1, 141.1, 137.0, 132.7, 130.8, 129.7, 129.6, 128.9, 127.7, 124.8, 34.7, 30.7.

^{19}F NMR (377 MHz, DMSO-d_6, 23 ºC, δ): –106.54 to –132.52 (m).

^{77}Se NMR (115 MHz, DMSO-d_6, 23 ºC, δ): 516.6.

HRMS-ESI(m/z) calc'd for $C_{22}H_{21}Se$ $[M]^+$, 365.0803; found, 365.0797; deviation: –1.6 ppm.

HRMS-ESI(m/z) calc'd for SbF_6 $[M]^-$, 234.8948; found, 234.8947; deviation: –0.4 ppm.

5-(4-Methoxyphenyl)-5*H*-dibenzo[*b*,*d*]selenophen-5-ium hexafluoroantimonate (S14)

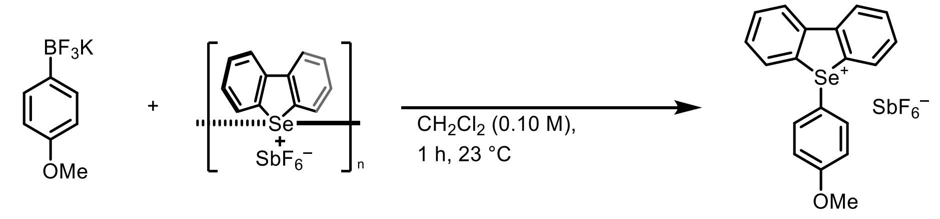

2.00 equiv. **85% yield** **S14**

Under an ambient atmosphere, to a 4-mL borosilicate vial equipped with a magnetic stir bar, selenurane **[2a]SbF$_6$** (187 mg, 0.40 mmol) and CH_2Cl_2 (2.00 mL, 0.10 M) were added. Then, potassium (4-methoxyphenyl)trifluoroborate (86 mg, 0.40 mmol, 2.00 equiv.) was added portionwise to the stirring (200 rpm) mixture. The reaction mixture was stirred at 23 °C for 1 h. After 1 h, the mixture was concentrated by rotary evaporation under reduced pressure. The residue was washed with Et_2O (3×4 mL) and purified for further characterization by flash column chromatography on silica gel using a MeOH/$CHCl_3$ (1:10 (v/v)) as eluent to afford the desired product as pink solid in 85% yield (97 mg, 0.17 mmol).

R$_f$ = 0.26 (MeOH/$CHCl_3$ (1:10 (v/v))).

NMR Spectroscopy:

^{1}H NMR (400 MHz, DMSO-d$_6$, 23 °C, δ): 8.44 – 8.37 (m, 2H), 8.34 – 8.29 (m, 2H), 7.85 (td, *J* = 7.7, 1.1 Hz, 2H), 7.68 (td, *J* = 7.9, 1.2 Hz, 2H), 7.42 – 7.33 (m, 2H), 7.06 – 6.99 (m, 2H), 3.72 (s, 3H).

^{13}C NMR (101 MHz, DMSO-d$_6$, 23 °C, δ): 161.9, 141.0, 137.4, 132.7, 131.0, 130.8, 129.6, 124.8, 122.6, 116.1, 55.6.

^{19}F NMR (659 MHz, DMSO-d$_6$, 23 °C, δ): –112.12 to –126.97 (m).

^{77}Se NMR (115 MHz, DMSO-d$_6$, 23 °C, δ): 513.8.

HRMS-ESI(m/z) calc'd for $C_{19}H_{15}OSe$ $[M]^+$, 339.0283; found, 339.0278; deviation: –1.5 ppm.

HRMS-ESI(m/z) calc'd for SbF_6 $[M]^-$, 234.8948; found, 234.8946; deviation: –0.9 ppm.

5-(4-(Trifluoromethyl)phenyl)-5*H*-dibenzo[*b*,*d*]selenophen-5-ium hexafluoroantimonate (S15)

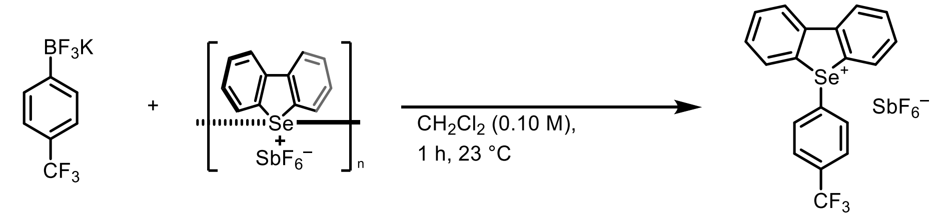

2.00 equiv. **72% yield** **S15**

Under an ambient atmosphere, to a 4-mL borosilicate vial equipped with a magnetic stir bar, selenurane **[2a]SbF$_6$** (187 mg, 0.40 mmol) and CH_2Cl_2 (2.00 mL, 0.10 M) were added. Then, potassium (4-(trifluoromethyl)phenyl)trifluoroborate (101 mg, 0.40 mmol, 2.00 equiv.) was added portionwise to the stirring (200 rpm) mixture. The reaction mixture was stirred at 23 °C for 1 h. After 1 h, the mixture was concentrated by rotary evaporation under reduced pressure. The residue was washed with Et_2O (3×4 mL) and purified for further characterization by flash column chromatography on silica gel using MeOH/$CHCl_3$ (1:10 (v/v)) as eluent to afford the desired product as white solid in 72% yield (88 mg, 0.14 mmol).

R_f = 0.38 (MeOH/$CHCl_3$ (1:10 (v/v))).

NMR Spectroscopy:

^{1}H NMR (700 MHz, DMSO-d6, 23 °C, δ): 8.44 – 8.41 (m, 2H), 8.40 – 8.38 (m, 2H), 7.89 – 7.85 (m, 4H), 7.74 (d, *J* = 8.5 Hz, 2H), 7.71 (td, *J* = 7.9, 1.2 Hz, 2H).

^{13}C NMR (176 MHz, DMSO-d6, 23 °C, δ): 141.2, 138.3, 136.7, 133.0, 132.0, 131.7 (q, *J* = 32.5 Hz), 130.22, 129.8, 127.4 (q, *J* = 3.7 Hz), 125.0, 123.3 (q, *J* = 273 Hz).

^{19}F NMR (659 MHz, DMSO-d6, 23 °C, δ): –61.69, –112.08 to –126.98 (m).

^{77}Se NMR (115 MHz, DMSO-d6, 23 °C, δ): 519.2.

HRMS-ESI(m/z) calc'd for $C_{19}H_{12}F_3Se$ $[M]^+$, 377.0051; found, 377.0047; deviation: –1.1 ppm.

HRMS-ESI(m/z) calc'd for SbF_6 $[M]^-$, 234.8948; found, 234.8948; deviation: 0.0 ppm.

5-(*O*-tolyl)-5*H*-dibenzo[*b*,*d*]selenophen-5-ium hexafluoroantimonate (S16)

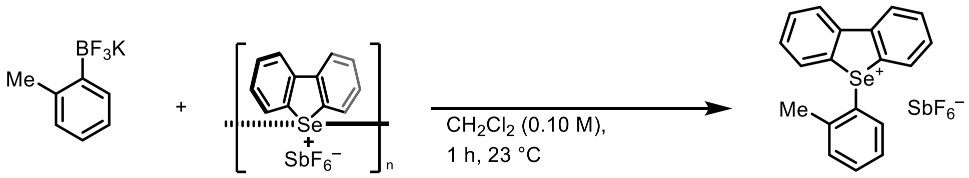

2.00 equiv. **75% yield** **S16**

Under an ambient atmosphere, to a 4-mL borosilicate vial equipped with a magnetic stir bar, selenurane **[2a]SbF_6** (187 mg, 0.40 mmol) and CH_2Cl_2 (2.00 mL, 0.10 M) were added. Then, potassium trifluoro(*o*-tolyl)borate (84.0 mg, 0.40 mmol, 2.00 equiv.) was added portionwise to the stirring (200 rpm) mixture. The reaction mixture was stirred at 23 °C for 1 h. After 1 h, the mixture was concentrated by rotary evaporation under reduced pressure. The residue was washed with Et_2O (3×4 mL) and purified for further characterization by flash column chromatography on silica gel using MeOH/$CHCl_3$ (1:10 (v/v)) as eluent to afford the desired product as beige crystalline solid in 75% yield (84 mg, .15 mmol).

R_f = 0.20 (MeOH/$CHCl_3$ (1:10 (v/v))).

NMR Spectroscopy:

^{1}H NMR (400 MHz, DMSO-d6, 23 °C, δ): 8.47 (d, J = 6.5 Hz, 2H), 8.28 (d, J = 8.0 Hz, 2H), 7.89 (td, J = 7.7, 0.9 Hz, 2H), 7.69 (td, J = 8.0, 1.2 Hz, 2H), 7.54 (d, J = 5.6 Hz, 1H), 7.46 (t, J = 7.4 Hz, 1H), 7.12 (t, J = 6.6 Hz, 1H), 6.40 (d, J = 9.5 Hz, 1H), 2.96 (s, 3H).

^{13}C NMR (101 MHz, DMSO-d6, 23 °C, δ): 141.2, 140.0, 136.5, 133.0, 132.8, 132.5, 132.4, 131.1, 129.7, 128.8, 126.7, 125.2, 21.0.

^{19}F NMR (659 MHz, DMSO-d6, 23 °C, δ): –112.14 to –126.98 (m).

^{77}Se NMR (115 MHz, DMSO-d6, 23 °C, δ): 497.2.

HRMS-ESI(m/z) calc'd for $C_{19}H_{15}Se$ $[M]^+$, 323.0333; found, 323.0329; deviation: –1.2 ppm.

HRMS-ESI(m/z) calc'd for SbF_6 $[M]^-$, 234.8948; found, 234.8948; deviation: 0.0 ppm.

Group transfer reactivity summary

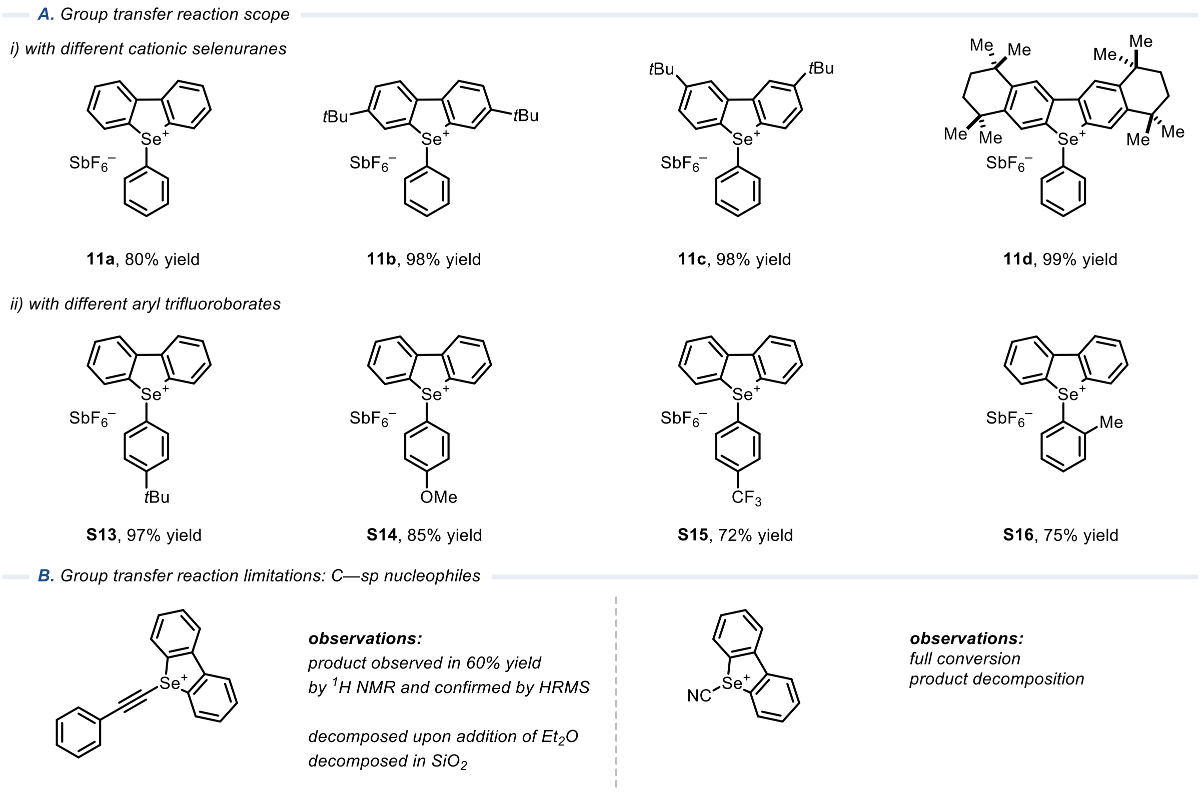

Figure S18. Summary of the group transfer reactions carried out during this study. **A.** *i)* Products derived from the reactions between different cationic selenuranes and $PhBF_3K$; and *ii)* Products derived from the reactions between **[2a]SbF₆** and Ar–BF_3K salts. **B.** Limitations of the reported group transfer reactions.

PROPOSED RADICAL REACTIVITY OF SE(III) RADICALS

Radical trap experiment

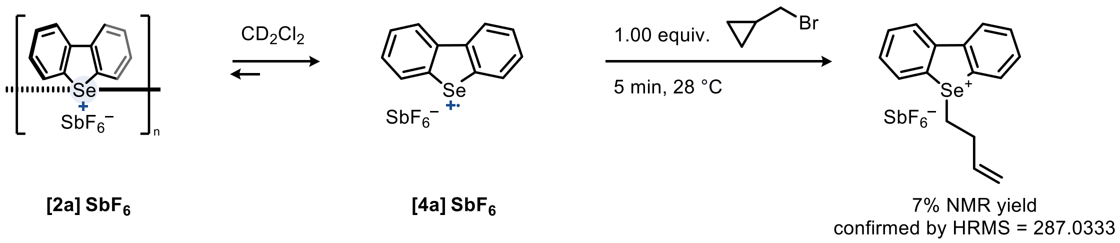

Upon dissolution of cationic selenurane **[2a]SbF₆** in CD_2Cl_2, 1.00 equivalent of (bromomethyl)cyclopropane was added. The deep blue color associated with Se(III) radicals faded immediately. The formation of an alkyl selenonium salt (7% yield by ^{1}H NMR using CH_2Br_2 as internal standard; confirmed by HRMS) is consistent with a cyclopropyl ring-opening event, suggesting the intermediacy of carbon-centered radicals trapped by **4a** and thus supporting the involvement of Se(III) radicals in the reaction.

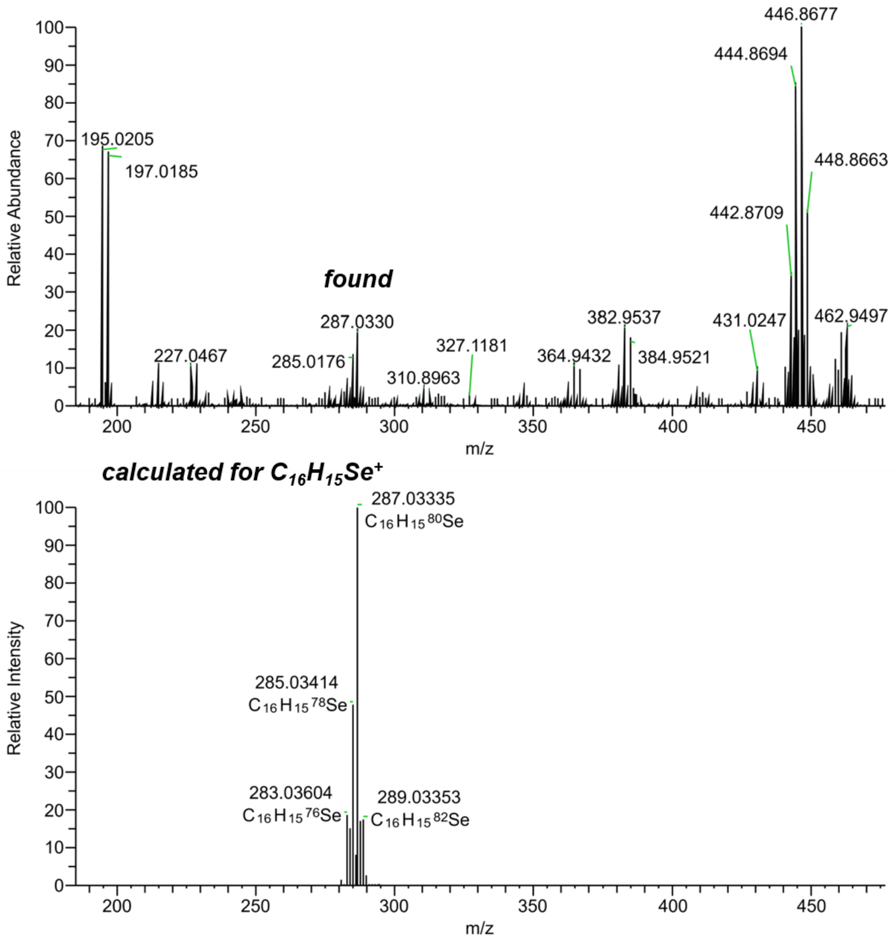

Figure S19. HRMS spectra of the alkyl selenonium salt produced from the radical clock experiment in positive ion mode.

Modification of selenonium salts with previously reported methods for sulfonium salts

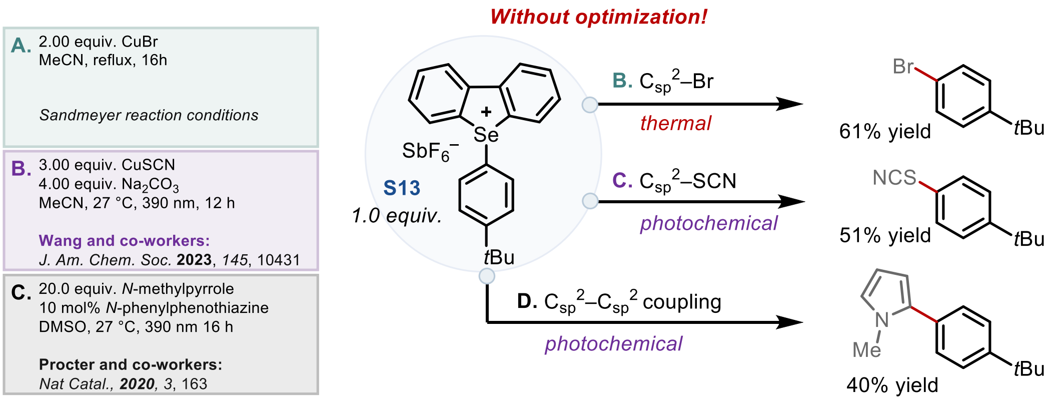

Figure S20. Summary of the selenonium salt manipulations carried out in this study by implementing previously reported conditions for the modification of sulfonium salts. No further optimizations were required.

A. *Thermal Csp²–Br transformation*

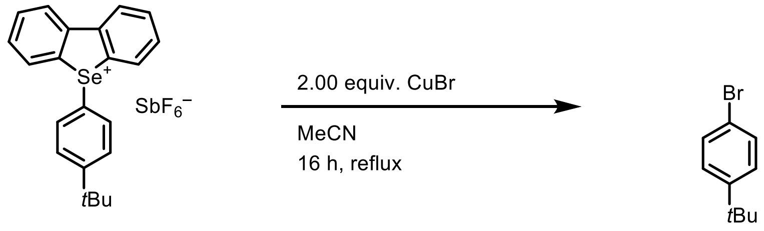

S13, 1.00 equiv. **63% yield**

A 4-mL vial equipped with a magnetic stir bar was flushed with argon for 2 min, and was charged with **S13** (30.0 mg, 0.050 mmol, 1.00 equiv.) and copper bromide (14.3 mg, 0.100 mmol, 2.00 equiv.). Then, acetonitrile was added (0.5 mL) using a 1.0-mL syringe. The vial was sealed with a PTFE cap and the reaction mixture was stirred (500 ppm) and heated at 80 °C for 16 h using an aluminum heating block. After 16 h, the vial was removed from the heating block and allowed to cool to 23 °C (ca. 10 minutes). The crude product was filtered through a short silica plug and concentrated *in vacuo*. Then, the residue was solubilized in $CDCl_3$ (0.50 mL), transferred to an NMR tube, CH_2Br_2 (7.0 µL, 0.10 mmol, 2.0 equiv.) was added as internal standard, and the yield was determined by ^{1}H NMR spectroscopy by comparing the integration of the CH_2Br_2 signal at 4.93 ppm (s, 2H) to the signal of the product at 7.36 ppm (d, 2H). (Yield = 0.63/1 = 63% yield).

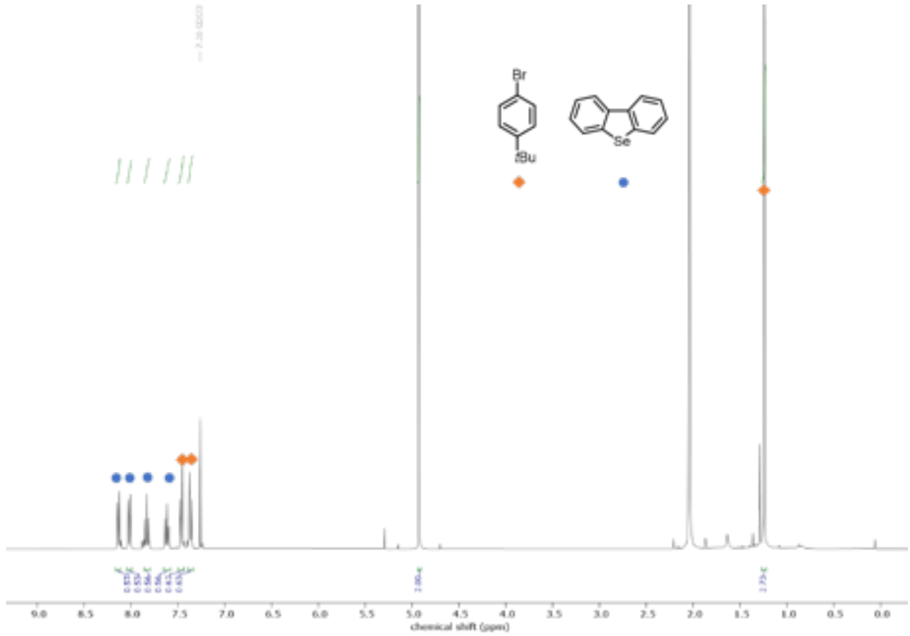

Figure S21. ^{1}H NMR spectrum at 500 MHz recorded in $CDCl_3$ solution of the reaction mixture for the formation of the aryl thiocyanate. The yield was determined by ^{1}H NMR spectroscopy at 500 MHz and 298 K by dissolving the residue of the reaction mixture in 0.5 mL of $CDCl_3$, and using CH_2Br_2 as internal standard (7.0 µL, 17 mg, 0.100 mmol, 1.0 equiv.). The integration of the CH_2Br_2 signal at 4.93 ppm (s, 2H, 2.0 equiv.) was compared to the signal of the product at 7.36 ppm (d, 2H, 1.00 equiv.). Orange diamonds = aryl thiocyanate, blue balls = dibenzoselenophene **1a**.

B. Photochemical Csp²–SCN transformation

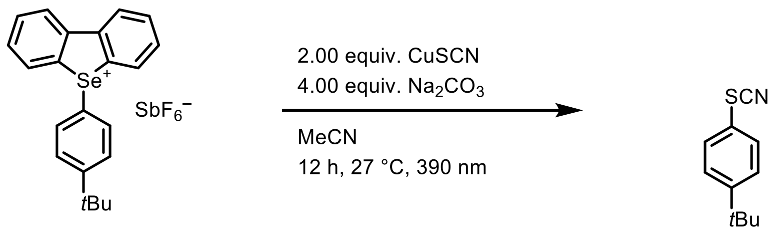

S13, 1.00 equiv. **52% yield**

A 4-mL vial equipped with a magnetic stir bar was flushed with argon for 2 min, and was charged with **S13** (60.0 mg, 0.100 mmol, 1.00 equiv.), copper thiocyanate (36.5 mg, 0.200 mmol, 2.00 equiv.), and sodium carbonate (42.4 mg, 0.400 mmol, 4.00 equiv.). Then, acetonitrile was added (1.0 mL) using a 1.0-mL syringe. The vial was sealed with a PTFE cap, the reaction mixture was stirred (500 ppm), and irradiated (390 nm EvoluChem lamp) at 27 °C for 16 h. After 16 h, the vial was removed from the light source. The crude product was filtered through a short silica plug and concentrated *in vacuo*. Then, the residue was fully solubilized in $CDCl_3$ (0.50 mL), transferred to an NMR tube, CH_2Br_2 (7.1 µL, 0.10 mmol, 1.0 equiv.) was added as internal standard, and the yield was determined by ^{1}H NMR spectroscopy by comparing the integration of the CH_2Br_2 signal at 4.93 ppm (s, 2H) to the signal of the product at 1.33 ppm (s, 9H). (Yield = 4.60/9.00 = 52%)

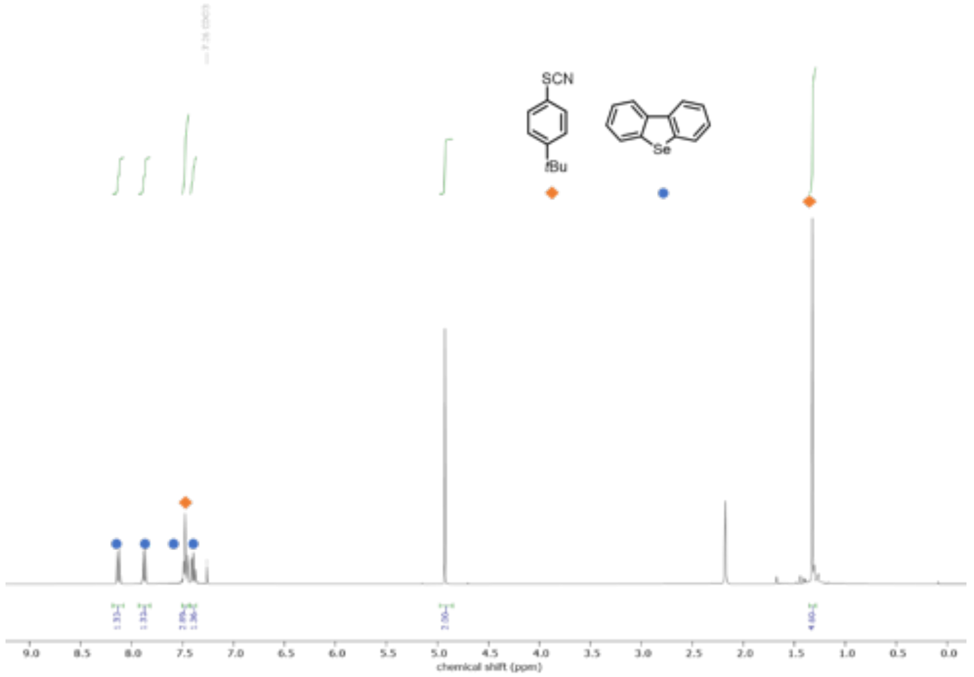

Figure S22. ^{1}H NMR spectrum at 500 MHz recorded in $CDCl_3$ solution of the reaction mixture for the formation of the aryl thiocyanate. The yield was determined by ^{1}H NMR spectroscopy at 500 MHz and 298 K by dissolving the residue of the reaction mixture in 0.5 mL of $CDCl_3$, and using CH_2Br_2 as internal standard (7.0 µL, 17 mg, 0.10 mmol, 1.0 equiv.). The integration of the CH_2Br_2 signal at 4.93 ppm (s, 2H) was compared to the signal of the product at 1.33 ppm (s, 9H). Orange diamonds = aryl thiocyanate, blue balls = dibenzoselenophene **1a**.

C. *Photochemical Csp^2–Csp^2 coupling*

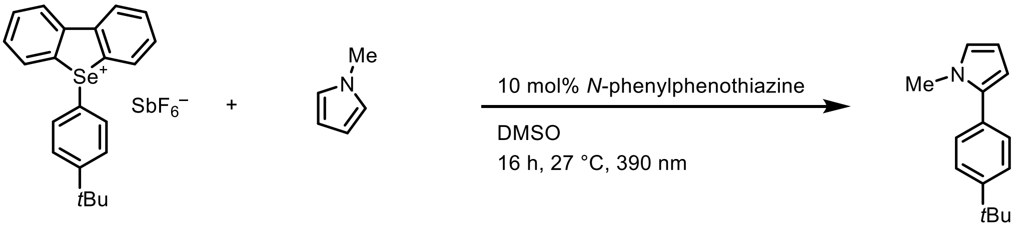

S13, 1.00 equiv. 20.0 equiv. **37% yield**

A 4-mL vial equipped with a magnetic stir bar was flushed with argon for 2 min, and was charged with **S13** (60.0 mg, 0.100 mmol, 1.00 equiv.), *N*-methylpyrrole (162 µL, 2.00 mmol, 20.0 equiv.), and *N*-phenylphenothiazine (2.8 mg, 0.010 mmol, 10 mol%). Then, DMSO-d6 was added (1.0 mL) using a 1.0-mL syringe. The vial was sealed with a PTFE cap, the reaction mixture was stirred (500 ppm), and irradiated (390 nm EvoluChem lamp) at 27 °C for 16 h. After 16 h, the vial was removed from the light source, and the volatiles (*N*-metyhlpyrrole excess) were removed *in vacuo*. Then, CH_2Br_2 (7.1 µL, 0.10 mmol, 1.0 equiv.) was added to the reaction mixture as internal standard, and 0.5 mL of the solution was transferred to an NMR tube. The yield was determined by ^{1}H NMR spectroscopy by comparing the integration of the CH_2Br_2 signal at 4.82 ppm (s, 2H) to the signal of the product at 3.57 ppm (s, 3H). (Yield = 1.10/3.00 = 37%)

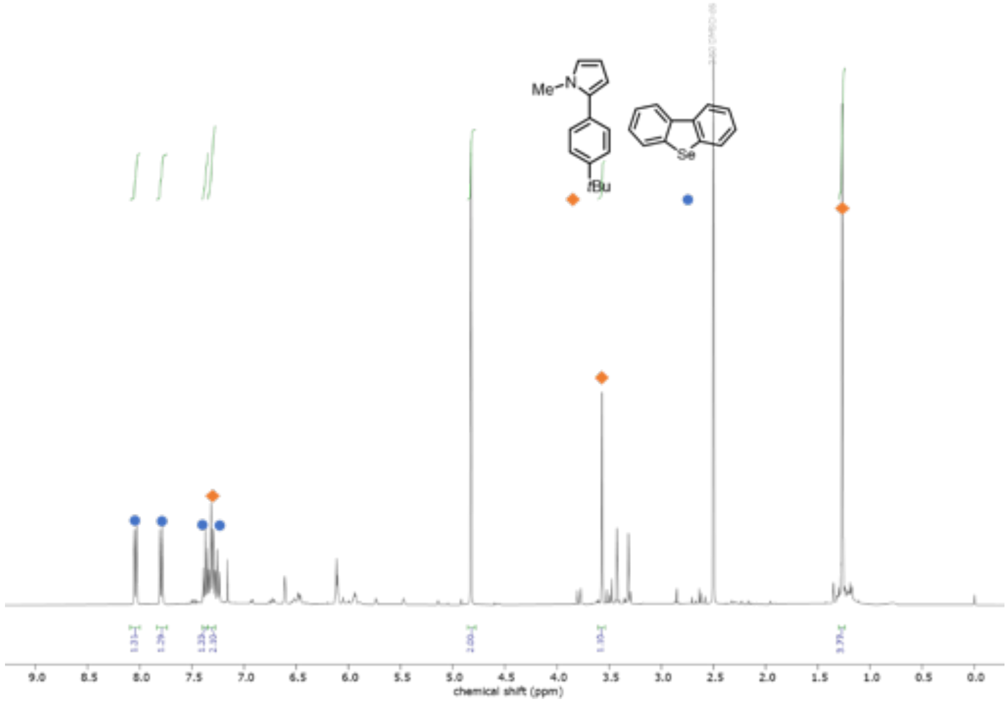

Figure S23. ^{1}H NMR spectrum at 500 MHz recorded in DMSO-d6 solution of the reaction mixture for the formation of the 2-aryl pyrrole. The yield was determined by ^{1}H NMR spectroscopy at 500 MHz and 298 K by dissolving the residue of the reaction mixture in 0.5 mL of DMSO-d6, and using CH_2Br_2 as internal standard (7.0 µL, 17 mg, 0.10 mmol, 1.0 equiv.). The integration of the CH_2Br_2 signal at 4.82 ppm (s, 2H) to the signal of the product at 3.57 ppm (s, 3H). Orange diamonds = aryl thiocyanate, blue balls = dibenzoselenophene **1a**.

Experimental data – Additional characterization

^{77}Se MAS-NMR spectroscopy of cationic selenuranes

This section intends to give additional information on the solid-state structure of cationic selenuranes in addition to the data of the previous section. For this reason, MAS-NMR spectra of cationic selenuranes **[2a]SbF$_6$-[2d]SbF$_6$** were measured.

Sample preparation: pre-weighted samples (in a glovebox) of cationic selenuranes (200.0 mg) were transferred to a laser marked 4 mm zirconia bore-rotor under a flow of argon, the rotor was capped, and the sample was introduced in the spectrometer for analysis.

Experiment parameters: The resonance frequency for ^{77}Se was set to 95.4 MHz, the MAS rotor spinning to 15 kHz. Except for **[2c]SbF$_6$**, which was recorded by direct excitation, cross polarization (CP) was used applying a ramped contact pulse with 8 ms contact time. The relaxation delay was set individually for every sample in a range between 10 s up to 300 s. During acquisition ^{1}H was high power decoupled using a swept-frequency two-pulse phase modulation scheme. To identify the isotropic chemical shift, some of the experiments were repeated using a rotor spinning speed of 8 or 11 kHz.

Isotropic chemical shifts are marked with an asterisk (*) in the spectra.

^{77}Se MAS-NMR of dibenzo[*b*,*d*]selenophene (1a)

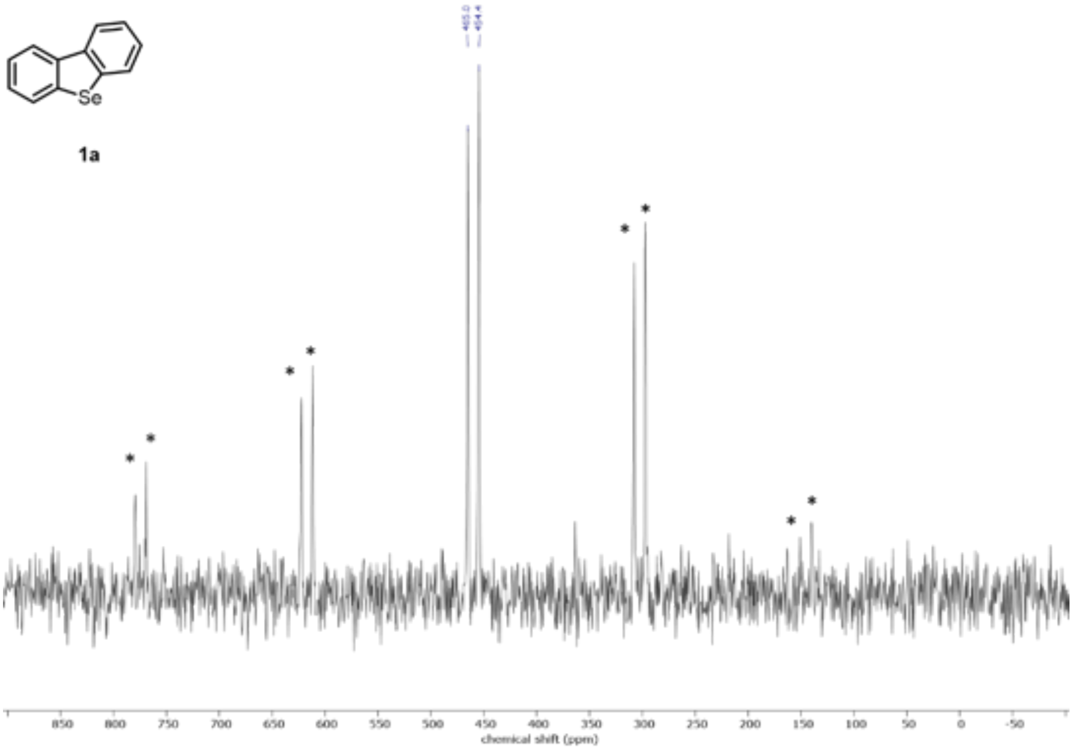

Figure S24. ^{77}Se MAS-NMR of **1a**. *isotropic chemical shift.

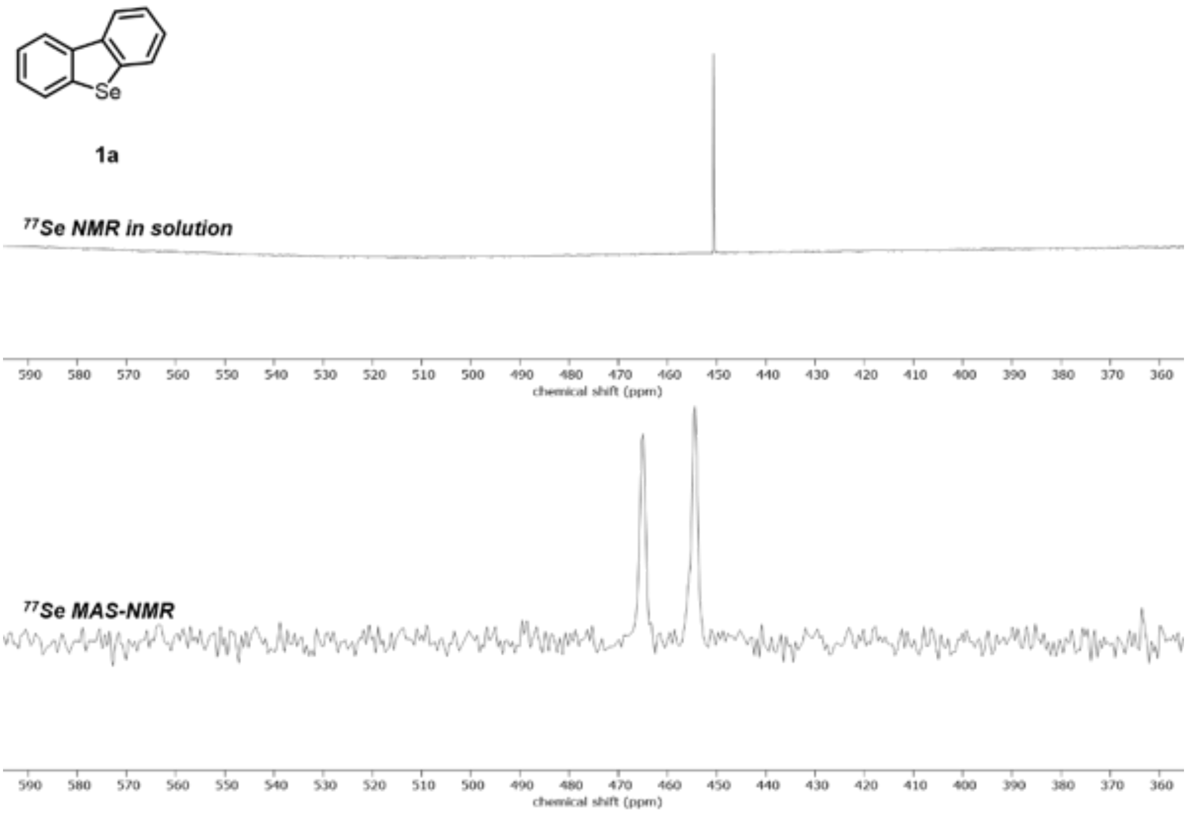

Figure S25. Comparison of ^{77}Se NMR spectra of **1a. Top:** in $CDCl_3$ solution ^{77}Se NMR vs. **Bottom:** ^{77}Se MAS-NMR in solid state. *isotropic chemical shift.

***1a** was used for method benchmarking. In the MAS-NMR spectrum of **1a** two signals centered at 460 ppm were identified. The central signal in the MAS-NMR experiment matches with the signal of **1a** in ^{77}Se NMR in solution (Figure S22) highlighting the utility of ^{77}Se MAS-NMR for the oxidation state determination of organoselenium compounds with similar structures.*

^{77}Se MAS-NMR of cationic selenurane [2a]SbF$_6$

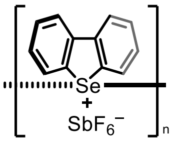

[2a] SbF$_6$

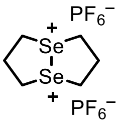

Furukawa dication
I

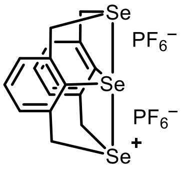

Furukawa dicationic selenurane
II

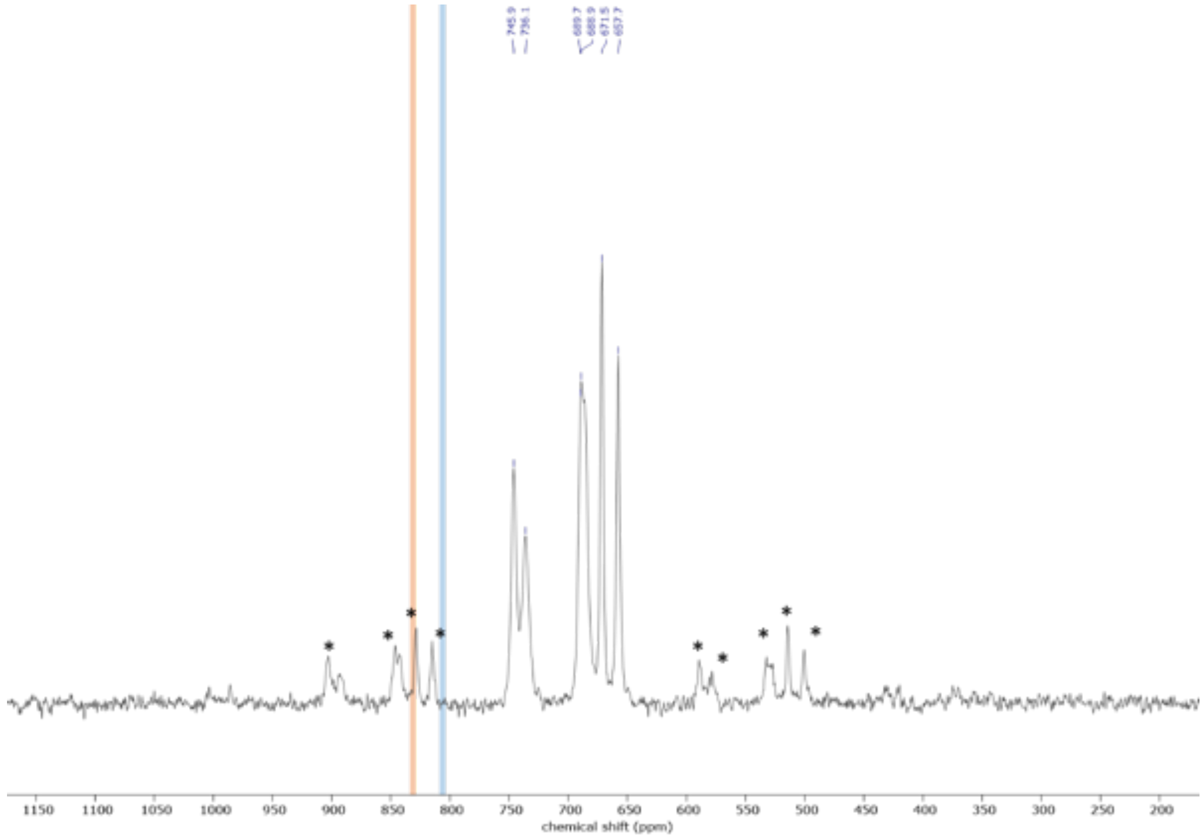

Figure S26. ^{77}Se MAS-NMR of cationic selenurane **[2a]SbF$_6$**. *isotropic chemical shift.

*In the MAS-NMR spectrum of **[2a]SbF$_6$** several (six) signals at the 750 to 650 ppm region (centered at 700 ppm) were identified. The signals for cationic Se(III) species previously reported by Furukawa are highlighted for clarity: a) at 806.5 ppm for Furukawa's dication **I** (blue line), and b) at 830.0 ppm for Furukawa's dicationic selenurane **II** (orange line).*[22],[23]

^{77}Se MAS-NMR of cationic selenurane [2b]SbF_6

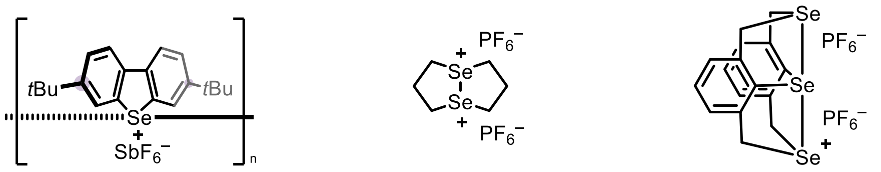

[2b] SbF_6

Furukawa dication
I

Furukawa dicationic selenurane
II

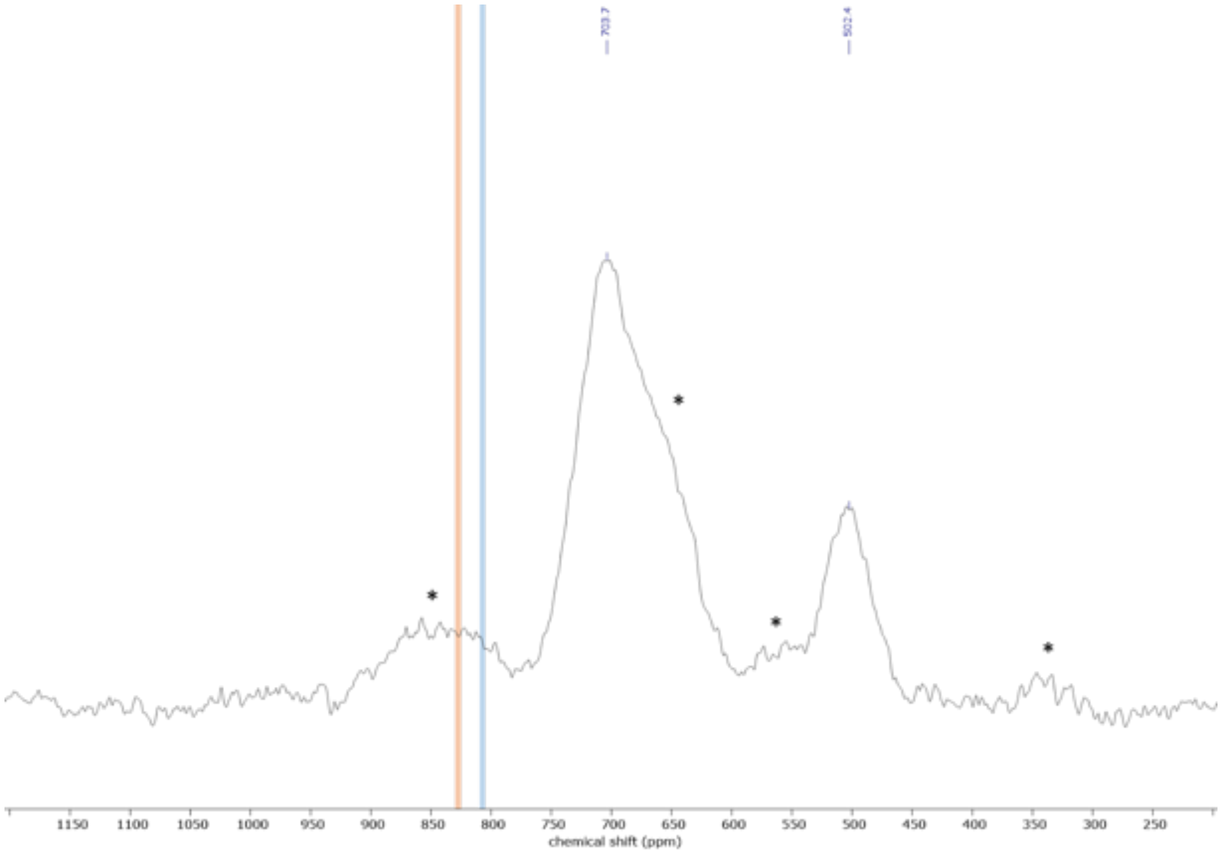

Figure S27. ^{77}Se MAS-NMR of cationic selenurane **[2b]SbF_6**. *isotropic chemical shift.

*In the MAS-NMR spectrum of **[2b]SbF_6** two broad signals at 704 and 500 ppm were identified. The signals for cationic Se(III) species previously reported by Furukawa are highlighted for clarity: a) at 806.5 ppm for Furukawa's dication **I** (blue line), and b) at 830.0 ppm for Furukawa's dicationic selenurane **II** (orange line).[22],[23]*

^{77}Se MAS-NMR of cationic selenurane [2c]SbF6

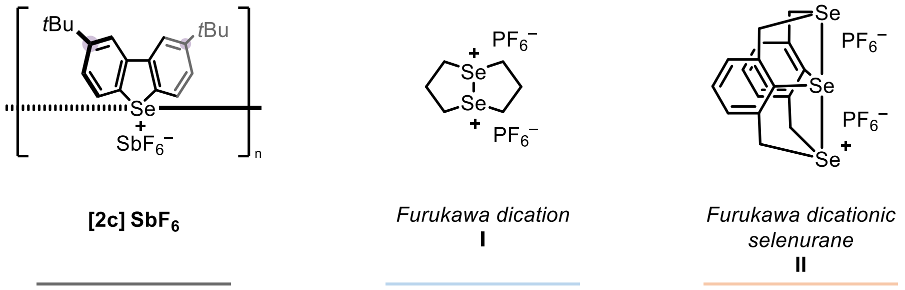

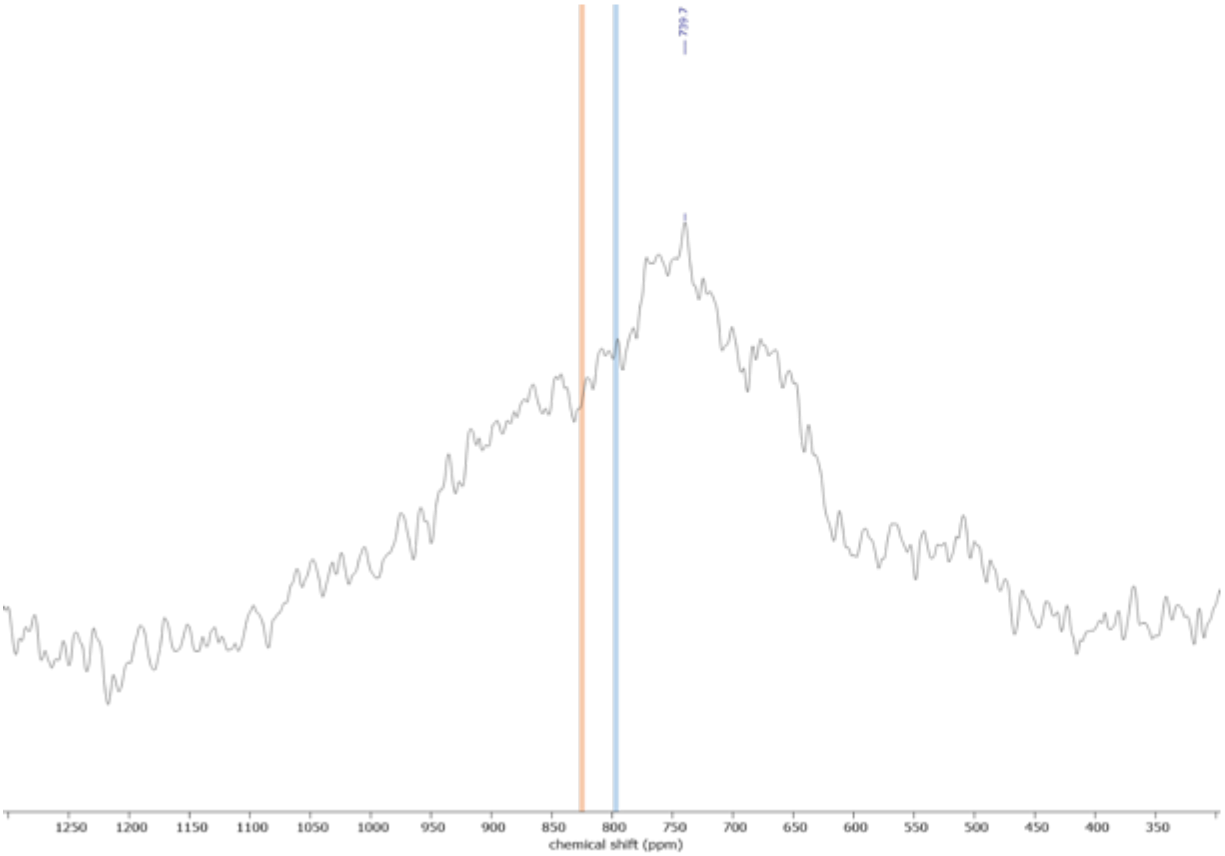

Figure S28. ^{77}Se MAS-NMR of cationic selenurane **[2c]SbF6**. *isotropic chemical shift.

*In the MAS-NMR spectrum of **[2c]SbF6** a broad signal centered at 740 ppm was identified. The signals for cationic Se(III) species previously reported by Furukawa are highlighted for clarity: a) at 806.5 ppm for Furukawa's dication **I** (blue line), and b) at 830.0 ppm for Furukawa's dicationic selenurane **II** (orange line).*[22],[23]

^{77}Se MAS-NMR of cationic selenurane [2d]SbF$_6$

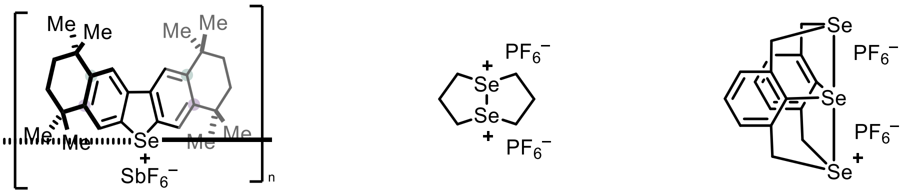

[2d] SbF$_6$

Furukawa dication
I

Furukawa dicationic selenurane
II

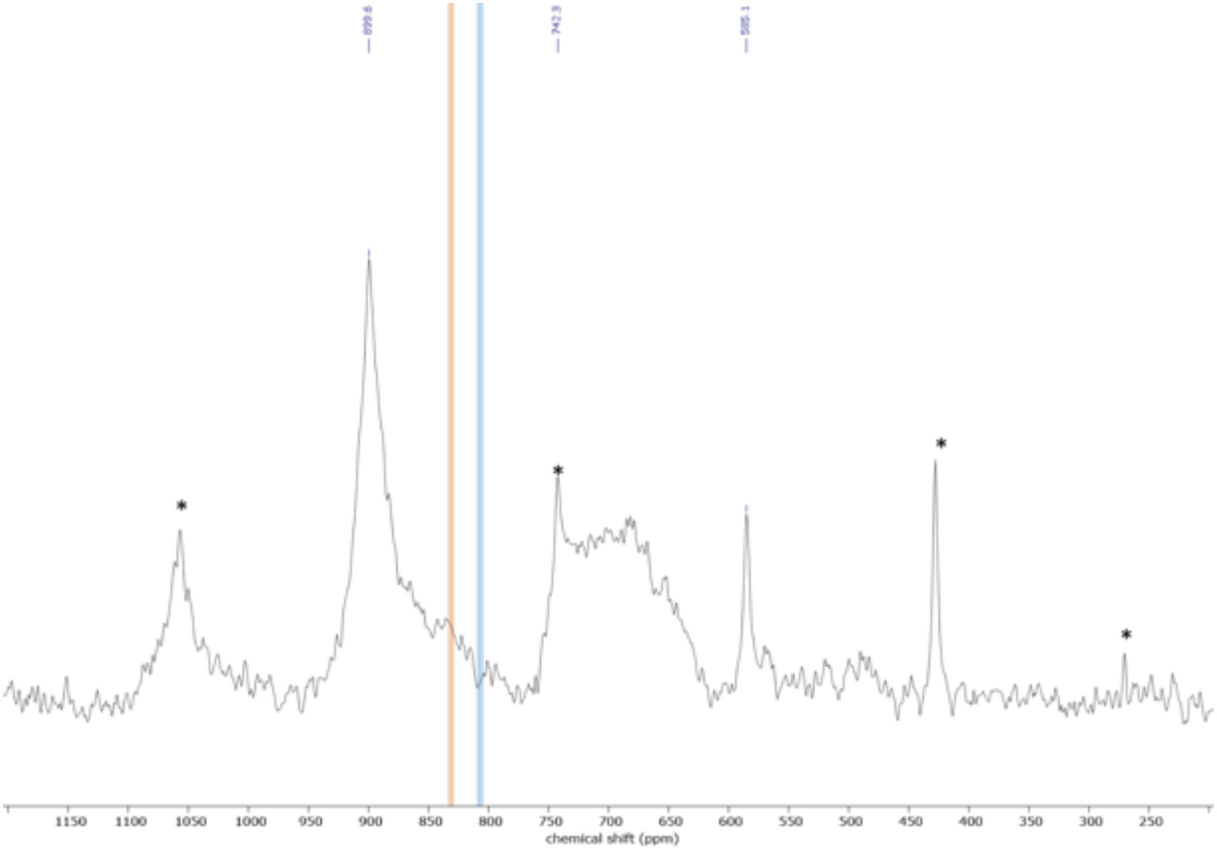

Figure S29. ^{77}Se MAS-NMR of cationic selenurane **[2d]SbF$_6$**. *isotropic chemical shift.

*In the MAS-NMR spectrum of **[2d]SbF$_6$** several (three) signals at the 900 to 560 ppm region (900 ppm, broad signal at ca. 700 ppm, and 586 ppm) were identified. The signals for cationic Se(III) species previously reported by Furukawa are highlighted for clarity: a) at 806.5 ppm for Furukawa's dication **I** (blue line), and b) at 830.0 ppm for Furukawa's dicationic selenurane **II** (orange line).[22],[23]*

^{77}Se MAS-NMR of [2,5'-bidibenzoselenophen]-5'-ium trifluoromethanesulfonate (Se–[5a]OTf)

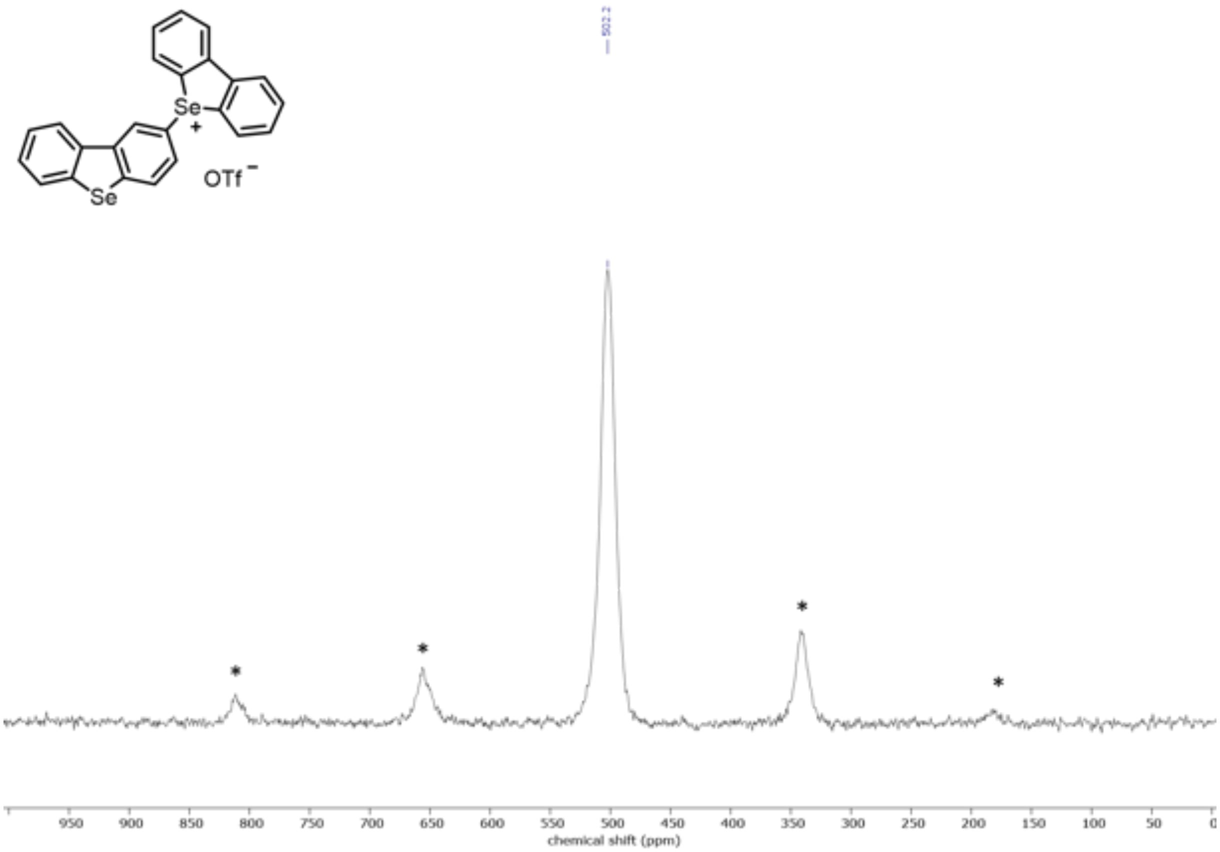

Figure S30.^{77}Se MAS-NMR of **Se–[5a]OTf**. *isotropic chemical shift.

Se–[5a]OTf *was used for comparison with cationic selenuranes* ***2a-d****. In the MAS-NMR spectrum of* **Se–[5a]OTf** *a single “sharp” signal centered at 502 ppm was identified (average of Se(IV) and Se(II) signal in solution), contrasting with the usual broad signals centered at the 900 to 700 ppm region observed with cationic selenuranes* ***2a-d****.*

^{77}Se MAS-NMR of 5-(phenyl) -dibenzoselenophenium hexafluoroantimonate (11)

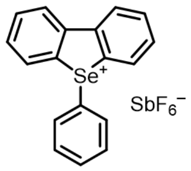

11

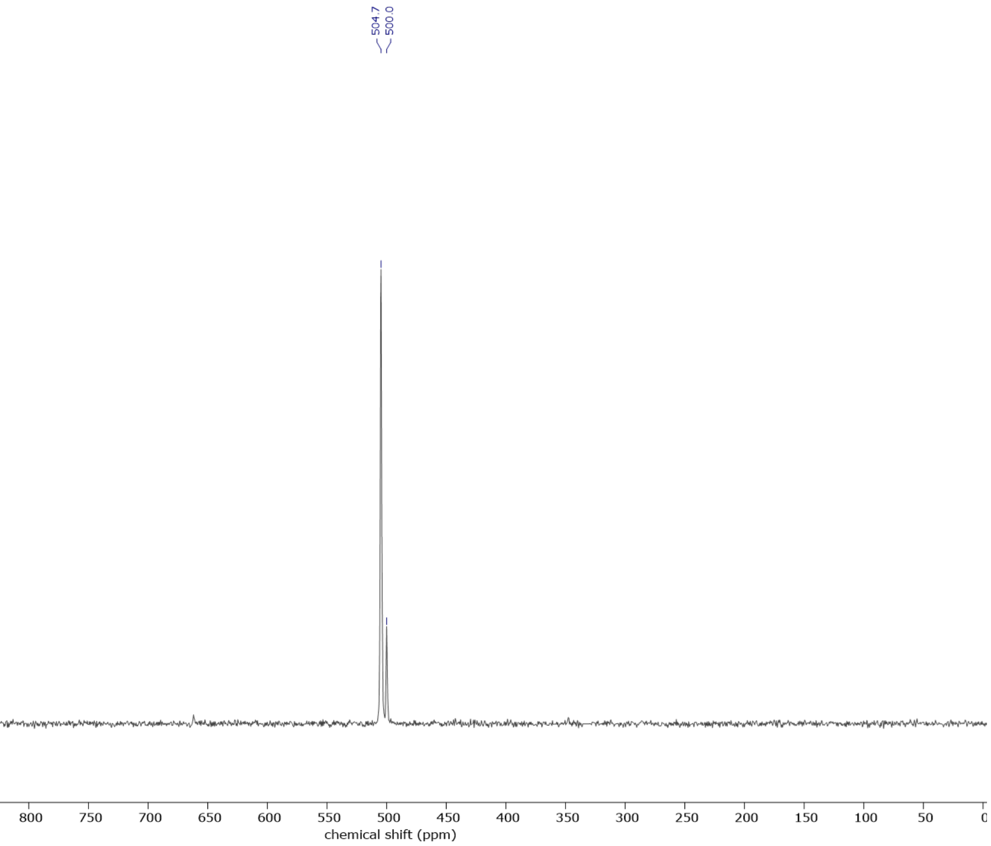

Figure S31. ^{77}Se MAS-NMR of **11**. *isotropic chemical shift.

11 *was used for comparison with cationic selenuranes* ***2a-d****. In the MAS-NMR spectrum of* **11** *two "sharp" signals at 505 and 500 ppm were identified (benchmarked with the common Se(IV) signal in solution), contrasting with the usual broad signals centered at the 900 to 700 ppm region observed with cationic selenuranes* ***2a-d****.*

NMR SPECTROSCOPY OF CATIONIC SELENURANES IN SOLUTION (^{1}H, ^{13}C, ^{19}F, AND ^{77}Se)

This section intends to give highlight the paramagnetic properties of cationic selenuranes in solution. For this reason, different NMR active nuclei were measured.

Samples contain solutions of:

a) freeze-pump-thawed CD_2Cl_2 cationic selenuranes **[2a]SbF_6**, **[2a]PF_6**, **[2a]BF_4**, and **[2b]SbF_6** prepared inside of a glovebox using J-Young NMR tubes; and

b) non-degassed CD_2Cl_2 cationic selenuranes **[2c]SbF_6** and **[2d]SbF_6** prepared under an ambient atmosphere.

^{1}H NMR of radical cation [4a]SbF_6 – *i.e.* [2a]SbF_6 sample dissolved in CD_2Cl_2

in solid state *in solution*

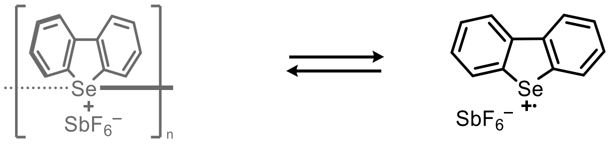

[2a] SbF_6 [4a] SbF_6

paramagnetic

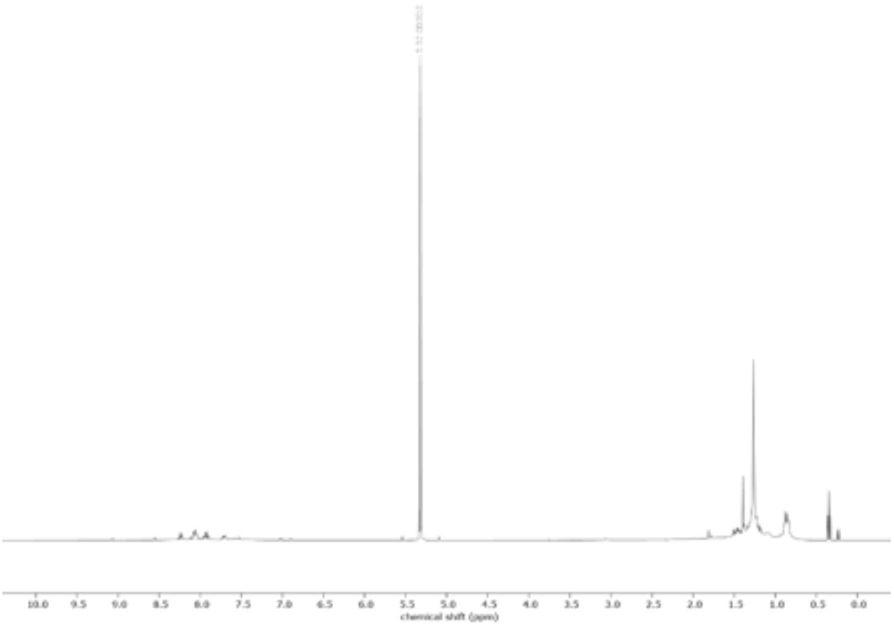

Figure S32. ^{1}H NMR spectrum of radical cation **[4a]SbF_6** in CD_2Cl_2.

*In the ^{1}H NMR spectrum of **[4a]SbF_6** in CD_2Cl_2 (99.6% purity) only signals of non-deuterated solvent, minor impurities (traces when compared with the 0.4% residual non-deuterated solvent) of dibenzo[b,d]selenophene (**1a**), and aliphatic products were identified. A signal broadening at the aromatic region is observed suggesting the formation of paramagnetic species.*

^{19}F NMR of radical cation [4a]SbF_6 – *i.e.* [2a]SbF_6 sample dissolved in CD_2Cl_2

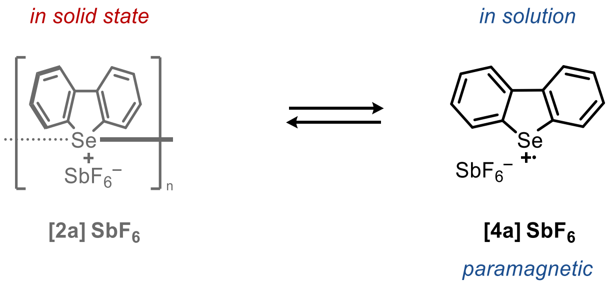

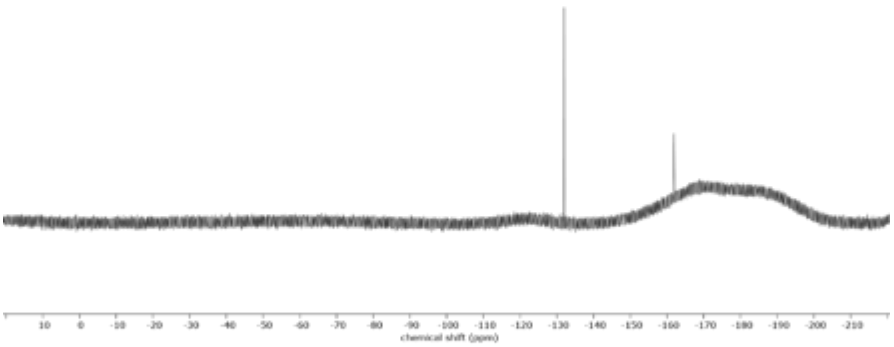

Figure S33. ^{19}F NMR spectrum of radical cation **[4a]SbF₆** in CD_2Cl_2.

Due to the low solubility of the cationic selenurane ***[2a]SbF₆*** in CD_2Cl_2, *^{13}C and ^{77}Se NMR spectra were not measured.*

^{1}H NMR of radical cation [4a]PF_6 – *i.e.* [2a]PF_6 sample dissolved in CD_2Cl_2

in solid state *in solution*

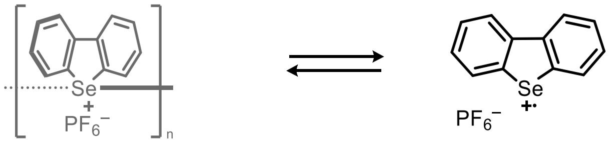

[2a] PF_6

[4a] PF_6
paramagnetic

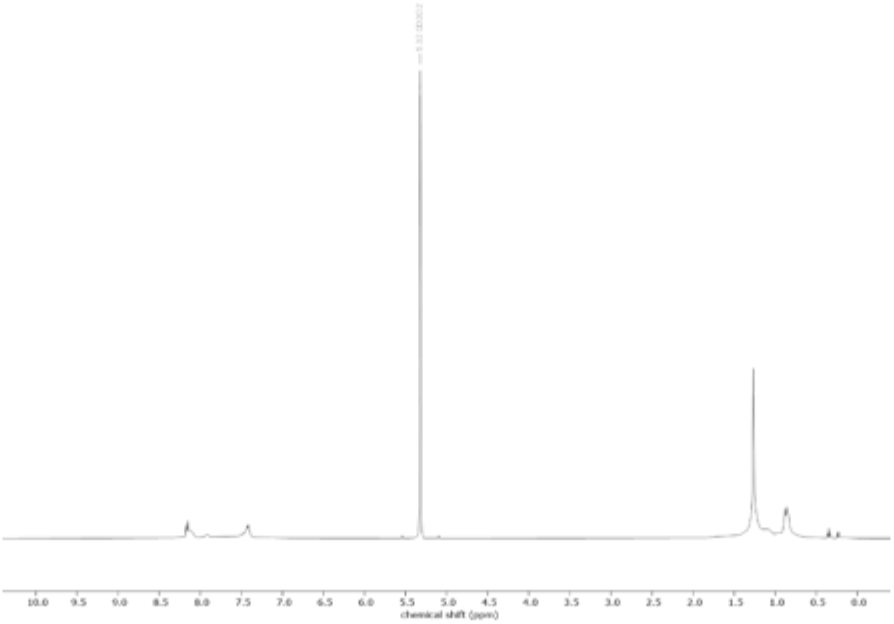

Figure S34. ^{1}H NMR spectrum of radical cation **[4a]PF_6** in CD_2Cl_2.

*In the ^{1}H NMR spectrum of **[4a]PF_6** in CD_2Cl_2 (99.6% purity) only signals of non-deuterated solvent, minor impurities (traces when compared with the 0.4% residual non-deuterated solvent) of dibenzo[b,d]selenophene (**1a**), and aliphatic products were identified. A signal broadening at the aromatic region is observed suggesting the formation of paramagnetic species.*

^{19}F NMR of radical cation [4a]PF_6 – *i.e.* [2a]PF_6 sample dissolved in CD_2Cl_2

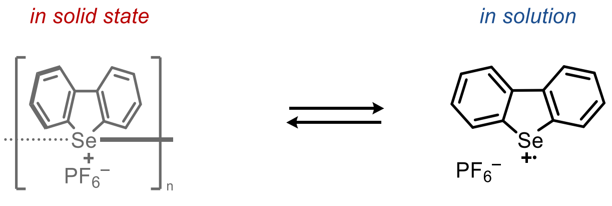

[2a] PF_6

[4a] PF_6
paramagnetic

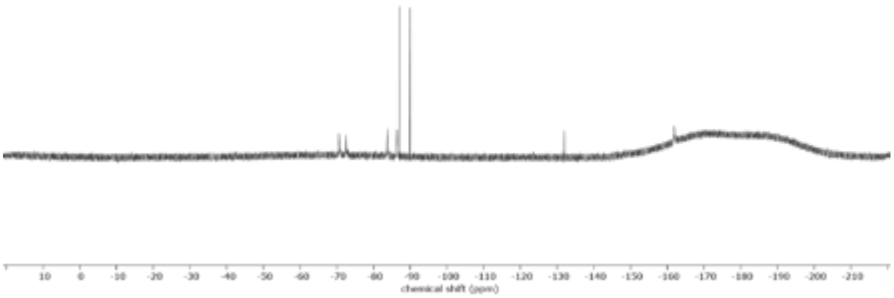

Figure S35. ^{19}F NMR spectrum of radical cation **[4a]PF_6** in CD_2Cl_2.

*Due to the low solubility of the cationic selenurane **[2a]PF_6**, ^{13}C and ^{77}Se NMR spectra were not measured.*

^{1}H NMR of radical cation [4a]BF_4 – *i.e.* [2a]BF_4 sample dissolved in CD_2Cl_2

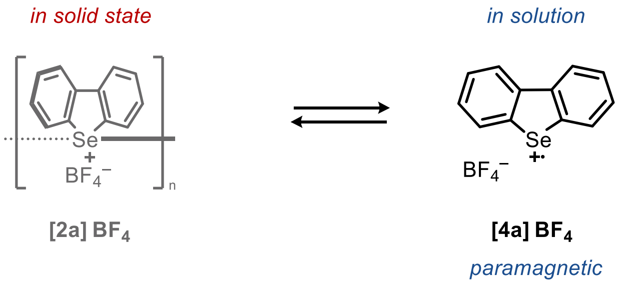

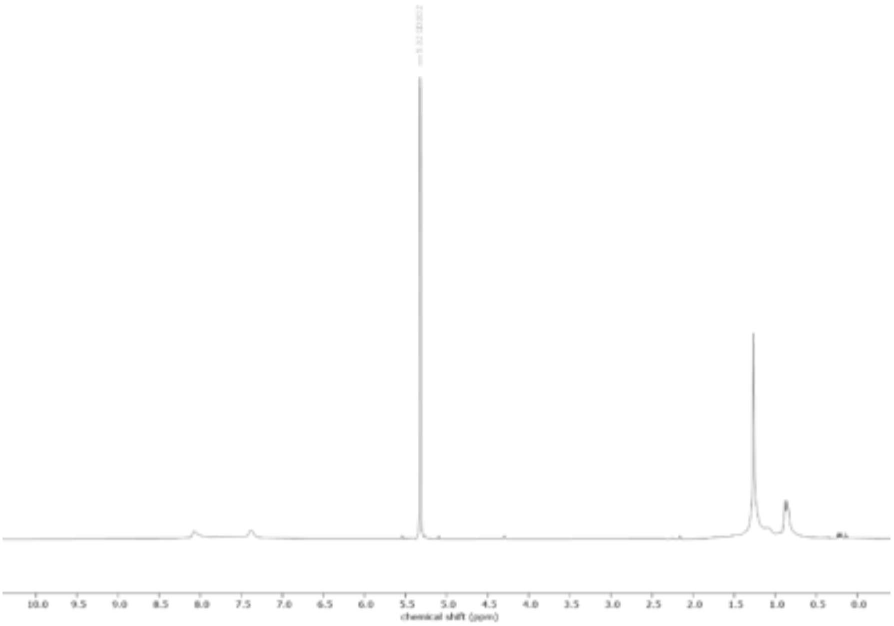

Figure S36. ^{1}H NMR spectrum of radical cation **[4a]BF$_4$** in CD_2Cl_2.

*In the ^{1}H NMR spectrum of **[4a]BF$_4$** in CD_2Cl_2 (99.6% purity) only signals of non-deuterated solvent, minor impurities (traces when compared with the 0.4% residual non-deuterated solvent) of dibenzo[b,d]selenophene (**1a**), and aliphatic products were identified. A signal broadening at the aromatic region is observed suggesting the formation of paramagnetic species.*

^{19}F NMR of radical cation [4a]BF_4 – *i.e.* [2a]BF_4 sample dissolved in CD_2Cl_2

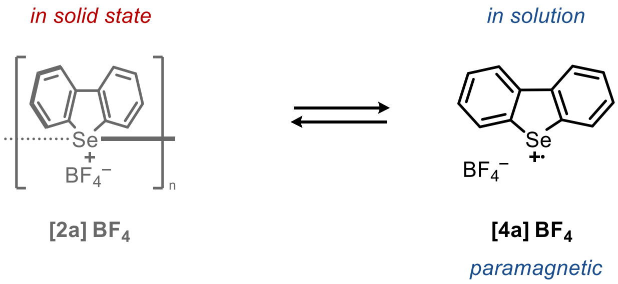

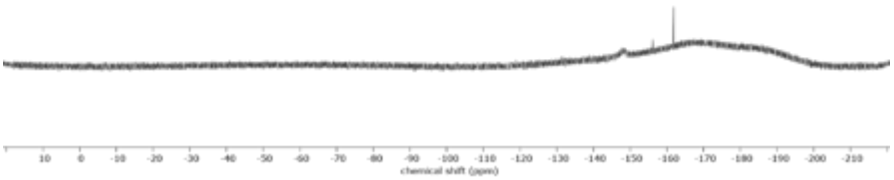

Figure S37. ^{19}F NMR spectrum of radical cation **[4a]BF₄** in CD_2Cl_2.

Due to the low solubility of the cationic selenurane ***[2a]BF₄****,* ^{13}C *and* ^{77}Se *NMR spectra were not measured.*

^{1}H NMR of radical cation [4b]SbF_6 – *i.e.* [2b]SbF_6 sample dissolved in CD_2Cl_2

in solid state | *in solution*

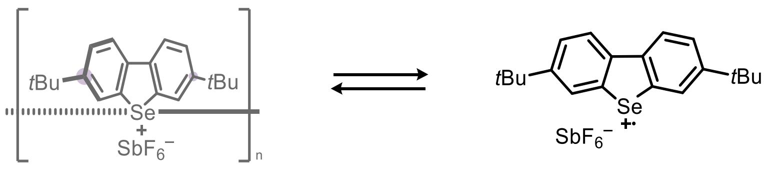

[2b] SbF_6 | **[4b] SbF_6** *paramagnetic*

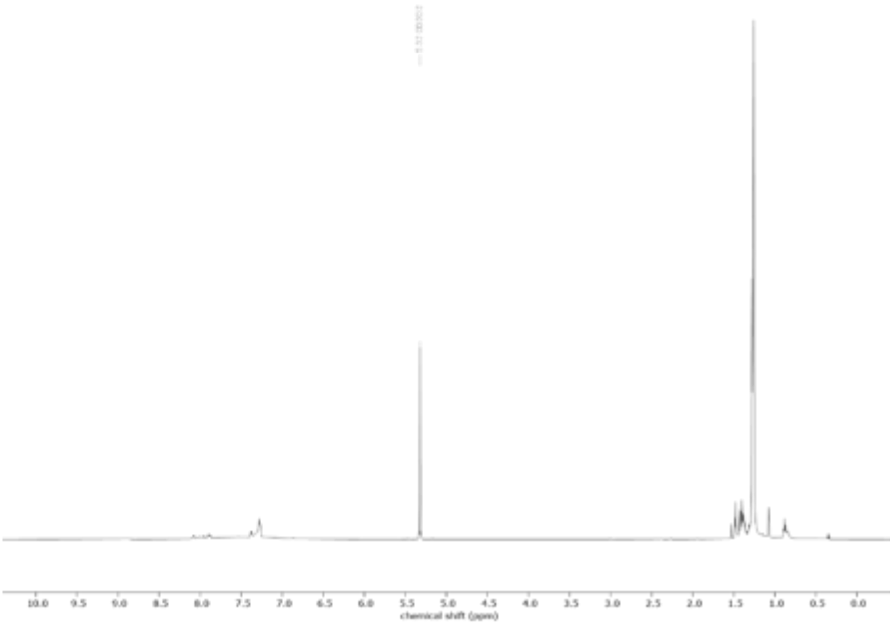

Figure S38. ^{1}H NMR spectrum of radical cation **[4b]SbF_6** in CD_2Cl_2.

*In the ^{1}H NMR spectrum of **[4b]SbF_6** in CD_2Cl_2 (99.6% purity) only signals of non-deuterated solvent, minor impurities (traces when compared with the 0.4% residual non-deuterated solvent), and broad signals in the range from 8.5 to 7.0 ppm and at 1.3 ppm were identified. The signal broadening suggests the formation of paramagnetic species.*

^{13}C NMR of radical cation [4b]SbF_6 – *i.e.* [2b]SbF_6 sample dissolved in CD_2Cl_2

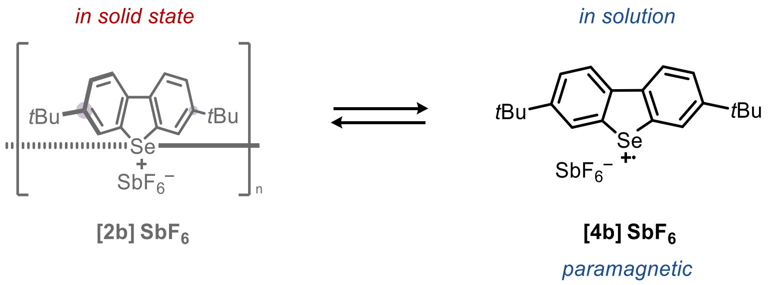

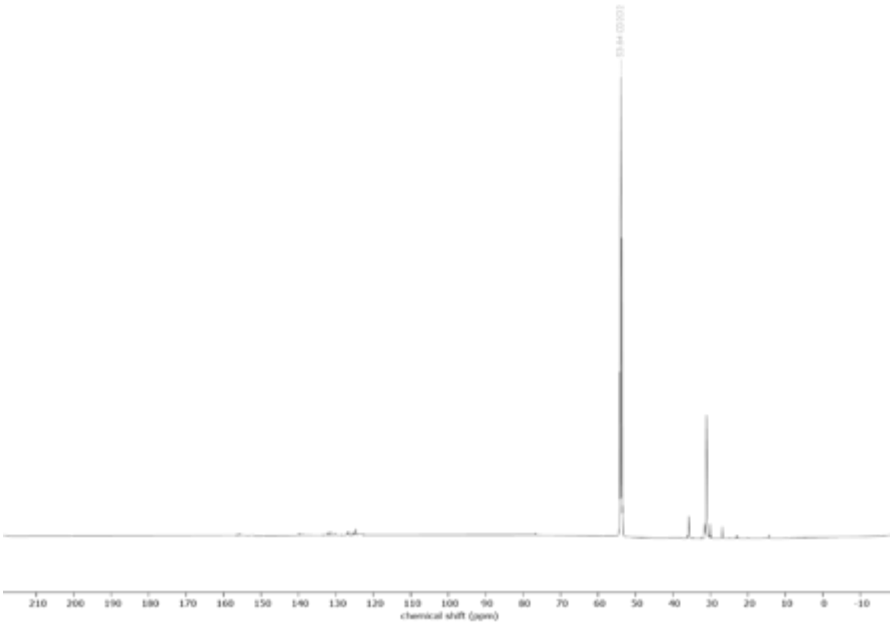

Figure S39. ^{13}C NMR spectrum of radical cation **[4b]SbF_6** in CD_2Cl_2.

*In the ^{13}C NMR spectrum of **[4b]SbF_6** in CD_2Cl_2 (99.6% purity) the signal of the solvent, broad signals in the range from 160 to 120 ppm, and a broad signal at 30 ppm were identified.*

^{19}F NMR of radical cation [4b]SbF_6 – *i.e.* [2b]SbF_6 sample dissolved in CD_2Cl_2

in solid state *in solution*

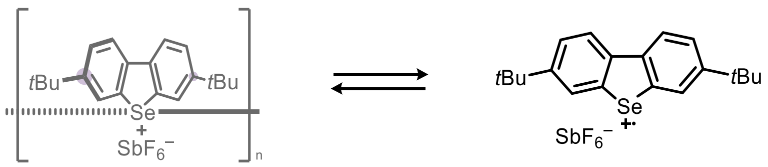

[2b] SbF_6 **[4b] SbF_6**
paramagnetic

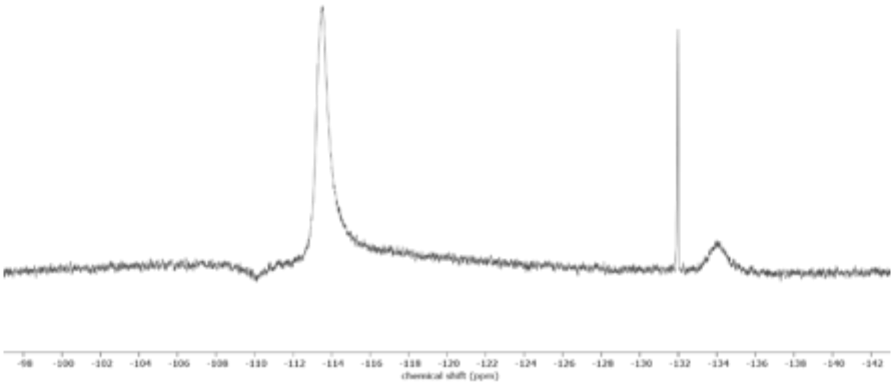

Figure S40. ^{19}F NMR spectrum of radical cation **[4b]SbF_6** in CD_2Cl_2.

*In the ^{19}F NMR spectrum of **[4b]SbF_6** a broad signal at –114 ppm, a signal at –132 ppm, and a broad signal at –134 ppm were identified.*

^{77}Se NMR of radical cation [4b]SbF_6 – *i.e.* [2b]SbF_6 sample dissolved in CD_2Cl_2

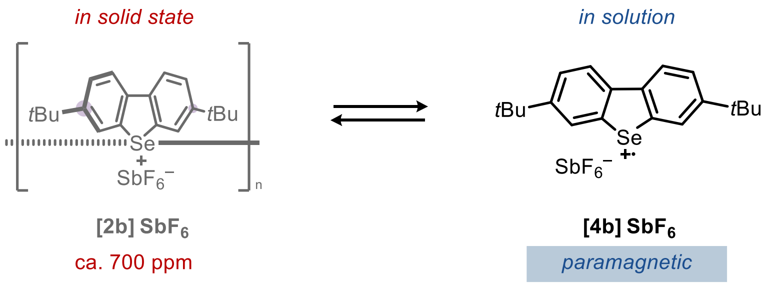

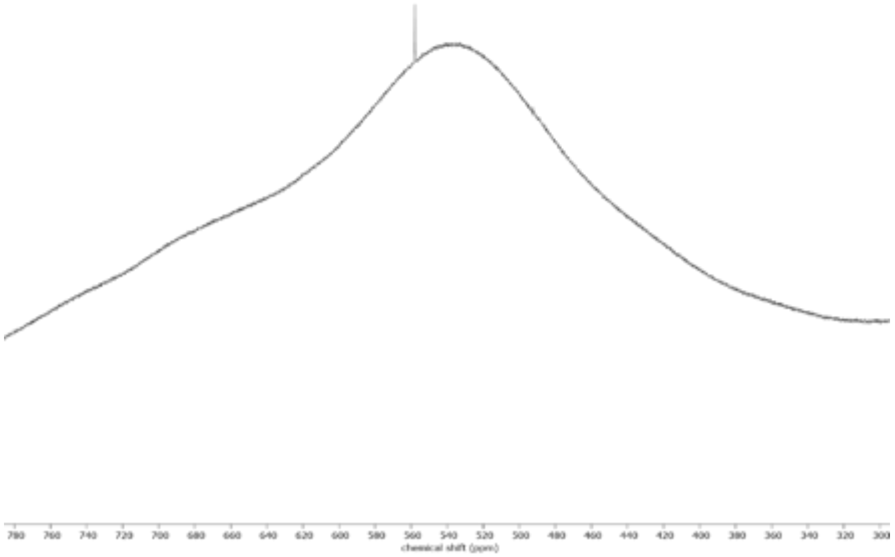

Figure S41. ^{77}Se NMR spectrum of radical cation **[4b]SbF_6** in CD_2Cl_2.

*In the ^{77}Se NMR spectrum of **[4b]SbF_6** a broad signal (300 ppm) in the range from 400 to 700 ppm was identified. The signal broadening suggests the formation of paramagnetic species.*

^1H NMR of radical cation [4c]SbF$_6$ – *i.e.* [2c]SbF$_6$ sample dissolved in CD_2Cl_2

in solid state *in solution*

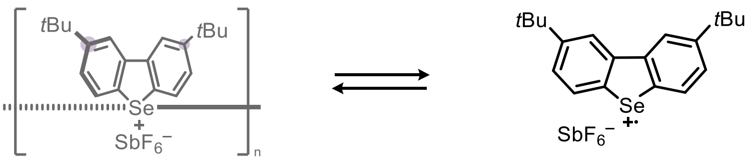

[2c] SbF$_6$ **[4c] SbF$_6$** *paramagnetic*

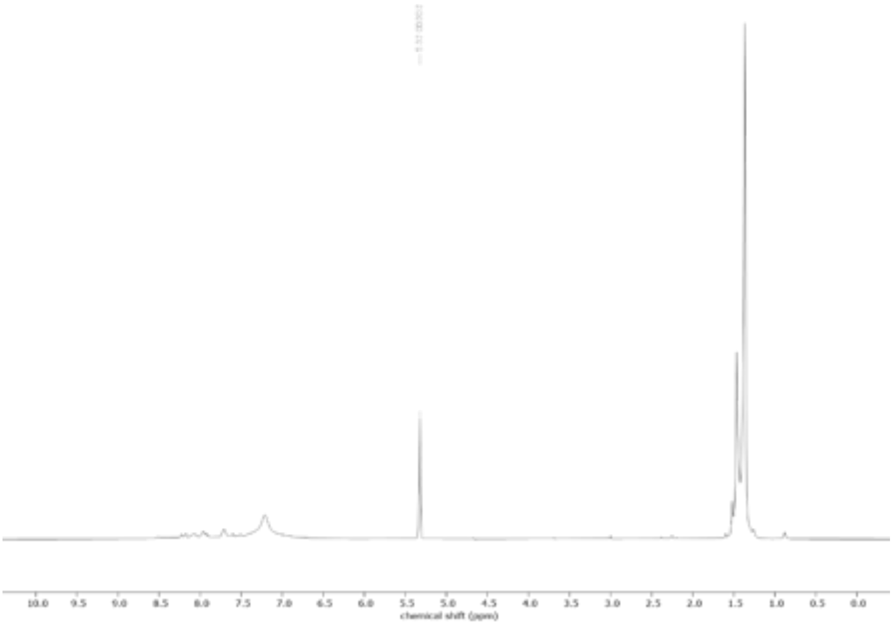

Figure S42. ^1H NMR spectrum of radical cation **[4c]SbF$_6$** in CD_2Cl_2.

*In the ^1H NMR spectrum of **[4c]SbF$_6$** in CD_2Cl_2 (99.6% purity) only signals of non-deuterated solvent, minor impurities (traces when compared with the 0.4% residual non-deuterated solvent), and broad signals in the range from 8.5 to 7.0 ppm and at 1.5 ppm were identified. The signal broadening suggests the formation of paramagnetic species.*

^{13}C NMR of radical cation [4c]SbF_6 – *i.e.* [2c]SbF_6 sample dissolved in CD_2Cl_2

in solid state — *in solution*

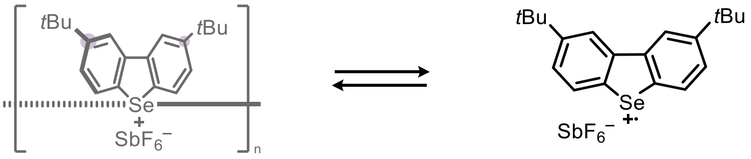

[2c] SbF_6 — **[4c] SbF_6** *paramagnetic*

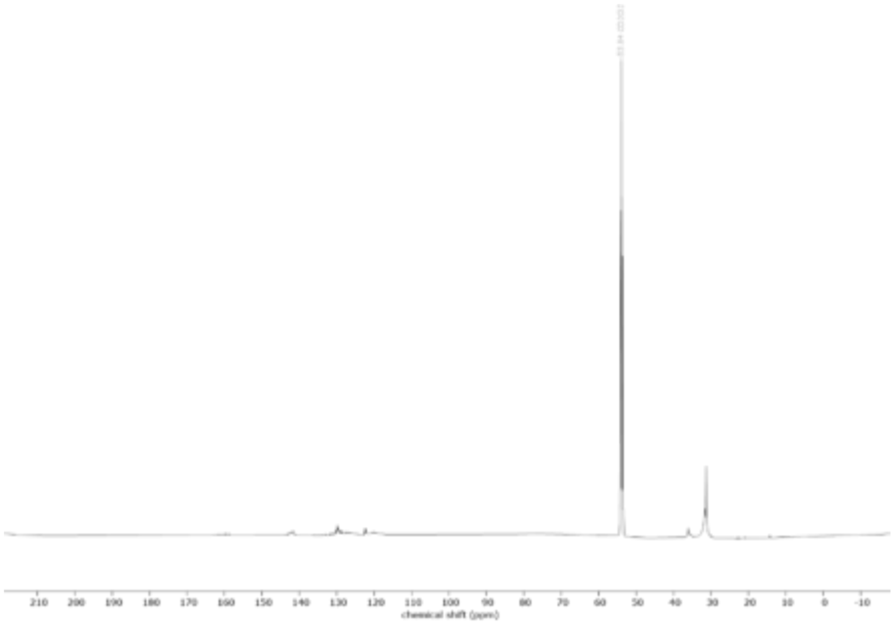

Figure S43. ^{13}C NMR spectrum of radical cation **[4c]SbF_6** in CD_2Cl_2.

*In the ^{13}C NMR spectrum of **[4c]SbF_6** in CD_2Cl_2 (99.6% purity) a signal solvent, broad signals in the range from 160 to 120 ppm, and a broad signal at 30 ppm were identified.*

^{19}F NMR of radical cation [4c]SbF_6 – *i.e.* [2c]SbF_6 sample dissolved in CD_2Cl_2

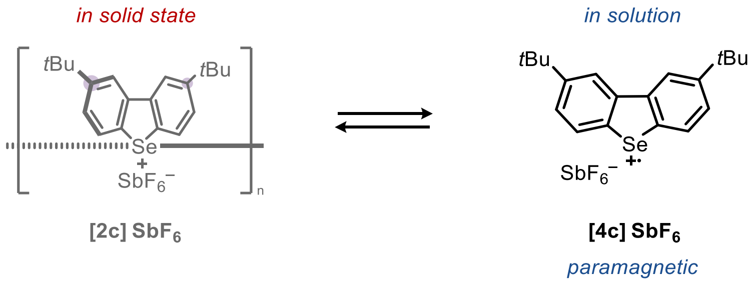

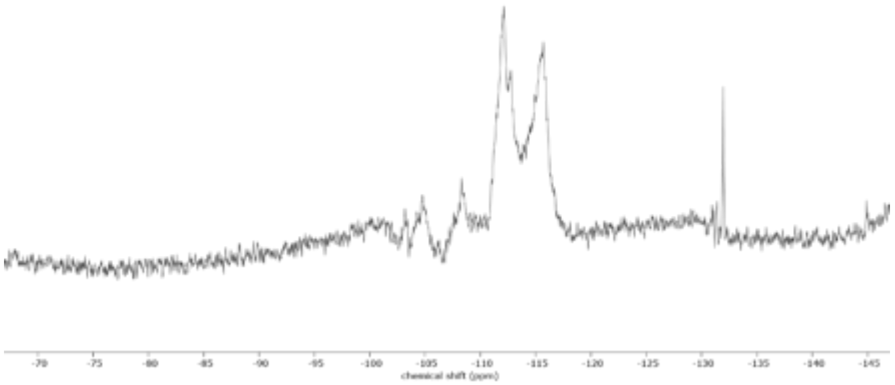

Figure S44. ^{19}F NMR spectrum of radical cation **[4c]SbF_6** in CD_2Cl_2.

*In the ^{19}F NMR spectrum of **[4c]SbF_6** series of broad signals in the range from –100 to –120 ppm and signal at –132 ppm were identified.*

^{77}Se NMR of radical cation [4c]SbF_6 – *i.e.* [2c]SbF_6 sample dissolved in CD_2Cl_2

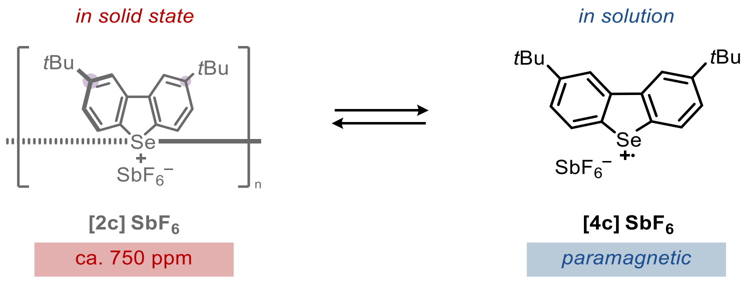

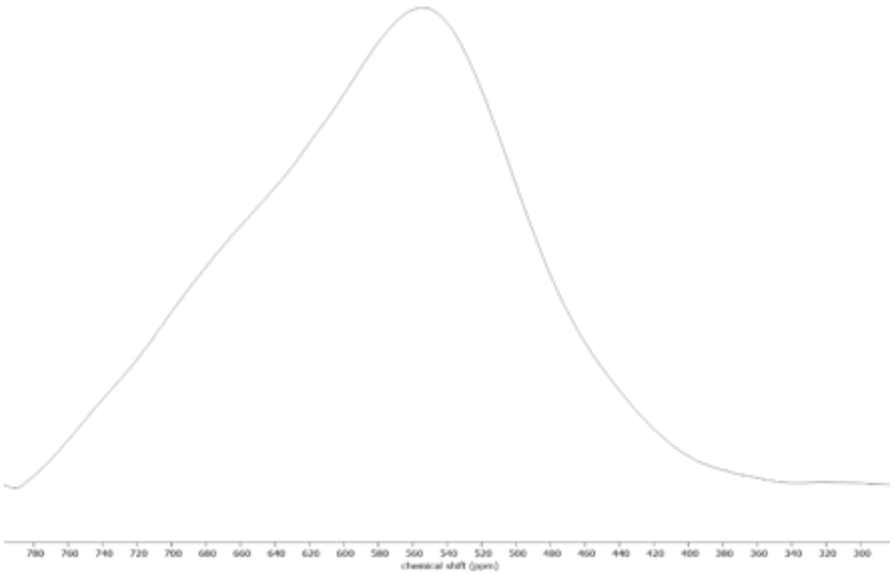

Figure S45. ^{77}Se NMR spectrum of radical cation **[4c]SbF_6** in CD_2Cl_2.

*In the ^{77}Se NMR spectrum of **[4c]SbF_6** broad signal in the range from 400 to 780 ppm was identified. The signal broadening suggests the formation of paramagnetic species.*

^{1}H NMR of radical cation [4d]SbF_6 – *i.e.* [2d]SbF_6 sample dissolved in CD_2Cl_2

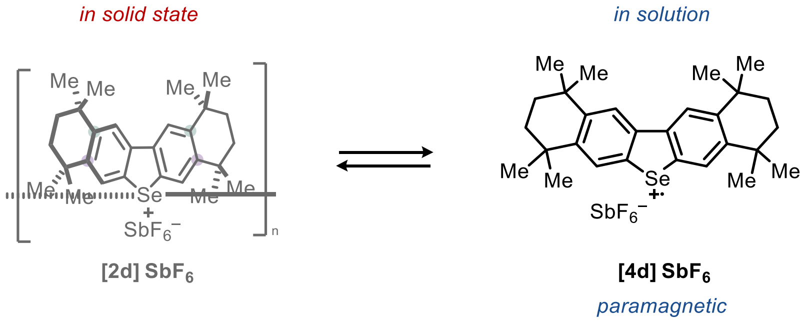

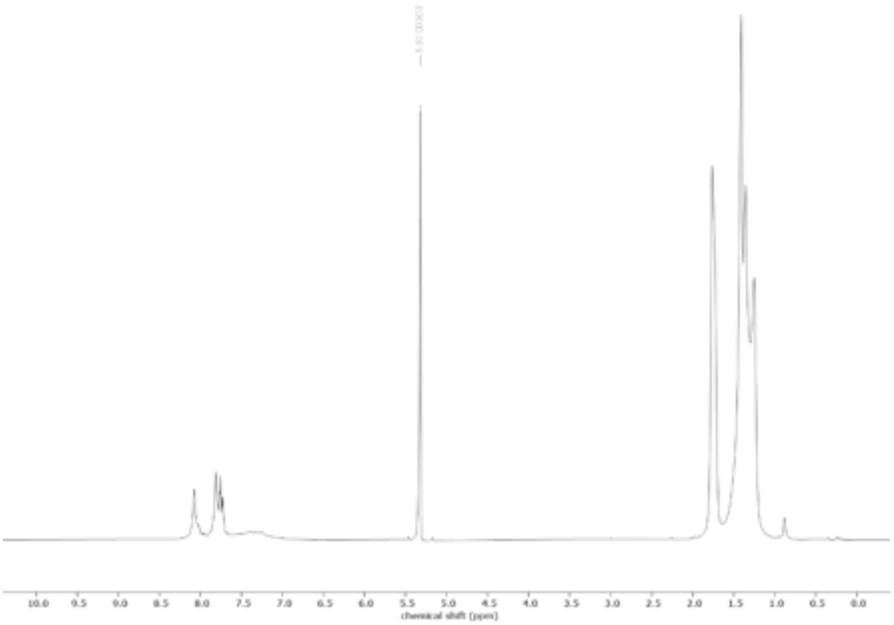

Figure S46.^{1}H NMR spectrum of radical cation **[4d]SbF_6** in CD_2Cl_2.

*In the ^{1}H NMR spectrum of **[4c]SbF_6** in CD_2Cl_2 (99.6% purity) only signals of non-deuterated solvent, minor impurities (traces when compared with the 0.4% residual non-deuterated solvent), and broad signals in the range from 8.3 to 7.0 ppm and at 2.0 to 1.0 ppm were identified. The signal broadening suggests the formation of paramagnetic species.*

^{13}C NMR of radical cation [4d]SbF_6 – *i.e.* [2d]SbF_6 sample dissolved in CD_2Cl_2

in solid state *in solution*

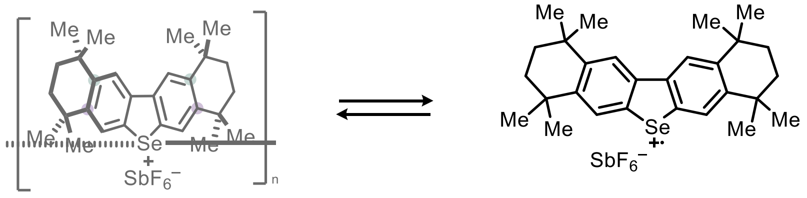

[2d] SbF_6 **[4d] SbF_6**

paramagnetic

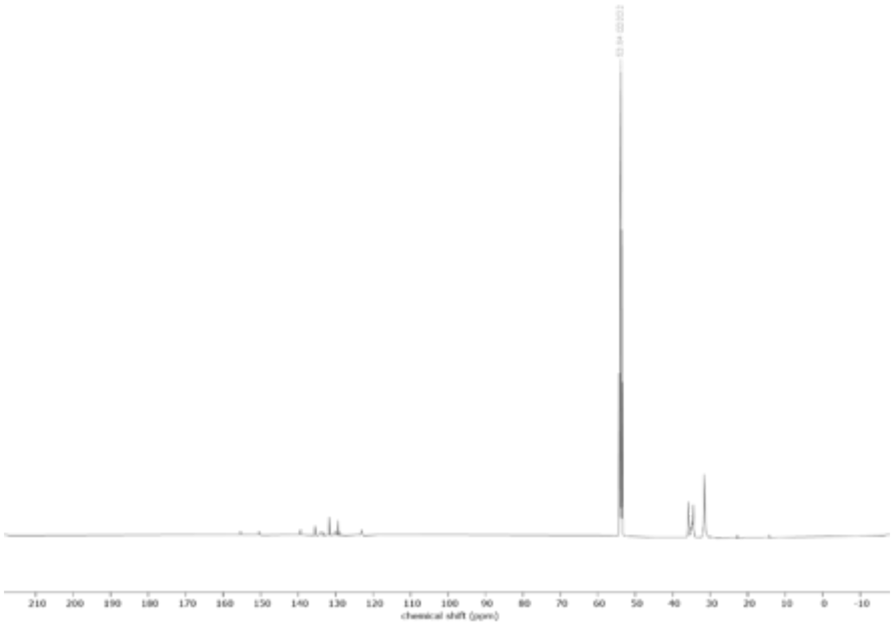

Figure S47. ^{13}C NMR spectrum of radical cation **[4d]SbF_6** in CD_2Cl_2.

*In the ^{13}C NMR spectrum of **[4d]SbF_6** in CD_2Cl_2 (99.6% purity) a signal of the solvent, broad signals in the range from 160 to 120 ppm, and broad signals in the range from 40 to 30 ppm were identified*

^{19}F NMR of radical cation [4d]SbF$_6$ – *i.e.* [2d]SbF$_6$ sample dissolved in CD_2Cl_2

in solid state *in solution*

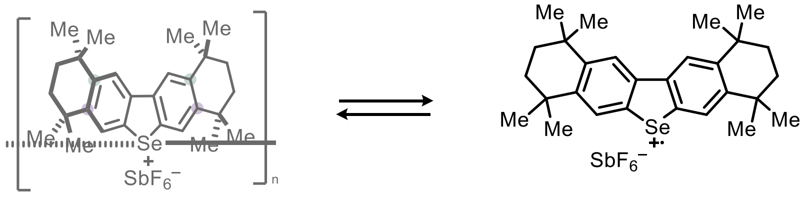

[2d] SbF$_6$ **[4d] SbF$_6$**

paramagnetic

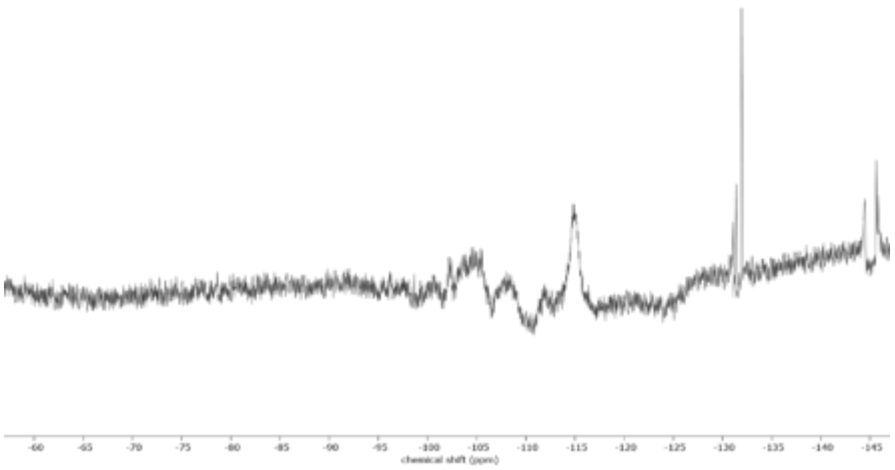

Figure S48. ^{19}F NMR spectrum of radical cation **[4d]SbF$_6$** in CD_2Cl_2.

*In the ^{19}F NMR spectrum of **[4d]SbF$_6$** series of broad signals in the range from –100 to –120 ppm and signal at –132 ppm were identified.*

^{77}Se NMR of radical cation [4d]SbF_6 – *i.e.* [2d]SbF_6 sample dissolved in CD_2Cl_2

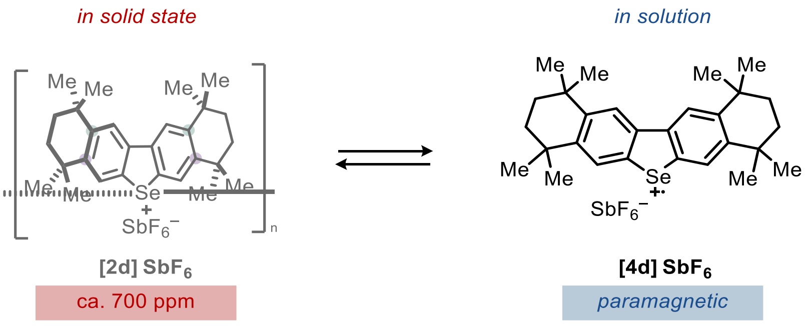

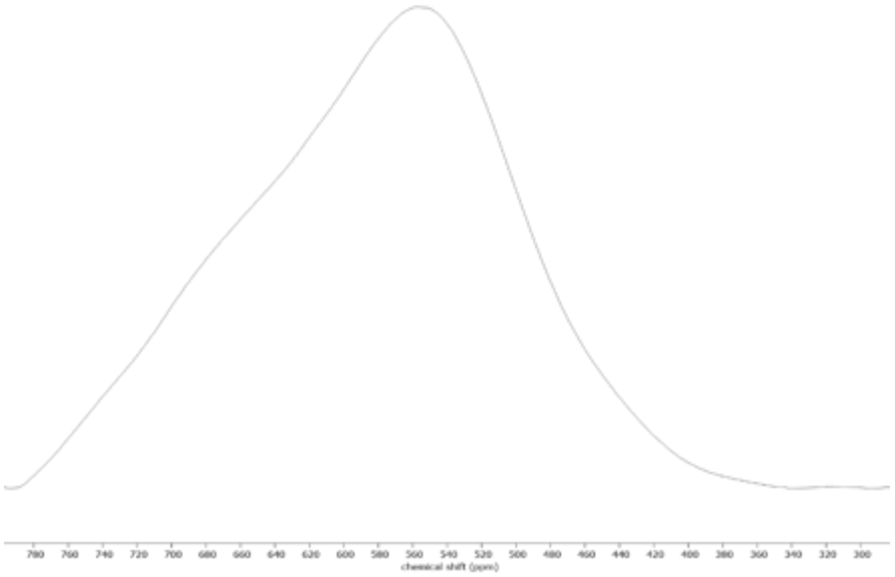

Figure S49. ^{77}Se NMR spectrum of radical cation **[4d]SbF₆** in CD_2Cl_2.

In the ^{77}Se NMR spectrum of ***[4d]SbF₆*** *a broad signal in the range from 400 to 780 ppm was identified. The signal broadening suggests the formation of paramagnetic species.*

VT-NMR EXPERIMENTS – OLIGOMERIZATION EQUILIBRIA

This section intends to give additional information on the equilibrium process between the oligomeric form of dibenzoselnophenium radical cations **4** and its oligomeric forms **2**. For this reason, variable temperature ^{1}H-NMR spectra of cationic selenurane **[2c]SbF₆** was measured at 7 different temperatures (from 298 K to 238 K).

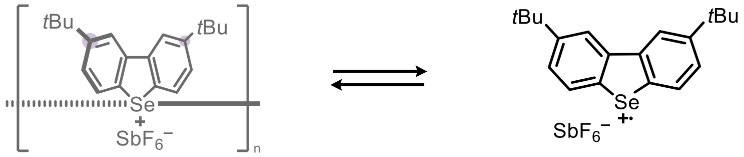

[2c] SbF_6 [4c] SbF_6

For the VT-NMR spectroscopy experiments at different temperatures, a sample of cationic selenurane **[2c]SbF_6** was weighed inside the glovebox, 18.0 mg of **[2c]SbF_6** (0.031 mmol) were introduced in a J-Young NMR tube, dissolved in 0.5 mL of CD_2Cl_2 [*c* = 0.06 M], and sealed inside of the glovebox. The sample was analyzed via ^{1}H NMR spectroscopy (700 MHz) at different temperatures. The residual non-deuterated solvent signal was used to reference the spectra. At 298 K, radical cation **4c** was observed as a broad (paramagnetic) signal at the aromatic region (7.36–7.20 ppm). At 238 K, the broad signal resolved into four different broad signals (Figure S50 and S51).

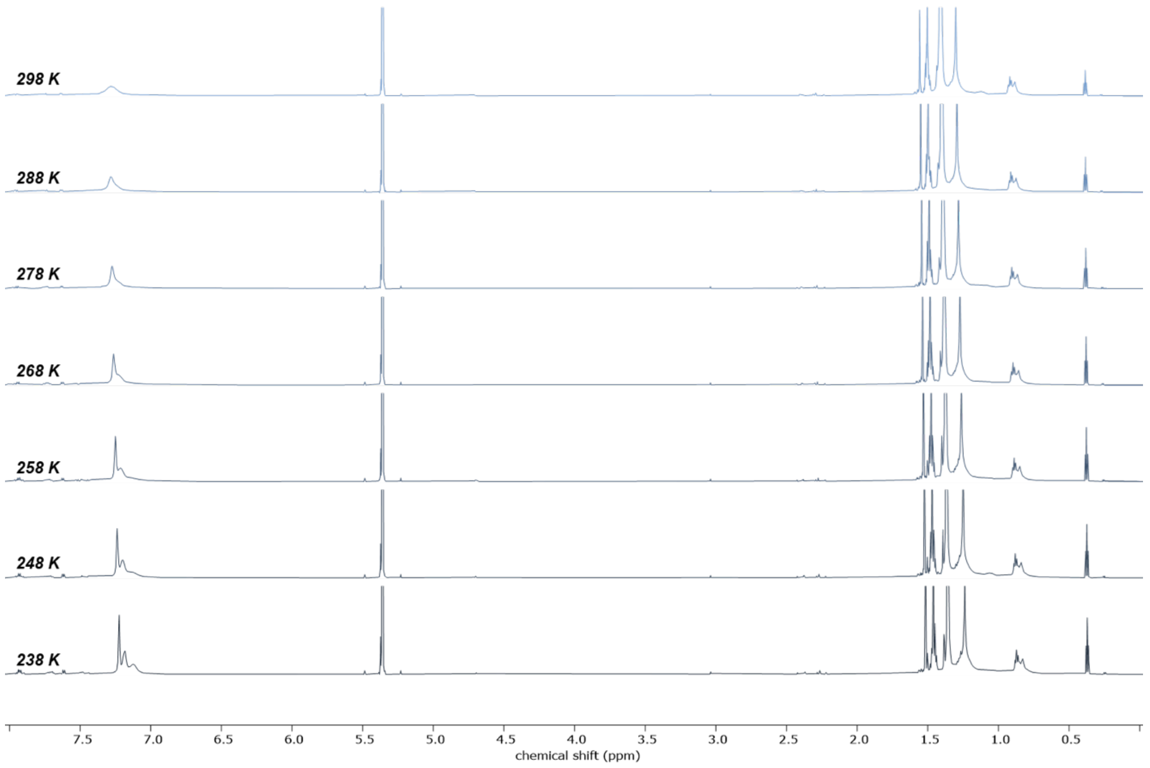

Figure S50. ^{1}H NMR spectra of a solution of **[2c]SbF_6** / **[4c]SbF_6** in CD_2Cl_2 from 298 K (top) to 238 K (bottom).

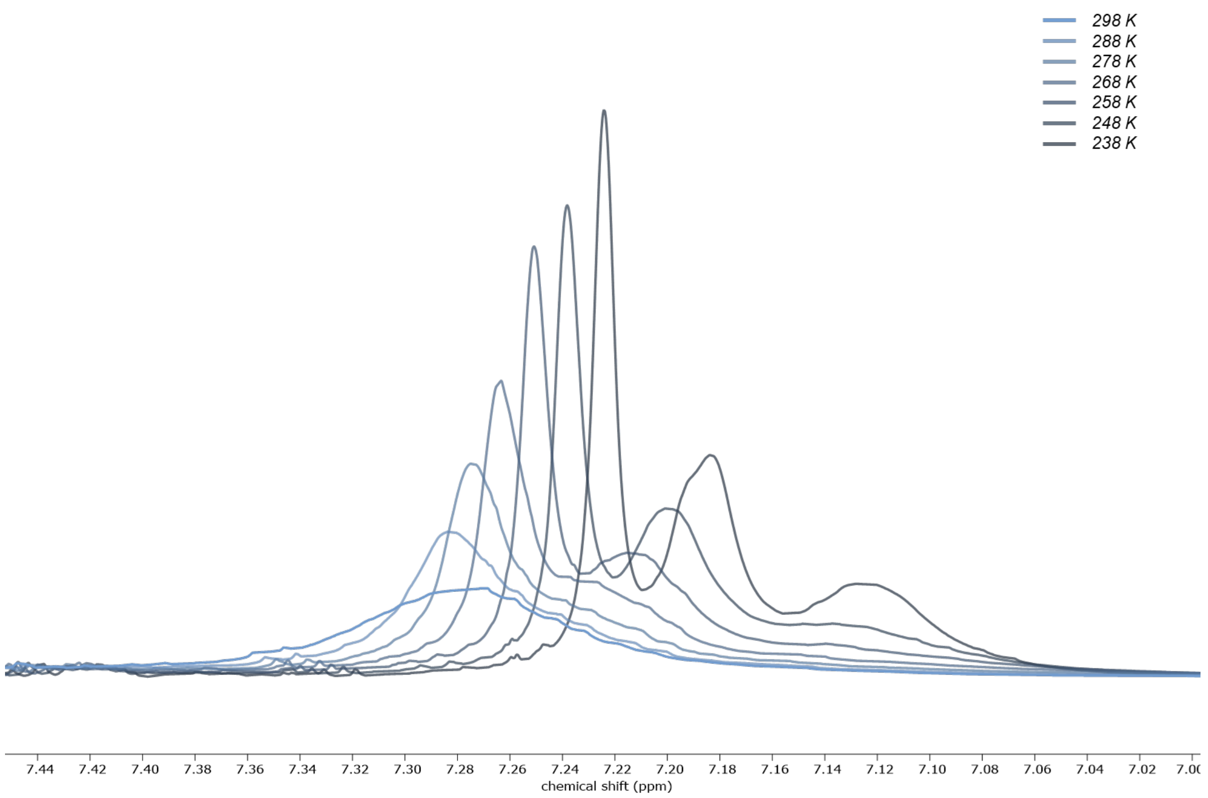

Figure S51. Overimposed aromatic region of the ^{1}H NMR spectra of a solution of **[2c]SbF$_6$** / **[4c]SbF$_6$** in CD_2Cl_2 from 298 K (light blue) to 238 K (dark grey).

Description of these results: *At 298 K, the equilibrium between* **[2c]SbF$_6$** and **[4c]SbF$_6$** results in a single broad aromatic signal, indicating coalescence of the distinct cationic selenurane and radical cation resonances into one signal in the 7.36–7.20 ppm region. Upon cooling, the signal sharpens and resolves into up to four distinct peaks at 238 K, accompanied by a downfield shift to a broad signal centered at 7.12 ppm.

Interpretation: *The coalescence of aromatic signals in cationic selenurane samples indicates an exchange process between* ***[2c]SbF$_6$*** *and* ***[4c]SbF$_6$****, consistent with a dynamic equilibrium in solution.*

EPR SPECTROSCOPY – CHARACTERIZATION OF PARAMAGNETIC SPECIES

SAMPLE PREPARATION

In solid state: pre-weighted samples (in a glovebox) of cationic selenuranes (*ca.* 5.0 mg) were transferred to Wilmad® Suprasil EPR tubes. The EPR tubes were capped and sealed to avoid exposure to oxygen and water, removed from the glovebox immediately before the experiment, and measured at 298 K.

In solution: pre-weighted samples (in a glovebox) of cationic selenuranes were dissolved in dry and degassed DCM. The 5.0 mM solutions were transferred to Norell® Quartz EPR tubes, capped and sealed to avoid exposure to oxygen and water. The EPR tubes were removed from the glovebox immediately before the experiment and measured at 298 K.

Solid-state EPR of [2a]SbF_6 at 298K

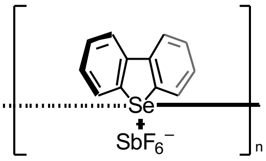

[2a]SbF_6

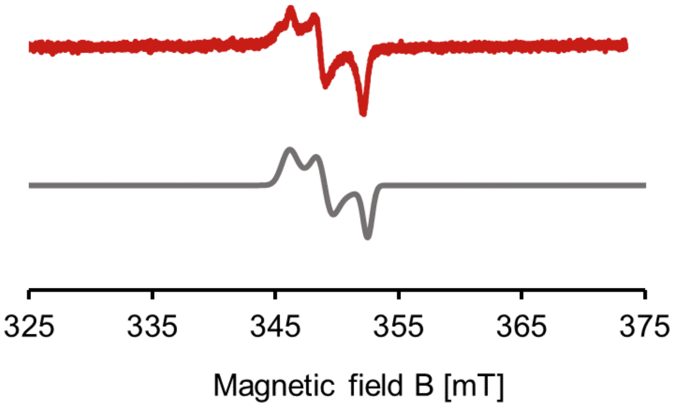

Figure S52. Experimental (top) and simulated (bottom) X-Band CW-EPR spectrum of **[2a]SbF_6** at 298K. The obtained signal is depicted in red and the Easyspin® simulation in grey ($\mathbf{g_x}$ = 2.0295, $\mathbf{g_y}$ = 1.9944, $\mathbf{g_z}$ = 2.0134).

Solution-phase EPR of [4a]SbF_6 in CH_2Cl_2 at 298K

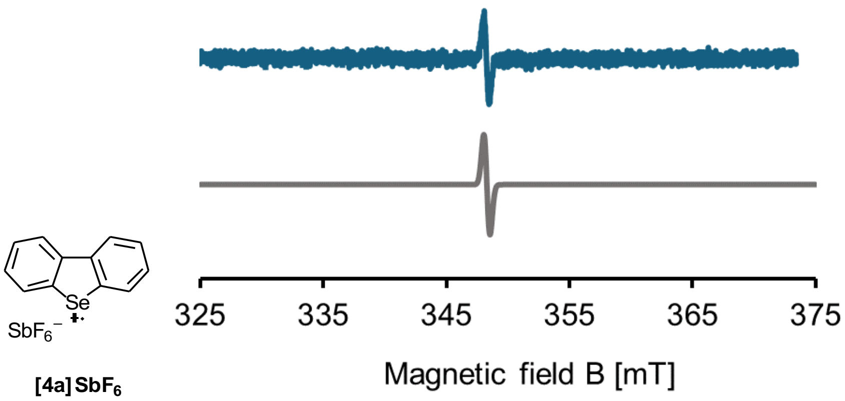

Figure S53. Experimental (top) and simulated (bottom) X-Band CW-EPR spectrum **[4a]SbF_6** in CH_2Cl_2 at 298K. The obtained signal is depicted in blue and the Easyspin® simulation in grey.

Solid-state vs solution-phase EPR at 298K – [2a]SbF_6 vs [4a]SbF_6

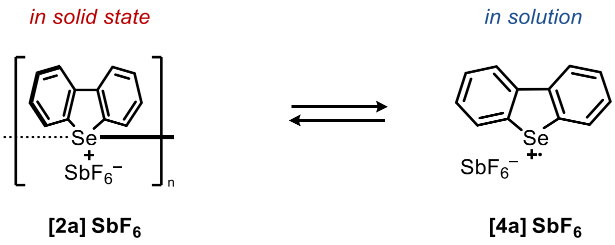

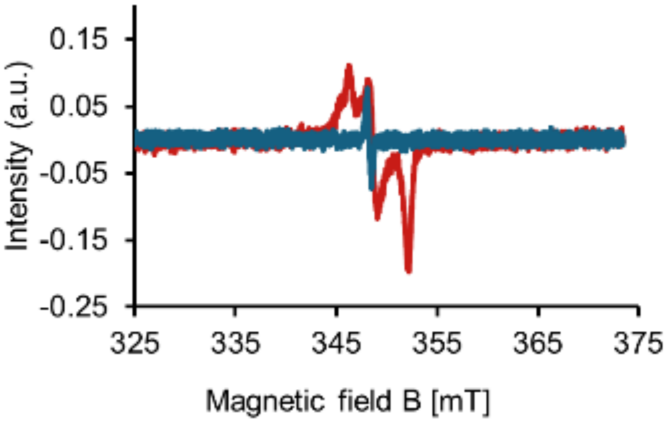

Figure S54. Comparison between solid-state and solution-phase X-Band CW-EPR spectra. **Red:** solid-state, **Blue:** solution-phase.

Solid-state EPR of [2b]SbF_6 at 298K

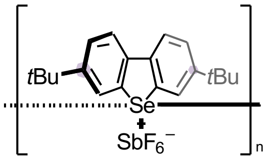

[2b]SbF_6

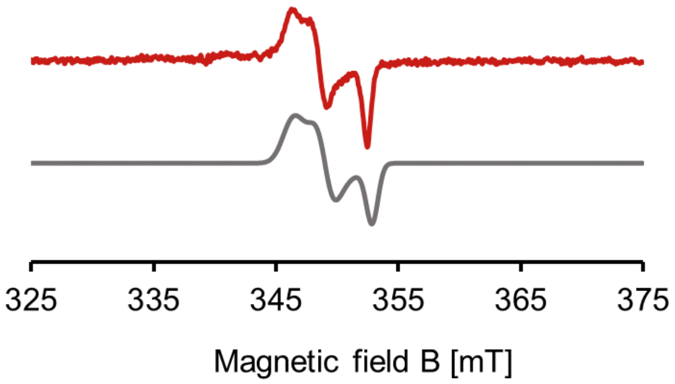

Figure S55. Experimental (top) and simulated (bottom) X-Band CW-EPR spectrum of **[2b]SbF_6** at 298K. The obtained signal is depicted in red and the Easyspin® simulation in grey ($\mathbf{g_x}$ = 2.0281, $\mathbf{g_y}$ = 1.9924, $\mathbf{g_z}$ = 2.0134).

Solution-phase EPR of [4b]SbF_6 in CH_2Cl_2 at 298K

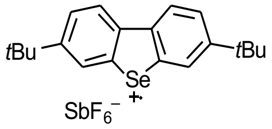

[4b] SbF_6

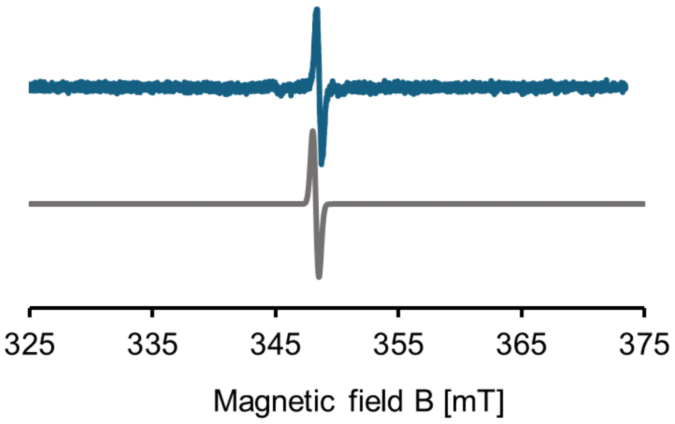

Figure S56. Experimental (top) and simulated (bottom) X-Band CW-EPR spectrum **[4b]SbF_6** in CH_2Cl_2 at 298K. The obtained signal is depicted in blue and the Easyspin® simulation in grey color.

Solid-state vs solution-phase EPR at 298K – [2b]SbF_6 vs [4b]SbF_6

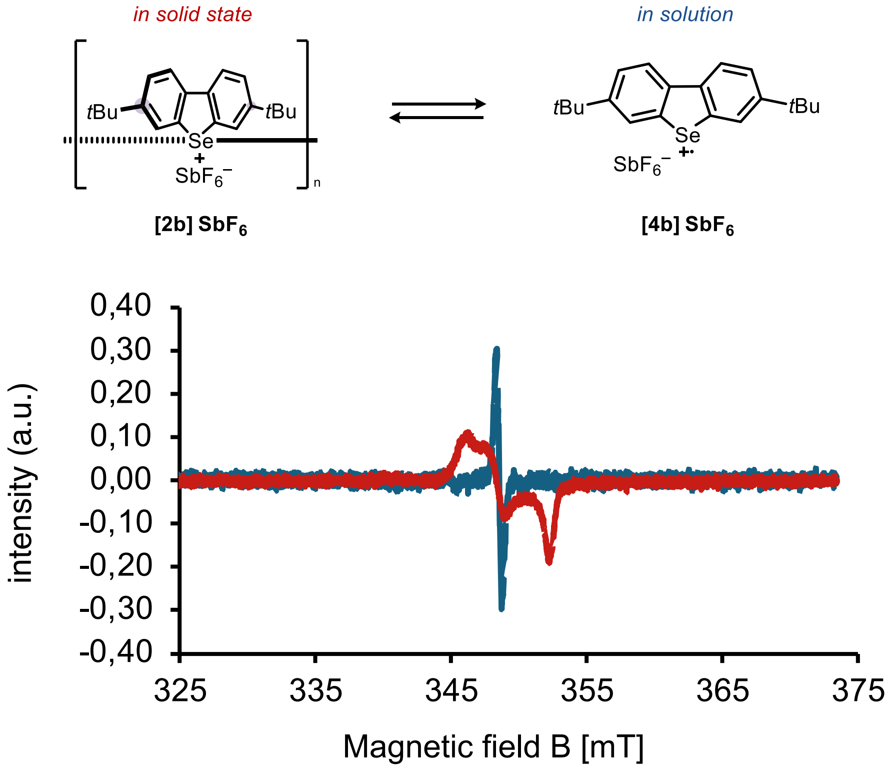

Figure S57. Comparison between solid-state and solution-phase X-Band CW-EPR spectra. **Red:** solid-state, **Blue:** solution-phase.

Solid-state EPR of [2c]SbF_6 at 298K

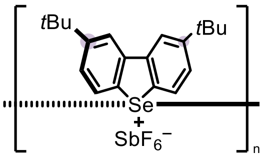

[2c] SbF₆

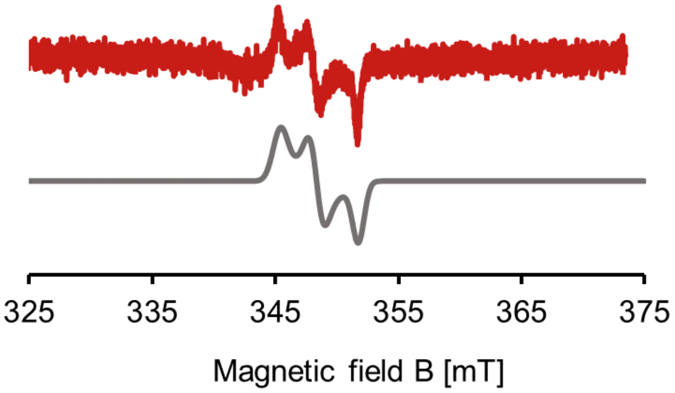

Figure S58. Experimental (top) and simulated (bottom) X-Band CW-EPR spectrum of **[2c]SbF$_6$** at 298K. The obtained signal is depicted in red and the Easyspin® simulation in grey (**g**$_x$ = 2.0333, **g**$_y$ = 1.9982, **g**$_z$ = 2.0168).

Solution-phase EPR of [4c]SbF_6 in CH_2Cl_2 at 298K

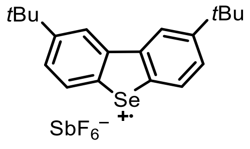

[4c] SbF₆

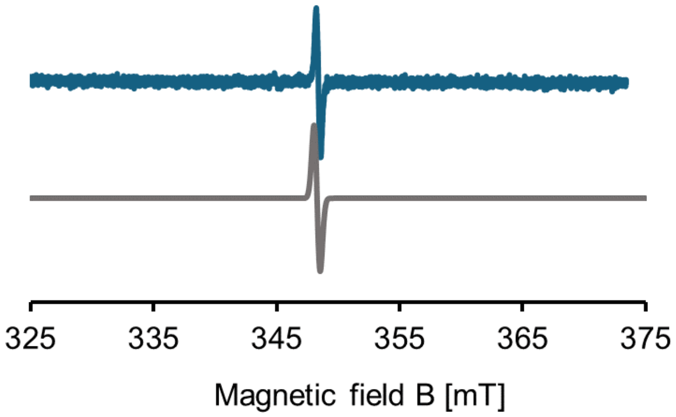

Figure S59. Experimental (top) and simulated (bottom) X-Band CW-EPR spectrum **[4c]SbF$_6$** in CH_2Cl_2 at 298K. The obtained signal is depicted in blue and the Easyspin®. The obtained signal is depicted in blue and the Easyspin® simulation in grey color.

Solid-state vs solution-phase EPR at 298K – [2c]SbF_6 vs [4c]SbF_6

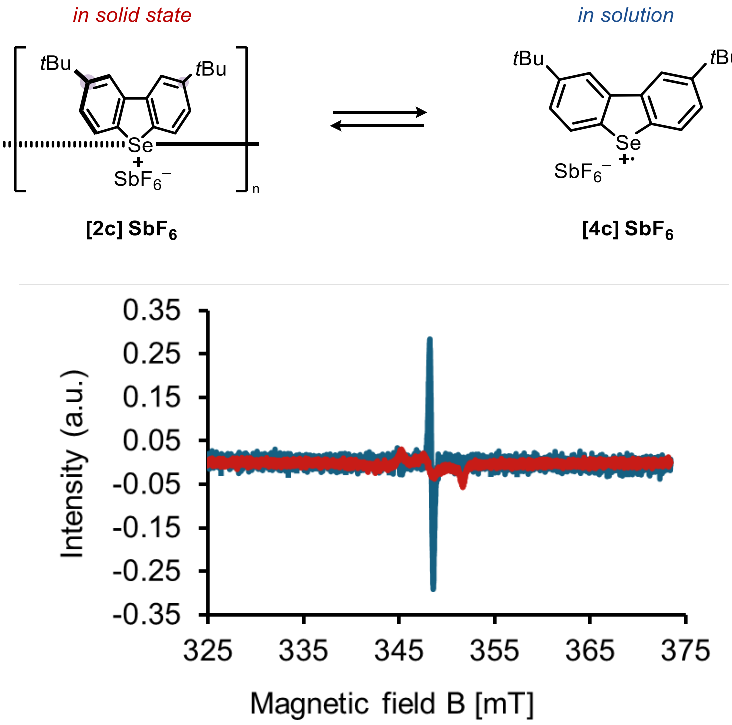

Figure S60. Comparison between solid-state and solution-phase X-Band CW-EPR spectra. Red: solid-state, Blue: solution-phase.

Solid-state EPR of [2d]SbF_6 at 298K

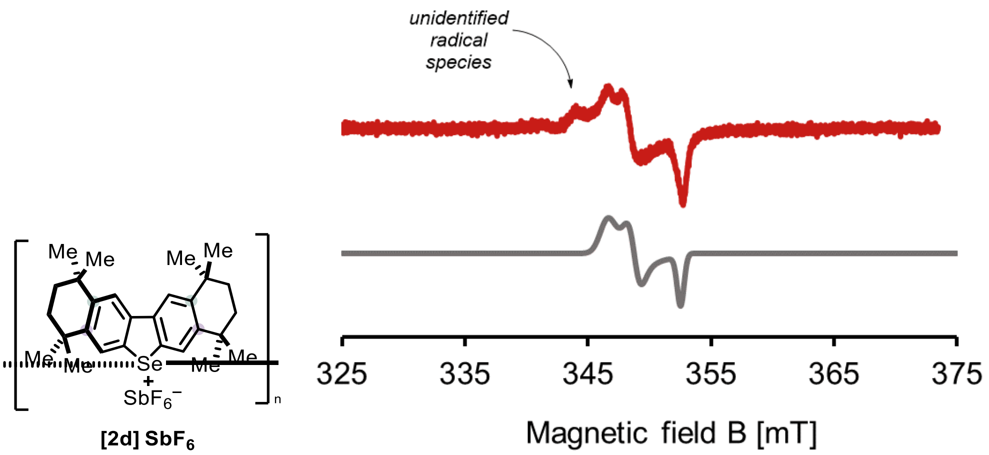

Figure S61. Experimental (top) and simulated (bottom) X-Band CW-EPR spectrum of **[2d]SbF_6** at 298K. The obtained signal is depicted in red and the Easyspin® simulation in grey (**g_x** = 2.0270, **g_y** = 1.9942, **g_z** = 2.0147).

Solution-phase EPR of [4d]SbF_6 in CH_2Cl_2 at 298K

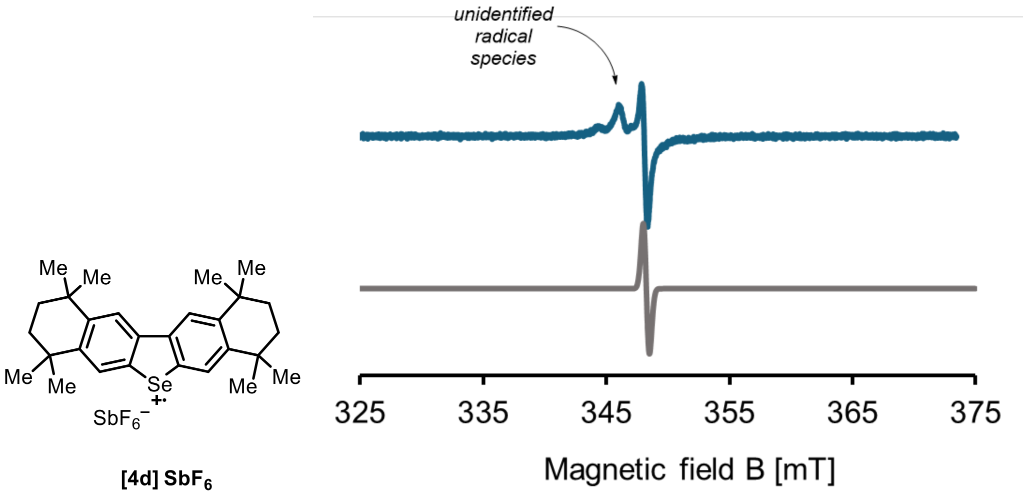

Figure S62. Experimental (top) and simulated (bottom) X-Band CW-EPR spectrum **[4d]SbF_6** in CH_2Cl_2 at 298K. The obtained signal is depicted in blue and the Easyspin®. The obtained signal is depicted in blue and the Easyspin® simulation in grey color.

Solid-state vs solution-phase EPR at 298K – [2d]SbF_6 vs [4d]SbF_6

in solid state *in solution*

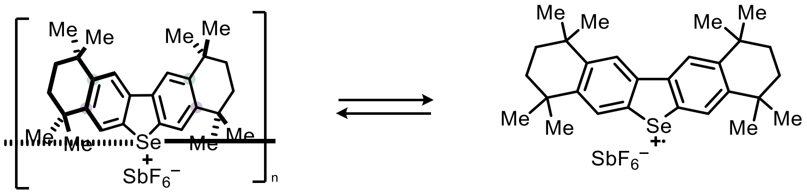

[2d] SbF_6 **[4d] SbF_6**

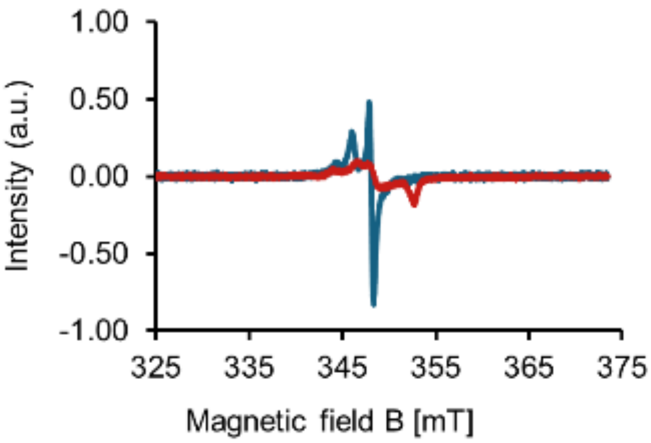

Figure S63. Comparison between solid-state and solution-phase X-Band CW-EPR spectra. Red: solid-state, Blue: solution-phase.

Cyclic Voltammetry

Sample Preparation

Dibenzoselenophenes **1a-d** (50.0 μmol), and NBu_4PF_6 (969 mg, 2.50 mmol) were dissolved in 5.0 mL of MeCN (**[1]** = 10.0 mM, **[NBu_4PF_6]** = 0.050 M). A glassy carbon disk working electrode and a platinum disk counter electrode were used. All potentials were measured versus an aqueous Ag/AgCl electrode with 3 M NaCl as electrolyte.

Oxidation of dibenzo[*b*,*d*]selenophene (1a)

Redox couple (1a/4a): E_p = +1533 mV vs. Ag/AgCl (= +1743 mV vs. standard hydrogen electrode).

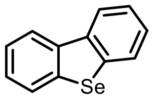

1a

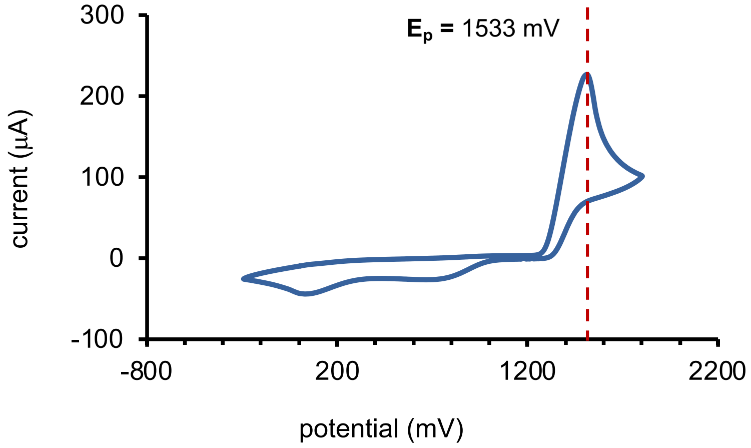

Figure S64. Anodic CV of **1a** in a 0.05 M NBu_4PF_6 MeCN solution. GC electrode. Scan rate: 100 mV/s, potential referred to Ag/AgCl at 298K using a platinum disk counter electrode as counter electrode.

Oxidation of 3,7-di-*tert*-butyldibenzo[*b*,*d*]selenophene (1b)

Redox couple (1b/4b): E_p = +1425 mV vs. Ag/AgCl (= +1635 mV vs. standard hydrogen electrode).

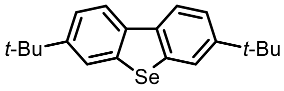

1b

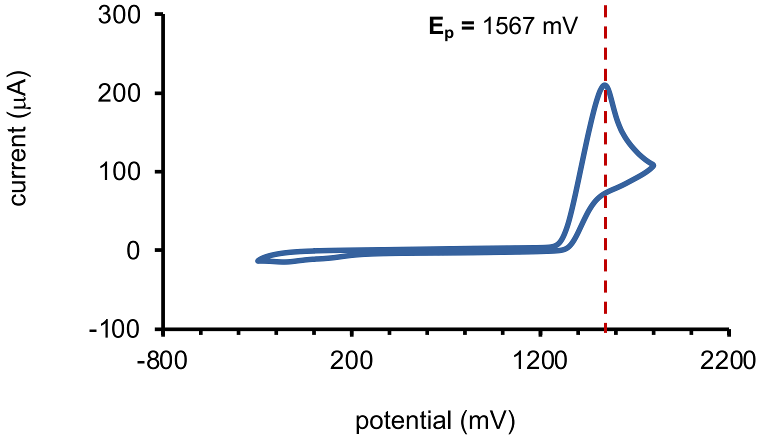

Figure S65. Anodic CV of **1c** in a 0.05 M NBu_4PF_6 MeCN solution. GC electrode. Scan rate: 100 mV/s, potential referred to Ag/AgCl at 298K using a platinum disk counter electrode as counter electrode.

Oxidation of 2,8-di-*tert*-butyldibenzo[*b*,*d*]selenophene (1c)

Redox couple (1c/4c): E_p = +1423 mV vs. Ag/AgCl (= +1633 mV vs. standard hydrogen electrode).

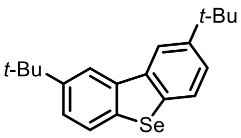

1c

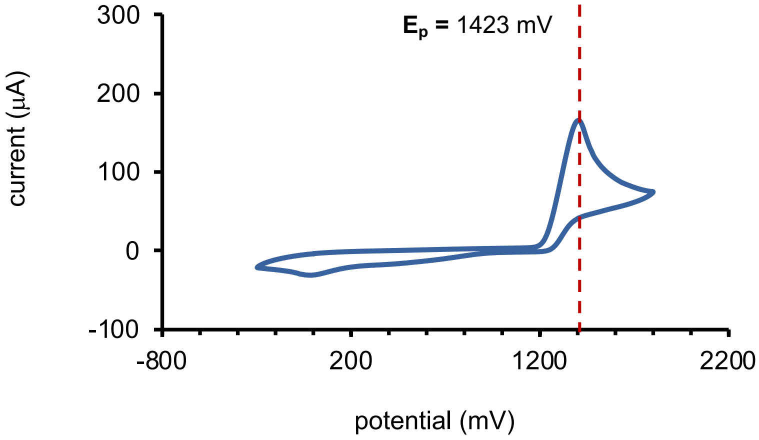

Figure S66. Anodic CV of **1c** in a 0.05 M NBu_4PF_6 MeCN solution. GC electrode. Scan rate: 100 mV/s, potential referred to Ag/AgCl at 298K using a platinum disk counter electrode as counter electrode.

Oxidation of octamethyl-octahydro dinaphthoselenophene 1d

Redox couple (1d/4d): E_p = +1473 mV vs. Ag/AgCl (= +1683 mV vs. standard hydrogen electrode).

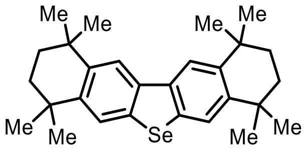

1d

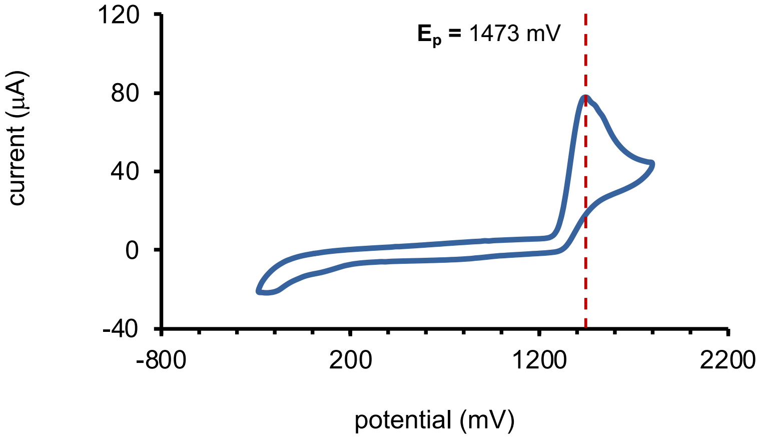

Figure S67. Anodic CV of **1d** in a 0.05 M NBu_4PF_6 MeCN solution. GC electrode. Scan rate: 100 mV/s, potential referred to Ag/AgCl at 298K using a platinum disk counter electrode as counter electrode.

X-RAY CRYSTALLOGRAPHIC ANALYSIS

TRIMERIC CATIONIC SELENURANE [2C]$_3^{\cdot}$(SBF$_6$)$_3$ (CCDC 2463764)

Single crystals suitable for X-ray analysis were obtained by slow gas phase diffusion of pentane (excess) into a concentrated CH_2Cl_2 solution of cationic selenurane **[2c]SbF$_6$** (10 mg in 1 mL of CH_2Cl_2) at –20°C for 120 h.

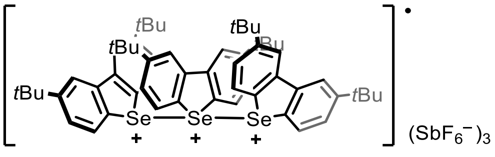

[2c]$_3$ (SbF$_6$)$_3^{\bullet}$

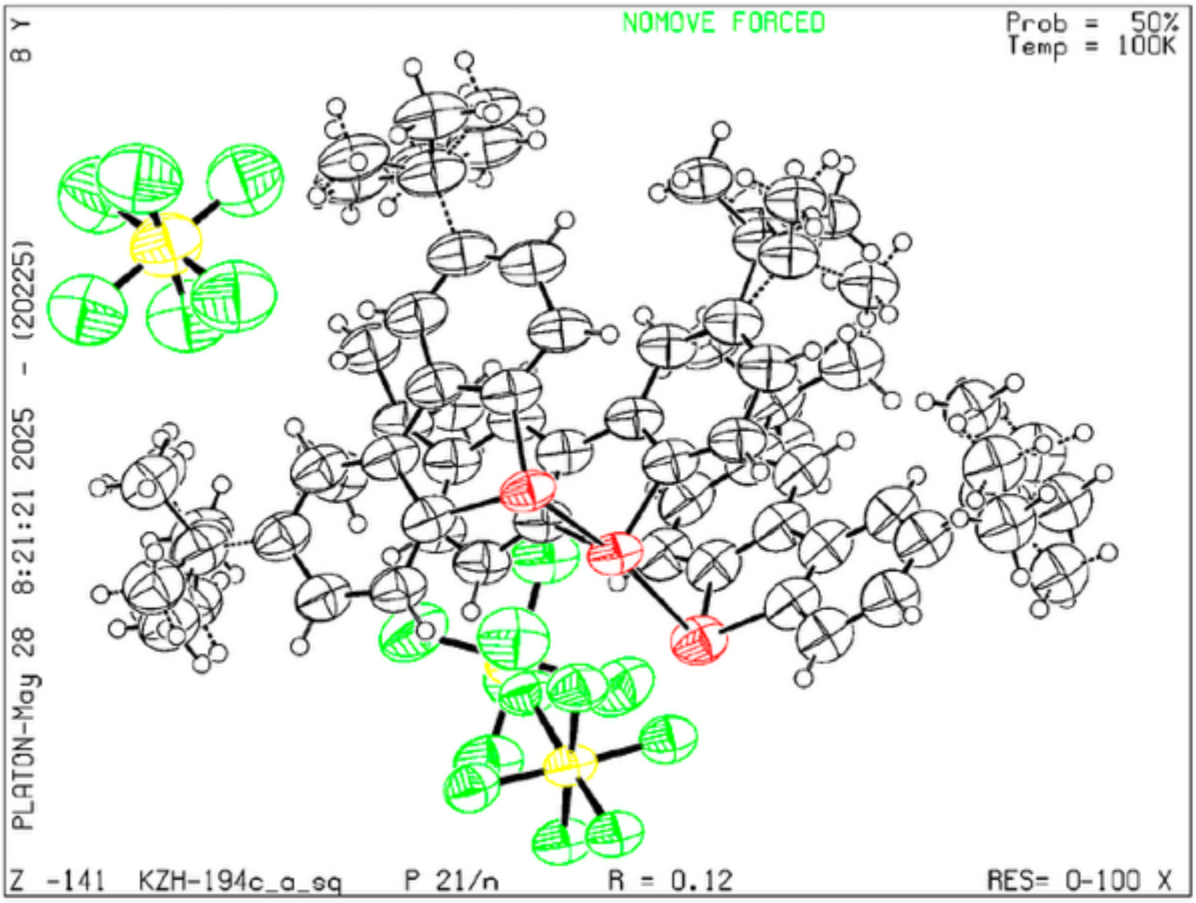

Table S5. Crystal data and structure refinement for **$[2c]_3 \cdot (SbF_6)_3$**.

Identification code	KZH-194c_a_sq	
Empirical formula	C_{30} H_{36} $F_{7.50}$ $Sb_{1.25}$ $Se_{1.50}$	
Color	blue	
Formula weight	809.71 g·mol^{-1}	
Temperature	100.0(1) K	
Wavelength	1.54178 Å	
Crystal system	Monoclinic	
Space group	$P2_1/n$	
Unit cell dimensions	a = 18.517(10) Å	a= 90°.
	b = 20.638(8) Å b= 110.32(2)°.	
	c = 18.839(8) Å g = 90°.	
Volume	6751(6) Å^3	
Z	8	
Density (calculated)	1.593 mg·m^3	
Absorption coefficient	10.407 mm^{-1}	
F(000)	3186	
Crystal size	0.260 x 0.048 x 0.035 mm^3	
Theta range for data collection	2.883 to 72.498°.	
Index ranges	–22<=h<=22, –25<=k<=15, –3<=l<=23	
Reflections collected	195589	
Independent reflections	13236 [R_{int} = 0.0706]	
Completeness to theta = 67.679°	99.4 %	
Absorption correction	Empirical	
Max. and min. transmission	0.4699 and 0.299	
Refinement method	Full-matrix least-squares on F^2	
Data / restraints / parameters	13236 / 4228 / 875	

Goodness-of-fit on F^2	1.042	
Final R indices I>2σ(I)	R1 = 0.1224,	wR2 = 0.3722
R indices (all data)	R1 = 0.1327,	wR2 = 0.3861
Extinction coefficient	n/a	
Largest diff. peak and hole	2.405 and –4.892 e·Å^{-3}	

Computational Data

Calculation of the dimerization enthalpy

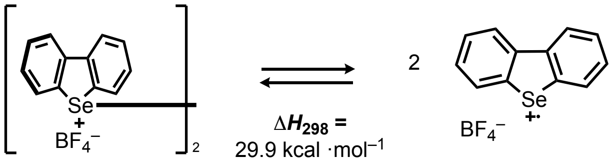

[2a]$_2$ (BF$_4$)$_2$
dimer

[4a] BF$_4$

The nature of all stationary points (minima and transition states) was verified through the computation of the vibrational frequencies. The thermal corrections to the Gibbs free energy were combined with the single point energies calculated at the ωB97X-D/def2-TZVP[58] to yield ωB97X-D/def2-TZVP//ωB97X-D/def2-SVP Gibbs free energies ("G_{298}") and enthalpies ("H_{298}") at 298.15 K. The enthalpies ("H_{298}") were utilized to obtain the dissociation enthalpies (ΔH_{298} = 2H_{298}(**Se-4a**)-H_{298}(**Se-3a**)).

Spin-density comparison

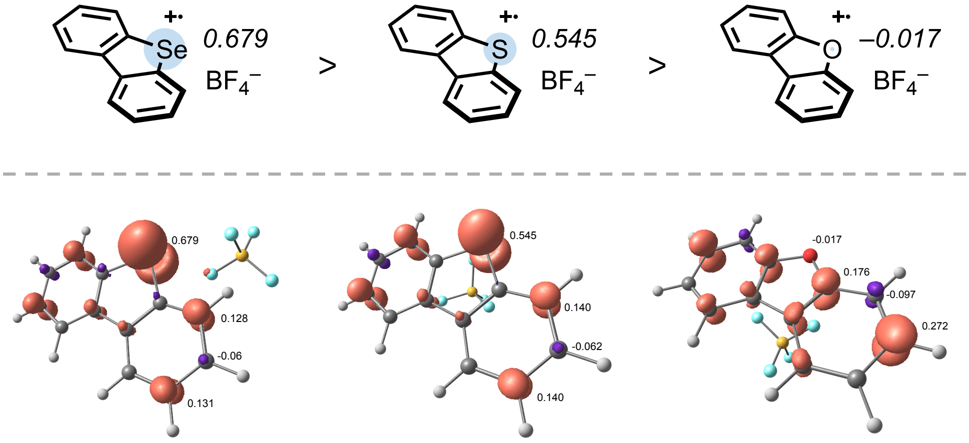

Se **S** **O**

Figure S68.Top: comparison of the spin-density at the chalcogen atom between Se, S, and O-dibenzochalcogenide radical cations. **Bottom.** Spin density distribution and atomic spin populations (Mulliken) for selected atoms of the **Se/S/O-4a** radical, resulting from the ωB97X-D3/def2-TZVP,CPCM(DCM)//ωB97X-D/def2-SVP,SMD(DCM) calculations.

Cartesian coordinates of the most stable ($\Delta G_{298,DCM}$) conformations as computed at the ωB97X-D/def2-TZVP,SMD(DCM)//ωB97X-D/def2-SVP,SMD(DCM) level of theory.

Selenium equilibria

Se-4a

34	-0.006064000	-0.929991000	-0.595857000
6	-1.814687000	-0.960784000	-0.139582000
6	-2.320106000	0.325235000	0.157452000
6	-2.611145000	-2.108737000	-0.080642000
6	-3.655846000	0.449283000	0.515972000
6	-3.948793000	-1.961414000	0.282151000
1	-2.195484000	-3.091708000	-0.310576000
6	-4.461931000	-0.695912000	0.576220000
1	-4.077740000	1.428532000	0.750589000
1	-4.595011000	-2.839202000	0.336748000
1	-5.511325000	-0.593459000	0.860131000
6	-0.034969000	0.923865000	-0.351519000
6	-1.311211000	1.390796000	0.036961000
6	1.056590000	1.778784000	-0.520325000
6	-1.482184000	2.750829000	0.257008000
6	0.859511000	3.140917000	-0.293796000
1	2.030667000	1.386400000	-0.811550000
6	-0.393615000	3.618910000	0.089984000
1	-2.455056000	3.143878000	0.558687000
1	1.695680000	3.831634000	-0.415089000
1	-0.533043000	4.687501000	0.266119000
5	3.346103000	-0.690894000	0.398367000
9	2.832992000	-0.713275000	-0.926858000
9	2.259364000	-0.783996000	1.288953000
9	4.203798000	-1.772539000	0.577728000
9	4.017989000	0.515362000	0.599392000

Se-3a

34	-0.072196000	-0.720137000	-1.278097000
6	-1.178972000	0.807722000	-1.453459000
6	-0.429278000	1.977352000	-1.680307000
6	-2.564501000	0.806230000	-1.334259000
6	-1.106666000	3.189292000	-1.771012000
6	-3.220709000	2.034097000	-1.428499000
1	-3.120171000	-0.115815000	-1.165316000
6	-2.497851000	3.209389000	-1.635636000
1	-0.557804000	4.116642000	-1.946600000
1	-4.307325000	2.068415000	-1.333713000
1	-3.026809000	4.162215000	-1.702886000
6	1.407506000	0.406688000	-1.660250000
6	1.019853000	1.749720000	-1.813907000
6	2.723598000	-0.019580000	-1.783511000
6	2.003034000	2.693828000	-2.090293000
6	3.692551000	0.944574000	-2.072019000
1	2.997257000	-1.061613000	-1.627169000
6	3.334522000	2.283550000	-2.217074000
1	1.737884000	3.745115000	-2.216201000
1	4.736181000	0.640600000	-2.169519000
1	4.103703000	3.027683000	-2.433364000
34	0.071992000	-0.720069000	1.277956000
6	0.429722000	1.977318000	1.680303000
6	1.179133000	0.807527000	1.453334000
6	2.564655000	0.805740000	1.334042000
6	3.221145000	2.033447000	1.428404000
6	2.498578000	3.208881000	1.635738000

6	1.107394000	3.189087000	1.771148000
1	3.120159000	-0.116365000	1.164865000
1	4.307763000	2.067523000	1.333553000
1	3.027758000	4.161576000	1.703102000
1	0.558760000	4.116545000	1.946865000
6	-3.692375000	0.945574000	2.071775000
6	-2.723665000	-0.018811000	1.783202000
6	-1.407456000	0.407119000	1.660039000
6	-1.019466000	1.750039000	1.813851000
6	-2.002404000	2.694380000	2.090304000
6	-3.334006000	2.284438000	2.216995000
1	-4.736086000	0.641856000	2.169198000
1	-2.997664000	-1.060740000	1.626750000
1	-1.736995000	3.745589000	2.216318000
1	-4.102997000	3.028751000	2.433337000
5	-2.897467000	-2.763739000	-0.693416000
5	2.896928000	-2.763987000	0.693734000
9	3.127575000	-4.129336000	0.709214000
9	4.037471000	-2.067162000	0.272920000
9	1.834095000	-2.472908000	-0.215304000
9	-2.516735000	-2.307055000	-1.964005000
9	-3.128462000	-4.129034000	-0.708666000
9	-1.834217000	-2.472817000	0.215150000
9	-4.037733000	-2.066617000	-0.272257000
9	2.515780000	-2.307067000	1.964119000

I

6	3.424560000	0.637792000	0.040673000
6	4.109843000	-0.453929000	0.593378000
6	3.844183000	1.952448000	0.189881000
6	5.257186000	-0.199969000	1.346793000
6	4.993460000	2.181930000	0.947440000
1	3.298221000	2.773453000	-0.271698000
6	5.687439000	1.115231000	1.521894000
1	5.817037000	-1.023284000	1.794161000
1	5.348936000	3.204836000	1.084587000
1	6.585141000	1.310738000	2.112035000
6	2.366611000	-1.721249000	-0.516553000
6	3.524008000	-1.769270000	0.277659000
6	1.713864000	-2.858885000	-0.969668000
6	4.011051000	-3.018885000	0.662024000
6	2.217624000	-4.097494000	-0.568426000
1	0.849540000	-2.800380000	-1.627463000
6	3.348423000	-4.173819000	0.245289000
1	4.909518000	-3.094335000	1.277398000
1	1.719469000	-5.008825000	-0.904371000
1	3.730779000	-5.150154000	0.550147000
6	-1.942142000	0.429307000	0.448750000
6	-1.885382000	-0.508733000	1.533655000
6	-0.648505000	-0.959015000	2.088969000
6	0.511396000	-0.496077000	1.572089000
6	0.519559000	0.479928000	0.466590000
6	-0.780615000	0.843207000	-0.123367000
1	-0.648504000	-1.660937000	2.923935000
1	1.467273000	-0.801238000	2.002932000
1	0.925045000	1.445432000	0.852652000
1	-0.782984000	1.526046000	-0.973956000
6	-6.016661000	1.166225000	-0.476541000
6	-5.647982000	0.277103000	0.529590000
6	-4.288487000	0.095986000	0.774649000

6	-3.303066000	0.773493000	0.040805000
6	-3.691995000	1.668166000	-0.960771000
6	-5.045978000	1.857496000	-1.212993000
1	-7.075599000	1.326358000	-0.690032000
1	-6.403150000	-0.260330000	1.106138000
1	-2.939220000	2.217413000	-1.529409000
1	-5.355265000	2.554293000	-1.994497000
5	0.744660000	3.839315000	-0.375363000
5	-1.481224000	-1.947427000	-1.942789000
9	-1.526327000	-2.390820000	-0.616956000
9	-2.698854000	-1.397923000	-2.320252000
9	-0.463863000	-0.946138000	-2.054650000
9	-0.628025000	3.977271000	-0.570328000
9	0.995532000	3.420220000	0.949970000
9	1.227807000	2.818721000	-1.248157000
9	1.419650000	5.020345000	-0.638775000
9	-1.129228000	-3.009854000	-2.781479000
34	1.880055000	0.082524000	-0.951571000
34	-3.533114000	-1.046372000	2.089600000

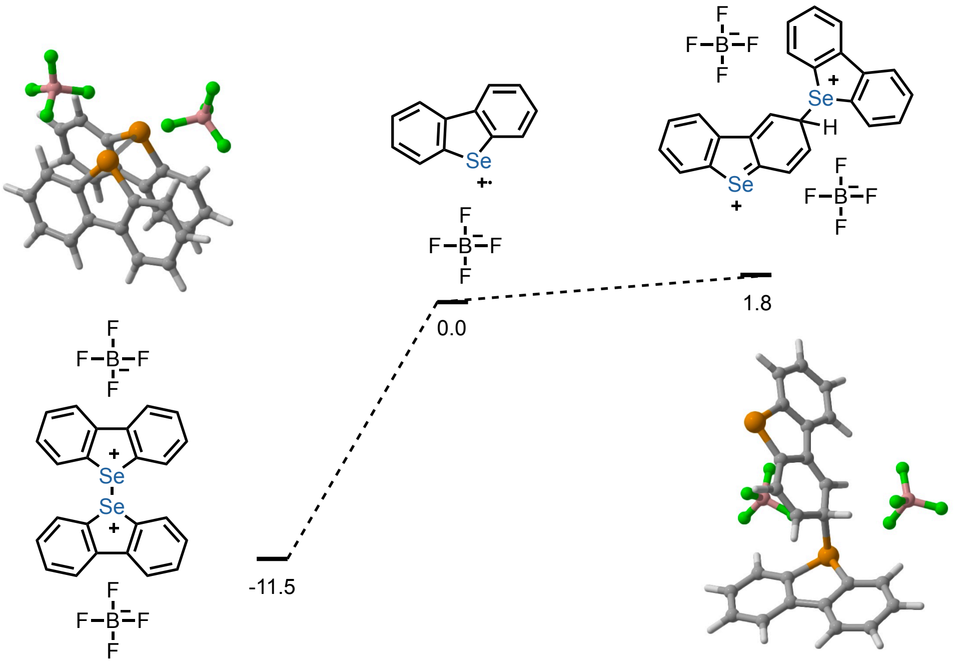

Figure S69. Representation of the calculated dimerization equilibria resulting from the ωB97X-D3/def2-TZVP,CPCM(DCM)//ωB97X-D/def2-SVP,SMD(DCM) calculations.

Selenium equilibria without consideration of the counterions

4a_no_counterion

Se	-1.73290	-0.00000	0.00000
C	-0.39694	-0.00001	-1.30073
C	0.89953	-0.00002	-0.73588
C	-0.61246	0.00000	-2.68221
C	1.99443	-0.00001	-1.58783
C	0.50250	0.00001	-3.51876
H	-1.62372	0.00002	-3.09301
C	1.78872	0.00001	-2.97583
H	3.00855	-0.00001	-1.18344
H	0.36554	0.00003	-4.60129
H	2.65426	0.00002	-3.64140
C	-0.39694	-0.00001	1.30073
C	0.89953	-0.00002	0.73588
C	-0.61246	0.00000	2.68221
C	1.99443	-0.00001	1.58783
C	0.50250	0.00001	3.51876
H	-1.62372	0.00002	3.09301
C	1.78872	0.00001	2.97583
H	3.00855	-0.00001	1.18344
H	0.36554	0.00003	4.60129
H	2.65426	0.00002	3.64140

Se–3a_head-to-head_no_counterion

Se	-0.43535	1.31876	-1.63305
C	-1.97967	0.98972	-0.59387
C	-1.72760	1.16940	0.77941
C	-3.22017	0.62331	-1.10007
C	-2.77158	0.95341	1.67137
C	-4.25576	0.41960	-0.18465
H	-3.38603	0.49865	-2.17142
C	-4.02813	0.57856	1.18165
H	-2.61832	1.08471	2.74418
H	-5.24527	0.13553	-0.54663
H	-4.84571	0.41372	1.88600
C	0.45991	1.79659	-0.04426
C	-0.35463	1.60685	1.08866
C	1.77525	2.24638	0.02428
C	0.19666	1.86258	2.33912
C	2.30525	2.49238	1.28940
H	2.37108	2.40023	-0.87628
C	1.52487	2.29191	2.42974
H	-0.39981	1.73151	3.24331
H	3.33448	2.84211	1.38433
H	1.95493	2.48477	3.41454
Se	0.42812	-1.16449	-1.73899
C	1.73212	-1.24294	0.67146
C	1.98167	-0.94362	-0.68102
C	3.22064	-0.53014	-1.15392
C	4.25801	-0.40890	-0.22569
C	4.03320	-0.68968	1.12123
C	2.77762	-1.10685	1.57725
H	3.38248	-0.30733	-2.20988
H	5.24630	-0.09110	-0.56216
H	4.85193	-0.58790	1.83627
H	2.62424	-1.33198	2.63432
C	-2.30476	-2.58429	1.07794
C	-1.77926	-2.22319	-0.16132

C	-0.46017	-1.78251	-0.19709
C	0.35925	-1.70253	0.94573
C	-0.18764	-2.06872	2.17067
C	-1.51789	-2.49722	2.22802
H	-3.33659	-2.93254	1.14538
H	-2.38133	-2.28243	-1.06930
H	0.41508	-2.02510	3.07950
H	-1.94465	-2.77870	3.19261

I_no_counterion

C	-1.14112	-1.25794	1.09839
C	-0.77924	0.03144	1.53325
C	-0.51446	-2.41764	1.54826
C	0.28996	0.14896	2.41787
C	0.54508	-2.27548	2.44000
H	-0.83657	-3.40263	1.20689
C	0.95016	-1.00284	2.85357
H	0.60982	1.13126	2.77030
H	1.06157	-3.16184	2.81203
H	1.78711	-0.90537	3.54782
C	-2.61884	0.67912	0.10259
C	-1.61166	1.11263	0.97932
C	-3.52368	1.53294	-0.50825
C	-1.50682	2.47934	1.23141
C	-3.39991	2.89845	-0.23803
H	-4.29711	1.16005	-1.18167
C	-2.39973	3.36134	0.61814
H	-0.73714	2.85748	1.90651
H	-4.09379	3.60290	-0.70000
H	-2.31541	4.43116	0.81862
C	0.65475	0.03316	-1.35183
C	1.28493	-1.23507	-1.05025
C	0.67531	-2.48450	-1.40127
C	-0.60313	-2.48024	-1.84616
C	-1.36016	-1.22204	-1.93794
C	-0.60411	0.03004	-1.86344
H	1.23346	-3.41490	-1.28826
H	-1.11611	-3.41237	-2.09119
H	-2.17584	-1.22815	-2.67355
H	-1.11034	0.96111	-2.12240
C	3.19837	3.12789	0.04872
C	3.53762	1.78364	0.19366
C	2.65866	0.83434	-0.31834
C	1.46238	1.18944	-0.96126
C	1.13935	2.54169	-1.09742
C	2.01192	3.50219	-0.59221
H	3.86874	3.89507	0.44138
H	4.46044	1.48962	0.69676
H	0.21478	2.84326	-1.59377
H	1.76580	4.56058	-0.69684
Se	-2.53906	-1.21280	-0.16728
Se	2.88044	-1.04815	-0.22229

Discussion on the observed equilibria in solution

Our experiments and calculations were conducted in dichloromethane (DCM), a relatively apolar solvent. This property of DCM supports the use of the ion pair complex approximation with reasonable confidence. However, we acknowledge the limitations of this approach, as even apolar solvents like DCM can solvate ions to some extent, partially disrupting or loosening the ion pair complexes. In this context, the alternative approximation of "naked" cations (ignoring counterions) would yield a completely opposite perspective.

To address the question of why both the monomer and dimer coexist in solution, we also applied the "naked" cation approximation. ***Figure S70*** *presents the relative Gibbs free energies (ΔG_{298}, kcal/mol, ωB97X-D/def2-TZVP,SMD(DCM)//ωB97X-D/def2-SVP,SMD(DCM)) for the studied Se-derivatives without considering counterions. As shown, similar to the ion pair approximation, the formation of the "head-to-tail" dimer* ***Se-5a*** *is thermodynamically unfavorable (9.7 kcal/mol). In contrast, the "head-to-head" dimer is 1.4 kcal/mol more stable than the cationic radical* ***4a****. This small Gibbs free energy difference of 1.4 kcal/mol provides a plausible explanation for the monomer/"head-to-head" dimer equilibrium observed in solution.*

Overall, both computational approximations have their respective strengths and limitations. Neither the tight ion pair complexes nor the "naked" cations (without counterions) fully capture the reality, which likely lies somewhere between these two extremes. However, based on our studies, we believe that employing both approximations, while remaining mindful of their limitations, provides valuable insights into the experimental observations.

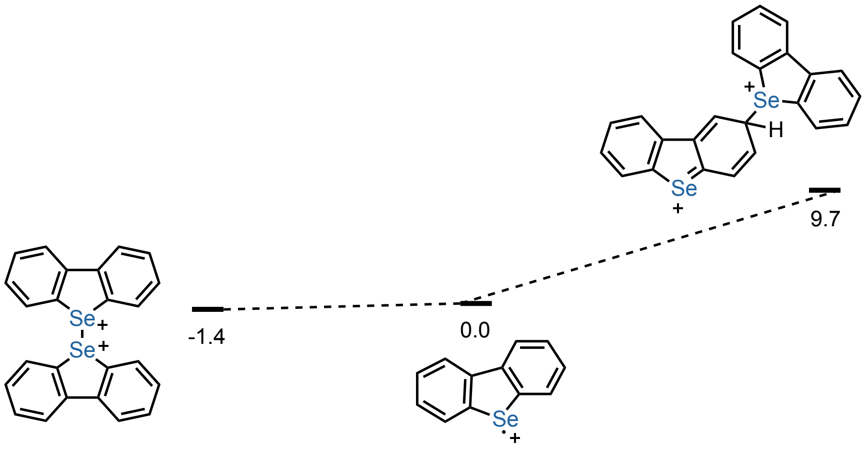

Figure S70. Relative Gibbs free energies (ΔG_{298}) for the selenium radical cation **4a** and the two alternative dicationic dimers: “head-to-head” **Se-3a** and “head-to-tail” Wheland-type intermediate. The radical cation **4a** is used as the reference (0.0 kcal/mol).

S equilibria

S-4a

6	0.463016000	0.945898000	0.948601000
6	-0.236801000	1.367207000	-0.209076000
6	1.746511000	1.413583000	1.269877000
6	0.362718000	2.285219000	-1.049415000
6	2.326467000	2.341732000	0.412768000
1	2.272645000	1.052702000	2.154517000
6	1.644735000	2.770136000	-0.728815000
1	-0.143745000	2.627124000	-1.953465000
1	3.323996000	2.726939000	0.628287000
1	2.118239000	3.492872000	-1.396180000
6	-1.756427000	-0.188903000	0.756512000
6	-1.538313000	0.701727000	-0.319881000
6	-2.921390000	-0.953168000	0.879928000
6	-2.516314000	0.825262000	-1.291622000
6	-3.892865000	-0.812591000	-0.109587000
1	-3.063895000	-1.639027000	1.716863000
6	-3.691609000	0.063045000	-1.178613000
1	-2.376573000	1.501061000	-2.137246000
1	-4.813525000	-1.394769000	-0.047600000
1	-4.461509000	0.158318000	-1.946879000
16	-0.415050000	-0.221308000	1.843298000
5	1.806515000	-1.696997000	-0.560502000
9	0.404155000	-1.707662000	-0.696989000
9	2.336290000	-2.869765000	-1.106629000
9	2.328670000	-0.580154000	-1.225623000
9	2.132463000	-1.626218000	0.807839000

S-3a

6	-1.525319000	0.718094000	0.998371000
6	-0.925476000	0.269427000	2.192017000
6	-2.892358000	0.955065000	0.879657000
6	-1.730534000	0.028934000	3.296818000
6	-3.680602000	0.705278000	2.000226000
1	-3.329607000	1.323023000	-0.046127000
6	-3.107768000	0.241841000	3.187405000
1	-1.291067000	-0.310611000	4.236118000
1	-4.756340000	0.877729000	1.942221000
1	-3.746382000	0.053703000	4.052832000
6	0.991978000	0.615352000	0.820160000
6	0.534622000	0.180201000	2.079130000
6	2.339705000	0.692520000	0.487439000
6	1.461833000	-0.229742000	3.021375000
6	3.259647000	0.271701000	1.451330000
1	2.662237000	1.081629000	-0.477848000
6	2.825315000	-0.191919000	2.690770000
1	1.137580000	-0.569260000	4.006563000
1	4.326101000	0.315155000	1.225535000
1	3.560268000	-0.516505000	3.429931000
6	2.091637000	-1.971528000	-1.031212000
6	1.323012000	-1.154134000	-1.879487000
6	1.857686000	-0.461075000	-2.962566000
6	3.223185000	-0.606052000	-3.197142000
6	4.002744000	-1.430193000	-2.377979000
6	3.448951000	-2.118749000	-1.297188000
1	1.238869000	0.178431000	-3.594459000
1	3.683723000	-0.074996000	-4.031627000
1	5.069264000	-1.535052000	-2.586814000

1	4.070680000	-2.752179000	-0.662445000
6	-0.617685000	-3.390387000	1.930009000
6	-1.045091000	-2.603205000	0.858076000
6	-0.073567000	-2.189509000	-0.047805000
6	1.286439000	-2.546150000	0.054034000
6	1.684832000	-3.335836000	1.121246000
6	0.725480000	-3.742602000	2.059735000
1	-1.345857000	-3.730011000	2.668128000
1	-2.089222000	-2.315207000	0.731856000
1	2.728936000	-3.634628000	1.230376000
1	1.038348000	-4.357715000	2.905809000
16	-0.334157000	0.999870000	-0.253332000
16	-0.365369000	-1.118942000	-1.400537000
5	-3.477996000	-0.324076000	-2.063004000
5	1.169137000	3.889875000	-0.066826000
9	2.234407000	3.840558000	0.829415000
9	0.942323000	5.188515000	-0.502500000
9	1.438109000	3.050519000	-1.168621000
9	-3.105543000	-1.273079000	-1.073561000
9	-3.543848000	-0.942505000	-3.302735000
9	-4.699219000	0.246452000	-1.715566000
9	-2.481633000	0.687508000	-2.085441000
9	-0.003796000	3.401883000	0.582838000

S-5a

6	-2.614968000	0.604369000	-1.059638000
6	-3.539792000	1.121854000	-0.142834000
6	-2.934295000	-0.344434000	-2.019395000
6	-4.850453000	0.647637000	-0.187094000
6	-4.248457000	-0.810653000	-2.039990000
1	-2.190400000	-0.725511000	-2.720760000
6	-5.191776000	-0.316969000	-1.134633000
1	-5.595372000	1.025670000	0.515690000
1	-4.536679000	-1.569928000	-2.768534000
1	-6.215584000	-0.694564000	-1.168155000
6	-1.581548000	2.324451000	0.528843000
6	-2.943916000	2.106881000	0.771724000
6	-0.784527000	3.170410000	1.283715000
6	-3.542942000	2.800228000	1.822961000
6	-1.403614000	3.839487000	2.340351000
1	0.276955000	3.288014000	1.064550000
6	-2.766099000	3.661720000	2.597952000
1	-4.604902000	2.664578000	2.036328000
1	-0.812752000	4.511123000	2.965729000
1	-3.231455000	4.202716000	3.424346000
6	2.178155000	-0.866092000	0.093374000
6	1.625470000	-1.817760000	0.973216000
6	0.266128000	-1.795497000	1.323000000
6	-0.544919000	-0.812987000	0.777730000
6	0.006596000	0.104110000	-0.134484000
6	1.347260000	0.103903000	-0.485373000
1	-0.153221000	-2.525485000	2.017977000
1	-1.600740000	-0.769952000	1.049850000
1	-0.828351000	-2.531175000	-0.594444000
1	1.755970000	0.829792000	-1.190417000
6	6.298974000	-1.782364000	-0.166961000
6	5.424977000	-2.547447000	0.596529000
6	4.079465000	-2.171280000	0.629683000
6	3.607900000	-1.057939000	-0.091692000
6	4.508428000	-0.289177000	-0.843093000

6	5.846163000	-0.658401000	-0.876567000
1	7.355464000	-2.056980000	-0.206087000
1	5.782818000	-3.413467000	1.157037000
1	4.156321000	0.601438000	-1.367322000
1	6.555786000	-0.063820000	-1.455544000
16	-1.016061000	1.372070000	-0.874890000
16	2.807388000	-2.965403000	1.546827000
5	-3.042239000	-3.684103000	0.069088000
5	2.410458000	3.359217000	-0.842297000
9	1.035711000	3.305981000	-1.161345000
9	2.587819000	2.878570000	0.462068000
9	3.103023000	2.529501000	-1.750595000
9	-3.299726000	-2.520190000	0.650051000
9	-3.768509000	-4.067742000	-0.965894000
9	-1.452893000	-2.994117000	-1.121021000
9	-2.306780000	-4.570205000	0.722227000
9	2.871416000	4.667204000	-0.945396000

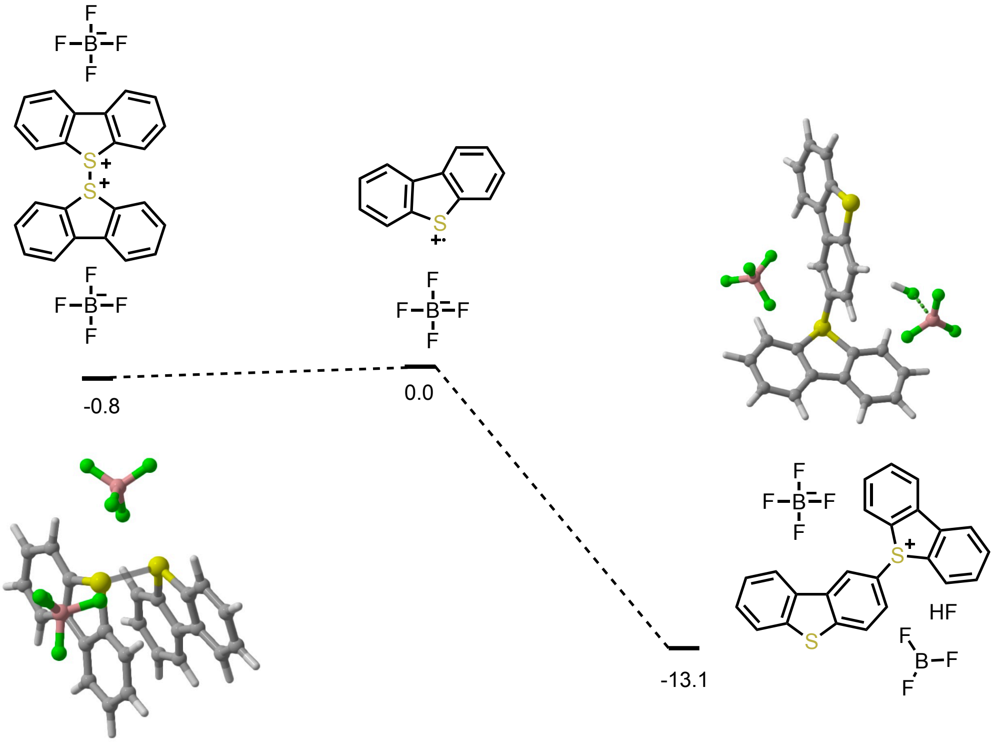

Figure S71. Representation of the calculated dimerization equilibria resulting from the ωB97X-D3/def2-TZVP,CPCM(DCM)//ωB97X-D/def2-SVP,SMD(DCM) calculations.

Sulfur equilibria without consideration of the counterions

4a_no_counterion

C	0.00001	0.71514	1.25170
C	0.00001	-0.60462	0.73369
C	-0.00003	0.99009	2.62828
C	-0.00003	-1.66491	1.62184
C	-0.00004	-0.09368	3.50106
H	-0.00000	2.01641	3.00104
C	-0.00004	-1.40002	3.00458
H	-0.00005	-2.69517	1.26229
H	-0.00004	0.08104	4.57780
H	-0.00006	-2.23914	3.70301

C	0.00001	0.71514	-1.25170
C	0.00001	-0.60462	-0.73369
C	-0.00003	0.99009	-2.62828
C	-0.00003	-1.66491	-1.62184
C	-0.00004	-0.09368	-3.50106
H	-0.00000	2.01641	-3.00104
C	-0.00004	-1.40002	-3.00458
H	-0.00005	-2.69517	-1.26229
H	-0.00004	0.08104	-4.57780
H	-0.00006	-2.23914	-3.70301
S	0.00011	1.89810	0.00000

S–3a_head-to-head_no_counterion

C	0.17331	-0.04218	1.55494
C	1.20970	0.86452	1.23164
C	-1.06116	0.36227	2.07463
C	0.98678	2.21546	1.40894
C	-1.26302	1.73321	2.24608
H	-1.83672	-0.35967	2.33774
C	-0.26231	2.64074	1.90610
H	1.76460	2.94237	1.17041
H	-2.21367	2.08968	2.64409
H	-0.43990	3.70923	2.04293
C	2.19050	-1.21828	0.60803
C	2.39427	0.17553	0.70764
C	3.16122	-2.09866	0.12155
C	3.62417	0.69972	0.32953
C	4.37745	-1.54719	-0.26480
H	2.97768	-3.17232	0.05040
C	4.60457	-0.16841	-0.15567
H	3.81524	1.77132	0.40874
H	5.16471	-2.19838	-0.64765
H	5.57252	0.23710	-0.45623
C	-2.39428	0.17548	-0.70768
C	-2.19053	-1.21832	-0.60793
C	-3.16128	-2.09864	-0.12139
C	-4.37751	-1.54711	0.26486
C	-4.60461	-0.16835	0.15558
C	-3.62418	0.69972	-0.32967
H	-2.97775	-3.17229	-0.05013
H	-5.16479	-2.19826	0.64776
H	-5.57257	0.23721	0.45606
H	-3.81524	1.77132	-0.40900
C	1.26307	1.73296	-2.24617
C	1.06118	0.36204	-2.07466
C	-0.17331	-0.04234	-1.55493
C	-1.20968	0.86441	-1.23169
C	-0.98671	2.21533	-1.40907
C	0.26240	2.64055	-1.90623
H	2.21374	2.08939	-2.64420
H	1.83671	-0.35994	-2.33772
H	-1.76451	2.94228	-1.17059
H	0.44003	3.70902	-2.04311
S	0.60094	-1.66972	1.15641
S	-0.60096	-1.66983	-1.15625

S-5a_head-to-tail_no_counterion

C	-2.99294	-0.14510	-0.40409
C	-2.59901	0.89595	0.45048
C	-3.93436	-0.01079	-1.41348
C	-3.18522	2.14612	0.27034

C	-4.50165	1.25353	-1.57766
H	-4.21865	-0.85045	-2.05059
C	-4.12863	2.31297	-0.74579
H	-2.91054	2.98103	0.91750
H	-5.24663	1.40915	-2.35959
H	-4.58872	3.29249	-0.88943
C	-1.25501	-0.88194	1.33019
C	-1.59115	0.47689	1.43483
C	-0.34084	-1.51919	2.15623
C	-0.95552	1.23675	2.41259
C	0.29127	-0.73198	3.12138
H	-0.11980	-2.58370	2.06176
C	-0.01221	0.62575	3.24191
H	-1.19507	2.29532	2.52711
H	1.02082	-1.18992	3.79118
H	0.48992	1.22099	4.00681
C	1.23420	-0.35712	-1.00883
C	1.93410	-1.57883	-0.66232
C	1.32151	-2.86982	-0.74047
C	0.01285	-2.93963	-1.06603
C	-0.78287	-1.71950	-1.36241
C	-0.06489	-0.42718	-1.38426
H	1.89771	-3.77120	-0.52658
H	-0.49765	-3.90463	-1.11416
H	-1.39126	-1.86426	-2.27499
H	-0.62049	0.47263	-1.66135
C	4.05963	2.72411	-0.34695
C	4.36869	1.38278	-0.12641
C	3.36976	0.45093	-0.38393
C	2.09467	0.81265	-0.84699
C	1.80367	2.16064	-1.05733
C	2.79390	3.10729	-0.80537
H	4.81924	3.48440	-0.15668
H	5.35429	1.08335	0.23481
H	0.81782	2.46662	-1.41255
H	2.57923	4.16497	-0.96838
S	-2.13022	-1.64647	-0.01750
S	3.51585	-1.29968	-0.17240

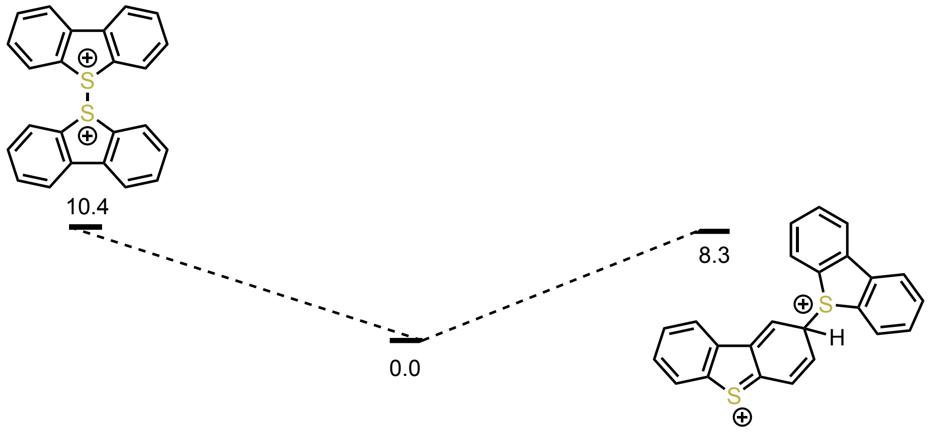

Figure S72. Representation of the calculated dimerization equilibria resulting from the ωB97X-D3/def2-TZVP,CPCM(DCM)//ωB97X-D/def2-SVP,SMD(DCM) calculations.

Closed-shell vs diradical nature of the Se dicationic dimer dimer Se-3a

In order to address the question of whether the Se–Se dimer has a closed-shell or diradical nature, we employed different strategies: first, verifying the applicability of DFT based on the obtained results, and second, performing the CASSCF calculations.

(1) First, we examined the HOMO/LUMO gap of the closed-shell Se–Se dimer computed at the DFT level of theory (ωB97X-D/def2-TZVP, SMD(DCM)//ωB97X-D/def2-SVP, SMD(DCM)). The HOMO/LUMO gap for the dimer complex with counterions is 5.9 eV, while for the "naked" dimer (without counterions), it is 5.7 eV. The influence of the counterions on this value is minimal. These large values are typical for closed-shell molecules with no significant diradical character, indicating that the likelihood of a singlet diradical competing with the closed-shell configuration of the dimer is negligible.

(2) Next, we recomputed the Se–Se dimer at the DFT level of theory in the triplet state, representing the triplet diradical system. The computed triplet state was found to be 19.3 kcal·mol^{-1} less stable than the ground-state singlet closed-shell system. This substantial energy gap strongly argues against the plausibility of a diradical triplet character for the Se-Se dimer.

(3) As a further step in this auxiliary DFT study, we attempted to localize a singlet diradical state based on the obtained triplet state using the broken-symmetry approach (the standard DFT method for singlet diradicals). Multiple broken-symmetry tests quickly converged back to the initially localized closed-shell state, further ruling out a diradical character. Additionally, a stability check of the singlet state confirmed that the closed-shell wave function is stable.

(4) Finally, to ensure that our conclusions are not biased by the limitations of DFT, we performed CASSCF calculations. CASSCF(2,2) shows that the singlet state is 97% single-reference, confirming that it is a closed-shell system, consistent with the DFT results. The triplet state was also computed but was found to be significantly (67.6 kcal/mol) less stable than the singlet state. To ensure consistency, we increased the active space to (6,6). The CASSCF(6,6) results are qualitatively very similar to those of CASSCF(2,2), with the singlet state being 92% single-reference. Thus, the CASSCF calculations corroborate the absence of a diradical character for the studied Se-Se dimer.

STRUCTURAL COMPARISONS AND BONDING

Organoselenium oxidation states

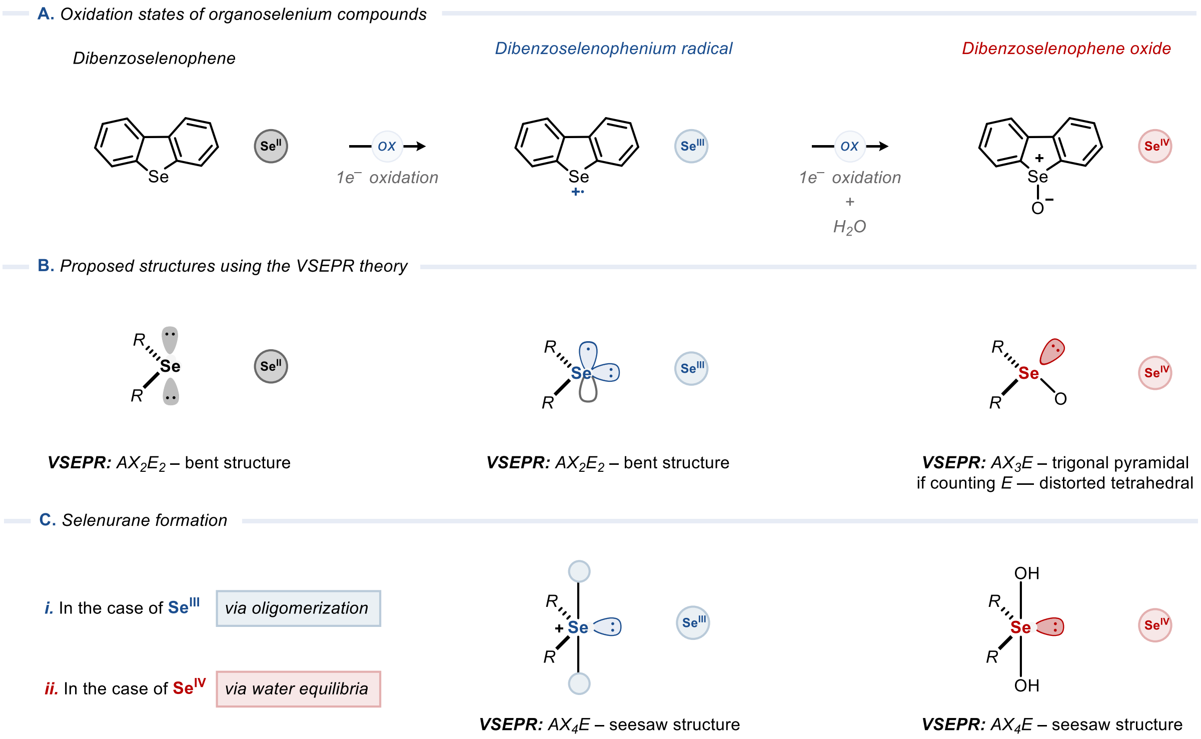

Figure S73. **A.** Oxidation states of: selenides (Se(II) – left), 1c1e selenium radical cations (Se(III) – middle), and selenoxides (Se(IV) – right). **B.** Structures of organoselenium compounds based on the application of the VSEPR theory. **C.** Formation of selenuranes (seesaw structure) from Se(III) or Se(IV) oxidation states. [67]

For an exhaustive analysis and the crystal structures of diaryl-selenide (Se(II)), -selenoxides (Se(IV)), and selenuranes (Se(IV)) see ***Reference 67:*** J. Beckmann, A. Duthie, *Z. Anorg. Allg. Chem.* **2005**, *631*, 1849–1855.

Chalcogen radical cations containing multiple heteroatoms

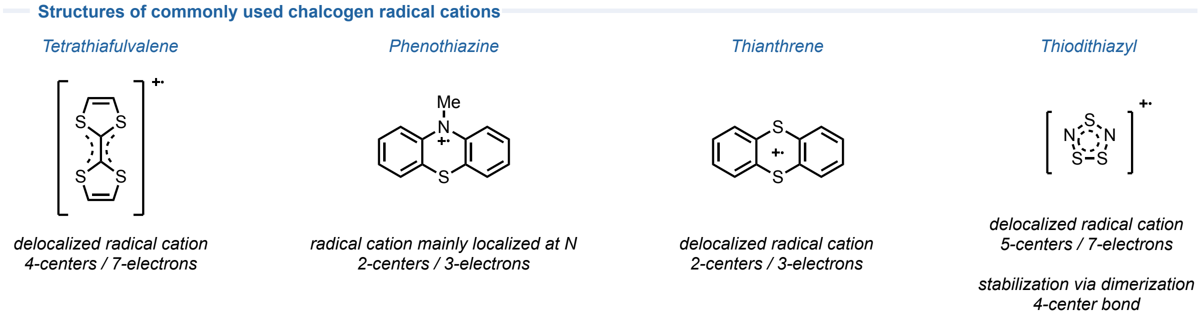

Figure S74. Structures of chalcogen-based radical cations with the spin-density delocalized over multiple heteroatoms.

Tetrathiafulvalene: *4-centers / 7-electron radical cation.* [16]

Phenothiazine: *2-centers / 3-electrons radical cation – spin-density mainly located at the nitrogen atom.* [17]

Thianthrene: *2-centers / 3-electrons radical cation.* [15]

Thiodithiazyl: *5-centers / 7-electrons radical cation – stabilized by dimerization via the formation of 4π-6π-4π dimeric structures (see below for a comparison with cationic selenurane systems).* [18]

Stable Ch(III) radical cations

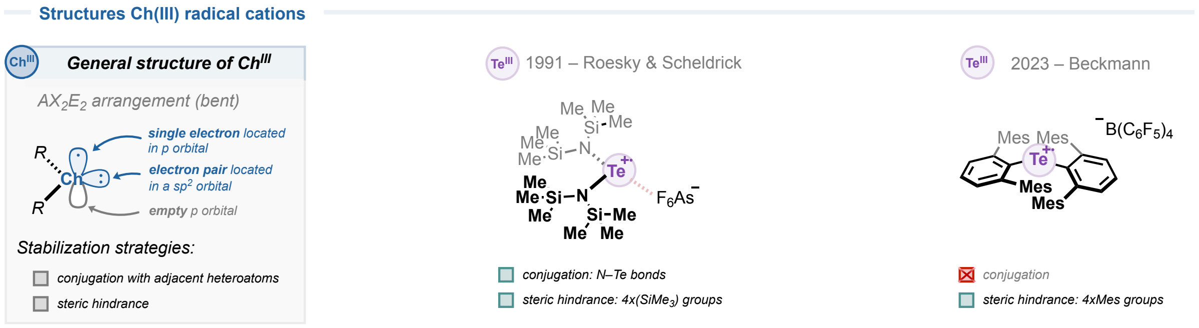

Figure S75. Examples of Ch(III) radical cations reported to date and their stabilization strategies as described by the authors.

Stable radicals defined as storable and bench-stable open-shell compounds.

Roesky & Sheldrick – *Three-fold stabilization: i. heavy-chalcogen atom, ii. steric hindrance, and iii. N–Te conjugation.* [68]

Beckmann – *Two-fold stabilization: i. heavy-chalcogen atom, and ii. steric hindrance.*[26]

Se – Se bond length comparison (graphical representation)

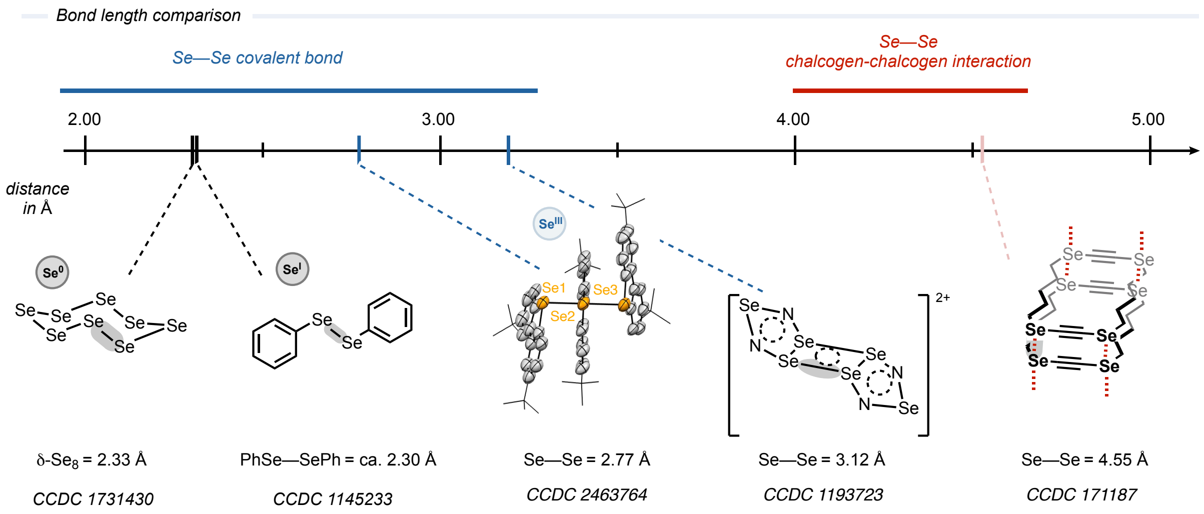

Figure S76. Comparison of the Se – Se bond distances as mentioned in the main text.

Bonding model

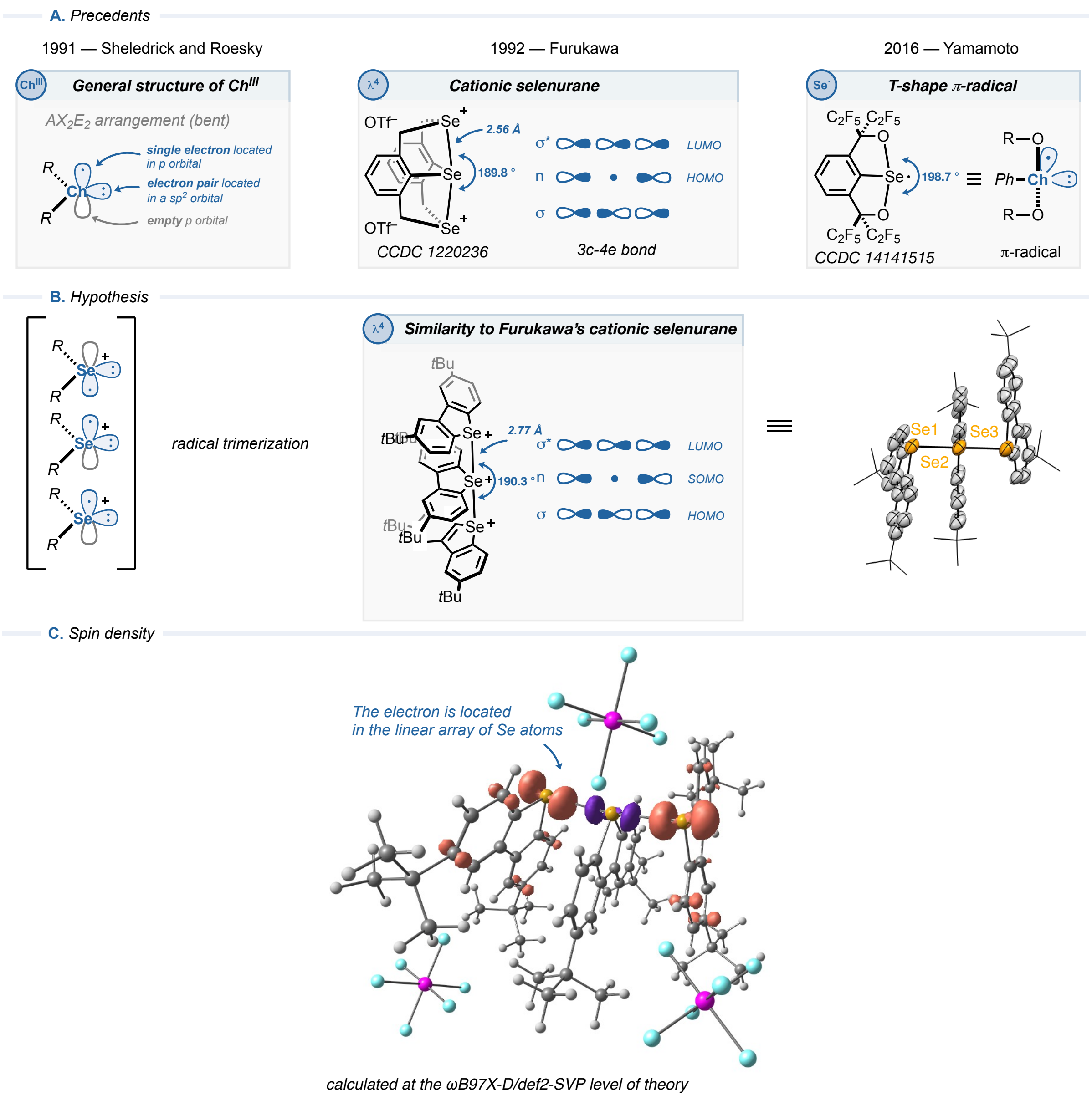

Figure S77. Precedents and comparison with previously reported Ch-based radicals. **A.** Precedents on bonding discussions of chalcogen-based radicals. **B.** Hypothesis and comparison of the reported trimeric structure bonding. **C.** Spin density of the tricationic radical crystal structure.

We hypothesize a bonding model that aligns with prior work from Sheldrick and Roesky [68] (Figure S77 A – left), Furukawa [26] (Figure S77 A – middle), and Yamamoto [69] (Figure S77 A – right).

Bonding hypothesis:

We hypothesize (Figure S77 B) a related framework in trimeric cationic selenuranes. Specifically:

1) *A dibenzoselenophenium radical (e.g., Se1) shares its unpaired electron with a second radical (Se2).*
2) *A third radical cation (Se3) donates an electron to the central Se2.*
3) *The resulting Se1–Se2–Se3 array forms a bonding system in which Se2 serves as a bridge sharing an electron with both Se1 and Se3.*

As pointed out during the revisions of this work, this model can also be understood as following (Figure S77C):

1) *The removal of an electron occurs from highest occupied orbital of a cationic selenurane.*
2) *Using Furukawa's model, the non-bonding orbital is the HOMO.*
3) *If the electron is removed from the non-bonding orbital, the spin density should be located in such.*

Spin density calculations support this hypothesis.

Spectroscopic Data

¹H NMR of dibenzo[*b*,*d*]selenophene (1a)

CDCl₃, 23 °C

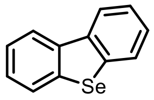

1a

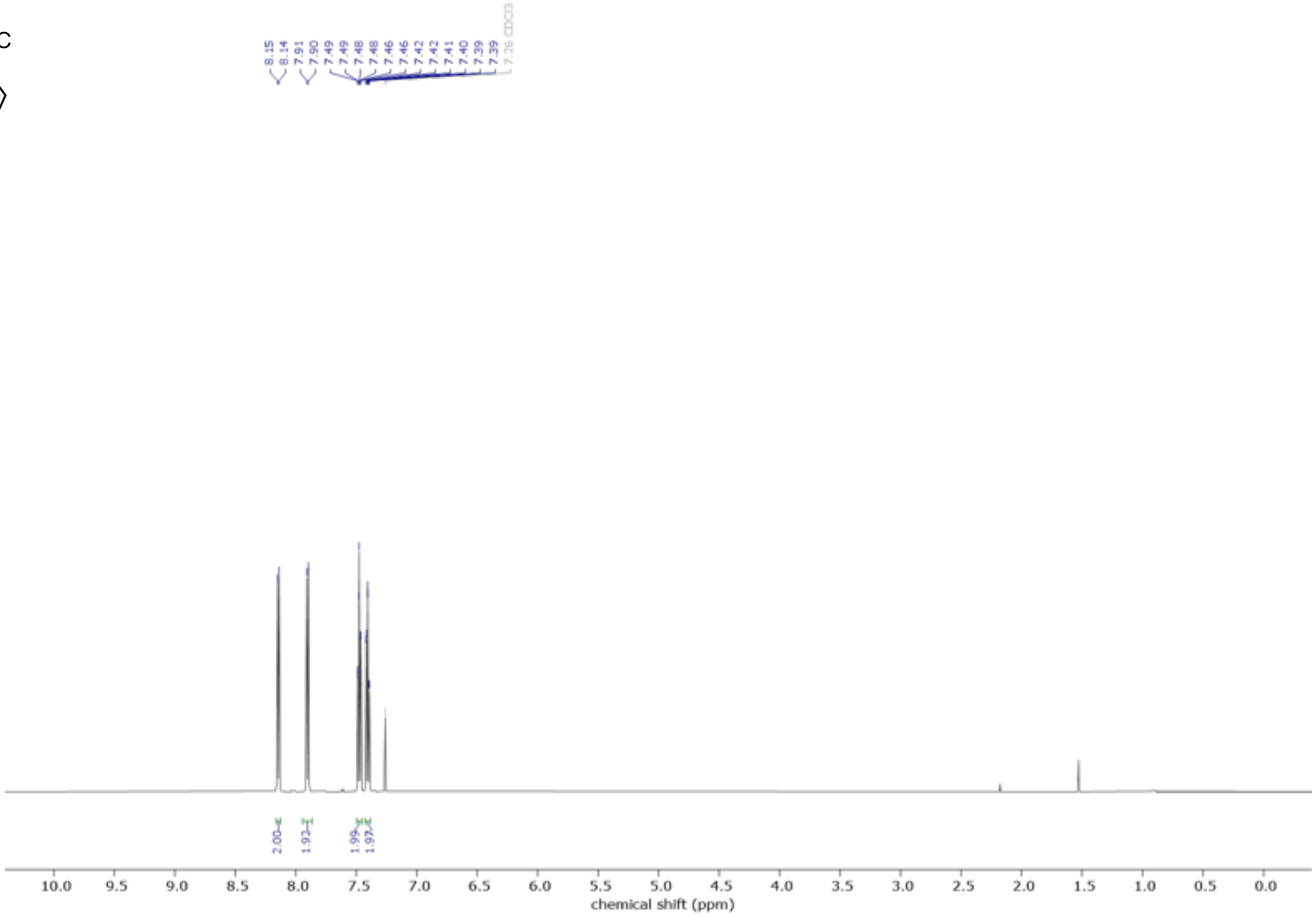

^{13}C NMR OF DIBENZO[*B*,*D*]SELENOPHENE (1A)

$CDCl_3$, 23 ºC

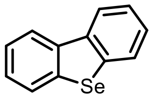

1a

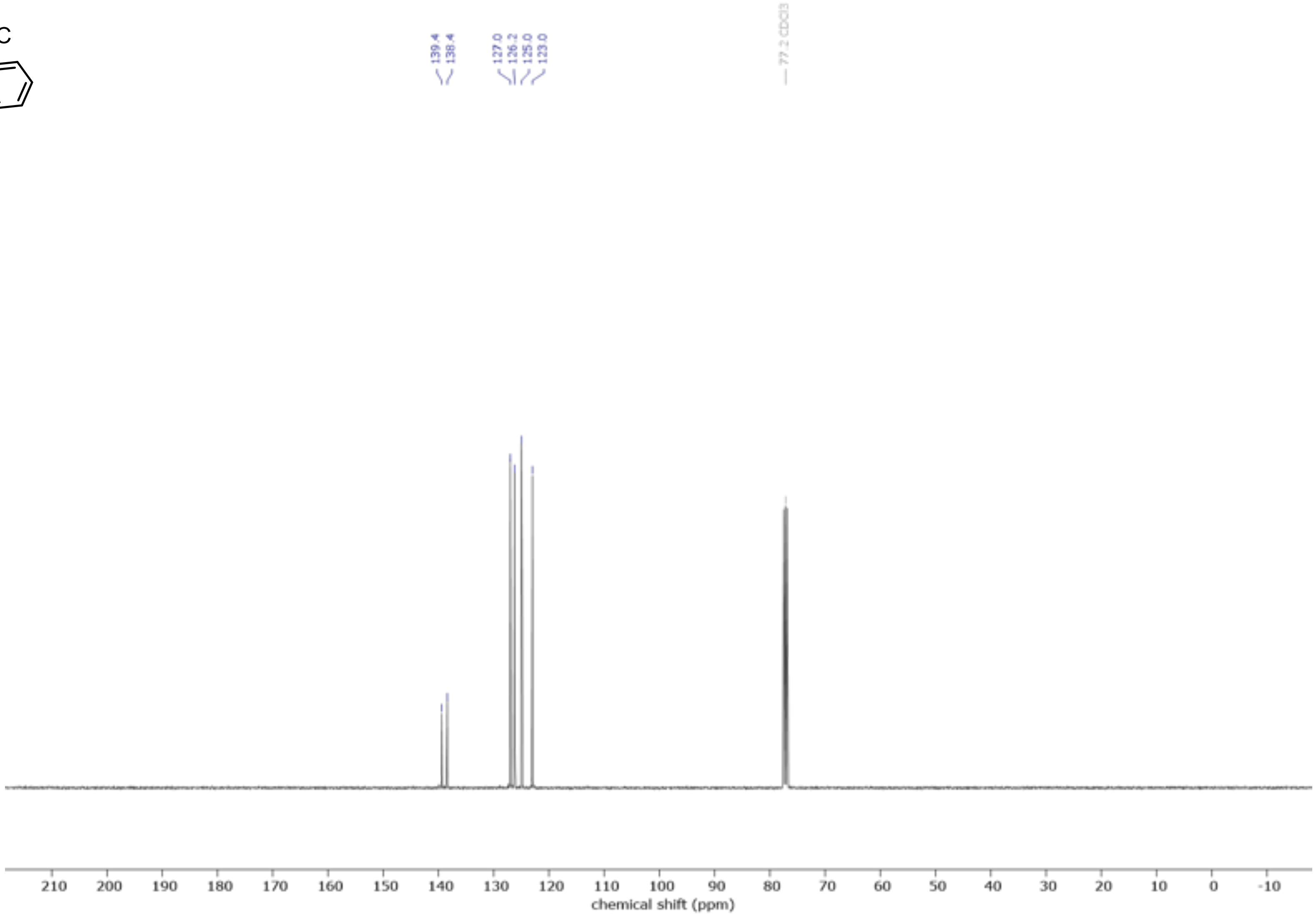

^{77}Se NMR of dibenzo[*b*,*d*]selenophene (1a)

CDCl$_3$, 23 ºC

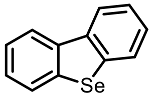

1a

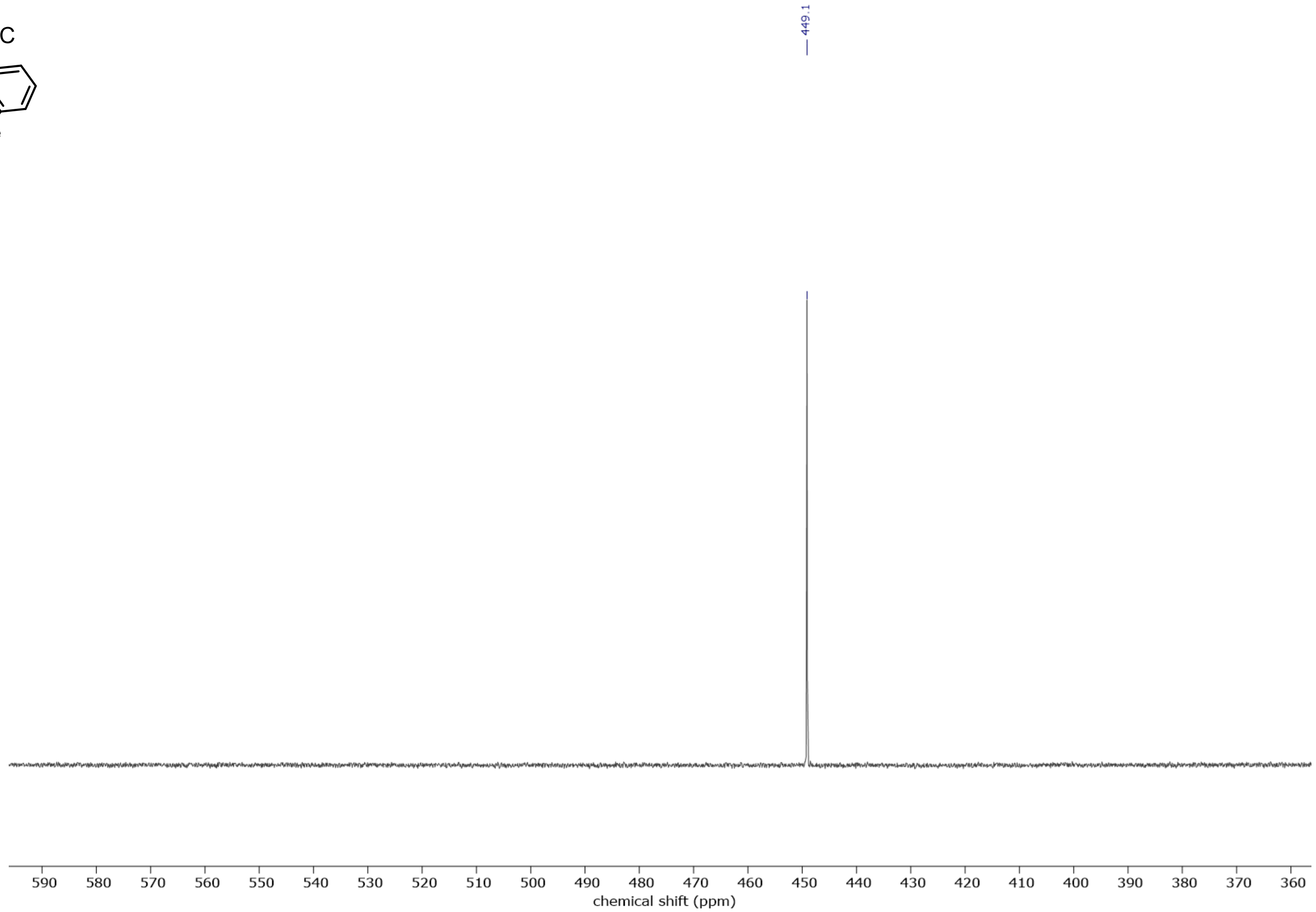

^{1}H NMR OF 3,7-DI-*TERT*-BUTYLDIBENZO[*B*,*D*]SELENOPHENE (1B)

$CDCl_3$, 23 ºC

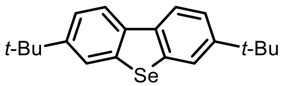

1b

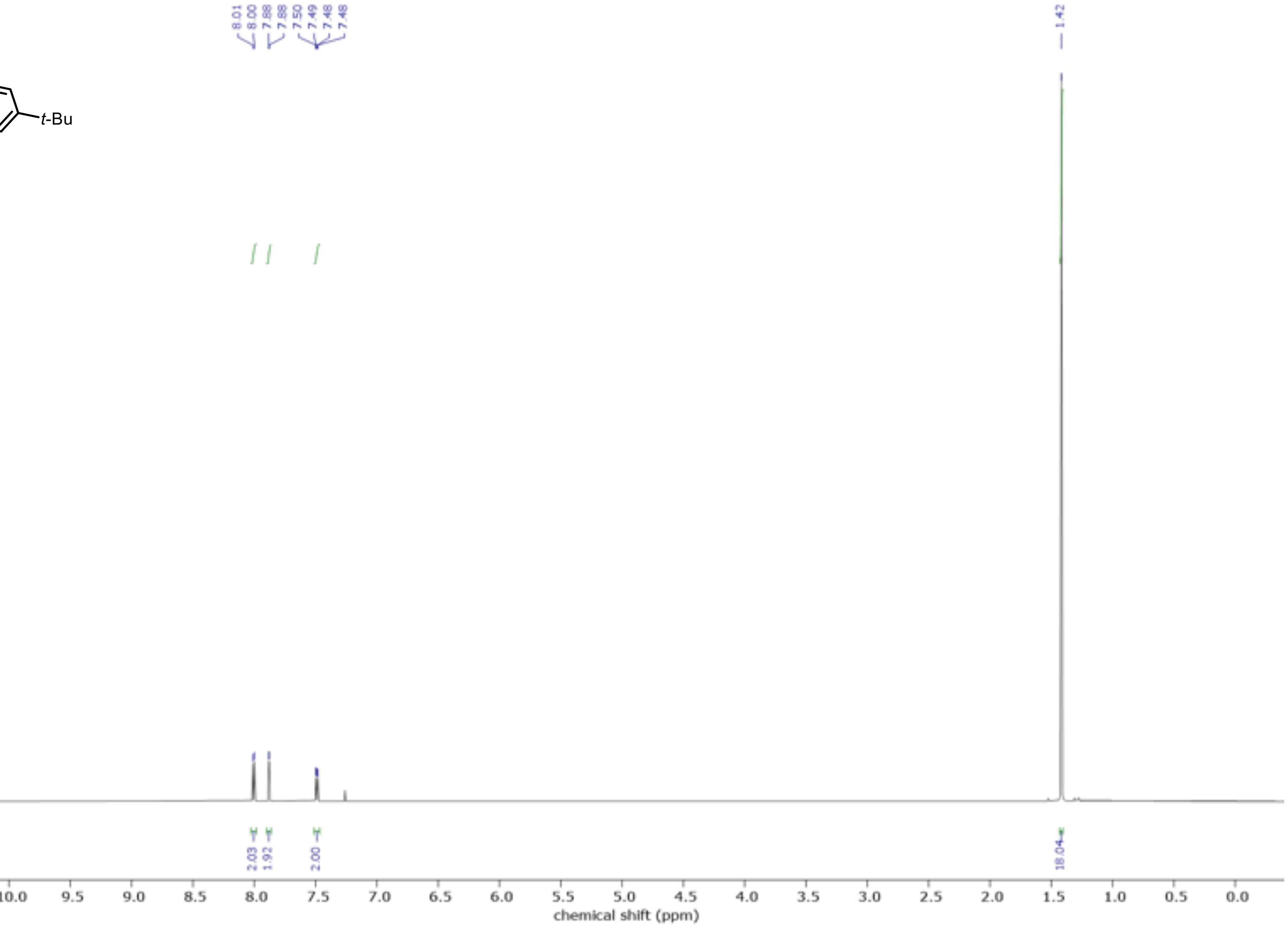

^{13}C NMR OF 3,7-DI-*TERT*-BUTYLDIBENZO[*B*,*D*]SELENOPHENE (1B)

CDCl3, 23 ºC

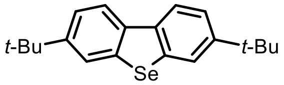

1b

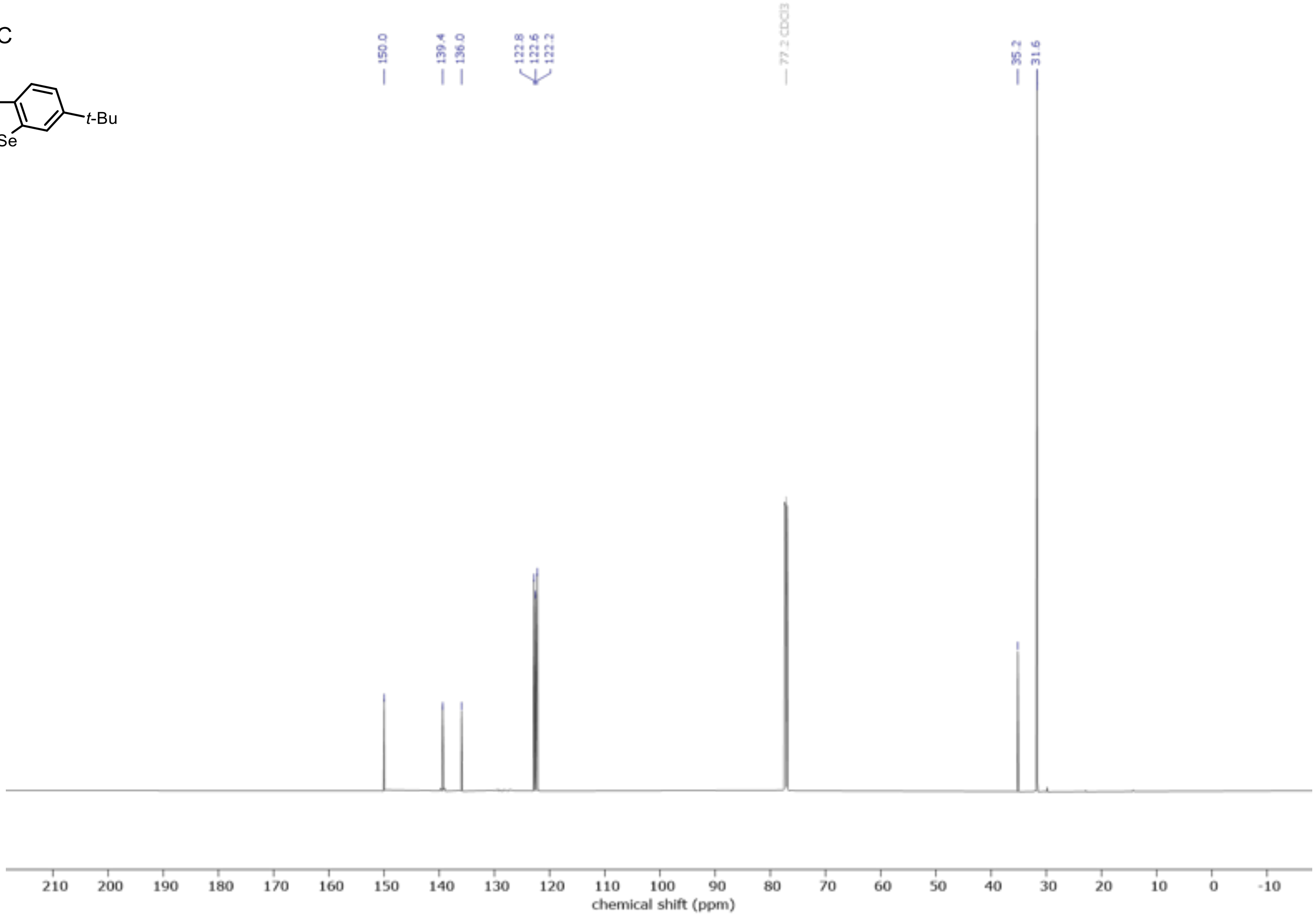

^{77}Se NMR of 3,7-di-*tert*-butyldibenzo[*b*,*d*]selenophene (1b)

CDCl$_3$, 23 ºC

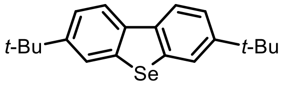

1b

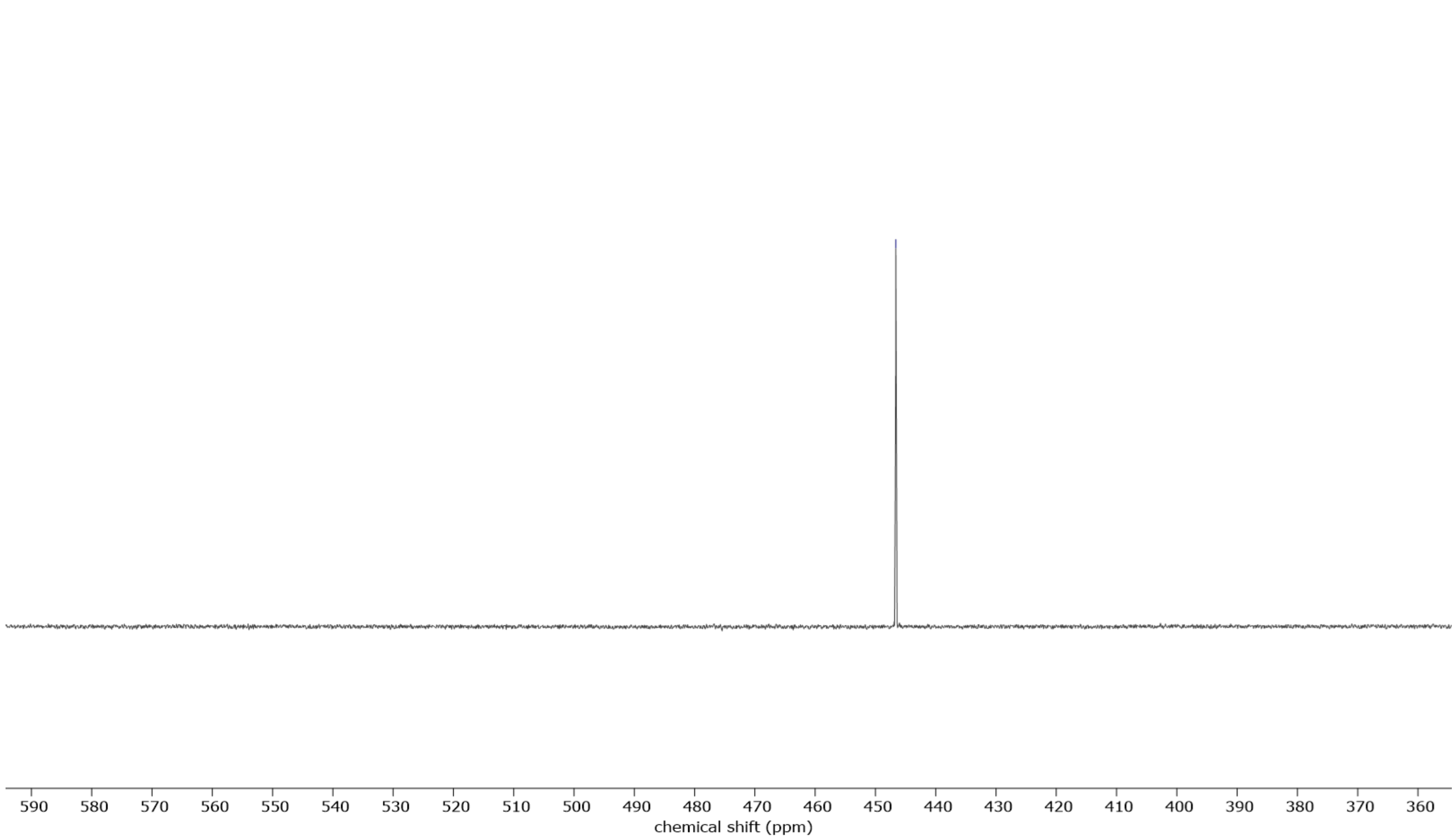

^{1}H NMR OF 2,8-DI-*TERT*-BUTYLDIBENZO[*B*,*D*]SELENOPHENE (1C)

CDCl$_3$, 23 ºC

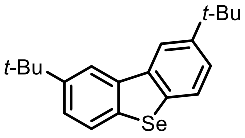

1c

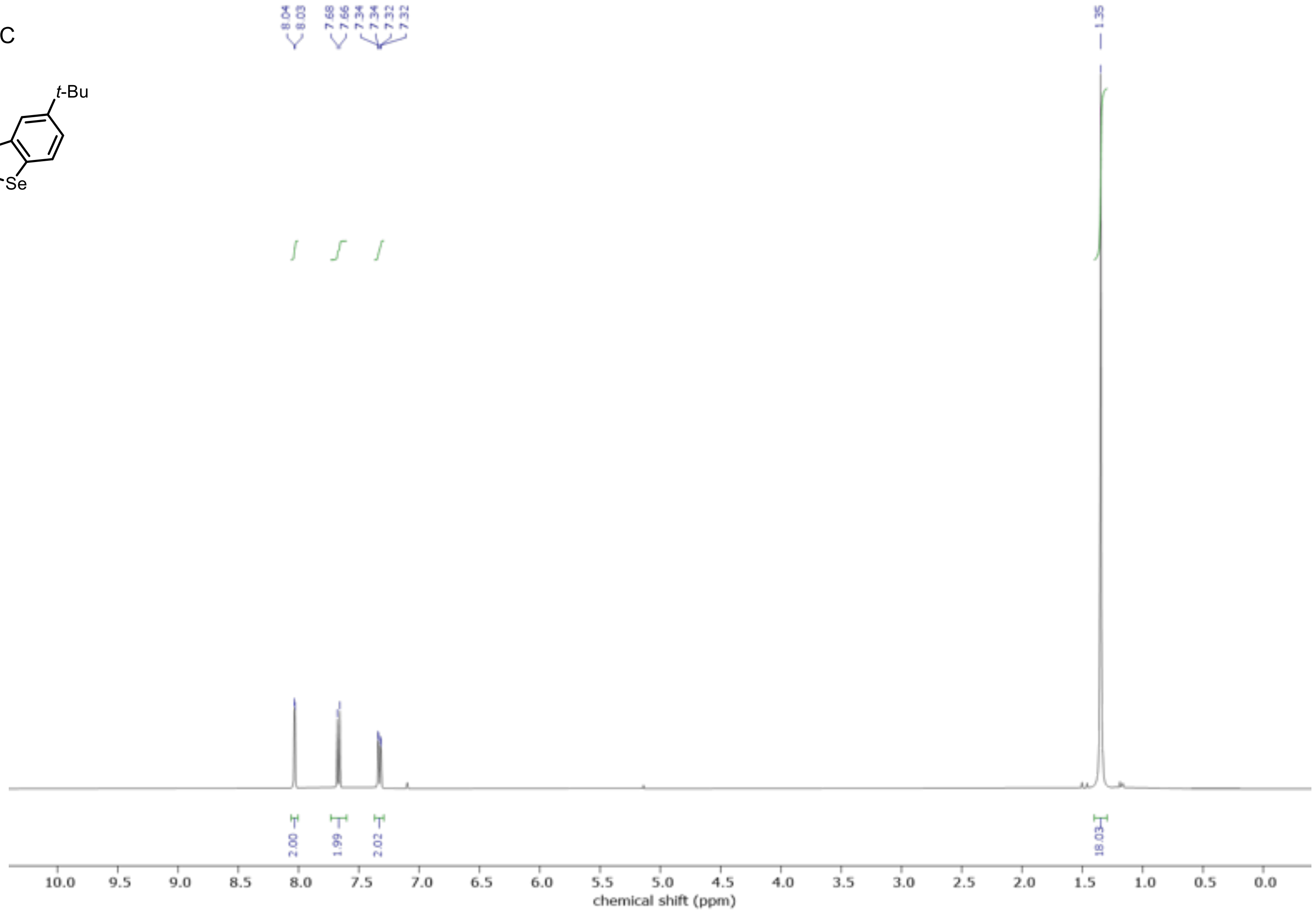

^{13}C NMR OF 2,8-DI-*TERT*-BUTYLDIBENZO[*B*,*D*]SELENOPHENE (1C)

CDCl3, 23 ºC

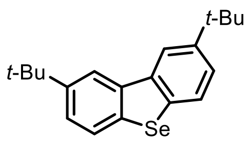

1c

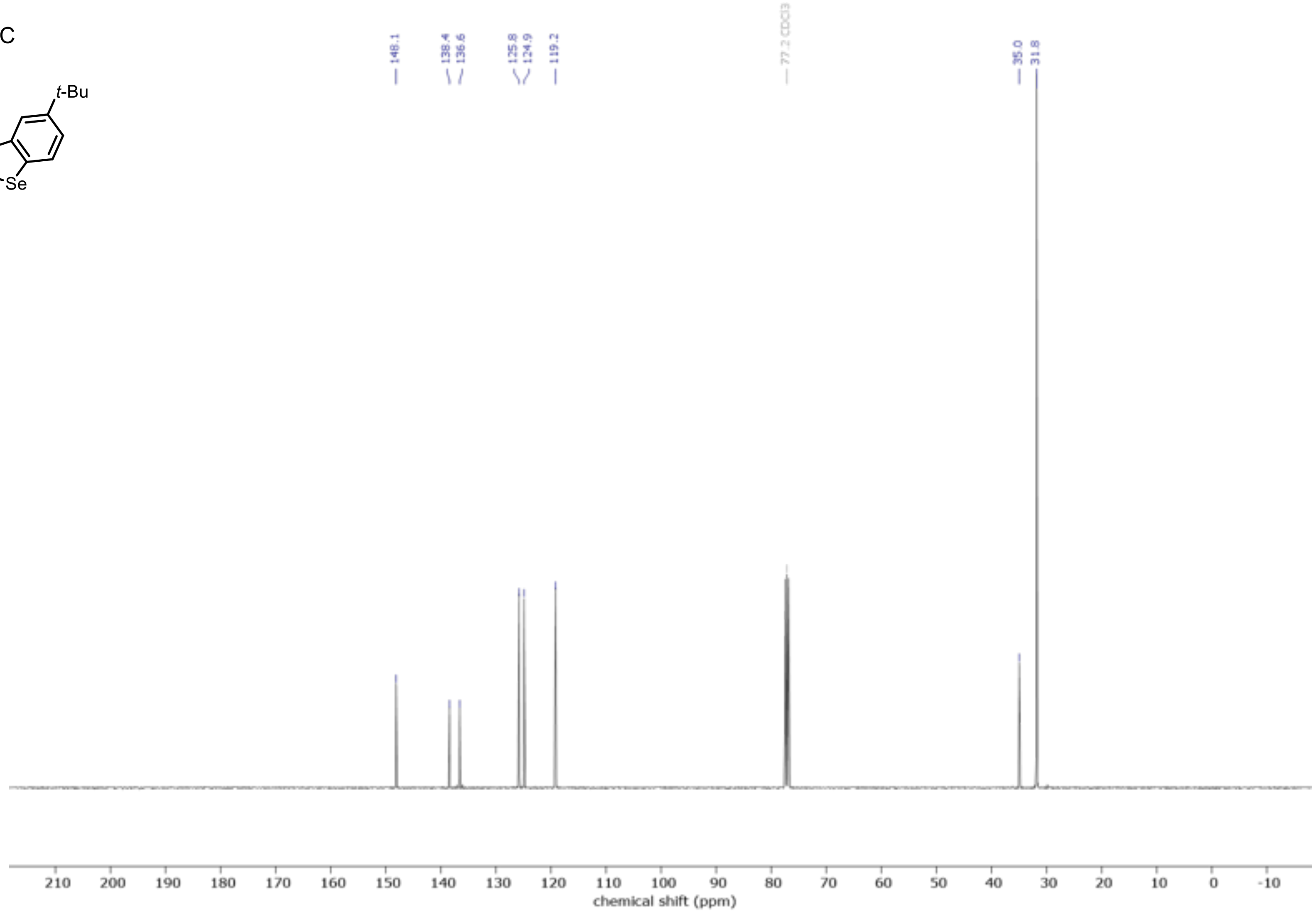

^{77}Se NMR of 2,8-di-*tert*-butyldibenzo[*b*,*d*]selenophene (1c)

CDCl$_3$, 23 ºC

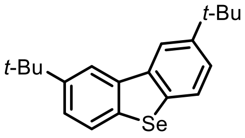

1c

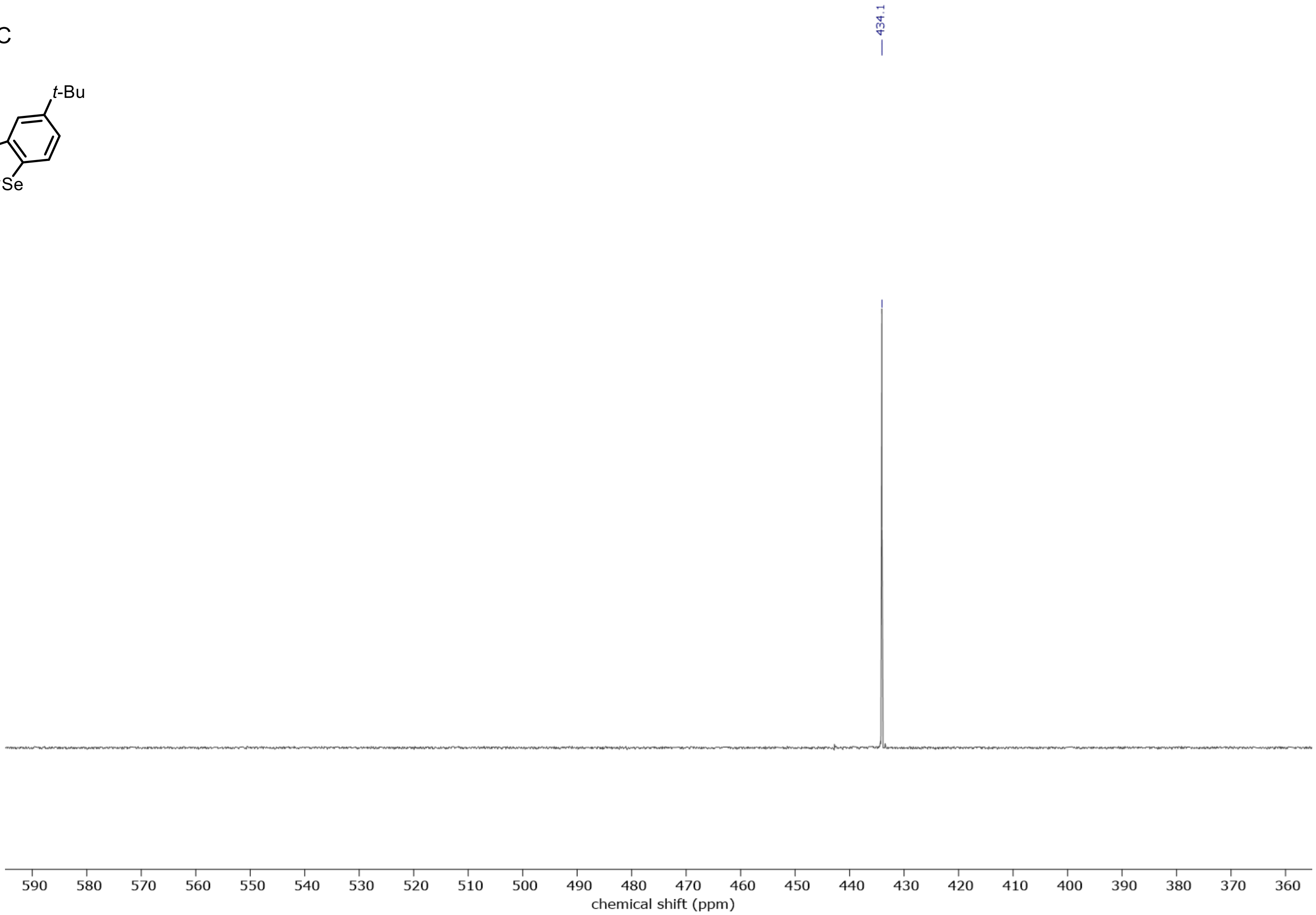

^{1}H NMR OF OCTAMETHYL-OCTAHYDRO DINAPHTHOSELENOPHENE (1D)

CDCl3, 23 ºC

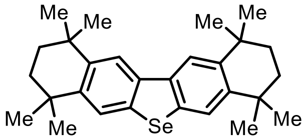

1d

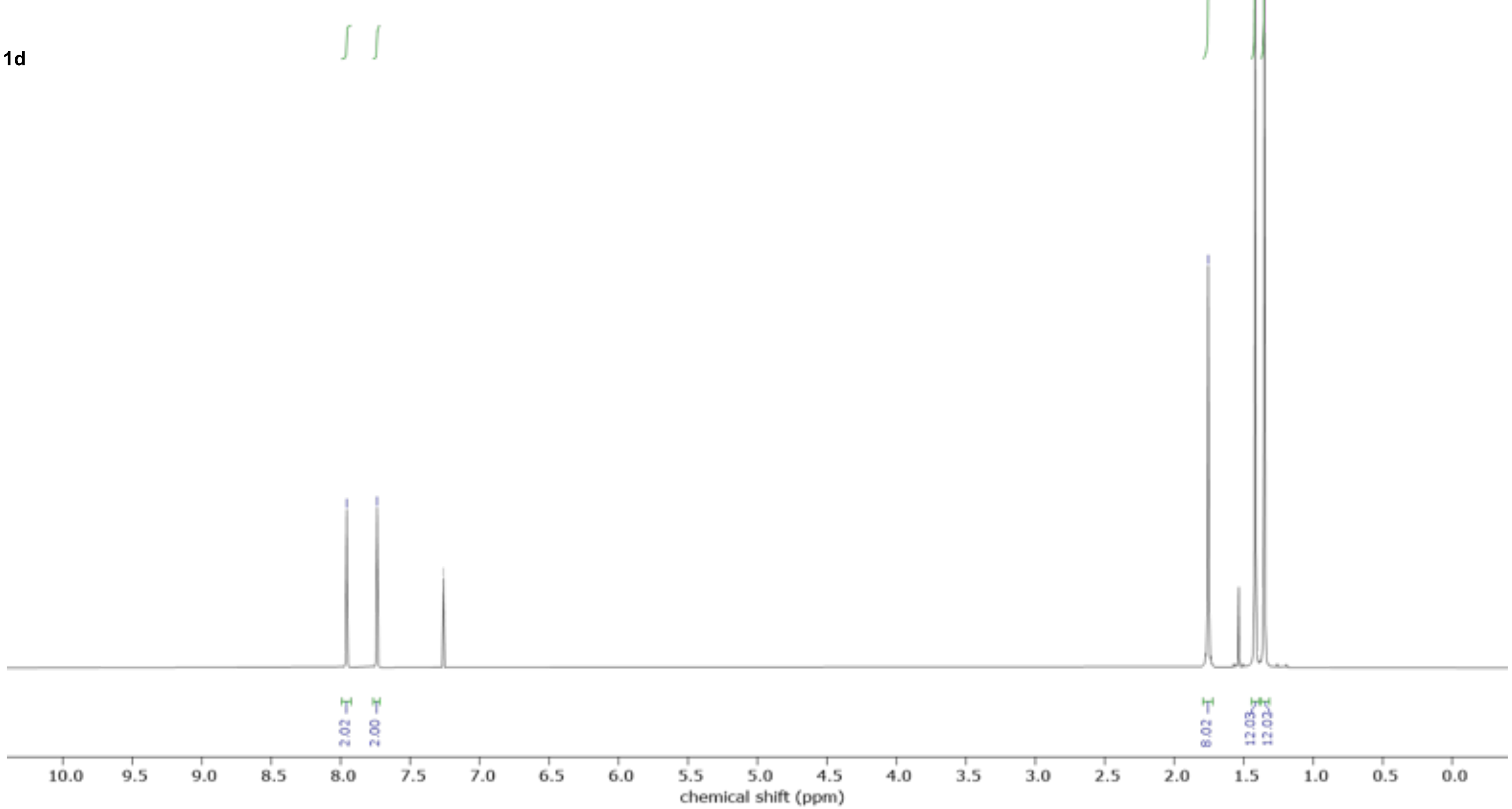

^{13}C NMR OF OCTAMETHYL-OCTAHYDRO DINAPHTHOSELENOPHENE (1D)

CDCl3, 23 ºC

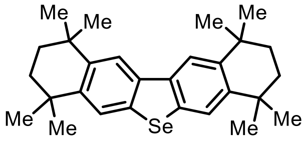

1d

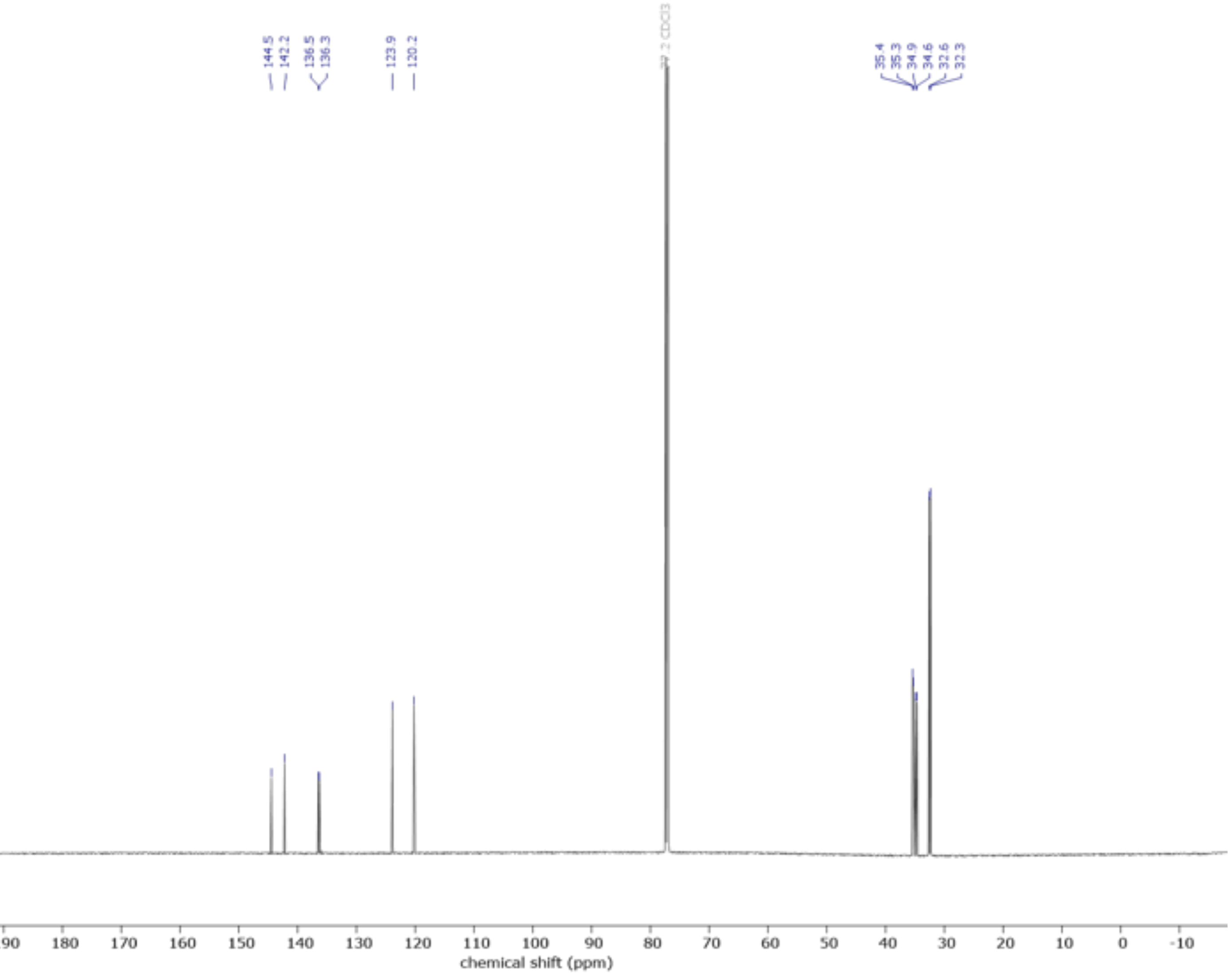

^{77}Se NMR of octamethyl-octahydro dinaphthoselenophene (1d)

CDCl$_3$, 23 ºC

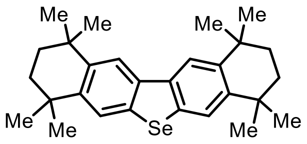

1d

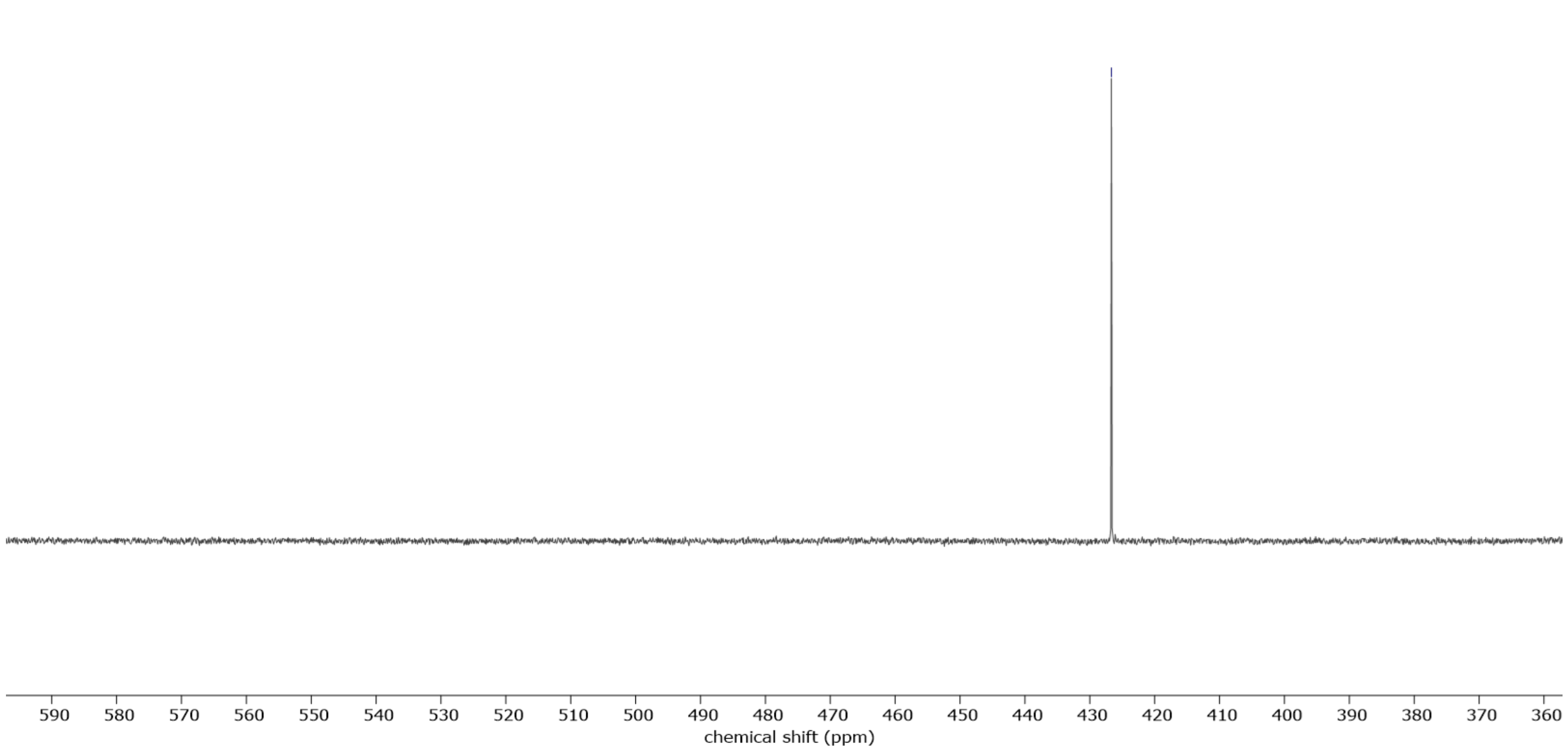

^{1}H NMR of 4-(2,6-diisopropylphenyl)dibenzo[*b*,*d*]selenophene (1e)

CDCl3, 23 ºC

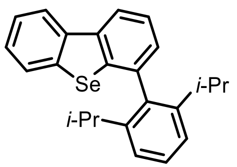

1e

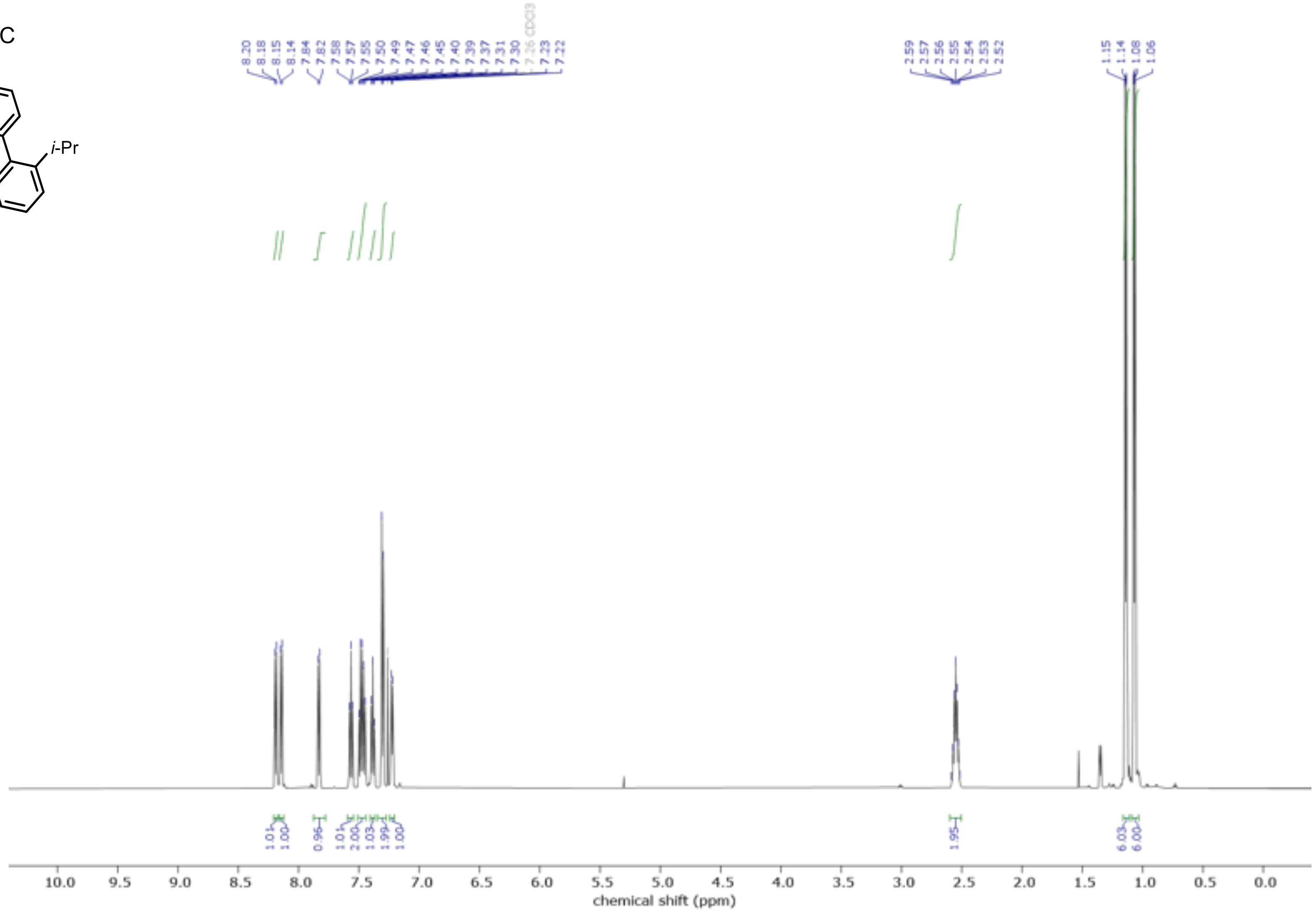

^{13}C NMR OF 4-(2,6-DIISOPROPYLPHENYL)DIBENZO[*B*,*D*]SELENOPHENE (1E)

$CDCl_3$, 23 ºC

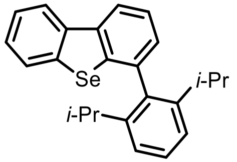

1e

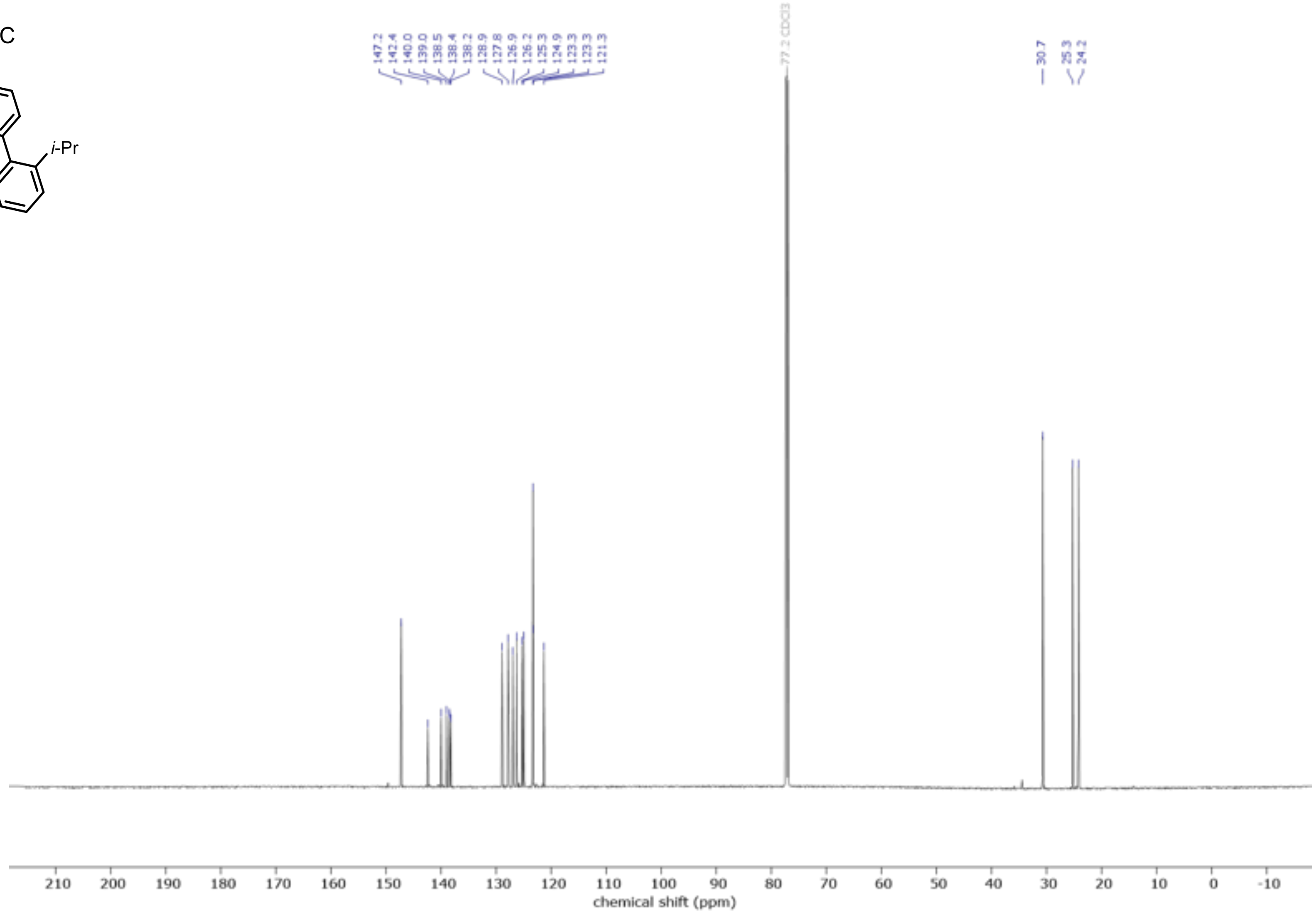

^{77}Se NMR of 4-(2,6-diisopropylphenyl)dibenzo[*b*,*d*]selenophene (1e)

CDCl$_3$, 23 ºC

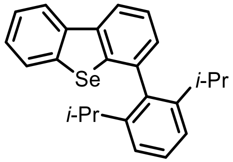

1e

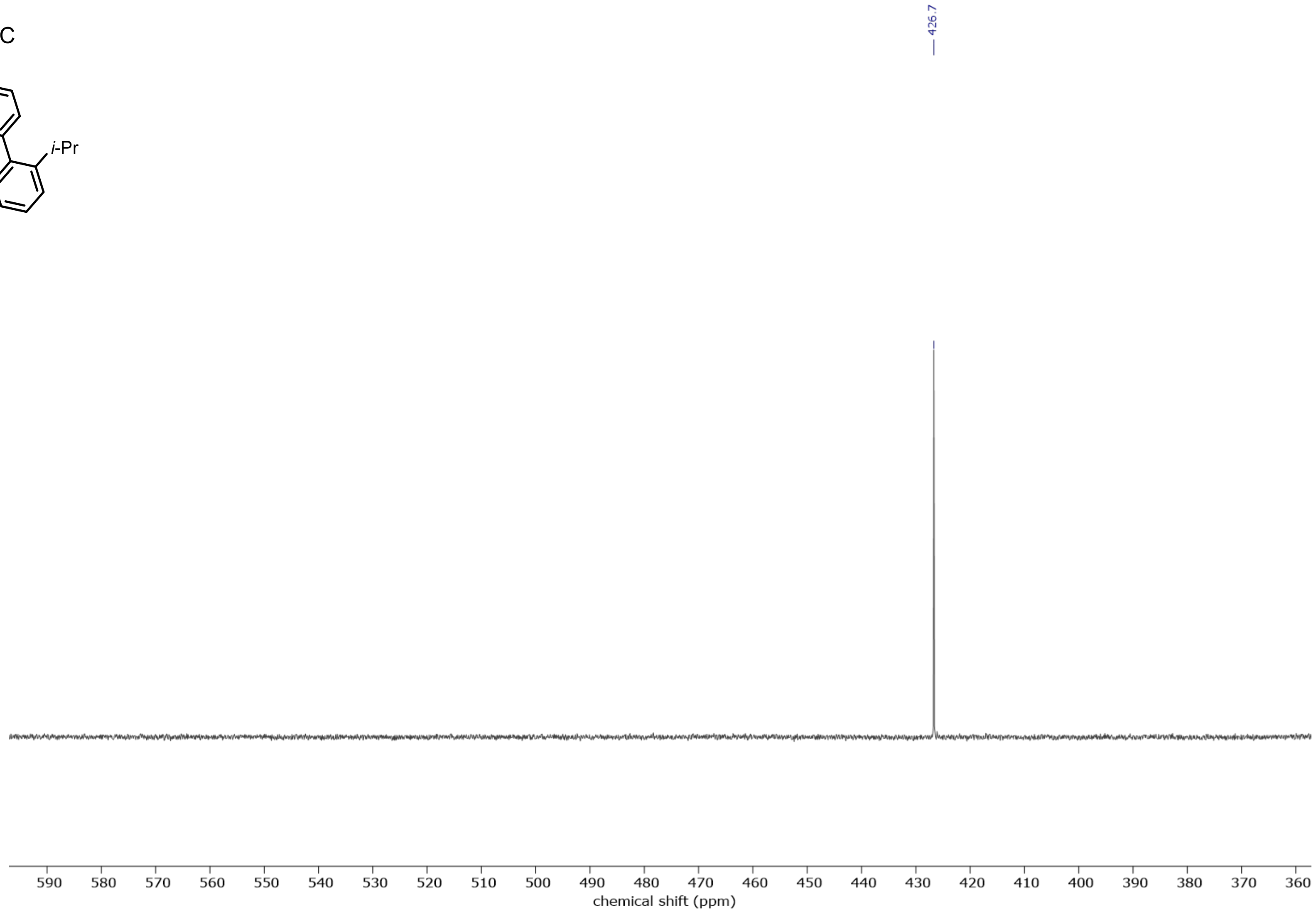

^{1}H NMR of 3,7-bis(trifluoromethyl)dibenzo[*b*,*d*]selenophene (1f)

$CDCl_3$, 23 ºC

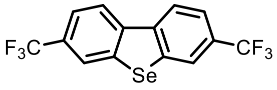

1f

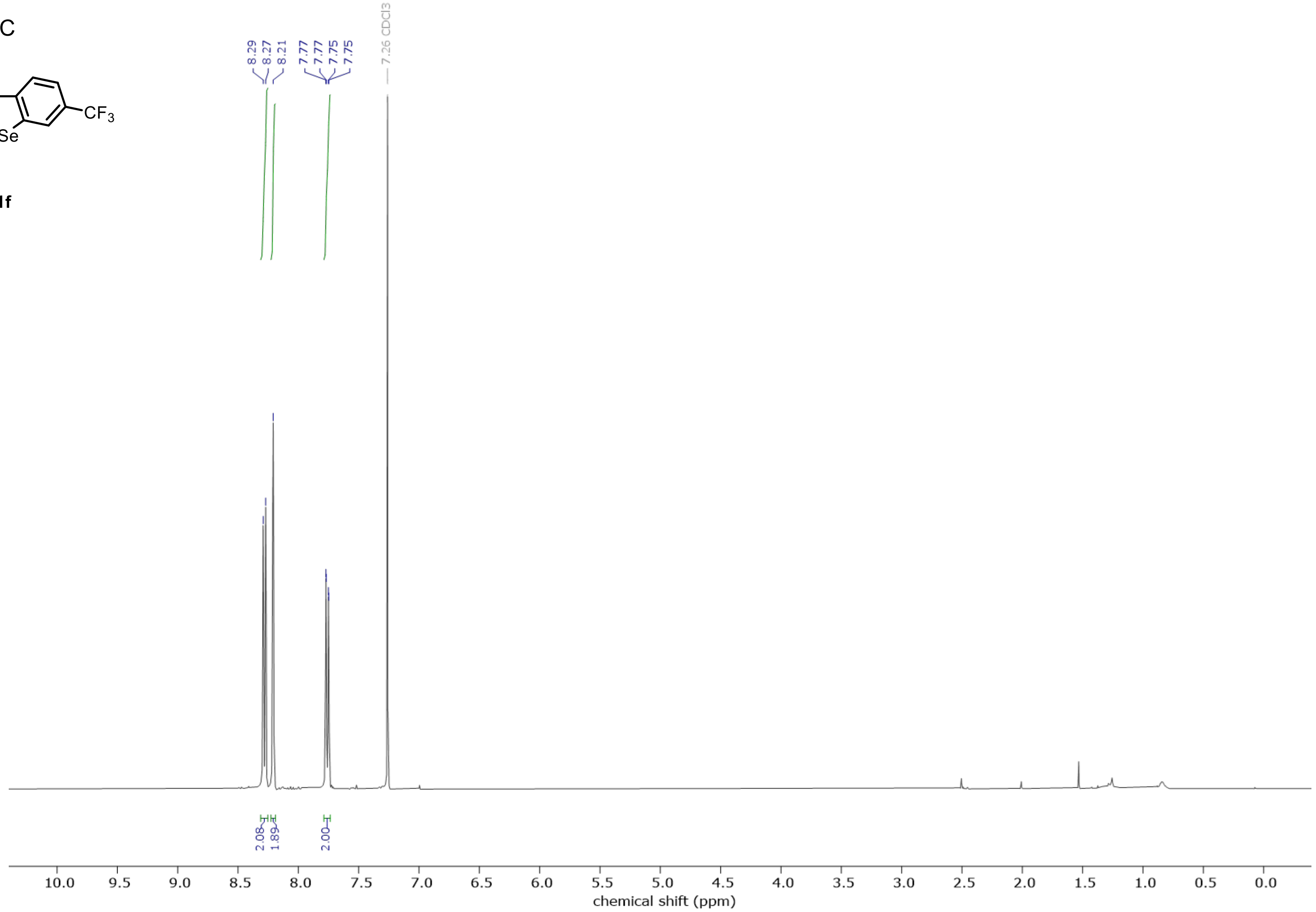

^{13}C OF 3,7-BIS(TRIFLUOROMETHYL)DIBENZO[*B*,*D*]SELENOPHENE (1F)

CDCl$_3$, 23 ºC

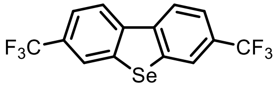

1f

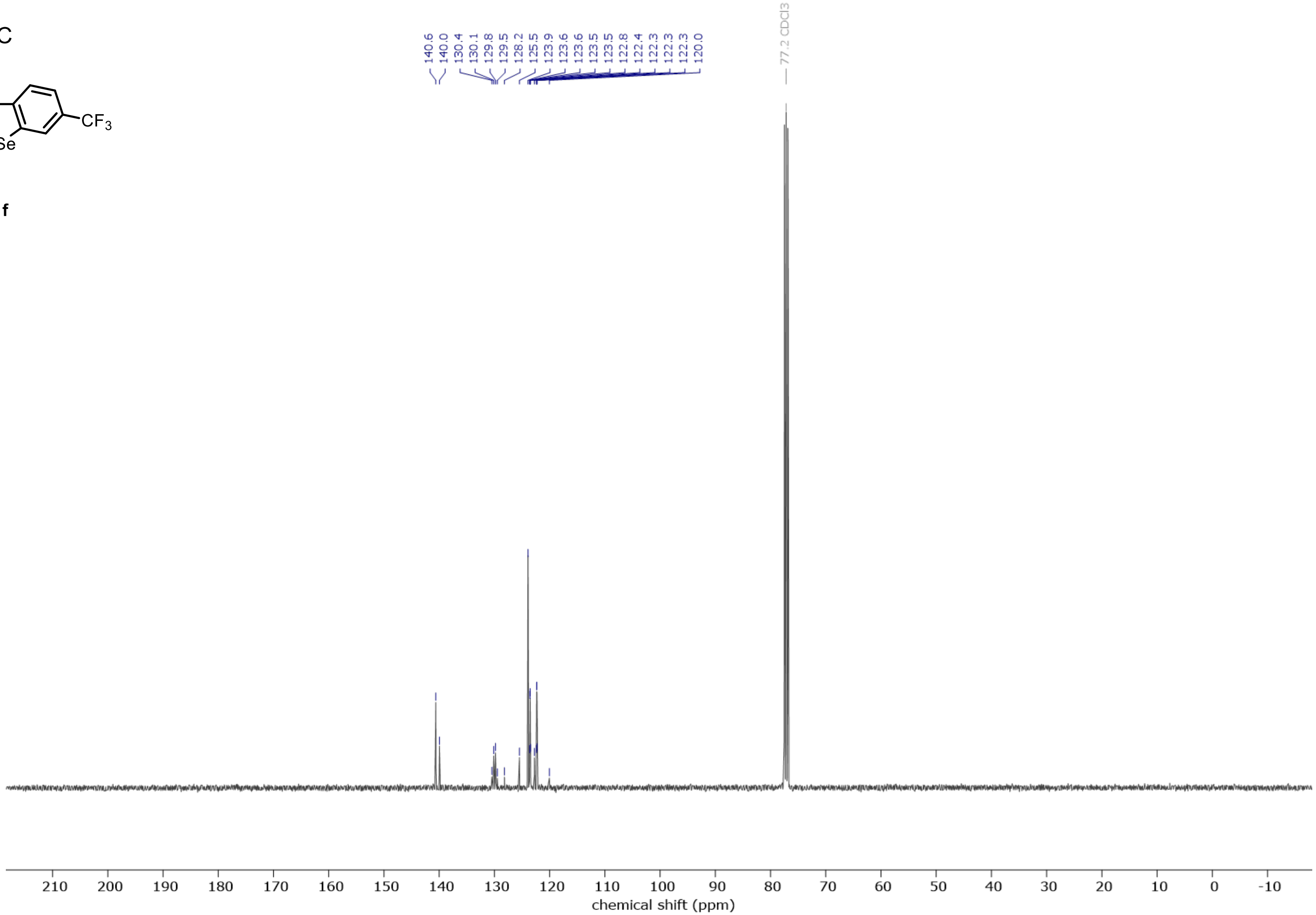

[19]F of 3,7-bis(trifluoromethyl)dibenzo[*b*,*d*]selenophene (1f)

$CDCl_3$, 23 ºC

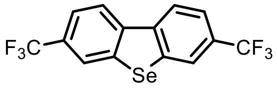

1f

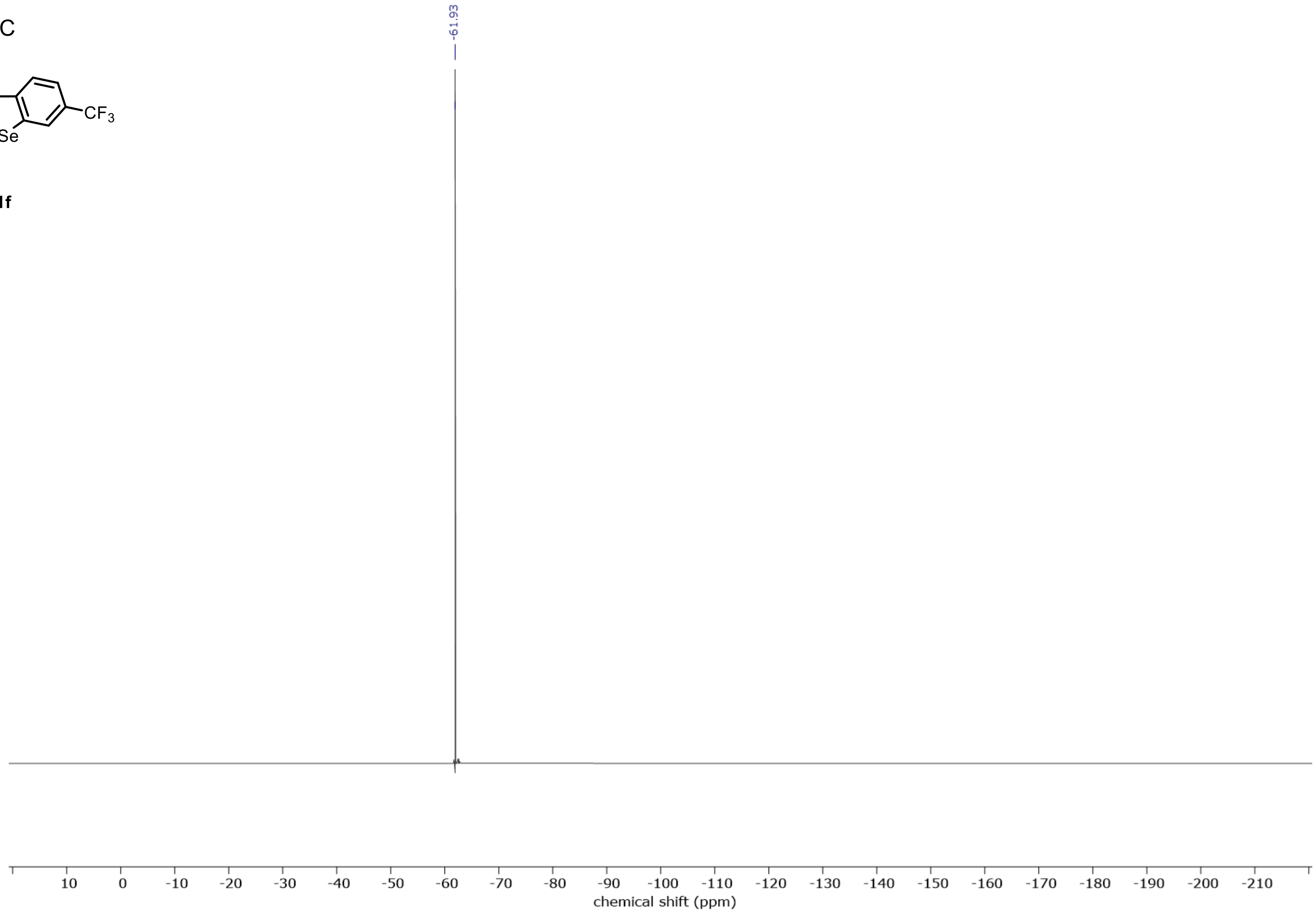

^{77}Se of 3,7-bis(trifluoromethyl)dibenzo[*b*,*d*]selenophene (1f)

CDCl$_3$, 23 °C

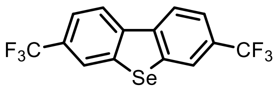

1f

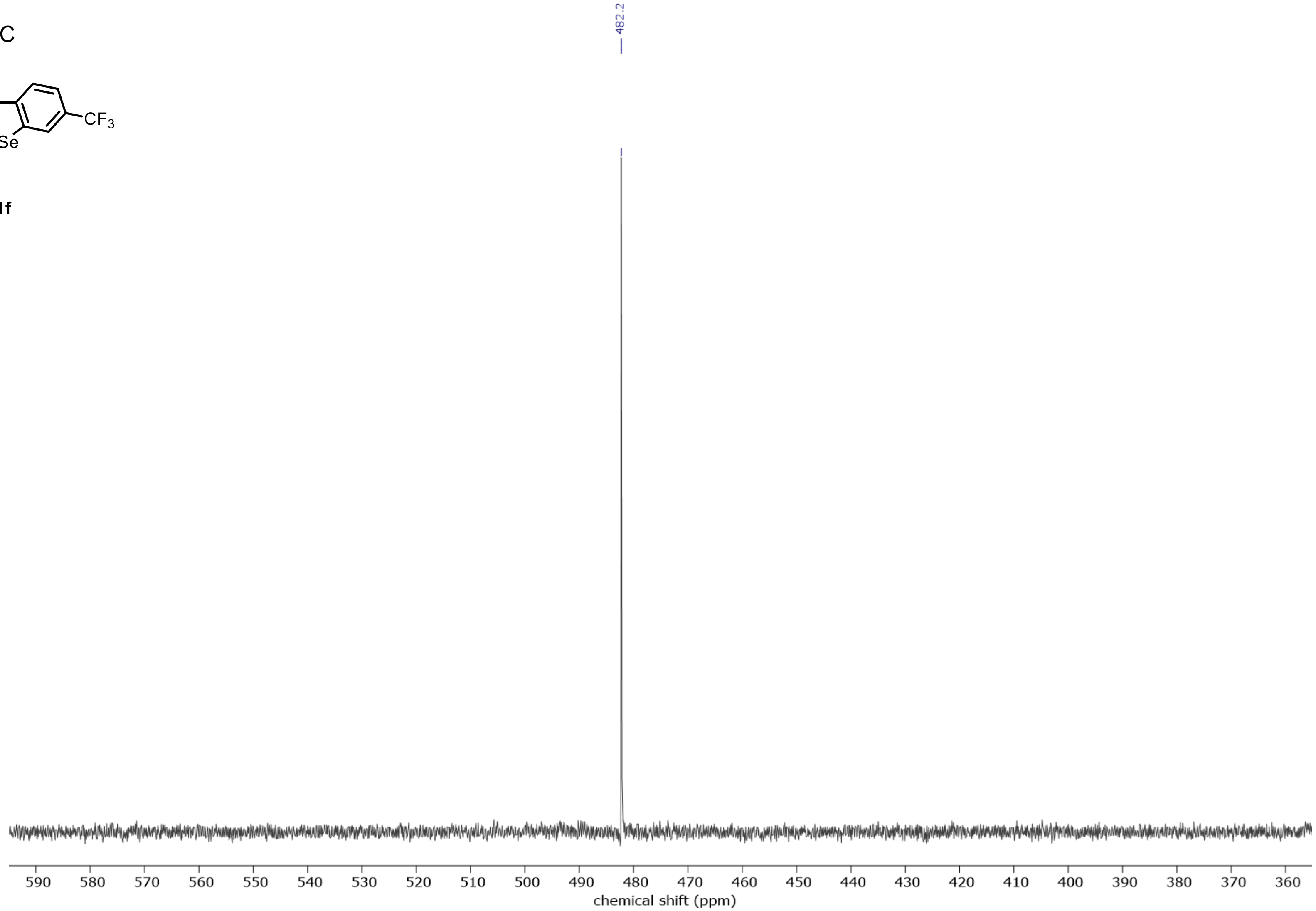

^{1}H NMR of [2,5'-bidibenzoselenophen]-5'-ium trifluoromethanesulfonate (Se–[5a]OTf)

DMSO-d_6, 23 ºC

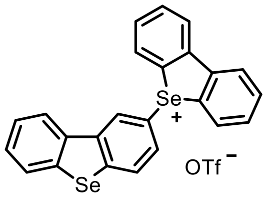

Se–[5a]OTf

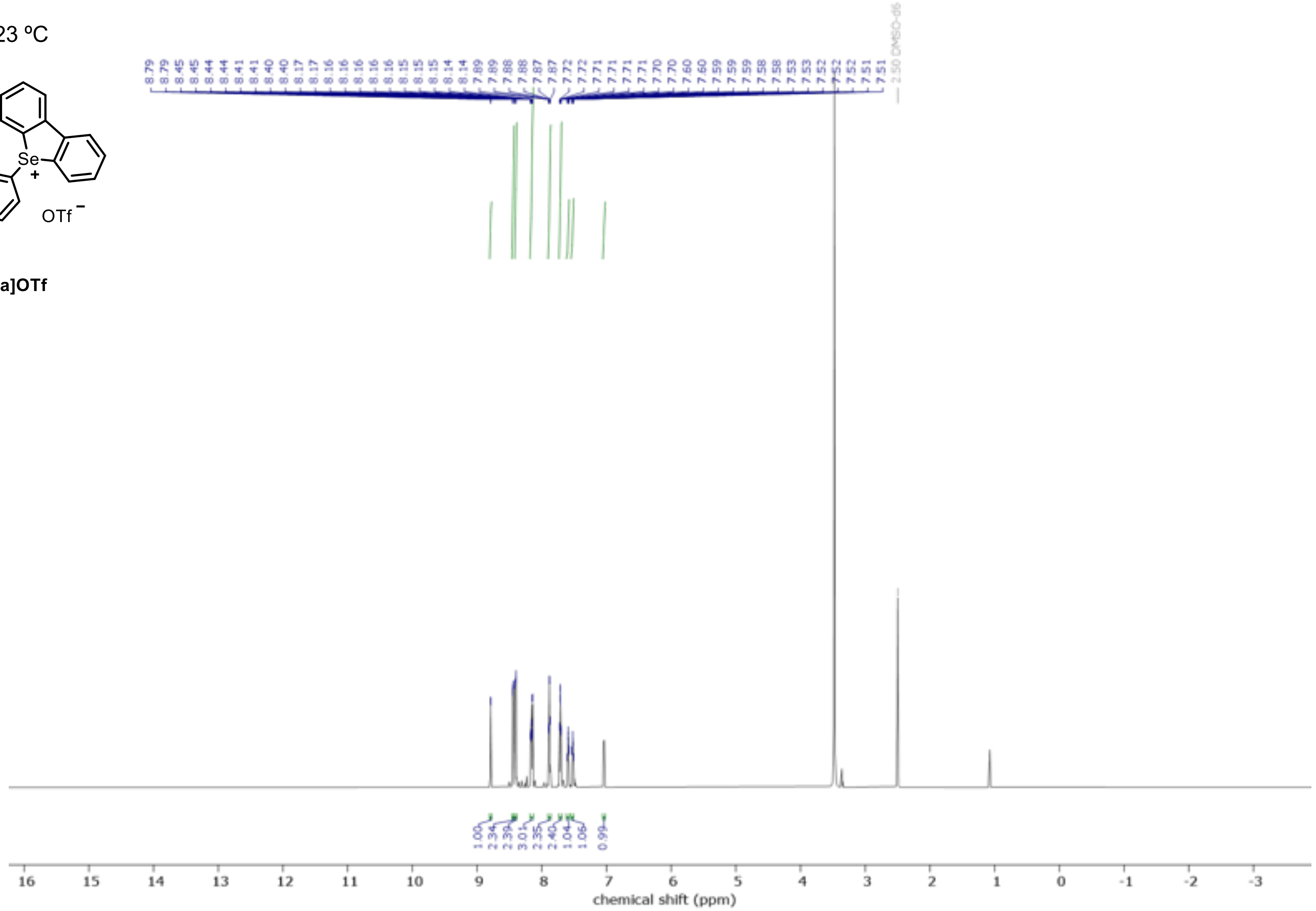

^{1}H NMR OF [2,5'-BIDIBENZOSELENOPHEN]-5'-IUM TRIFLUOROMETHANESULFONATE (SE–[5A]OTF)

CD3CN, 23 ºC

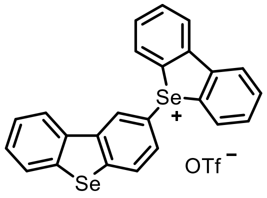

Se–[5a]OTf

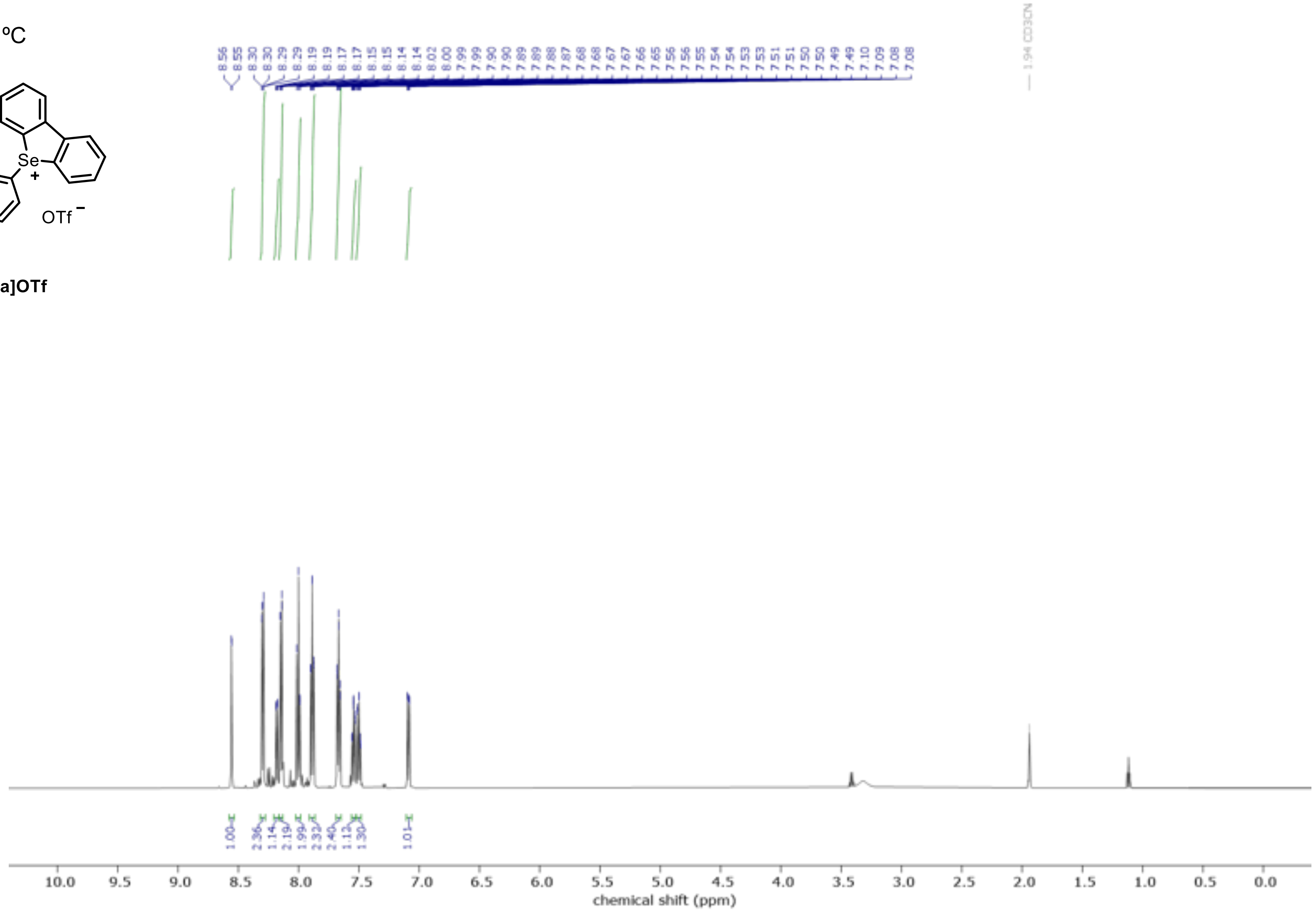

^{13}C NMR OF [2,5'-BIDIBENZOSELENOPHEN]-5'-IUM TRIFLUOROMETHANESULFONATE (SE–[5A]OTF)

DMSO-d6, 23 °C

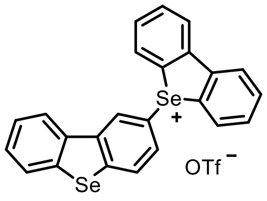

Se–[5a]OTf

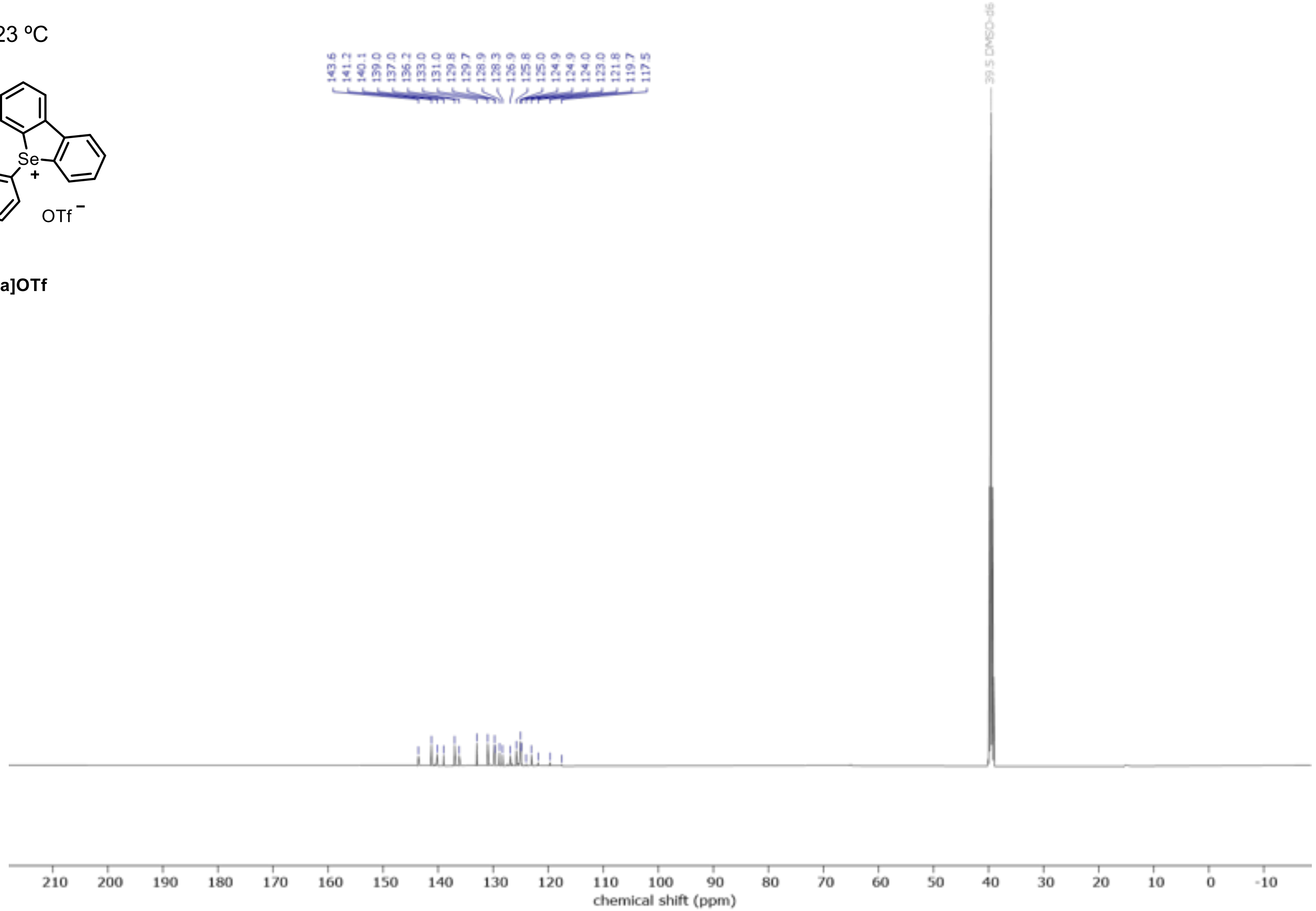

^{19}F NMR OF [2,5'-BIDIBENZOSELENOPHEN]-5'-IUM TRIFLUOROMETHANESULFONATE (SE–[5A]OTF)

DMSO-d6, 23 ºC

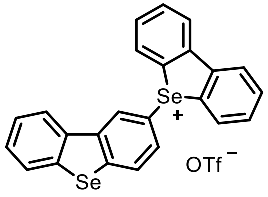

Se–[5a]OTf

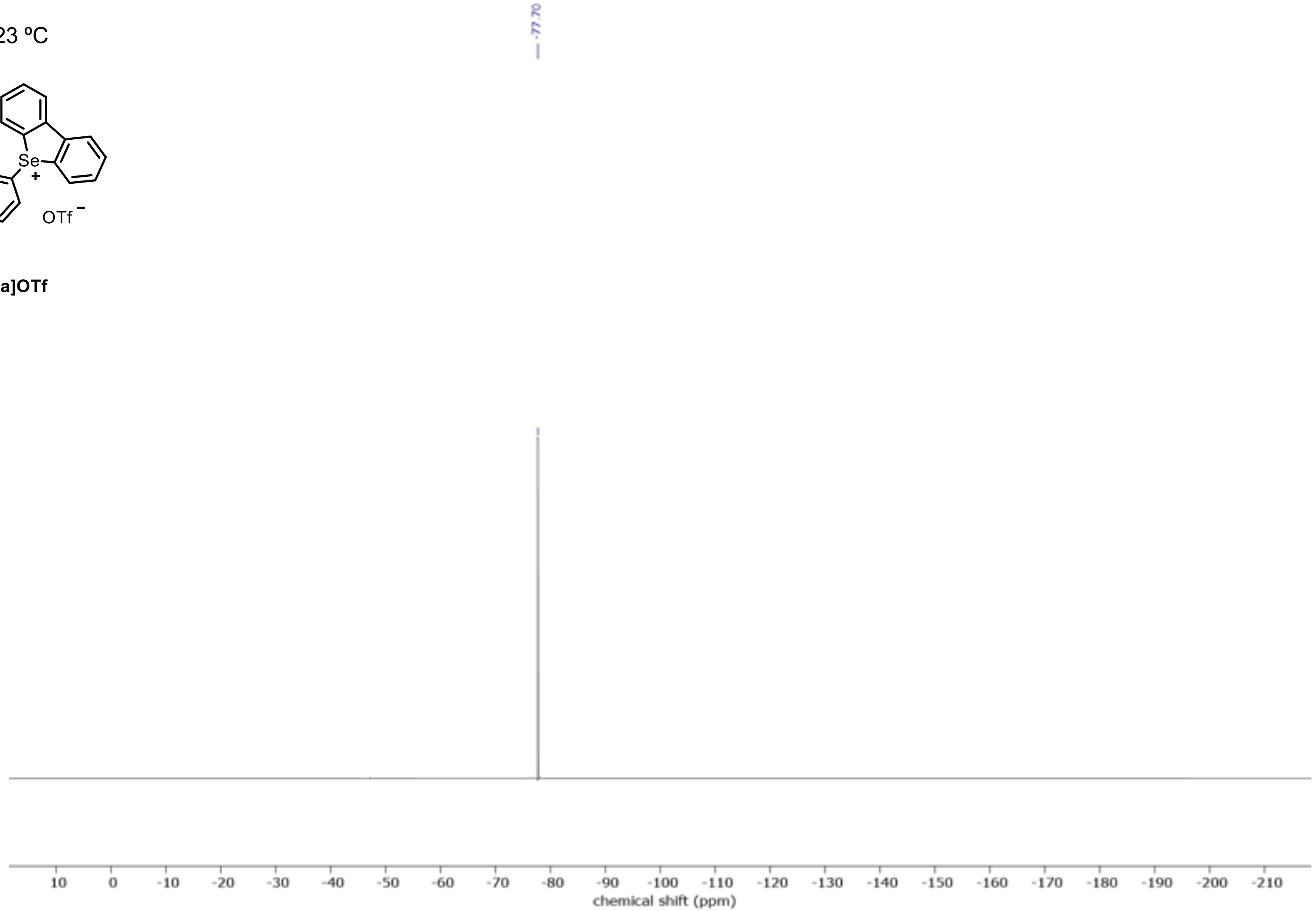

^{77}Se NMR of [2,5'-bidibenzoselenophen]-5'-ium trifluoromethanesulfonate Se–[5a]OTf

CD$_3$CN, 23 ºC

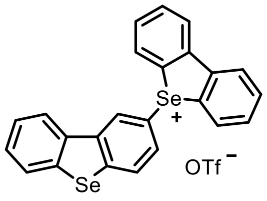

Se–[5a]OTf

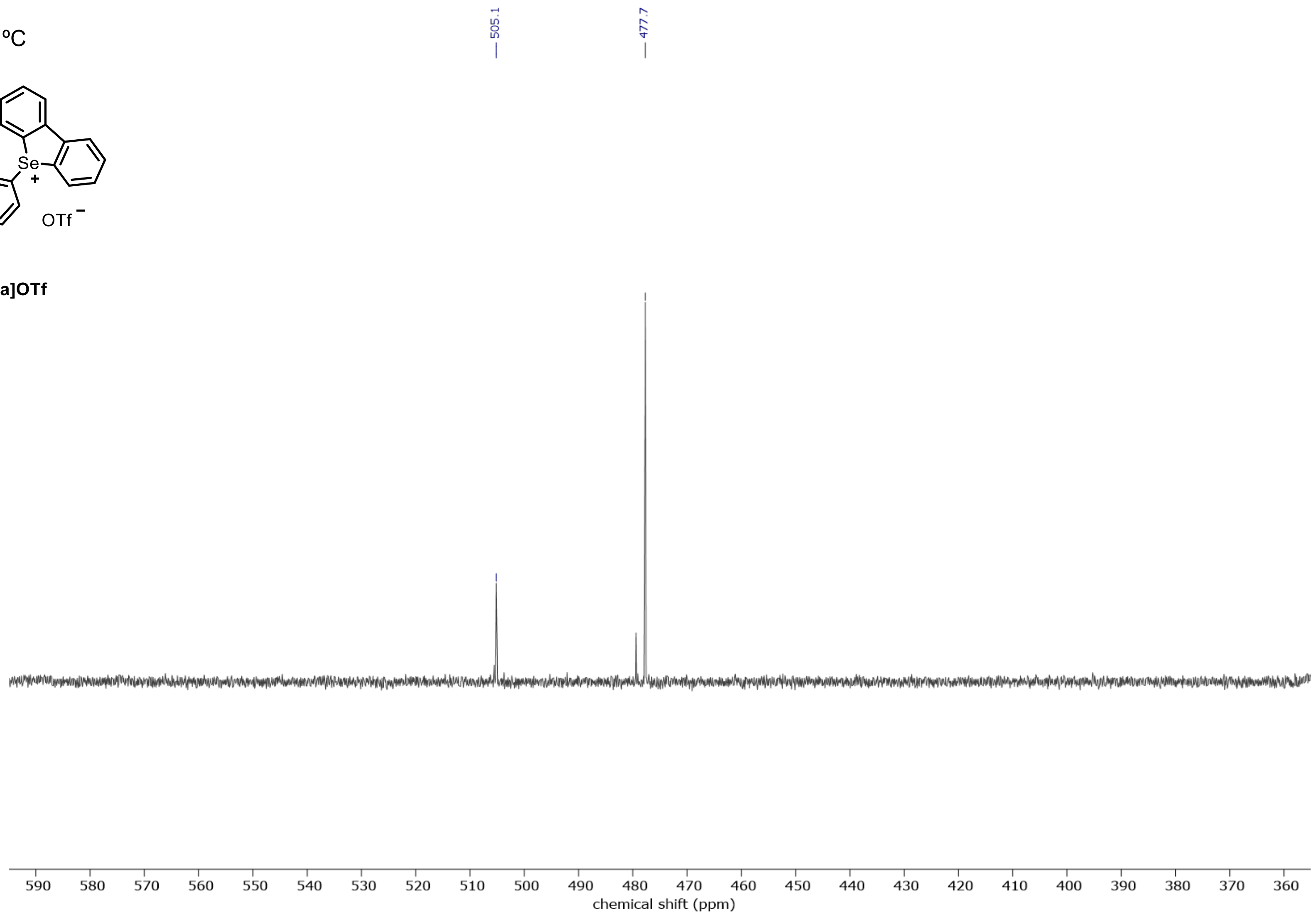

^{1}H NMR OF (*E*)-1,2-DIPHENYLDIAZENE (7)

$CDCl_3$, 23 °C

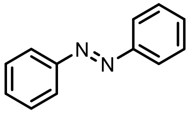

7

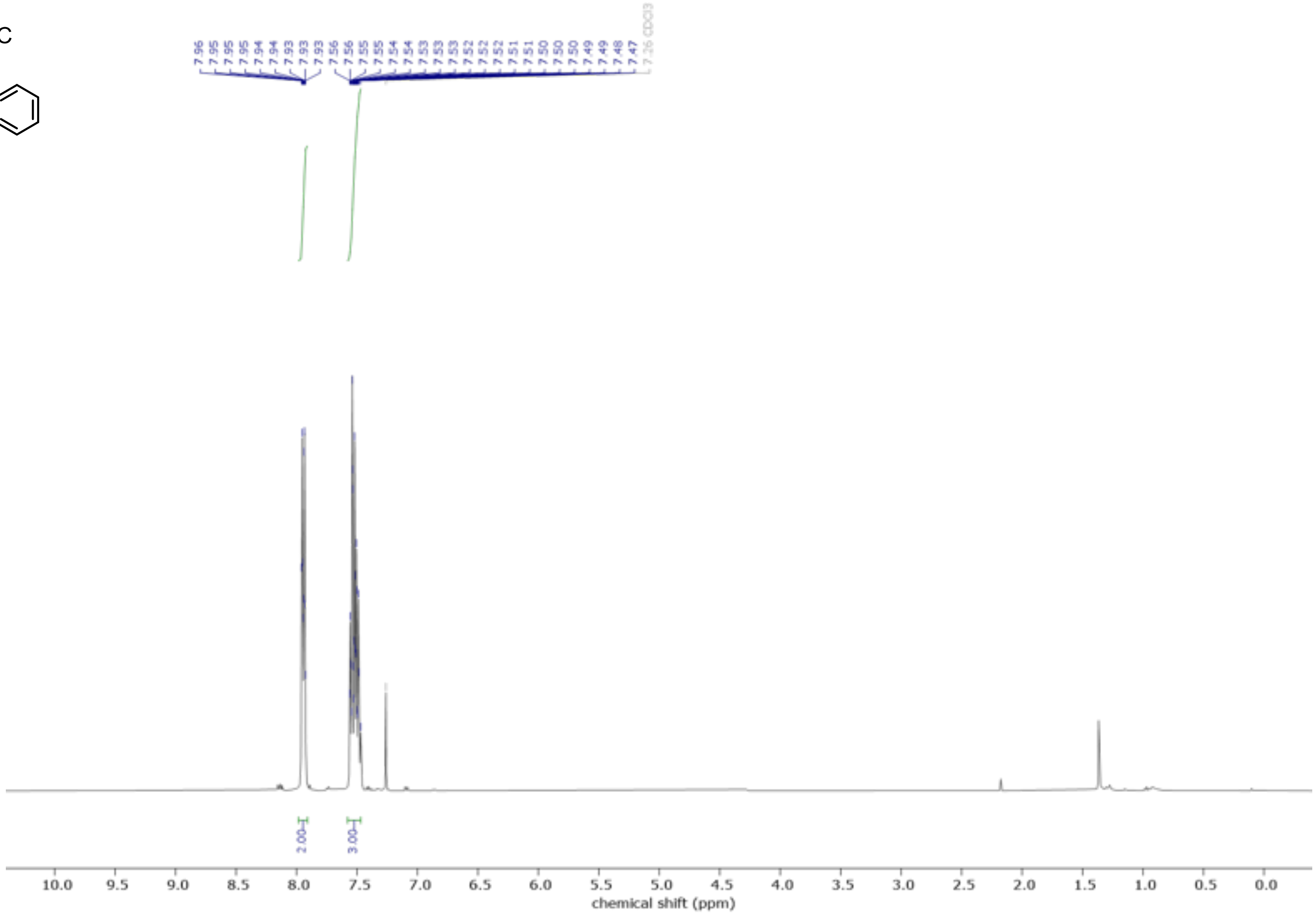

^{13}C NMR of (*E*)-1,2-diphenyldiazene (7)

CDCl$_3$, 23 ºC

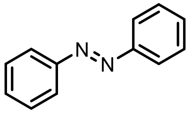

7

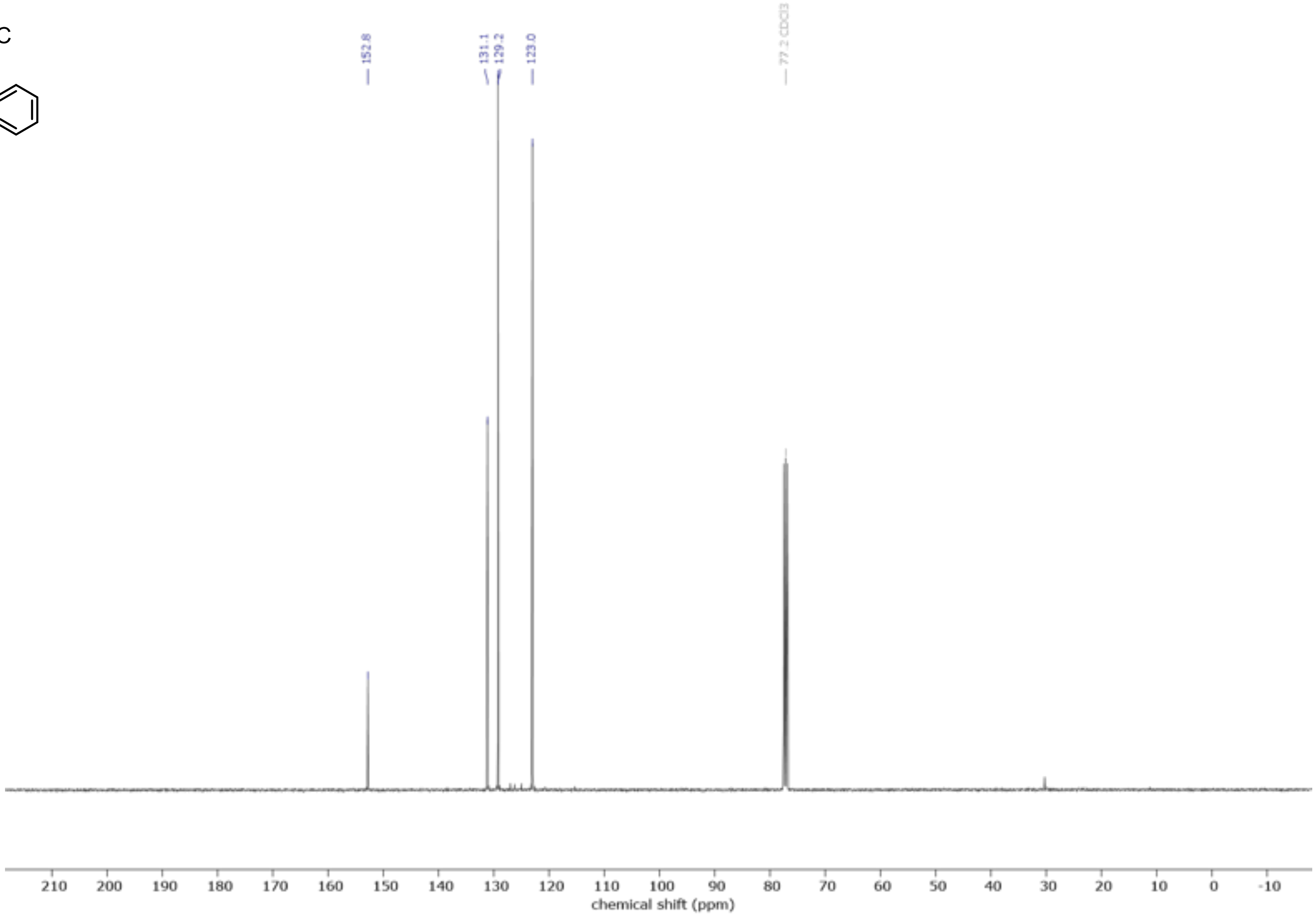

^{1}H NMR OF FLUORENONE (9)

CDCl3, 23 ºC

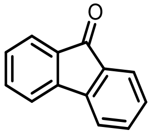

9

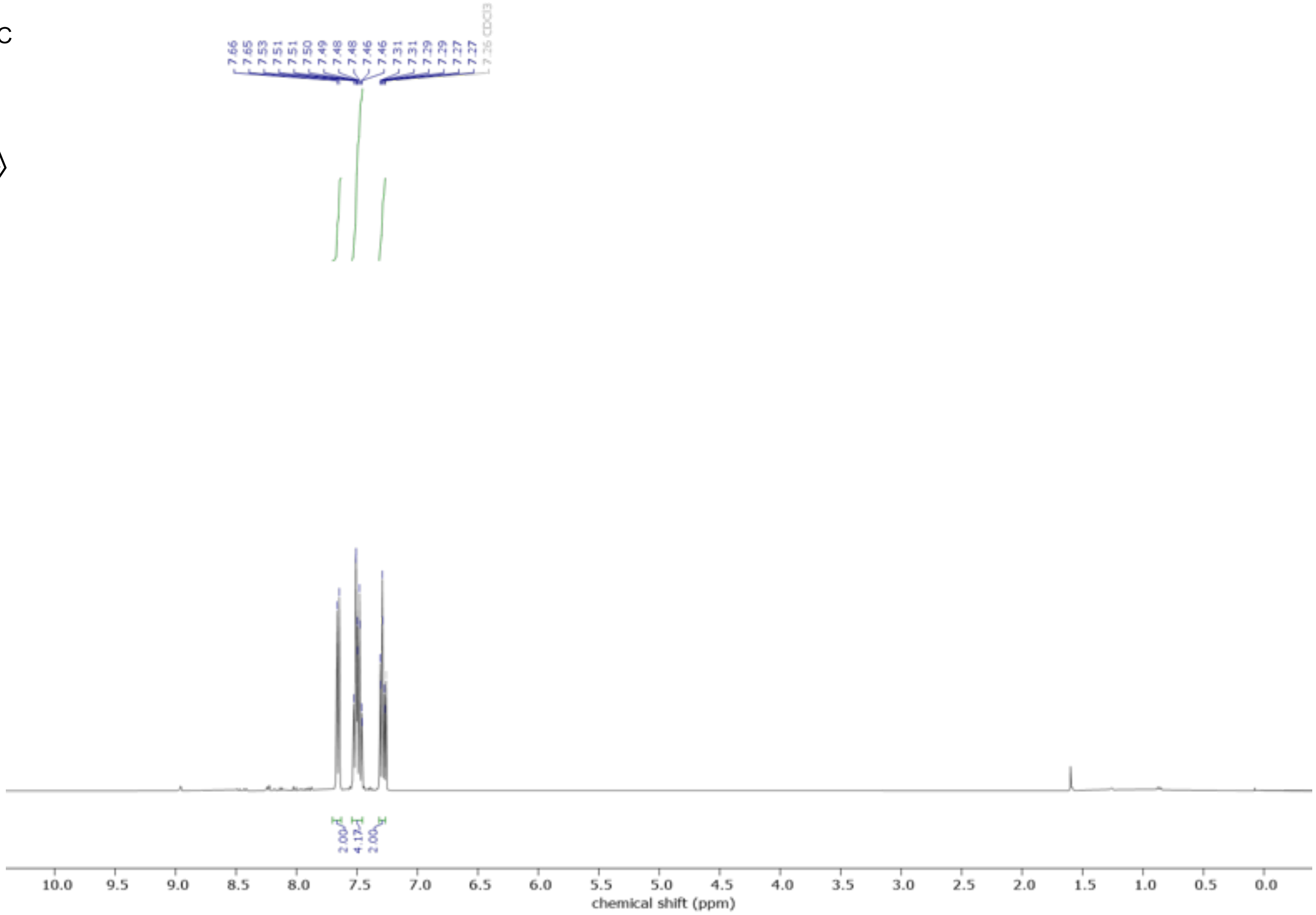

^{13}C NMR OF FLUORENONE (9)

CDCl$_3$, 23 ºC

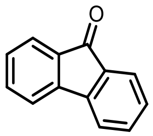

9

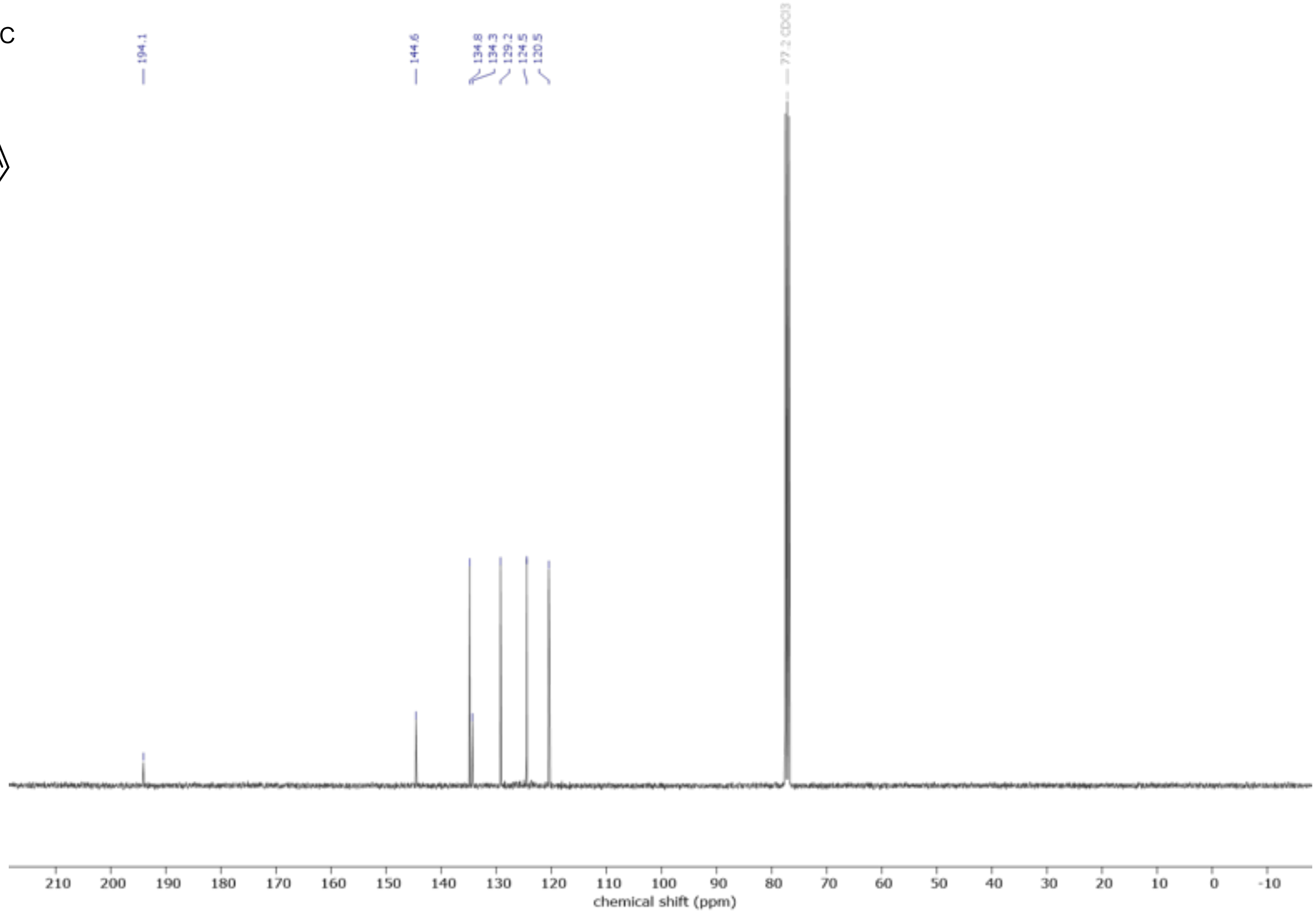

^{1}H NMR OF 5-(TRIFLUOROMETHYL) -DIBENZOSELENOPHENIUM HEXAFLUOROANTIMONATE (10)

CD_3CN, 23 ºC

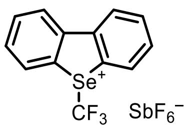

10

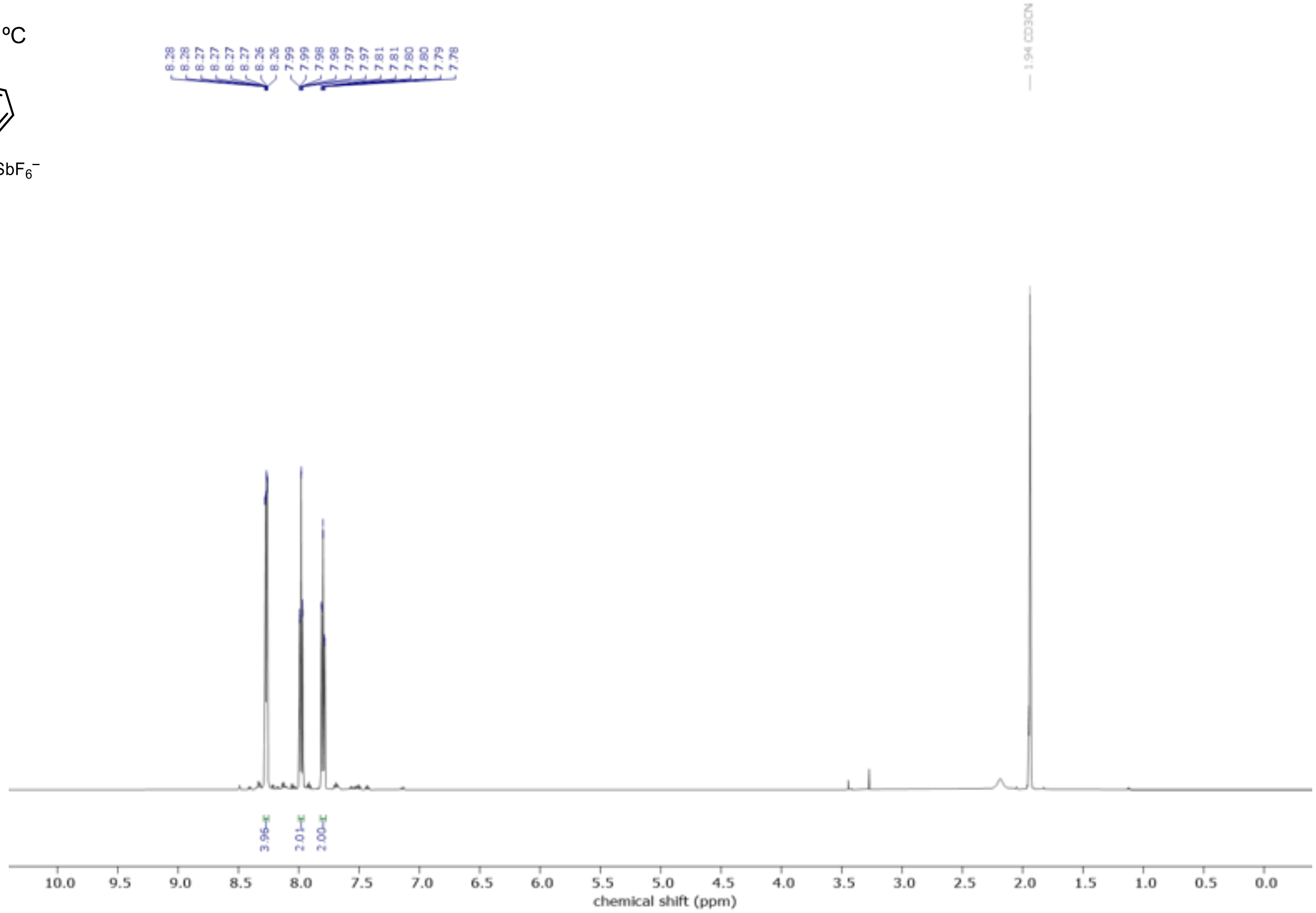

^{13}C NMR of 5-(trifluoromethyl) -dibenzoselenophenium hexafluoroantimonate (10)

CD_3CN, 23 ºC

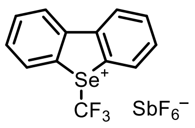

10

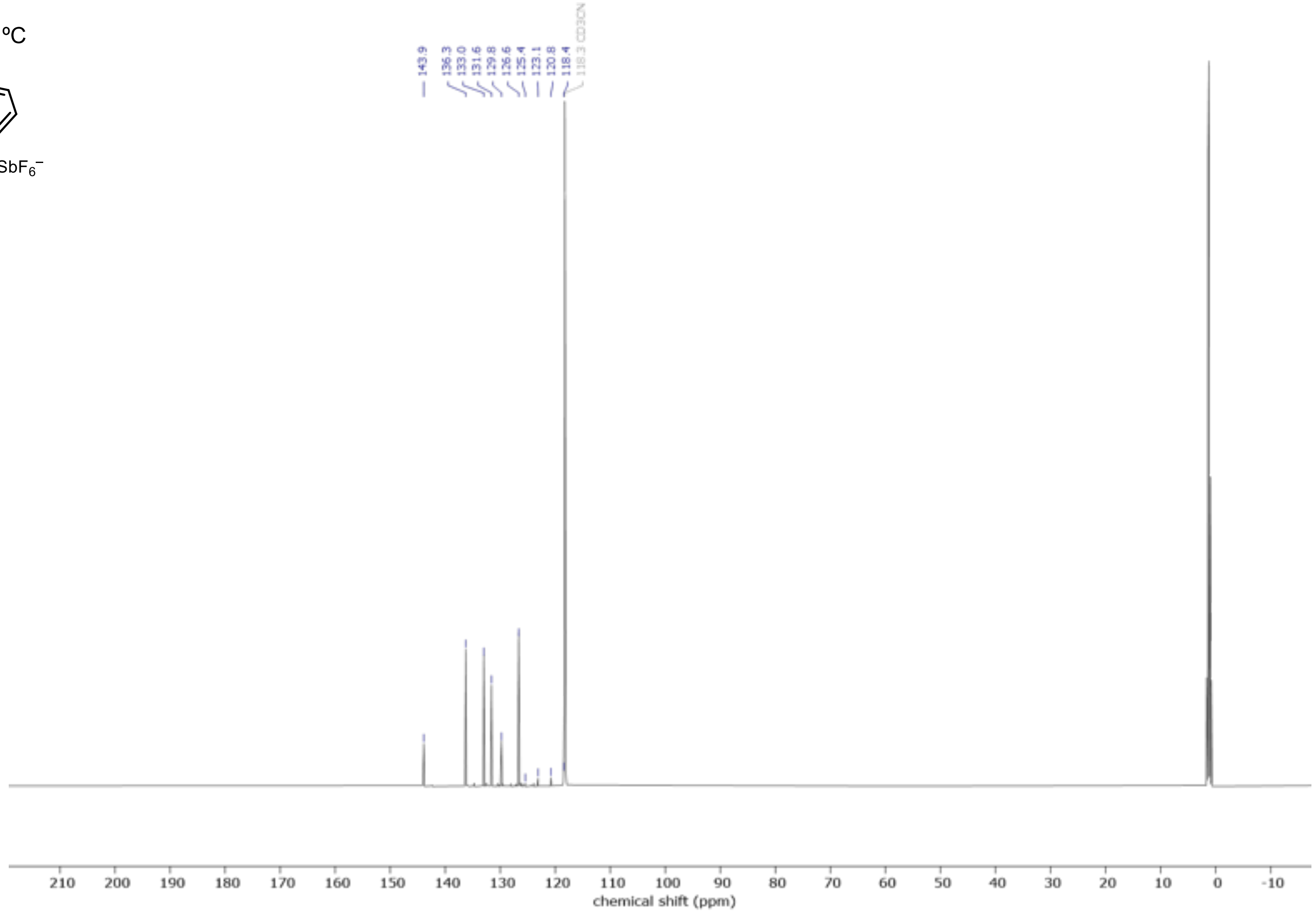

^{19}F NMR of 5-(trifluoromethyl) -dibenzoselenophenium hexafluoroantimonate (10)

CD_3CN, 23 ºC

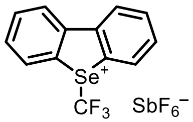

10

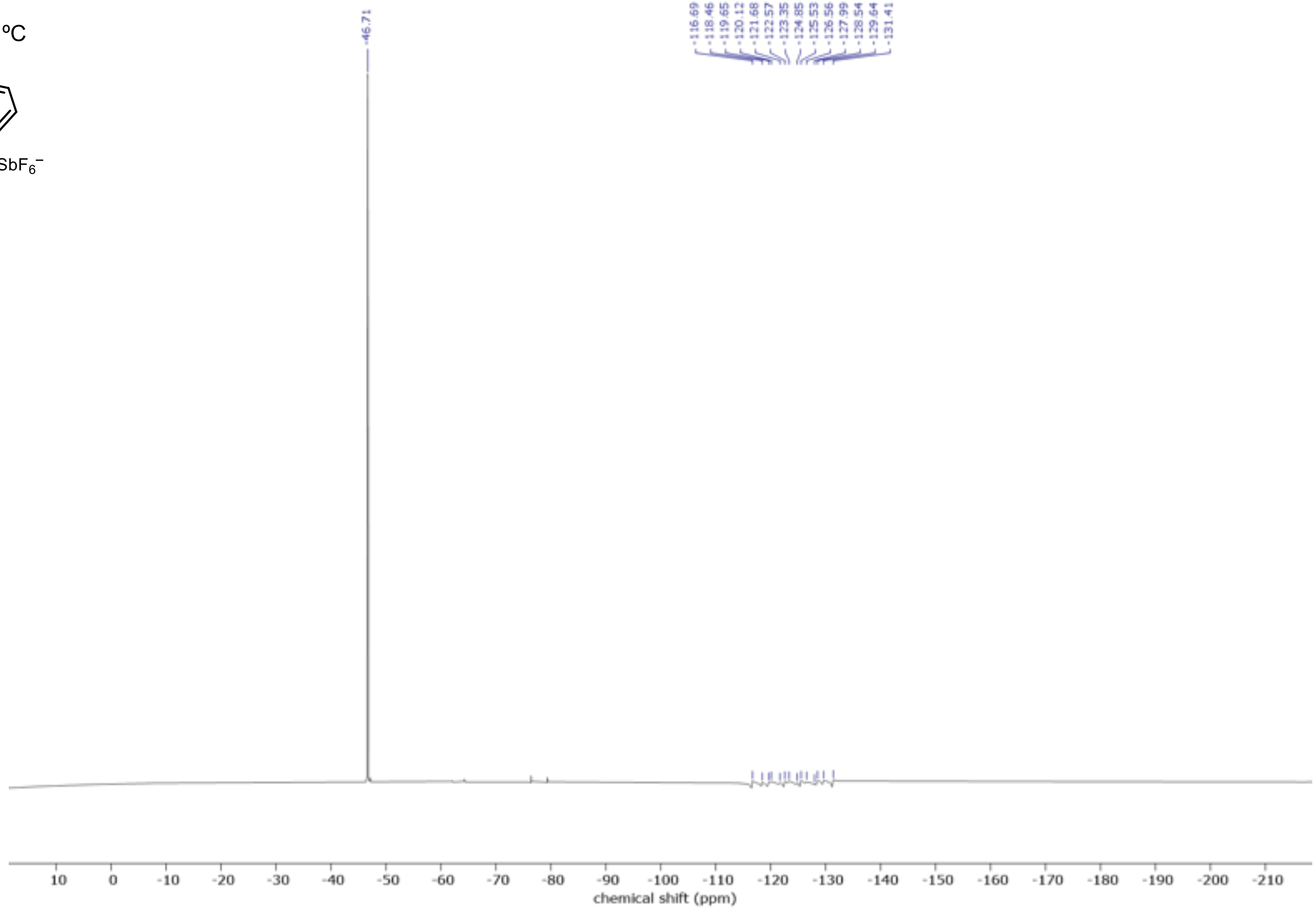

^{77}Se NMR of 5-(trifluoromethyl) -dibenzoselenophenium hexafluoroantimonate (10)

CD_3CN, 23 ºC

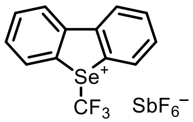

10

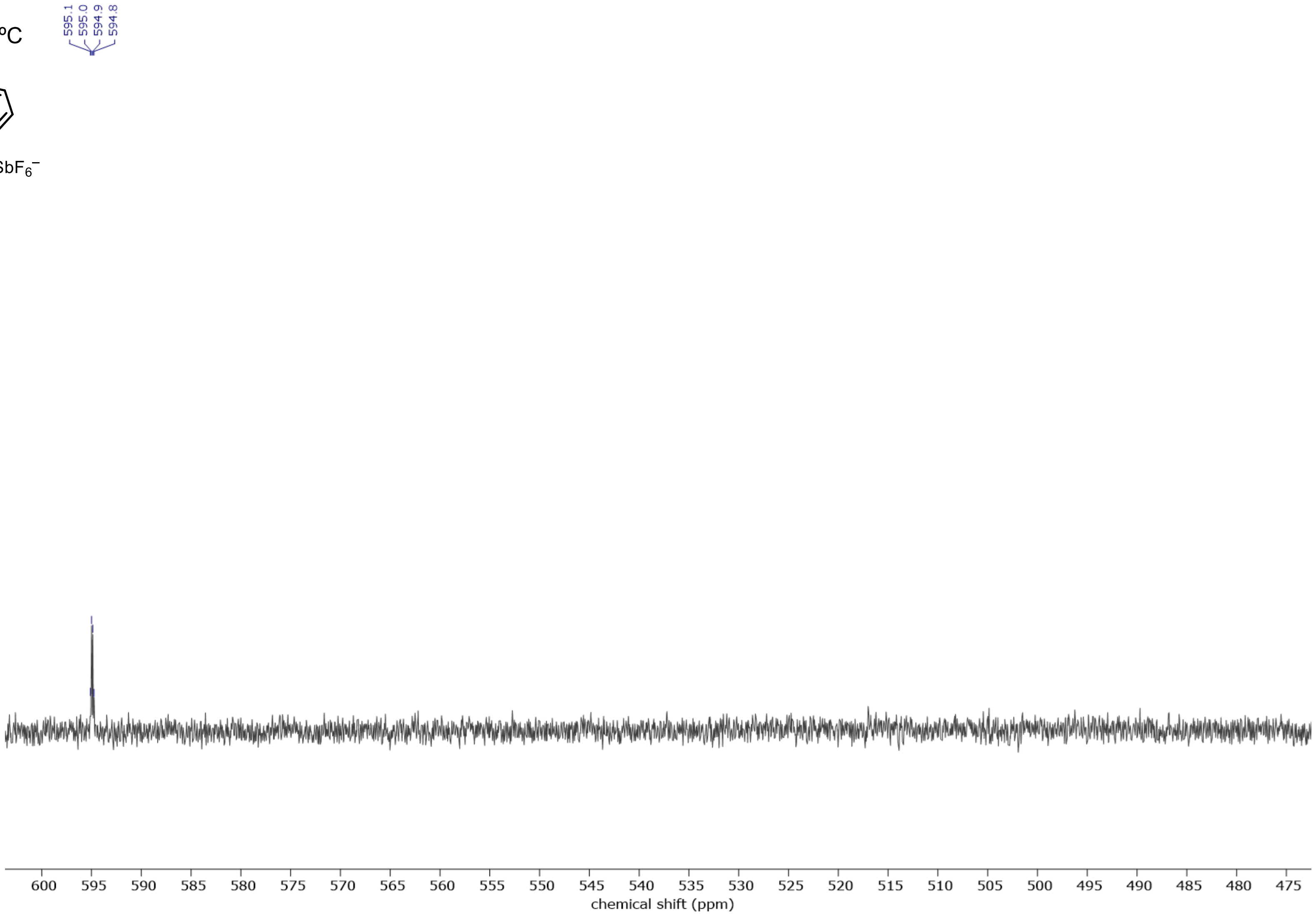

^{1}H NMR OF 5-(PHENYL) -DIBENZOSELENOPHENIUM HEXAFLUOROANTIMONATE (11)

DMSO-d6, 23 ºC

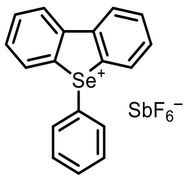

11

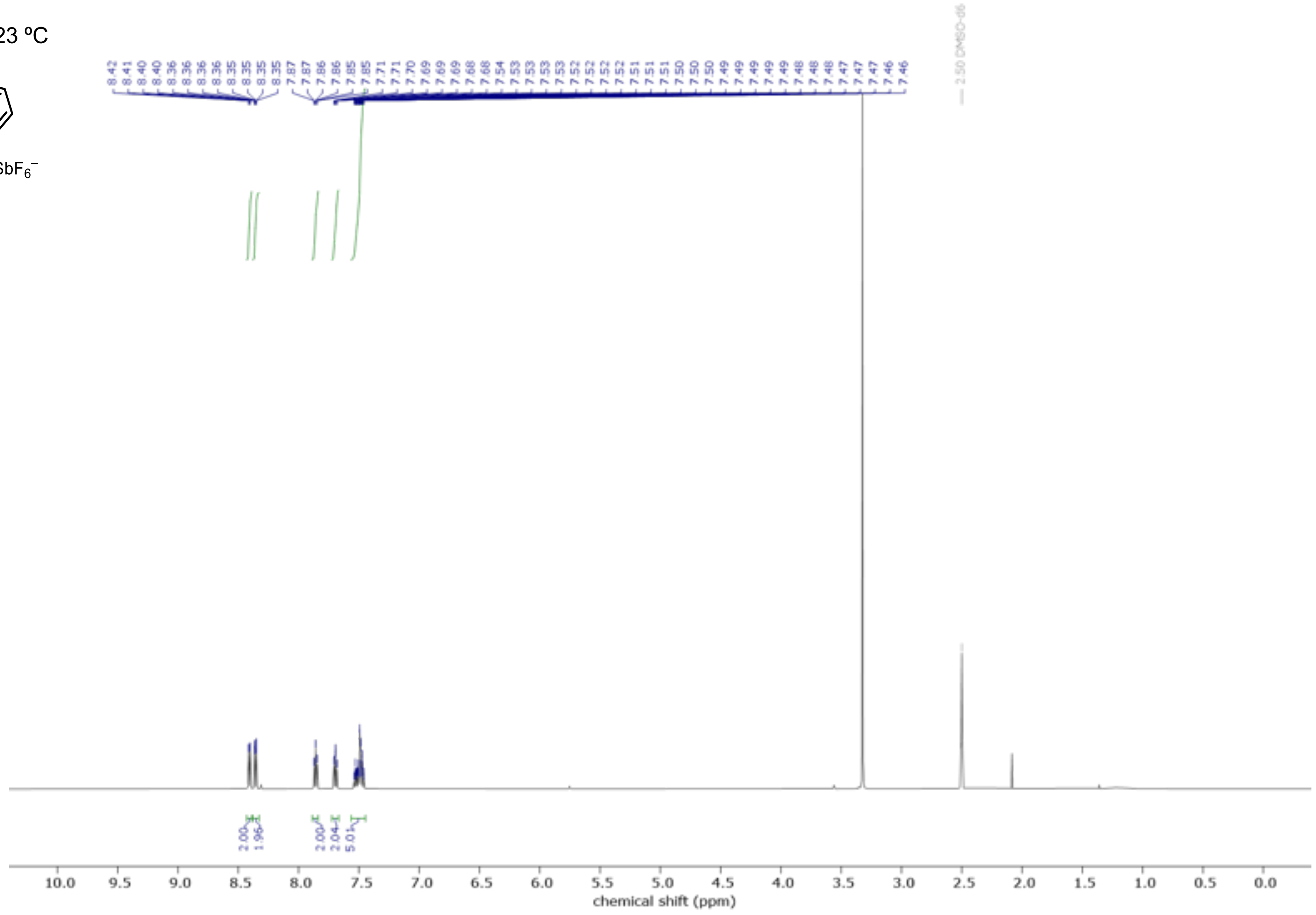

^{13}C NMR OF 5-(PHENYL) -DIBENZOSELENOPHENIUM HEXAFLUOROANTIMONATE (11)

DMSO-d6, 23 ºC

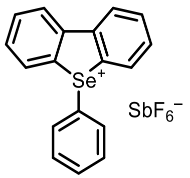

11

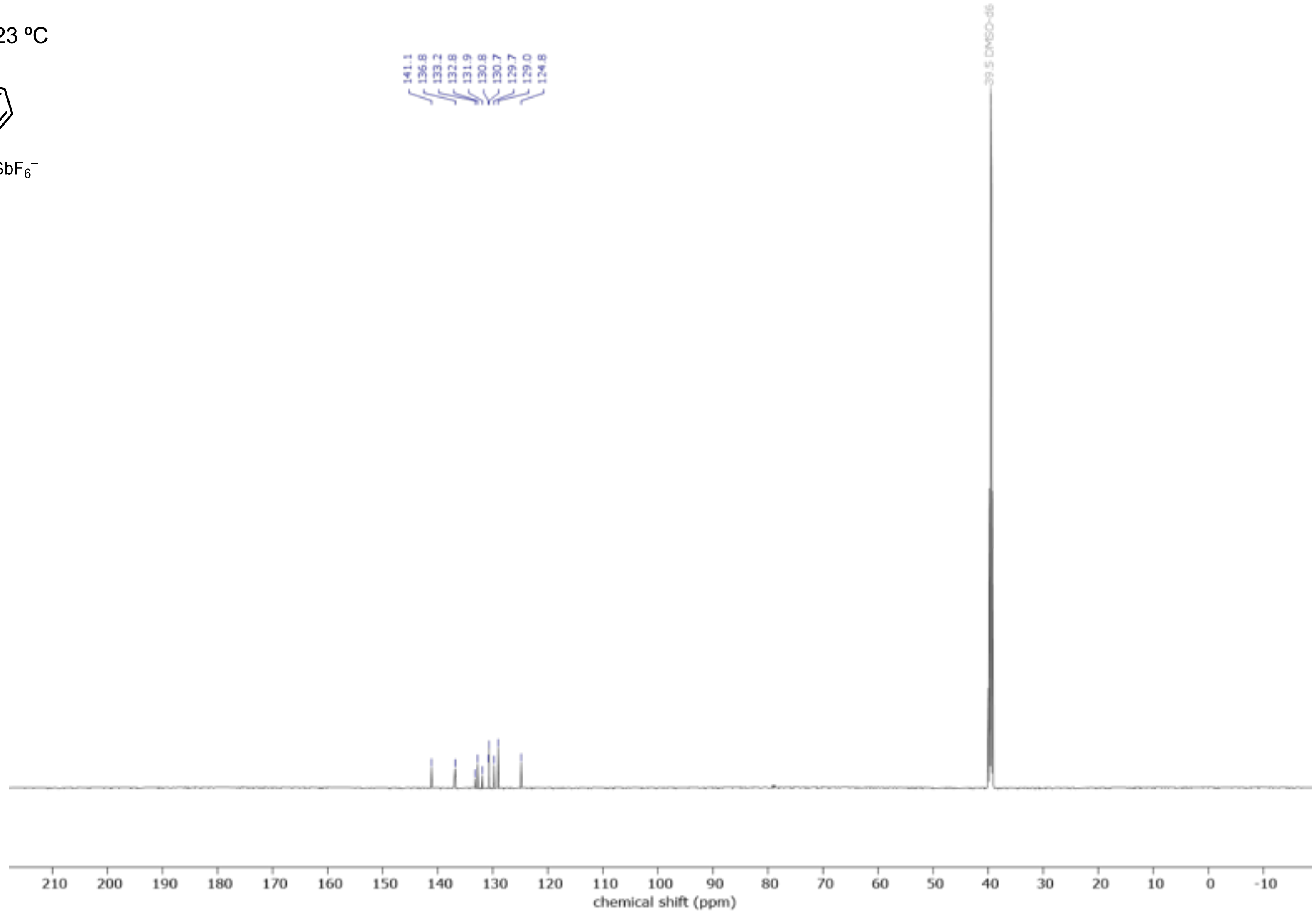

^{19}F NMR OF 5-(PHENYL) -DIBENZOSELENOPHENIUM HEXAFLUOROANTIMONATE (11)

DMSO-d6, 23 ºC

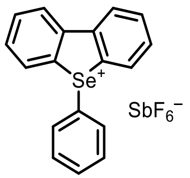

11

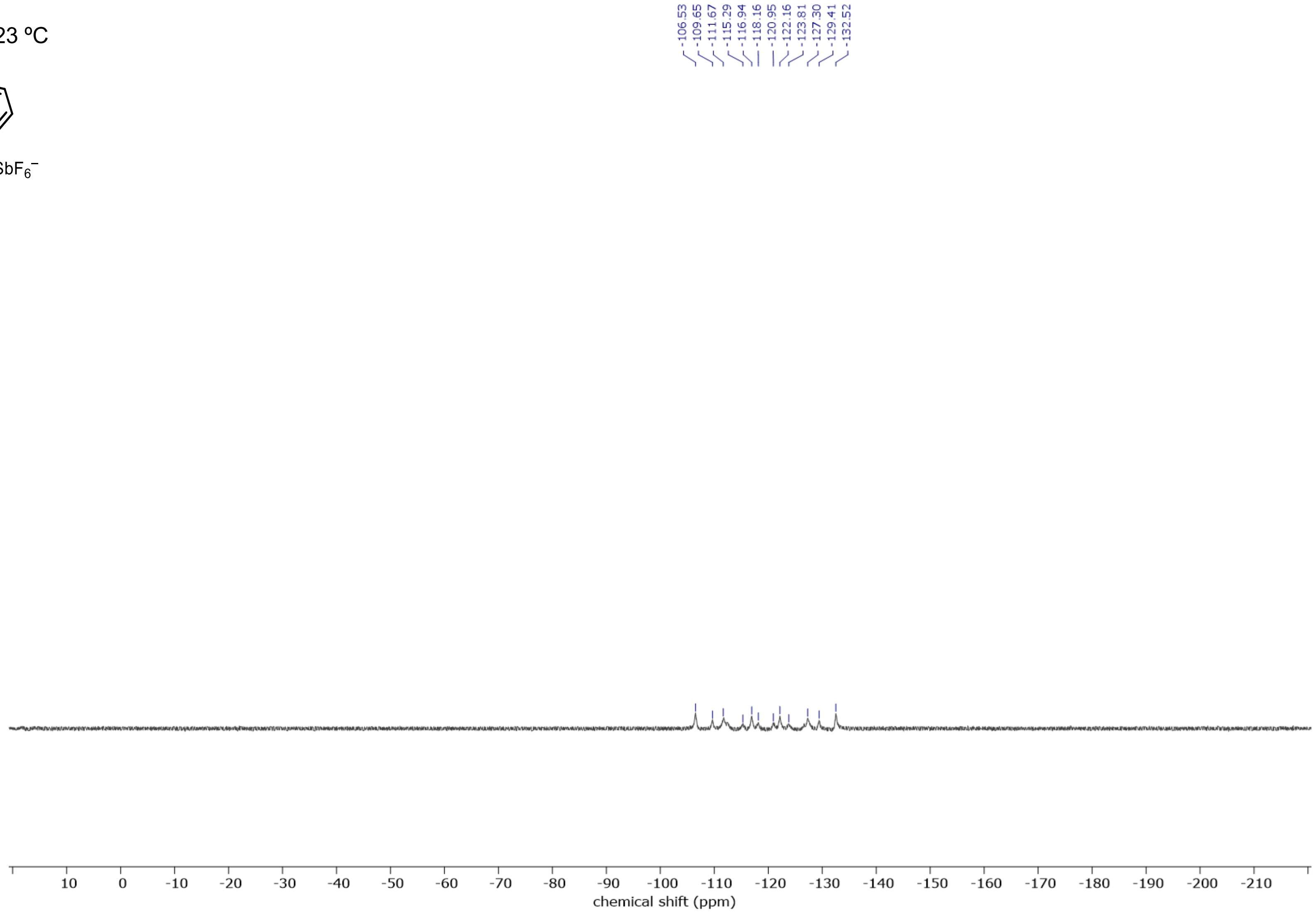

^{77}Se NMR of 5-(phenyl) -dibenzoselenophenium hexafluoroantimonate (11)

DMSO-d6, 23 ºC

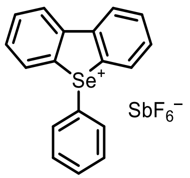

11

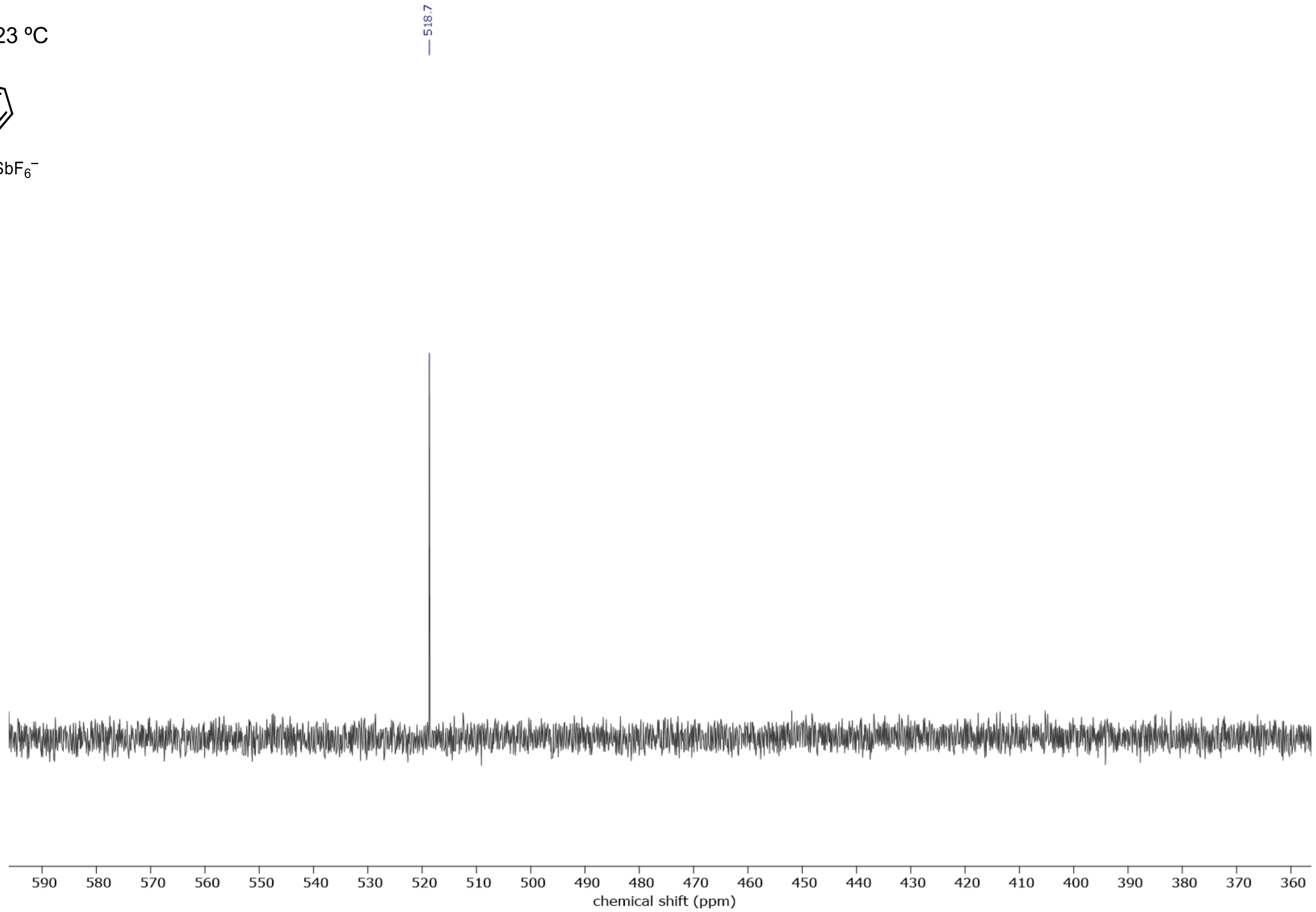

^{1}H NMR of 5-(phenyl) -dibenzoselenophenium hexafluoroantimonate (11b)

DMSO-d6, 23 ºC

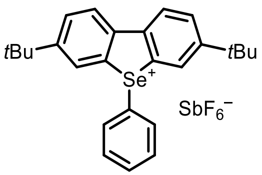

11b

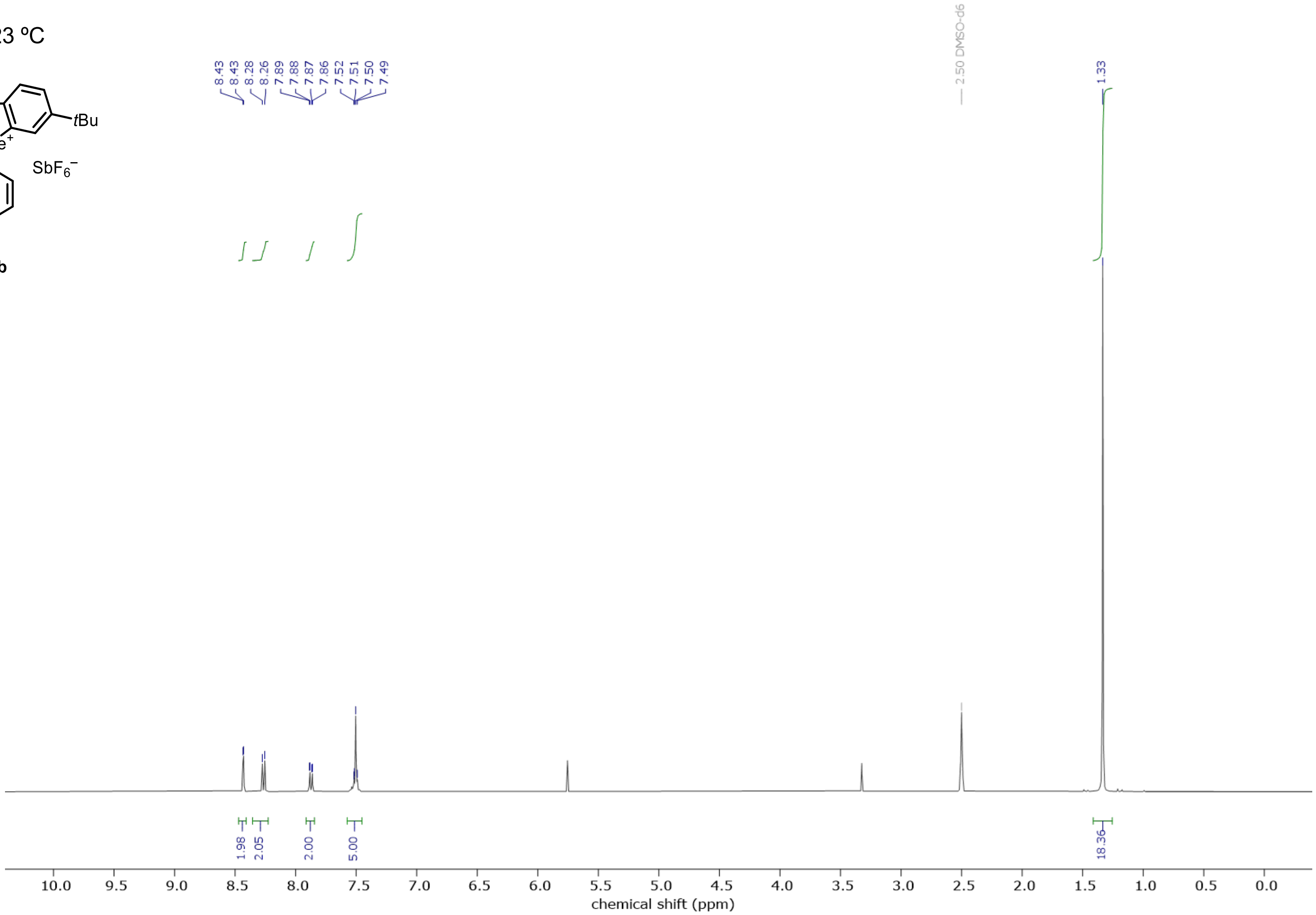

^{13}C NMR OF 5-(PHENYL) -DIBENZOSELENOPHENIUM HEXAFLUOROANTIMONATE (11B)

DMSO-d6, 23 ºC

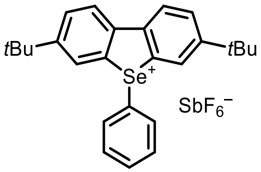

11b

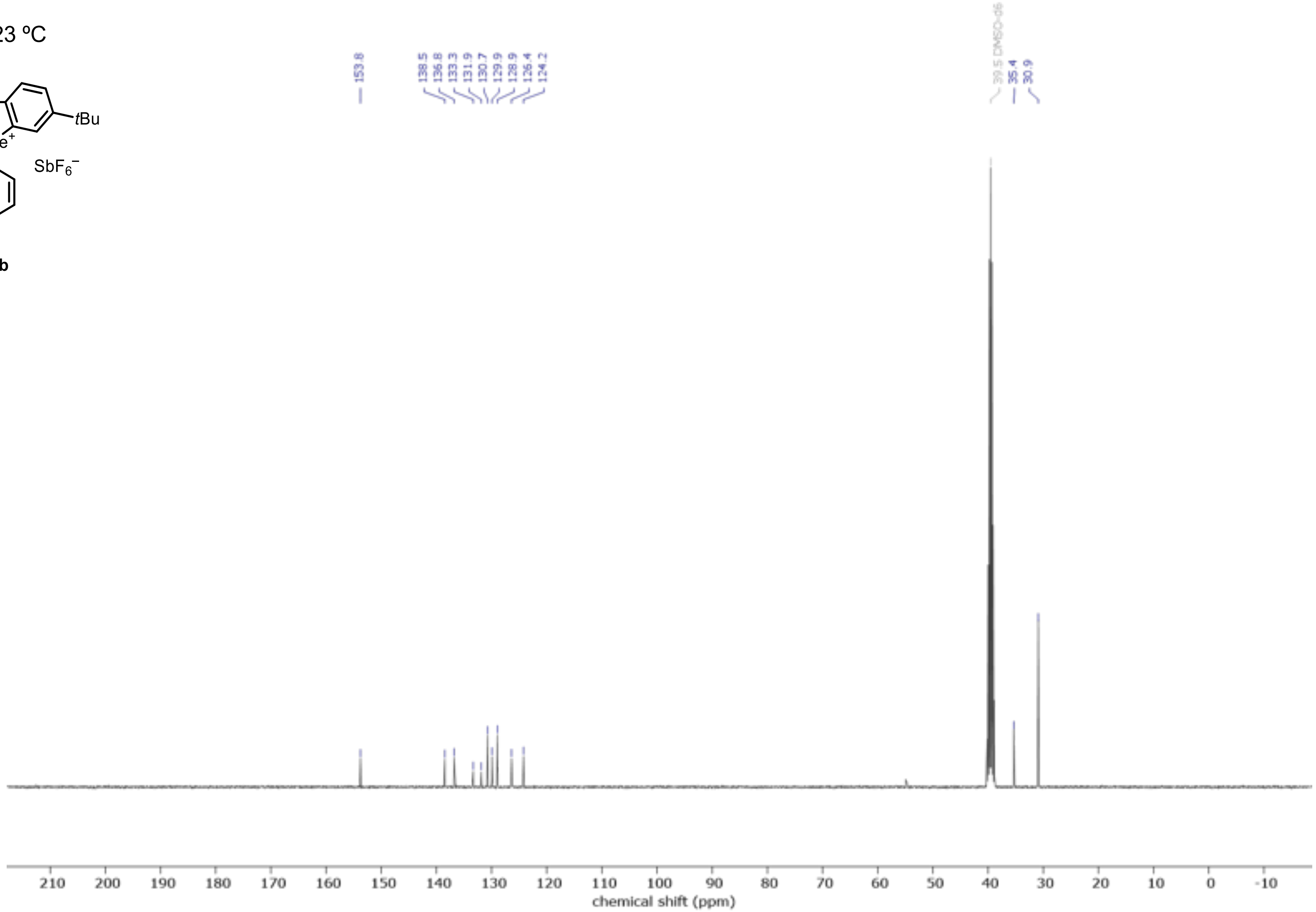

^{19}F NMR OF 5-(PHENYL) -DIBENZOSELENOPHENIUM HEXAFLUOROANTIMONATE (11B)

DMSO-d6, 23 ºC

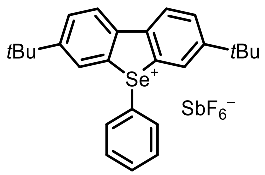

11b

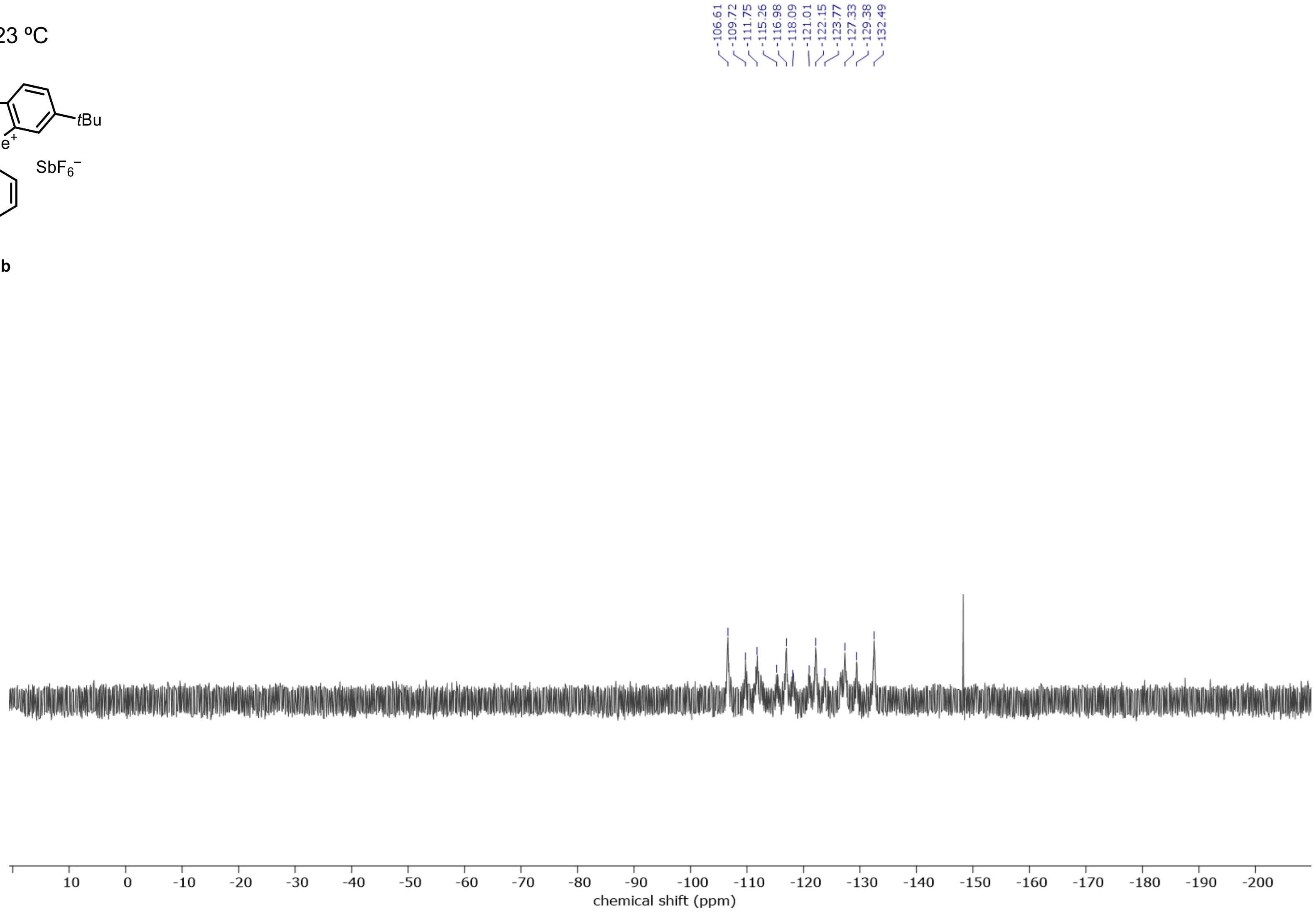

^{77}Se NMR of 5-(phenyl) -dibenzoselenophenium hexafluoroantimonate (11b)

DMSO-d6, 23 ºC

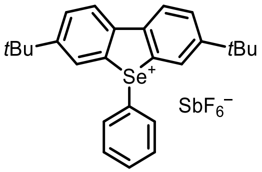

11b

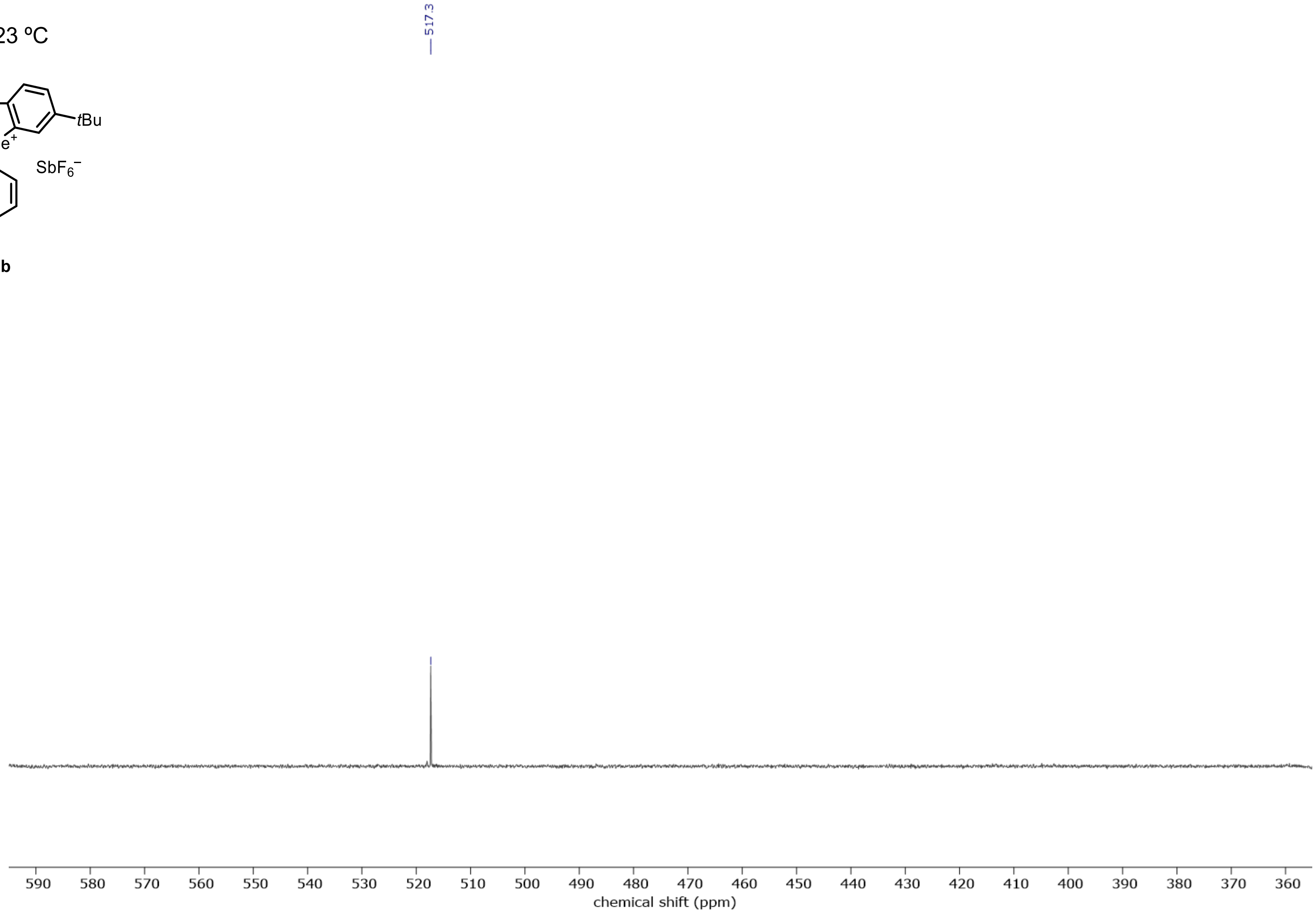

^{1}H NMR OF 5-(PHENYL) -DIBENZOSELENOPHENIUM HEXAFLUOROANTIMONATE (11C)

CDCl3, 23 ºC

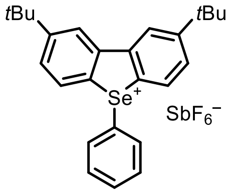

11c

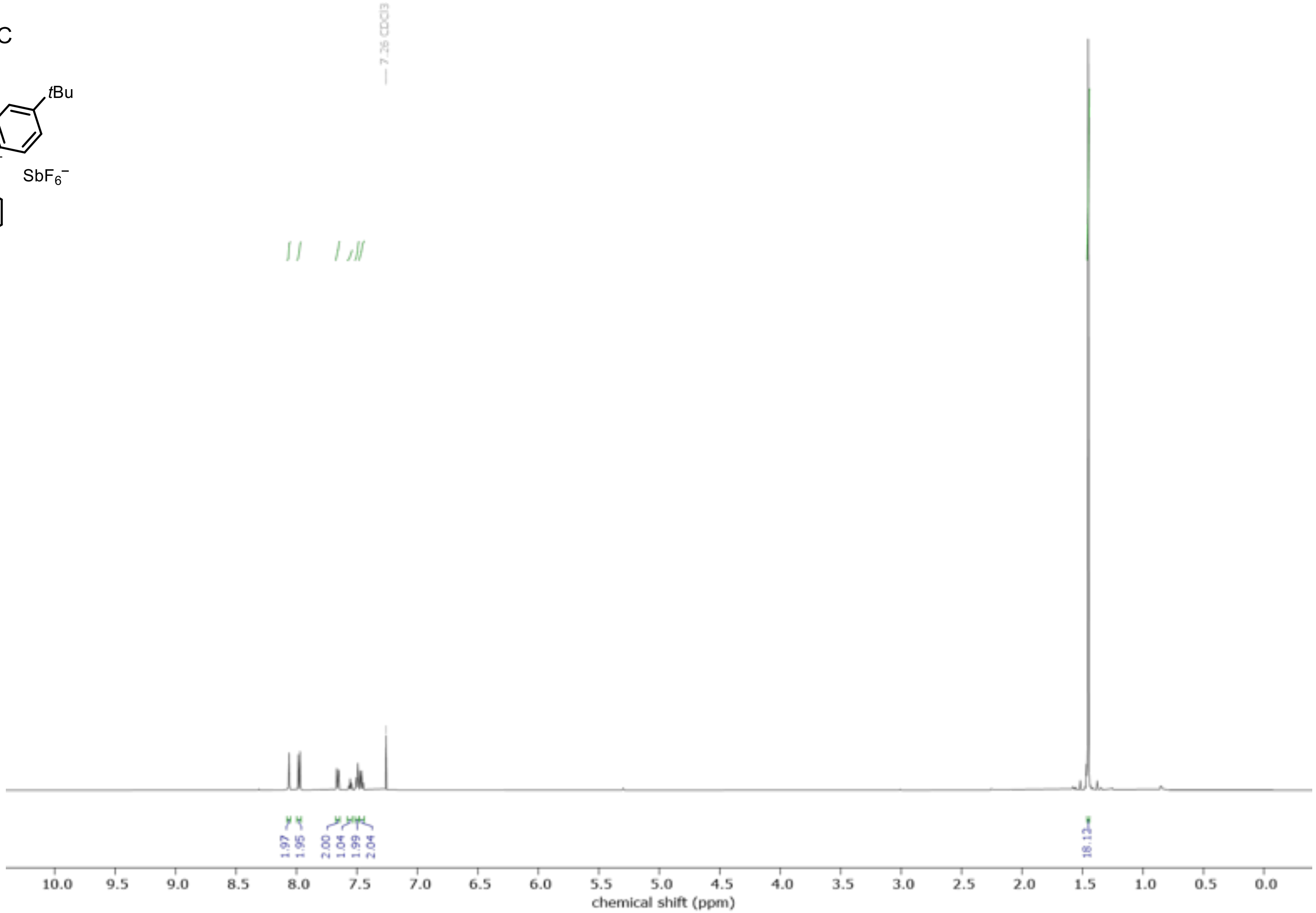

^{13}C NMR OF 5-(PHENYL) -DIBENZOSELENOPHENIUM HEXAFLUOROANTIMONATE (11C)

CDCl$_3$, 23 ºC

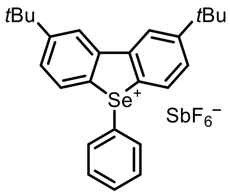

11c

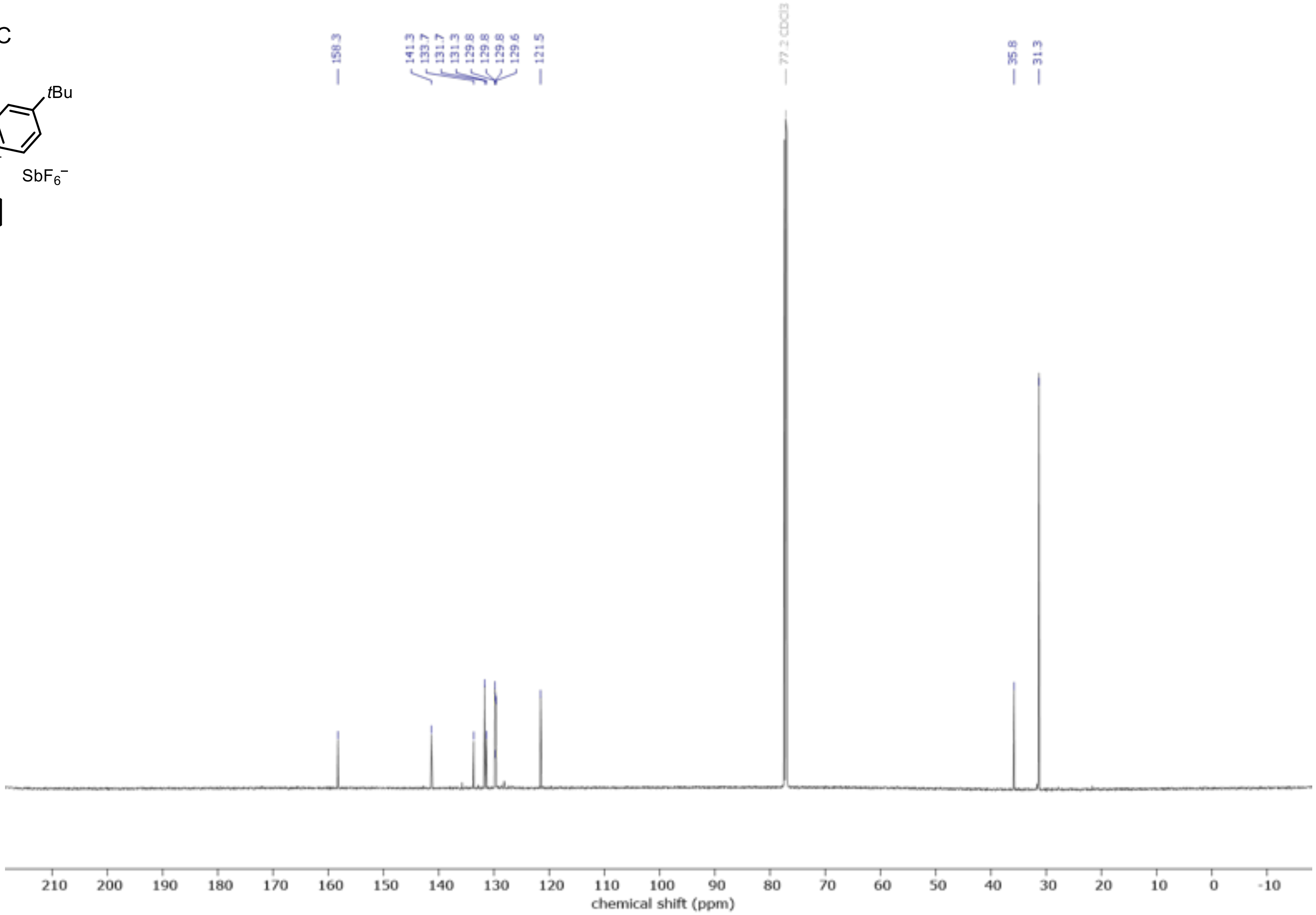

^{19}F NMR of 5-(phenyl)-dibenzoselenophenium hexafluoroantimonate (11c)

CDCl$_3$, 23 ºC

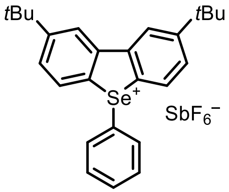

11c

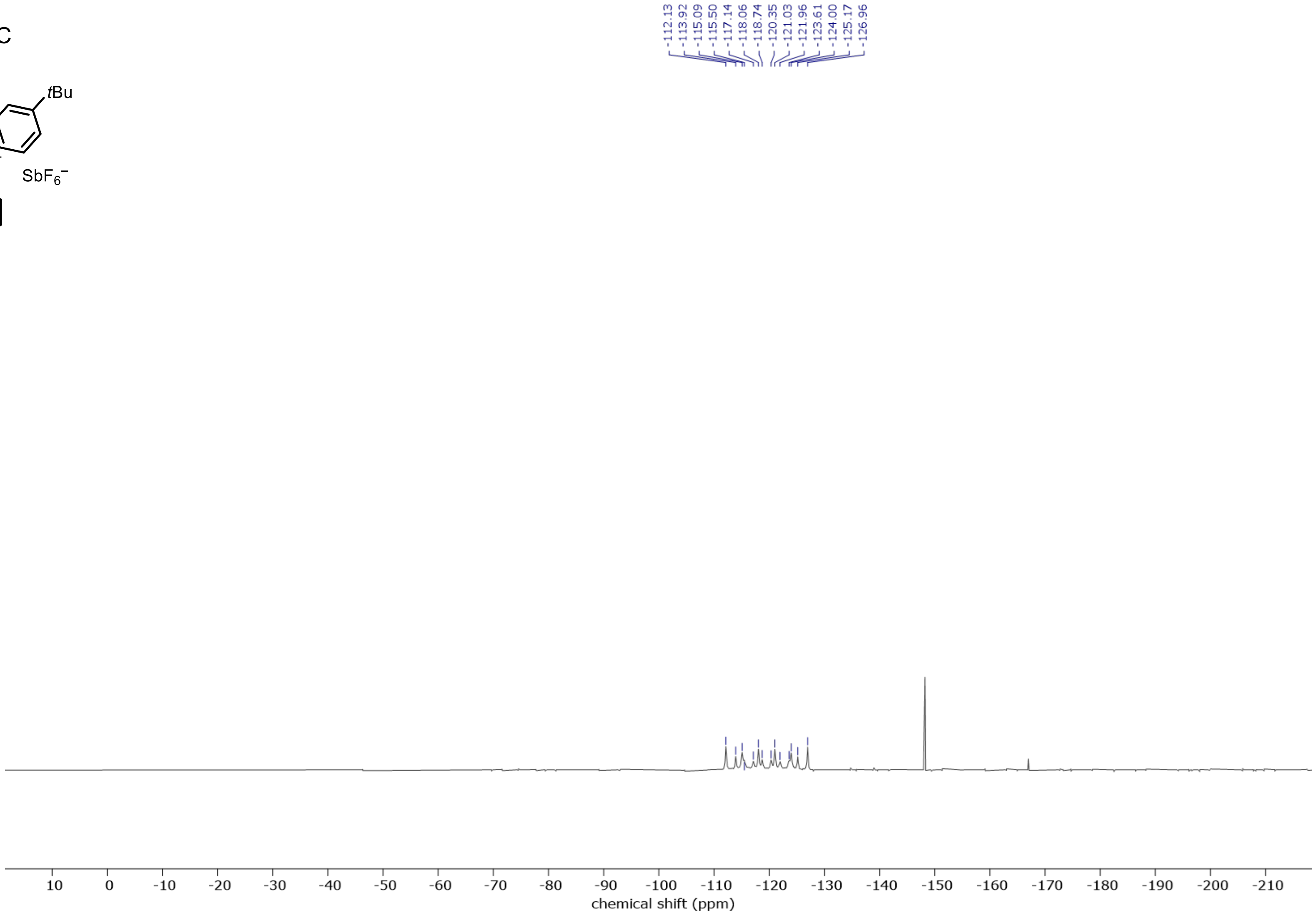

^{77}Se NMR of 5-(phenyl) -dibenzoselenophenium hexafluoroantimonate (11c)

CDCl$_3$, 23 ºC

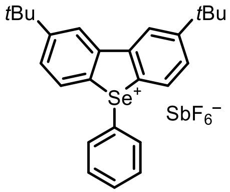

11c

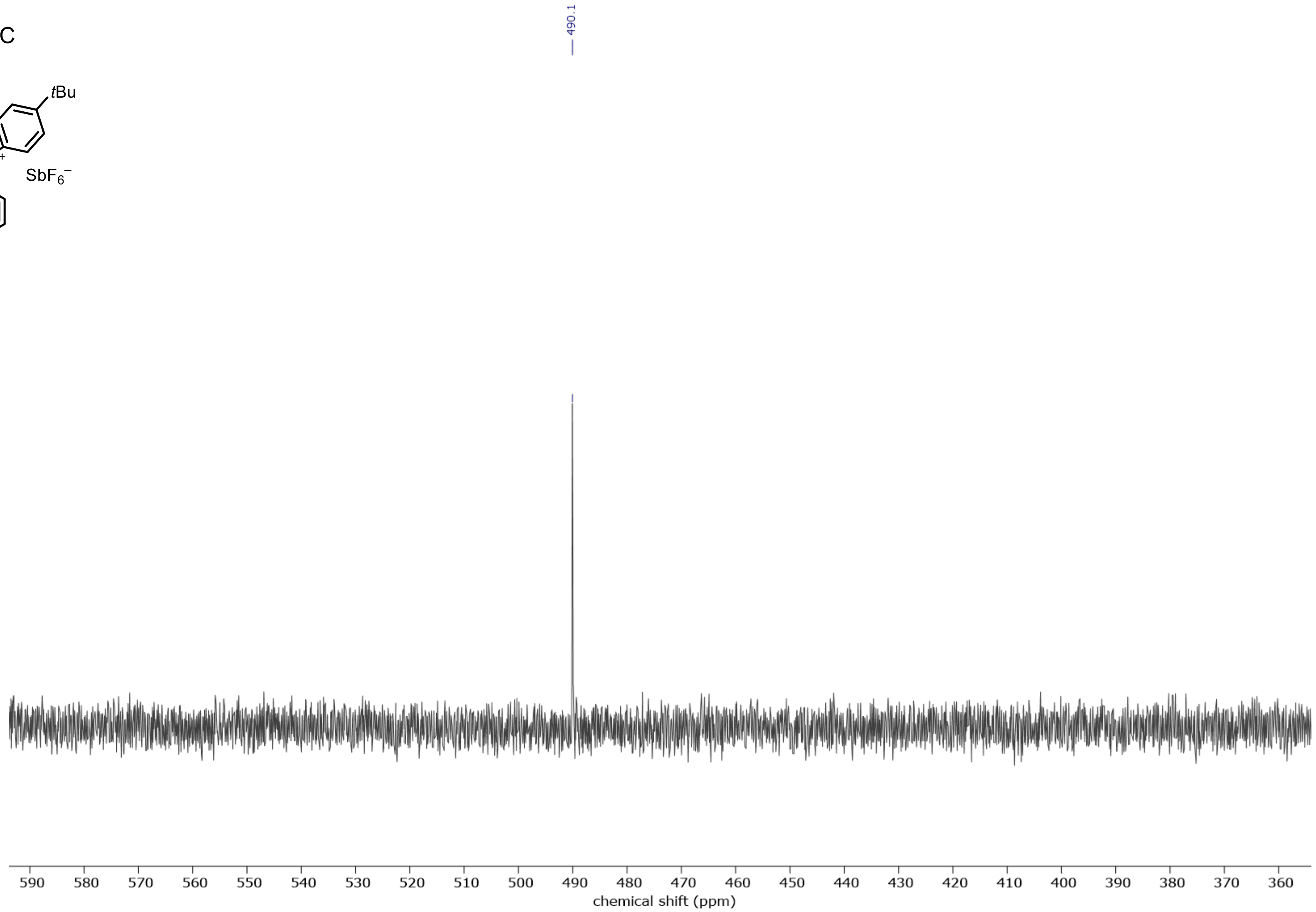

^{1}H NMR of 5-(phenyl) -dibenzoselenophenium hexafluoroantimonate (11d)

CDCl3, 23 ºC

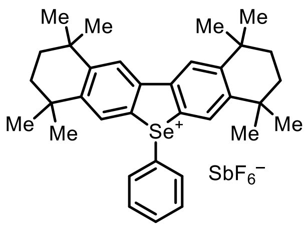

11d

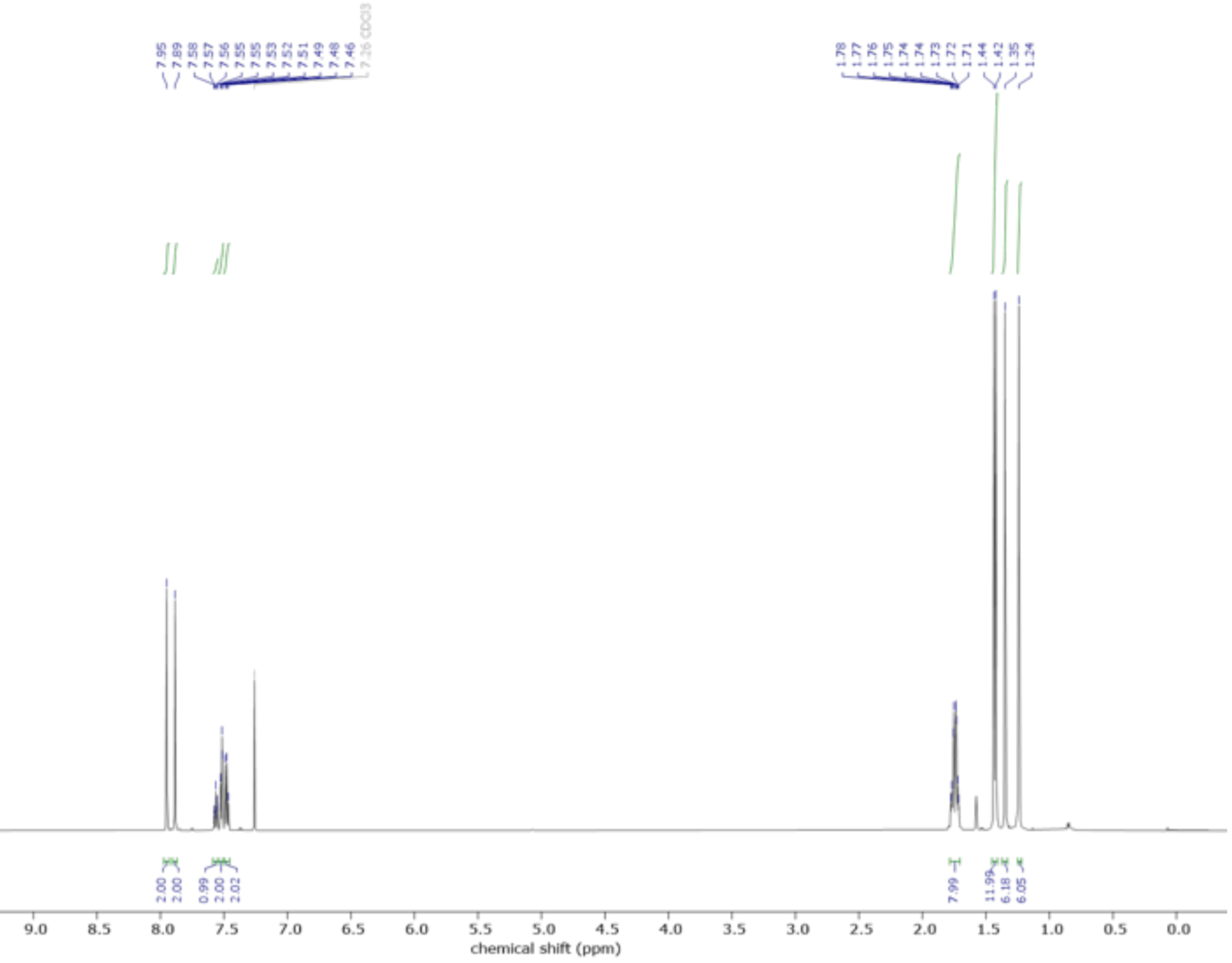

^{13}C NMR of 5-(phenyl) -dibenzoselenophenium hexafluoroantimonate (11d)

CDCl₃, 23 °C

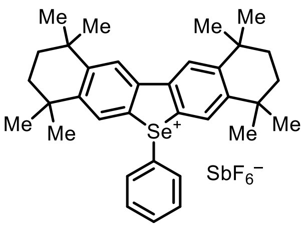

11d

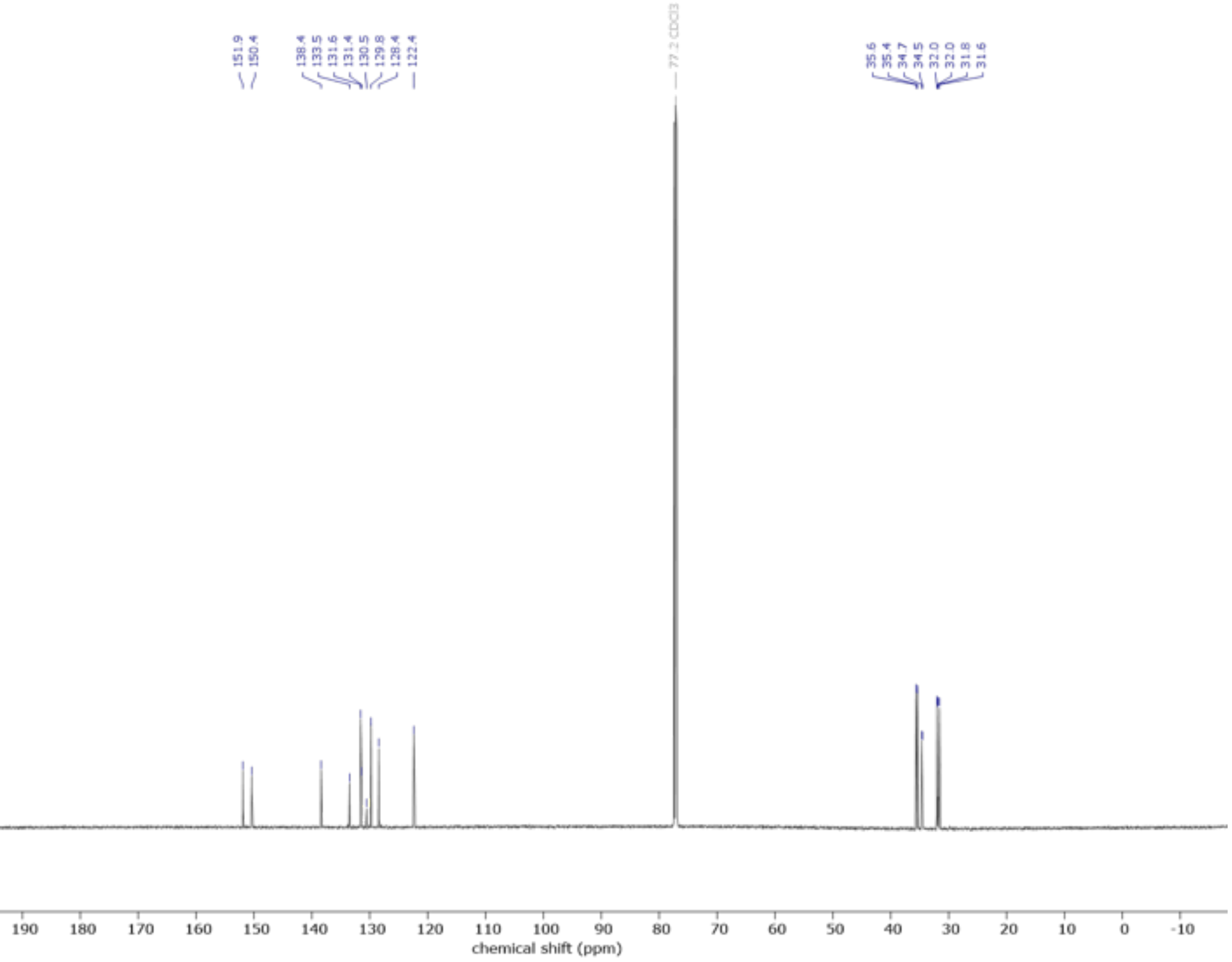

^{19}F NMR OF 5-(PHENYL) -DIBENZOSELENOPHENIUM HEXAFLUOROANTIMONATE (11D)

CD3CN, 23 ºC

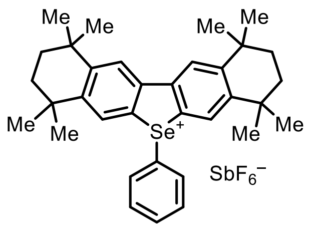

11d

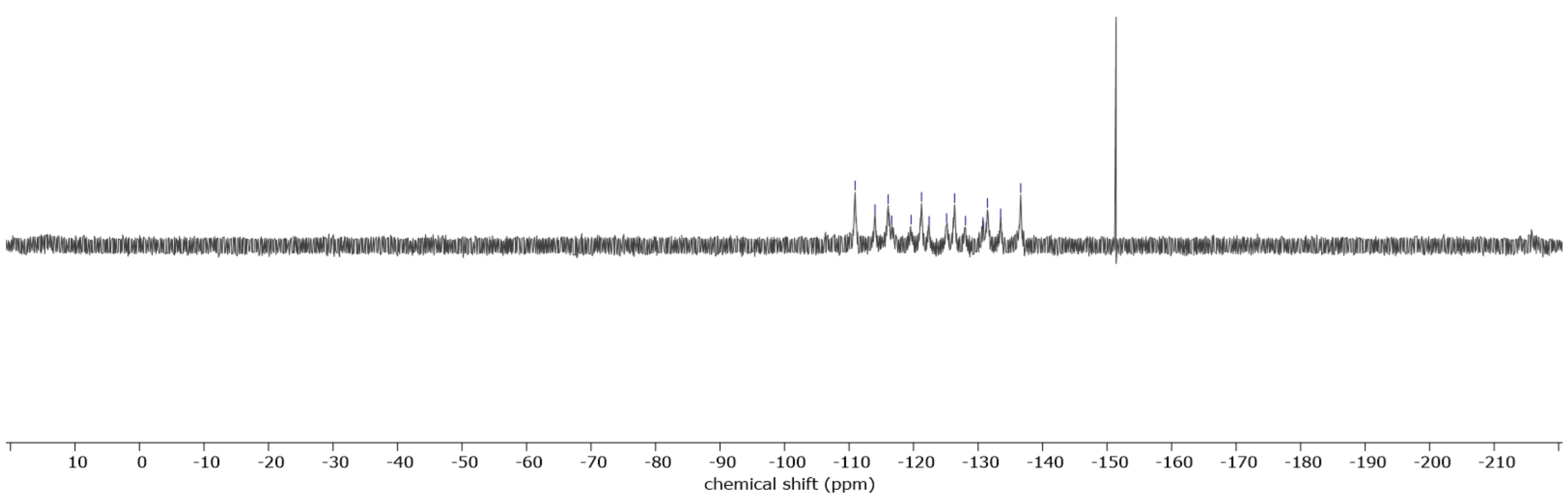

^{77}Se NMR of 5-(phenyl) -dibenzoselenophenium hexafluoroantimonate (11d)

CDCl₃, 23 ºC

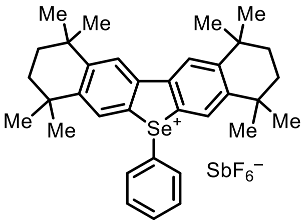

11d

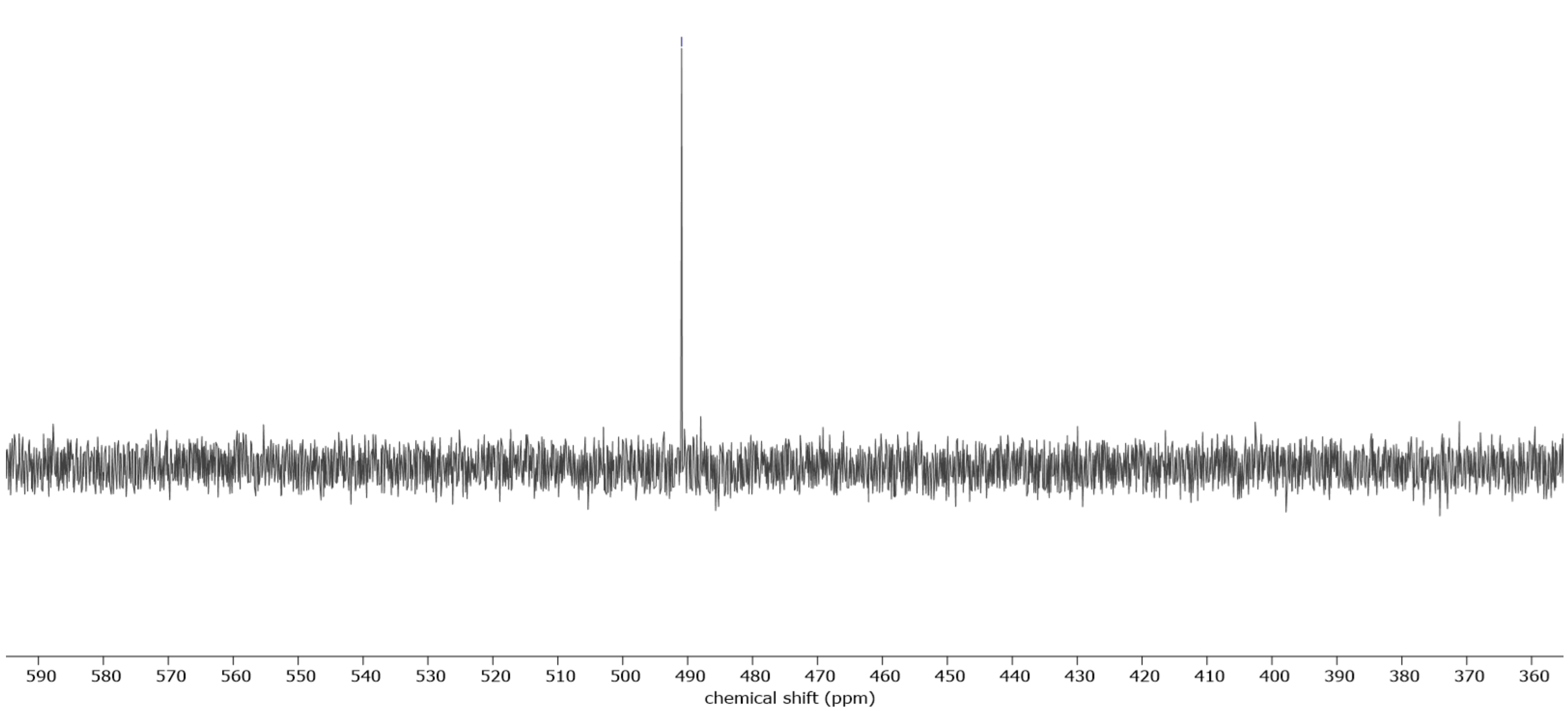

^{1}H NMR of BF_3K fenofibrate derivative (12)

DMSO-d6, 23 ºC

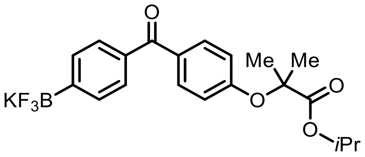

12

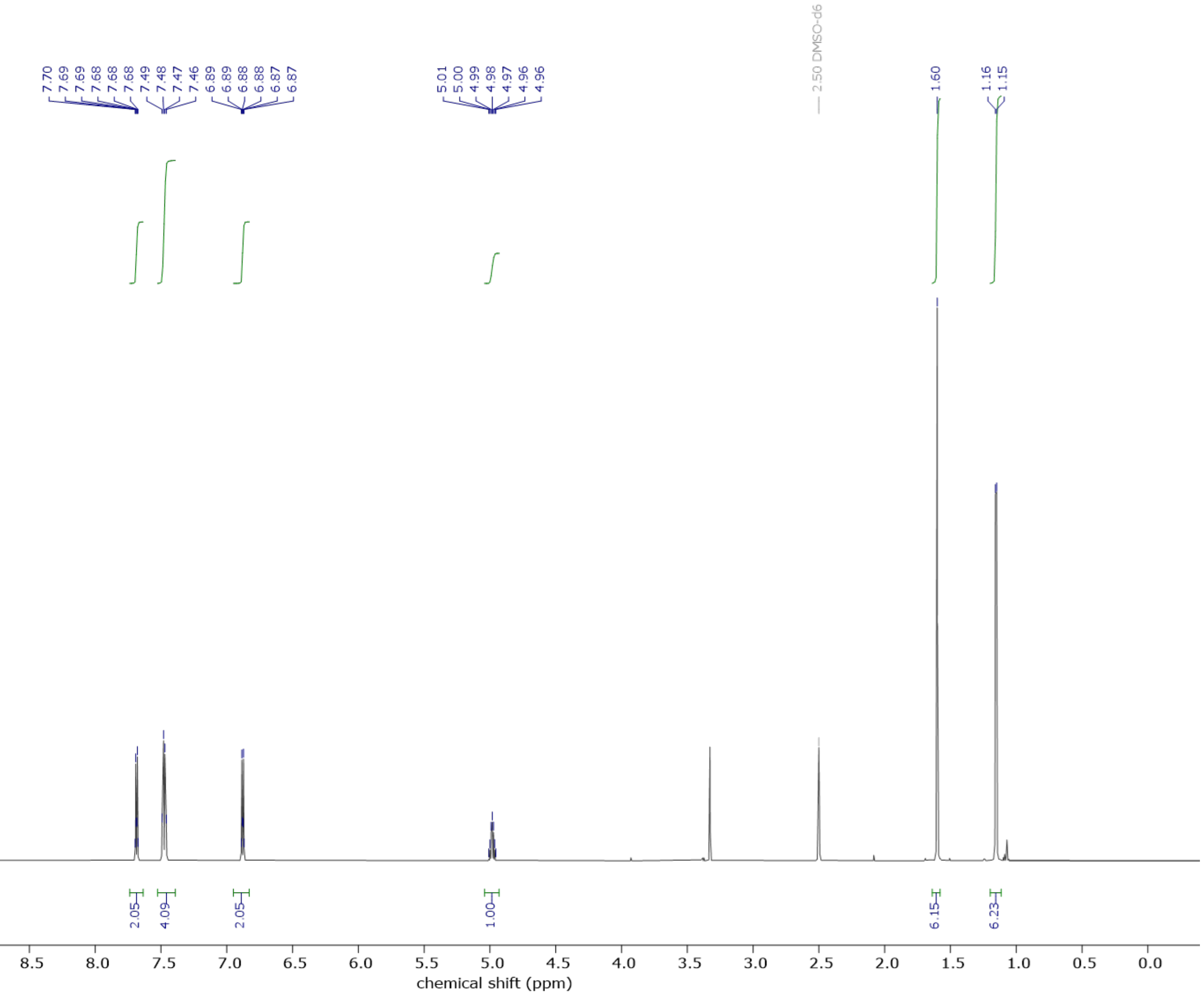

^{13}C NMR of BF_3K fenofibrate derivative (12)

DMSO-d6, 23 ºC

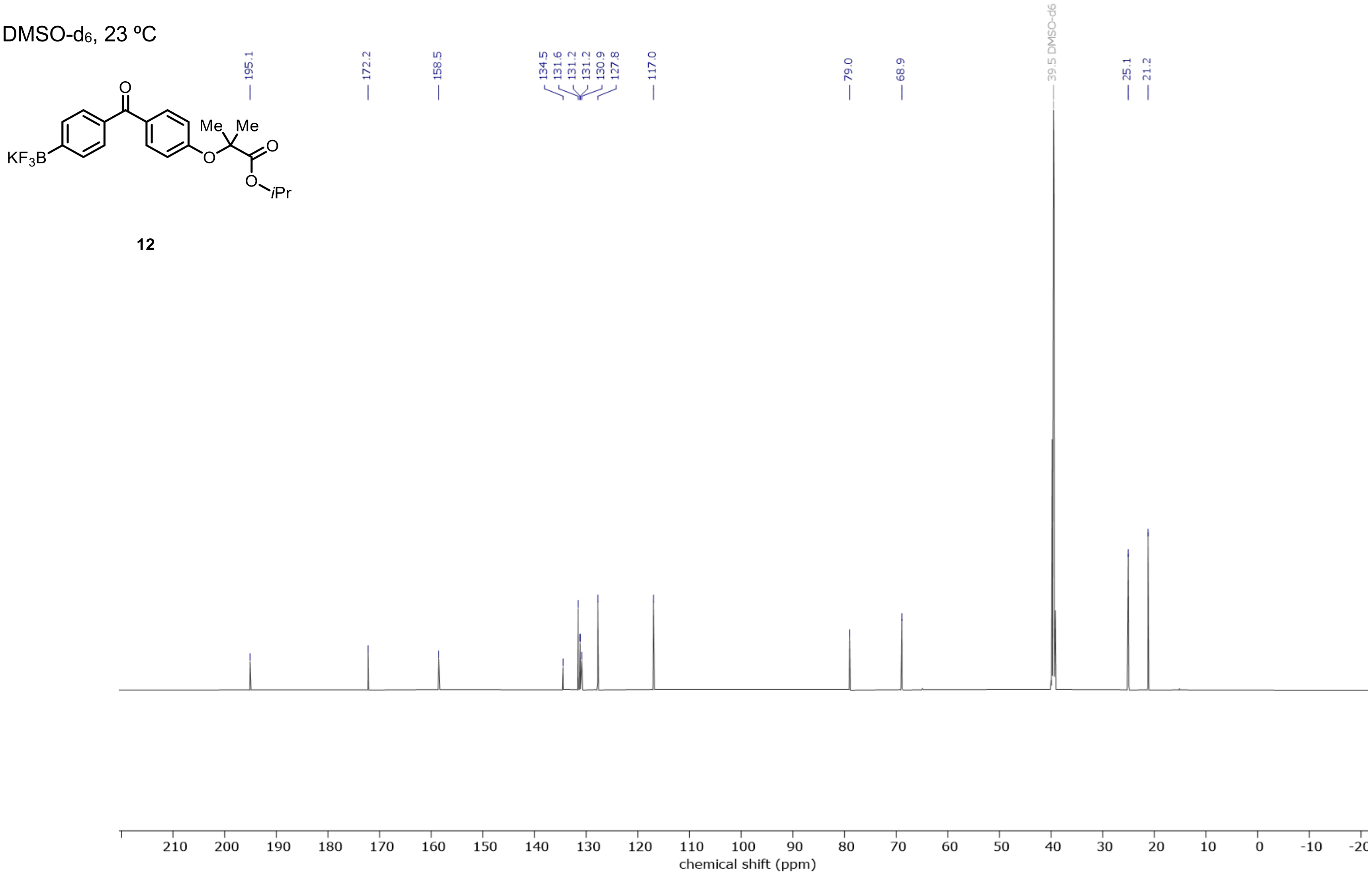

^{19}F NMR of BF_3K fenofibrate derivative (12)

DMSO-d6, 23 ºC

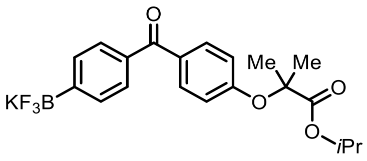

12

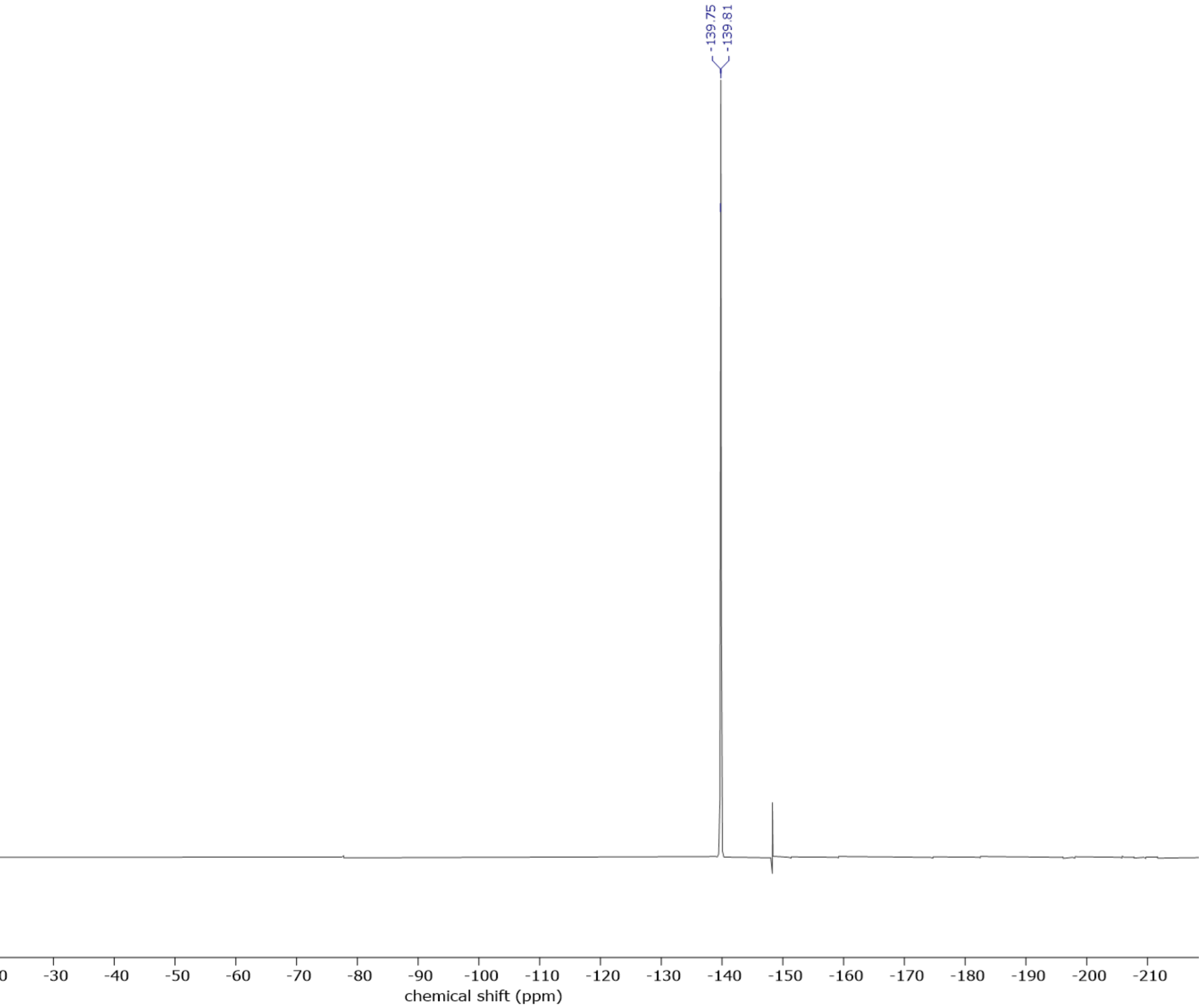

^{11}B NMR of BF_3K fenofibrate derivative (12)

DMSO-d6, 23 ºC

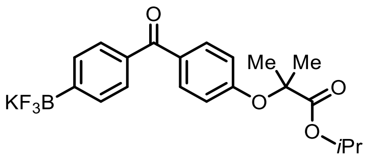

12

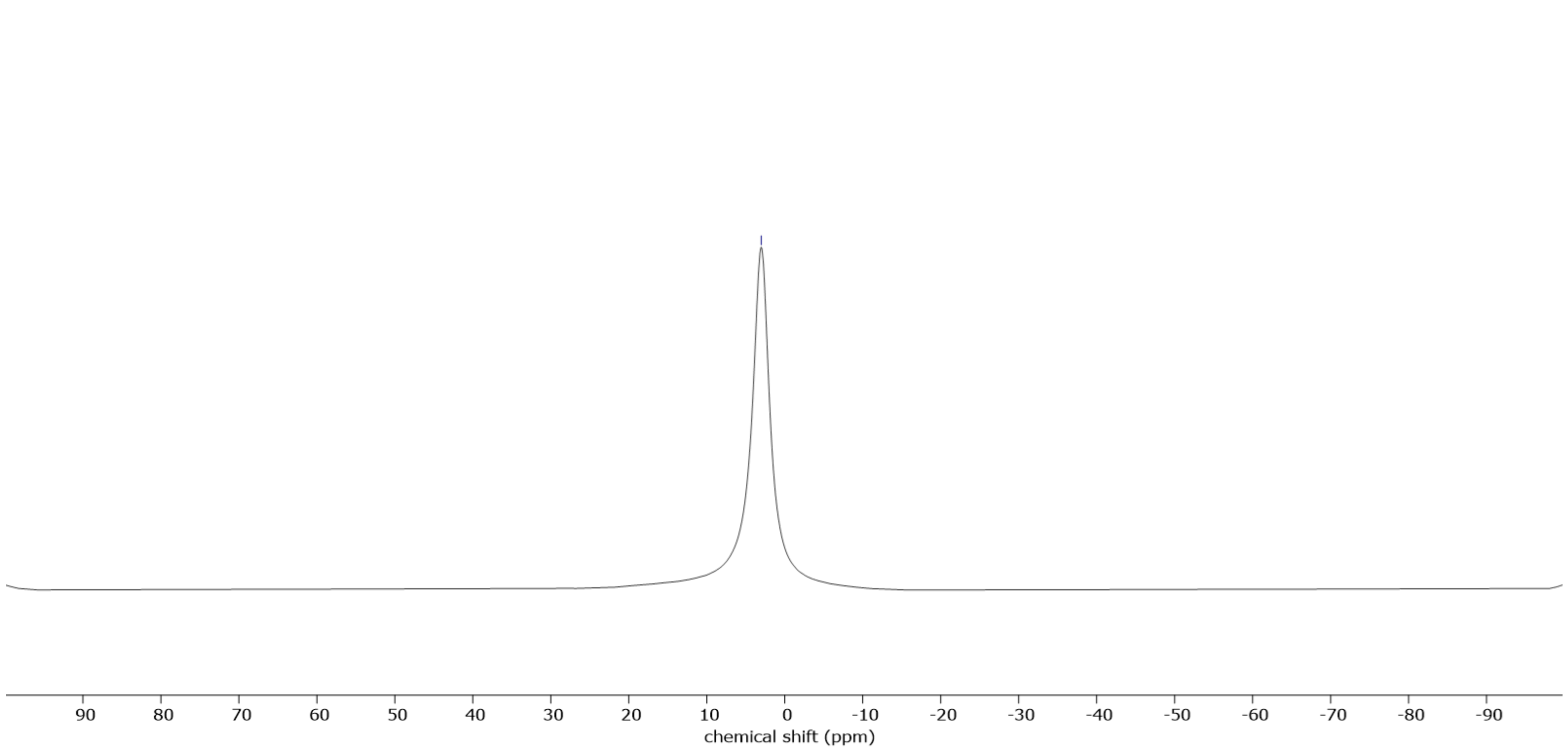

^{1}H NMR OF SELENONIUM FENOFIBRATE DERIVATIVE (13)

DMSO-d6, 23 ºC

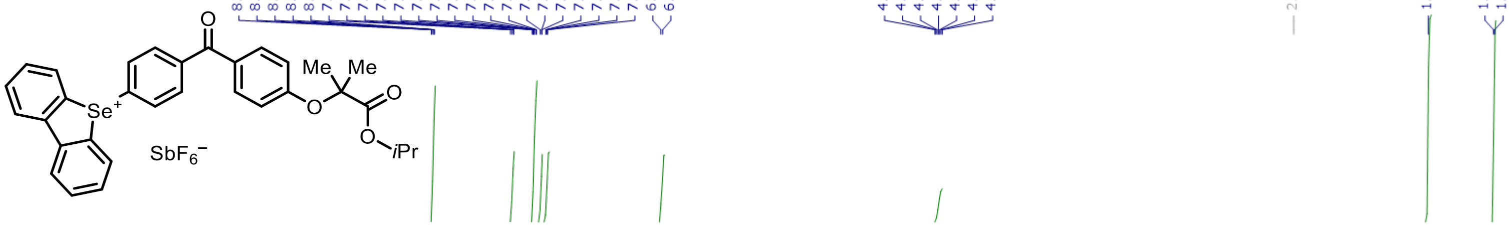

13

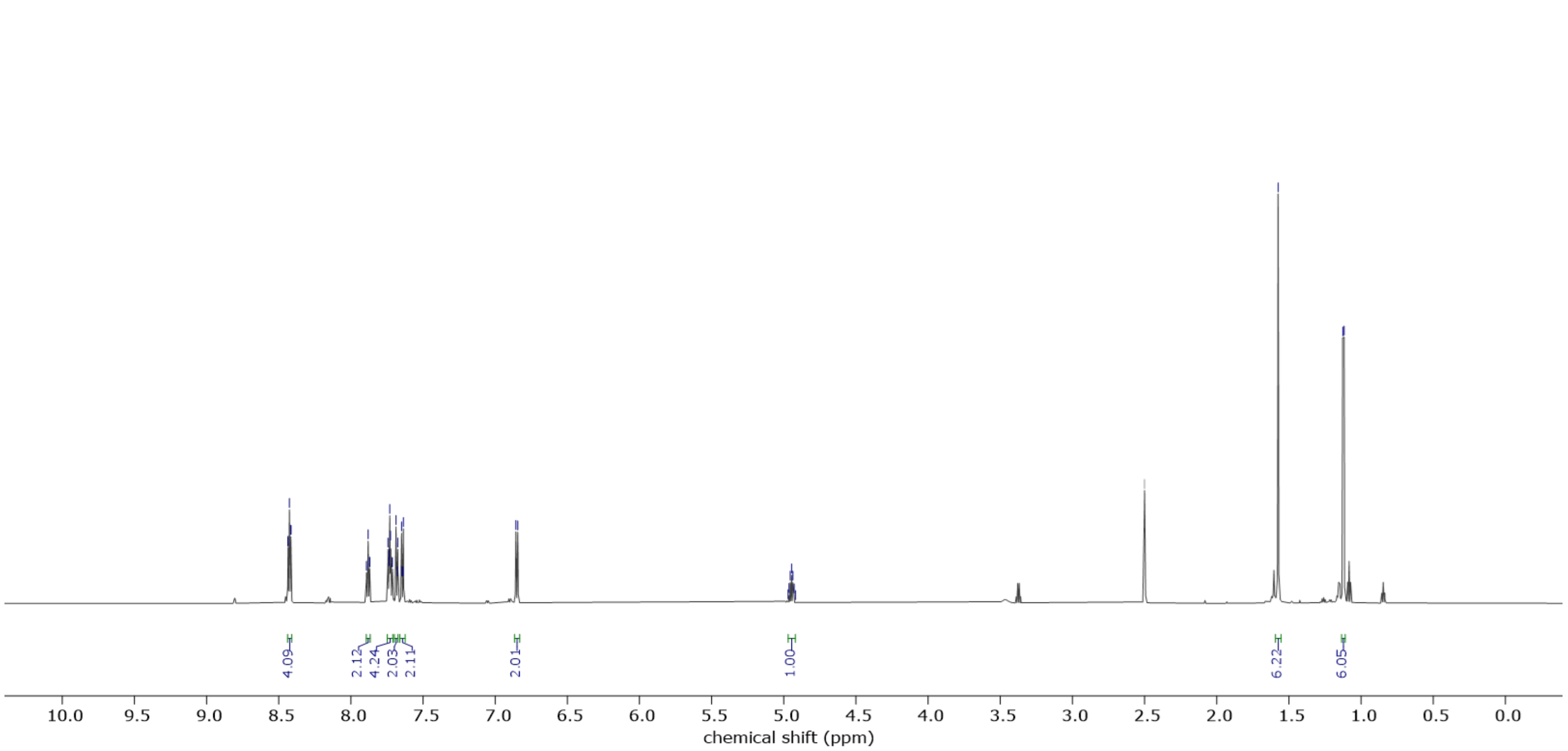

^{13}C NMR OF SELENONIUM FENOFIBRATE DERIVATIVE (13)

DMSO-d6, 23 ºC

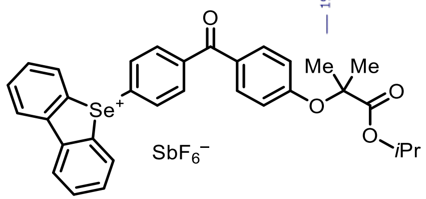

13

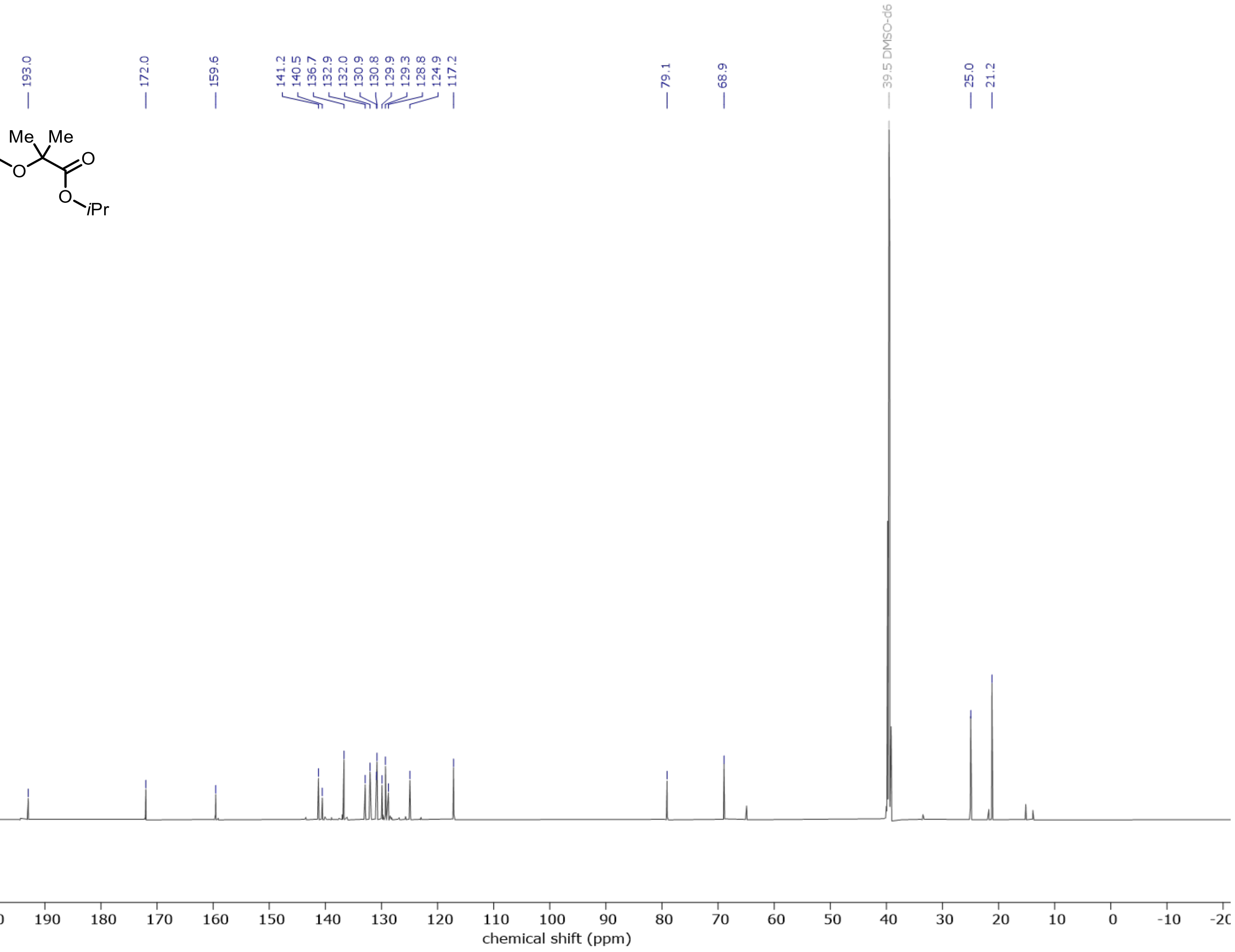

^{19}F NMR OF SELENONIUM FENOFIBRATE DERIVATIVE (13)

DMSO-d6, 23 ºC

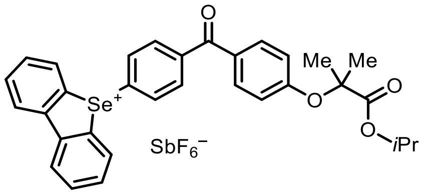

13

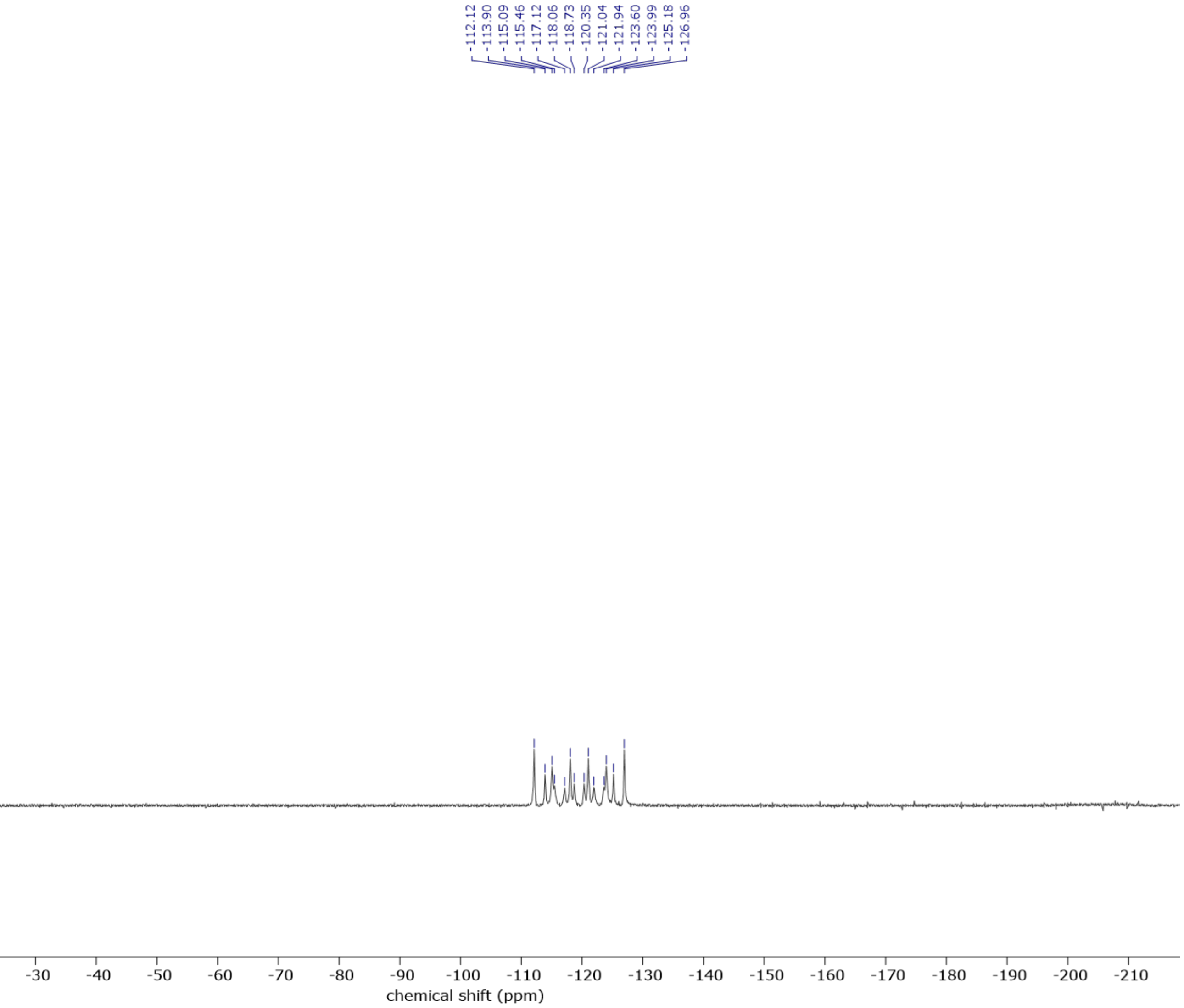

^{77}Se NMR of selenonium fenofibrate derivative (13)

DMSO-d6, 23 ºC

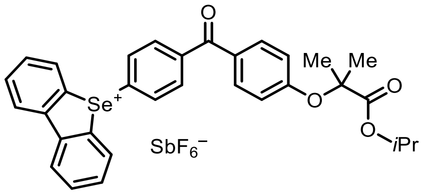

13

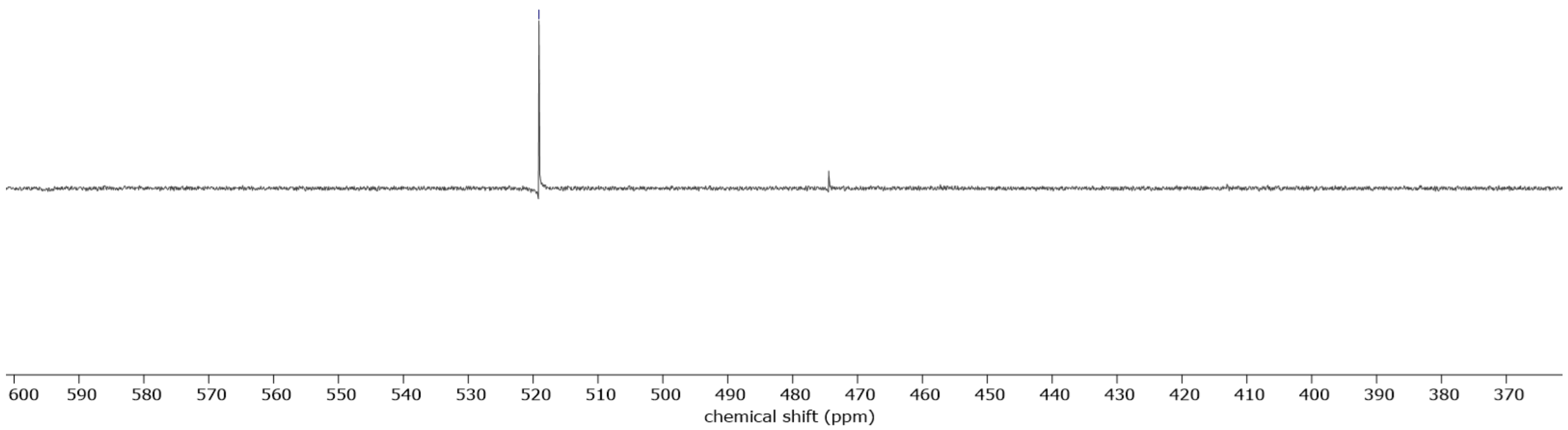

^{1}H NMR of BF_3K tianeptine intermediate (14)

DMSO-d6, 23 ºC

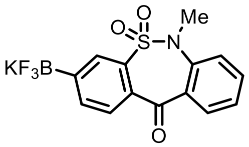

14

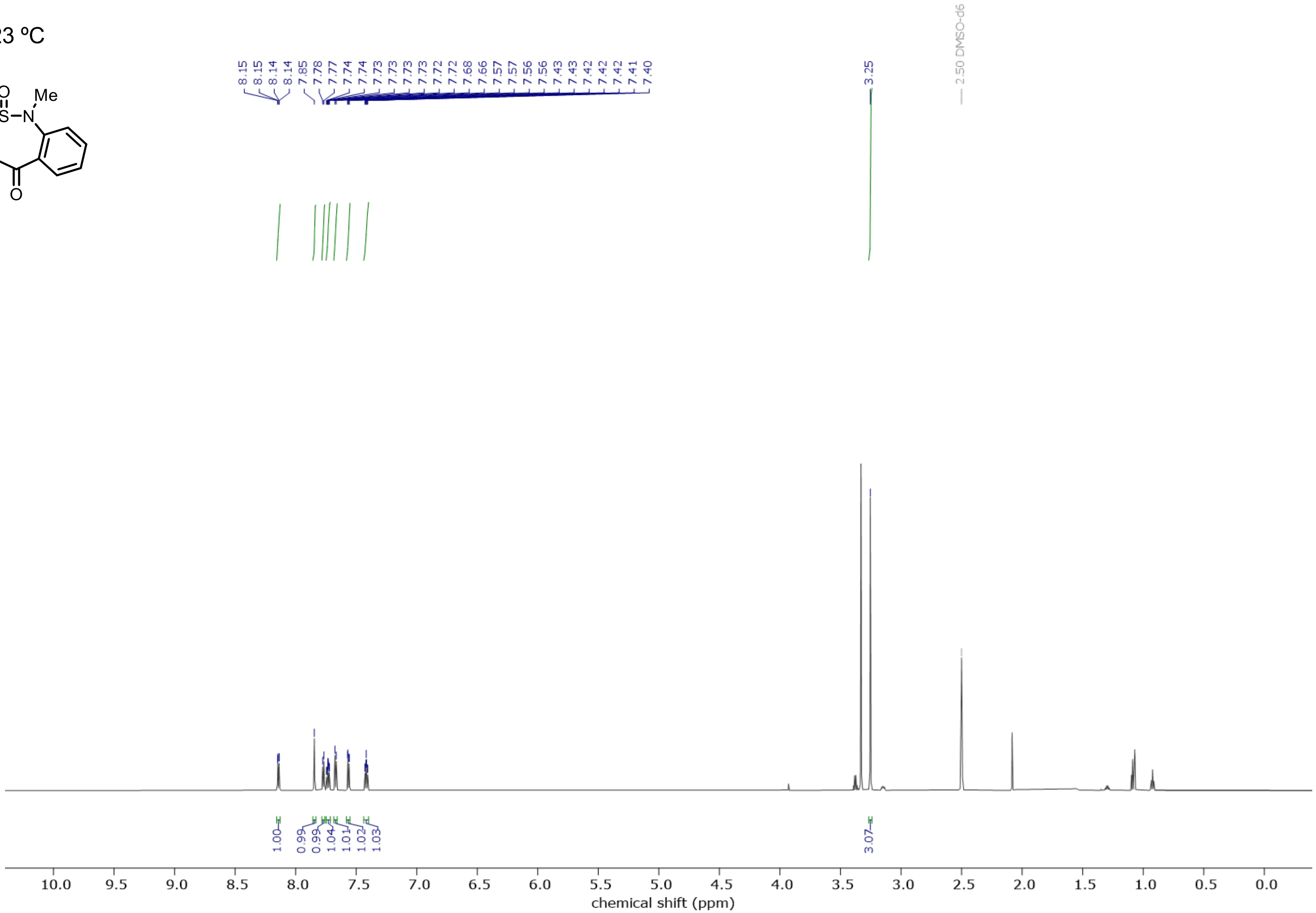

^{13}C NMR OF 5-(PHENYL) -DIBENZOSELENOPHENIUM HEXAFLUOROANTIMONATE (14)

DMSO-d6, 23 ºC

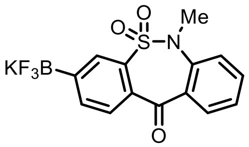

14

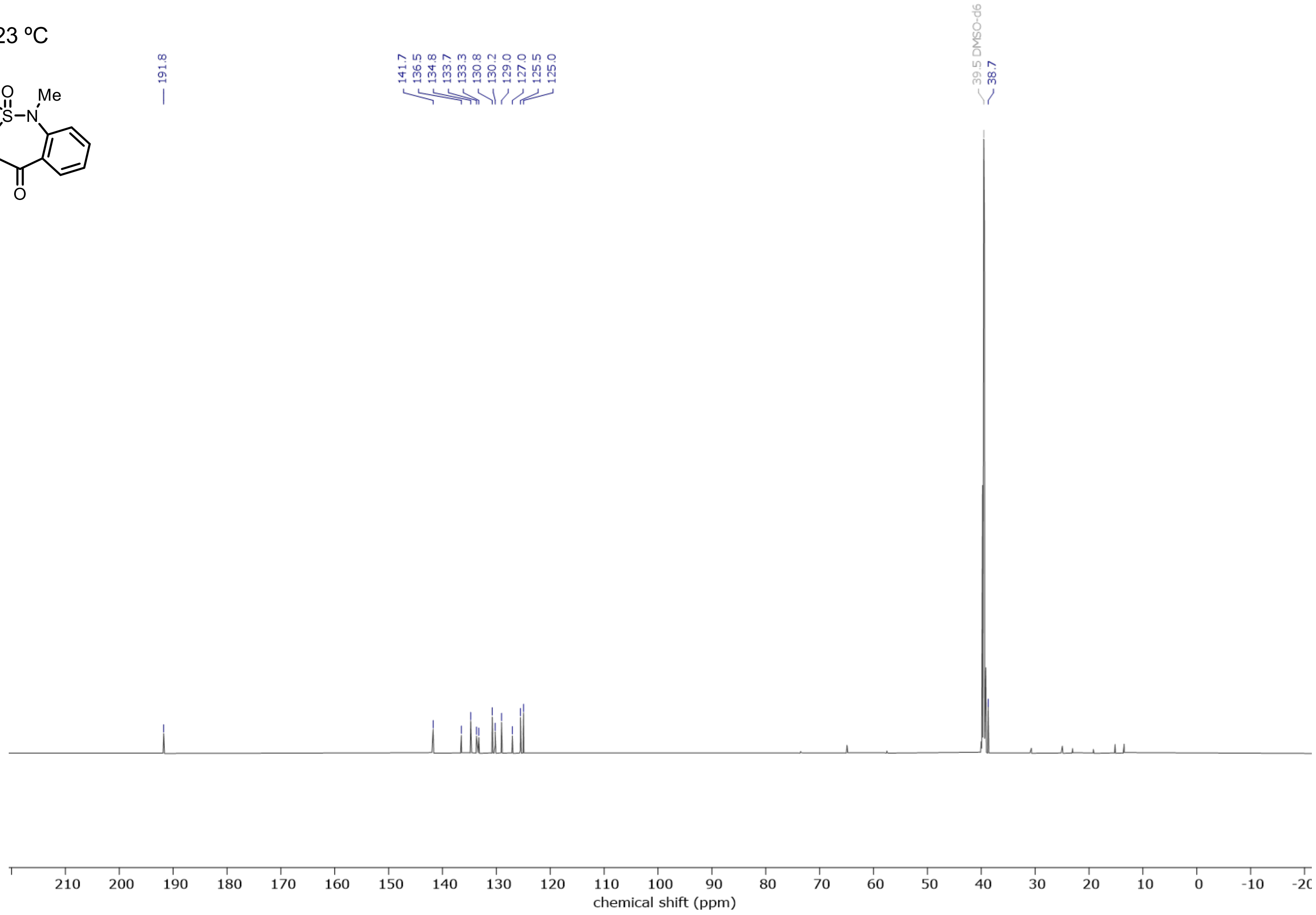

^{19}F NMR of BF_3K tianeptine intermediate (14)

DMSO-d6, 23 ºC

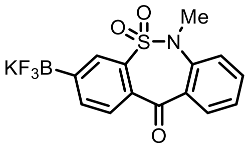

14

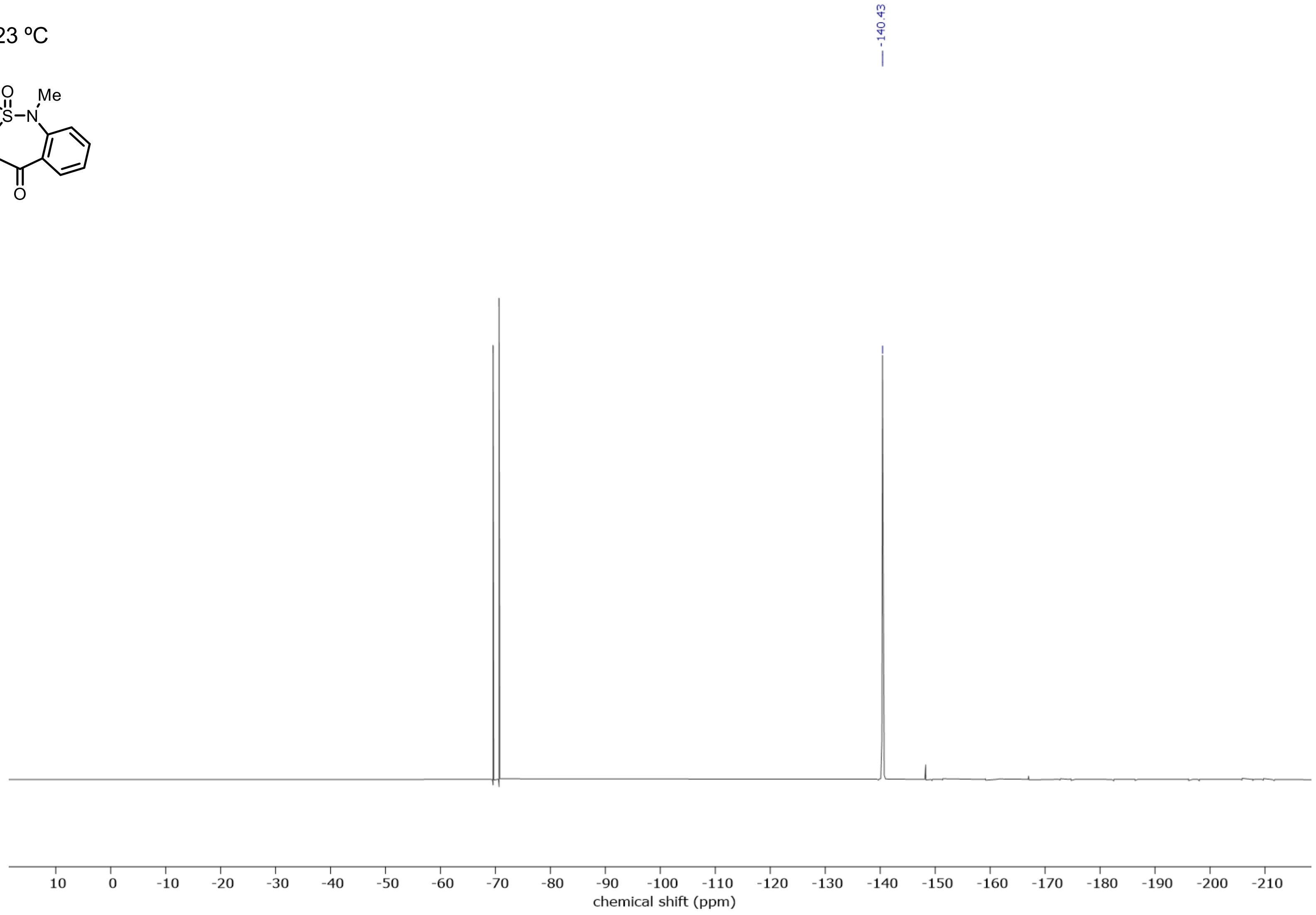

^{11}B NMR of BF_3K tianeptine intermediate (14)

DMSO-d6, 23 ºC

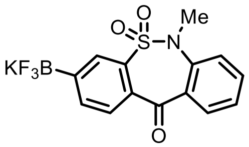

14

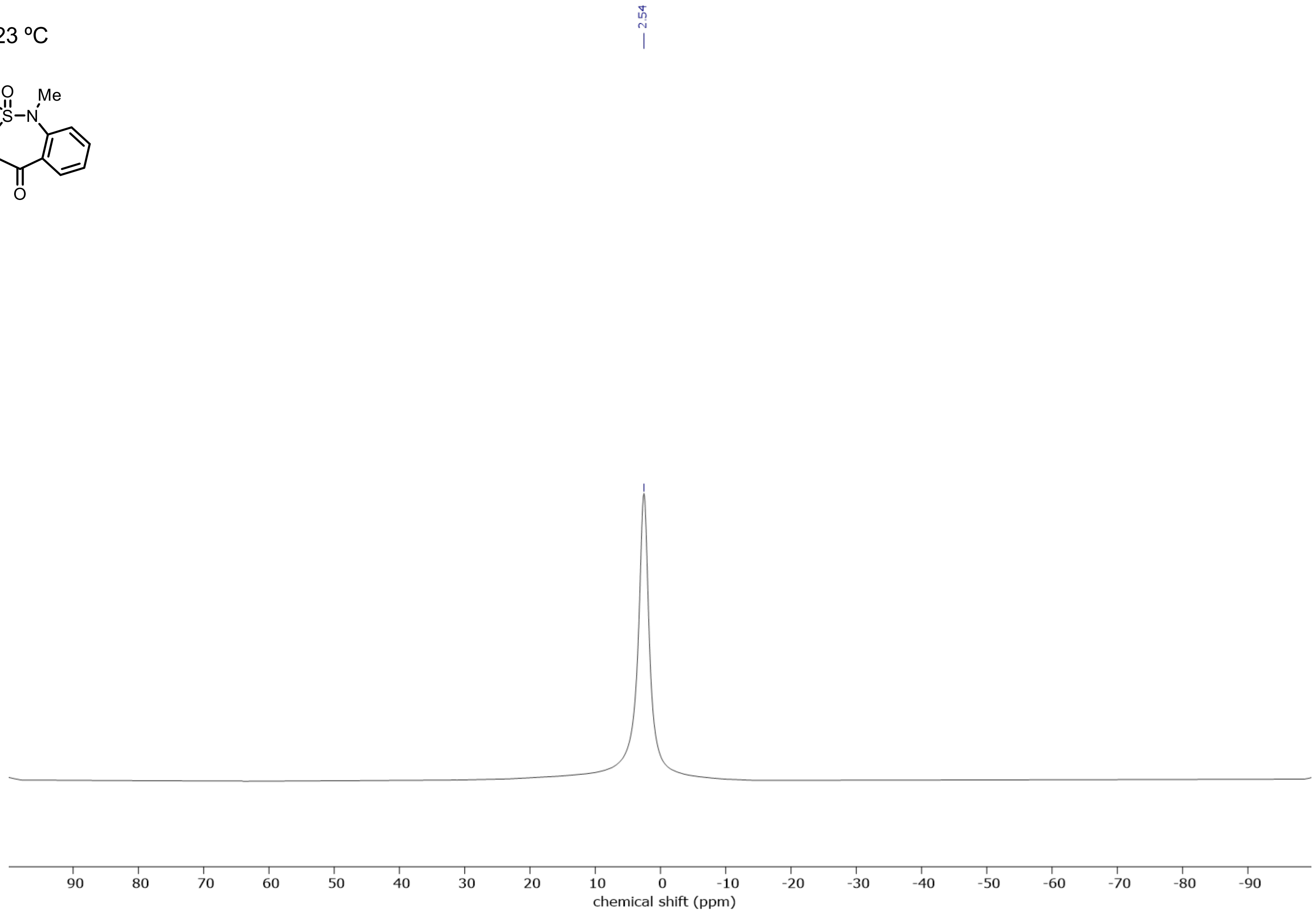

^{1}H NMR OF SELENONIUM TIANEPTINE DERIVATIVE (15)

DMSO-d6, 23 °C

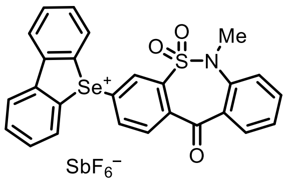

15

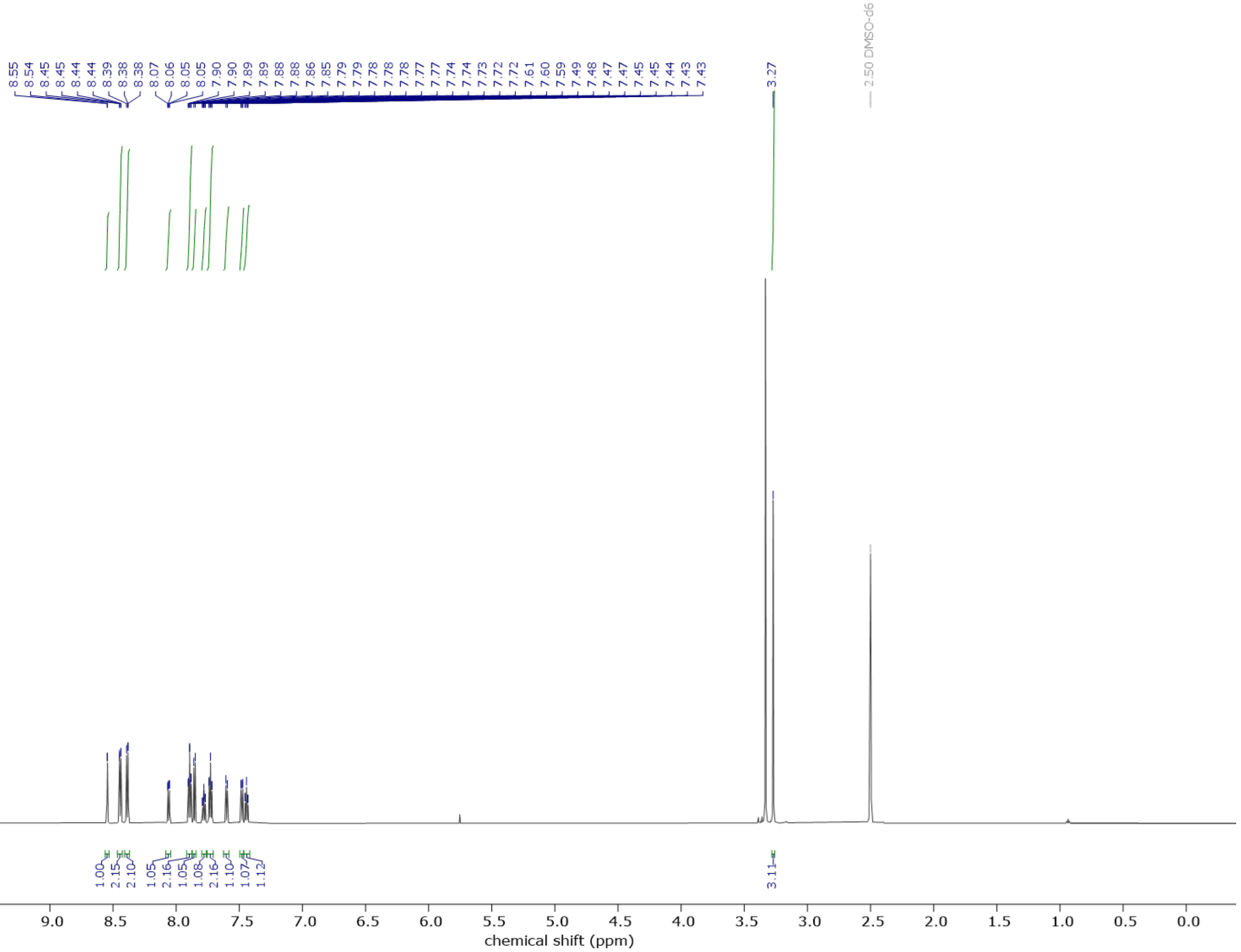

^{13}C NMR OF SELENONIUM TIANEPTINE DERIVATIVE (15)

DMSO-d6, 23 ºC

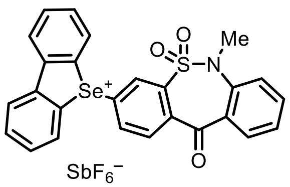

15

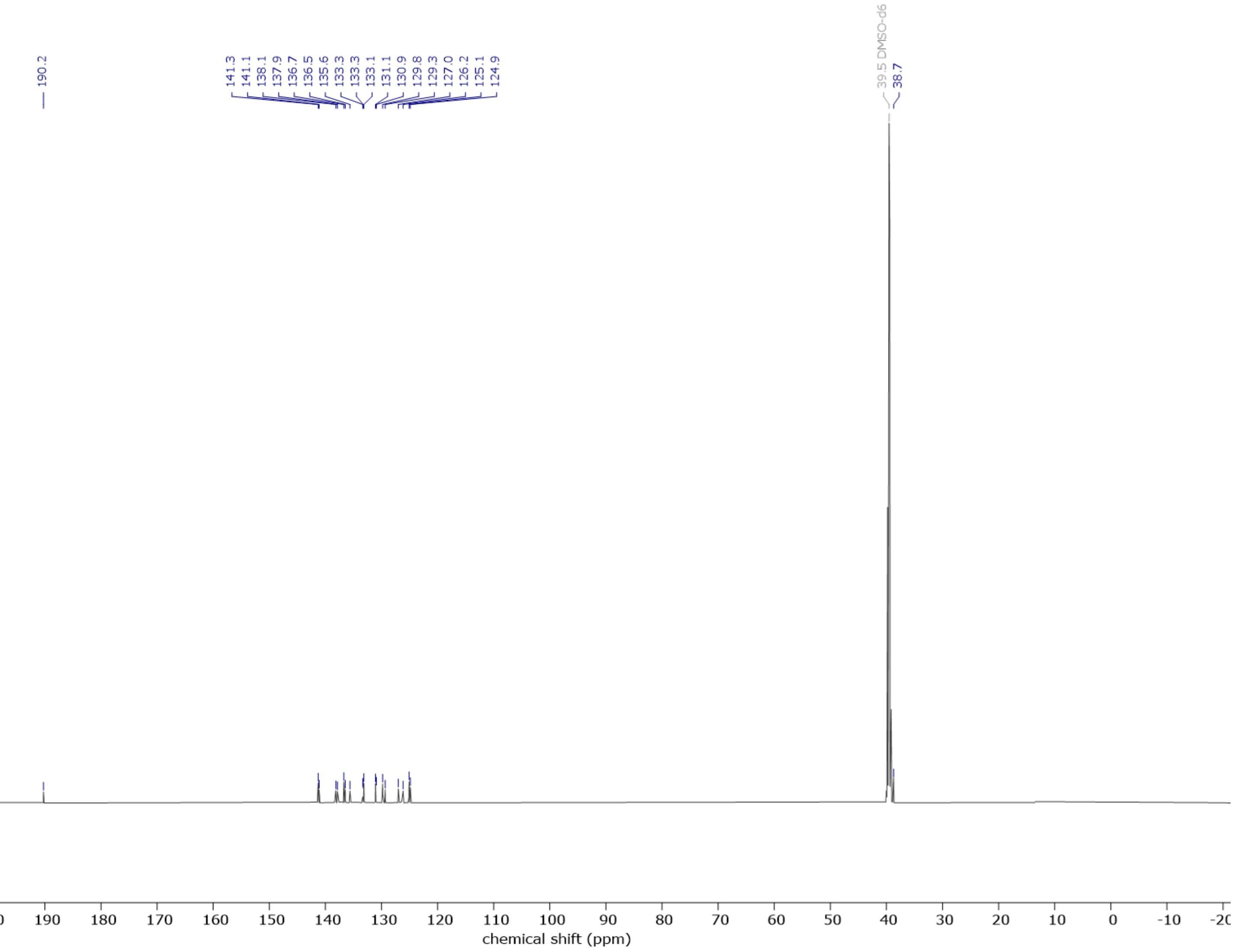

^{19}F NMR OF SELENONIUM TIANEPTINE DERIVATIVE (15)

DMSO-d6, 23 ºC

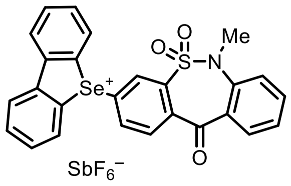

15

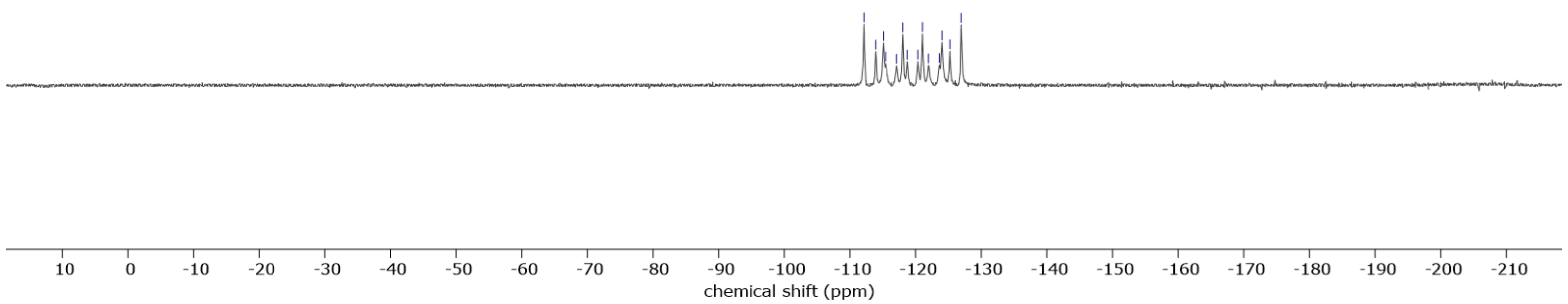

^{77}Se NMR of selenonium tianeptine derivative (15)

DMSO-d6, 23 ºC

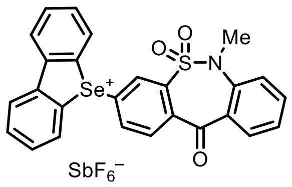

15

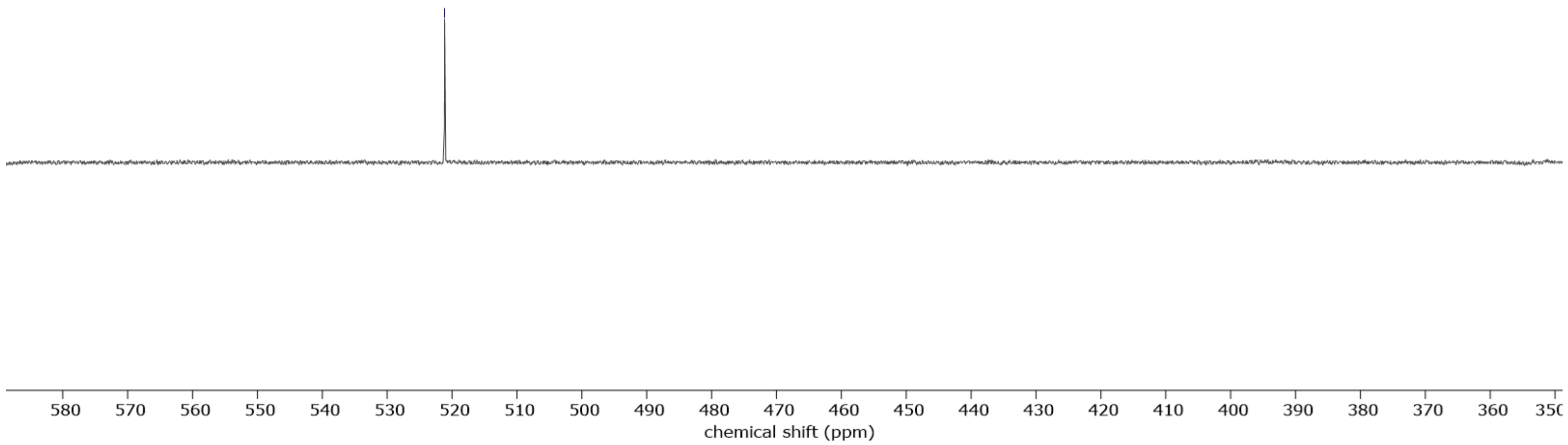

^{1}H NMR OF 3,3'-DI-*TERT*-BUTYL-1,1'-BIPHENYL (S1)

$CDCl_3$, 23 ºC

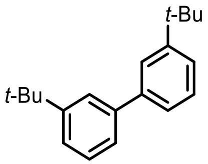

S1

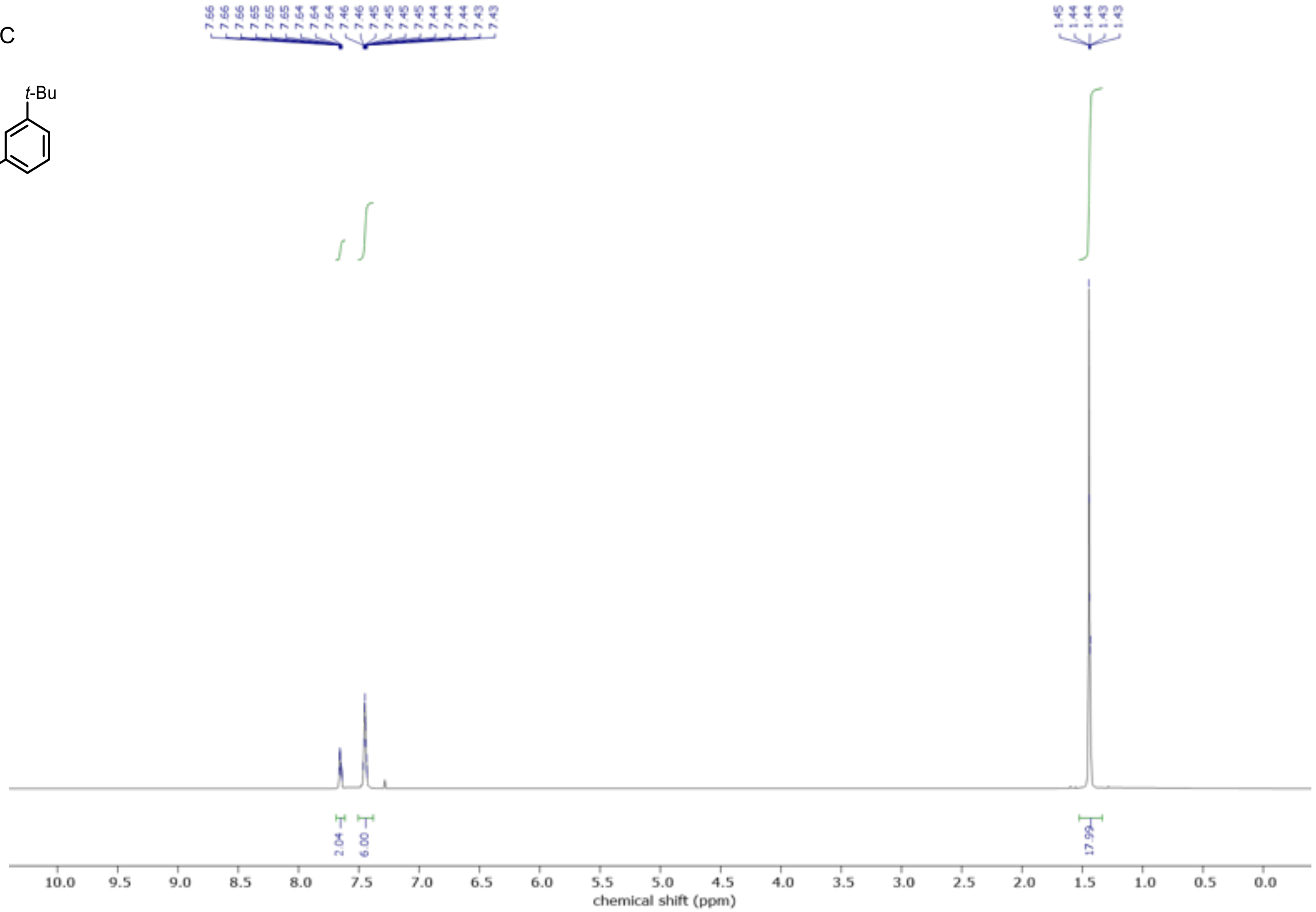

^{13}C NMR OF 3,3'-DI-*TERT*-BUTYL-1,1'-BIPHENYL (S1)

CDCl3, 23 ºC

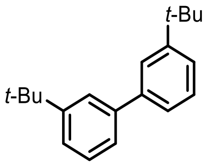

S1

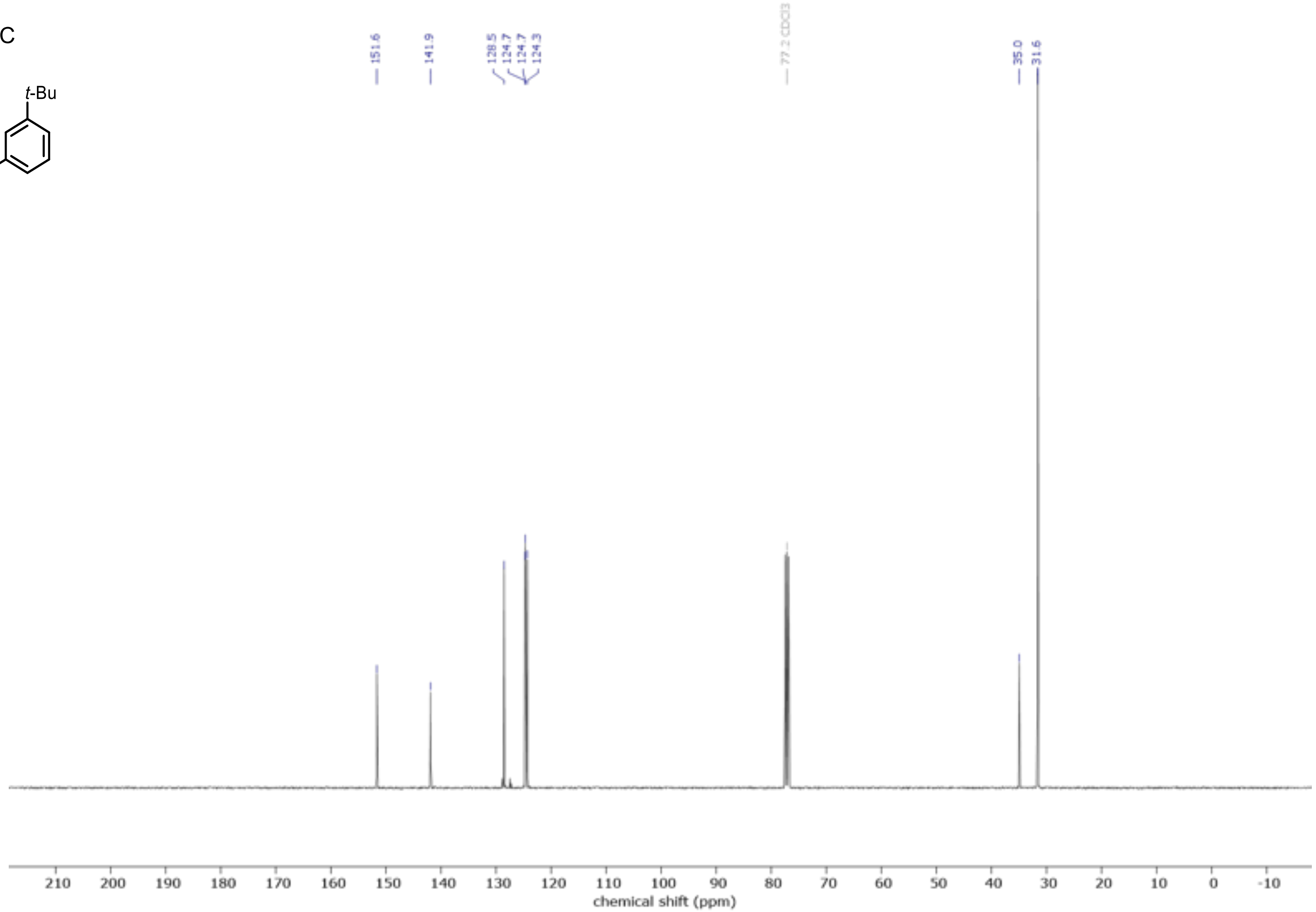

^{1}H NMR OF OCTAMETHYL-OCTAHYDRO BINAPHTHALENE (S2)

$CDCl_3$, 23 ºC

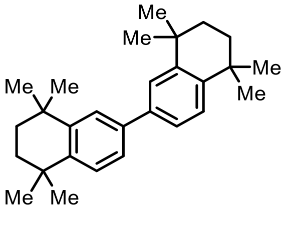

S2

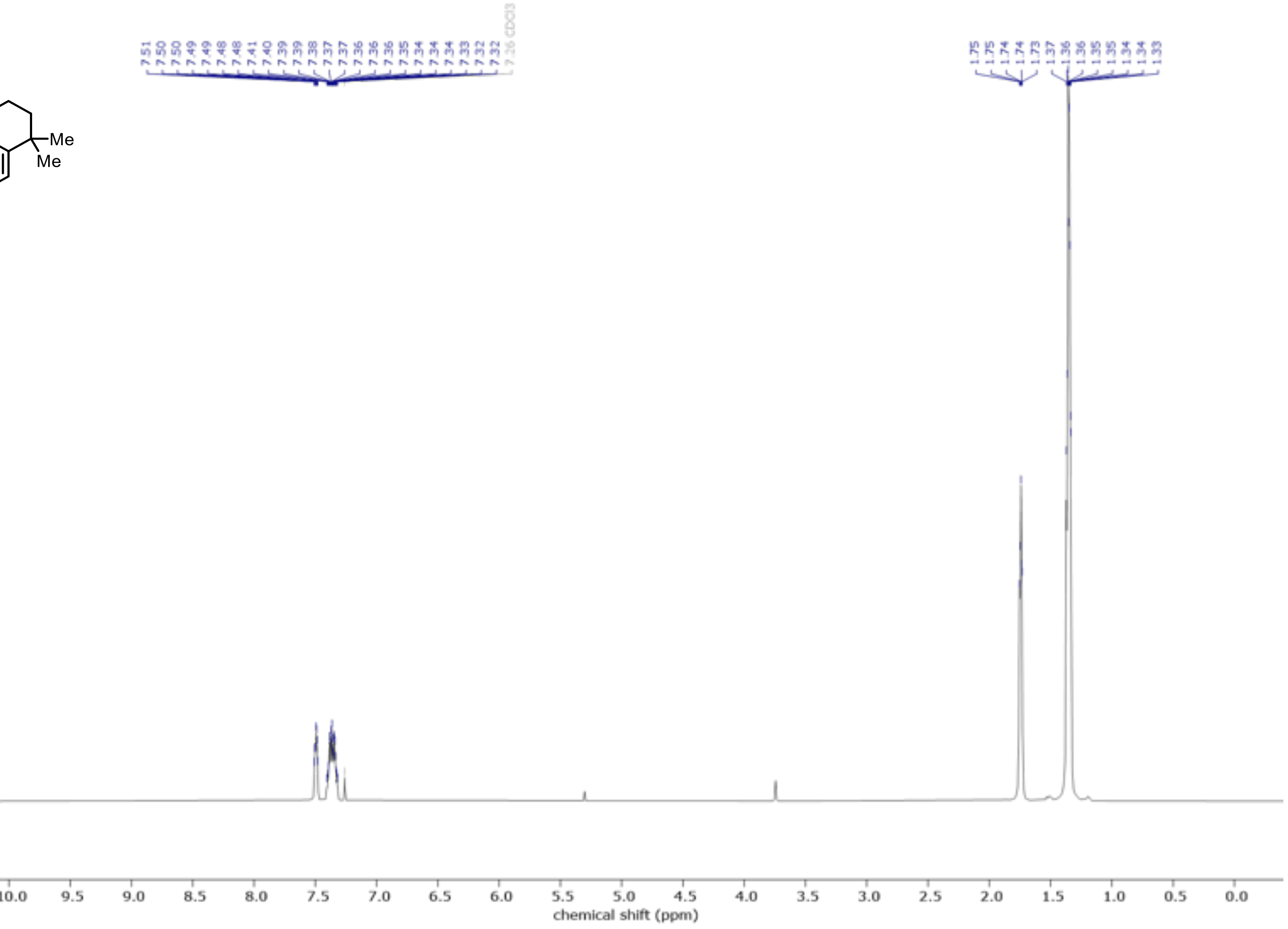

^{13}C NMR OF OCTAMETHYL-OCTAHYDRO BINAPHTHALENE (S2)

CDCl$_3$, 23 ºC

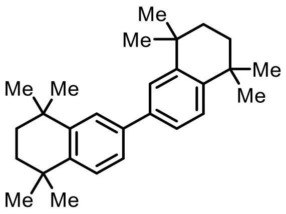

S2

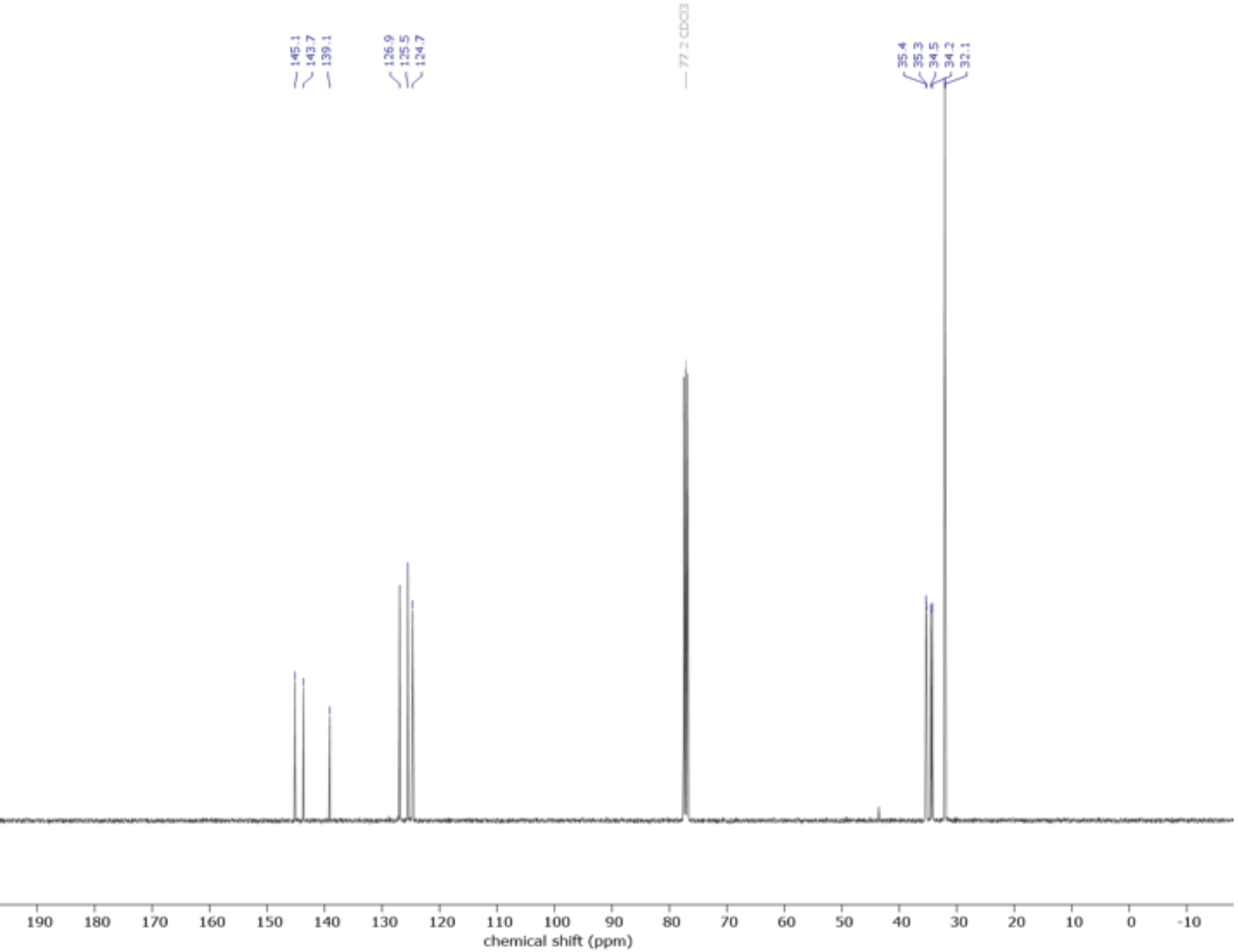

^{1}H NMR of 4,4'-bis(trifluoromethyl)-[1,1'-biphenyl]-2-amine (S3)

$CDCl_3$, 23 ºC

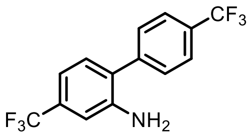

S3

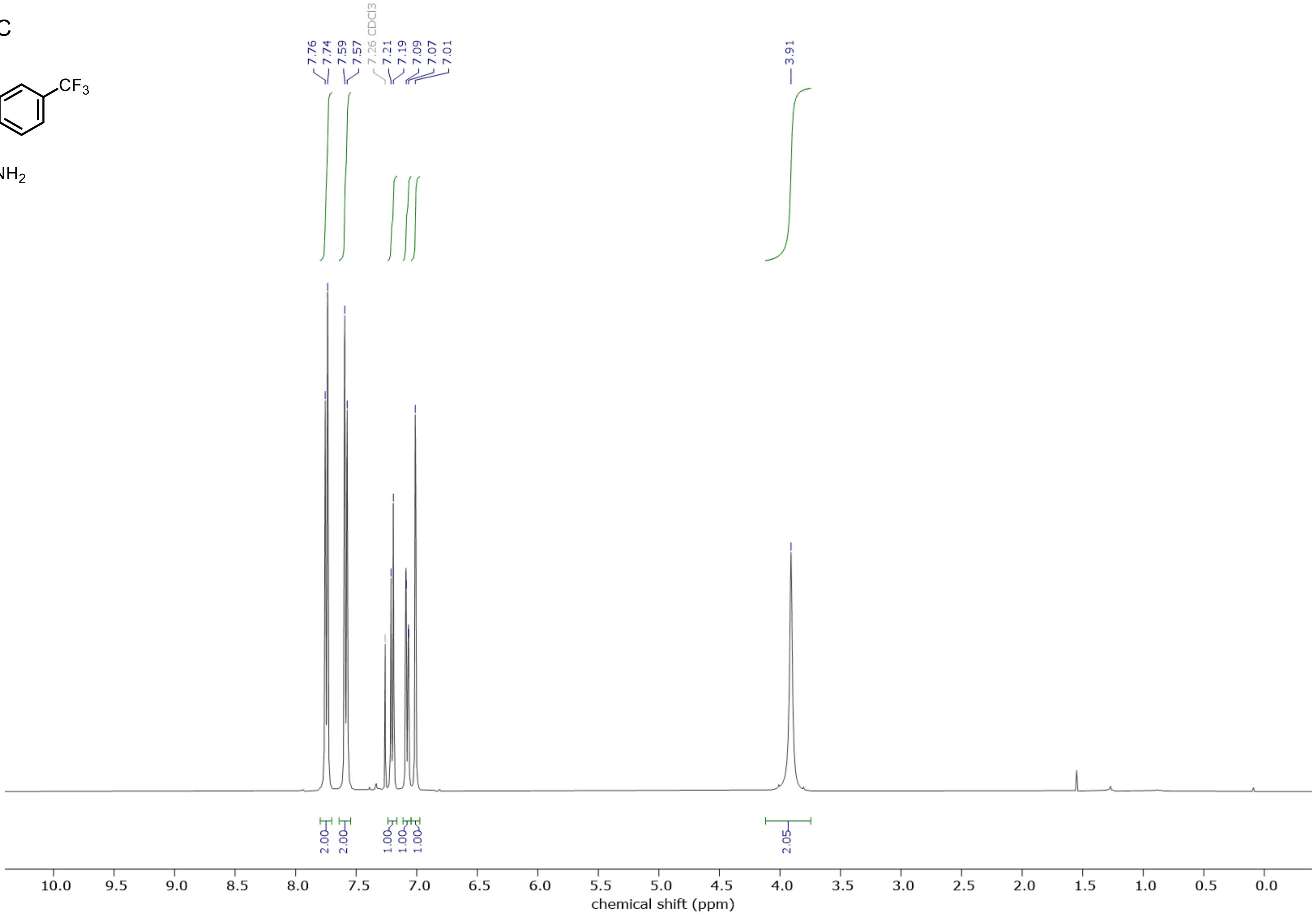

^{13}C NMR OF 4,4'-BIS(TRIFLUOROMETHYL)-[1,1'-BIPHENYL]-2-AMINE (S3)

CDCl3, 23 °C

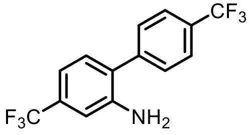

S3

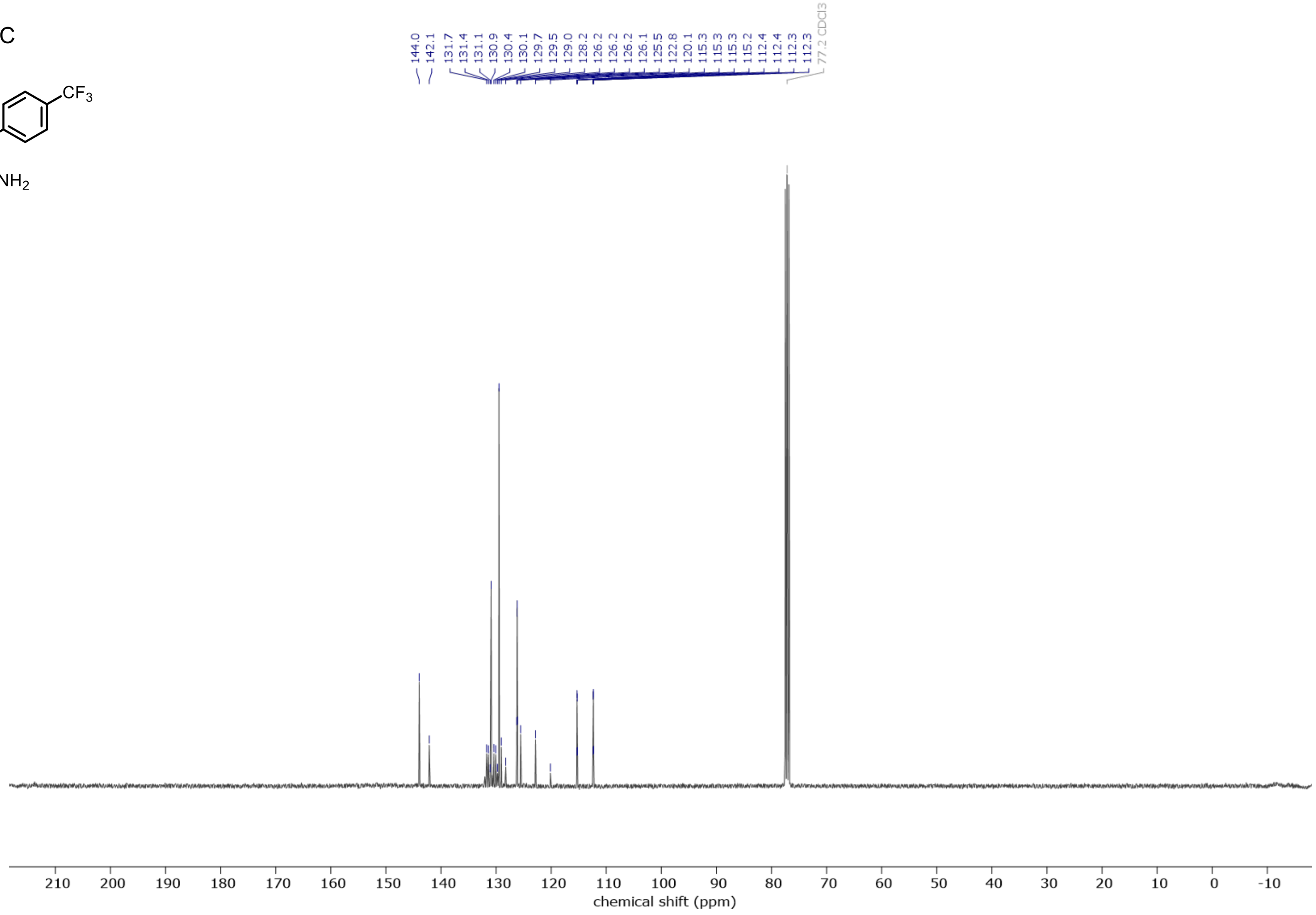

^{19}F NMR of 4,4'-bis(trifluoromethyl)-[1,1'-biphenyl]-2-amine (S3)

CD3CN, 23 ºC

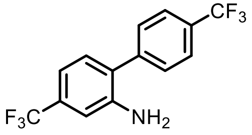

S3

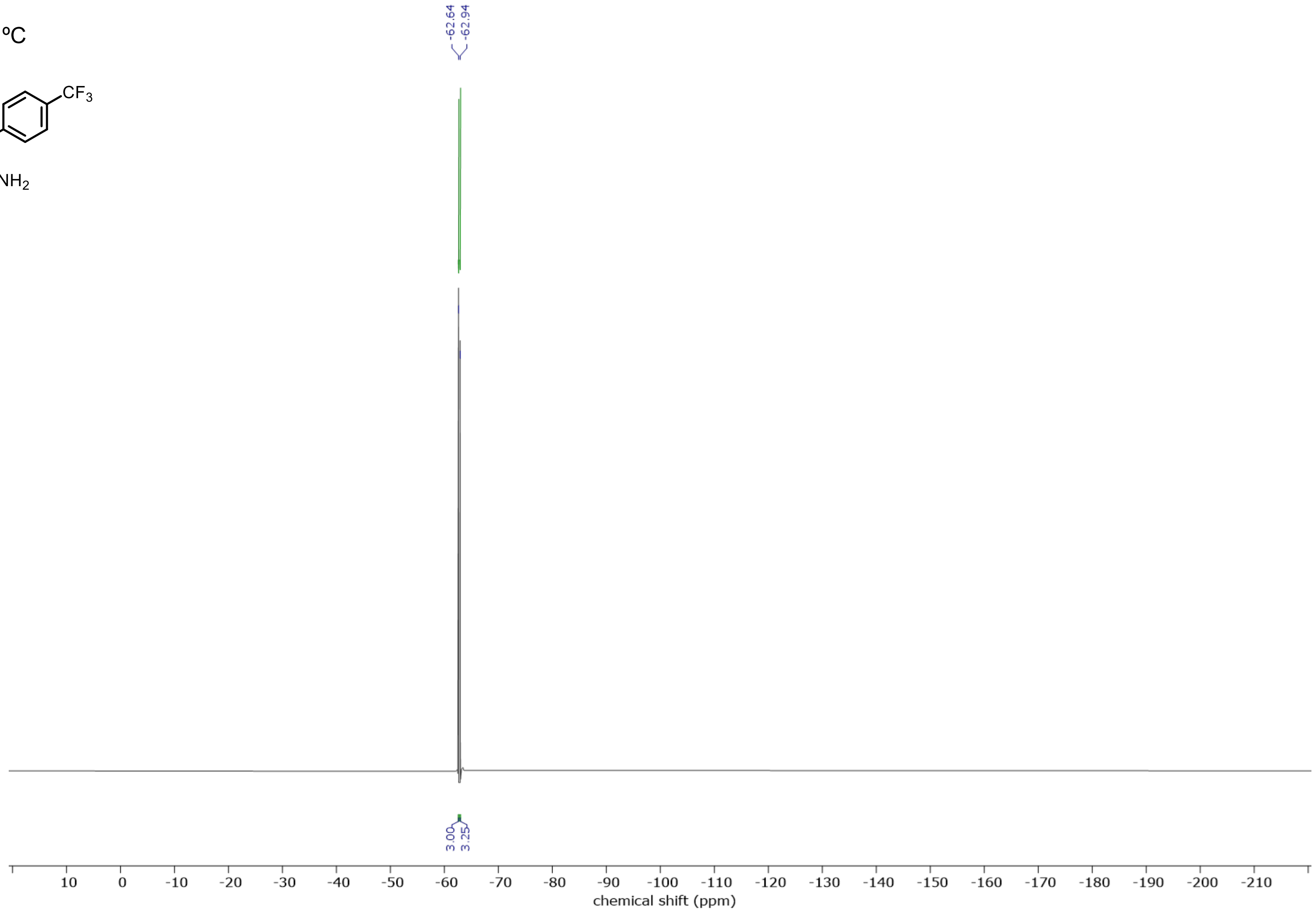

^{1}H NMR of 2-iodo-4,4'-bis(trifluoromethyl)-1,1'-biphenyl (S4)

CDCl3, 23 ºC

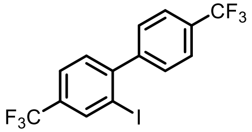
S4

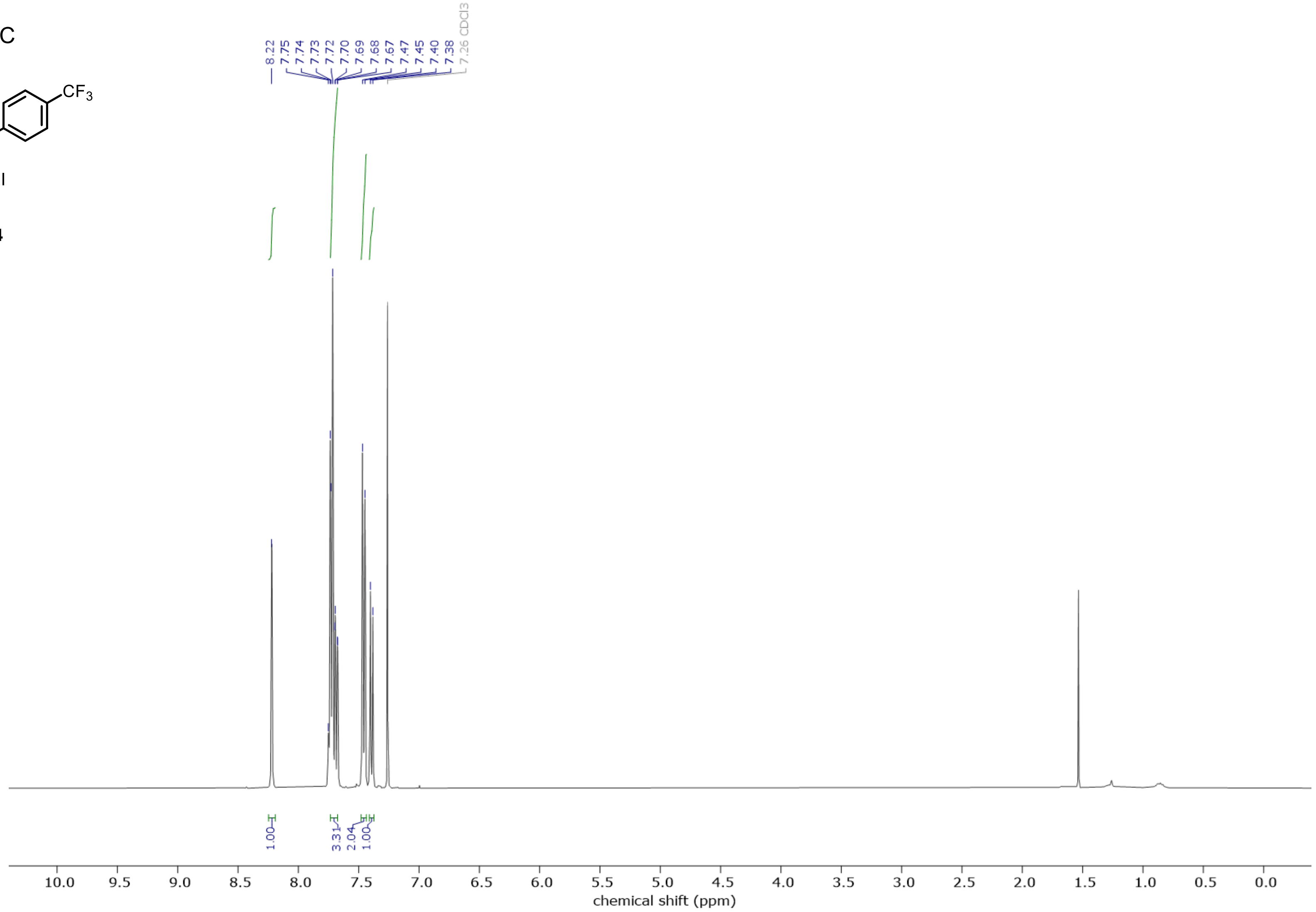

^{13}C NMR OF 2-IODO-4,4'-BIS(TRIFLUOROMETHYL)-1,1'-BIPHENYL (S4)

$CDCl_3$, 23 ºC

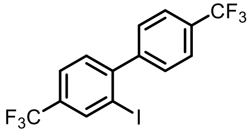

S4

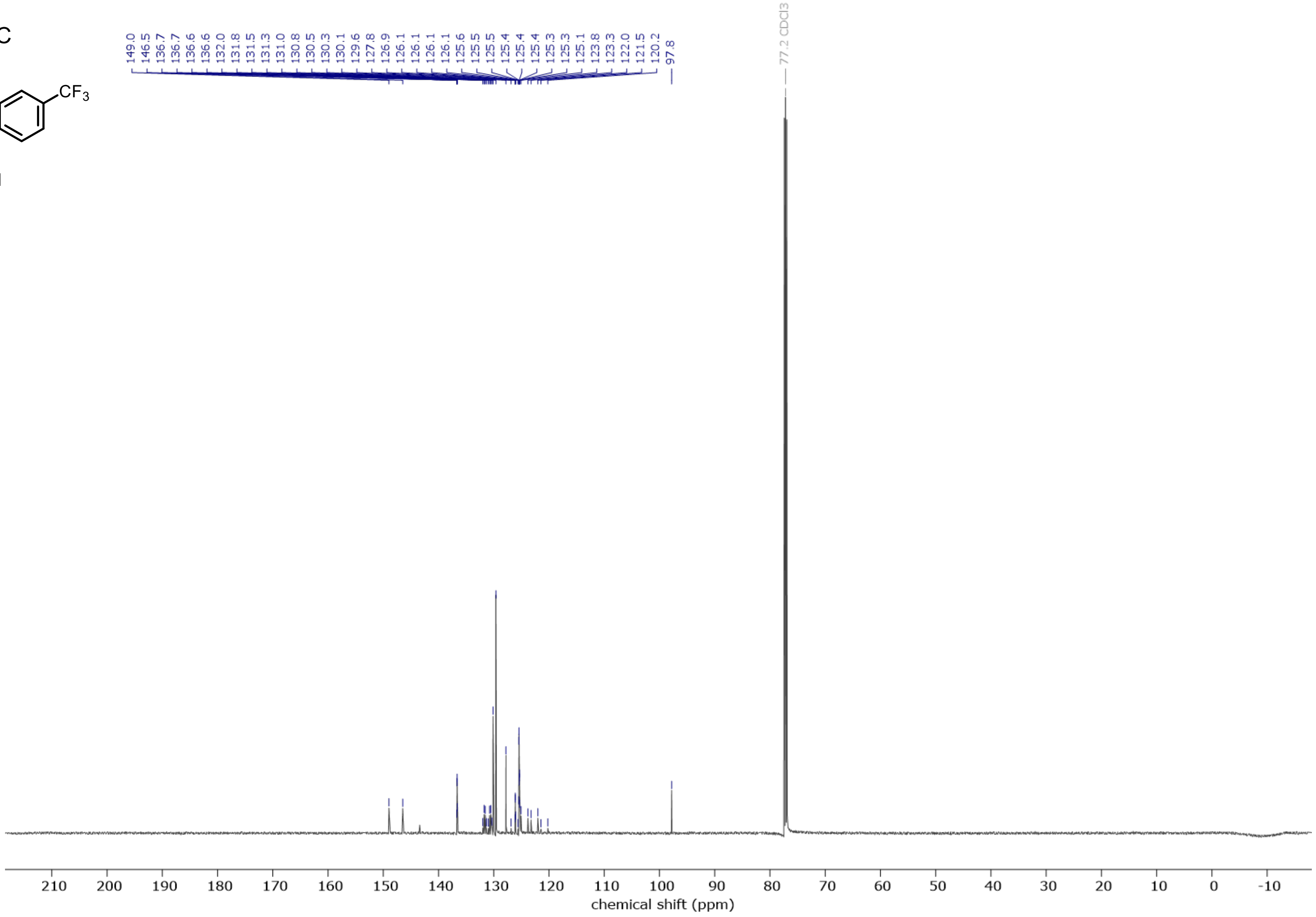

^{19}F NMR OF 2-IODO-4,4'-BIS(TRIFLUOROMETHYL)-1,1'-BIPHENYL (S4)

CD3CN, 23 ºC

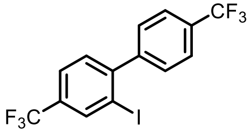

S4

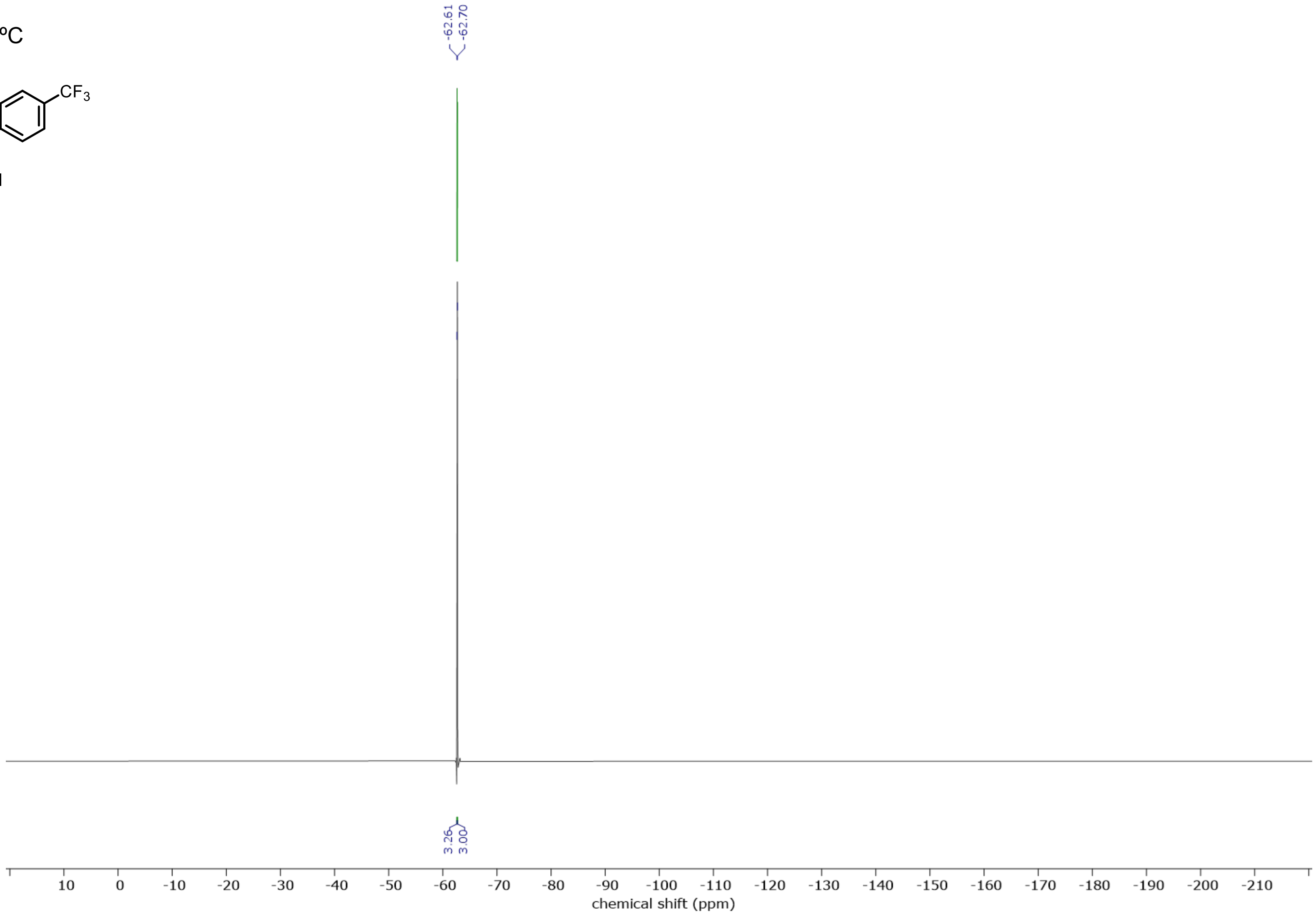

^{1}H NMR OF 3,7-BIS(TRIFLUOROMETHYL)DIBENZO[B,D]IODOL-5-IUM TRIFLUOROMETHANESULFONATE (S5)

DMSO-d6, 23 ºC

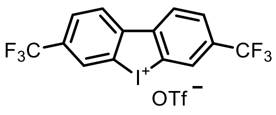

S5

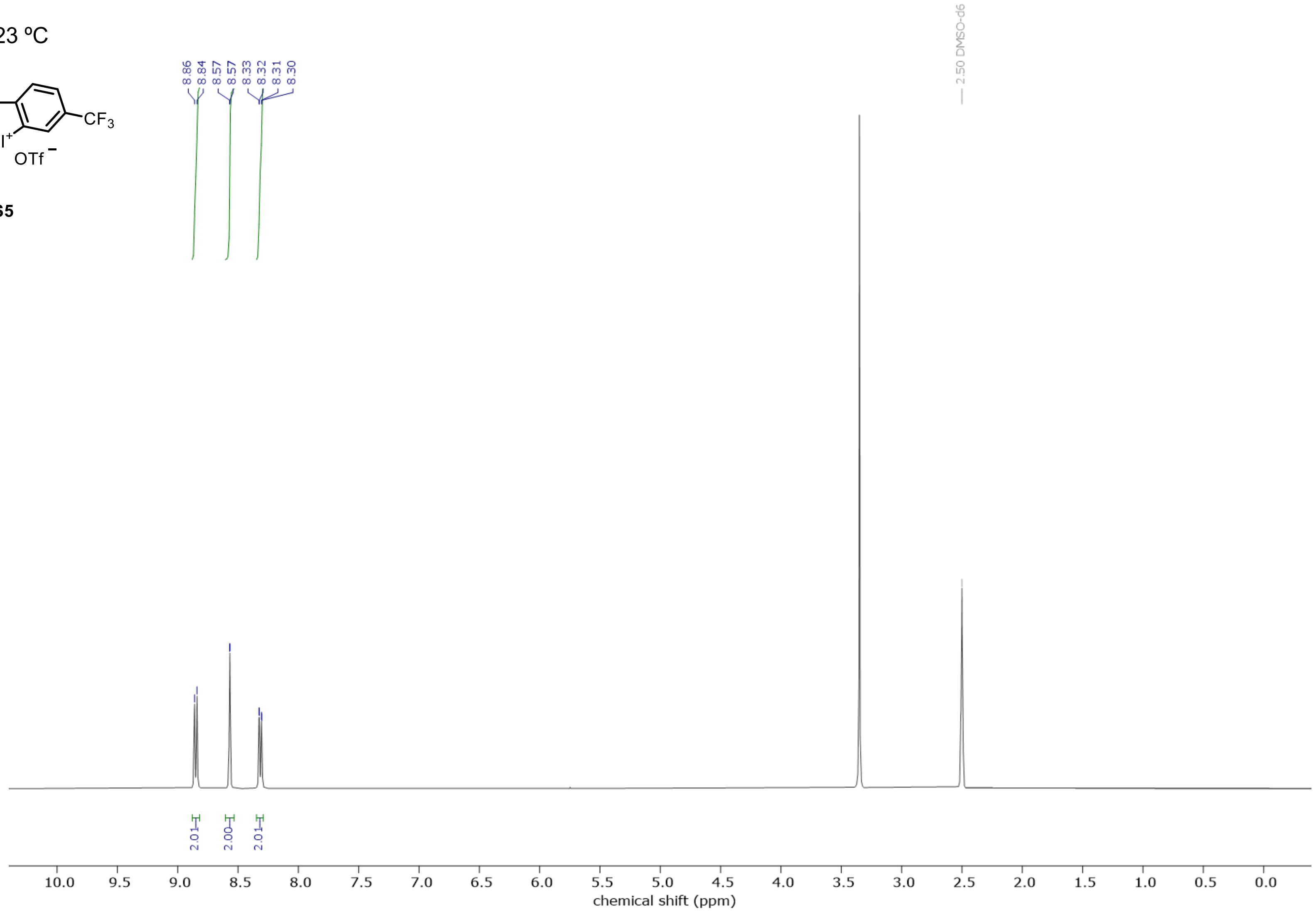

^{13}C NMR of 3,7-bis(trifluoromethyl)dibenzo[*b*,*d*]iodol-5-ium trifluoromethanesulfonate (S5)

DMSO-d6, 23 ºC

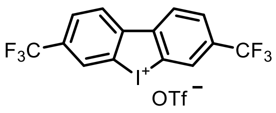

S5

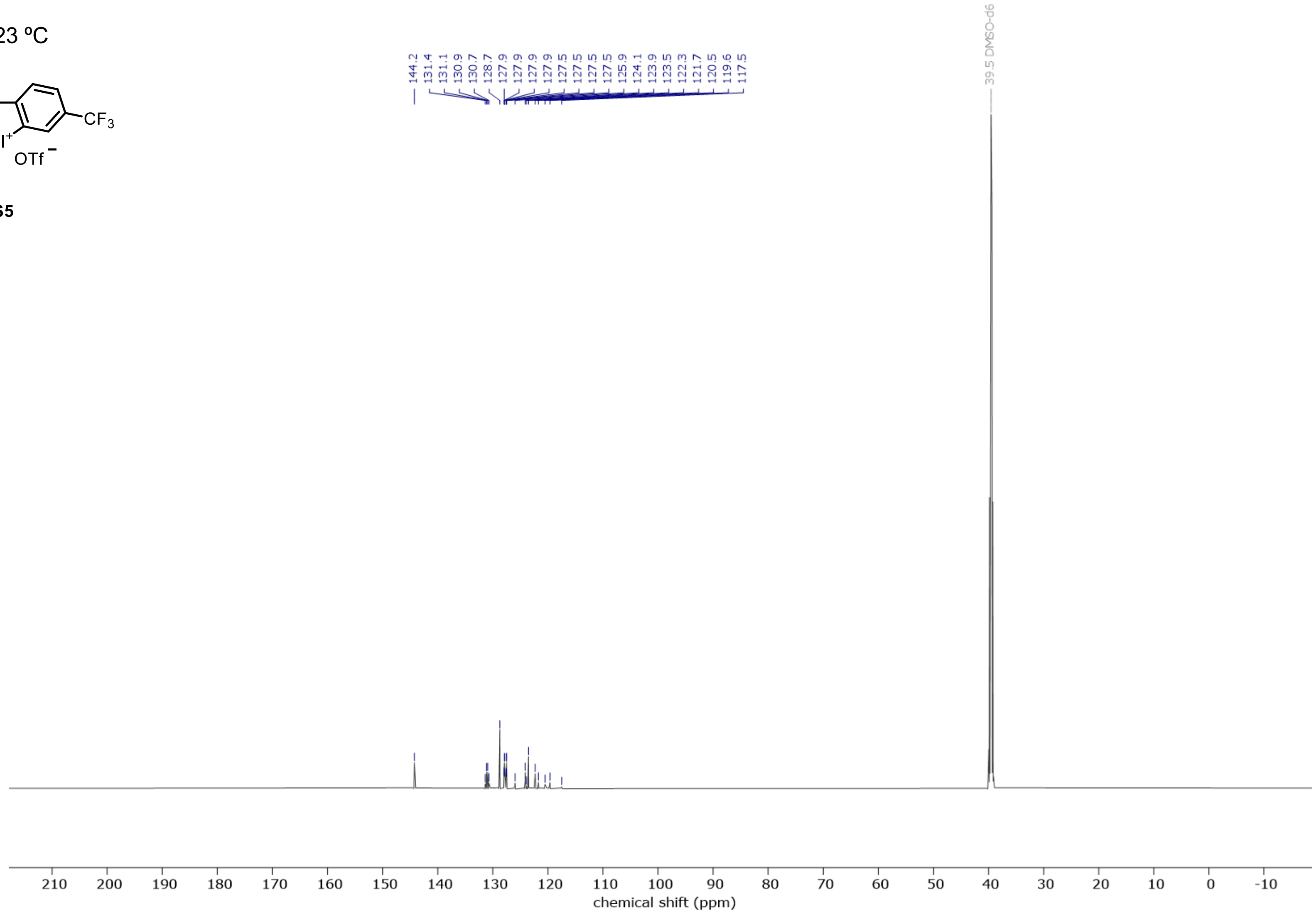

^{19}F NMR OF 3,7-BIS(TRIFLUOROMETHYL)DIBENZO[*B*,*D*]IODOL-5-IUM TRIFLUOROMETHANESULFONATE (S5)

DMSO-d6, 23 ºC

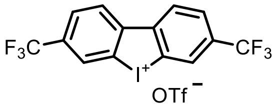

S5

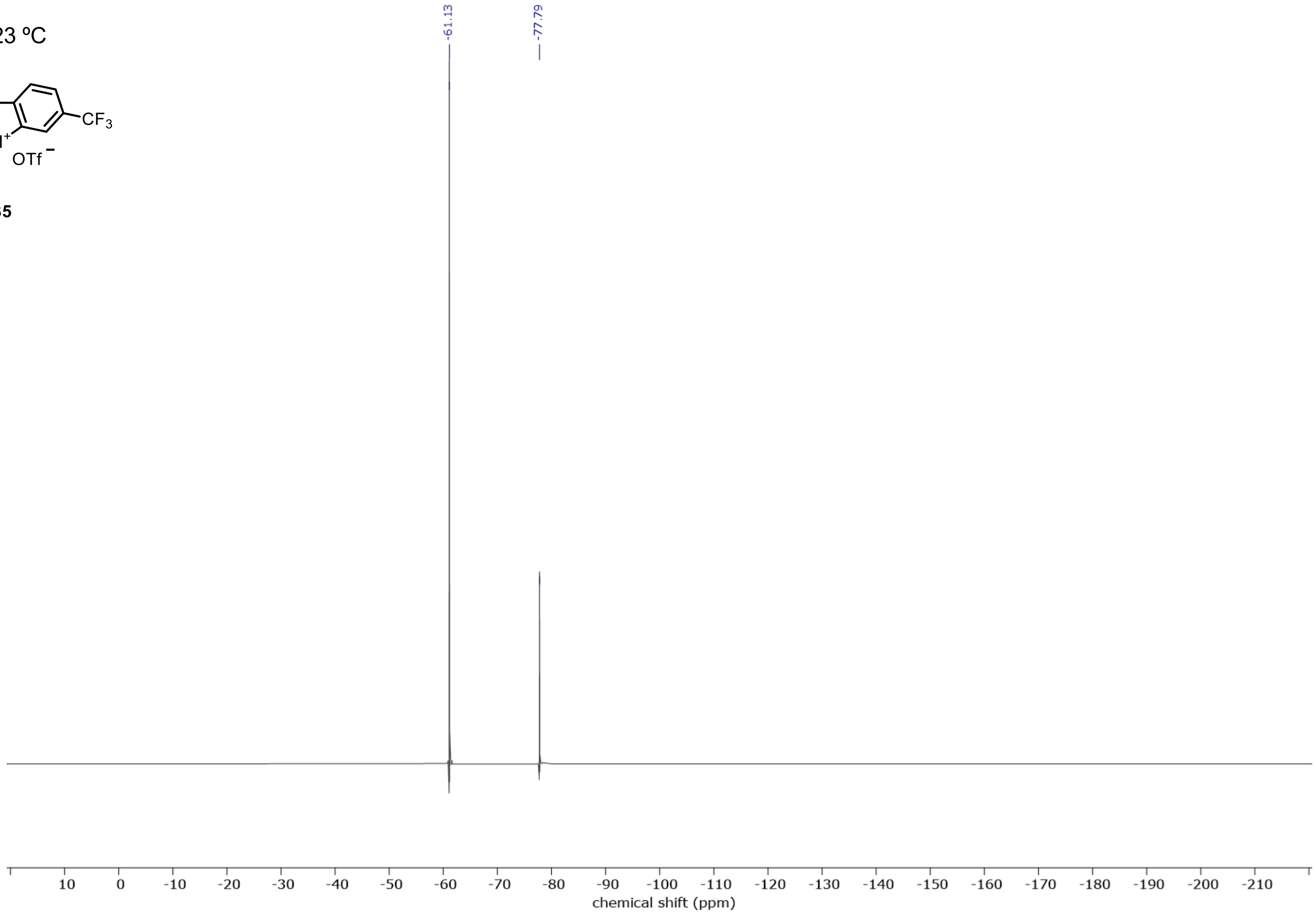

^{1}H NMR OF DIPHENYLSELENIDE (S6)

CDCl3, 23 ºC

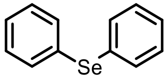

S6

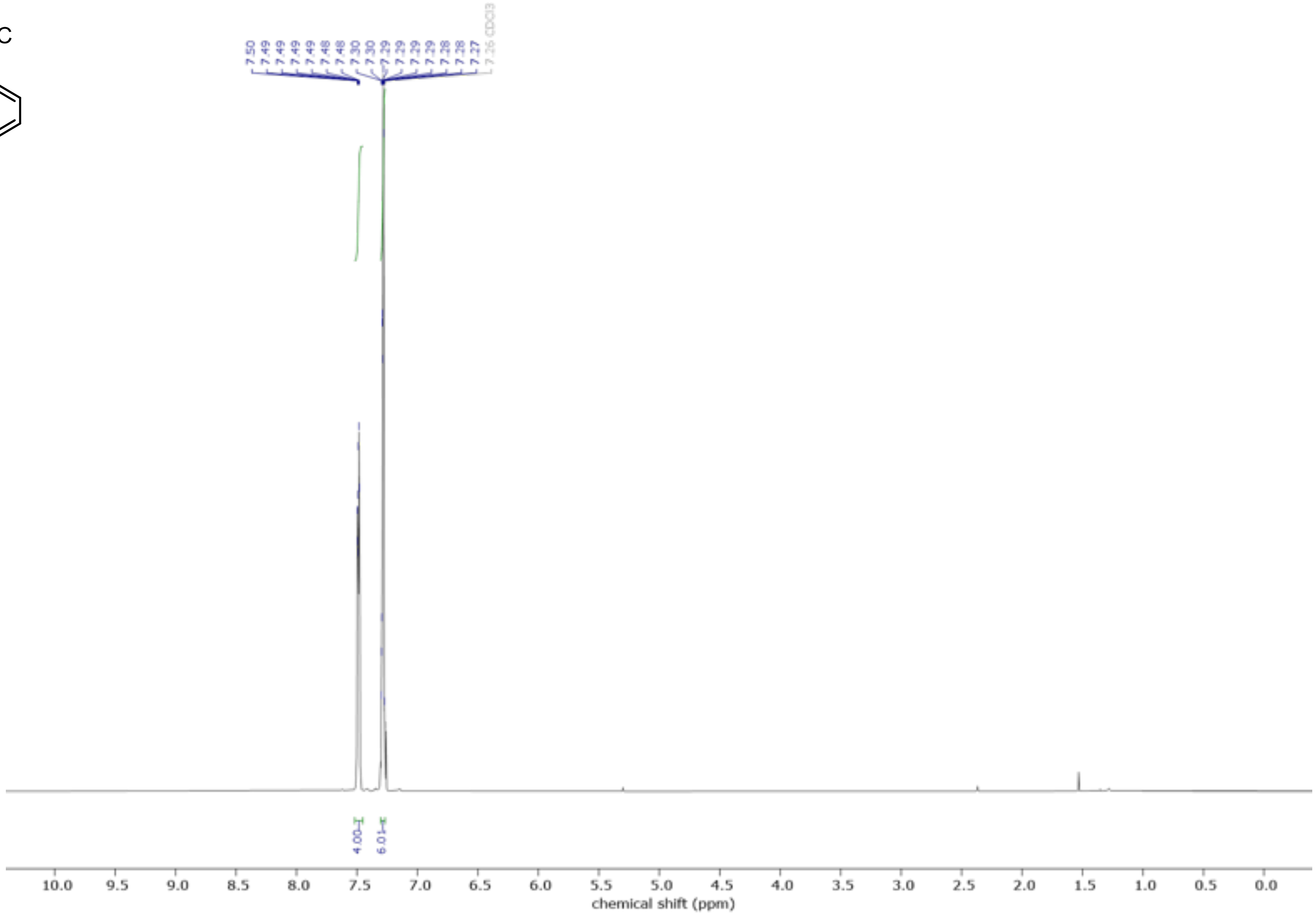

^{13}C NMR OF DIPHENYLSELENIDE (S6)

CDCl3, 23 ºC

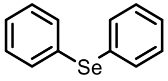

S6

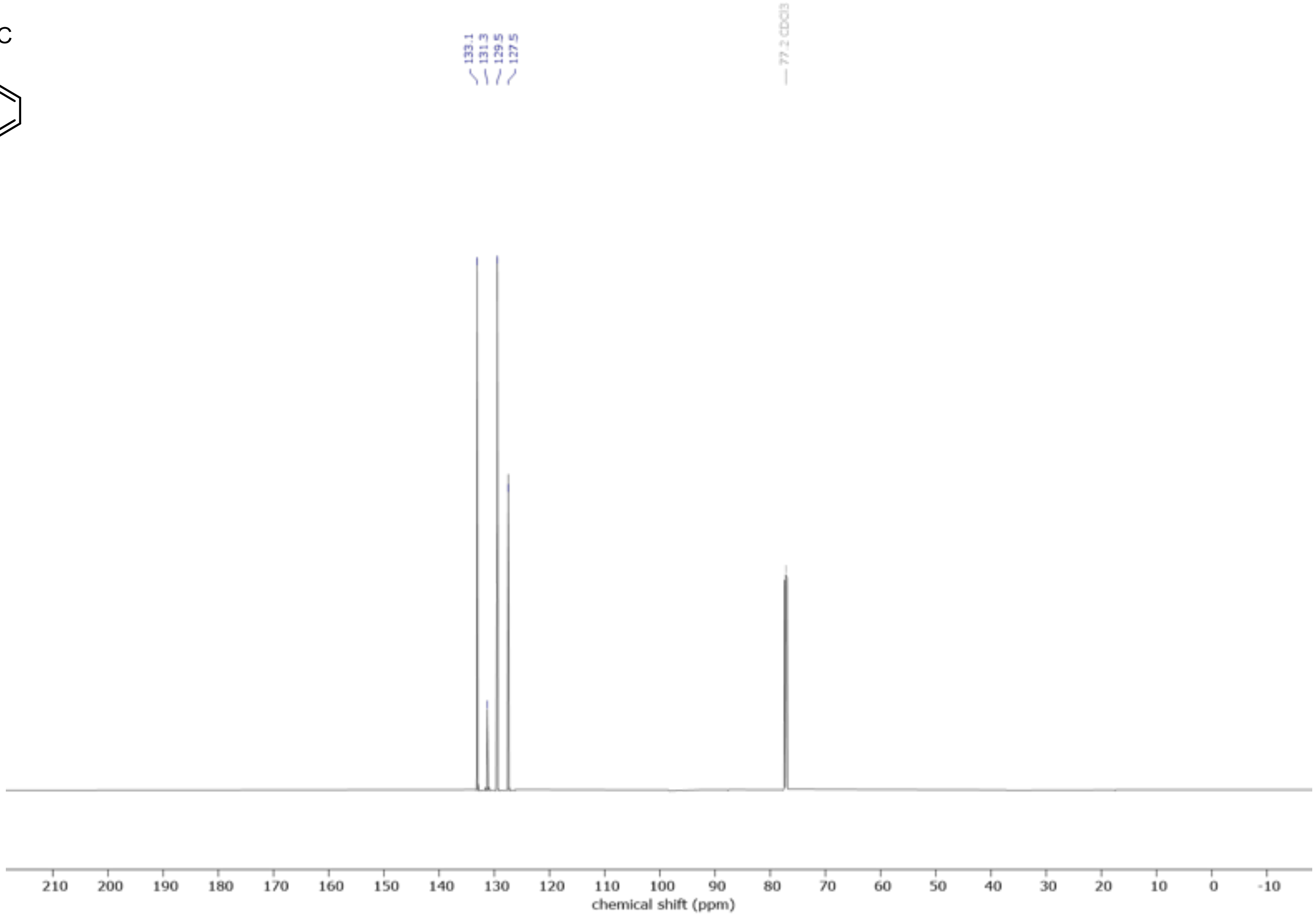

^{77}Se NMR of diphenylselenide (S6)

CDCl$_3$, 23 ºC

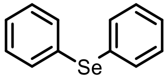

S6

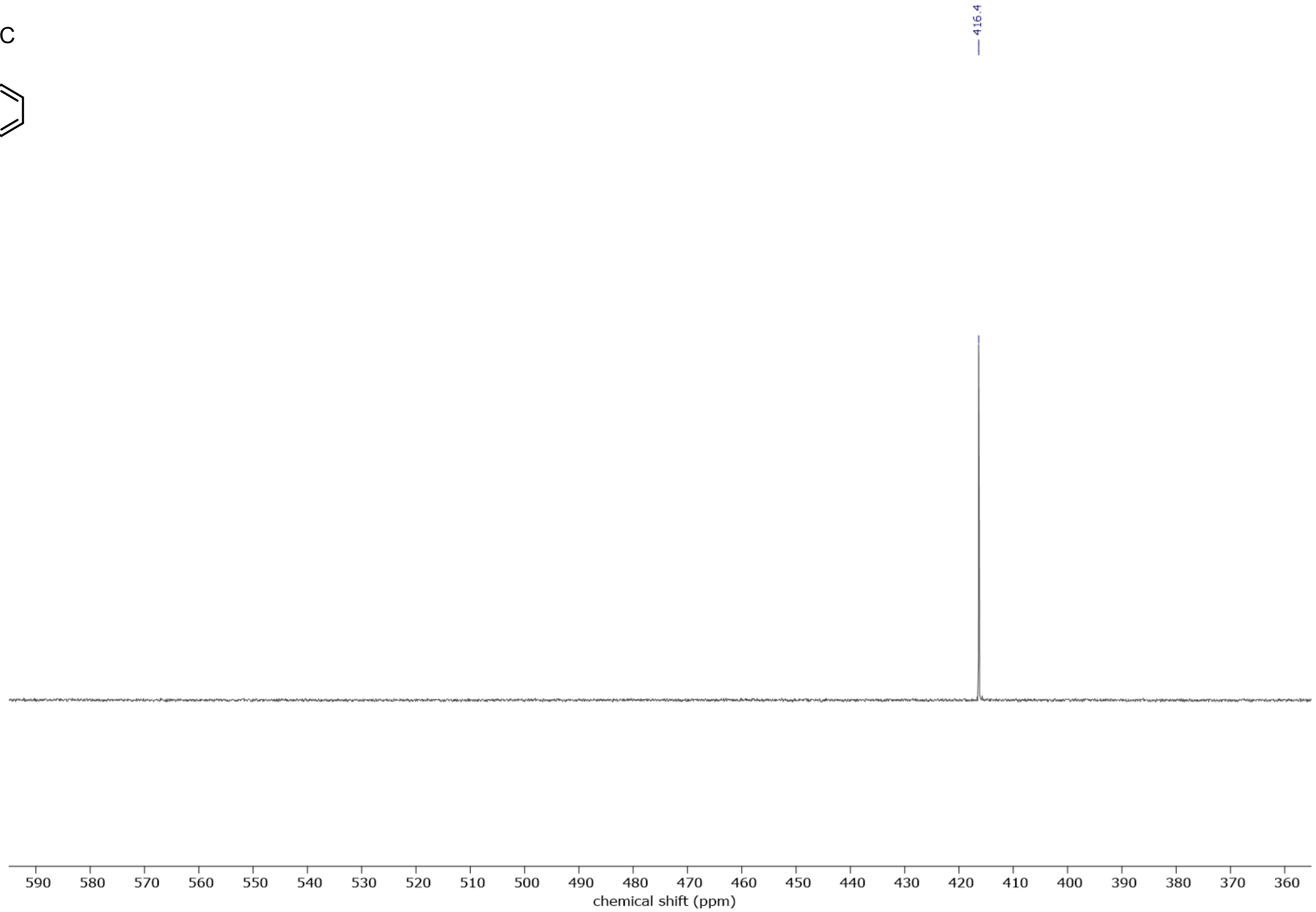

^{1}H NMR OF DIBENZYLSELENIDE (S7)

CDCl3, 23 ºC

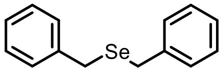

S7

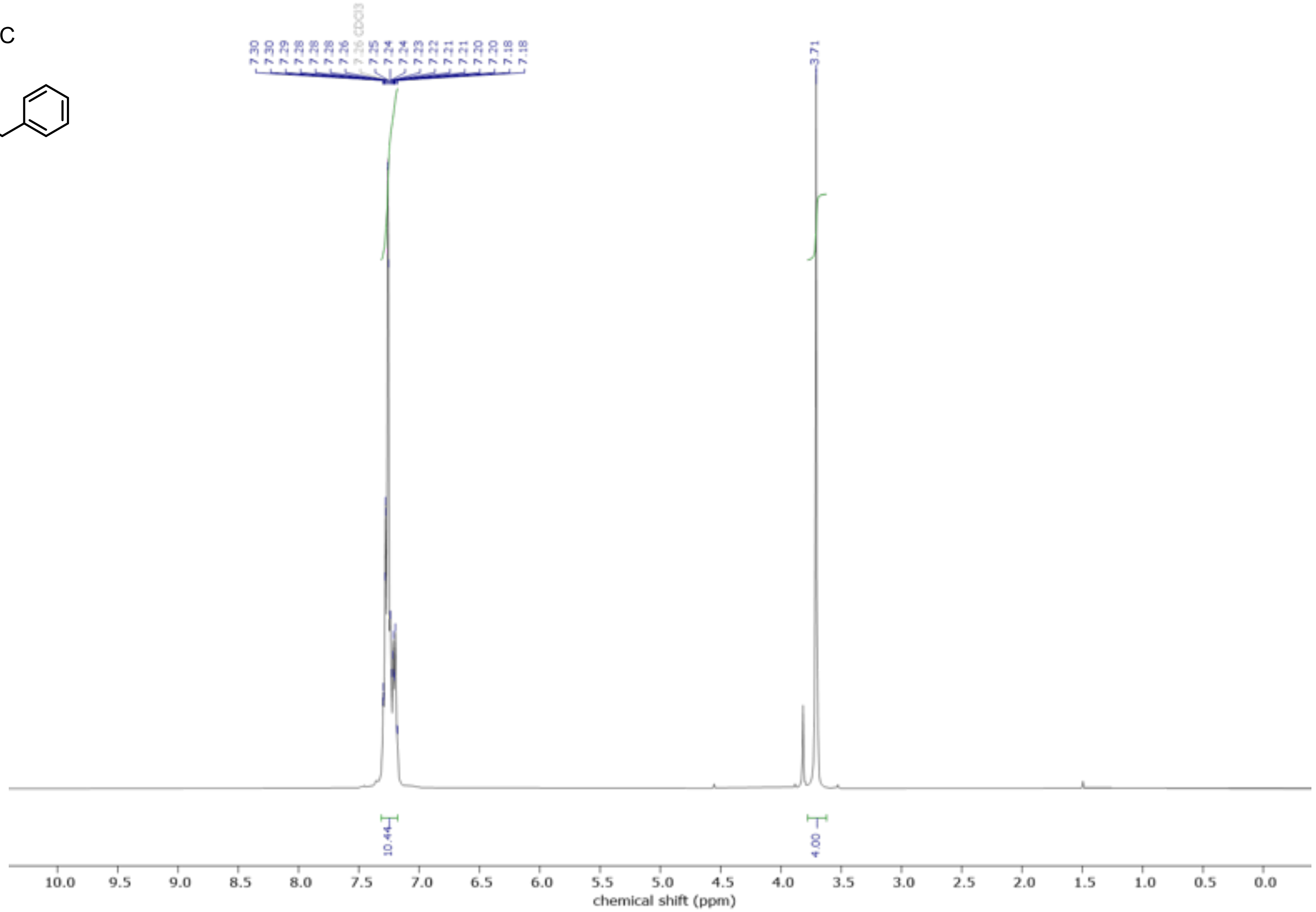

^{13}C NMR OF DIBENZYLSELENIDE (S7)

$CDCl_3$, 23 ºC

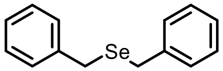

S7

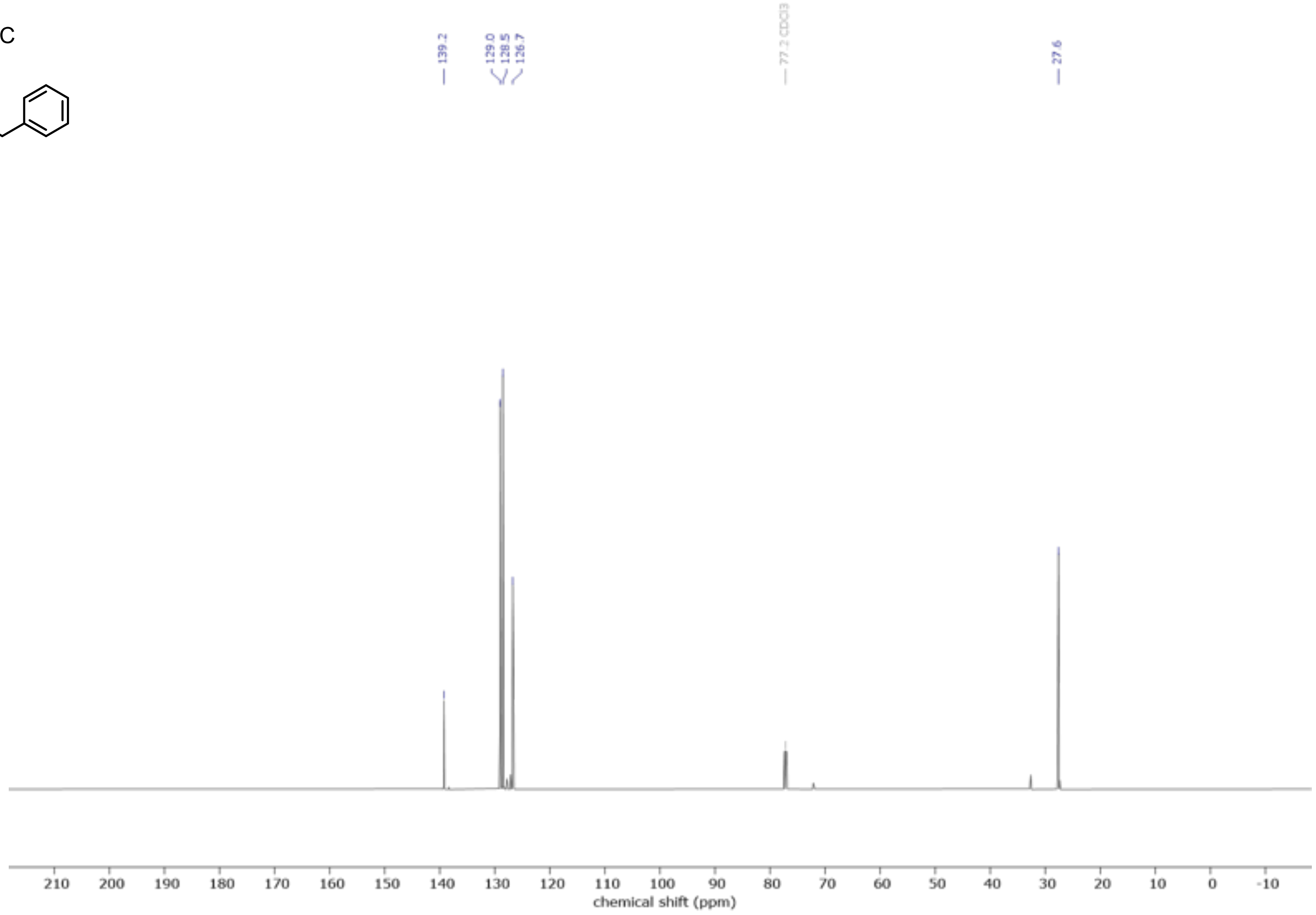

^{77}Se NMR of dibenzylselenide (S7)

CDCl$_3$, 23 ºC

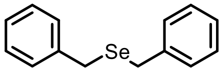

S7

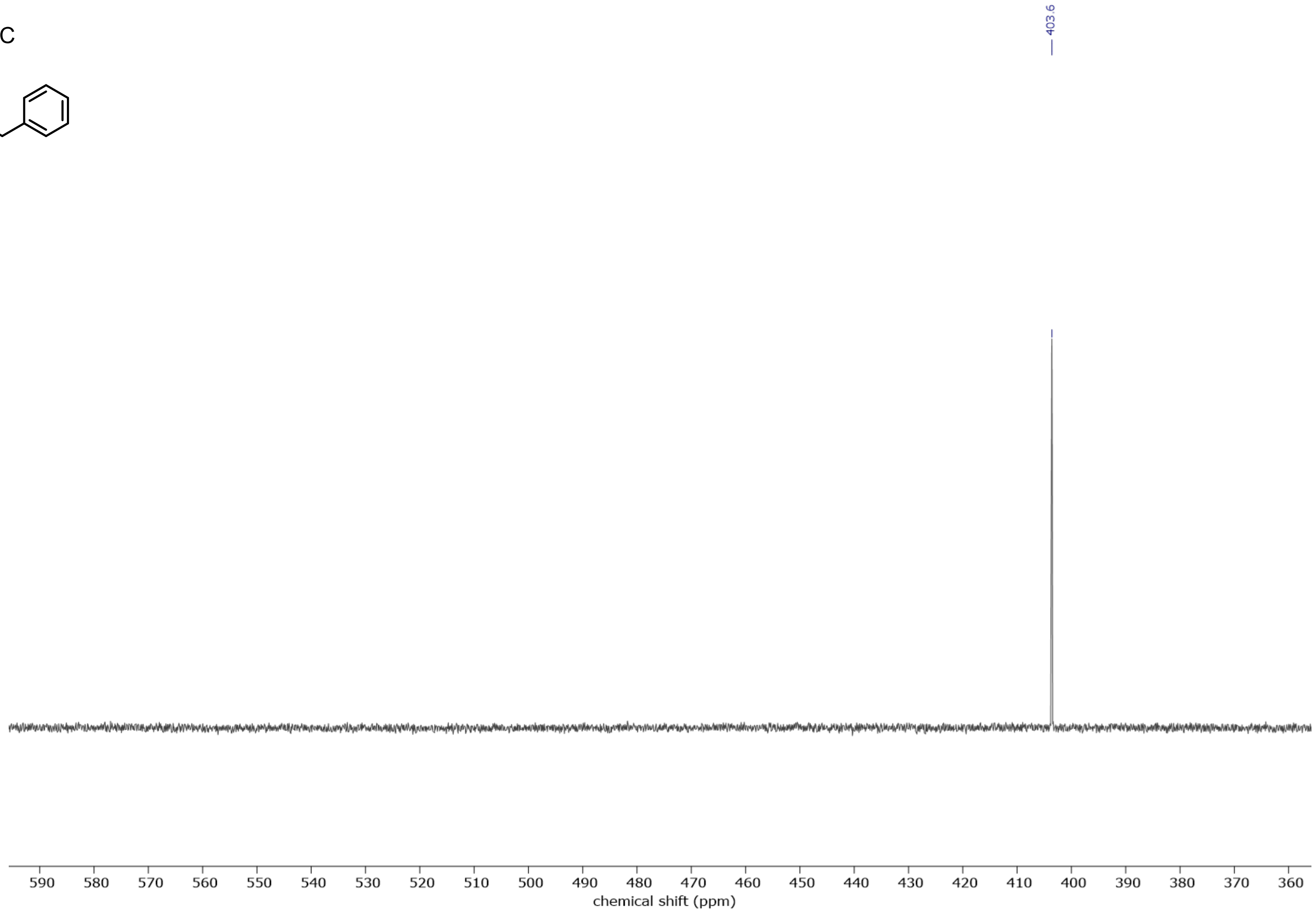

^{1}H NMR of 3,7-di-*tert*-butyldibenzo[*b*,*d*]thiophene (S8)

$CDCl_3$, 23 ºC

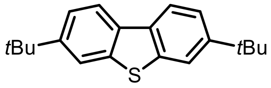

S8

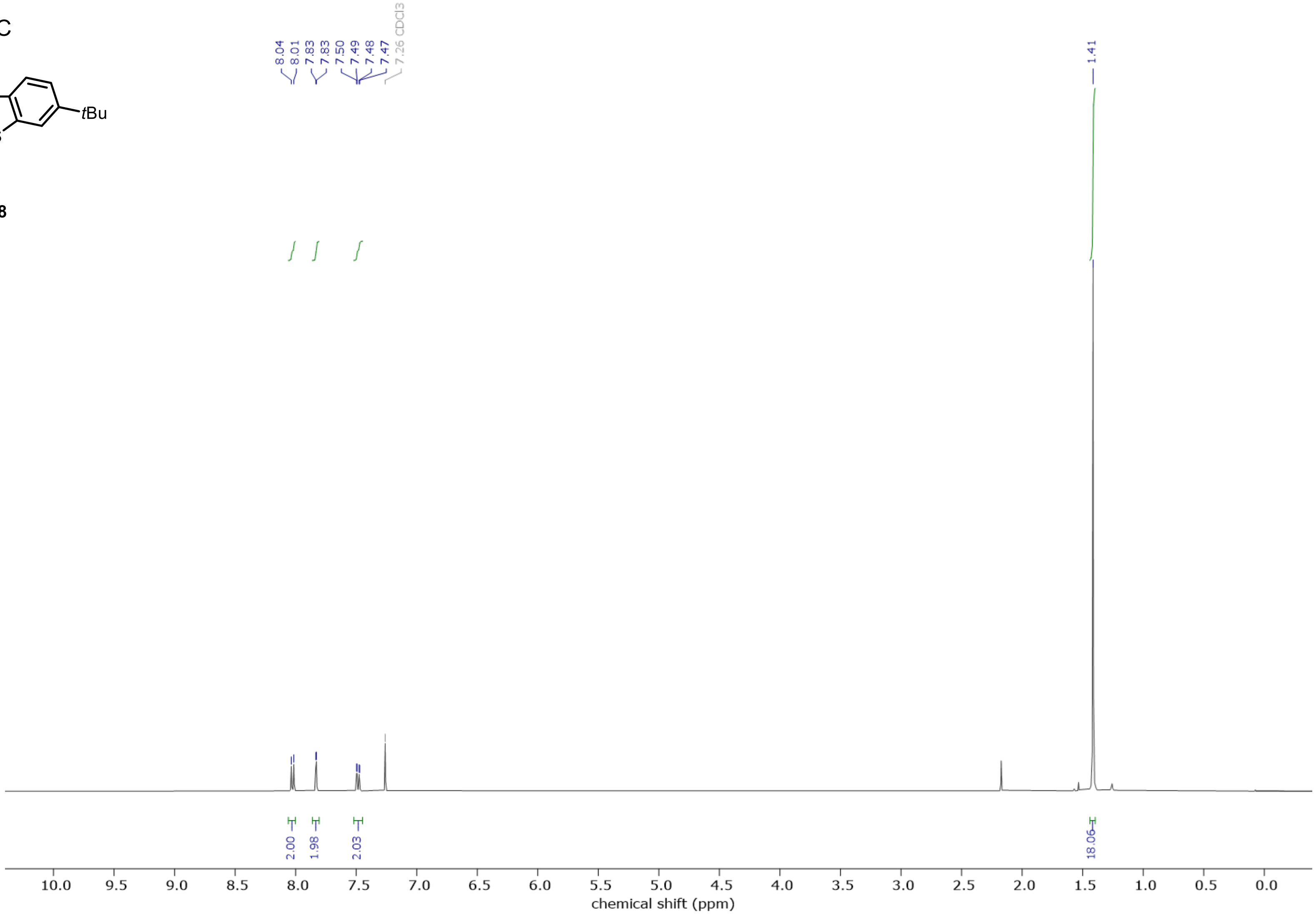

^{13}C NMR of 3,7-di-*tert*-butyldibenzo[*b*,*d*]thiophene (S8)

CDCl$_3$, 23 ºC

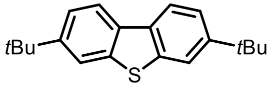

S8

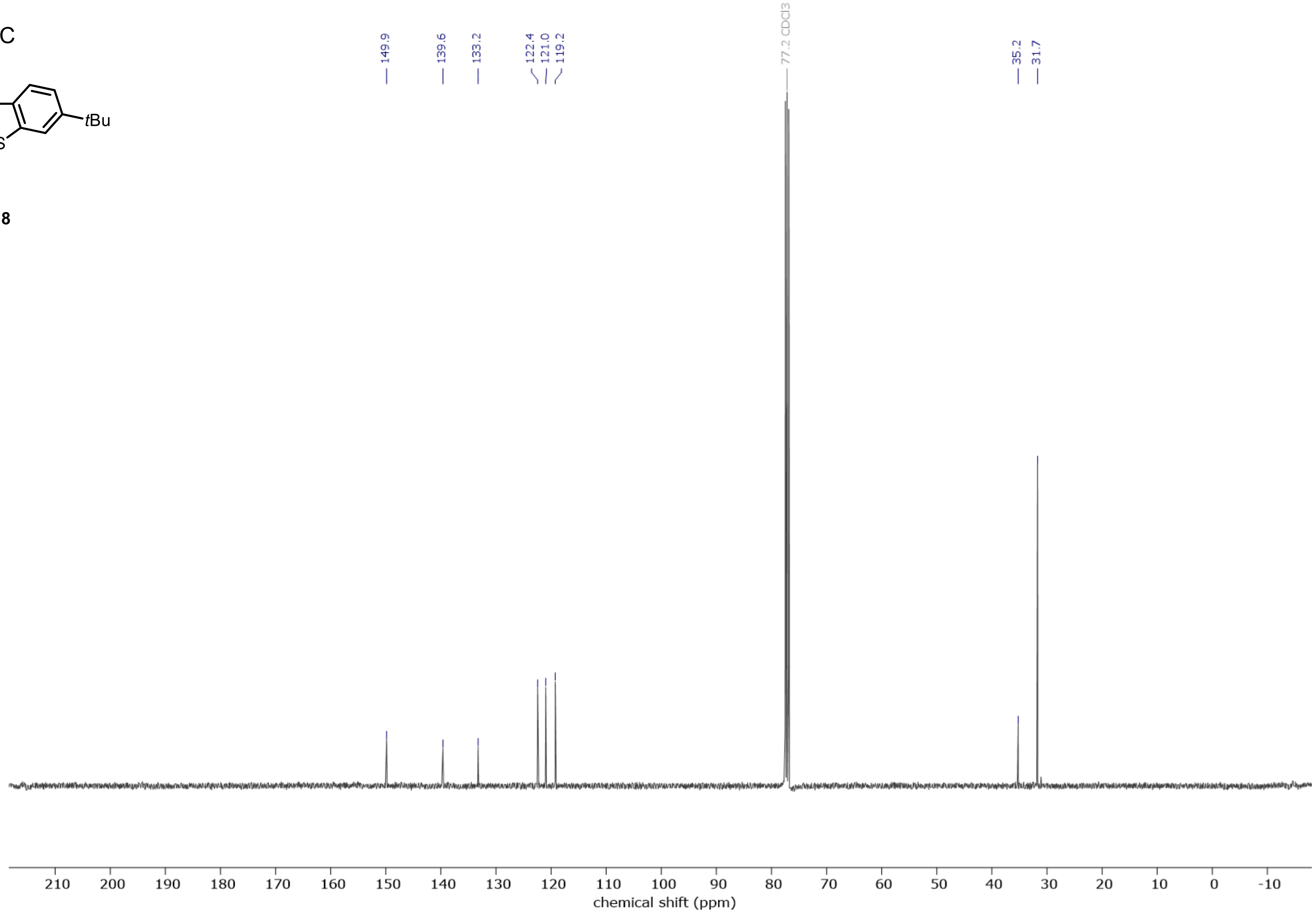

^{1}H NMR OF DIBENZO[B,D]SELENOPHENE 5-OXIDE (S9)

CDCl₃, 23 ºC

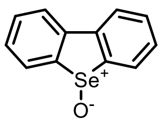

S9

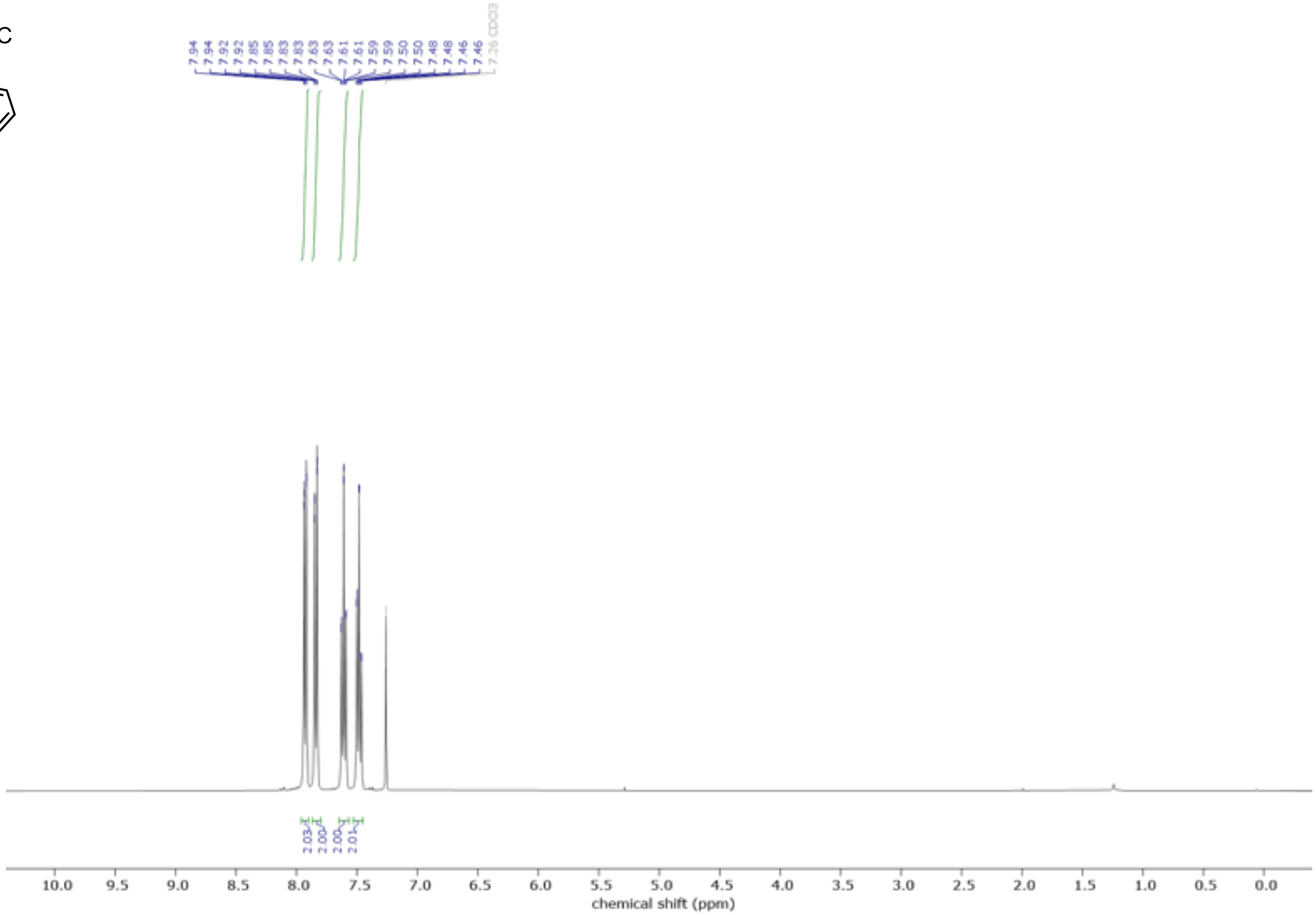

^{13}C NMR OF DIBENZO[B,D]SELENOPHENE 5-OXIDE (S9)

CDCl$_3$, 23 ºC

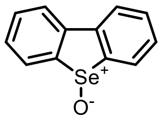

S9

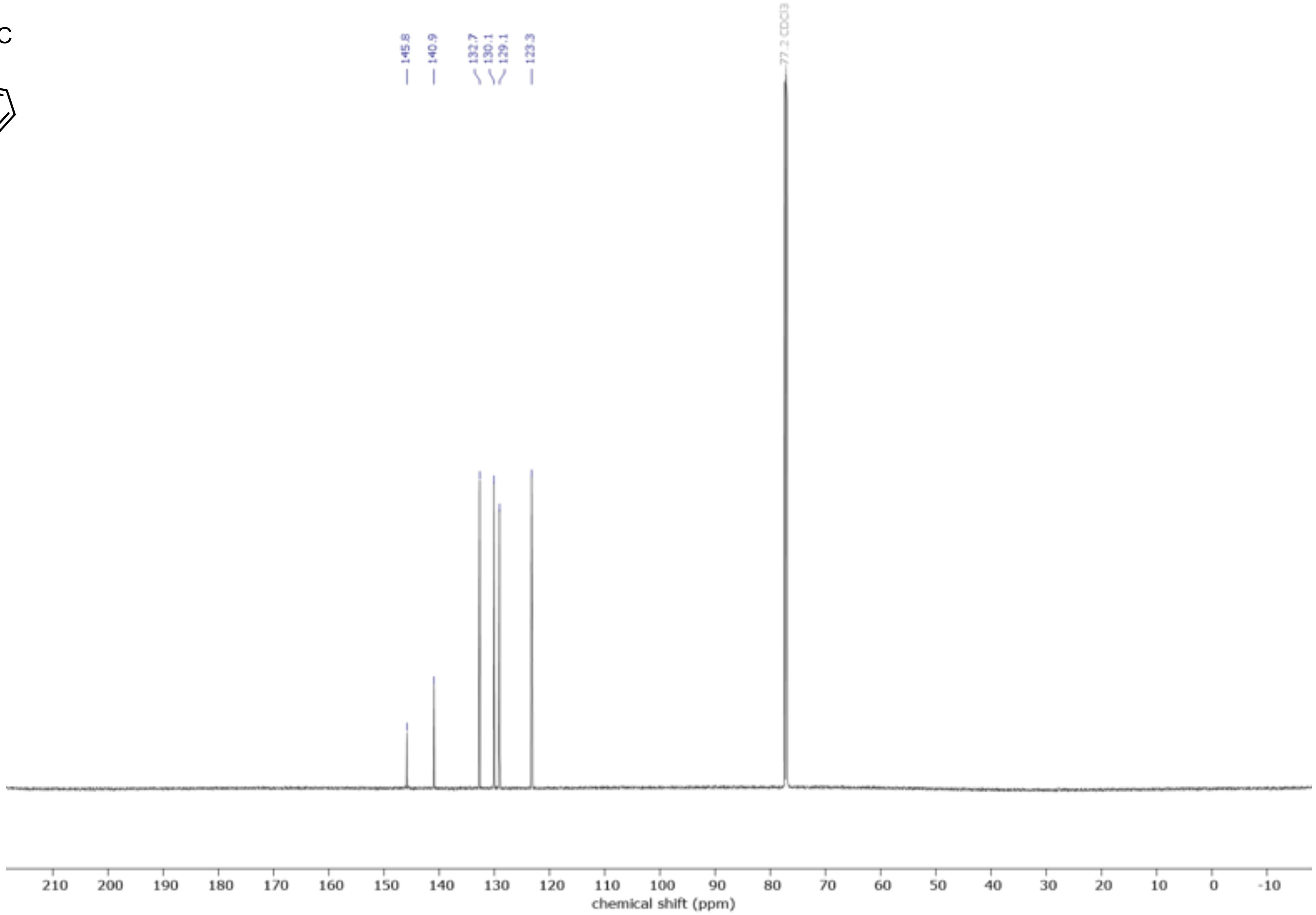

^{77}Se NMR of dibenzo[*b*,*d*]selenophene 5-oxide (S9)

CDCl3, 23 ºC

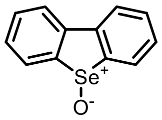

S9

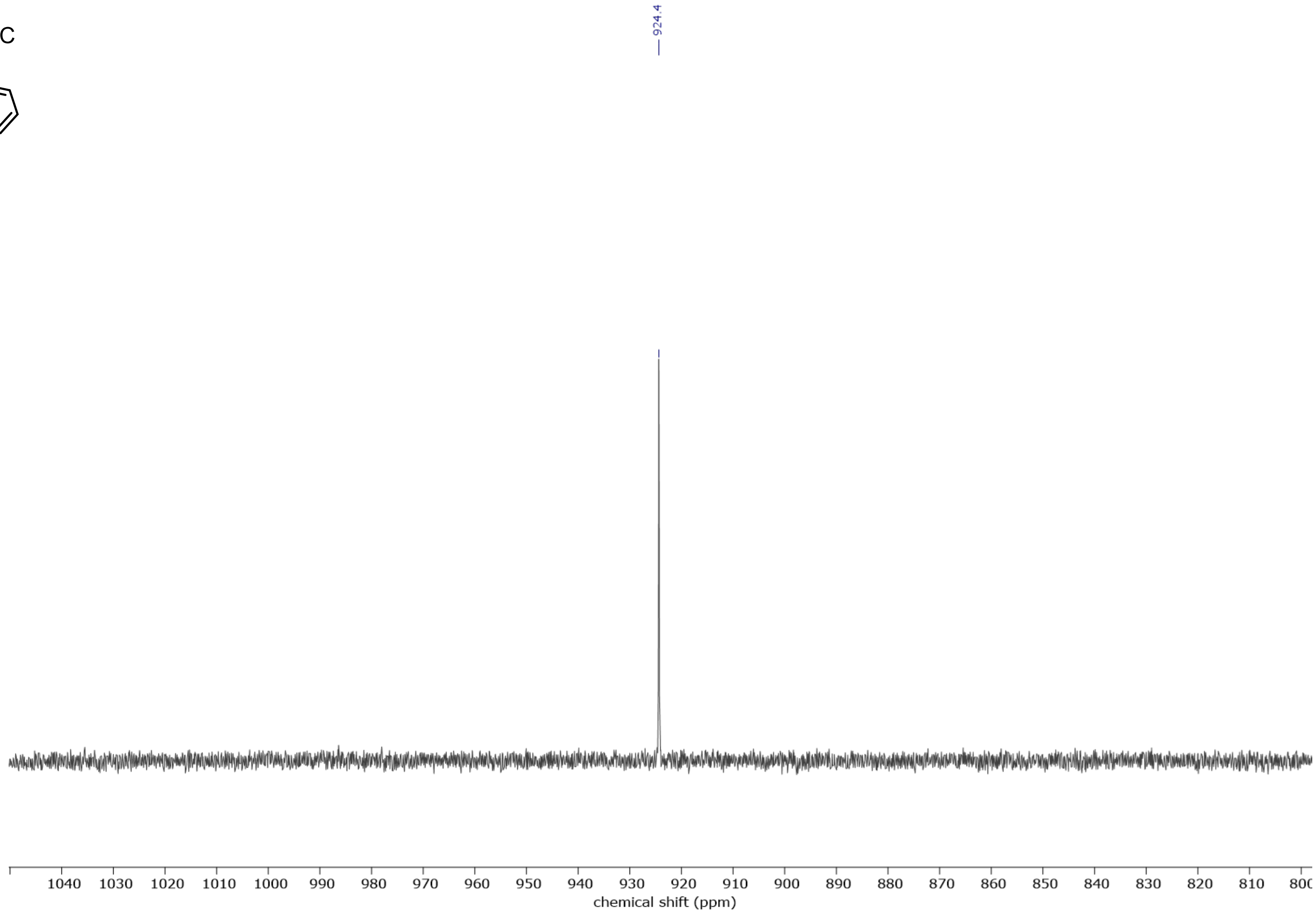

^{1}H NMR OF BPIN FENOFIBRATE DERIVATIVE (S11)

CDCl$_3$, 23 ºC

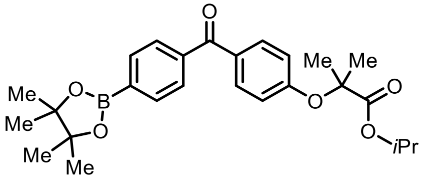

S11

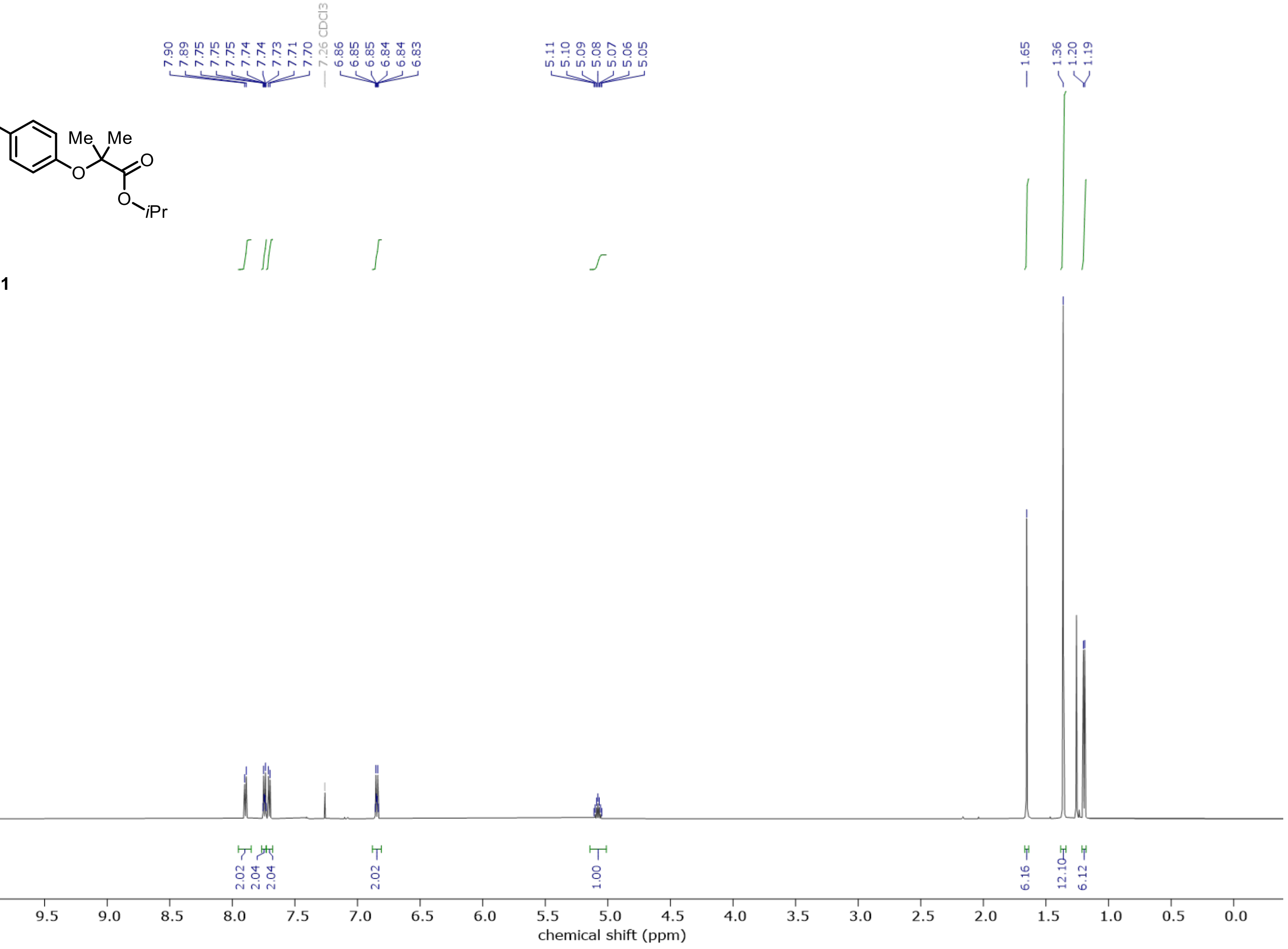

^{13}C NMR of Bpin fenofibrate derivative (S11)

CDCl3, 23 ºC

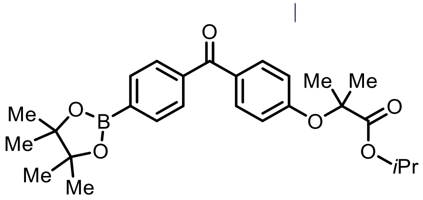

S11

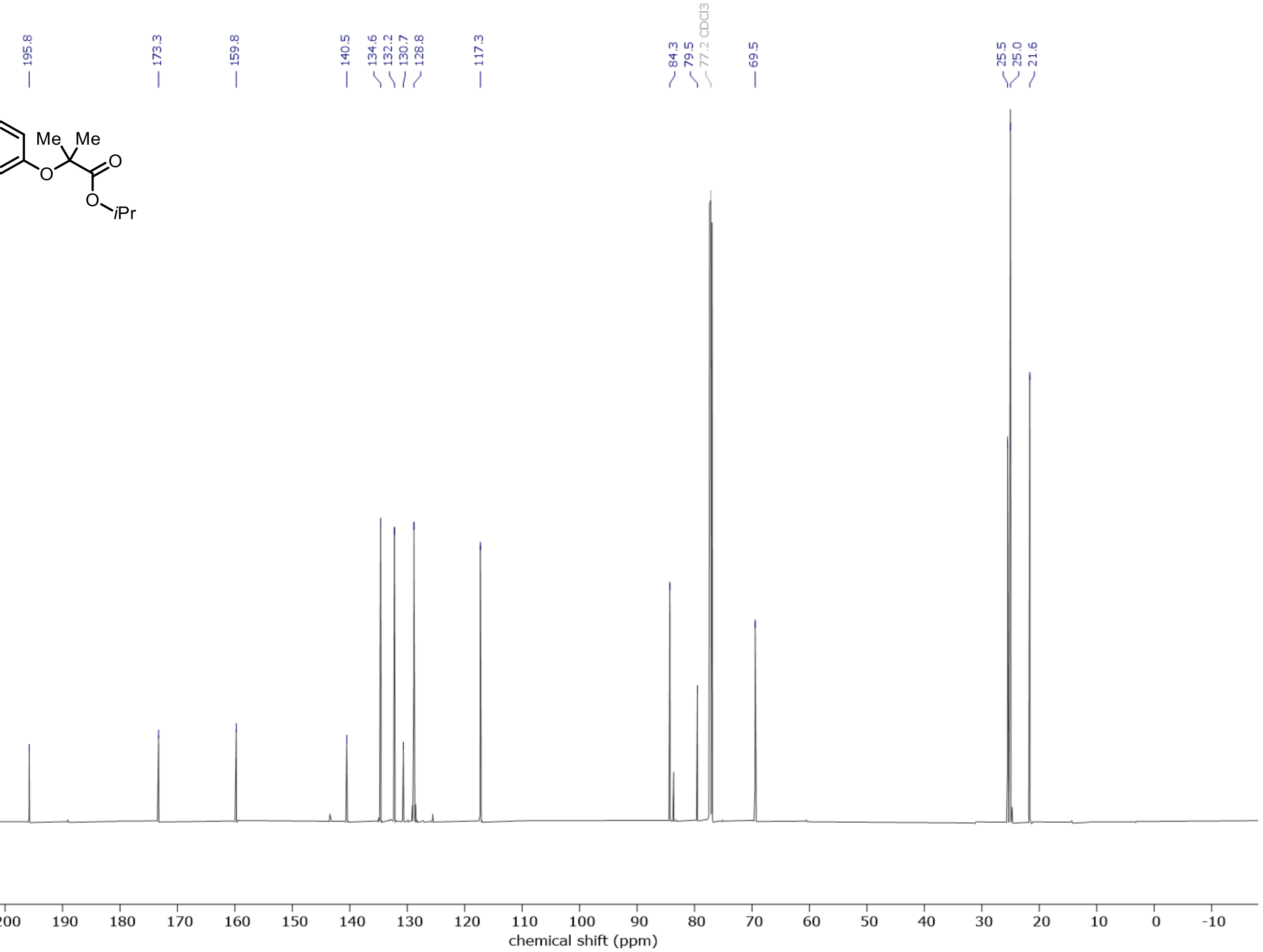

^{11}B NMR of Bpin fenofibrate derivative (S11)

CDCl3, 23 ºC

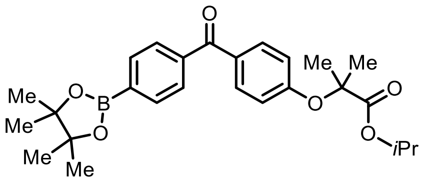

S11

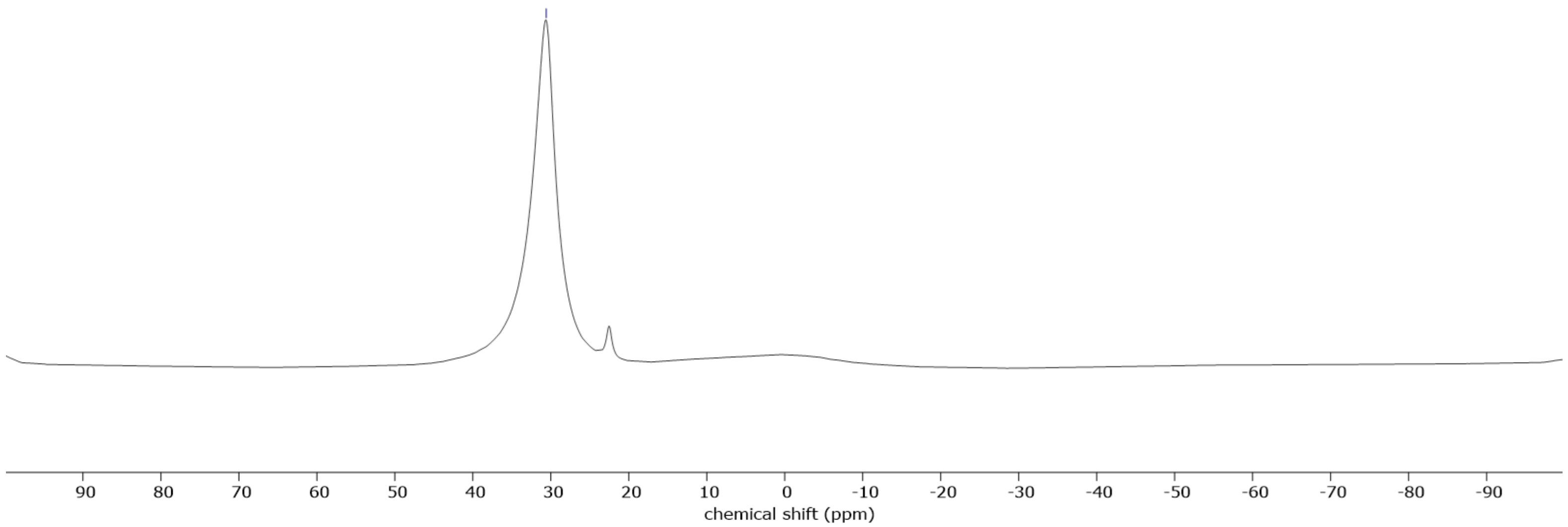

^{1}H NMR of Bpin tianeptine intermediate (S12)

CDCl₃, 23 ºC

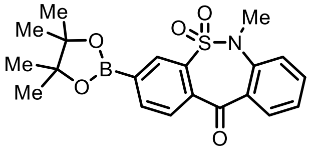

S12

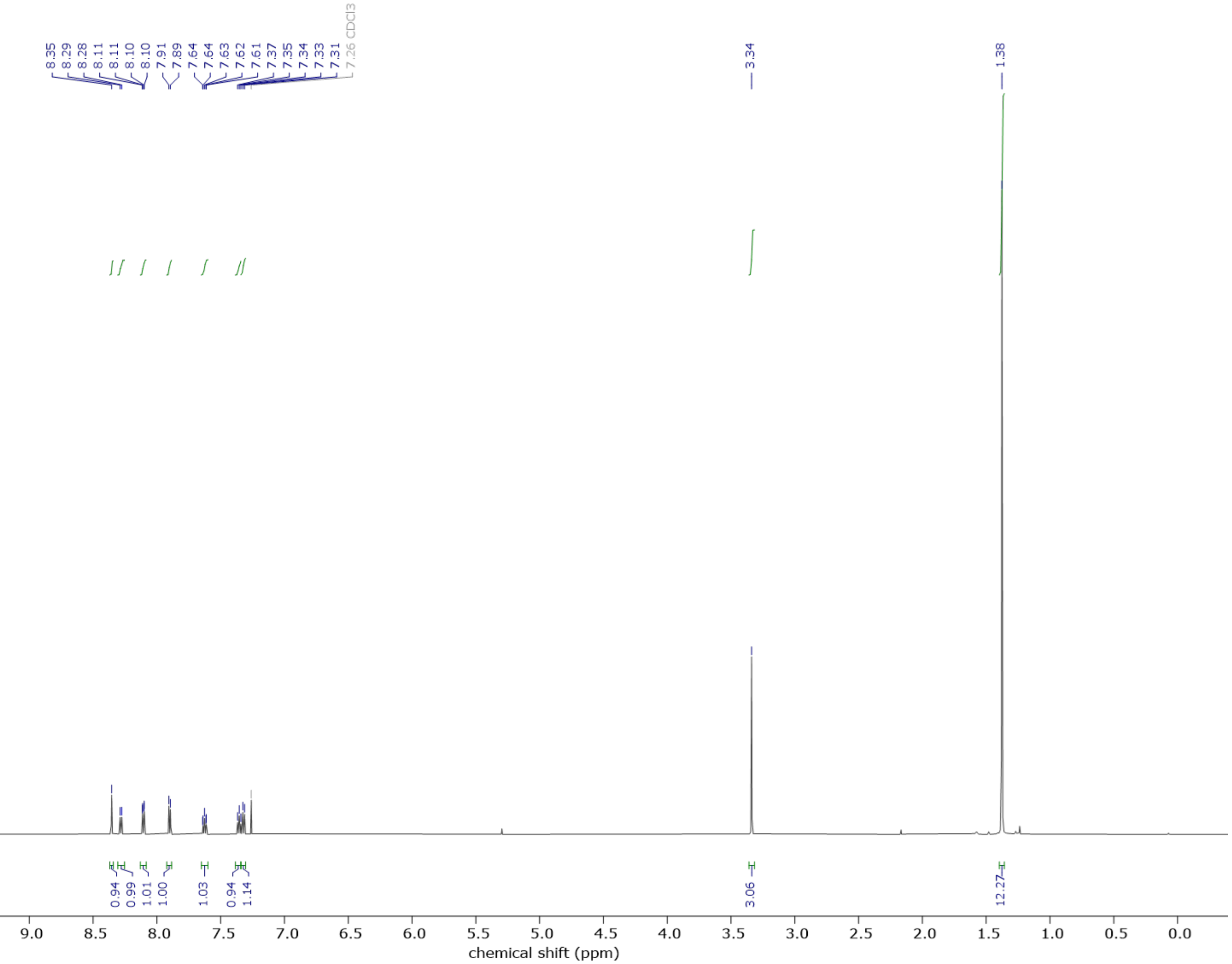

^{13}C NMR of Bpin tianeptine intermediate (S12)

CDCl$_3$, 23 ºC

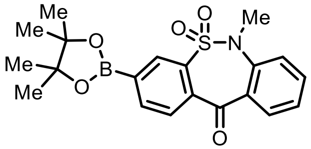

S12

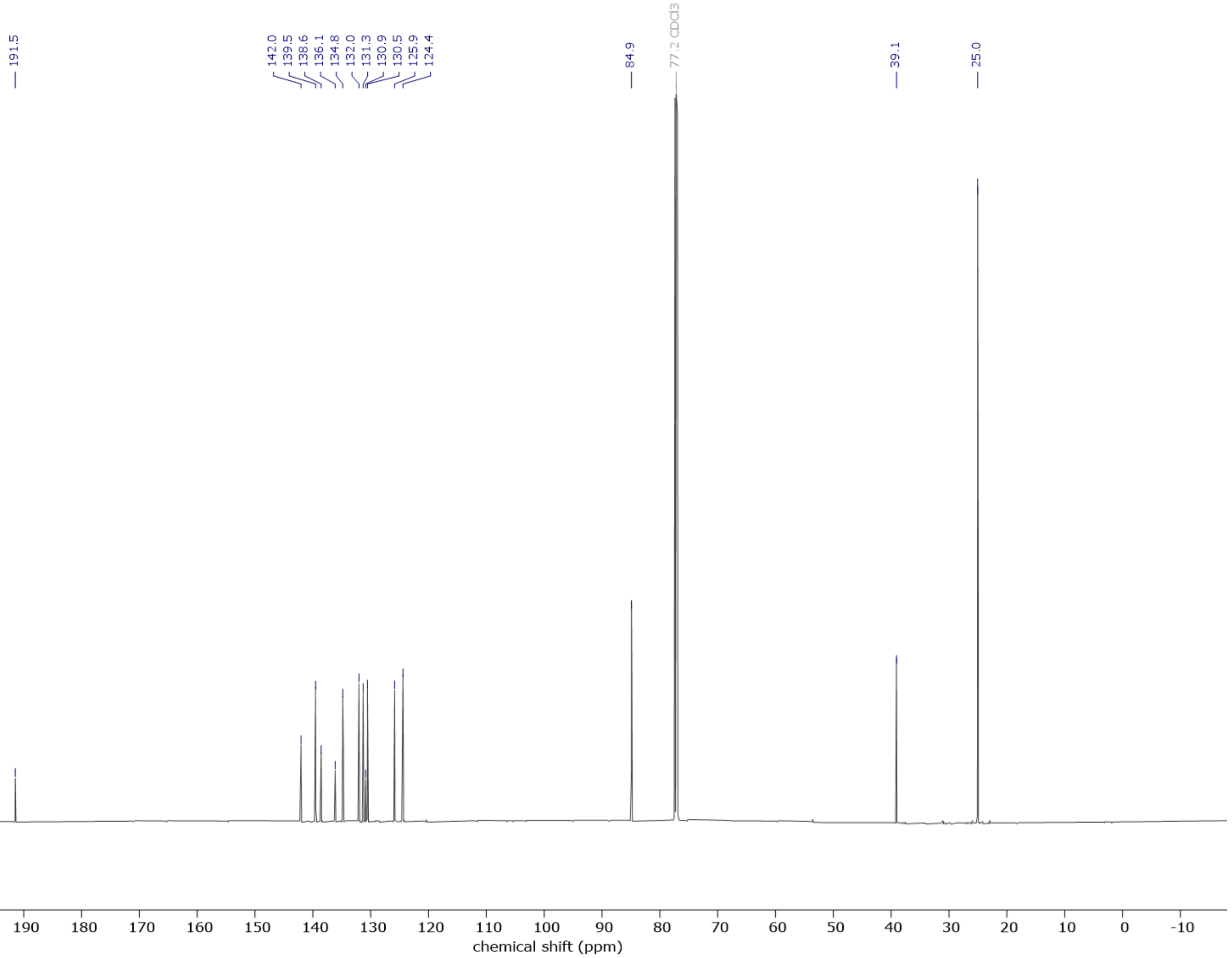

^{11}B NMR of Bpin tianeptine intermediate (S12)

CDCl3, 23 ºC

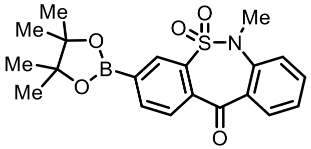

S12

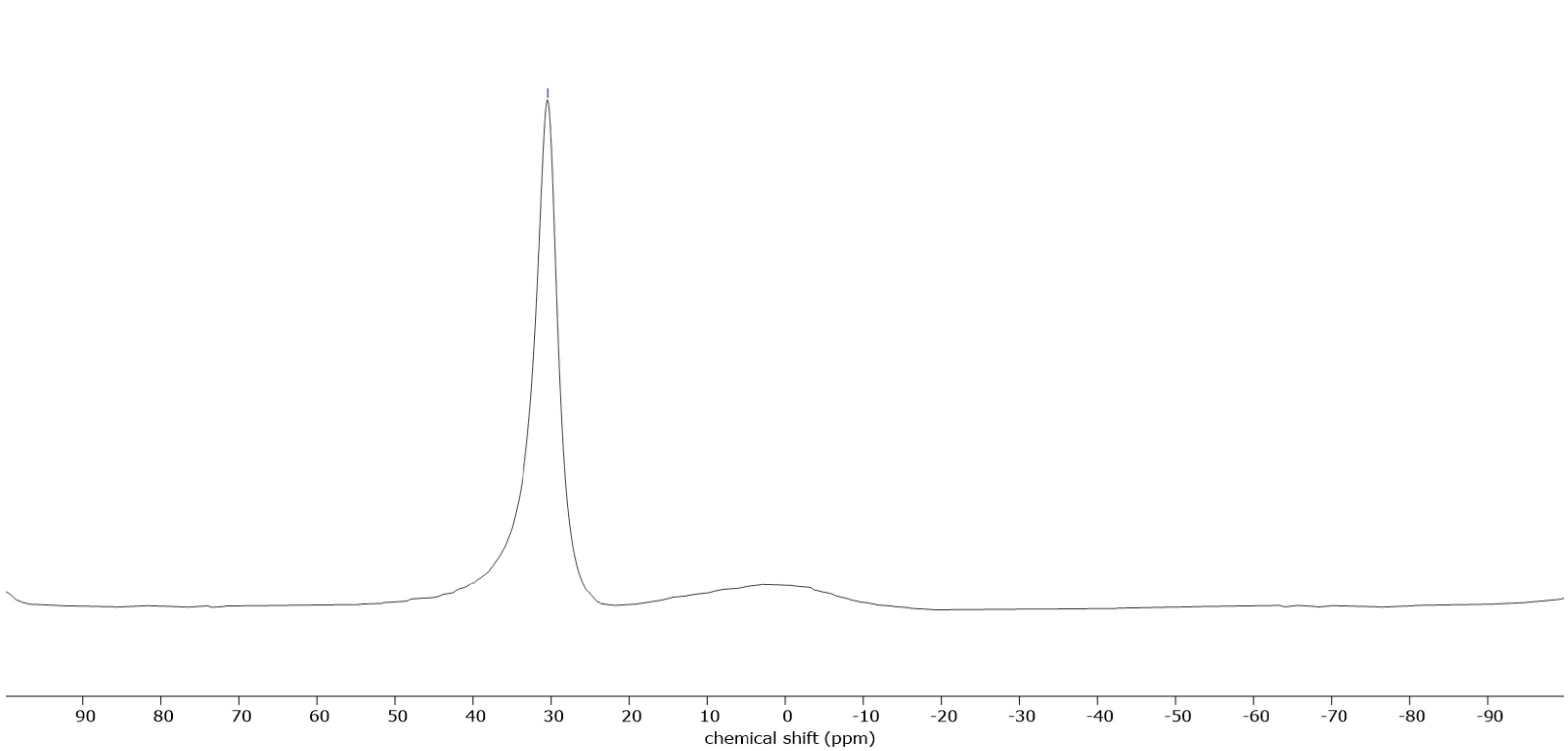

^{1}H NMR OF 5-(4-(*TERT*-BUTYL)PHENYL) -DIBENZOSELENOPHENIUM HEXAFLUOROANTIMONATE (S13)

DMSO-d6, 23 ºC

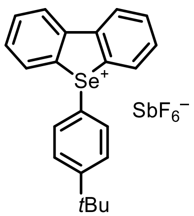

S13

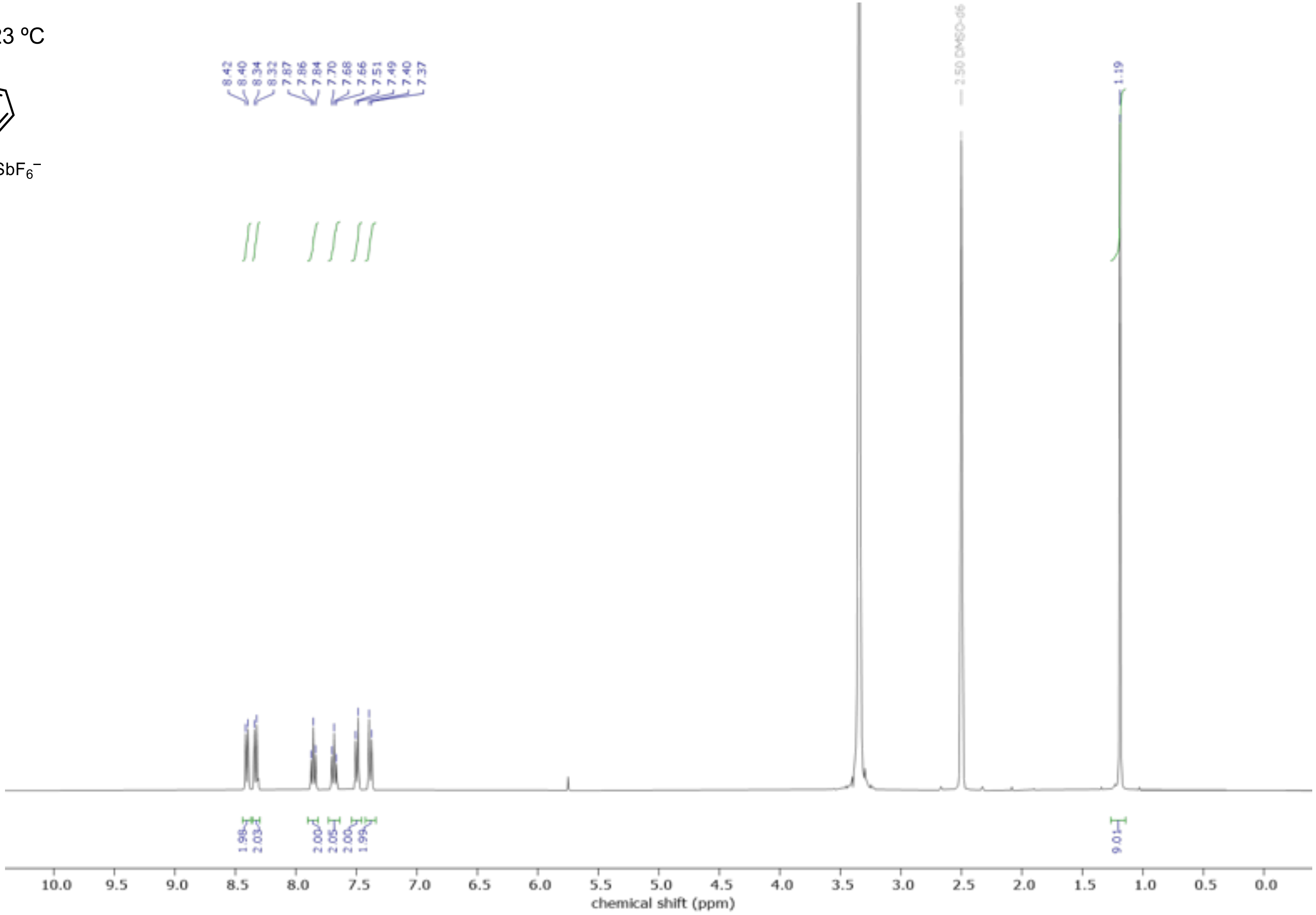

^{13}C NMR of 5-(4-(*tert*-butyl)phenyl) -dibenzoselenophenium hexafluoroantimonate (S13)

DMSO-d6, 23 ºC

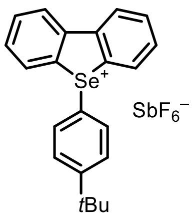

S13

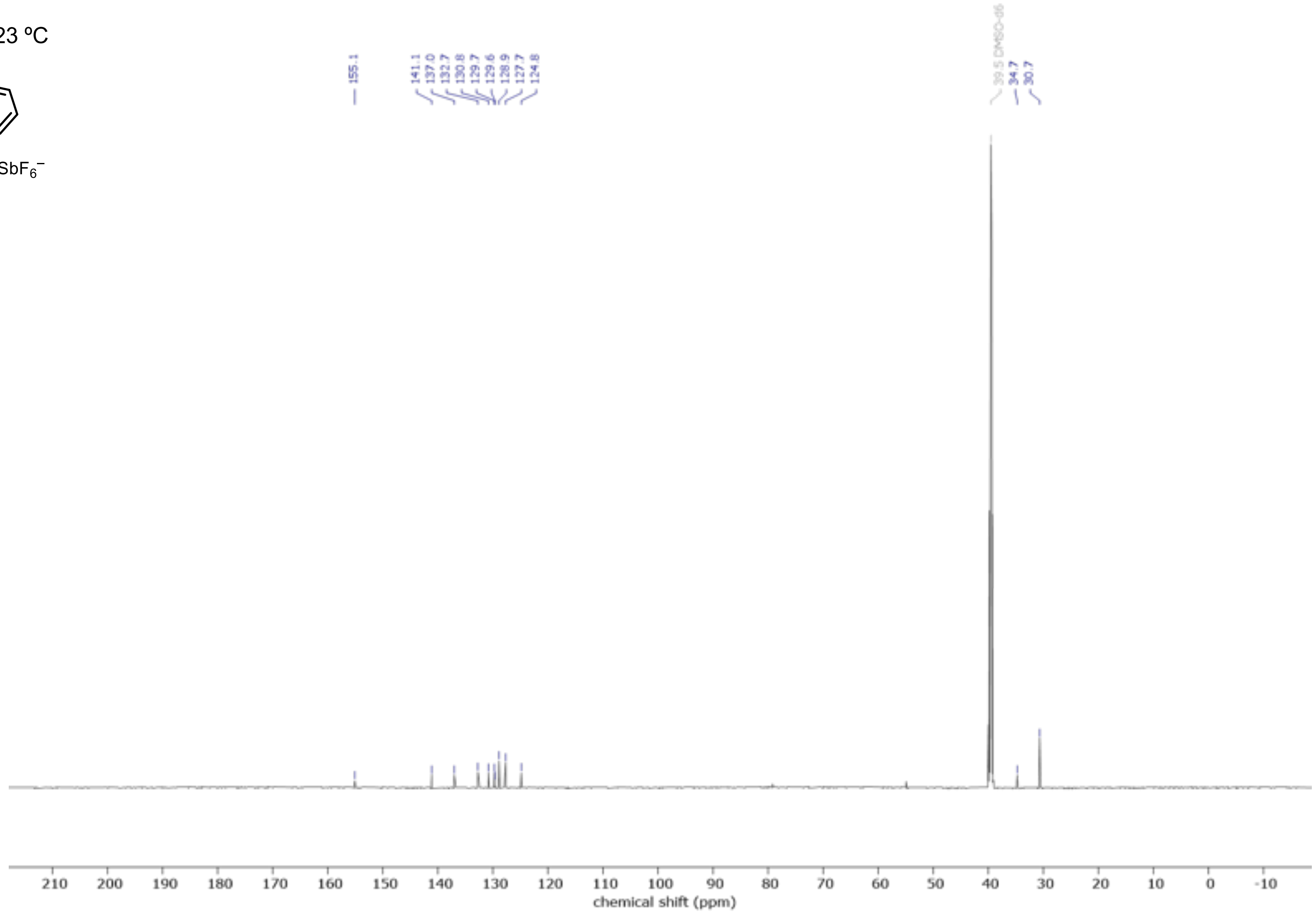

^{19}F NMR of 5-(4-(*tert*-butyl)phenyl) -dibenzoselenophenium hexafluoroantimonate (S13)

DMSO-d6, 23 ºC

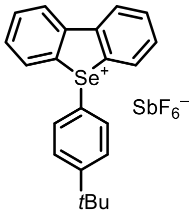

S13

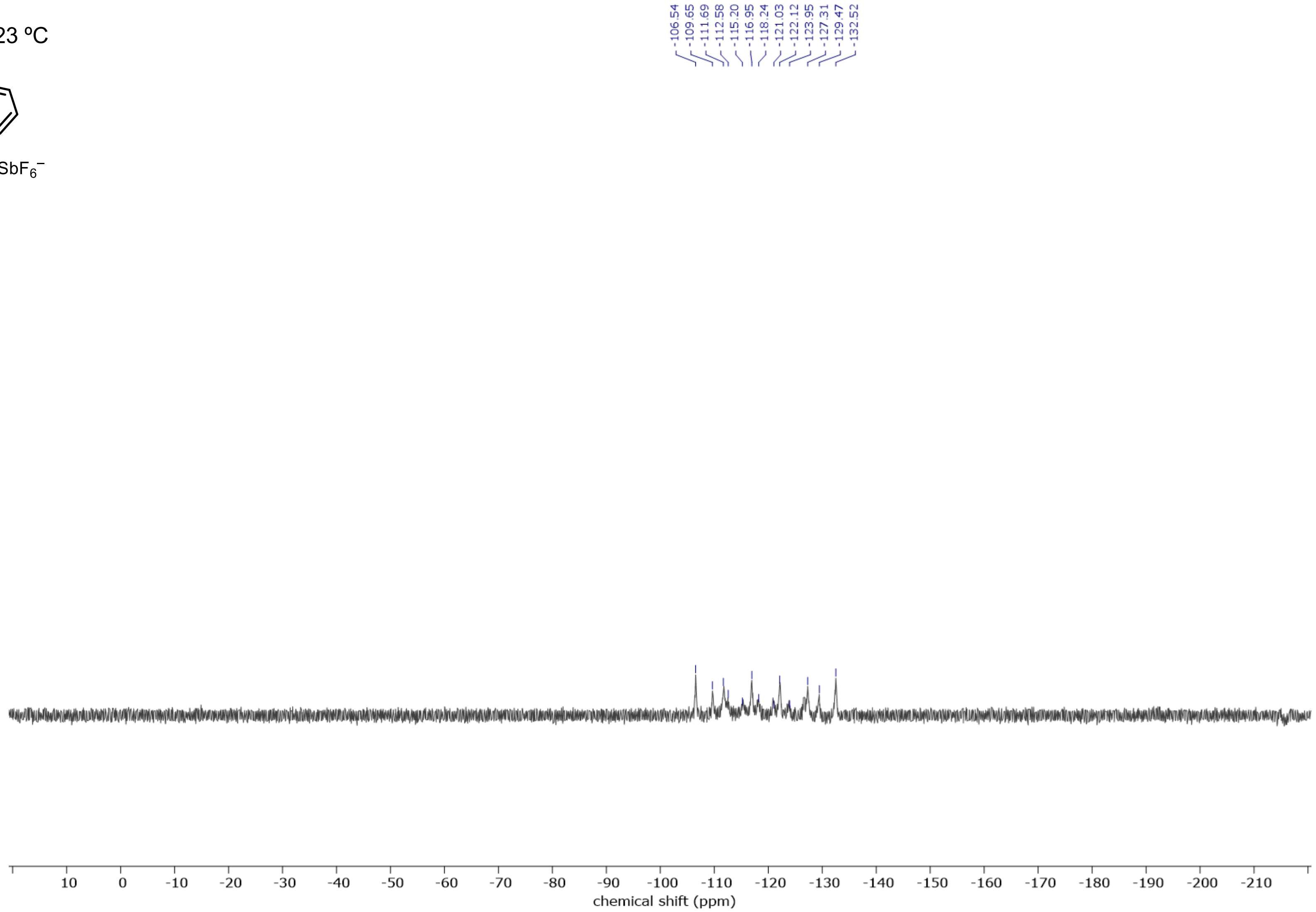

^{77}SE NMR OF 5-(4-(*TERT*-BUTYL)PHENYL) -DIBENZOSELENOPHENIUM HEXAFLUOROANTIMONATE (S13)

DMSO-d6, 23 ºC

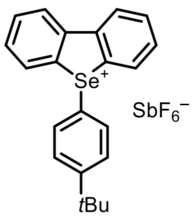

S13

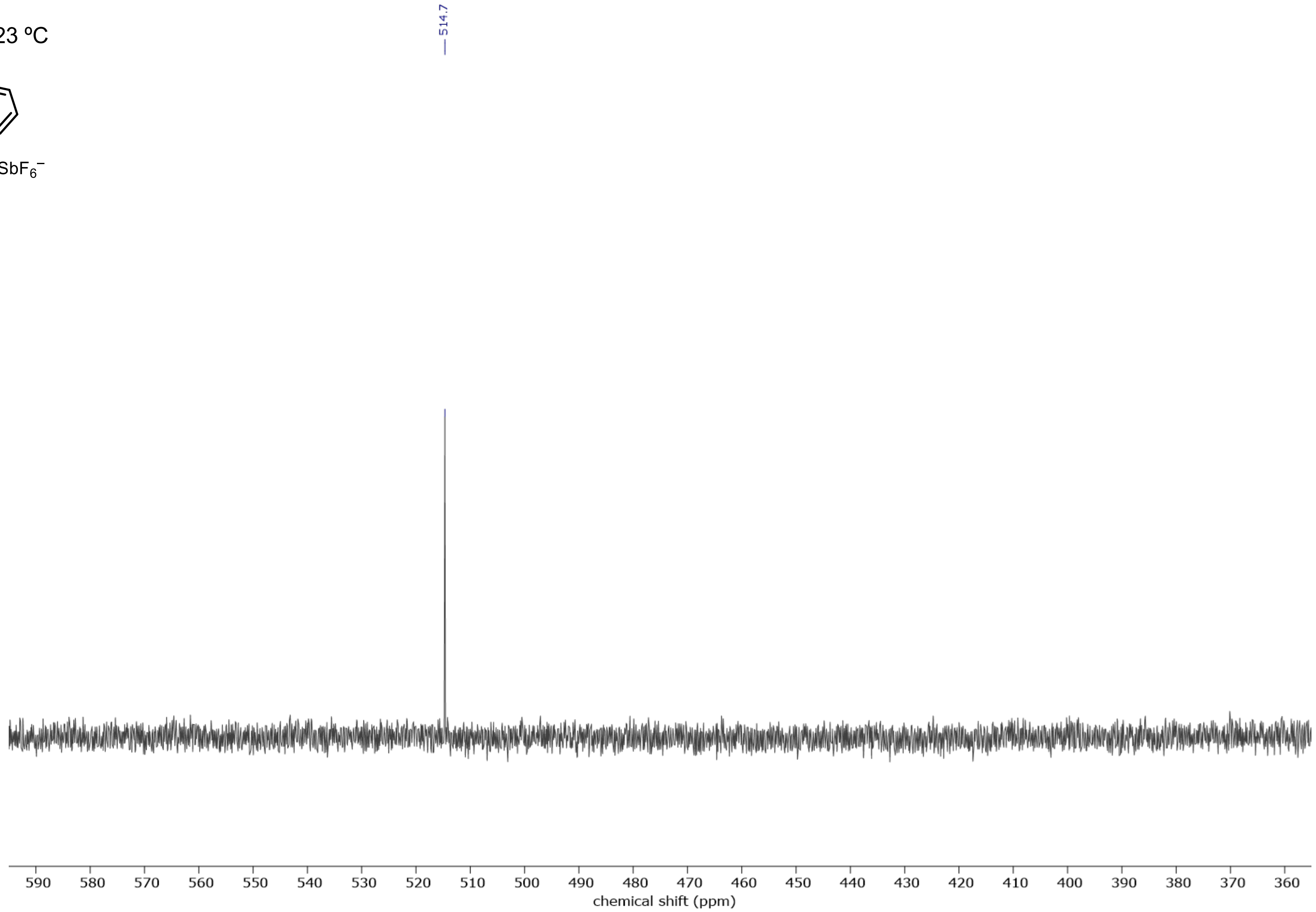

^{1}H NMR of 5-(4-methoxyphenyl)-5*H*-dibenzo[*b*,*d*]selenophen-5-ium hexafluoroantimonate (S14)

DMSO-d_6, 23 ºC

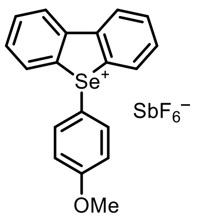

S14

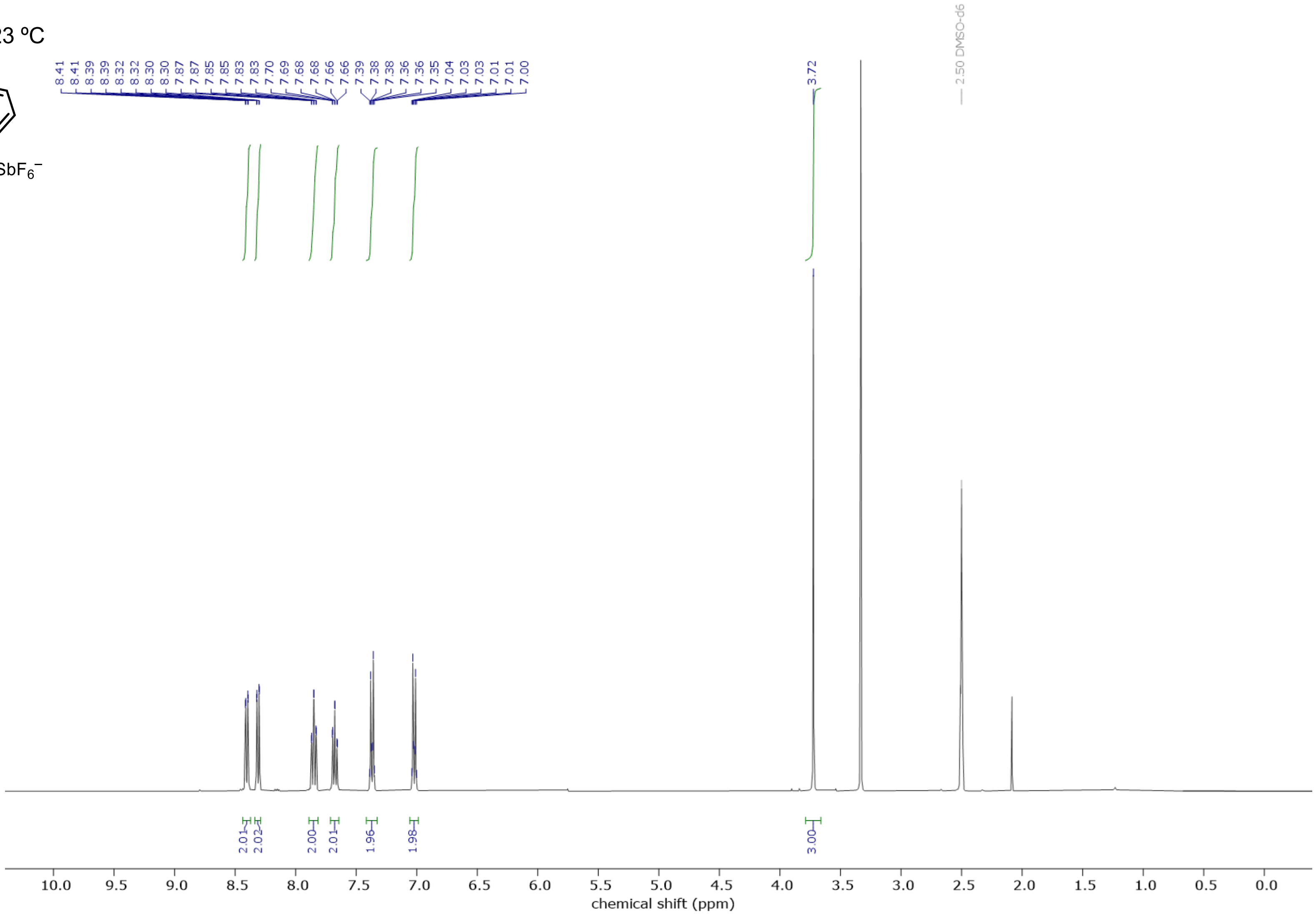

^{13}C NMR of 5-(4-methoxyphenyl)-5*H*-dibenzo[*b*,*d*]selenophen-5-ium hexafluoroantimonate (S14)

DMSO-d6, 23 ºC

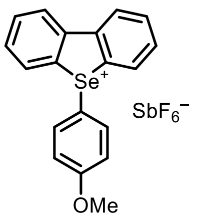

S14

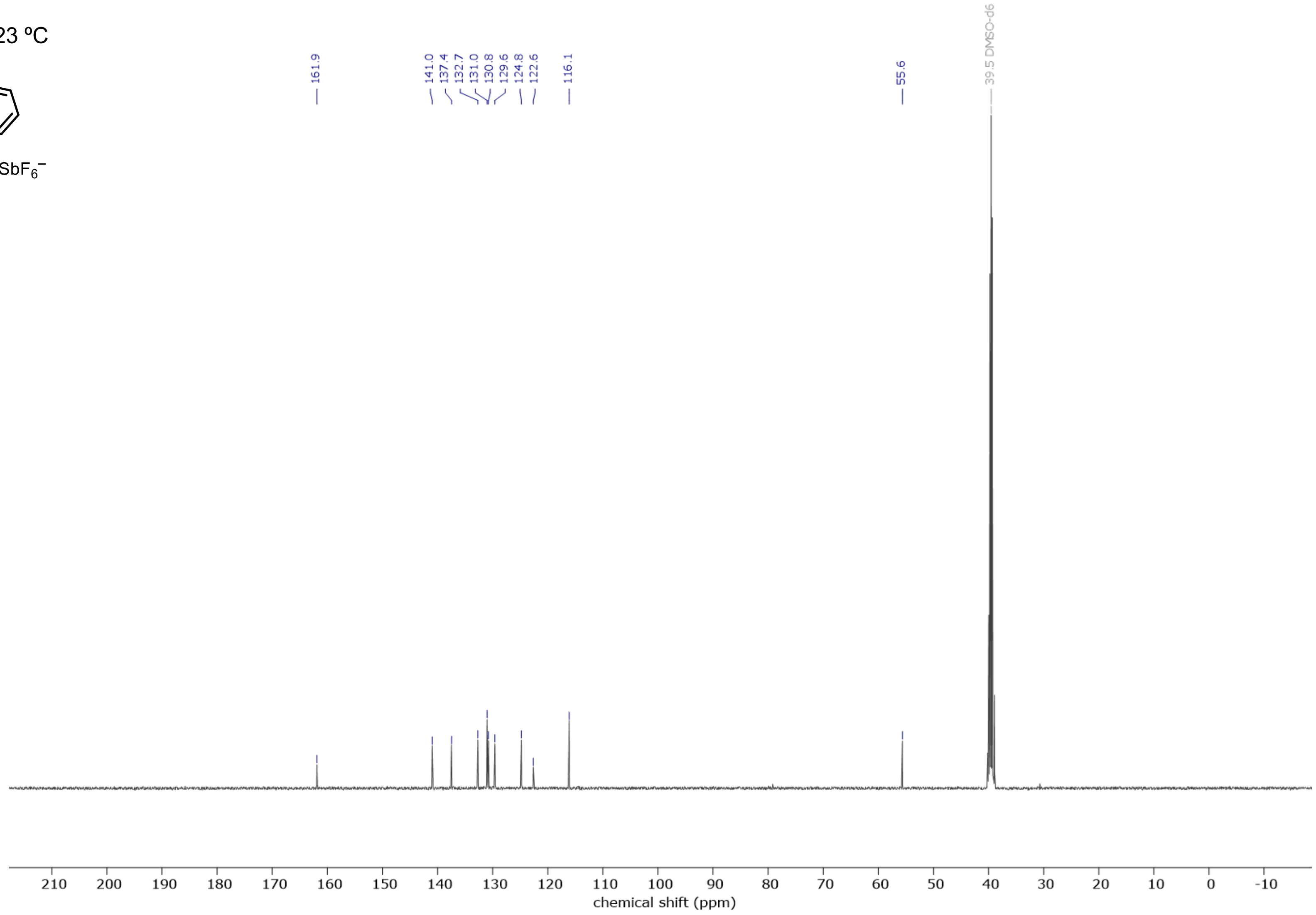

^{19}F NMR OF 5-(4-METHOXYPHENYL)-5*H*-DIBENZO[*B*,*D*]SELENOPHEN-5-IUM HEXAFLUOROANTIMONATE (S14)

DMSO-d6, 23 ºC

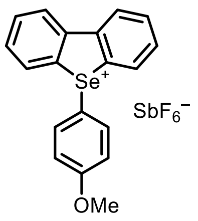

S14

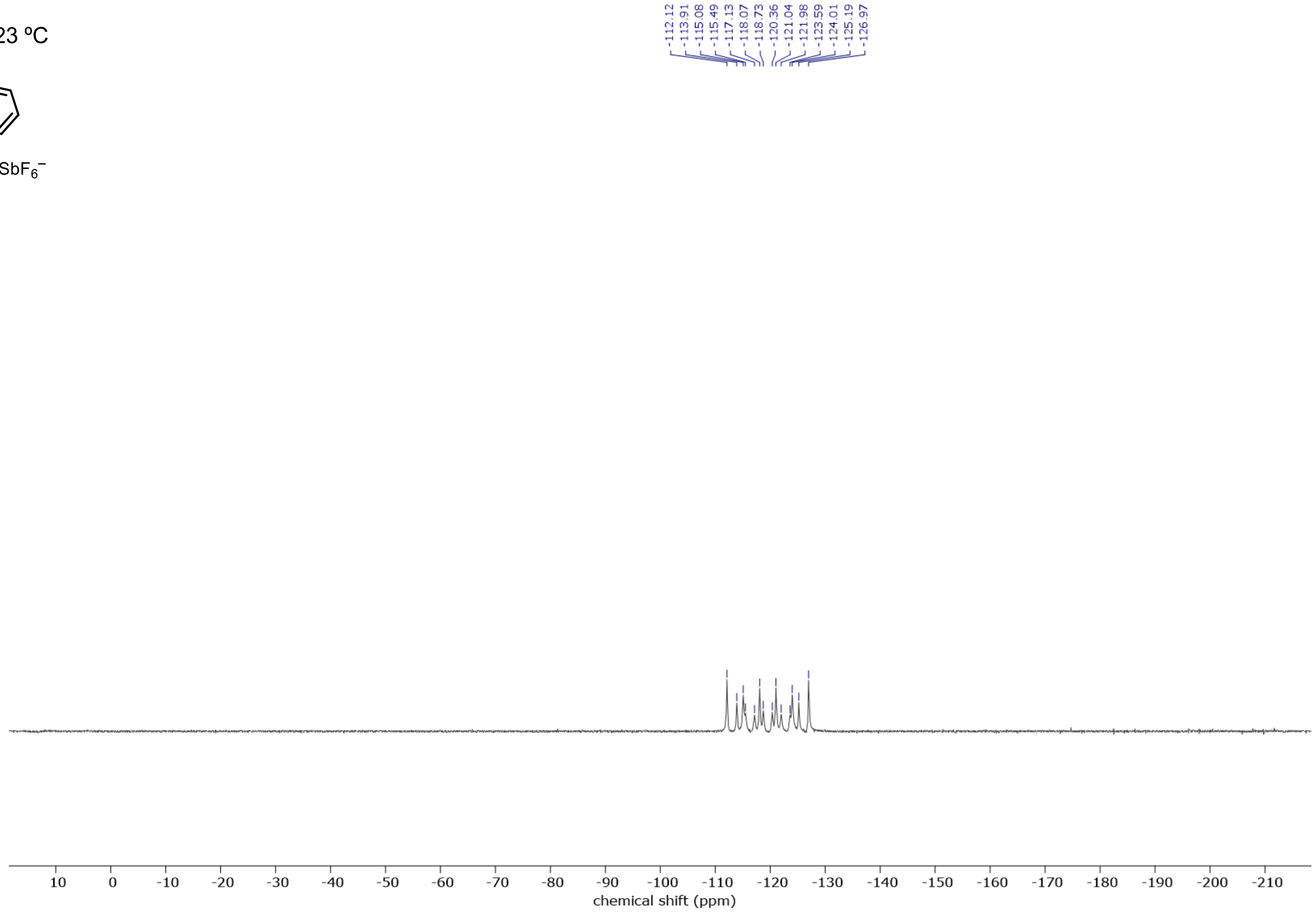

^{77}Se NMR of 5-(4-methoxyphenyl)-5*H*-dibenzo[*b*,*d*]selenophen-5-ium hexafluoroantimonate (S14)

DMSO-d6, 23 ºC

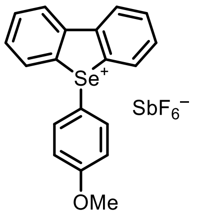

S14

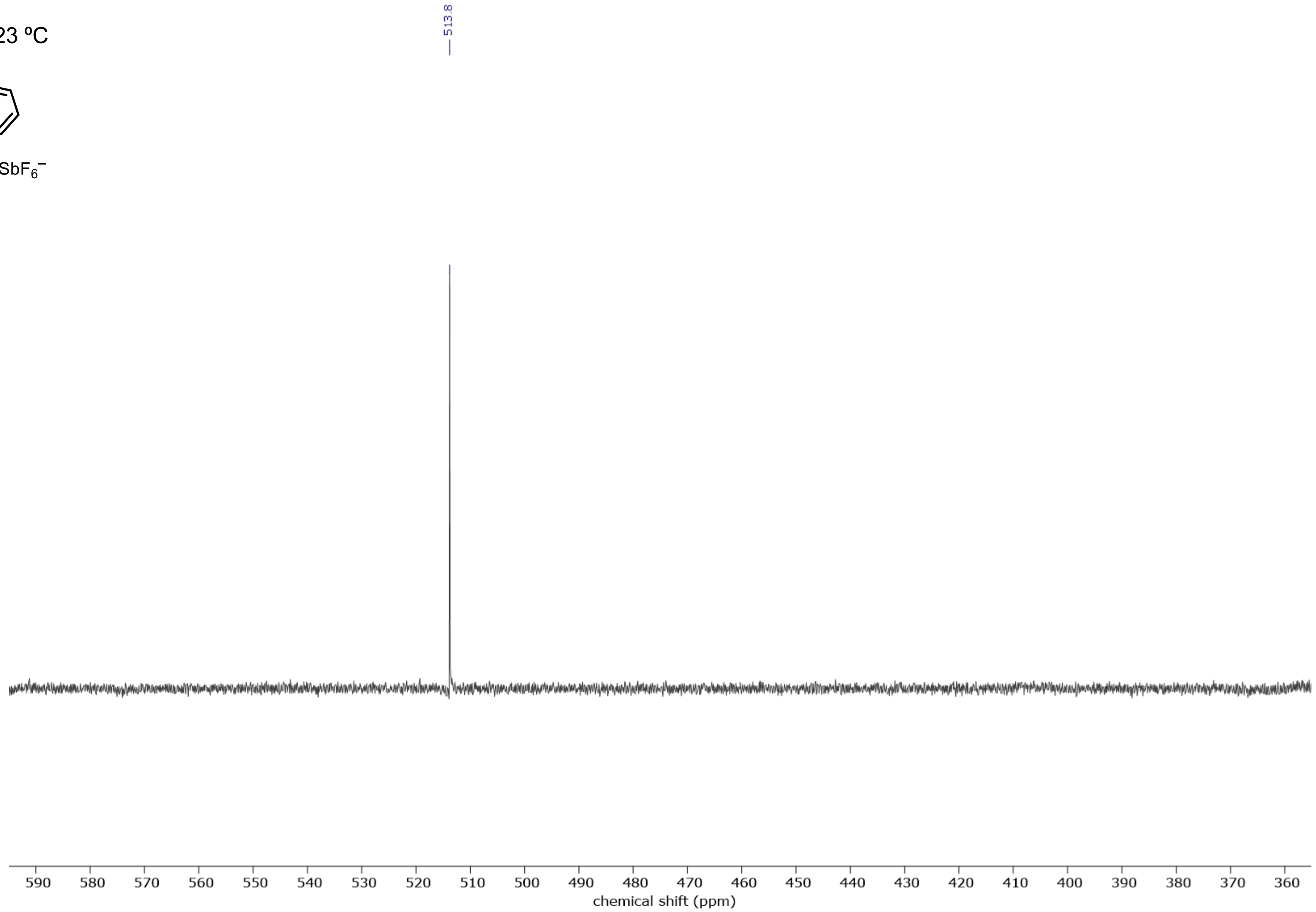

^{1}H NMR of 5-(4-(trifluoromethyl)phenyl)-5*H*-dibenzo[*b*,*d*]selenophen-5-ium hexafluoroantimonate (S15)

DMSO-d6, 23 ºC

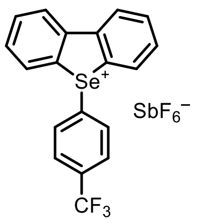

S15

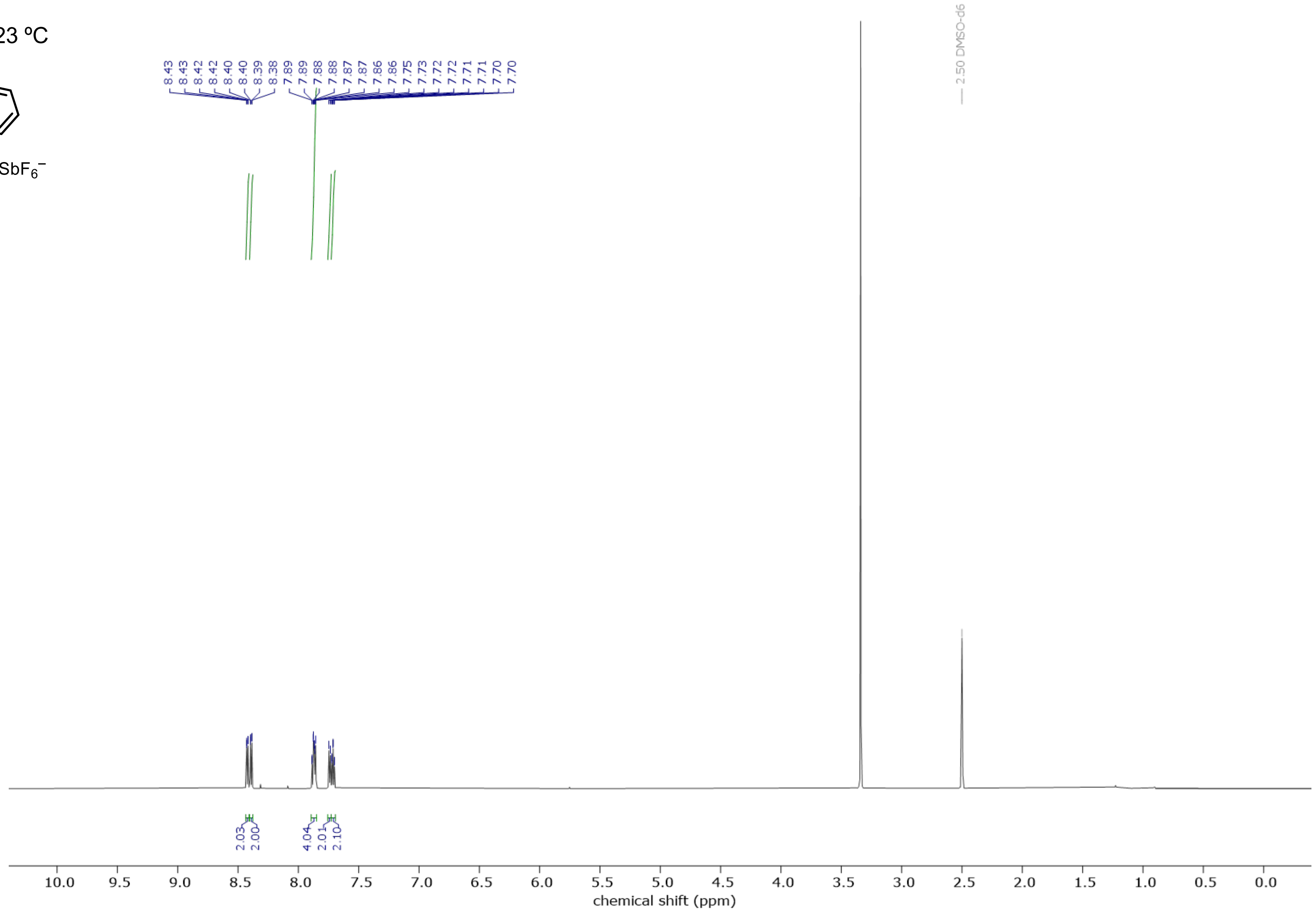

^{13}C NMR of 5-(4-(trifluoromethyl)phenyl)-5*H*-dibenzo[*b*,*d*]selenophen-5-ium hexafluoroantimonate (S15)

DMSO-d6, 23 ºC

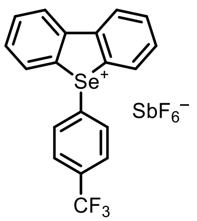

S15

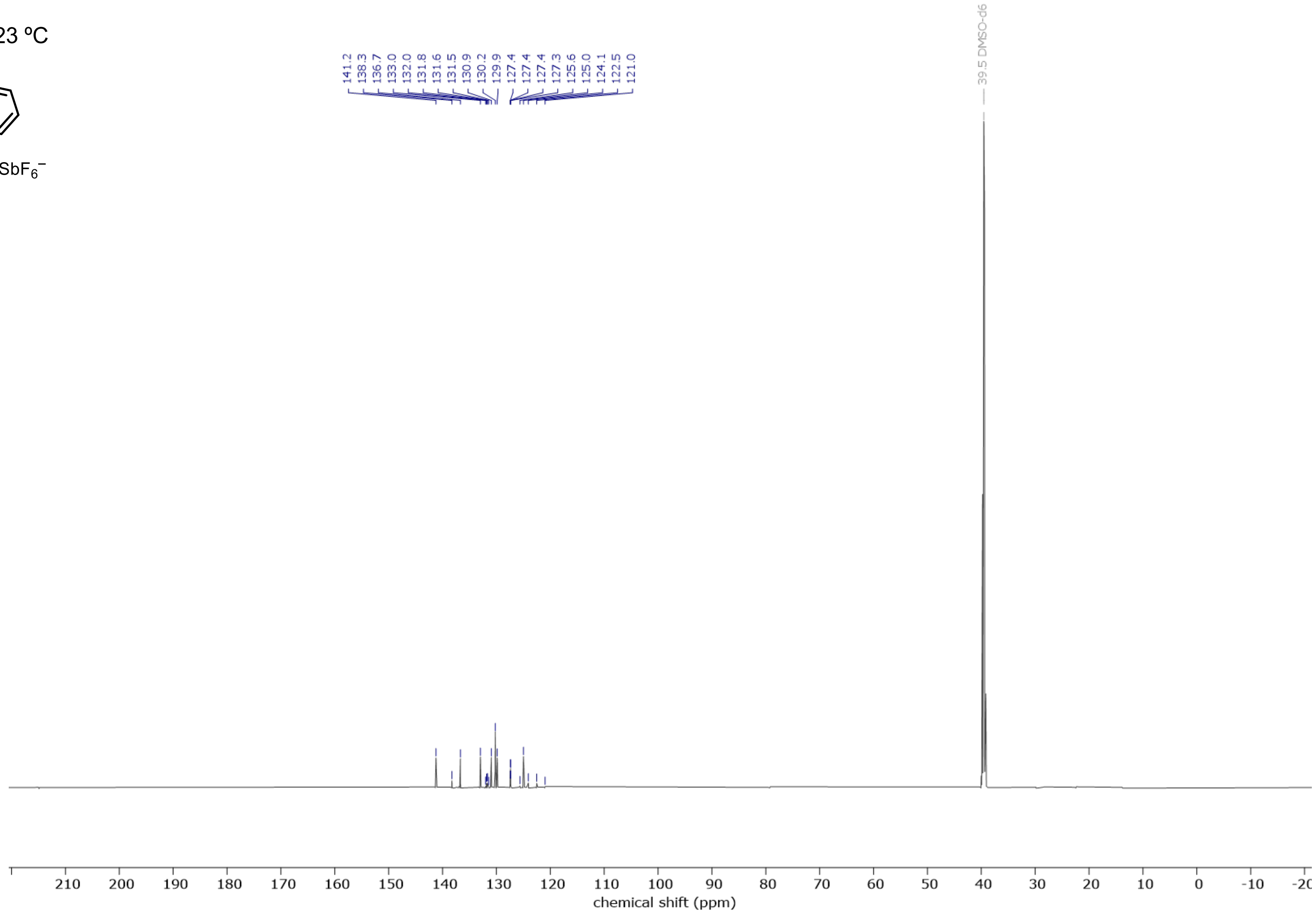

^{19}F NMR of 5-(4-(trifluoromethyl)phenyl)-5*H*-dibenzo[*b*,*d*]selenophen-5-ium hexafluoroantimonate (S15)

DMSO-d6, 23 ºC

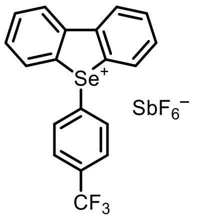

S15

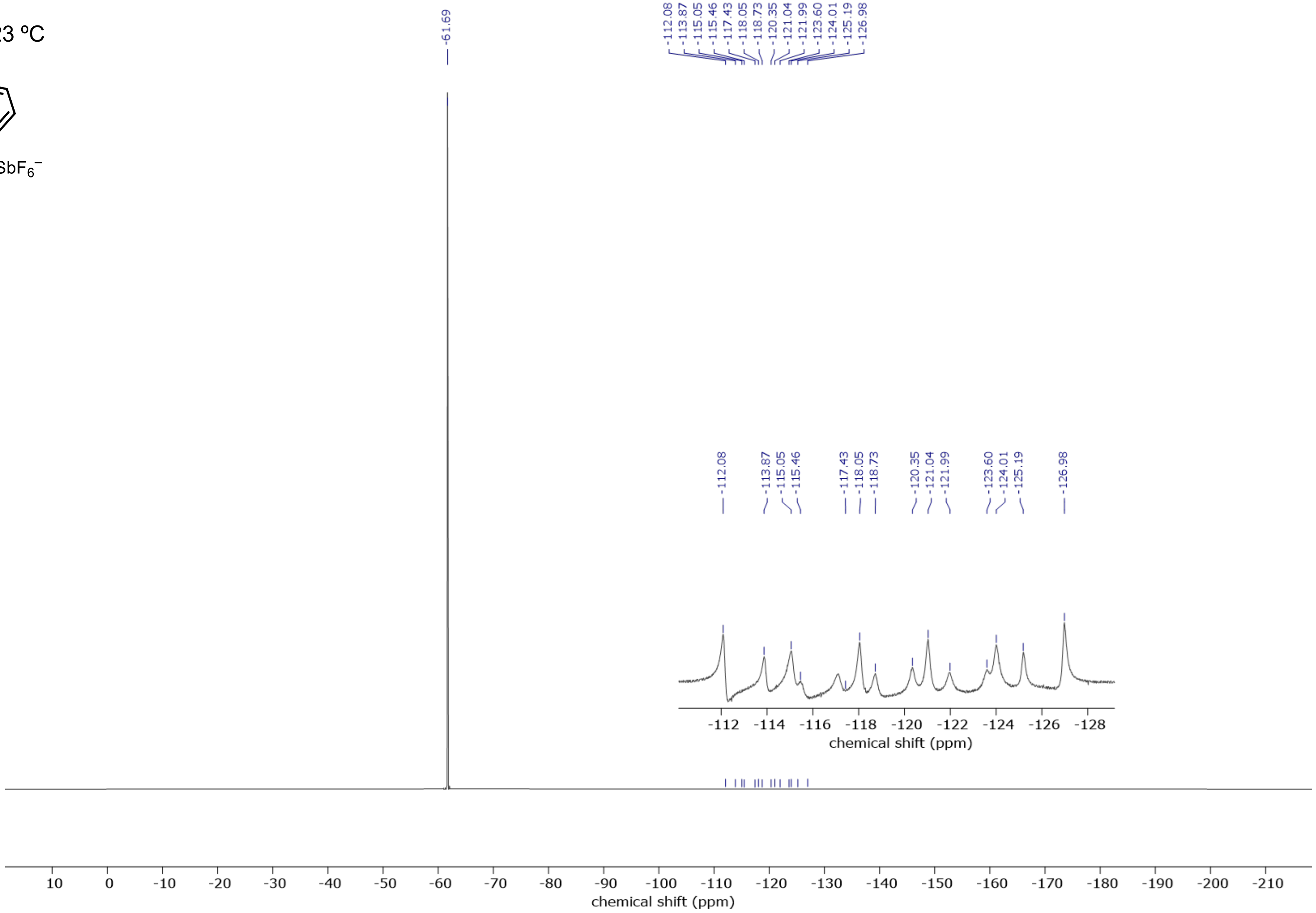

^{77}Se NMR of 5-(4-(trifluoromethyl)phenyl)-5*H*-dibenzo[*b*,*d*]selenophen-5-ium hexafluoroantimonate (S15)

DMSO-d6, 23 ºC

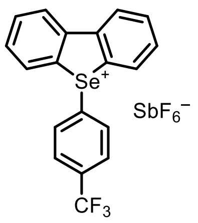

S15

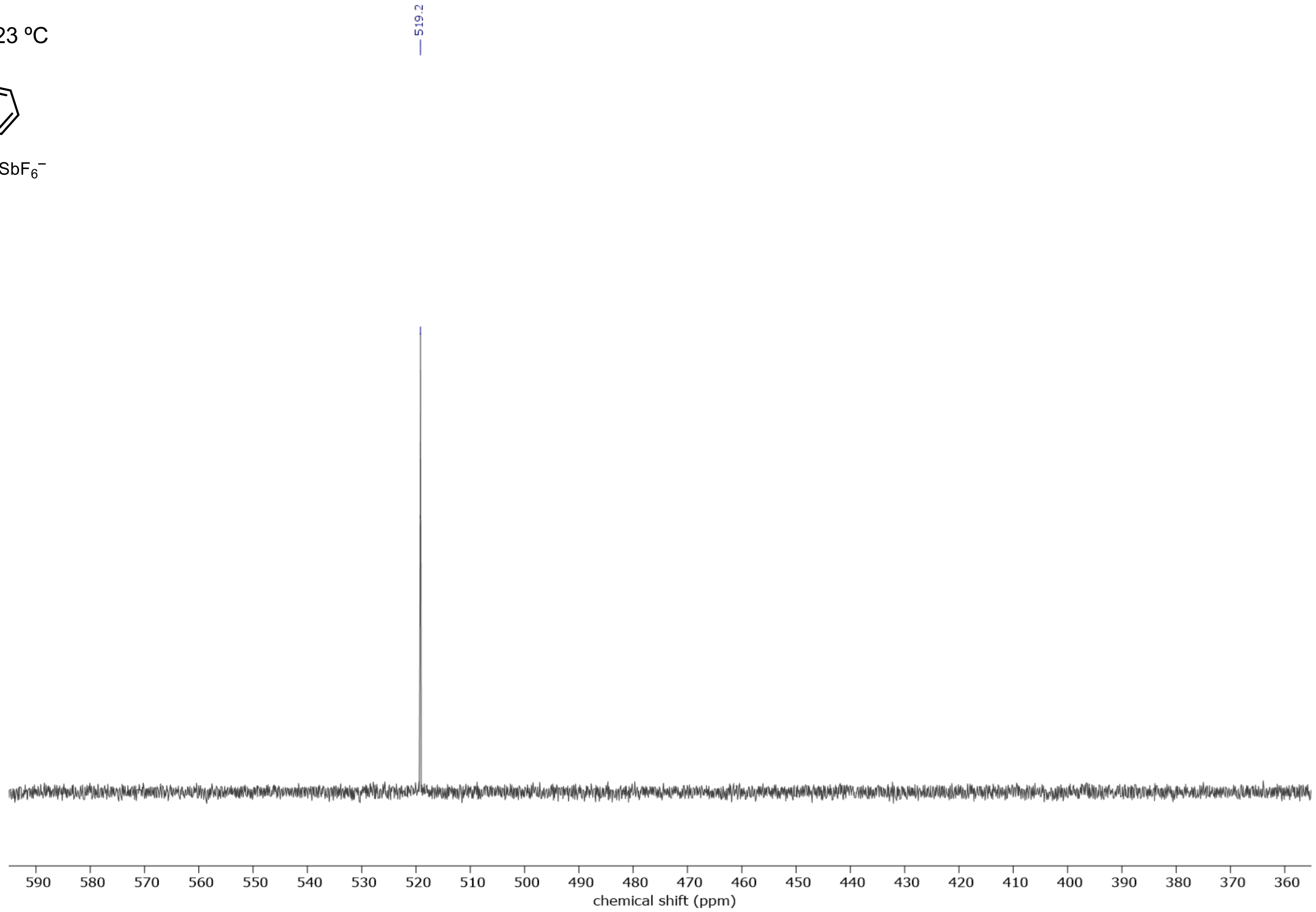

^{1}H NMR OF 5-(*O*-TOLYL)-5*H*-DIBENZO[*B*,*D*]SELENOPHEN-5-IUM HEXAFLUOROANTIMONATE (S16)

DMSO-d6, 23 ºC

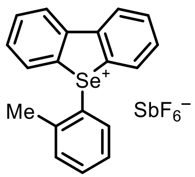

S16

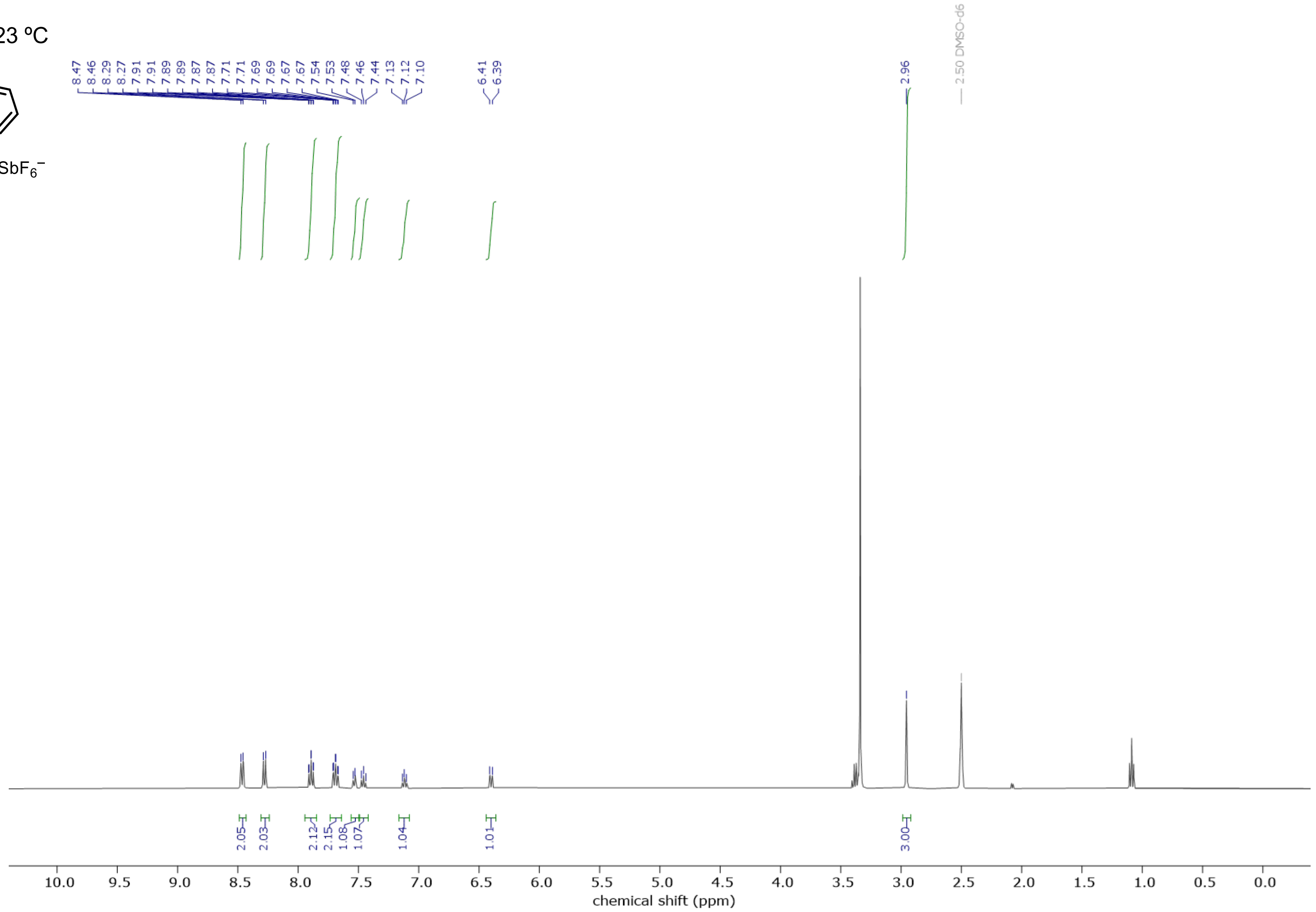

^{13}C NMR of 5-(o-tolyl)-5H-dibenzo[b,d]selenophen-5-ium hexafluoroantimonate (S16)

DMSO-d6, 23 ºC

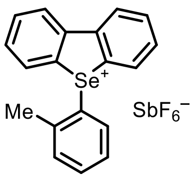

S16

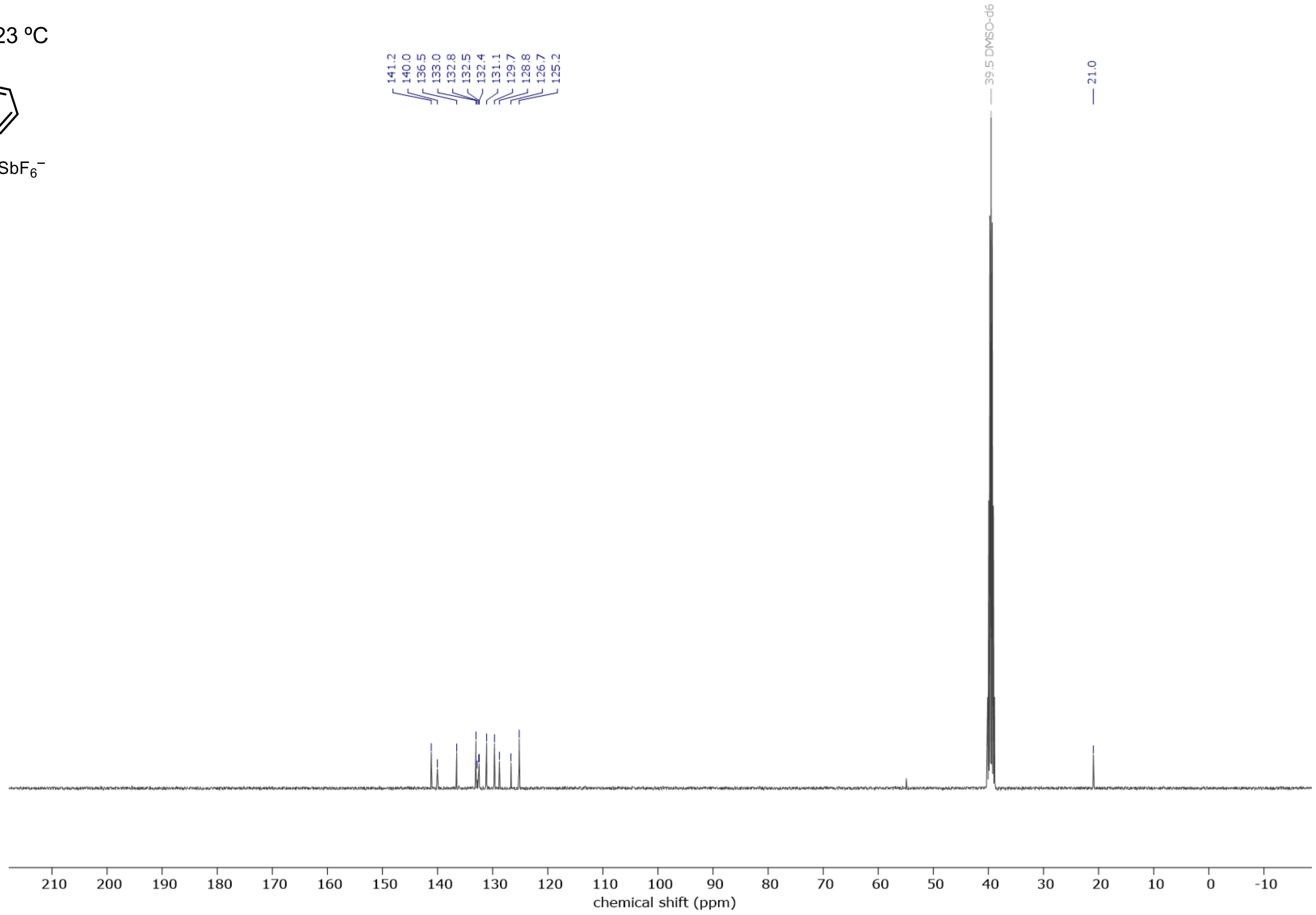

^{19}F NMR OF 5-(*O*-TOLYL)-5*H*-DIBENZO[*B*,*D*]SELENOPHEN-5-IUM HEXAFLUOROANTIMONATE (S16)

DMSO-d6, 23 ºC

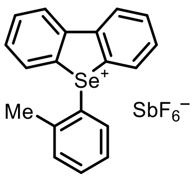

S16

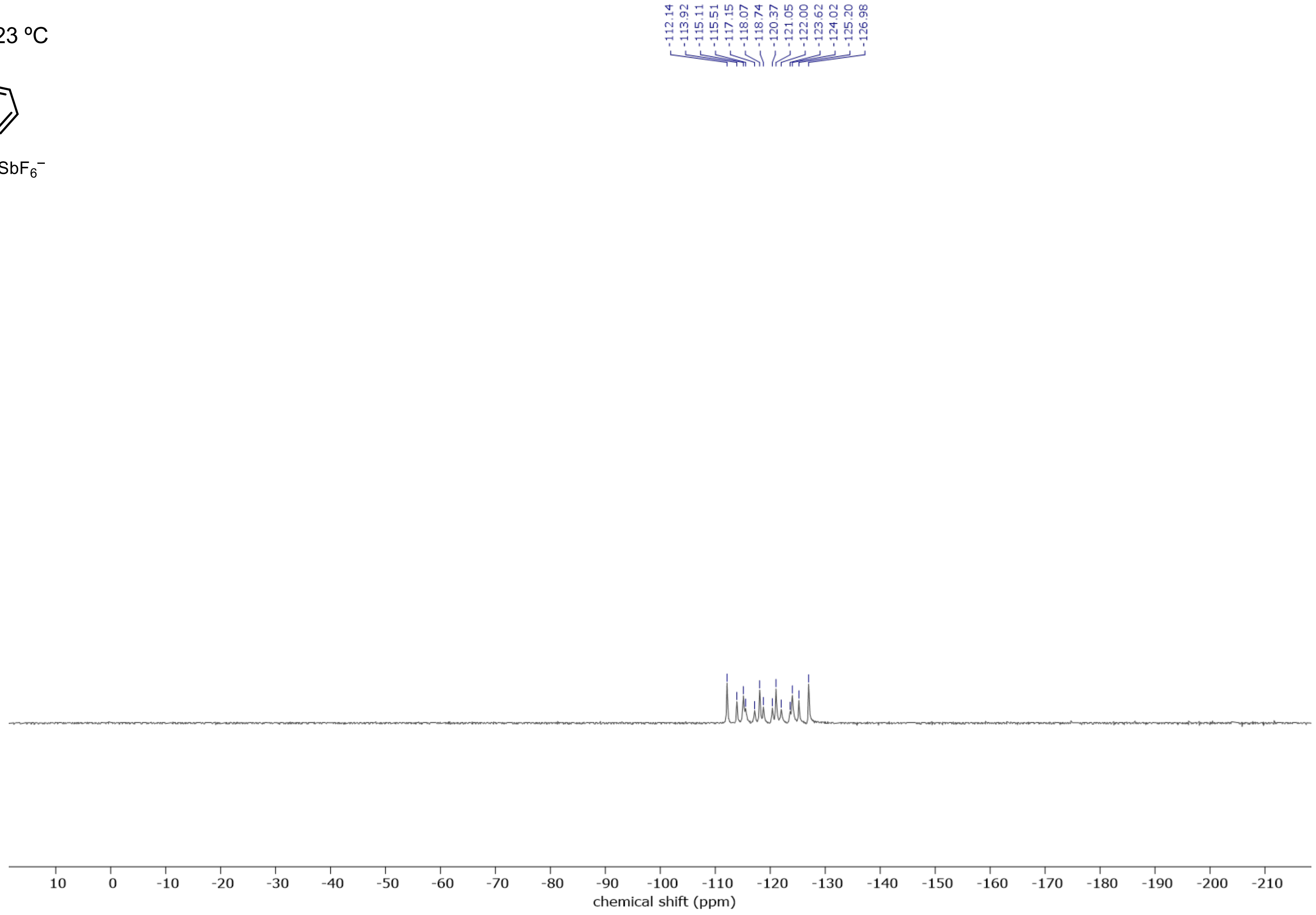

^{77}Se NMR of 5-(*o*-tolyl)-5*H*-dibenzo[*b*,*d*]selenophen-5-ium hexafluoroantimonate (S16)

DMSO-d_6, 23 ºC

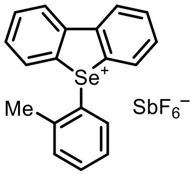

S16

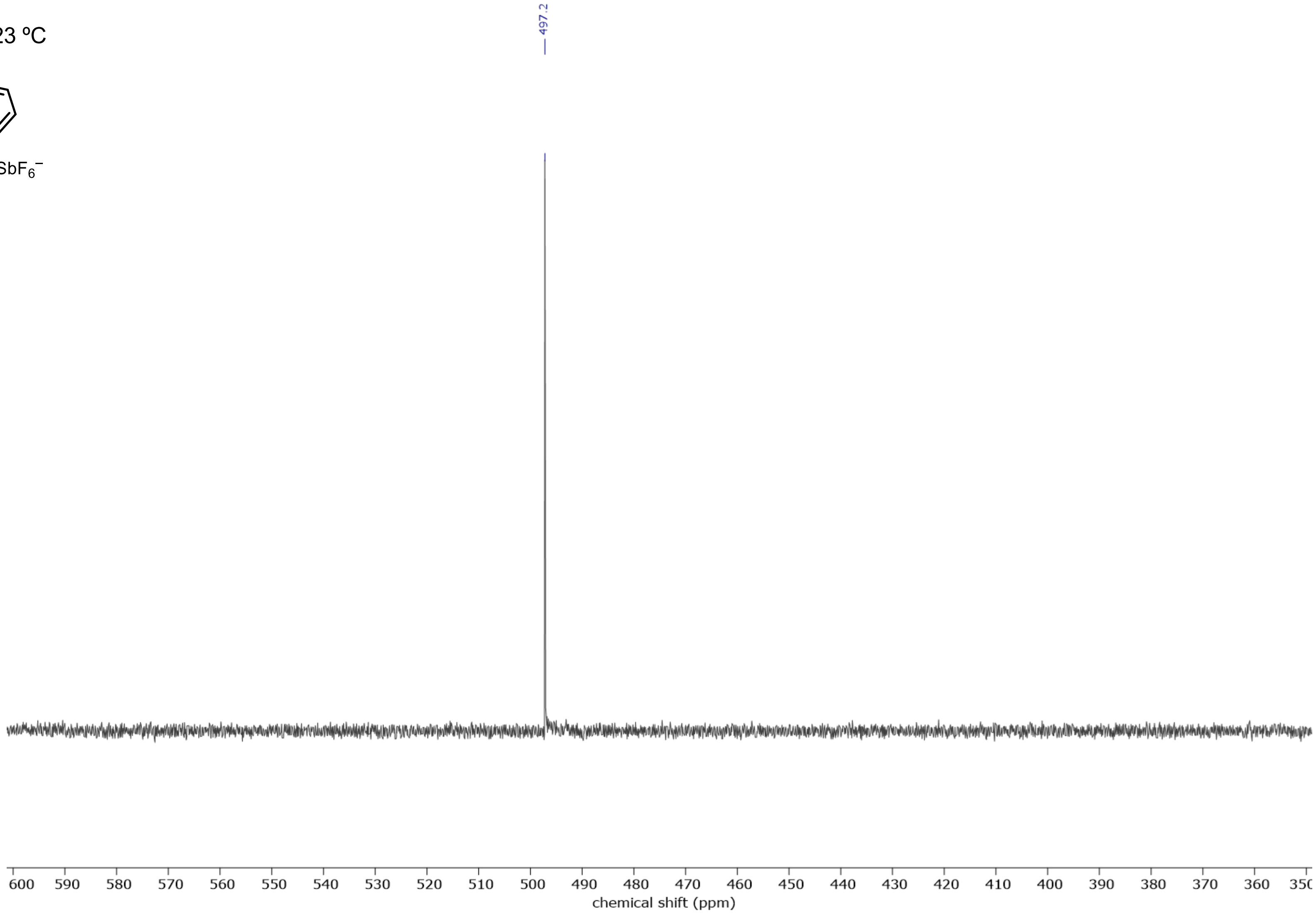

References

[48] R. K. Harris, E. D. Becker, S. M. Cabral De Menezes, P. Granger, R. E. Hoffman, K. W. Zilm, *Pure and Applied Chemistry* **2008**, *80*, 59–84.
[49] S. Stoll, A. Schweiger, *Journal of Magnetic Resonance* **2006**, *178*, 42–55.
[50] G. M. Sheldrick, *Acta Crystallogr A Found Adv* **2015**, *71*, 3–8.
[51] G. M. Sheldrick, *Acta Crystallogr C Struct Chem* **2015**, *71*, 3–8.
[52] O. V. Dolomanov, L. J. Bourhis, R. J. Gildea, J. A. K. Howard, H. Puschmann, *J Appl Crystallogr* **2009**, *42*, 339–341.
[53] C. B. Hübschle, G. M. Sheldrick, B. Dittrich, *J Appl Crystallogr* **2011**, *44*, 1281–1284.
[54] S. Spicher, S. Grimme, *Angew Chem Int Ed Engl* **2020**, *59*, 15665–15673.
[55] P. Pracht, F. Bohle, S. Grimme, *Phys. Chem. Chem. Phys.* **2020**, *22*, 7169–7192.
[56] S. Grimme, *J. Chem. Theory Comput.* **2019**, *15*, 2847–2862.
[57] J.-D. Chai, M. Head-Gordon, *Phys. Chem. Chem. Phys.* **2008**, *10*, 6615–6620.
[58] F. Weigend, R. Ahlrichs, *Phys. Chem. Chem. Phys.* **2005**, *7*, 3297–3305.
[59] E. Cancès, B. Mennucci, J. Tomasi, *The Journal of Chemical Physics* **1997**, *107*, 3032–3041.
[60] A. V. Marenich, C. J. Cramer, D. G. Truhlar, *J. Phys. Chem. B* **2009**, *113*, 6378–6396.
[61] Zobel, J. P., Widmark, P.-O. & Veryazov, V. J. *Chem. Theory Comput.* **2009**, *16*, 278–294.
[62] M. J. Frisch, G. W. Trucks, J. R. Cheeseman, G. Scalmani, M. Caricato, H. P. Hratchian, X. Li, V. Barone, J. Bloino, G. Zheng, T. Vreven, J. A. Montgomery, G. A. Petersson, G. E. Scuseria, H. B. Schlegel, H. Nakatsuji, A. F. Izmaylov, R. L. Martin, J. L. Sonnenberg, J. E. Peralta, J. J. Heyd, E. Brothers, F. Ogliaro, M. Bearpark, M. A. Robb, B. Mennucci, K. N. Kudin, V. N. Staroverov, R. Kobayashi, J. Normand, A. Rendell, R. Gomperts, V. G. Zakrzewski, M. Hada, M. Ehara, K. Toyota, R. Fukuda, J. Hasegawa, M. Ishida, T. Nakajima, Y. Honda, O. Kitao, H. Nakai, Gaussian 16, Revision C.01.
[63] F. Neese, *WIREs Comput Mol Sci* **2022**, *12*, e1606.
[64] Li Manni, G.; Fdez. Galvan, I.; Alavi, A.; Aleotti, F.; Aquilante, F.; Autschbach, J.; Avagliano, D.; Baiardi, A.; Bao, J. J.; Battaglia, S.; & others *J. Chem. Theory Comput.* **2023**, *19*, 6933–6991.
[65] National research council, Ed. , *Prudent Practices in the Laboratory: Handling and Management of Chemical Hazards*, National Academies Press, Washington (D.C.), **2011**.
[66] K. Kafuta, A. Korzun, M. Böhm, C. Golz, M. Alcarazo, *Angew. Chem. Int. Ed.* **2020**, *59*, 1950–1955.
[67] J. Beckmann, A. Duthie, *Z. Anorg. Allg. Chem.* **2005**, *631*, 1849–1855.
[68] M. Björgvinsson, T. Heinze, H. W. Roesky, F. Pauer, D. Stalke, G. M. Sheldrick, *Angew. Chem. Int. Ed.* **1991**, *12*, 1677–1678.
[69] Y. Imada, H. Nakano, K. Furukawa, R. Kishi, M. Nakano, H. Maruyama, M. Nakamoto, A. Sekiguchi, M. Ogawa, T. Ohta, Y. Yamamoto, *J. Am. Chem. Soc.* **2016**, *138*, 479–482.
